# Sixteen cytosolic glutamine synthetase *genes* identified in the *Brassica napus* L. genome are differentially regulated depending on nitrogen regimes and leaf senescence

**DOI:** 10.1093/jxb/eru041

**Published:** 2014-02-24

**Authors:** Mathilde Orsel, Michaël Moison, Vanessa Clouet, Justine Thomas, Françoise Leprince, Anne-Sophie Canoy, Jérémy Just, Boulos Chalhoub, Céline Masclaux-Daubresse

**Affiliations:** ^1^INRA, UMR 1349 Institut de Génétique, Environnement et Protection des Plantes, INRA, Agrocampus Ouest, Université de Rennes 1, F-35653 Le Rheu, France; ^2^INRA, UMR 1345 Institut de Recherche en Horticulture et Semences, F-49071 Beaucouzé, France; ^3^Université d’Angers, UMR 1345 Institut de Recherche en Horticulture et Semences, SFR 4207 QUASAV, PRES L’UNAM, F-49045 Angers, France; ^4^AgroCampus-Ouest, UMR 1345 Institut de Recherche en Horticulture et Semences, F-49045 Angers, France; ^5^UMR1318, INRA, Institut Jean-Pierre Bourgin, RD10, 78026 Versailles cedex, France; ^6^AgroParisTech, Institut Jean-Pierre Bourgin, RD10, 78026 Versailles cedex, France; ^7^Biogemma, Groupe de Recherche Génomique Amont, F-63028 Clermont-Ferrand, France; ^8^INRA-CNRS, Unité de Recherche en Génomique Végétale, 2 rue Gaston Crémieux, CP 5708, 91057 Evry Cedex, France

**Keywords:** Alloploidization, *Brassica napus*, *Brassica oleracea*, *Brassica rapa*, nitrogen metabolism, nitrogen remobilization, senescence.

## Abstract

A total of 16 *BnaGLN1* genes coding for cytosolic glutamine synthetase isoforms (EC 6.3.1.2.) were found in the *Brassica napus* genome. The total number of *BnaGLN1* genes, their phylogenetic relationships, and genetic locations are in agreement with the evolutionary history of *Brassica* species. Two *BnaGLN1.1*, two *BnaGLN1.2*, six *BnaGLN1.3*, four *BnaGLN1.4*, and two *BnaGLN1.*5 genes were found and named according to the standardized nomenclature for the *Brassica* genus. Gene expression showed conserved responses to nitrogen availability and leaf senescence among the Brassiceae tribe. The *BnaGLN1.1* and *BnaGLN1.4* families are overexpressed during leaf senescence and in response to nitrogen limitation. The *BnaGLN1.2* family is up-regulated under high nitrogen regimes. The members of the *BnaGLN1.3* family are not affected by nitrogen availability and are more expressed in stems than in leaves. Expression of the two *BnaGLN1.5* genes is almost undetectable in vegetative tissues. Regulations arising from plant interactions with their environment (such as nitrogen resources), final architecture, and therefore sink–source relations *in planta*, seem to be globally conserved between *Arabidopsis* and *B. napus*. Similarities of the coding sequence (CDS) and protein sequences, expression profiles, response to nitrogen availability, and ageing suggest that the roles of the different *GLN1* families have been conserved among the Brassiceae tribe. These findings are encouraging the transfer of knowledge from the *Arabidopsis* model plant to the *B. napus* crop plant. They are of special interest when considering the role of glutamine synthetase in crop yield and grain quality in maize and wheat.

## Summary

*BnaGLN1* coding sequences and expression profiles in response to nitrogen availability and ageing are essentially conserved compared with *A. thaliana*, suggesting that the roles of *GLN1* families are conserved among the Brassiceae tribe.

## Introduction

Winter oilseed rape (*Brassica napus* L.) is the dominant oilseed crop in northern Europe, and nitrogen (N) fertilization is the main operational cost for farmers (50% of the total cost of production). When compared with other crops, oilseed rape is characterized by low nitrogen use efficiency (NUE) (Rathke *et al.*, 2006). Despite a high N-uptake efficiency (Laine *et al.*, 1993), only half the N originating from fertilizer application is recovered in the seeds (Schjoerring *et al.*, 1995). Oilseed rape is characterized by early leaf shedding and unusual high N loss in senescing falling leaves. The plant can lose up to 15% of its entire N content in this way (Rossato *et al.*, 2001). Leaf senescence generally corresponds to the mobilization of N reserves from source leaves to sink organs such as seeds (Masclaux-Daubresse *et al.*, 2008). In oilseed rape, it has been shown that N can be remobilized from senescing leaves to expanding leaves at the vegetative stage (sequential senescence) as well as from senescing leaves to seeds at the reproductive stage (monocarpic senescence) (Malagoli *et al.*, 2005).

The rate of senescence and remobilization of leaf N are related to the N nutrition status of the plant and to source–sink relations (Masclaux *et al.*, 2000). N remobilization towards new developing organs is largely dependent on senescence-related catabolism events and translocation of leaf N compounds. Amino acids derived from protein catabolism are exported via the phloem to growing parts of the plant; the concentration of amino acids in the phloem sap increases during leaf senescence (Herrera-Rodriguez *et al.*, 2006, 2007; Masclaux-Daubresse *et al.*, 2006). In many species including *B. napus*, aspartate, glutamate, and their corresponding amides are the principal forms of amino N compounds transported in the phloem and play a key role in rendering N available for remobilization from senescing leaves (Tilsner *et al.*, 2005). Enzymes involved in the biosynthesis and metabolism of amino acids destined for phloem loading are of special interest.

In plants, glutamine synthetase (GS; EC 6.3.1.2) is a key enzyme which catalyses an ATP-dependent conversion of glutamate to glutamine using ammonium derived from primary N uptake and various internal N recycling pathways including catabolic release of ammonium during senescence (Bernard and Habash, 2009). In a large variety of plants, induction of cytosolic glutamine synthetase (GS1) genes has been detected during leaf senescence, while chloroplastic synthetase isoenzyme (GS2) expression decreases (Masclaux *et al.*, 2000; Guo *et al.*, 2004; Martin *et al.*, 2006). It has been proposed that in young photosynthetic leaves, the chloroplastic isoenzyme GS2 is mainly involved in the assimilation of ammonium provided by nitrate reduction and photorespiration through the GS/GOGAT cycle (Masclaux *et al.*, 2001). In old senescing leaves, as chloroplasts are breaking down, glutamine to be exported would be synthesized by the newly expressed cytosolic GS1 isoforms (Masclaux-Daubresse *et al.*, 2006).

The importance of GS1 in N management, growth rate, leaf senescence onset and severity, yield, and grain filling has been confirmed by co-location of quantitative trait loci (QTLs) and functional genomics approaches mainly performed on maize (Hirel *et al.*, 2001; Martin *et al.*, 2006) and rice (Tabuchi *et al.*, 2005). In maize, *Gln1.4* is up-regulated during senescence (Martin *et al.*, 2005). The *Gln1.4* knockout mutation led to a dysfunction in N export and a sharp reduction of kernel yield (Martin *et al.*, 2006). GLN1.4 was proposed to be involved in re-assimilation of ammonium released during leaf protein degradation. In rice, mutants lacking *OsGS1;1* are severely impaired in growth rate and grain filling, and glutamine levels in mutant leaf blades are reduced (Tabuchi *et al.*, 2005). As the gene product is located in companion cells and parenchyma cells of leaf tissues, it has been proposed that OsGS1;1 is responsible for generation of glutamine for remobilization via the phloem.

To date, all studies on plant genomes have revealed multigenic families coding for several GS1 isoforms. In rice, three *GLN1* genes have been identified, with seven in wheat, five in maize, and five in *Arabidopsis thaliana*. Transcriptomic data showed that three *A. thaliana* genes, *AtGLN1.1*, *AtGLN1.2*, and *AtGLN1.4*, are induced during leaf ageing (Guo *et al.*, 2004). Promoter::GFP (green fluoresacent protein) fusions were used to investigate *AtGLN1* gene expression in roots. *AtGLN1.1* was localized at the root surface layer, whereas *AtGLN1.2*, *AtGLN1.3*, and *AtGLN1.4* were expressed in root vascular tissues (Ishiyama *et al.*, 2004). Detailed expression of *AtGLN1* in leaves was only reported for *AtGLN1.2* that is induced in root and leaf tissues under a high N regime and is mainly expressed in veins and mesophyll cells in older leaf tissues (Lothier *et al.*, 2011). In veins, AtGLN1.2 protein was localized in the companion cells. The knock-out mutant phenotype led to the conclusion that *AtGLN1.2* is essential for N assimilation under ample nitrate supply and for ammonium detoxification (Lothier *et al.*, 2011). For all plant species, it is clear that not all GS1 isoforms participate equally in N management and remobilization. Regulation of expression is then a key clue towards the identification of *GLN1* genes potentially involved in N remobilization.

Accumulation of GS1 and a decrease in GS2 polypeptides were observed in *B. napus* leaves after onset of leaf senescence (Ochs *et al.*, 1999). Up to now, four closely related genes coding for GS1 isoenzymes, *BnGSR1-1*, *BnGSR1-2*, *BnGSR2-1*, and *BnGSR2-2*, have been identified using *B. napus* root-derived expressed sequence tag (EST) libraries (Ochs *et al.*, 1999). Analysis of different tissue types has also revealed that these genes are expressed in senescing leaves (Buchanan-Wollaston and Ainsworth, 1997). Recent studies of Brassicaceae genomes show that the genome of *B. napus*, which is a recent allotetraloid (2*n*=4*x*=38, AACC) arising from the natural hybridization of monogenomic diploids *Brassica rapa* (AA) and *Brassica oleracea* (CC) (Nagaharu, 1935), contains additional genes coding for GS1 isoenzymes. Analysis of *Brassica* lineage genomes revealed that a whole-genome triplication occurred shortly after their divergence from *Arabidopsis* (Parkin *et al.*, 2005). Therefore, gene families are more frequent, larger, and more complex in *B. napus* than in *A. thaliana*. Brassicaceae genome sequences are also highly conserved and many synthenic regions have been identified (Paterson *et al.*, 2001; Parkin *et al.*, 2005; Schranz *et al.*, 2006), allowing the identification of ‘true’ orthologous genes between *A. thaliana* and *B. napus*.

In the present study, advantage is taken of the Brassicaceae genome structure and of its recent sequencing (unpublished) in order to identify all *BnaGLN1* genes coding for GS1 isoenzymes. It is demonstrated that they are differentially regulated depending on tissue type, senescence, and N availability. The potential role of the *BnaGLN1* genes in N remobilization during leaf senescence, the impact of whole-genome duplications and merging on the evolution of the *GLN1* multigenic family in the Brassiceae tribe, and strategies based on knowledge transfer from *A. thaliana* to crop plants are discussed.

## Materials and methods

### *Brassica* gene identification

Sequence searches by similarity to *A. thaliana AtGLN1* coding sequences were performed in the GenBank and Genoplante databases using the BLAST algorithm (Altschul and Lipman, 1990) and the *A. thaliana AtGLN1* coding sequences *AtGLN1.1* (NM_123119, At5g37600), *AtGLN1.2* (NM_105291, At1g66200), *AtGLN1.3* (NM_112663, At3g17820), *AtGLN1.4* (NM_121663, At5g16570), and *AtGLN1.5* (NM_103743, At1g48470). The BlastN option was used to recover *B. napus*, *B. rapa*, and *B. oleracea* ESTs and complete mRNAs as well as genomic sequences of *B. napus* and *B. rapa* (Cheng *et al.*, 2011; http://brassicadb.org/brad/). Contigs of ESTs were built using the CAP3 algorithm (Huang and Madan, 1999) and validated through multiple sequence alignments with ESTs and *AtGLN1* coding sequences, allowing a manual proofreading. Alignments were generated with the ClustalW algorithm (Thompson *et al.*, 1994) using Clustalw-sequence and Clustalw-profil options available at the MOBYLE platform (http://mobyle.pasteur.fr). ESTs included in each contig are listed in Supplementary Data File 1 available at *JXB* online.

*BnaGLN1* contigs were then enriched and/or their coding sequence completed with new cDNA sequences: clones from Genoplante oilseed rape cDNA libraries and the ADIZ-MPIZ 021 library corresponding to ESTs were sequenced when available (Supplementary Table S1 at *JXB* online). When the coding sequence from cDNAs was incomplete or no clone was available, specific primers were designed to clone the total or missing coding region (Supplementary Table S2). The amplified fragments were cloned into pGEM^®^-T Easy plasmids (Promega) according to the recommendation of the supplier, and sequenced. Universal T6 and SP7 primers, as well as specific primers were used to sequence the clones on the positive and negative strands (Supplementary Table S3). All DNA sequencing was performed by Cogenics (Grenoble, France) and sequences were submitted to GenBank (accession numbers are given in Supplementary Table S1).

### Sequence analysis

A global alignment of coding sequences from mRNA, inferred coding sequences, or newly created contigs from ESTs was generated with ClustalW (Thompson *et al.*, 1994). Distance matrixes were computed using the Dnadist algorithm with a Kimura 2 nucleotide substitution model, and bootstrap analysis was performed with 1000 iterations. A consensus unrooted tree was then generated using the Neighbor–Joining method. All algorithms are contained in the Phylip 3.67 package available at the MOBYLE platform (http://mobyle.pasteur.fr). The NCBI Conserved Domain Database (Marchler-Bauer *et al.*, 2011) was searched with translated *B. napus* and *A. thaliana* GLN1-coding sequences. A multiple protein sequence alignment was generated with the ClustalW algorithm.

### Genetic mapping and genome or chromosome assignment

Genetic mapping of *BnaGLN1* genes was realized using three different *B. napus* double haploid (DH) populations. The Stellar×Drakkar (SD), Darmor×Samouraï (DS), and Darmor-*bzh*×Yudal (DY) populations consist, respectively, of 94, 134, and 445 genotype DH lines described by Lombard and Delourme (2001) and Delourme *et al.* (2006). Gene-specific primers were designed and selected for a presence/absence polymorphism in one of the three populations (Supplementary Table S2 at *JXB* online). Linkage analyses were performed as previously described by Auger *et al.* (2009) using MAPMAKER/EXP version 3.0b (Lander *et al.*, 1987) and framework maps constructed in Lombard and Delourme (2001) and updated in Delourme *et al.* (2006). *BnaGLN1* genes were assigned to a linkage group using the ASSIGN command (LOD threshold=8.0) and then placed in the most confident interval with the PLACE command (LOD threshold=2.0). Recombination frequencies were converted into centiMorgans (cM) with the Kosambi function (Kosambi, 1944).

*BnaGLN1* gene assignment to A or C *Brassica* genomes was performed using a panel of diverse *B. napus*, *B. oleracea*, and *B. rapa* genotypes available in the authors’ group. The panel contains genomic DNA from *B. napus* genotypes Darmor-*bzh*, Yudal, Stellar, Drakkar, Samouraï, aburamassari, Aviso, Tenor, Express, Montego; *B. rapa* genotypes Z1, C1.3, Chiifu; and *B. oleracea* genotypes HDEM and C102. This panel was PCR screened with specific but not polymorphic gene markers (Supplementary Table S2 at *JXB* online).

Chromosome assignment for *BnaC.GLN1* genes was realized using monosomic and polysomic addition lines carrying one or several additional C chromosomes from Darmor-*bzh*. Lines were selected from a cross between *B. napus* Darmor-*bzh* and *B. oleracea* C1.3 (A.M. Chèvre and F. Eber, INRA Rennes, unpublished results). Genomic DNA from these lines was PCR screened with specific but not polymorphic markers (Supplementary Table S2 at *JXB* online).

### Plant material and growth conditions

*Brassica napus* L. plants from the Darmor-*bzh* genotype were grown in a greenhouse at INRA Versailles, France. Seeds were sown on sand and watered with nutritive solution during 2 weeks in order to allow germination and subsequent growth of plantlets. When the first two true leaves appeared, plantlets were transferred into pots containing sand and were separated into two groups with contrasting N fertilization regimes (LN for low nitrate and HN for high ntrate, 0.4mM and 8mM NO_3_^–^, respectively) according to Albert *et al.* (2012). At 56 d after sowing, four plants of each nutrition regime were harvested and sampled. For each plant, all leaf ranks were collected: primary and secondary veins were separated from the rest of the leaf, described as the limb. All fresh samples were frozen immediately in liquid nitrogen and stored at –80 °C.

*Brassica napus* L. plants from the Express genotype were grown in field trials in 2009–2010, in Le Rheu (Brittany), France. Seeds were sown on 7 September 2009 with plant density set at 40 plants m^–2^. The field trial was conducted with contrasting N fertilization regimes. Plant N status was monitored over the vegetative stage by calculating the nitrogen nutrition index (NNI) (Colnenne *et al.*, 1998). The balance-sheet method was used as a decision tool for N fertilization, setting the potential yield for LN and HN regimes at 20 q ha^–1^ and 35 q ha^–1^, respectively (Makowski *et al.*, 2005). LN plants did not receive N fertilizer, while the HN plants received a total input of 110kg N ha^–1^ spread at two different times (12 February and 19 March 2010). LN and HN plants were harvested, respectively, on 9 and 12 April, at the beginning of the flowering period when half the plants of the plot had their first flowers open on the main stem (F1, or 60 on the BBCH scale), and 400 degree-days later (base 0) on 17 and 20 May at the beginning of the seed filling period (G2, or 71–73 on the BBCH scale).

Plants from 0.5 m^2^ per plot (~20 plants) were harvested in the early morning and sampled during the subsequent hour. For each batch, plants were ranked according to their length and developmental stage; the six median plants were selected for sampling. On the main stem, the lowest leaf starting to yellow (Old) and the highest leaf at least 5cm long (Young) were selected, the petiole and main vein were removed, and limbs were sampled. The stems above young and old leaf insertions were selected and sampled over 2cm and 4cm, respectively. All fresh samples were frozen immediately in liquid nitrogen and stored at –80 °C.

### Nucleic acid manipulation

PCRs were conducted in a 20 μl mix containing 2–10ng of DNA, 0.25mM dNTPs (Promega), 0.5 μM of each primer (Eurogentec, Angers, France), and 0.5U of *Taq* DNA polymerase (Promega) in the appropriate buffer supplemented with 2.5mM MgCl_2_. The amplification program was run on a PTC-225 thermocycler (MJ Research, Waltham, MA, USA) with the following conditions: 35 cycles of denaturation at 94 °C for 30 s (3min for the first cycle), annealing at 55–60 °C for 30 s, and elongation at 72 °C for 1–2min (10min for the last cycle).

Total RNAs were extracted with the SV Total RNA Isolation System (Promega) from 70mg fresh weight (FW) of ground frozen tissue. First, samples were homogenized in the RNA lysis buffer (400 μl) using TissueLyserII from Qiagen. Then, all cell debris was eliminated by filtering the lysate through a ‘Nucleospin 96 RNA filter Plate’ (Macherey-Nagel). The manufacturer’s protocol was then followed. In order to remove any remaining DNA traces, 1.5 μg of RNA was treated with DNase using the Turbo DNA-free kit (Ambion) according to the manufacturer’s protocol. The quality of RNA was assessed by an electrophoresis on agarose gel (1.3%, w/v), and the absence of DNA contamination in samples was confirmed by PCR amplification. First-strand cDNA was synthesized using 2 μg of total RNA with oligo(dT)_12–18_ primers and Superscript III reverse transcriptase (Invitrogen) according to the manufacturer’s instructions. Two independent reverse transcription reactions were performed as technical replicates. cDNA samples were diluted 26-fold with sterile water before use.

### Gene expression analysis with qPCR

Quantitative PCRs (qPCRs) were set up with the LightCycler 480 SYBR Green I Master mix (Roche Diagnostics) and 4 μl of diluted cDNA in a final volume of 12 μl. The concentration of specific forward and reverse primers was set at 0.42 μM. qPCRs were run on a Light Cycler LC480 (Roche Diagnostics) under the following conditions: an initial step at 95 °C for 10min, then 50 cycles of 95 °C for 10 s, 60 °C for 10 s, and 72 °C for 20 s.

Primer pairs were designed for short and specific amplification of individual members of the multigenic *BnaGLN1* family and five reference genes (Supplementary Table S4 at *JXB* online). Identification of *B. napus* reference genes for reverse transcription–qPCR analysis was based on EST sequence similarity to *A. thaliana* genes with verified expression stability over a wide range of tissues and growing conditions. *Arabidopsis thaliana* genes were selected from among the list established by Czechowski *et al.* (2005). Their respective coding sequences were used to retrieve highly similar *B. napus* ESTs from GenBank using the BlastN algorithm. One *B. napus* EST per reference gene was selected to design qPCR primers (Supplementary Table S4).

For each run, single product amplification was confirmed by melt curve analysis. PCR products from each primer pair and genotype were sequenced in a preliminary analysis. The amplification efficiency was assessed for each genotype, with each primer pair using a dilution curve method over six orders of magnitude, on a pool of cDNAs from different tissues and modalities. Selected primer pairs have efficiencies >1.8.

The results reported were obtained from four biological replicates and two reverse transcriptions as technical replicates. All samples, including reverse transcription and biological replicates, were run at the same time for each primer pair. Raw fluorescence data were collected and analysed with the R package ‘qpcR’ (Ritz and Spiess, 2008). The ‘pcrbatch’ function was used to select sigmoid models for the fluorescence curves and then allowing the determination of the intrinsic amplification efficiency (sig.eff) and threshold cycle (sig.CpD2) at the second derivative maximum (Rutledge, 2004). For each run, cDNA relative quantity (RQ) was calculated using the efficiency mean value from the two technical and the four biological repetitions (mean sig.eff), and the run-specific threshold cycle as: RQ=1/(mean sig.eff) (sig.CpD2).

The most stable reference genes were selected using the GeNorm method from Vandesompele *et al.* (2002) available through the ‘SLqPCR’ R package (Dr Matthias Kohl SIRS-Lab GmbH). The four reference genes *BnaX.PTB*, *BnaX.SAND*, *BnaX.PP2A*, and *BnaX.UBC21* from the five tested were retained with an average M value equal to 0.33. For each cDNA sample, a normalization factor (NF) was calculated as the geometrical mean of RQ from the four selected genes, and normalized RQ (NRQ) was then calculated as NRQ=RQ/NF. The mean of both technical replicates was then calculated for each sample.

## Results

### Identification of *GLN1* coding sequences in EST databases of *Brassica napus* and its progenitors *Brassica oleracea* and *Brassica rapa*

**Fig. 1.**
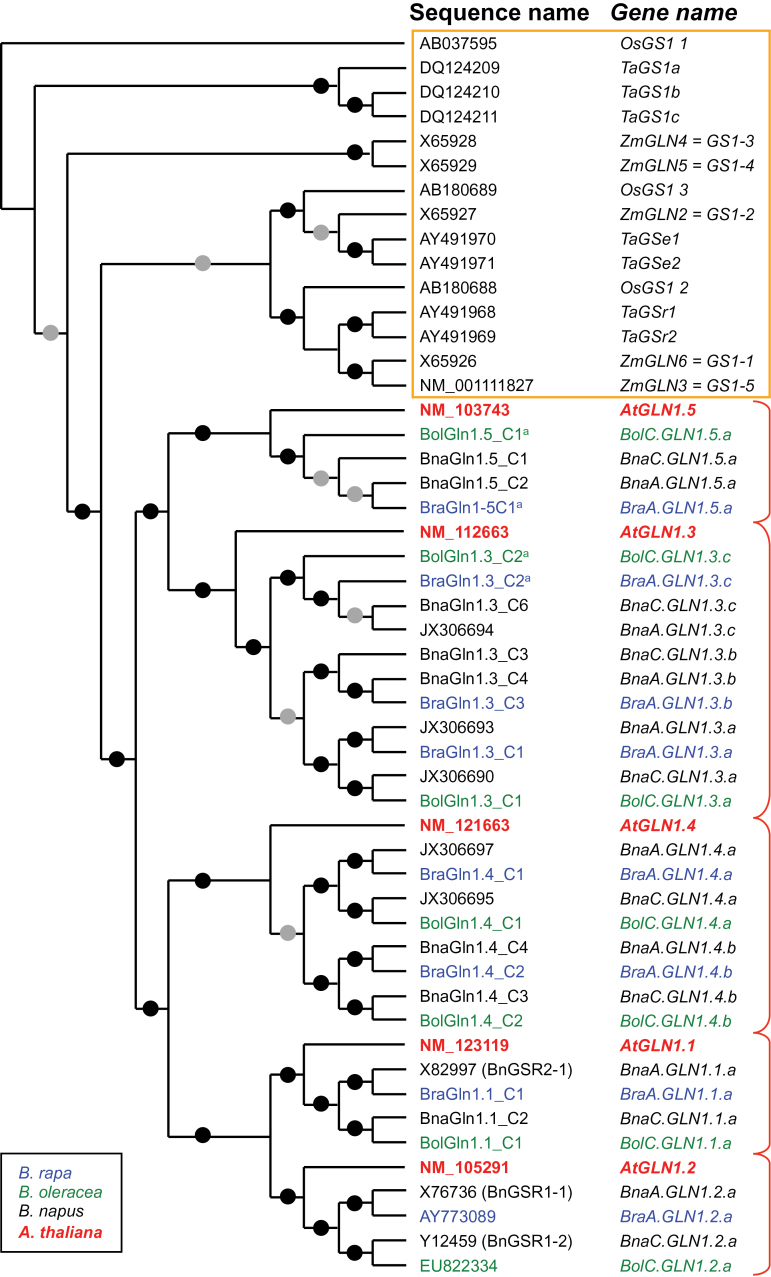
Cytosolic glutamine synthetase (GS1) phylogenetic tree. DNA coding sequences (CDS) were aligned using Clustal. The distance matrix was computed using Dnadist with a Kimura 2 nucleotide substitution model (bootstrap analysis, 1000 iterations). A consensus unrooted tree was then generated using the Neighbor–Joining method from the Phylip 3.67 package. Black and grey dots indicate bootstrap values >90% and 50%, respectively. All programs are available at www.mobyle.pasteur.fr. ^a^Incomplete CDS sequence when compared with the *A. thaliana* reference sequence.

A total of 588 *B. napus* ESTs, 126 *B. rapa* ESTs, and 36 *B. oleracea* ESTs highly similar to one or several of the five *AtGLN1.1*–*AtGLN1.5* mRNA-coding sequences from *A. thaliana* were isolated from public and private Genoplante databases (Supplementary Data File S1 at *JXB* online). Sequence assembly and alignments with *AtGLN1* mRNA sequences revealed different groups of transcripts that allowed ESTs to be grouped and 16 individual contigs that might correspond to different *BnaGLN1* genes of *B. napus* to be extracted (Supplementary Data File S2). Eight contigs for *B. rapa* and seven contigs for *B. oleracea* were also isolated (Supplementary Data Files S1, S2). The *BnaGLN1*, *BraGLN1*, and *BolGLN1* contigs from *B. napus*, *B. rapa*, and *B. oleracea*, respectively, show high levels of sequence similarity with all the five *AtGLN1* genes (Table 1). The analysis of similarity levels and a phylogenetic tree (Fig. 1) allowed homologous sequences for each *AtGLN1* mRNA to be clearly identified in *B. napus*, *B. rapa*, and *B. oleracea*. The phylogenetic tree reveals that the *GLN1* mRNA sequences are divided into two distinct groups for monocotyledonous (wheat, maize, and rice) and dicotyledonous species. The Brassicaceae sequences are divided into five clusters, each one including one *A. thaliana* and one or several *B. napus*, *B. oleracea*, and *B. rapa* sequences (Fig. 1). Each *B. napus* sequence is closely related to one sequence from either *B. oleracea* or *B. rapa* progenitors, illustrating the ancestral relationship with the A and C *Brassica* genomes. Each *B. napus* sequence is also closely related to one of the five *A. thaliana AtGLN1* mRNA sequences, allowing the identification of homeologous related sequences between the four species. The contigs were then named according to the *AtGLN1.x* (*x* from 1–5) gene with the highest sequence similarity. Since for each *AtGLN1.x* sequence several *BnaGLN1* sequences were found, contig names were also extended by a copy number (Cn): BnaGLN1.x_Cn (Tables 1, 12; Fig. 1). Similarly, the names of the *B. rapa* and *B. oleracea* contigs follow the same rules (BraGLN1.x_Cn and BolGLN1.x_Cn, respectively; Table 2).

**Table 1.** *BnaGLN1 proteins and nucleotide sequence identities*The percentage identity within the *BnaGLN1* and *AtGLN1* family between contig nucleotide coding sequences (top right), and between translated protein sequences (bottom left). Contig names are used for the column index and protein names for the line index.

	*AtGLN1.1*	BnaGLN1.1_C1	BnaGLN1.1_C2	*AtGLN1.2*	BnaGLN1.2_C1	BnaGLN1.2_C2	*AtGLN1.3*	BnaGLN1.3_C2	BnaGLN1.3_C1	BnaGLN1.3_C4	BnaGLN1.3_C3	BnaGLN1.3_C5	BnaGLN1.3_C6	*AtGLN1.4*	BnaGLN1.4_C1	BnaGLN1.4_C2	BnaGLN1.4_C4	BnaGLN1.4_C3	*AtGLN1.5*	BnaGLN1.5_C2	BnaGLN1.5_C1
AtGLN1.1	–	92.7	93.1	87.5	87.5	88.0	78.8	78.2	77.7	76.6	77.1	77.5	77.0	78.4	79.9	79.7	78.8	78.9	74.7	74.1	73.9
BnaA.GLN1.1.a	94.4	–	**97.9**	87.1	87.7	88.0	78.3	77.4	77.2	76.3	77.1	77.0	76.8	79.1	81.3	81.0	79.8	79.5	74.4	73.1	73.2
BnaC.GLN1.1.a	94.4	**98.3**	–	87.1	87.4	88.0	78.2	77.5	77.0	76.0	76.8	77.2	77.0	78.7	81.1	80.8	79.6	79.4	74.8	73.5	73.6
AtGLN1.2	92.2	92.2	91.1	–	91.4	91.8	78.1	77.4	77.1	75.9	77.3	77.4	76.8	78.8	79.2	79.6	78.1	78.3	74.4	73.6	73.7
BnaA.GLN1.1.b	92.7	93.9	92.7	**95.5**	–	**99.1**	77.7	77.1	77.0	76.5	77.4	76.8	76.4	79.4	79.6	79.8	78.1	78.7	73.6	73.2	73.3
BnaC.GLN1.1.b	93.3	93.3	93.3	**95.5**	**99.4**	–	78.1	77.5	77.4	76.4	77.4	77.3	76.8	79.9	80.1	80.2	78.5	79.1	74.0	73.4	73.5
AtGLN1.3	85.8	86.3	85.8	83.8	84.9	84.9	–	90.8	90.3	89.3	89.9	90.5	90.4	76.1	76.9	76.5	76.0	76.2	80.7	80.0	80.0
BnaA.GLN1.3.a	84.6	85.5	85.2	83.8	84.4	84.4	94.1	–	**97.1**	92.8	93.7	93.2	93.0	75.4	76.3	75.8	75.1	75.4	81.0	81.2	81.4
BnaC.GLN1.3.a	84.6	85.5	85.2	83.8	84.4	84.4	94.1	**100**	–	93.3	94.2	93.5	93.1	75.8	76.5	76.0	75.0	75.5	80.3	80.4	80.5
BnaA.GLN1.3.b	83.2	84.4	83.5	83.0	84.1	83.5	93.0	**96.1**	**96.1**	–	**97.7**	93.2	93.2	75.9	76.5	76.0	75.7	76.0	80.1	80.0	80.1
BnaC.GLN1.3.b	83.2	84.4	83.5	83.0	84.1	83.5	93.0	**96.1**	**96.1**	**100**	–	94.0	93.9	76.2	76.9	76.5	75.9	76.1	80.3	80.2	80.3
BnaA.GLN1.3.c	83.8	85.2	84.9	82.7	83.2	83.2	93.9	**95.8**	**95.8**	94.7	**94.7**	–	**98.6**	76.6	76.4	76.3	75.9	76.0	80.1	80.4	80.3
BnaC.GLN1.3.c	83.2	84.6	84.4	82.1	82.7	82.7	93.9	**95.3**	**95.3**	94.7	94.7	**99.4**	–	76.5	76.4	76.3	76.2	76.0	80.6	80.8	80.7
AtGLN1.4	88.5	87.4	87.7	86.6	87.4	88.0	83.0	83.0	83.0	82.4	82.4	82.4	82.7	–	92.0	91.6	91.8	92.0	74.7	73.8	73.6
BnaA.GLN1.4.a	88.5	88.3	88.5	88.0	88.3	88.8	83.2	83.8	83.8	82.1	82.1	82.4	82.7	**95.3**	–	**97.4**	93.6	93.0	74.3	73.7	73.7
BnaC.GLN1.4.a	88.0	87.7	88.0	87.7	88.0	88.5	83.0	83.5	83.5	81.8	81.8	82.1	82.4	**95.0**	**99.2**	–	92.9	92.8	74.3	73.6	73.7
BnaA.GLN1.4.b	89.1	88.0	88.3	88.0	88.5	89.1	83.2	83.5	83.5	81.8	81.8	82.1	82.4	**96.4**	**96.6**	**96.4**	–	**97.3**	73.7	72.6	72.7
BnaC.GLN1.4.b	88.5	88.0	88.3	87.7	88.5	89.1	83.8	83.8	83.8	82.4	82.4	82.4	82.7	**96.6**	**96.4**	**96.1**	**99.2**	–	74.4	72.8	73.1
AtGLN1.5	78.8	80.4	80.4	78.8	79.1	79.6	83.0	83.2	83.2	82.1	82.1	82.7	82.4	79.3	79.6	79.3	79.3	79.6	–	91.3	91.1
BnaA.GLN1.5.a	79.6	80.4	80.4	78.8	79.1	79.6	83.8	83.5	83.5	83.2	83.2	83.5	83.2	79.6	79.1	78.8	79.1	79.3	**95.0**	–	**98.7**
BnaC.GLN1.5.a	79.6	80.4	80.4	78.8	79.1	79.6	83.8	83.5	83.5	83.2	83.2	83.5	83.2	79.6	79.1	78.8	79.1	79.3	**95.0**	**100**	–

Light grey indicates percentage identity between *A. thaliana* and *B. napus* orthologous genes.

Dark grey indicates identity between *B. napus* homeologous genes.

Identities >95% are in bold.

**Table 2.** *Names of contigs of ESTs, mRNA, and* AtGLN1 *homologous genes in* Brassica napus, B. oleracea*, and* B. rapaThe names of genes encoding each contig were assigned according to the Ostergaard and King (2008) nomenclature, taking into account A or C genome location, and the closest *AtGLN1* sequence homology.

*A. thaliana*	*Brassica napus*		*Brassica oleracea*		*Brassica rapa*	
Gene name	Name of contig of ESTs; mRNA name	Gene name	Name of contig of ESTs; mRNA name	Gene name	Name of contig of ESTs; mRNA name	Gene name (BRAD name; LG)^*a*^
*AtGLN1.1* (At5g37600)	BnaGLN1.1_C1; X82997 (BnGSR2.1)	*BnaA.GLN1.1.a*			BraGLN1.1_C1	BraA.GLN1.1.a (Bra028132; A04)
	BnaGLN1.1_C2; Y12460 (BnGSR2.2)^*b*^	*BnaC.GLN1.1.a*	BolGLN1.1_C1	*BolC.GLN1.1.a*		
*AtGLN1.2* (At1g66200)	BnaGLN1.2_C1; X76736 (BnGSR1.1)	*BnaA.GLN1.2.a*			BraGLN1.2_C1; EU499383; AY773089	BraA.GLN1.2.a (Bra039756; A02)
	BnaGLN1.2_C2; Y12459 (BnGSR1.2)	*BnaC.GLN1.2.a*	BolGLN1.2_C1; EU822334; EU822335	*BolC.GLN1.2.a*		
*AtGLN1.3* (At3g17820)	BnaGLN1.3_C2; JX306693	*BnaA.GLN1.3.a*			BraGLN1.3_C1	BraA.GLN1.3.a (Bra022247; A05)
	BnaGLN1.3_C1; JX306690	*BnaC.GLN1.3.a*	BolGLN1.3_C1	*BolC.GLN1.3.a*		
	BnaGLN1.3_C4	*BnaA.GLN1.3.b*			BraGLN1.3_C3	BraA.GLN1.3.b (Bra021276; A01)
	BnaGLN1.3_C3	*BnaC.GLN1.3.b*				
	BnaGLN1.3_C5; JX306694	*BnaA.GLN1.3.c*			BraGLN1.3_C2^*b*^	BraA.GLN1.3.c; (Bra001686; A03)^*c*^
	BnaGLN1.3_C6	*BnaC.GLN1.3.c*	BolGLN1.3_C2^*b*^	*BolC.GLN1.3.c*		
*AtGLN1.4* (At5g16570)	BnaGLN1.4_C1; JX306697; JX306692^*c*^	*BnaA.GLN1.4.a*			BraGLN1.4_C1	BraA.GLN1.4.a (Bra023573; A02)
	BnaGLN1.4_C2; JX306695	*BnaC.GLN1.4.a*	BolGLN1.4_C1	*BolC.GLN1.4.a*		
	BnaGLN1.4_C4; JX306700^*c*^	*BnaA.GLN1.4.b*			BraGLN1.4_C2	BraA.GLN1.4.b (Bra008612; A10)
	BnaGLN1.4_C3; JX306698^*c*^	*BnaC.GLN1.4.b*	BolGLN1.4_C2	*BolC.GLN1.4.b*		
*AtGLN1.5* (*At1g48470*)	BnaGLN1.5_C2	*BnaA.GLN1.5.a*			BraGLN1.5_C1^*b*^	BraA.GLN1.5.a (Bra018729; A06)
	BnaGLN1.5_C1; JX306691^*b*^	*BnaC.GLN1.5.a*	*BolGLN1.5_C1* ^*b*^	*BolC.GLN1.5.a*		

^*a*^ Annotation and localization on the linkage group from BRAD, the *Brassica rapa* genome sequencing project consortium (Wang *et al.*, 2011).

^*b*^ Incomplete CDS sequence when compared with the *A. thaliana* CDS.

^*c*^ SNP insertion disrupting the ORF when compared with the *A. thaliana* CDS reference sequence and the BnaGLN1 contig.

Most of the EST assemblies have been confirmed through sequencing partial or full-length cDNA clones when available (Supplementary Table S1 at *JXB* online). For the few EST assemblies that cannot be confirmed in this way, the cloning of missing coding sequences (CDS) was performed by designing primers from the *B. rapa* and *B. oleracea* homologous contig sequences (see the Materials and methods). This allowed the completion of the BnaGLN1.4_C3 and BnaGLN1.4_C4 sequences.

Except for the four BnaGLN1.1_C1, BnaGLN1.1_C2, BnaGLN1.2_C1, and BnaGLN1.2_C2 contigs, no other *BnaGLN1* sequences have ever been described previously in the literature or reported in databases as glutamine synthetase gene products. While the BnaGLN1.1_C1, BnaGLN1.1_C2, BnaGLN1.2_C1, and BnaGLN1.2_C2, sequences have been identified as BnGSR2-1, BnGSR2-2, BnGSR1-1, and BnGSR1-2 mRNA, respectively (Table 2; Ochs *et al.*, 1999), contig analysis allowed the completion of the 5′ end (untranslated region and coding sequence) of the BnGSR2-2 sequence that was previously missing (Supplementary Data File S3 at *JXB* online).

### Genetic localization of the *BnaGLN1* loci on the A or C *Brassica* genome using PCR and gene name annotation

Phylogenetic analyses showed a strong relationship between each *BnaGLN1* gene and a gene from one or other of the progenitors *B. rapa* and *B. oleracea*, suggesting a common ancestral origin on the A or C *Brassica* genome, respectively. The phylogenetic tree also shows that each *B. napus* sequence, related to one sequence from either progenitor, is also related to another *B. napus* sequence, itself related to the other progenitor. The two *B. napus* homeologous genes, the *B. rapa* and the *B. oleracea* genes, are thus defining in this way a homeology group (a, b, or c). It was found therefore that each of the *AtGLN1.1*, *AtGLN1.2*, and *AtGLN1.5* genes is related to one homeology group, while the *AtGLN1.3* and *AtGLN1.4* genes are related to three and two groups, respectively. It has to be noted that the b group related to *AtGLN1.3* is incomplete as no *BolGLN1.3* expressed sequence has been identified. Both the homeology groups and the *Brassica* genome were used to ascribe names to the *BnaGLN1* genes; thus, the genes are named *Bna[A or C genome]GLN1.x[a, b, or c homeology group]* according to Ostergaard and King (2008). A similar notation was used for the *BraGLN1* and *BolGLN1* genes (Table 2).

In order to identify the A or C genome origin, the potential localization of the *BnaGLN1* genes on linkage groups and/or chromosomes known to arise from the A or C *Brassica* genomes was then investigated. Specific primer pairs were designed to localize each *BnaGLN1* gene (Supplementary Table S1 at *JXB* online). Five genes were mapped in this way on at least one of the three mapping populations available (Stellar×Drakar, Darmor×Samouraï, and Darmor-*bzh*×Yudal), recording the presence/absence of polymorphism. In good agreement with the phylogenetic tree analysis, the three genes *BnaA.GLN1.3.c*, *BnaA.GLN1.3.a*, and *BnaA.GLN1.5.a* were localized on linkage groups associated with the A genome on chromosomes A03, A05, and A06, respectively. The two genes *BnaC.GLN1.2.a* and *BnaC.GLN1.3.c* were localized on linkage groups associated with the C genome on chromosomes C02 and C03 (Table 3; Supplementary Fig. S1 at *JXB* online).

**Table 3.** *Genetic mapping of* BnaGLN1 *genes*Mapping was performed using specific primers for each contig and different mapping populations or genotypes for *B. napus*, *B. oleracea*, and *B. rapa*. Linkage groups used to assign each gene to A or C genomes are presented.

BnaGLN1		Mapping population	Linkage group (previous name)	LOD	Upstream marker		Downstream marker	
Gene name	Contig name				Name	Distance (cM)	Name	Distance (cM)
*BnaA.GLN1.1.a*	BnaGLN1.1_C1	Panel^*a*^	A					
*BnaC.GLN1.1.a*	BnaGLN1.1_C2	Add^*b*^	C06					
*BnaA.GLN1.2.a*	BnaGLN1.2_C1	Panel^*a*^	A					
*BnaC.GLN1.2.a*	BnaGLN1.2_C2	SD^*c*^	C02 (SD02)	13.5	PFM504	9.1	J15.1200	9.1
*BnaA.GLN1.3.a*	BnaGLN1.3_C2	DS^*d*^	A05 (DS19)	17	E1M4.21	2.7	BN614	13.5
*BnaC.GLN1.3.a*	BnaGLN1.3_C1	Add^*b*^	C05					
*BnaA.GLN1.3.b*	BnaGLN1.3_C4	Panel^*a*^	A					
*BnaC.GLN1.3.b*	BnaGLN1.3_C3	Add^*b*^	C01					
*BnaA.GLN1.3.c*	BnaGLN1.3_C5	DS^*d*^	A03 (DS04)	16.1	PFM 193	3.8	BN466	7.9
*BnaC.GLN1.3.c*	BnaGLN1.3_C6	SD^*c*^, add^*b*^	C03 (SD717)	25.9	BN04C	1.3	IGF0193c	2.2
*BnaA.GLN1.4.a*	BnaGLN1.4_C1	Panel^*a*^	A					
*BnaC.GLN1.4.a*	BnaGLN1.4_C2	Panel^*a*^	C					
*BnaA.GLN1.4.b*	BnaGLN1.4_C4	Panel^*a*^	A					
*BnaC.GLN1.4.b*	BnaGLN1.4_C3							
*BnaA.GLN1.5.a*	BnaGLN1.5_C2	DY^*e*^	A06 (DY06)	8.9	nr	nr	BN57463	21.8
*BnaC.GLN1.5.a*	BnaGLN1.5_C1	Panel^*a*^	C					

^a^ Panel of *B. napus*, *B. oleracea*, and *B. rapa* genotypes.

^*b*^ Monosomic and polysomic addition lines obtained from the Darmor-*bzh*×C1.3 cross.

^*c*^ Stellar×Drakar mapping population.

^*d*^ Darmor×Smouraï mapping population.

^*e*^ Darmor-*bzh*×Yudal mapping population.

For the other members of the *BnaGLN1* gene family, an attempt to assign the *BnaGLN1* genes to the A or C genomes using mapping populations was unsuccessful. Therefore, a panel of various Brassicaceae genotypes (Supplementary Fig. S2 at *JXB* online) was used in order to detect the *BolGLN1* and *BraGLN1* orthologous genes, using specific *BnaGLN1* primers (Supplementary Table S1). The number of genotypes used for each *Brassica* species was adjusted in order to take into account the possible allelic variations and to detect the presence/absence of polymorphism. Furthermore, additional lines carrying the full A genome and one or several *B. napus* C chromosomes were used in order to determine preciselg the localization of the *BnaC.GLN1* genes (Auger *et al.*, 2009) (Supplementary Fig. S3).

The results are summarized in Table 3 and detailed in Supplementary Figs S1, S2, and S3 at *JXB* online. In brief, with the exception of *BnaC.GLN1.4.b*, all the identified *BnaGLN1* genes were assigned to the A or C *Brassica* genome, confirming the two by two relationship of homology between them, which allowed them to be named according to their ancestral genome origin as recommended by Ostergaard and King (2008).

### Identification of *GLN1* genes of *Brassica rapa* and *Brassica napus genomes*

The recently sequenced and annotated *B. rapa* genome (BRAD; Cheng *et al.*, 2011) was used to perform BLAST searches and sequence alignments using the *BraGLN1* contig sequences identified here. Analysis revealed eight annotated *BraGLN1* genes (Table 2). Alignments between *BraGLN1* gene sequences and contigs revealed potential splicing variants. Indeed, the CDS deduced from the *BraGLN1.3_C2* contig appeared incomplete at the 5′ end. The most highly similar Bra001686 annotated gene on the A03 chromosome also appeared incomplete when compared with the *AtGLN1.3* CDS, as it is missing the first exon. The BLAST search on A03 chromosome v1.1 revealed the presence of a sequence highly similar to the *AtGLN1.3* first exon, 4kb upstream of the identified Bra001686 sequence (bp 17 854 801 to 17 854 849). According to the BRAD annotation, this inserted region has been described as an long terminal repeat (LTR) transposon of 3746bp on the minus strand. The identified *B. rapa* EST (EX089134) that allowed identification of the 5′ region of the BraGLN1.3_C2 contig starts in the transposon region and continues into the first identified exon of Bra001686 which corresponds to the second *AtGLN1.3* exon.

**Fig. 2.**
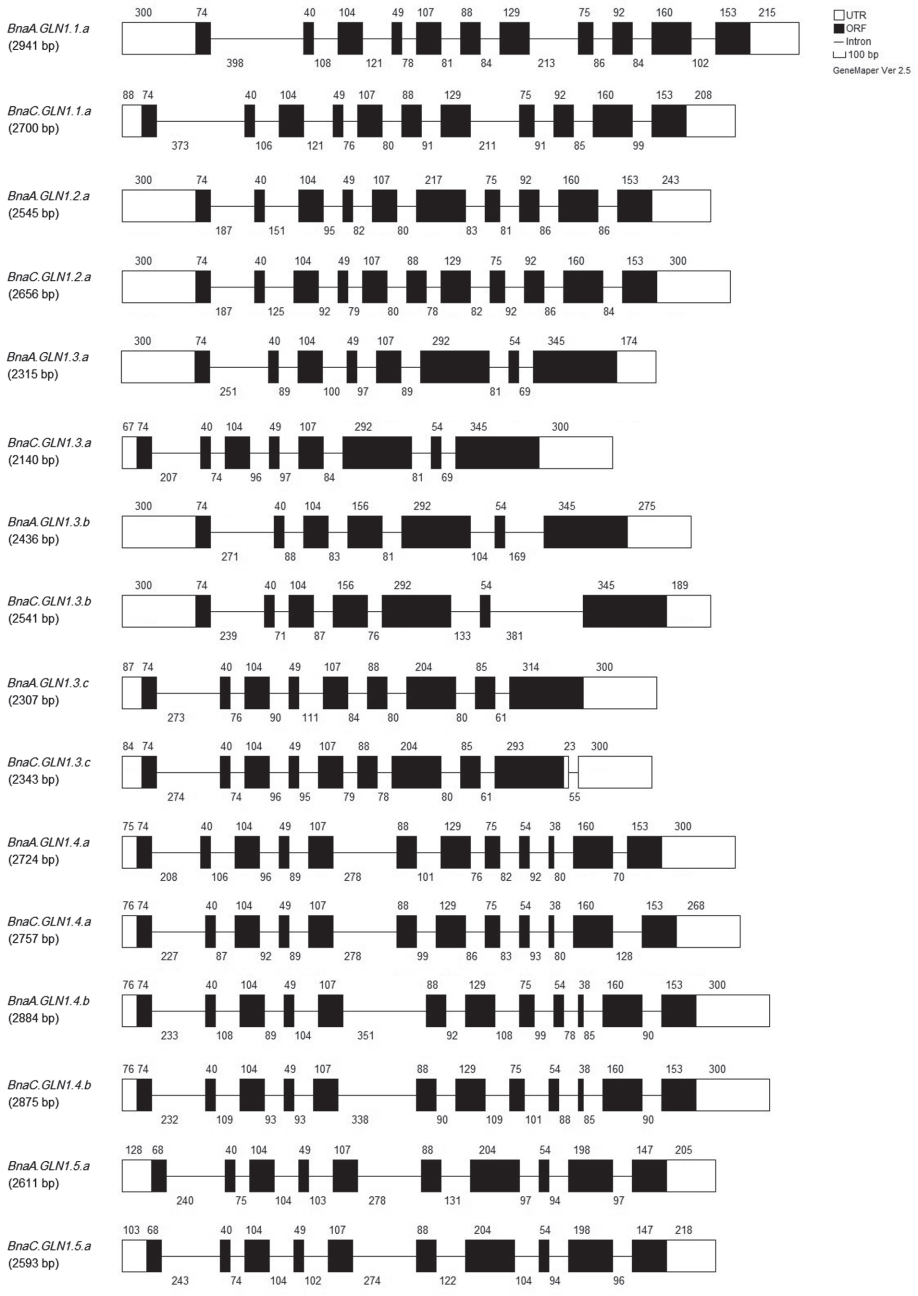
Structure of *BnaGLN1* genes. For each *BnaGLN1* gene, the length of the 5′ and 3′ untranslated regions (UTRs) (white boxes), exons (black boxes), and introns (black lines) is represented by a number corresponding to base pairs.

The sequencing programme performed at URGV allowed identification of *BnaGLN1* sequences in the *B. napus* Darmor-*bzh* genome (SEQ-POLYNAP, ANR-09-GENM-021). The BraGLN1 protein sequences were deduced from the identified *BraGLN1* genes using the BRAD tool, and used to search the database of protein sequences built from *B. napus* genomic sequence analysis (unpublished data). From the BnaGLN1 protein sequences identified, genomic sequences were recovered (Supplementary Data File S4 at *JXB* online). Interestingly, 16 putative *BnaGLN1* genes and two putative *BnaGSL* genes (coding for the GS2 isoform) were found. The 16 *BnaGLN1* genomic sequences (Supplementary Data File S4) were used to analyse similarities with the *B. napus* contigs and to create a phylogenetic tree (Supplementary Fig. S4). Interestingly, each genomic sequence was closely associated with one contig sequence, suggesting that all the genes with the 16 *BnaGLN1* contigs had been found. The deduced mRNA sequences (Supplementary Data File S5), obtained using FGENESH software available on the SoftBerry website (http://linux1.softberry.com/berry.phtml?topic=fgenesh&group =programs&subgroup=gfind), showed very a high similarity with the contig sequences (Table 4) and allowed the gene structures to be deduced (Fig. 2). Except for BnaGLN1.3_C5 and BnaGLN1.3_C6, similarities between mRNA and associated contigs were near 100% (Table 4). Knowing that contig sequences and mRNA sequences are obtained from different *B. napus* genotypes (Supplementary Data File S1), this indicates that there is almost no polymorphism between the different *BnaGLN1* coding sequences regarding the various genotypes of *B. napus* used for genome and EST sequencing. The *BnaGLN1* genes contained between seven and 12 exons. *GLN1.4* and *GLN1.5* genes have the same number of exons in both *B. napus* and *Arabidopsis* (Table 4). For the other *GLN1* genes, exon numbers are different between *Arabidopsis* and *B. napus*, but quite close; for example, *AtGLN1.3* and *BnaGLN1.3* contain fewer exons than other *AtGLN1* and *BnaGLN1* genes.

**Table 4.** *Comparison between contigs of ESTs and the mRNA sequences deduced from the* BnaGLN*1 genomic sequences*Comparison of nucleotide length (bp), % similarities, and exon numbers between the *B. napus* contigs and the mRNA sequences (Supplementary Data File S5 at *JXB* online) deduced from the *BnaGLN1* genomic sequences (Supplementary Data File S4). Similarities were estimated using BLAST (NCBI) and exons using FGENESH software at the SoftBerry website.

Gene name	Contig name	Contig length (bp)	Deduced mRNA name	Putative mRNA length (bp)	% similarity between contig and mRNA	No.of exons
*AtGLN1.1*				1494		9
*BnaA.GLN1.1.a*	BnaGLN1.1_C1	1374	mRNA.BnaA.GLN1.1.a	1746	100	11
*BnaC.GLN1.1.a*	BnaGLN1.1_C2	1367	mRNA.BnaC.GLN1.1.a	1474	100	11
*AtGLN1.2*				1499		10
*BnaA.GLN1.2.a*	BnaGLN1.2_C1	1430	mRNA.BnaA.GLN1.2.a	2587	100	9
*BnaC.GLN1.2.a*	BnaGLN1.2_C2	1431	mRNA.BnaC.GLN1.2.a	2123	99	11
*AtGLN1.3*				1341		9
*BnaA.GLN1.3.a*	BnaGLN1.3_C2	1336	mRNA.BnaA.GLN1.3.a	2012	100	8
*BnaC.GLN1.3.a*	BnaGLN1.3_C1	1487	mRNA.BnaC.GLN1.3.a	1555	99	8
*BnaA.GLN1.3.b*	BnaGLN1.3_C4	1273	mRNA.BnaA.GLN1.3.b	2051	99	7
*BnaC.GLN1.3.b*	BnaGLN1.3_C3	1275	mRNA.BnaC.GLN1.3.b	1803	100	7
*BnaA.GLN1.3.c*	BnaGLN1.3_C5	1253	mRNA.BnaA.GLN1.3.c	1840	96	9
*BnaC.GLN1.3.c*	BnaGLN1.3_C6	1245	mRNA.BnaC.GLN1.3.c	1761	96	9
*AtGLN1.4*				1269		12
*BnaA.GLN1.4.a*	BnaGLN1.4_C1	1273	mRNA.BnaA.GLN1.4.a	1461	100	12
*BnaC.GLN1.4.a*	BnaGLN1.4_C2	1259	mRNA.BnaC.GLN1.4.a	1415	99	12
*BnaA.GLN1.4.b*	BnaGLN1.4_C4	1102	mRNA.BnaA.GLN1.4.b	2582	99	12
*BnaC.GLN1.4.b*	BnaGLN1.4_C3	1123	mRNA.BnaC.GLN1.4.b	1494	100	12
*AtGLN1.5*				1307		10
*BnaA.GLN1.5.a*	BnaGLN1.5_C2	1392	mRNA.BnaA.GLN1.5.a	1329	99	10
*BnaC.GLN1.5.a*	BnaGLN1.5_C1	1380	mRNA.BnaC.GLN1.5.a	1349	100	10

### BnaGLN1 protein sequence conservation

Protein sequences of the BnaGLN1 family deduced from the coding sequences of contigs or from the deduced mRNA are similar. The BnaGLN1 proteins share between 93% and 96.6% identity with the AtGLN1 proteins encoded by their respective orthologous genes (Table 1). Within each homeology group, the A and C BnaGLN1 proteins share 98.3–100% identity.

**Fig. 3.**
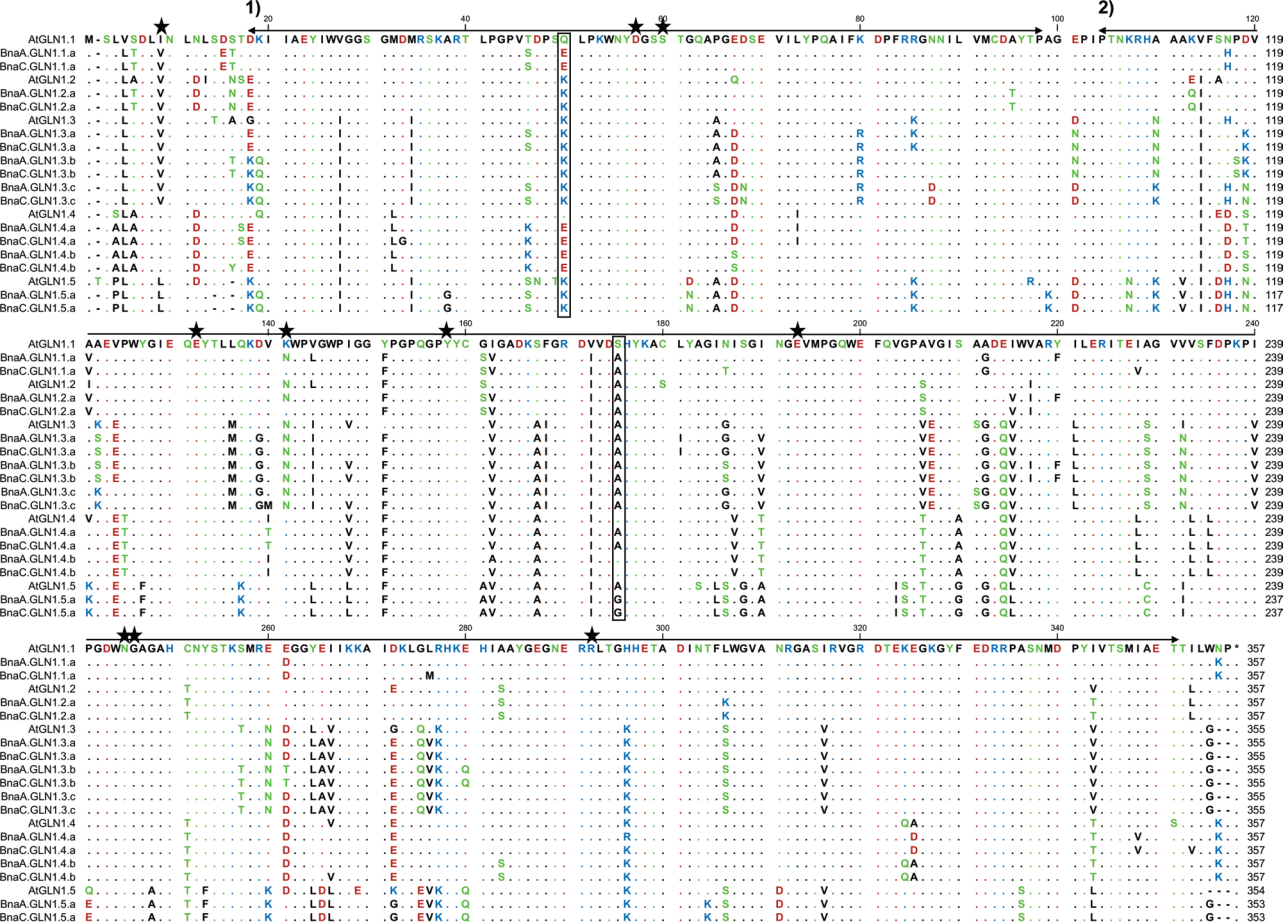
Alignment of *Brassica napus* and *Arabidopsis thaliana* GS1 proteins. Protein sequences were deduced from DNA coding sequences and aligned using Clustal. Stars indicate residues involved in the ammonium/glutamate-binding pocket (Eisenberg *et al.*, 2000). Boxes indicate residues involved in ammonium affinity properties (Ishiyama *et al.*, 2006). Arrows indicate conserved domains (1) pfam 03951 Gln-synt_N glutamine synthetase bet-Gasp domain; and (2) pfam 00120 gln-synt_C catalytic domain. Residues are coloured according to their polarity properties (neutral non-polar as black, neutral polar as green, acidic as red, and basic as blue).

In all BnaGLN1 protein sequences, two conserved pfam domains specific to glutamine synthetase enzymes (pfam 03951 and pfam 00120) were identified (Fig. 3). The residues involved in the ammonium/glutamate-binding pocket (Eisenberg *et al.*, 2000) are also strictly conserved. In contrast, the polar amino acids Q49 and S174, shown to be involved in the ammonium high affinity properties of AtGLN1.1 and AtGLN1.4 (Ishiyama *et al.*, 2006), are not strictly conserved in all the BnaGLN1.1 and BnaGLN1.4 proteins. The polar Q49 was converted into an acidic glutamate E49 in all the BnaGLN1.1 and BnaGLN1.4 sequences, and the S174 is conserved only in the two BnaGLN1.4.b sequences but was converted into an A174 in the BnaGLN1.4.a and BnaGLN1.1.a sequences. Depending on the effect of such amino acid modifications, it might be possible that ammonium affinity properties have not been conserved within the BnaGLN1.1 and BnaGLN1.4 protein families. In contrast, the residues K49 and A174 present in the low affinity enzymes AtGLN1.2 and AtGLN1.3 are conserved in all the BnaGLN1.2 and BnaGLN1.3 protein sequences, suggesting the conservation of the low ammonium affinity properties in those two protein families (Fig. 3).

### Expression of *BnaGLN1* genes is modified depending on the nitrogen regime and leaf senescence

A first analysis of EST distribution between libraries and BnaGLN1 contigs led to the conclusion that *BnaGLN1* genes are probably differentially expressed according to tissue and developmental stage (Supplementary Data File S1 at *JXB* online).

**Fig. 4.**
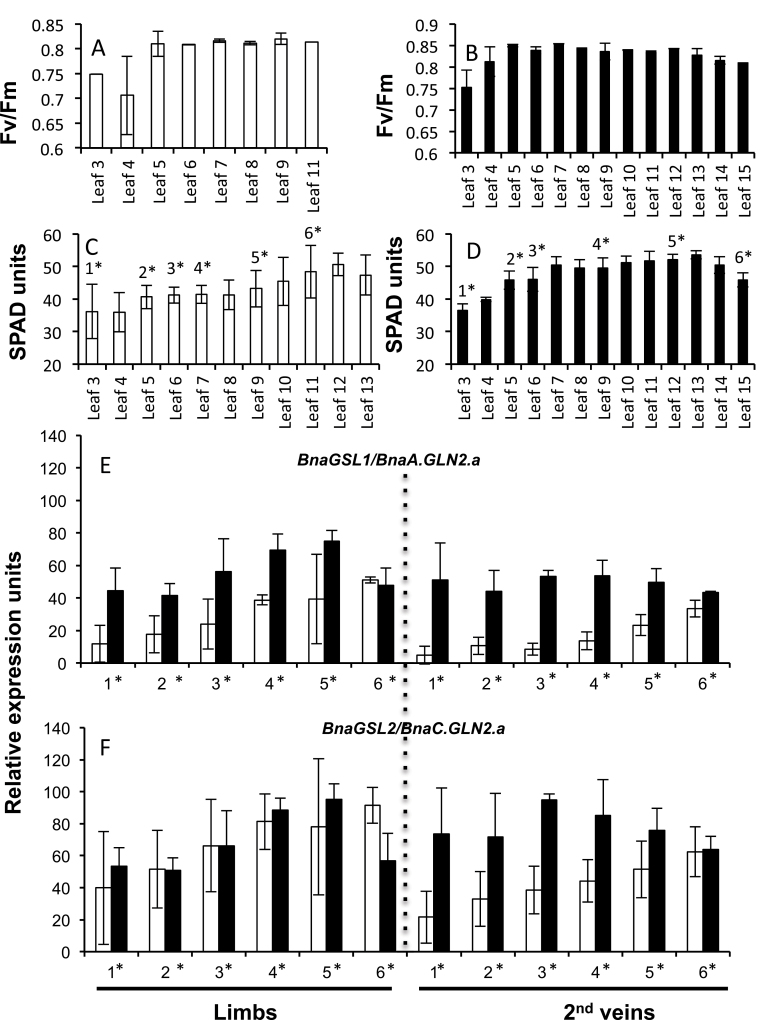
Leaf senescence markers on vegetative *B. napus* plants. *F*_v_/*F*_m_ photosystem II capacity (A, B) and chlorophyll relative content (C, D) were monitored at the vegetative stage in all the leaf ranks of four *B. napus* plants grown under low (white bars) or high (black bars) nitrate conditions. The expression of the *BnaA.GLN2.a* and *BnaC.GLN2.a* marker genes was quantified (E, F) on selected leaf ranks (*) and confirmed differential senescence symptoms. Mean and standard deviation of four plant repeats are shown.

The *BnaGLN1* gene expression was monitored at the vegetative stage measuring transcript levels by quantitative real-time RT–PCR in samples of taproot, crown, limbs, and veins of plants grown under low or high nitrate conditions. Plants grown under low or high nitrate conditions grew 13 and 17 leaves, respectively. *F*_v_/*F*_m_ and SPAD measurements on all the leaf ranks (numbered from the bottom leaf to the top leaf) were done to estimate the relative leaf senescence status of each leaf. From both SPAD and *F*_v_/*F*_m_ as senescence markers, six leaves were selected from each nitrate condition presenting differential senescence levels to perform further experiments (Fig. 4). Leaves of rank 3, 5, 6, 7, 9, and 11 were harvested on plants grown under low nitrate conditions. Leaves 3, 5, 6, 9, 12, and 15 were harvested on plants grown under high nitrate conditions. To simplify the presentation of further results, the collected leaf ranks were renamed 1, 2, 3, 4, 5, and 6, respectively, with 1 designating the bottom-most and oldest collected leaf and 6 the youngest collected leaf. Leaves dissected as limbs, and primary and secondary veins were used to measure *BnaGLN1.1* gene expression levels in the different tissues. In addition to *BnaGLN1.1* expression, the expression of *BnaGSL1* and *BnaGSL2* encoding the chloroplastic GS2 isoenzymes was also monitored and used as a control for leaf senescence as it is known that genes encoding GS2 izoenzymes are down-regulated with leaf ageing in all the plant species studied so far (Masclaux-Daubresse *et al.*, 2008). *BnaGSL1* and *BnaGSL2* expression levels confirmed the differential senescence phenotype of the chosen leaf ranks. Leaves 1, 2, and 3 can be considered as senescing leaves, 4 and 5 as mature leaves, and 6 as a young leaf according to Masclaux *et al.* (2000) (Fig. 4E, F).

**Fig. 5.**
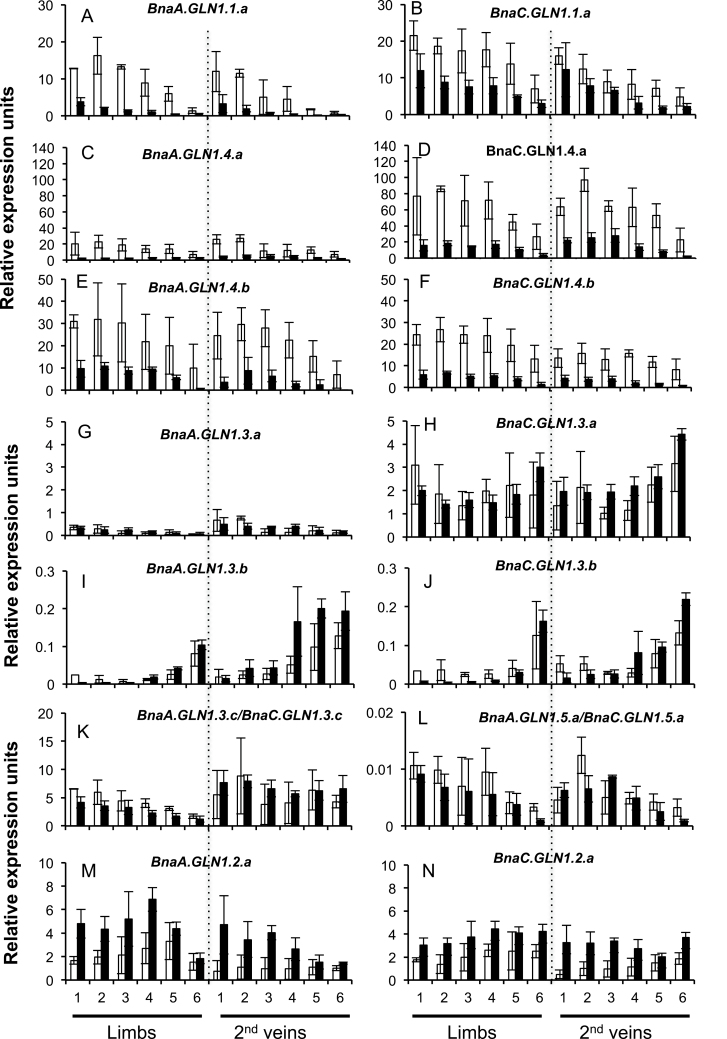
Expression of *BnaGLN1* genes is modified depending on nitrate availability and leaf ageing. The relative expression level of *BnaGLN1* genes was monitored in limbs and secondary veins of six leaf ranks harvested on vegetative plants grown under low (white bars) or high (black bars) nitrate conditions. Leaf ranks represented as number 1 (bottom and older leaf) to 6 (top and younger leaf) showed differential senescence symptoms. Mean and standard deviation of four plant repeats are shown.

**Fig. 6.**
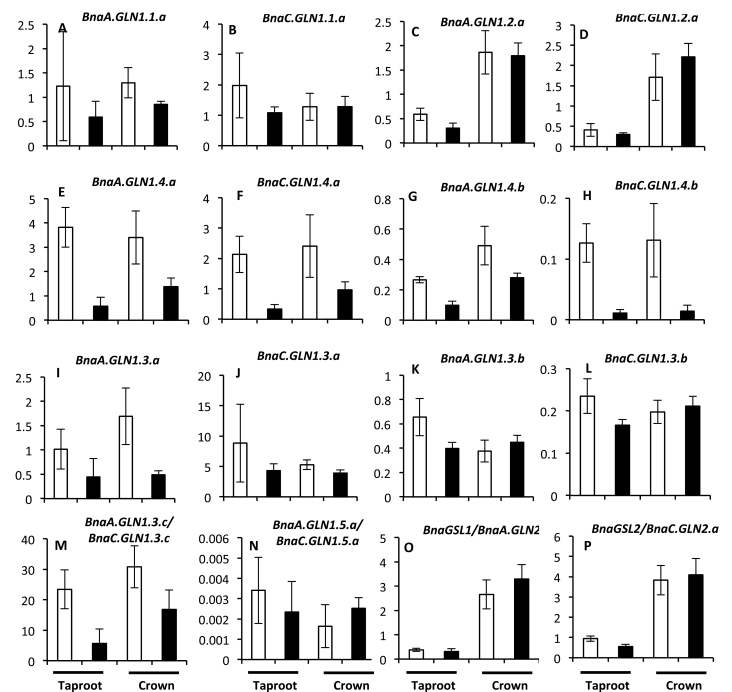
Expression of *BnaGLN1* genes is modified depending on nitrate availability in the taproot and crown of vegetative *B. napus* plants. The relative expression level of *BnaGLN1* genes was monitored at the vegetative stage in the taproot and crown of four plants grown under low (white bars) or high (black bars) nitrate conditions. Mean and standard deviation of four plant repeats are shown.

Genes that are preferentially expressed under high or low nitrate conditions were identified. The results showed that regarding the N regime, all the members of the same gene family respond similarly, with a few exceptions from the *BnaGLN1.3* family. It was observed that all the members of the *BnaGLN1.1* and *BnaGLN1.4* gene families were significantly induced under low nitrate conditions compared with high nitrate. This was observed in limbs, secondary veins (Fig. 5A–F), and also in primary veins for some *BnaGLN1.4* genes (Supplementary Fig. S5E–H at *JXB* online). Induction under low nitrate conditions was also clearly observed in the taproot and crown (Fig. 6A, B; E–H). In contrast, the two *BnaGLN1.2* genes were significantly more expressed under high nitrate conditions in leaf limbs and veins but not in the taproot and crown (Fig. 5M, N; C, D). Finally no difference was observed in the expression of the *BnaGLN1.3* and *BnaGLN1.5* families in leaf limbs or veins between the high nitrate and low nitrate conditions (Fig. 5G–L). Surprisingly, all the *BnaGLN1.3* genes are significantly induced under low nitrate conditions in the taproot but not in crown tissue (Fig. 6I–M). Therefore, N-dependent regulation might be different in the root and shoot.

*BnaGLN1* genes also appeared to be differentially expressed depending on leaf ageing and senescence. The two *BnaGLN1.1* genes and the four *BnaGLN1.4* genes were significantly induced in leaf limbs and veins with ageing and during senescence independently of nitrate conditions (Fig. 5A–F; Supplementary Fig, S5B, E–H at *JXB* online). Cumulated expression of the *BnaA.GLN1.5.a/BnaC.GLN1.5.a* genes (Fig. 5L) also increased with leaf ageing and senescence in limbs and veins of plants grown under low and high nitrate conditions. In contrast, senescence triggers an opposite effect on the mRNA level of the two *BnaGLN1.2* genes, especially under low nitrate nutrition (Fig. 5M, N). The effect of senescence was less evident under high nitrate conditions in limbs and veins due to the already high *BnaGNL1.2* expression in mature leaves. In these leaves, expression profiles are biphasic, increasing from young to mature leaves then decreasing in senescing leaves. Profiles are more complex in the *BnaGLN1.3* family since the expression of *BnaC.GLN1.3.a*, *BnaA.GLN1.3.b*, and *BnaC.GLN1.3.b* (Fig. 5H–J) is repressed with leaf ageing in limbs and veins, while the expression of *BnaA.GLN1.3.a* (Fig. 5G) and cumulated *BnaA.GLN1.3.c/BnaC.GLN1.3.c* (Fig. 5K) is increased with ageing in limbs.

These results show that within all the *BnaGLN1* families except *BnaGLN1.3*, all members show similar expression levels. The four *BnaGLN1.4* genes are the most highly expressed in all the tissues studied. *BnaGLN1.4* gene expression is four times higher than that of the *BnaGLN1.1* genes and 20 times higher than that of *BnaGLN1.2*. The expression level of all the *BnaGLN1.3* and *BnaGLN1.5* genes is much lower, except that of the cumulated *BnaGLN1.3c* genes that reach a similar level to *BnaGLN1.2*. Table 5 summarizes the N and senescence effects observed on the *BnaGLN1* expression levels.

**Table 5.** *Nitrogen and senescence effects observed on the* BnaGLN1 *expression levels at vegetative, flowering and seed filling stages*Significant positive and negative effects of nitrogen limitation (A) and ageing (B) on the expression of the *BnaGLN1* genes recorded at vegetative, flowering, and seed filling stages are reported. The average level of expression of each *BnaGLN1* gene as the mean of the data recorded in all the samples analysed is reported in column 2 (relative to the *BnaX.SAND* gene).

Gene(s) name	Mean relative expression level	(A) Nitrogen limitation effect									(B) Age effect						
		Vegetative stage					Flowering		Seed filling		Vegetative stage			Flowering		Seed filling	
		Limb	Primary vein	Secondary vein	Tap root	Crown	Limb	Stem	Limb	Stem	Limb	Primary vein	Secondary vein	Limb	Stem	Limb	Stem
*BnaA.GLN1.1.a*	4.59	+		+	+	+	+		+	+	+		+		+		+
*BnaC.GLN1.1.a*	9.64	+	+	+	+		+	+	+	+	+	+	+	+	+	+	+
*BnaA.GLN1.2.a*	2.69	–	–	–	+		–	–	–	+	+	+	+	+	+	+	+
*BnaC.GLN1.2.a*	2.59	–	–	–		–	–	–	–			+		+		+	
*BnaA.GLN1.3.a*	0.26				+	+				+	+		+		+		+
*BnaC.GLN1.3.a*	2.07				+	+				+							
*BnaA.GLN1.3.b*	0.06		–	–	+			–	–	+	–		–		–		–
*BnaC.GLN1.3.b*	0.06		–	–	+			–	–	+	–		–		–		–
*Bna.GLN1.3.ca*	4.82				+	+				+	+						
*BnaA.GLN1.4.a*	9.54	+		+	+	+	+		+	+	+	+	+	+	+	+	+
*BnaC.GLN1.4.a*	38.29	+		+	+	+	+		+	+	+	+	+	+	+	+	+
*BnaA.GLN1.4.b*	14.18	+		+	+	+	+		+	+	+	+	+	+	+	+	+
*BnaC.GLN1.4.b*	10.65	+	+	+	+	+	+	+	+	+	+	+		+		+	
*Bna.GLN1.5.aa*	<0.01										+		+				

^a^Relative expression level associated to both A and C genes.

### *BnaGLN1* genes are differentially expressed at the reproductive stage depending on plant organs or leaf ageing

In order to monitor *BnaGLN1* gene expression at the reproductive stage, plants were grown in field conditions under low or high N regimes. Two leaf ranks (young top leaf and old bottom leaf) and the two corresponding stem sections (also referred to hereafter as young and old) were collected at flowering and during grain filling.

Globally, effects of N limitation on *BnaGLN1* expression were similar to those found at the vegetative stage, except that the magnitude of gene repression or induction was lower than that observed at the vegetative stage (Supplementary Table S5 at *JXB* online).

**Fig. 7.**
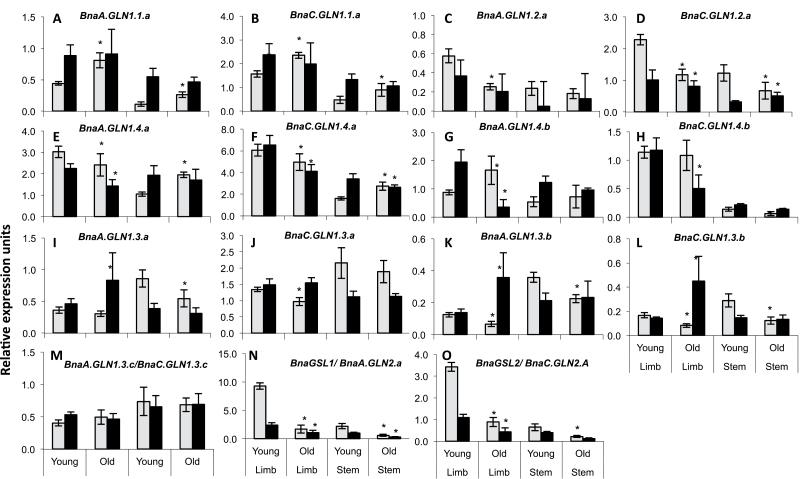
*BnaGLN1* genes are differentially expressed depending on flowering or seed filling stages and leaf age. The relative expression level of *BnaGLN1* genes was monitored in young and old leaves of plants grown in the field and supplemented with nitrogen. Leaf limbs and stems were collected at flowering (white bars) and seed filling (black bars) stages. Mean and standard deviation of four plant repeats are shown. * indicates significant difference (Student’s *t*-test, *P*<0.05) between flowering and seed filling stages.

Figure 7 reports the effect of senescence on the expression of the *BnaGLN1* genes in leaves and stems of plants grown under a sufficient N regime. As a control of leaf senescence stages, the *BnaGSL1* and *BnaGSL2* genes are significantly more highly expressed in the young tissues than in older tissues (Fig. 7N, O). There was a sharp decrease in *BnaGSL* gene expression at the flowering stage, while at the seed filling stage the magnitude of *BnaGSL* repression was much lower but still significant.

As observed at the vegetative stage, the *BnaGLN1.1* genes were up regulated with leaf and stem senescence, but this was only observed at the flowering stage (Fig. 7A, B). During seed filling, expression in leaves and stems was higher than during flowering, showing an effect of plant ageing. However, no difference was observed between the young and old leaves, suggesting that both types of leaves had become senescent between flowering and seed filling. The two *BnaGLN1.1* genes were expressed more highly in leaf blades than in stems at both the flowering and seed filling stages. Similarly the *BnaGLN1.2* genes were more expressed in leaf blades than in stems (Fig. 7C, D). The effect of senescence on *BnaGLN1.2* genes was opposite to the effect observed on *BnaGLN1.1* genes. *BnaGLN1.2* expression decreased 2- to 3-fold in old leaf blades and old stems compared with young leaf blades and young stems, respectively. The biphasic profile obtained for *BnaA.GLN1.2* at the vegetative stage was also observed at the flowering stage (data not shown). As observed with the *BnaGLN1.1* genes, the effect of senescence was no more significant at seed filling.

Among the four *BnaGLN1.4* genes, only *BnaA.GLN1.4.a* and *BnaC.GLN1.4.a* shared similar expression profiles (Fig. 7E–H). They are preferentially expressed in leaf blades rather than in stems. In contrast to the vegetative stage, *BnaA.GLN1.4.a* and *BnaC.GLN1.4.a* tend to be repressed by senescence in leaf blades but induced by senescence in stems. This trend was especially significant at the flowering stage. In contrast, *BnaA.GLN1.4.b* was induced by senescence in leaf blades and stems at the flowering stage but repressed during seed filling (Fig. 7G). Finally, *BnaC.GLN1.4.b* expression was higher in leaf blades than in stems and was repressed by senescence at the flowering stage, similarly to the two *BnaA.GLN1.4.a* and *BnaC.GLN1.4.a* genes (Fig. 7H). In contrast to the vegetative stage, the members of the *BnaGLN1.4* family have developed specificities and are differentially expressed at the flowering and seed filling stages. It is likely that they have different roles and influences on N metabolism after flowering.

Among the *BnaGLN1.3* members, similar profiles were observed for *BnaA.GLN1.3.a*, *BnaA.GLN1.3.b*, and *BnaC.GLN1.3.b* (Fig. 7I, K, L). These three genes are down-regulated in old leaves and stems compared with young leaves at the flowering stage. However, their expression increased sharply in old limbs at the seed filling stage. The other *BnaC.GLN1.3.a* and *BnaA.GLN1.3.c/ BnaC.GLN1.3.c* expression profiles did not show any modification associated with leaf or stem senescence (Fig. 7J, M). All the *BnaGLN1.3* genes appeared to be more highly expressed in stems than in leaves, especially at the flowering stage.

It was not possible to measure *BnaA.GLN1.5.c/BnaC.GLN1.5.c* gene expression, possibly due to the very low expression level in vegetative tissues that cannot be accurately measured in field-grown plants.

Results obtained at the flowering and seed filling stages confirm results from the vegetative stage. *BnaGLN1* genes are generally similarly regulated according to their orthology group, although exceptions were observed particularly at the seed filling stage, such as with *BnaA.GLN1.4.b* and *BnaC.GLN1.3.a* (Fig. 7G, J).

## Discussion

Glutamine synthetase is a key enzyme of N metabolism involved in ammonium assimilation and remobilization. Recent studies highlight the important role of GS1 cytosolic isoenzymes for N management linked to yield establishment and seed filling in monocotyledonous crops (Tabuchi *et al.*, 2005; Martin *et al.*, 2006; Bernard and Habash, 2009; Swarbreck *et al.*, 2011). The GS1-coding genes are therefore good candidates for improving yield traits and grain quality (Masclaux-Daubresse *et al.*, 2008). The complexity of studying glutamine synthetases arises from the fact that two isoenzymes exist, one in the chloroplast and the other in the cytosol, and that several isoforms exist for the cytosolic enzyme. The numerous isoforms are encoded by a multigenic family, and the five *GLN1* genes in *A. thaliana* are likely to present different roles depending on plant organs and nitrate availability in the soil (Lothier *et al.*, 2011). Similarly the five maize *GLN1* genes do not participate equally in N management at the whole-plant level (Martin *et al.*, 2006; Hirel *et al.*, 2007). The aim of this study was to identify the whole *BnaGLN1* gene family and to characterize the expression of the different genes depending on nitrate availability as well as depending on ageing and leaf and stem tissue senescence.

Using the sequences obtained from EST libraries and genome sequencing, a total of 16 genes belonging to the *BnaGLN1* family, eight genes from each of the A and C genomes, were found. In accordance with the history of *B. napus* genome formation (Nagaharu, 1935), it was found that each *BnaGLN1* gene is closely related to a *BraA.GLN1* or *BolC.GLN1* gene depending on its A or C genome location. Therefore, it can be stated with confidence that all the *GLN1.1* genes of *B. napus* have been described in this report. Sequence analyses also showed that *B. napus* coding sequences are highly conserved between the A and C genomes and also between *B. napus* genotypes. The level of sequence divergence observed in the *BnaGLN1* family is between 0.9% and 2.9% SNPs (single nucleotide polymorphisms) in CDS, which is less than the preliminary observation showing sequence divergences of ~3–5% SNPs in CDS from SLR1 (Inaba and Nishio, 2002).

It is well known that *B. napus* shows a high degree of collinearity to its diploid progenitors *B. rapa* and *B. oleracea* (Rana *et al.*, 2004). Many studies have investigated the segmental structure of the *Brassica* genomes and led to the identification and genetic mapping of syntenics blocks between *A. thaliana* and the *Brassica* genomes (Parkin *et al.*, 2005; Schranz *et al.*, 2006; J. Wang *et al.*, 2011). The number of potential *BnaGLN1* genes and their localization on linkage groups can then be predicted depending on the number of times the blocks are replicated and on their localization on each *B. napus* linkage group. According to the whole-genome triplication event that occurred in Brassicaceae genome species after divergence from *Arabidopsis* (Lysak *et al.*, 2005; Parkin *et al.*, 2005), and the recent hybridization between *B. rapa* and *B. oleracea* leading to the appearance of *B. napus*, each *AtGLN1* gene could have been found in triplicate in each A and C genome from *B. napus* to form three pairs of homeologous genes. From EST and genome sequence analyses, it is revealed here that only one homeology group exists for *AtGLN1.1*, *AtGLN1.2*, and *AtGLN1.5* and two groups for *AtGLN1.4*. *AtGLN1.3* is the only *GLN1* gene for which the six *BnaGLN1* orthologous genes were retained in the *B. napus* genome. This illustrates the massive gene loss that occurred in the *Brassica* lineage after the whole-genome triplication event. Indeed, using *A. thaliana* as an outgroup, Town *et al.* (2006) found that 35% of genes inferred to be present when genome triplication occurred in the *Brassica* lineage have been lost in *B. oleracea*. Similarly, whole-genome analysis of *B. rapa* revealed a high rate of gene loss, from 30% to 64% depending on the degree of fractionation of the region considered (X. Wang *et al.*, 2011). *BnaGLN1* families are a good example of this.

With the exception of *BnaGLN1.4.b* genes, the present study points out that pairs of *BnaGLN1* homeologous genes share very similar transcription profiles. Furthermore, within one orthology group, when several groups of homeologues were retained, paralogous genes conserved similar expression profiles (*BnaGLN1.3* and *BnaGLN1.4*). This suggests that coding but also regulatory sequences were essentially conserved after genome merging of *B. napus* progenitors, but also after the whole-genome duplication (WGD) and diploidization events that occurred in the *Brassica* lineage after the divergence from the *Arabidopsis* genus. WGD is generally thought to provide raw material for gene neo- and subfunctionalization, extending resilience to deleterious mutations, increasing the net speciation rate and species richness (Soltis *et al.*, 2009), as well as providing the adaptive advantage for colonizing harsh and unstable environments (Franzke *et al.*, 2011). On the other hand, maintenance of redundancy can confer robustness against mutations (De Smet and Van de Peer, 2012) and/or a selective advantage in increasing the abundance of encoded proteins (Bekaert *et al.*, 2011). As GS1 is an essential enzyme of primary N metabolism, linked to central carbon metabolism via the GS/GOGAT cycle that might also play a role in the adaptation of plant to nutrient deficiency and pathogen attack (Brauc *et al.*, 2011; Seifi *et al.*, 2013), maintenance of multicopy of *GLN1* could confer robustness against mutations. Nevertheless, *GLN1* expression profiles have not been exhaustively investigated and there might be particular environmental or developmental conditions allowing the differentiation of expression profiles between homeologous and/or paralogous genes. Partially overlapping profiles could provide robustness against mutations, but also adaptive advantages for colonizing harsh and unstable environments.

Allopolyploidization involves the merger of two different, and often divergent, genomes whose reconciliation in a common nucleus often leads to myriad changes, including unequal expression of the two merging genomes. Biased expression among homeologues has been found in cotton and wheat (Pumphrey *et al.*, 2009; Rapp *et al.*, 2009). Previous studies have suggested bias toward the *B. rapa* A genome in the transcriptional expression of rRNA genes (Chen and Pikaard, 1997). In the present study it is shown that most of the *BnaGLN1* homeologous pairs display similar expression levels in the various tissues studied. Differences in mRNA contents observed between homeologous pairs of A and C genome origins were generally very small. No systematic bias towards the *B. oleracea* C parental genome or *B. rapa* A genome can be identified in this study. Bias towards the *B. oleracea* C parental genome was identified for *BnaGSL* (at flowering and seed filling stages), *BnaGLN1.3.a* (especially at the vegetative stage), and *BnaGLN1.4.a* homeologues, and in favour of the *B. rapa* parental genome for *BnaGLN1.1.a* and *BnaGLN1.2.a*. These results are in agreement with the recent finding that nearly 7% of the potentially identified homeologous genes expressed in a leaf extract are displaying a differential expression level in favour of the A or C parental genome for 1/3 and 2/3 of the pairs, respectively, and that genes involved in metabolic processees tend to be over-represented (Higgins *et al.*, 2012).

Beside their intrinsic expression levels, it was found that the *BnaGLN1* genes are similarly regulated depending on their orthology group and that they are differentially regulated between groups. Overall it was found that specificities of expression are conserved between *BnaGLN1* genes and their respective *AtGLN1* orthologues, raising the hypothesis of conserved physiological functions.

In *Arabidopsis*, several studies have shown that *AtGLN1.1* is highly induced in leaves during senescence (Guo *et al.*, 2004) and is up-regulated when exogenous N sources are limiting (Lothier *et al.*, 2011). Up-regulation of *AtGLN1.1* under nitrate starvation is in good agreement with the kinetic properties described by Ishiyama *et al.* (2004) that suggested that the high affinity of AtGLN1.1 for ammonium is correlated with a role for the enzyme under low N conditions. The induction of the expression of the two *BnaGLN1.1* genes in older stems and leaves is conserved, as already shown by Buchanan-Wollaston and Ainsworth (1997) and Ochs *et al.* (1999). It was also found that the two *BnaGLN1.1* genes are overexpressed under low nitrate conditions especially at the vegetative stage. As the amino acid residues known to be involved in the high affinity of AtGLN1 towards ammonium are not conserved in any of the BnaGLN1 proteins, no information about the potential kinetic properties can be extrapolated from the protein sequence. Regarding expression profiles, it is at least suspected that BnaGLN1.1 and AtGLN1.1 proteins might have similar roles

In a previous study, it was found that *AtGLN1.2* is slightly induced by leaf ageing and that the expression profile is biphasic, with an increase from young to mature leaves and then a decrease in strongly senescing leaves (Diaz *et al.*, 2008; Lothier *et al.*, 2011). In addition, it was found that *AtGLN1.2* is mainly expressed in roots and leaves under a high N regime (Guo *et al.*, 2004; Ishiyama *et al.*, 2004; Lothier *et al.*, 2011). A detailed functional analysis led to the conclusion that *AtGLN1.2* was involved in primary ammonium assimilation under high N regimes (Ishiyama *et al.*, 2004; Lothier *et al.*, 2011). *BnaGLN1.2* genes (also named *BnGSR2.1* and *BnGSR2.*2) are also more highly expressed in roots than in shoots (Ochs *et al.*, 1999). It is shown here that the two *BnaGLN1.2* genes are more expressed in young leaves than in old leaves and are overexpressed under high N regimes, suggesting a similar role to *AtGLN1.2* in primary ammonium assimilation. Interestingly, a *BraA.GLN1.2* gene, named *BcGS1* (Sun *et al.*, 2010), was found also to be expressed in root and induced under high N regimes, suggesting the conservation of the regulation in the Brassiceae tribe.

Similarly to *AtGLN1.3*, the *BnaGLN1.3* genes are not induced in older leaves at the vegetative stage and do not respond to differential N regimes (Guo *et al.*, 2004; Lothier *et al.*, 2011). The hypothesis about the physiological roles of *AtGLN1.3* in N export via phloem tissues in roots is supported by the high capacity of the enzyme for glutamine synthesis and by the location of *AtGLN1.3* expression in the root vasculature (Ishiyama *et al.*, 2004). It has to be noted that *BnaGLN1.3* genes are the only *BnaGLN1* genes preferentially expressed in stem tissues compared with leaf blades. As stems are richer in vascular tissues than leaf blades, the higher expression of *BnaGLN1.3* in stems might be related to a potential vascular localization that remains to be explored.

*AtGLN1.4* is one of the markers used for leaf senescence (Guo *et al.*, 2004; Wingler *et al.*, 2009). *AtGLN1.4* is induced by N limitation or starvation in both the root and shoot (Ishiyama *et al.*, 2004; Lothier *et al.*, 2011). AtGLN1.4 protein exhibits high affinity towards ammonium and is expressed in the pericycle cells of roots (Ishiyama *et al.*, 2004). Evidence for *BnaGLN1.4a* root expression was found in EST libraries. However, induction of gene expression in leaves in response to ageing and low N regime is not well conserved among the four *BnaGLN1.4* orthologues in regards to developmental stages. The *BnaGLN1.4* genes are significantly overexpressed in senescing leaves and under low nitrate conditions at the vegetative stage. However, this trend is not conserved at the flowering and seed filling stages. *BnaA.GLN1.4*.*a*, *BnaC.GLN1.4.a*, and *BnaC.GLN1.4*.*b* are clearly and significantly less highly expressed in old than in young leaves at these stages. Furthermore, as the residues conferring the high affinity property are partially conserved in BnaGLN1.4 proteins, it might be suspected that *BnaGLN1.4* genes have a role when N resources are low. Surprisingly, in contrast to *Arabidopsis* in which *AtGLN1.4* expression is one of the lowest, *BnaGLN1.4* expression levels are the highest found among all the *BnaGLN1* genes.

In *Arabidopsis*, *AtGLN1.5* expression is known as the lowest of the *AtGLN1* gene family. Expression was mainly found in seeds (Lothier *et al.*, 2011), and very little is known about *AtGLN1.5*. *BnaGLN1.5* gene expression is also very low, and it was not possible to measure it in leaf and stem tissues at flowering and vegetative stages. Similarly to *AtGLN1.*5, *BnaGLN1.5* ESTs were found in reproductive tissues and seeds, thus suggesting specific roles during seed maturation.

### Conclusion

A total of 16 *B. napus GLN1* genes were identified, among which 12 have never been described. The total number of *BnaGLN1* genes, their phylogenetic relationships, and genetic location are in agreement with the evolutionary history of *Brassica* species. Some specificities of expression seemed to be conserved among the Brassiceae tribe and especially between *A. thaliana* and *B. napus*. Regulations arising from plants interactions with their environment (such as N resources), final architecture, and therefore sink–source relations *in planta*, seem to be globally conserved when compared with data available from the *Arabidopsis* model. Considering the architectural, size, and lifespan differences between *A. thaliana* and *B. napus*, it is not surprising to find some differences in gene expression profiles. Also, due to the higher number of *GLN1* genes conserved in the *B. napus* genome, it seems correct to find some specificities in the expression of each *BnaGLN1* in contrast to genes involved in flavonoid biosynthesis that display highly conserved expression profiles between *A. thaliana* and *B. napus* during seed development and are highly dependent on tissue differentiation (Auger *et al.*, 2009). A more detailed localization of *BnaGLN1* gene expression would refine the hypothesis concerning their physiological role. Indeed, the present expression study relied on leaf blade and stem samples consisting of different tissues with contrasting physiological roles, in particular parenchyma and vascular tissues. Such a localization study could be advantageously performed in *Arabidopsis* and *B. napus* to provide a new basis for comparison of the evolution of this gene family.

## Supplementary data

Supplementary data are available at *JXB* online

Figure S1. *BnaGLN1* gene localization on the A or C genome using a panel of various *Brassica* genotypes.

Figure S2. *BnaGLN1* gene localization on LGs using mono- and polysomic additional lines.

Figure S3. Positions of *BnaGLN1* genes on the *B. napus* genetic map.

Figure S4. Alignments of the genomic sequence, deduced mRNA sequence, and contig of EST sequences are reported for each *BnaGLN1* gene.

Figure S5. Expression of *BnaGLN1* genes is modified depending on nitrate availability and ageing in primary veins of vegetative *B. napus* plants.

Table S1. Clones from Genoplante and ADIS-MPIZ oilseed rape cDNA libraries used for *BnaGLN1* mRNA sequencing.

Table S2. Primers used for cloning and genetic mapping of the *BnaGLN1* gene.

Table S3. Specific primers used for sequencing *BnaGLN1* cDNAs.

Table S4. qPCR primers used for *BnaGLN1* gene expression analysis.

Table S5. Induction of *BnaGLN1* gene expression under low N fertilization in field-grown plants.

Data File S1. List and description of EST sequences belonging to each *B. napus*, *B. oleracea.* and *B. rapa* GLN1 contig, and tables of the distribution of ESTs between libraries according to their BnaGLN1 contigs.

Data File S2. BnaGLN1 contig sequences in fasta format.

Data File S3. Global multiple alignment of nucleotide sequences in the study.

Data File S4. Genomic sequences of the *BnaGLN1* genes.

Data File S5. Deduced *BnaGLN1* mRNA sequences.

Supplementary Data

## Abbreviations:

GS: glutamine synthetase
GLN1: cytosolic glutamine synthetase gene
GSL: chloroplastic glutamine synthetase gene.

## References

[CIT0001] Albert B Le Caherec F Niogret MF Faes P Avice JC Leport L Bouchereau A 2012 Nitrogen availability impacts oilseed rape (Brassica napus L.) plant water status and proline production efficiency under water-limited conditions. Planta 236, 659–676 22526495 10.1007/s00425-012-1636-8 PMC3404282

[CIT0002] Altschul SF Lipman DJ 1990 Protein database searches for multiple alignments. Proceedings of the National Academy of Sciences, USA 87, 5509–5513 10.1073/pnas.87.14.5509 PMC54354 2196570

[CIT0003] Auger B Baron C Lucas M Vautrin S Berges H Chalhoub B Fautrel A Renard M Nesi N 2009 Brassica orthologs from BANYULS belong to a small multigene family, which is involved in procyanidin accumulation in the seed. Planta 230, 1167–1183 19760260 10.1007/s00425-009-1017-0 PMC2764081

[CIT0004] Bekaert M Edger PP Pires JC Conant GC 2011 Two-phase resolution of polyploidy in the Arabidopsis metabolic network gives rise to relative and absolute dosage constraints. The Plant Cell 23, 1719–1728 21540436 10.1105/tpc.110.081281 PMC3123947

[CIT0005] Bernard SM Habash DZ 2009 The importance of cytosolic glutamine synthetase in nitrogen assimilation and recycling. New Phytologist 182, 608–620 19422547 10.1111/j.1469-8137.2009.02823.x

[CIT0006] Brauc S De Vooght E Claeys M Hofte M Angenon G 2011 Influence of over-expression of cytosolic aspartate aminotransferase on amino acid metabolism and defence responses against Botrytis cinerea infection in Arabidopsis thaliana. Journal of Plant Physiology 168, 1813–1819 21676488 10.1016/j.jplph.2011.05.012

[CIT0007] Buchanan-Wollaston V Ainsworth C 1997 Leaf senescence in Brassica napus: cloning of senescence related genes by subtractive hybridisation. Plant Molecular Biology 33, 821–834 9106506 10.1023/a:1005774212410

[CIT0008] Chen ZJ Pikaard CS 1997 Transcriptional analysis of nucleolar dominance in polyploid plants: biased expression/silencing of progenitor rRNA genes is developmentally regulated in Brassica. Proceedings of the National Academy of Sciences, USA 94, 3442–3447 10.1073/pnas.94.7.3442 PMC20389 9096413

[CIT0009] Cheng F Liu S Wu J Fang L Sun S Liu B Li P Hua W Wang X 2011 BRAD, the genetics and genomics database for Brassica plants. BMC Plant Biology 11, 136 21995777 10.1186/1471-2229-11-136 PMC3213011

[CIT0010] Colnenne C Meynard JM Reau R Justes E Merrien A 1998 Determination of a critical nitrogen dilution curve for winter oilseed rape. Annals of Botany 81, 311–317

[CIT0011] Czechowski T Stitt M Altmann T Udvardi MK Scheible WR 2005 Genome-wide identification and testing of superior reference genes for transcript normalization in Arabidopsis. Plant Physiology 139, 5–17 16166256 10.1104/pp.105.063743 PMC1203353

[CIT0012] De Smet R Van de Peer Y 2012 Redundancy and rewiring of genetic networks following genome-wide duplication events. Current Opinion in Plant Biology 15, 168–176 22305522 10.1016/j.pbi.2012.01.003

[CIT0013] Delourme R Falentin C Huteau V 2006 Genetic control of oil content in oilseed rape (Brassica napus L.). Theoretical and Applied Genetics 113, 1331–1345 16960716 10.1007/s00122-006-0386-z

[CIT0014] Diaz C Lemaitre T Christ A Azzopardi M Kato Y Sato F Morot-Gaudry JF Le Dily F Masclaux-Daubresse C 2008 Nitrogen recycling and remobilization are differentially controlled by leaf senescence and development stage in Arabidopsis under low nitrogen nutrition. Plant Physiology 147, 1437–1449 18467460 10.1104/pp.108.119040 PMC2442554

[CIT0015] Eisenberg D Gill HS Pfluegl GMU Rotstein SH 2000 Structure–function relationships of glutamine synthetases. Biochimica et Biophysica Acta 1477, 122–145 10708854 10.1016/s0167-4838(99)00270-8

[CIT0016] Franzke A Lysak MA Al-Shehbaz IA Koch MA Mummenhoff K 2011 Cabbage family affairs: the evolutionary history of Brassicaceae. Trends in Plant Science 16, 108–116 21177137 10.1016/j.tplants.2010.11.005

[CIT0017] Guo Y Cai Z Gan S 2004 Transcriptome of Arabidopsis leaf senescence. Plant, Cell and Environment 27, 521–549

[CIT0018] Herrera-Rodriguez MB Maldonado JM Perez-Vicente R 2006 Role of asparagine and asparagine synthetase genes in sunflower (Helianthus annuus) germination and natural senescence. Journal of Plant Physiology 163, 1061–1070 16368161 10.1016/j.jplph.2005.10.012

[CIT0019] Herrera-Rodriguez MB Perez-Vicente R Maldonado JM 2007 Expression of asparagine synthetase genes in sunflower (Helianthus annuus) under various environmental stresses. Plant Physiology and Biochemistry 45, 33–38 17258907 10.1016/j.plaphy.2006.12.002

[CIT0020] Higgins J Magusin A Trick M Fraser F Bancroft I 2012 Use of mRNA-seq to discriminate contributions to the transcriptome from the constituent genomes of the polyploid crop species Brassica napus. BMC Genomics 13, 247 22703051 10.1186/1471-2164-13-247 PMC3428664

[CIT0021] Hirel B Bertin P Quillere I 2001 Towards a better understanding of the genetic and physiological basis for nitrogen use efficiency in maize. Plant Physiology 125, 1258–1270 11244107 10.1104/pp.125.3.1258 PMC65606

[CIT0022] Hirel B Le Gouis J Ney B Gallais A 2007 The challenge of improving nitrogen use efficiency in crop plants: towards a more central role for genetic variability and quantitative genetics within integrated approaches. Journal of Experimental Botany 58, 2369–2387 17556767 10.1093/jxb/erm097

[CIT0023] Huang XQ Madan A 1999 CAP3: a DNA sequence assembly program. Genome Research 9, 868–877 10508846 10.1101/gr.9.9.868 PMC310812

[CIT0024] Inaba R Nishio T 2002 Phylogenetic analysis of Brassiceae based on the nucleotide sequences of the S-locus related gene, SLR1. Theoretical and Applied Genetics 105, 1159–1165 12582894 10.1007/s00122-002-0968-3

[CIT0025] Ishiyama K Inoue E Watanabe-Takahashi A Obara M Yamaya T Takahashi H 2004 Kinetic properties and ammonium-dependent regulation of cytosolic isoenzymes of glutamine synthetase in Arabidopsis. Journal of Biological Chemistry 279, 16598–16605 14757761 10.1074/jbc.M313710200

[CIT0026] Ishiyama K Inoue E Yamaya T Takahashi H 2006 Gln49 and Ser174 residues play critical roles in determining the catalytic efficiencies of plant glutamine synthetase. Plant and Cell Physiology 47, 299–303 16338958 10.1093/pcp/pci238

[CIT0027] Kosambi DD 1944 The estimation of map distance from recombination values. Annals of Eugenics 12, 172–175

[CIT0028] Laine P Ourry A Macduff J Boucaud J Salette J 1993 Kinetic-parameters of nitrate uptake by different catch crop species—effects of low-temperatures or previous nitrate starvation. Physiologia Plantarum 88, 85–92

[CIT0029] Lander ES Green P Abrahamson J Barlow A Daly MJ Lincoln SE Newberg LA Newburg L 1987 MAPMAKER: an interactive computer package for constructing primary genetic linkage maps of experimental and natural populations. Genomics 1, 174–181 3692487 10.1016/0888-7543(87)90010-3

[CIT0030] Lombard V Delourme R 2001 A consensus linkage map for rapeseed (Brassica napus L.): construction and integration of three individual maps from DH populations. Theoretical and Applied Genetics 103, 491–507

[CIT0031] Lothier J Gaufichon L Sormani R 2011 The cytosolic glutamine synthetase GLN1;2 plays a role in the control of plant growth and ammonium homeostasis in Arabidopsis rosettes when nitrate supply is not limiting. Journal of Experimental Botany 62, 1375–1390 20959627 10.1093/jxb/erq299

[CIT0032] Lysak MA Koch MA Pecinka A Schubert I 2005 Chromosome triplication found across the tribe Brassiceae. Genome Research 15, 516–525 15781573 10.1101/gr.3531105 PMC1074366

[CIT0033] Makowski D Maltas A Morison M Reau R 2005 Calculating N fertilizer doses for oil-seed rape using plant and soil data. Agronomy for Sustainable Development 25, 159–161

[CIT0034] Malagoli P Laine P Rossato L Ourry A 2005 Dynamics of nitrogen uptake and mobilization in field-grown winter oilseed rape (Brassica napus) from stem extension to harvest—I. Global N flows between vegetative and reproductive tissues in relation to leaf fall and their residual N. Annals of Botany 95, 853–861 15701662 10.1093/aob/mci091 PMC4246740

[CIT0035] Marchler-Bauer A Lu S Anderson JB 2011 CDD: a conserved domain database for the functional annotation of proteins. Nucleic Acids Research 39, D225–D229 21109532 10.1093/nar/gkq1189 PMC3013737

[CIT0036] Martin A Belastegui-Macadam X Quillere I Floriot M Valadier MH Pommel B Andrieu B Donnison I Hirel B 2005 Nitrogen management and senescence in two maize hybrids differing in the persistence of leaf greenness: agronomic, physiological and molecular aspects. New Phytologist 167, 483–492 15998400 10.1111/j.1469-8137.2005.01430.x

[CIT0037] Martin A Lee J Kichey T 2006 Two cytosolic glutamine synthetase isoforms of maize are specifically involved in the control of grain production. The Plant Cell 18, 3252–3274 17138698 10.1105/tpc.106.042689 PMC1693956

[CIT0038] Masclaux C Quillere I Gallais A Hirel B 2001 The challenge of remobilisation in plant nitrogen economy. A survey of physio-agronomic and molecular approaches. Annals of Applied Biology 138, 69–81

[CIT0039] Masclaux C Valadier MH Brugiere N Morot-Gaudry JF Hirel B 2000 Characterization of the sink/source transition in tobacco (Nicotiana tabacum L.) shoots in relation to nitrogen management and leaf senescence. Planta 211, 510–518 11030550 10.1007/s004250000310

[CIT0040] Masclaux-Daubresse C Reisdorf-Cren M Orsel M 2008 Leaf nitrogen remobilisation for plant development and grain filling. Plant Biology (Stuttgart) 10 Suppl 1, 23–36 10.1111/j.1438-8677.2008.00097.x 18721309

[CIT0041] Masclaux-Daubresse C Reisdorf-Cren M Pageau K Lelandais M Grandjean O Kronenberger J Valadier MH Feraud M Jouglet T Suzuki A 2006 Glutamine synthetase–glutamate synthase pathway and glutamate dehydrogenase play distinct roles in the sink–source nitrogen cycle in tobacco. Plant Physiology 140, 444–456 16407450 10.1104/pp.105.071910 PMC1361315

[CIT0042] Nagaharu U 1935 Genome analysis in *Brassica* with special reference to the experimental formation of *B. napus* and peculliar mode of fertilisation. Japanese Journal of Botany 7, 389–452

[CIT0043] Ochs G Schock G Trischler M Kosemund K Wild A 1999 Complexity and expression of the glutamine synthetase multigene family in the amphidiploid crop Brassica napus. Plant Molecular Biology 39, 395–405 10092169 10.1023/a:1006193717093

[CIT0044] Ostergaard L King GJ 2008 Standardized gene nomenclature for the Brassica genus. Plant Methods 4, 10 18492252 10.1186/1746-4811-4-10 PMC2408569

[CIT0045] Parkin IAP Gulden SM Sharpe AG Lukens L Trick M Osborn TC Lydiate DJ 2005 Segmental structure of the Brassica napus genome based on comparative analysis with Arabidopsis thaliana. Genetics 171, 765–781 16020789 10.1534/genetics.105.042093 PMC1456786

[CIT0046] Paterson AH Lan T-h Amasino R Osborn TC Quiros C 2001 Brassica genomics: a complement to, and early beneficiary of, the Arabidopsis sequence. Genome Biology 2, REVIEWS1011 10.1186/gb-2001-2-3-reviews1011 PMC138917 11276431

[CIT0047] Pumphrey M Bai J Laudencia-Chingcuanco D Anderson O Gill BS 2009 Nonadditive expression of homoeologous genes is established upon polyploidization in hexaploid wheat. Genetics 181, 1147–1157 19104075 10.1534/genetics.108.096941 PMC2651049

[CIT0048] Rana D Boogaart T O’Neill CM Hynes L Bent E Macpherson L Park JY Lim YP Bancroft I 2004 Conservation of the microstructure of genome segments in Brassica napus and its diploid relatives. The Plant Journal 40, 725–733 15546355 10.1111/j.1365-313X.2004.02244.x

[CIT0049] Rapp RA Udall JA Wendel JF 2009 Genomic expression dominance in allopolyploids. BMC Biology 7, 10 19409075 10.1186/1741-7007-7-18 PMC2684529

[CIT0050] Rathke GW Behrens T Diepenbrock W 2006 Integrated nitrogen management strategies to improve seed yield, oil content and nitrogen efficiency of winter oilseed rape (Brassica napus L.): a review. Agriculture Ecosystems and Environment 11, 80–108

[CIT0051] Ritz C Spiess A-N 2008 qpcR: an R package for sigmoidal model selection in quantitative real-time polymerase chain reaction analysis. Bioinformatics 24, 1549–1551 18482995 10.1093/bioinformatics/btn227

[CIT0052] Rossato L Laine P Ourry A 2001 Nitrogen storage and remobilization in Brassica napus L. during the growth cycle: nitrogen fluxes within the plant and changes in soluble protein patterns. Journal of Experimental Botany 52, 1655–1663 11479330

[CIT0053] Rutledge RG 2004 Sigmoidal curve-fitting redefines quantitative real-time PCR with the prospective of developing automated high-throughput applications. Nucleic Acids Research 32, e178 15601990 10.1093/nar/gnh177 PMC545475

[CIT0054] Schjoerring JK Bock JGH Gammelvind L Jensen CR Mogensen VO 1995 Nitrogen incorporation and remobilization in different shoot components of field-grown winter oilseed rape (Brassica napus L) as affected by rate of nitrogen application and irrigation. Plant and Soil 177, 255–264

[CIT0055] Schranz ME Lysak MA Mitchell-Olds T 2006 The ABC’s of comparative genomics in the Brassicaceae: building blocks of crucifer genomes. Trends in Plant Science 11, 535–542 17029932 10.1016/j.tplants.2006.09.002

[CIT0056] Seifi HS Van Bockhaven J Angenon G Hofte M 2013 Glutamate metabolism in plant disease and defense: friend or foe? Molecular Plant-Microbe Interactions 26, 475–485 23342972 10.1094/MPMI-07-12-0176-CR

[CIT0057] Soltis D Albert V Leebens-Mack J Bell C Paterson A Zheng C Sankoff D dePamphilis C Wall P Soltis P 2009 Polyploidy and angiosperm diversification. American Journal of Botany 96, 336–348 21628192 10.3732/ajb.0800079

[CIT0058] Sun FF Yang XD Li Y Hou XL 2010 Molecular cloning and characterisation of cytoplasmic glutamine synthetase gene BcGS1 from non-heading Chinese cabbage. Journal of the Science of Food and Agriculture 90, 891–897 20355127 10.1002/jsfa.3900

[CIT0059] Swarbreck SM Defoin-Platel M Hindle M Saqi M Habash DZ 2011 New perspectives on glutamine synthetase in grasses. Journal of Experimental Botany 62, 1511–1522 21172814 10.1093/jxb/erq356

[CIT0060] Tabuchi M Sugiyama K Ishiyama K Inoue E Sato T Takahashi H Yamaya T 2005 Severe reduction in growth rate and grain filling of rice mutants lacking OsGS1;1, a cytosolic glutamine synthetase1;1. The Plant Journal 42, 641–651 15918879 10.1111/j.1365-313X.2005.02406.x

[CIT0061] Thompson JD Higgins DG Gibson TJ 1994 CLUSTAL-W—improving the sensitivity of progressive multiple sequence alignment through sequence weighting, position-specific gap penalties and weight matrix choice. Nucleic Acids Research 22, 4673–4680 7984417 10.1093/nar/22.22.4673 PMC308517

[CIT0062] Tilsner J Kassner N Struck C Lohaus G 2005 Amino acid contents and transport in oilseed rape (Brassica napus L.) under different nitrogen conditions. Planta 221, 328–338 15599760 10.1007/s00425-004-1446-8

[CIT0063] Town CD Cheung F Maiti R 2006 Comparative genomics of Brassica oleracea and Arabidopsis thaliana reveal gene loss, fragmentation, and dispersal after polyploidy. The Plant Cell 18, 1348–1359 16632643 10.1105/tpc.106.041665 PMC1475499

[CIT0064] Vandesompele J De Preter K Pattyn F Poppe B Van Roy N De Paepe A Speleman F 2002 Accurate normalization of real-time quantitative RT-PCR data by geometric averaging of multiple internal control genes. Genome Biology 3, RESEARCH0034. 10.1186/gb-2002-3-7-research0034 PMC126239 12184808

[CIT0065] Wang J Lydiate DJ Parkin IAP Falentin C Delourme R Carion PWC King GJ 2011 Integration of linkage maps for the Amphidiploid Brassica napus and comparative mapping with Arabidopsis and Brassica rapa. BMC Genomics 12, 101 21306613 10.1186/1471-2164-12-101 PMC3042011

[CIT0066] Wang X Wang H Wang J 2011 The genome of the mesopolyploid crop species Brassica rapa. Nature Genetics 43, 1035–1039 21873998 10.1038/ng.919

[CIT0067] Wingler A Masclaux-Daubresse C Fischer AM 2009 Sugars, senescence, and ageing in plants and heterotrophic organisms. Journal of Experimental Botany 60, 1063–1066 19276191 10.1093/jxb/erp067

