# Supplementary material for: Sixteen cytosolic glutamine synthetase genes identified in the Brassica napus L. genome are differentially regulated depending on nitrogen regimes and leaf senescence
Source: J Exp Bot. 2014 Feb 24;65(14):3927–47. doi: 10.1093/jxb/eru041 (PMC4106436; doi:10.1093/jxb/eru041)
Supplement: Supplementary Data [file supp_65_14_3927__index.html]

Sixteen cytosolic glutamine synthetase genes identified in the Brassica napus L. genome are differentially regulated depending on nitrogen regimes and leaf senescence — Sixteen cytosolic glutamine synthetase genes identified in the Brassica napus L. genome are differentially regulated depending on nitrogen regimes and leaf senescence — Supplementary Data 

# Sixteen cytosolic glutamine synthetase *genes* identified in the *Brassica napus* L. genome are differentially regulated depending on nitrogen regimes and leaf senescence

## Supplementary Data

Data files

**Files in this Data Supplement:**

- Supplementary Data - Supplementary Data
- Supplementary Data - Supplementary Data
- Supplementary Data - Supplementary Data
- Supplementary Data - Supplementary Data
- Supplementary Data - Supplementary Data
- Supplementary Data - Supplementary Data
