# Supplementary material for: Sixteen cytosolic glutamine synthetase genes identified in the Brassica napus L. genome are differentially regulated depending on nitrogen regimes and leaf senescence
Source: J Exp Bot. 2014 Feb 24;65(14):3927–47. doi: 10.1093/jxb/eru041 (PMC4106436; doi:10.1093/jxb/eru041)
Supplement: Supplementary Data [file supp_eru041_jexbot114215_file006.pdf]

Journal of Experimental Botany

Sixteen cytosolic glutamine synthetase *genes* identified in *Brassica napus* L. genome are differentially regulated depending on nitrogen regimes and leaf senescence

Mathilde Orsel, Michaël Moison, Vanessa Clouet, Justine Thomas, Françoise Leprince, Anne-Sophie Canoy, Jérémy Just, Boulos Chalhoub, Céline Masclaux-Daubresse.

5 Supplementary Tables
5 Supplementary Figures
5 supplementary Data Files

Supplementary Table S1: Clones from Genoplante and ADIS-MPIZ oilseedrape cDNA libraries used for *BnaGLN1* mRNA sequencing.

A. thaliana	***B. napus***				
**Gene**	**Gene**	**Accession (mRNA name)**	**Clone**	**Library (NCBI ID)**	**Genotype**
AtGLN1.1	*BnaA.GLN1.1.a*	X82997 (BnGSR2.2)			Arabella
	BnaC.GLN1.1.a	Y12460 (BnGSR2.2)[b]			Arabella
AtGLN1.2	*BnaA.GLN1.2.a*	X76736 (BnGSR1.1)			Arabella
	BnaC.GLN1.2.a	Y12459 (BnGSR1.2)			Arabella
AtGLN1.3	*BnaC.GLN1.3.a*	JX306690	MPIZp1022G0813Q	ADIS-MPIZ 021 (Lib.16807)	Express 617
	BnaA.GLN1.3.a	JX306693	BN20.052B22	Genoplante BN20 (Lib.13978)	JetNeuf
	BnaA.GLN1.3.c	JX306694	BN40.043K19	Genoplante BN40 (Lib.13980)	JetNeuf
AtGLN1.4	*BnaA.GLN1.4.a*	JX306696[a]			Express
		JX306697			Tenor
		JX306692[a]	MPIZp1022C0924Q	ADIS-MPIZ 021 (Lib.16807)	Express 617
	BnaC.GLN1.4.b	JX306698[b]			Express
		JX306699[b]			Tenor
	BnaA.GLN1.4.b	JX306700[b]			Express
		JX306701[b]			Tenor
	BnaC.GLN1.4.a	JX306695	BN15.001A07	Genoplante BN15 (Lib.13977)	JetNeuf
AtGLN1.5	*BnaC.GLN1.5.a*	JX306691[b]	MPIZp1022A1123Q	ADIS-MPIZ 021 (Lib.16807)	Express 617

[a] cDNA clone with SNP insertion disrupting the ORF when compared to *A. thaliana* cds reference sequence and BnaGLN1 contig.
[b] Incomplete cds when compared to *A. thaliana* cds reference sequence.

Supplementary Table S2: Primers used for cloning and genetic mapping of *BnaGLN1* gene.

A. thaliana	***B. napus***				
**Gene**	**Gene**	**Primer couple F / R primer name**	**Forward primer sequence**	**Reverse primer sequence**	**Usage**
AtGLN1.1	*BnaA.GLN1.1.a*	BnGSR2.1 U / L	TCTTGGTTGGCCTATTGGC	GCGTATAAGCAGGCCTTGTAG	Panel [a], add [b]
	BnaC.GLN1.1.a	BnGS1.9 U / L	TGGTATCTTTTGACCCAAAACCT	CGGATTGATGCTCCACGA	Panel [a], add [b]
AtGLN1.2	*BnaA.GLN1.2.a*	BnGS1.82 U / L	GAAGTGTGGATTGCTCGTTTT	ACCCCATTTGAAAGTGTTGATA	Panel [a]
	BnaC.GLN1.2.a	BnGSR1-2_Y12459_cDNA-M1-F / R	CTACAAGGCTTGTTTGTATGCC	CGAGCAATCCACACTTCATC	Panel [a], add [b]
	BnaC.GLN1.2.a	BnGS1.81 U / L	ATGAAGTGTGGATTGCTCGTTAC	GTGTGAGCACCAGCTCCA	SD [c]
AtGLN1.3	*BnaC.GLN1.3.a*	BnGS1.1 U / L	GTGACATCGTGGATGCG	CAAGAGAACGTGTTGATGTCC	Panel [a], add [b]
	BnaA.GLN1.3.a	BnGS1.2 U / L	GGGAGCTGACAAGGCAA	GCCTTCTTTCTCAGTGTCTCTC	DS [d], Panel [a], add [b]
	BnaC.GLN1.3.b	BnGS1.31 UP2 / RP2	AGCTTCGACCCAAAACC	GCTTTCTTTATCACCGCTAGT	Panel [a], add [b]
	BnaA.GLN1.3.b	BnGS1.32 U / L	TTGAAGGGATTAGTGCC	TACTGTAGTTGCAGTGCG	Panel [a]
	BnaA.GLN1.3.c	BnGS1.41 U / L	ACGTTGCCAAGGAGGTG	CGGAACTGGTTTAGGGTCG	DS [d], Panel [a]
	BnaC.GLN1.3.c	BnGS1.42 U / L	TTGCCAAAGAAGTGCCTT	AGGGAAGCCACCAACAG	SD [c] ,Panel [a], add [b]
AtGLN1.4	*BnaA.GLN1.4.a*	BnGS1.61 5U / 5L	TTCAGATTCCTAGAAAAATGTCG	TCAAGGTTTCTTCCTTCATGG	Panel [a] , cloning
	BnaC.GLN1.4.a	BnGS1.7 2U / 2L	TGTCGGCTCTTGCAGATTTAAT	ATCATCGCCGGGAGCTTGC	Panel [a] , cloning
		BnGS1.7 4U / 5L	CTCCCGGCGATGATAGTG	TTCCTCATTCATGGTTTCCAG	Cloning
	BnaA.GLN1.4.b	BnGS1.64 5U / 5L	ATCCCGGCAAACAAAAGG	TTTTGTTTGCCGGGATCG	Panel [a] , cloning
		BnGS1.64 2U / 2L	TCTCGATCTCTCCGATTCCA	CCGGCCATTTAATATCCTTTT	Cloning
	BnaC.GLN1.4.b	BnGS1.63 4U / 5L	GTGAACCGATTCCGACGAAC	TTCTTCATTCATGGCTTCCA	Panel [a]
		BnGS1.63 2U / 3L	GATCAATCTCGATCTCTCCGATTA	CCTACCGGCCACTTAATATCC	Cloning
AtGLN1.5	*BnaC.GLN1.5.a*	BnGS1.51 U / L	AGGGAAGCCACCAACAG	TCATGCTTCCCAGTGAGG	Panel [a]
	BnaA.GLN1.5.a	BnGS1.52 U / L	CAGAAAAAATGTCTCCACTCTCC	CCATCCACAATGTCACGC	DY [e], Panel [a]

[a] Panel of *B. napus*, *B. oleracea* and *B. rapa* genotypes.
[b] Monnosomic and polysomic addition lines obtained from a cross Darmor-*bzh* x C1.3.
[c] Mapping population Stellar x Drakar.
[d] Mapping population Darmor x Smouraï.
[e] Mapping population Darmor-*bzh* x Yudal.

Supplementary Table S3: Specific primers used for sequencing *BnaGLN1* cDNAs.

A. thaliana	***B. napus***			
**Gene**	**Gene**	**Accession**	**Primer name**	**Primer sequence**
AtGLN1.3	*BnaC.GLN1.3.a*	JX306690	GS1-1-13G8-1	CGATGAGGAACGACGGAG
			GS1.1-rev-1	GAAACTAGGAAGAAAAAAAA
			GS1.1-rev-2	CCAGTTCACACCCTTTTG
	BnaA.GLN1.3.a	JX306693	BnGS_ContigEST2_1	GCTAGATACCTTCTCGAGAG
			GS1.2-52B22-1	CGGTGAGAGTGGGGAGAGAC
			GS1.2_EST_1	TCAGCTTCGACCCTAAACCA
			GS1.2-rev-1	TTATTGAAACCGAAATAGAC
			GS1.2-rev-2	TTGTGCCTCTTGTTGGTC
	BnaA.GLN1.3.c	JX306694	BnGS_ContigEST4_1	AACCAGTTCCGGGTGATTGG
			GS1.41_EST_1	GTGATTGGAACGGAGCTGGA
			GS1-41-rev-1	CATCATGAAAATCCTTTATTT
			GS1-41-rev-2	CGTTAGGATGGCTAAAGA
AtGLN1.4	*BnaA.GLN1.4.a*	JX306692	BnGS_ContigEST6_1	GTAGATGCTCATTACAAAGC
			GS1-61-24C9-1	TGGAAGACACGAGACTGCTG
			GS1.61-rev-1	TTATTTCTTGGAAAGAGTTT
			GS1.61-rev-2	GTAGTATGGTCCCTGAGGA
		JX306696	GS1-61-6U	CACCAAGTCGATGAGGGAAG
		JX306697	GS1-61-3L	CTTCCCTCATCGACTTGGTG
	BnaC.GLN1.4.a	JX306695	BnGS_ContigEST7_1	GTTGTTTTATCTCTTGACCC
			GS1.7_EST_1	CGATGAGAGAAGATGGAGGG
			GS1-7-rev-1	GACCGAGACTTGAATCTTGT
			GS1-7-rev-2	ATACCATGTTTCTTCGGC
	BnaC.GLN1.4.b	JX306698	GS1-63-2U	GATCAATCTCGATCTCTCCGATTA
		JX306699	GS1-63-L	CCAACCTACCGGCCATTTA
	BnaA.GLN1.4.b	JX306700	GS1-64-2U	TCTCGATCTCTCCGATTCCA
		JX306701	GS1-64-2L	CCGGCCATTTAATATCCTTTT
AtGLN1.5	*BnaC.GLN1.5.a*	JX306691	GS1-51-23A11-1	GGAGCATCGGTGAGAGTGG
			GS1.51-rev-1	CAAACTGAAATTAAACCG
			GS1.51-rev-2	ACCAGGAAAGCCACCAAG

Supplementary Table S4: qPCR Primers used for *BnaGLN1* gene expression analysis.

A. thaliana	***B. napus***				
**Reference gene**	**Target gene(s)**	**Reference sequence or accession**	**Primer names forward / reverse**	**Forward primer sequence**	**Reverse primer sequence**
At3g01150 (AtPTB)	*BnaX.PTB.a*	EV058089	BnPTB-UP / RP	AGCGAAAGAAGCATTGGAAG	CAGGAGTAGGCTCAGATCAGG
At1g13320 (AtPP2A)	*BnaX.PP2A.a*	EE464526	BnPP2A-UP1 /RP1	GCTTCAATCCCTCAATCCCTCATTCCAA	ATCCTCGCTTAGCTCCACAA
At1g07920 (AtEF1α)	*BnaX.EF1.a*	CD827228	Q-BnEF1-1-UP6 /RP6	CGGTGTCATCAAGAGCGTTG	GGACTTTGATTCATCTGCGGAT
At2g28390 (AtSAND)	*BnaX.SAND.a*	EV384210	BnSAND-UP / RP	CTGCTATGCTGGGTCACTCC	TGATTGCATATCTTTATCGCCATC
At5g25760 (AtUBC21)	*BnaX.UBC21.a*	EE493452	UBC21-UP1 /RP1	GTCCTCTCAACTGCGACTCA	GTGTGTACATGCGTGCCATT
At5g37600 (AtGLN1.1)	*BnaA.GLN1.1.a*	BnaGLN1.1_C1	BnGSR2-1_X82997_cDNA-M1-F / R	TCTTGGTTGGCCTATTGGC	GCGTATAAGCAGGCCTTGTAG
	BnaC.GLN1.1.a	BnaGLN1.1_C2	BnGSR2-2_Y12460_cDNA-M2-F / R	CCTGTTGGTTGGCCTATTG	TCCATTGATGCCACTAATGG
At1g66200 (AtGLN1.2)	*BnaA.GLN1.2.a*	BnaGLN1.2_C1	BnGSR1-1_X76736_cDNA-M1-F / R	AAAGATGTGAATTGGCCTGTC	GCATCAACAATGTCTCTTCCA
	BnaC.GLN1.2.a	BnaGLN1.2_C2	BnGSR1-2_Y12459_cDNA-M1-F / R	CTACAAGGCTTGTTTGTATGCC	CGAGCAATCCACACTTCATC
At3g17820 (AtGLN1.3)	*BnaC.GLN1.3.a*	BnaGLN1.3_C1	BnGS_contigEST_1-M3-F / R	GTGACATCGTGGATGCG	AACAGGACCGACTTGGAAC
	BnaA.GLN1.3.a	BnaGLN1.3_C2	BnGS_contigEST_2-M1-F / R	GAGCGATCCATCAAAGCTG	TGAACGGATCACGGAATATC
	BnaC.GLN1.3.b	BnaGLN1.3_C3	BnGS1_31_UP2 / RP2	AGCTTCGACCCAAAACC	GCTTTCTTTATCACCGCTAGT
	BnaA.GLN1.3.b	BnaGLN1.3_C4	BnGS1_32_UP2 / RP2	CTGTTGAAGGGATTAGTGCC	GGACTGGTTTTGGGTCG
	Bna.GLN1.3.c [a]	BnaGLN1.3_C5	BnGS_contigEST_4-M2-F / R	ATGCATACACACCGGCC	TCTTGCTCAATCCCATACCA
At5g16570 (AtGLN1.4)	*BnaA.GLN1.4.a*	BnaGLN1.4_C1	BnGS_contigEST_6-M1-F / R	GAACCGATCCCAACCAAC	GGCCACTTAGTATCCTTTTGG
	BnaC.GLN1.4.a	BnaGLN1.4_C2	BnGS_contigEST_7-M2-F / R	GCTGGAATCAATGTCAGTGG	CGACCCAGACCTGATCG
	BnaC.GLN1.4.b	BnaGLN1.4_C3	BnGS1_63_UP2 / RP2	GACATAGTAGATTCTCATTACAAAGCC	CGAGCGACCCAGACTTG
	BnaA.GLN1.4.b	BnaGLN1.4_C4	BnGS1_64_UP2 / RP2	AGCTGGGAAAGGGTACTTC	ATGGCTTCCAGAGGATTGT
At1g48470 (AtGLN1.5)	*Bna.GLN1.5.a* [a]	BnaGLN1.5_C1	BnGS_contigEST_5-M2-F / R	TCGATCATCCCAATGTGAAG	CAATAGTACGGTCCCTGAGG
At5g35630 (AtGLN2)	*BnGSL1*	X72751	BnGSL1_X72751_cDNA-M3-F / R	GGAGCGTATCCTGGTCCC	GTCCTGGCATAACTTCACCA
	BnGSL2	Y12458	BnGSL2_Y12458_cDNA-M1-F / R	ATTCCAAGTTGGCCCCGA	AACCAGCACCGTTCCAGT

[a] Primers are not specific to one target gene but towards both BnaA.GLN1 and BnaC.GLN1 genes.

Supplementary Table S5: Induction of *BnaGLN1* genes expression under low N fertilisation in field grown plants. *BnaGLN1* genes expression level were monitored in limbs and stems from plants grown in field, supplemented (HN) or not (LN) with nitrogen, in young and old tissues harvested at early flowering and seed filling stages (60 and 71 to 73 on BBCH scale). Data are presented as Log Ratio defined as $\log_2$(LN/HN). * indicate significant differences (Student Test, $p<0.05$), mean of four plants repeat.

	Early flowering				Seed filling			
	Limb		Stem		Limb		Stem	
	Old	Young	Old	Young	Old	Young	Old	Young
BnaA.GLN1.1.a	0.06	0.44*	0.18	1.71*	-0.01	-0.03	-0.83	-0.26
BnaC.GLN1.1.a	-0.12	0.23	0.46	1.22*	-0.25	0.00	-0.84	-0.38
BnaA.GLN1.2.a	-0.06	-0.43*	-0.09	-0.06	-0.50	-0.26	-1.53	-0.33
BnaC.GLN1.2.a	-0.21	-0.49*	0.11	0.00	-0.40	0.04	-1.22*	0.12
BnaA.GLN1.3.a	0.20	-0.29	-0.33	-0.18	0.26	0.16	-0.76*	-0.33
BnaC.GLN1.3.a	0.27*	-0.05	-0.37	-0.07	0.43*	0.26	-0.74*	0.02
BnaA.GLN1.3.b	1.00*	-0.51*	-0.19	-0.01	-0.07	0.09	-1.38	-0.21
BnaC.GLN1.3.b	0.90	-0.38	-0.32	-0.12	0.14	0.25	-0.35	0.19
BnaGLN1.3.c	-0.18	-0.05	0.01	-0.01	0.37	-0.14	-0.86	-0.17
BnaA.GLN1.4.a	0.27	0.20	0.47	0.96*	0.44	0.13	-0.88	-0.41
BnaC.GLN1.4.a	0.28	-0.03	0.65*	0.70*	0.21	0.05	-0.40	-0.23
BnaA.GLN1.4.b	-0.52	0.22	0.95	0.84*	0.20	-0.45	-0.01	-0.06
BnaC.GLN1.4.b	-0.48	-0.48*	1.97*	0.72*	0.47	-0.02	0.39*	0.07
BnGSL1	0.29	-0.13	0.92	0.23	0.26	0.34	0.40*	-0.01
BnGSL2	0.14	-0.14	0.86	0.10	0.25	0.17	0.04*	-0.24

* Statistically significant induction under LN (bilateral t test, p-value < 0.05).

Supplementary Figure legends

Supplementary Figure S1: BnaGLN1 genes localisation on A or C genome using a panel of various Brassica genotypes. Amplification of DNA fragments for each BnaGLN1 gene on Brassica napus (1, Darmor-bzh; 2, Yudal; 3, Stellar; 4, Drakkar; 5, Samouraï; 6, Aburamasari; 7, Aviso; 8, Tenor; 9, Express; 10, Montego), Brassica rapa (11, Z1; 12, C1.3; 13, Chiifu) and Brassica oleracea (14, HDEM; 15, C102) were performed using specific primers designed for each gene (Supplementary Table S1) in order to reveal a presence / absence polymorphism between B. rapa (A) and B. oleracea (C) genomes. Water control is presented (lane 16). Genes in bold letters are also mapped on the genetic map (Supplementary Figure S3). Stars indicate genes for which there is no agreement with the genetic mapping performed on segregating populations or for which some results remain uncertain.

Supplementary Figure S2: BnaGLN1 genes localisation on LG using mono- and polysomic additional lines. Amplification of specific DNA fragment for each BnaGLN1 gene was performed on mono- and polysomic additional lines selected among the descendants from a cross between Brassica napus Darmor-bzh and Brassica rapa C1.3. Each line is carrying the full A genome (mixture from B. napus and B. rapa) and one or several C chromosomes originating from Brassica napus Darmor-bzh. Specific primers designed for each gene (Supplementary Table S1) were used in order to reveal the presence or absence of specific amplicon on additional C chromosomes carried by the plant lines (indicated above each lane, Auger et al., 2009).

Supplementary Figure S3: Positions of BnaGLN1 genes on B. napus genetic map. Markers corresponding to BnaGLN1 genes mapping positions are indicated with stars. Cumulative distances between each marker are given in cM on left side of the chromosome representation. DS, SD and DY are referring to Darmor x Samouraï, Stellar x Drakkar and Darmor-bzh x Yudal double haploid mapping populations and reference maps (Lombard and Delourme, 2001; Delourme et al., 2006). Markers are coloured according to the localisation of their homologous region identified in A. thaliana (chromosome C1 to C5).

Supplementary Figure S4: Alignments of genomic sequence, deduced mRNA sequence and contigs' sequence are reported for each *BnaGLN1* gene.

Supplementary Figure S5: Expression of *BnaGLN1* genes is modified depending on nitrate availability and ageing in primary veins of vegetative *B. napus* plants.
BnaGLN1 genes relative expression level was monitored in primary veins of six leaf ranks harvested on vegetative plants grown under low (white bars) or high (black bars) nitrate conditions. Leaf ranks represented as number 1 (bottom and older leaf) to 6 (top and younger leaf) showed differential senescence symptoms. Mean and standard deviation of four plant repeats are shown.

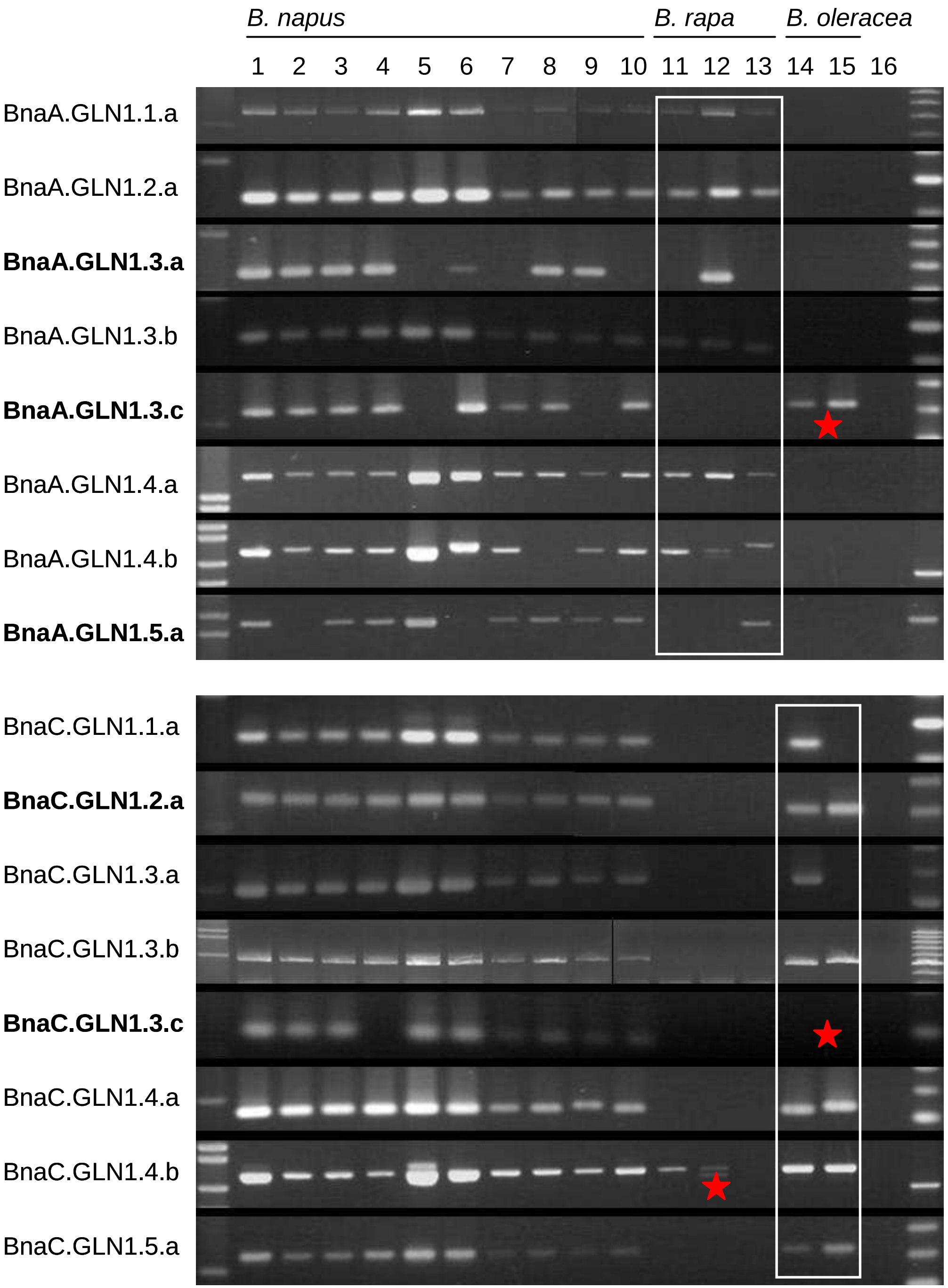

Supplementary Figure S1: *BnaGLN1* genes localisation on A or C genome using a panel of various *Brassica* genotypes:

Amplification of DNA fragments for each *BnaGLN1* gene on *Brassica napus* (1, Darmor-bzh; 2, Yudal; 3, Stellar; 4, Drakkar; 5, Samouraï; 6, Aburamasari; 7, Aviso; 8, Tenor; 9, Express; 10, Montego), *Brassica rapa* (11, Z1; 12, C1.3; 13, Chiifu) and *Brassica oleracea* (14, HDEM; 15, C102) were performed using specific primers designed for each gene (Table S1) in order to reveal a presence / absence polymorphism between *B. rapa* (A) and *B. oleracea* (C) genomes.

Water control is presented (lane 16). Genes in bold letters are also mapped on the genetic map (supplemental figure S1). Stars indicate genes for which there is no agreement with the genetic mapping performed on segregating populations or for which some results remain uncertain.

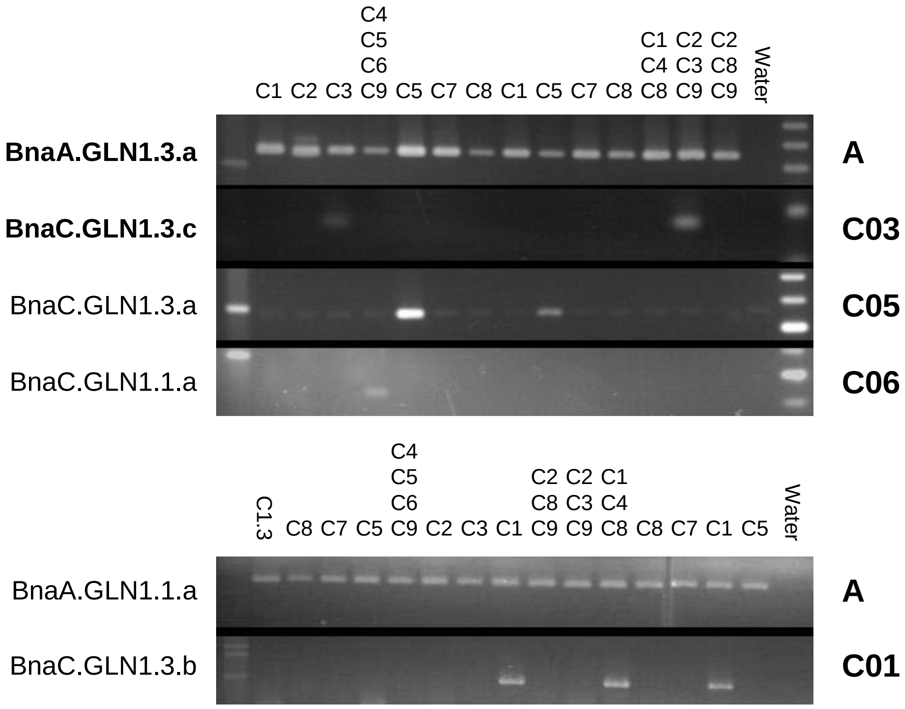

Supplementary Figure S2: *BnaGLN1* genes localisation on linkage group using mono- and polysomic additional lines.

Amplification of specific DNA fragment for each *BnaGLN1* gene was performed on mono- and polysomic additional lines selected among the descendants from a cross between *Brassica napus* Darmor-bzh and *Brassica rapa* C1.3. Each line is carrying the full A genome (mixture from *B. napus* and *B. rapa*) and one or several C chromosomes originating from *Brassica napus* Darmor-bzh. Specific primers designed for each gene (Table S1) were used in order to reveal the presence or absence of specific amplicon on additional C chromosomes carried by the plant lines (indicated above each lane, Auger et al. 2009).

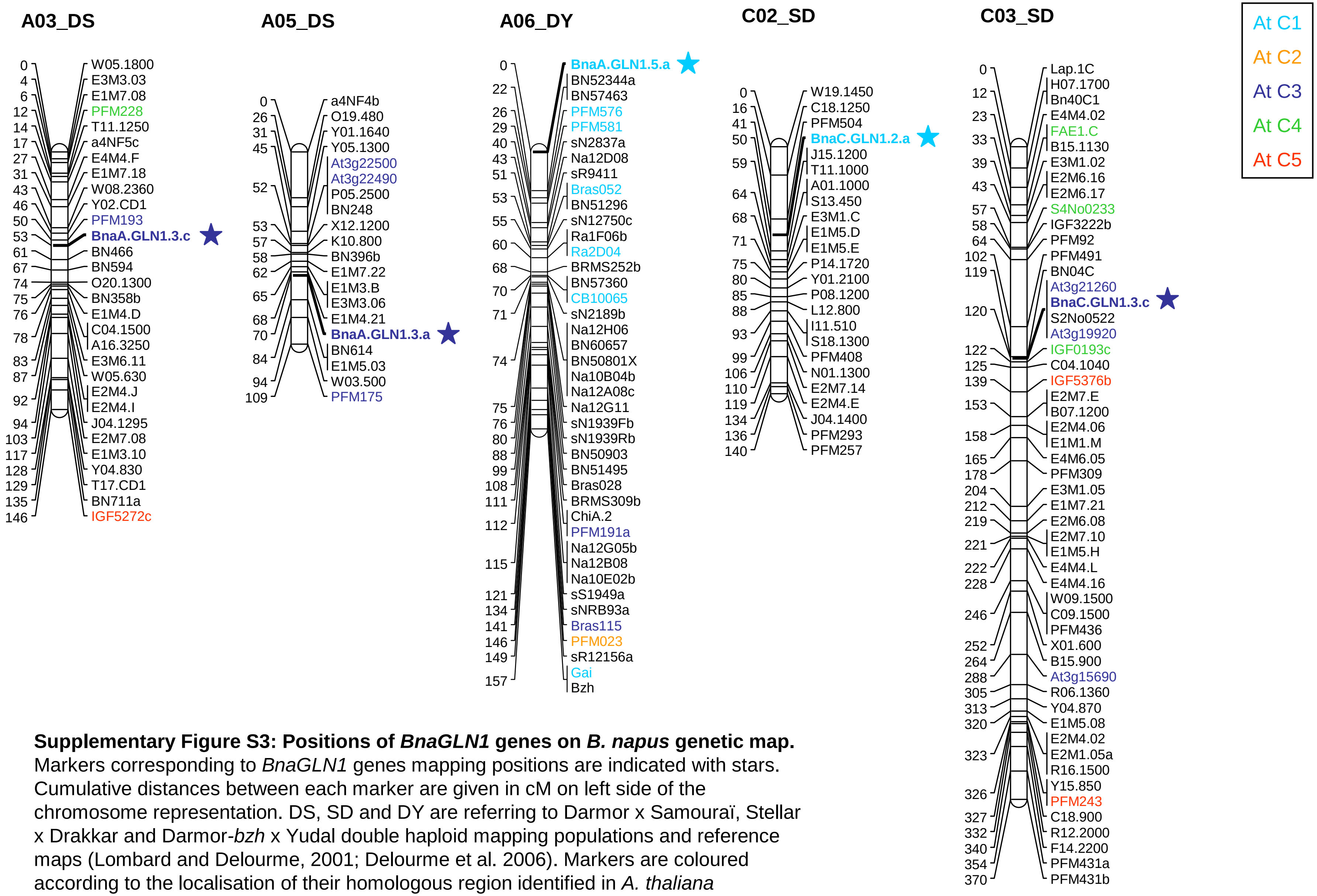

Supplementary Figure S3: Positions of *BnaGLN1* genes on *B. napus* genetic map. Markers corresponding to *BnaGLN1* genes mapping positions are indicated with stars. Cumulative distances between each marker are given in cM on left side of the chromosome representation. DS, SD and DY are referring to Darmor x Samouraï, Stellar x Drakkar and Darmor-*bzh* x Yudal double haploid mapping populations and reference maps (Lombard and Delourme, 2001; Delourme et al. 2006). Markers are coloured according to the localisation of their homologous region identified in *A. thaliana* (chromosome C1 to C5).

Supplementary Figure S4: Alignments of genomic sequence, deduced mRNA sequence and contig of ESTs sequence are reported for each *BnaGLN1* gene.

```
                 1       10        20        30        40        50        60        70        80        90        100       110       120       130
                 |---------+---------+---------+---------+---------+---------+---------+---------+---------+---------+---------+---------+---------|
   BnaA.GLN1.1.a TTATATAAAAAAACGGAGGGAGTAGTATTTAAAAGAACTGATAAATTTTTGATAAGTAGTATCTTCATAATTCCTACCGCCGCTTTTTAGAAATATTATAGGGAAATGGATTTTACCTTAAATAATGTTT
mRNA.BnaA.GLN1.1.a TTATATAAAAAAACGGAGGGAGTAGTATTTAAAAGAACTGATAAATTTTTGATAAGTAGTATCTTCATAATTCCTACCGCCGCTTTTTAGAAATATTATAGGGAAATGGATTTTACCTTAAATAATGTTT
    BnaGln1.1_C1
       Consensus ttatataaaaaaacggagggagtagtatttaaaagaactgataaatttttgataagtagtatcttcataattcctaccgccgctttttagaaatattatagggaaatggattttaccttaaataatgttt

                 131     140       150       160       170       180       190       200       210       220       230       240       250       260
                 |---------+---------+---------+---------+---------+---------+---------+---------+---------+---------+---------+---------+---------|
   BnaA.GLN1.1.a TTTTCCGATATCTCATGGAAAGTGTCACCCTGTGTGTACATGTCTGAAAAAAATAAAAAATACATAAGTATATGTTAACTTTGTAAACATTACTAAATATATAAGATTCCATTGGGCCGCAGGAGAATCT
mRNA.BnaA.GLN1.1.a TTTTCCGATATCTCATGGAAAGTGTCACCCTGTGTGTACATGTCTGAAAAAAATAAAAAATACATAAGTATATGTTAACTTTGTAAACATTACTAAATATATAAGATTCCATTGGGCCGCAGGAGAATCT
    BnaGln1.1_C1
       Consensus ttttccgatatctcatggaaagtgtcaccctgtgtgtacatgtctgaaaaaaataaaaaatacataagtatatgttaactttgtaaacattactaaatatataagattccattgggccgcaggagaatct

                 261     270       280       290       300       310       320       330       340       350       360       370       380       390
                 |---------+---------+---------+---------+---------+---------+---------+---------+---------+---------+---------+---------+---------|
   BnaA.GLN1.1.a TTTATATTTTATTTCTGATTTTTACGTTTTGTAATTTTGGTATTTTGTGGAGAGCCAGAAAGTCTTCTAACTCTTCACGTCACCAACTTTCTCGATCTCTGCACCCTCTTATTCTATAAGTACTCTTCAC
mRNA.BnaA.GLN1.1.a TTTATATTTTATTTCTGATTTTTACGTTTTGTAATTTTGGTATTTTGTGGAGAGCCAGAAAGTCTTCTAACTCTTCACGTCACCAACTTTCTCGATCTCTGCACCCTCTTATTCTATAAGTACTCTTCAC
    BnaGln1.1_C1
       Consensus tttatattttatttctgatttttacgttttgtaattttggtattttgtggagagccagaaagtcttctaactcttcacgtcaccaactttctcgatctctgcaccctcttattctataagtactcttcac

                 391     400       410       420       430       440       450       460       470       480       490       500       510       520
                 |---------+---------+---------+---------+---------+---------+---------+---------+---------+---------+---------+---------+---------|
   BnaA.GLN1.1.a ATCGACCAATATCAATACACAACCAAAACACGAGAAGATTTTATTCGATTTTATCTGTCATTTGCTTCAACTGTTACTATCGACCAATCTCGGTGTTCGTAGCCATGAGTCTTCTTACAGATCTCGTTAA
mRNA.BnaA.GLN1.1.a ATCGACCAATATCAATACACAACCAAAACACGAGAAGATTTTATTCGATTTTATCTGTCATTTGCTTCAACTGTTACTATCGACCAATCTCGGTGTTCGTAGCCATGAGTCTTCTTACAGATCTCGTTAA
    BnaGln1.1_C1                 ACACAACCAAAACACGAGAAGATTTTATTCGATTTTATCTGTCATTTGCTTCAACTGTTACTATCGACCAATCTCGGTGTTCGTAGCCATGAGTCTTCTTACAGATCTCGTTAA
       Consensus atcgaccaatatcaatACACAACCAAAACACGAGAAGATTTTATTCGATTTTATCTGTCATTTGCTTCAACTGTTACTATCGACCAATCTCGGTGTTCGTAGCCATGAGTCTTCTTACAGATCTCGTTAA

                 521     530       540       550       560       570       580       590       600       610       620       630       640       650
                 |---------+---------+---------+---------+---------+---------+---------+---------+---------+---------+---------+---------+---------|
   BnaA.GLN1.1.a CCTTAACCTCTCAGAGACCACTGACAAAATCATTGCGGAATACATATGGTTCGTCTTCTTTCTTCACAAAATCGTTGTTGTTTATGTATTAGATCGTATGCACCCTCATGTTGACCGTAAATTAAATGGG
mRNA.BnaA.GLN1.1.a CCTTAACCTCTCAGAGACCACTGACAAAATCATTGCGGAATACATATGG---------------------------------------------------------------------------------
    BnaGln1.1_C1 CCTTAACCTCTCAGAGACCACTGACAAAATCATTGCGGAATACATATGG---------------------------------------------------------------------------------
       Consensus CCTTAACCTCTCAGAGACCACTGACAAAATCATTGCGGAATACATATGG.................................................................................

                 651     660       670       680       690       700       710       720       730       740       750       760       770       780
                 |---------+---------+---------+---------+---------+---------+---------+---------+---------+---------+---------+---------+---------|
   BnaA.GLN1.1.a TTCATGTTGTTTTTCCAACTCTTAACTATTTTTGGGGTTTGTATGGAGCAGATTCTGCTAAACACATTTACAAAAAAAAAAAAGATTCTGCTAAACACACATATTTAATTTTCATTTTATTAGTCCTTCG
mRNA.BnaA.GLN1.1.a ----------------------------------------------------------------------------------------------------------------------------------
    BnaGln1.1_C1 ----------------------------------------------------------------------------------------------------------------------------------
       Consensus ..................................................................................................................................

                 781     790       800       810       820       830       840       850       860       870       880       890       900       910
                 |---------+---------+---------+---------+---------+---------+---------+---------+---------+---------+---------+---------+---------|
   BnaA.GLN1.1.a ATTTTTCTTATTAGTCCTTTAGATCCAATTTGGTTATGTTATCATGGTAGTTTTTTTGTTTAGTTTTCTGATTAAATCTACTGTTTCAAAATCTGCTGTAAAACTATGTCTGCTCTGTTTCTGGCTCTGT
mRNA.BnaA.GLN1.1.a ----------------------------------------------------------------------------------------------------------------------------------
    BnaGln1.1_C1 ----------------------------------------------------------------------------------------------------------------------------------
       Consensus ..................................................................................................................................

                 911     920       930       940       950       960       970       980       990       1000      1010      1020      1030      1040
                 |---------+---------+---------+---------+---------+---------+---------+---------+---------+---------+---------+---------+---------|
   BnaA.GLN1.1.a TCCTAAAGACTATTTAAAAAAATGTTATTGTTGTATTATTTCATATGCCAAATCAGGGTTGGAGGTTCAGGAATGGATATGAGAAGCAAAGCCAGGGTAACTTTTCTTTTCTCTAGCTATTTTTGGTAATT
mRNA.BnaA.GLN1.1.a ---------------------------------------------------------GTTGGAGGTTCAGGAATGGATATGAGAAGCAAAGCCAGG----------------------------------
    BnaGln1.1_C1 ---------------------------------------------------------GTTGGAGGTTCAGGAATGGATATGAGAAGCAAAGCCAGG----------------------------------
       Consensus .........................................................GTTGGAGGTTCAGGAATGGATATGAGAAGCAAAGCCAGG..................................

                 1041    1050      1060      1070      1080      1090      1100      1110      1120      1130      1140      1150      1160      1170
                 |---------+---------+---------+---------+---------+---------+---------+---------+---------+---------+---------+---------+---------|
   BnaA.GLN1.1.a TGTAACTTTTATCTATTTCTATTTTTGATACTCTACCAAAAGTTTGATTTATAGTAAATCTTGTTTATCTGTAGACTCTTCCTGGACCAGTGAGTGACCCTTCGGAGCTACCAAAGTGGAACTATGATGG
mRNA.BnaA.GLN1.1.a ---------------------------------------------------------------------------ACTCTTCCTGGACCAGTGAGTGACCCTTCGGAGCTACCAAAGTGGAACTATGATGG
    BnaGln1.1_C1 ---------------------------------------------------------------------------ACTCTTCCTGGACCAGTGAGTGACCCTTCGGAGCTACCAAAGTGGAACTATGATGG
       Consensus ...........................................................................ACTCTTCCTGGACCAGTGAGTGACCCTTCGGAGCTACCAAAGTGGAACTATGATGG

                 1171    1180      1190      1200      1210      1220      1230      1240      1250      1260      1270      1280      1290      1300
                 |---------+---------+---------+---------+---------+---------+---------+---------+---------+---------+---------+---------+---------|
   BnaA.GLN1.1.a CTCAAGCACAGGCCAAGCTCCTGGTGAAGACAGTGAAGTCATCTTATAGTTAATAATCGTTTCTTTCTGATCTTATATCATACCAATTTTCTGCATAAACATTTCTCTATGATTTTATTCAGATTCTTGT
mRNA.BnaA.GLN1.1.a CTCAAGCACAGGCCAAGCTCCTGGTGAAGACAGTGAAGTCATCTTATA----------------------------------------------------------------------------------
    BnaGln1.1_C1 CTCAAGCACAGGCCAAGCTCCTGGTGAAGACAGTGAAGTCATCTTATA----------------------------------------------------------------------------------
       Consensus CTCAAGCACAGGCCAAGCTCCTGGTGAAGACAGTGAAGTCATCTTATA..................................................................................

                 1301    1310      1320      1330      1340      1350      1360      1370      1380      1390      1400      1410      1420      1430
                 |---------+---------+---------+---------+---------+---------+---------+---------+---------+---------+---------+---------+---------|
   BnaA.GLN1.1.a TTATTAATCATTTTTATGTTTTGTATTATGTCTTTGTAGCCCTCAAGCCATCTTCAAAGATCCTTTCCGTAGAGGCAATAACATTCTTGTGAGTTTTGATCTTCTTGTTGTTTGCAGCCATATTTTTTTT
mRNA.BnaA.GLN1.1.a ---------------------------------------CCCTCAAGCCATCTTCAAAGATCCTTTCCGTAGAGGCAATAACATTCTTGT-----------------------------------------
    BnaGln1.1_C1 ---------------------------------------CCCTCAAGCCATCTTCAAAGATCCTTTCCGTAGAGGCAATAACATTCTTGT-----------------------------------------
       Consensus .......................................CCCTCAAGCCATCTTCAAAGATCCTTTCCGTAGAGGCAATAACATTCTTGT.........................................

                 1431    1440      1450      1460      1470      1480      1490      1500      1510      1520      1530      1540      1550      1560
                 |---------+---------+---------+---------+---------+---------+---------+---------+---------+---------+---------+---------+---------|
   BnaA.GLN1.1.a GACACGTCATAACCTTTTAATGGTTTTAATAATCAGGTCATGTGCGATGCTTACACTCCAGCGGGCGAACCAATCCCAACAAACAAAAGACACGCTGCGGCTAAGGTCTTTAGCCACCCTGATGTTGTAG
mRNA.BnaA.GLN1.1.a --------------------------------------CATGTGCGATGCTTACACTCCAGCGGGCGAACCAATCCCAACAAACAAAAGACACGCTGCGGCTAAGGTCTTTAGCCACCCTGATGTTGTAG
    BnaGln1.1_C1 --------------------------------------CATGTGCGATGCTTACACTCCAGCGGGCGAACCAATCCCAACAAACAAAAGACACGCTGCGGCTAAGGTCTTTAGCCACCCTGATGTTGTAG
       Consensus ......................................CATGTGCGATGCTTACACTCCAGCGGGCGAACCAATCCCAACAAACAAAAGACACGCTGCGGCTAAGGTCTTTAGCCACCCTGATGTTGTAG

                 1561    1570      1580      1590      1600      1610      1620      1630      1640      1650      1660      1670      1680      1690
                 |---------+---------+---------+---------+---------+---------+---------+---------+---------+---------+---------+---------+---------|
   BnaA.GLN1.1.a CTGAAGTGCCATGGTGAGTCCCTATTTAATATTGTTTTAGCCGGTTCTTGTAAAAAATGCGTAGTTATTGATGAGATCTTACTTTGAAAACCAGGTATGGTATTGAGCAAGAGTATACTTTACTTCAGAA
mRNA.BnaA.GLN1.1.a CTGAAGTGCCATGGT--------------------------------------------------------------------------------ATGGTATTGAGCAAGAGTATACTTTACTTCAGAA
    BnaGln1.1_C1 CTGAAGTGCCATGGT--------------------------------------------------------------------------------ATGGTATTGAGCAAGAGTATACTTTACTTCAGAA
       Consensus CTGAAGTGCCATGGT................................................................................ATGGTATTGAGCAAGAGTATACTTTACTTCAGAA

                 1691    1700      1710      1720      1730      1740      1750      1760      1770      1780      1790      1800      1810      1820
                 |---------+---------+---------+---------+---------+---------+---------+---------+---------+---------+---------+---------+---------|
   BnaA.GLN1.1.a AGATGTGAACTGGCCTCTTGGTTGGCCTATTGGCGGCTTCCCTGGCCCTCAGGTGAAGAAGTGATTGATAAATGGGTTTTGATTCTTTTCGATGTTCGGATTTACTAAGTTCTTAAGGTGAAATTTATCT
mRNA.BnaA.GLN1.1.a AGATGTGAACTGGCCTCTTGGTTGGCCTATTGGCGGCTTCCCTGGCCCTCAGG-----------------------------------------------------------------------------
    BnaGln1.1_C1 AGATGTGAACTGGCCTCTTGGTTGGCCTATTGGCGGCTTCCCTGGCCCTCAGG-----------------------------------------------------------------------------
       Consensus AGATGTGAACTGGCCTCTTGGTTGGCCTATTGGCGGCTTCCCTGGCCCTCAGG.............................................................................

                 1821    1830      1840      1850      1860      1870      1880      1890      1900      1910      1920      1930      1940      1950
                 |---------+---------+---------+---------+---------+---------+---------+---------+---------+---------+---------+---------+---------|
   BnaA.GLN1.1.a TTGCAGGGACCATACTATTGTAGTGTTGGAGCAGATAAATCTTTTGGTAGAGACATCGTTGATGCTCACTACAAGGCCTGCTTATACGCTGGCATCAATATTAGTGGCATCAACGGAGAAGTCATGCCTG
mRNA.BnaA.GLN1.1.a -------GACCATACTATTGTAGTGTTGGAGCAGATAAATCTTTTGGTAGAGACATCGTTGATGCTCACTACAAGGCCTGCTTATACGCTGGCATCAATATTAGTGGCATCAACGGAGAAGTCATGCCTG
    BnaGln1.1_C1 -------GACCATACTATTGTAGTGTTGGAGCAGATAAATCTTTTGGTAGAGACATCGTTGATGCTCACTACAAGGCCTGCTTATACGCTGGCATCAATATTAGTGGCATCAACGGAGAAGTCATGCCTG
```

```
      Consensus  .......GACCATACTATTGTAGTGTTGGAGCAGATAAATCTTTTGGTAGAGACATCGTTGATGCTCACTACAAGGCCTGCTTATACGCTGGCATCAATATTAGTGGCATCAACGGAGAAGTCATGCCTG

                 1951  1960      1970      1980      1990      2000      2010      2020      2030      2040      2050      2060      2070      2080
                 |--------+---------+---------+---------+---------+---------+---------+---------+---------+---------+---------+---------+---------|
  BnaA.GLN1.1.a  GTCAGGTAGTTTCCGTGACCTACAAGAAATTTTGATCTTTTACATAAAGATCTGATATTTTTCTACATTAATTTATAAATCATAAAGATCAAATCTTTTTCATAATGTTTTGATCTTCCTTTTTTCTTGT
mRNA.BnaA.GLN1.1.a  GTCAG-----------------------------------------------------------------------------------------------------------------------------
   BnaGln1.1_C1  GTCAG-----------------------------------------------------------------------------------------------------------------------------
      Consensus  GTCAG.............................................................................................................................

                 2081  2090      2100      2110      2120      2130      2140      2150      2160      2170      2180      2190      2200      2210
                 |--------+---------+---------+---------+---------+---------+---------+---------+---------+---------+---------+---------+---------|
  BnaA.GLN1.1.a  TGATAATTAATTGTGAGGTTCAATAATCAAGATCAAATATTTTGAATAAAGTACTAATTACCAAATCTTTTTTTTGTGTTGAGGGCAGTGGGAGTTCCAAGTTGGTCCAGCTGTTGGTATCTCGGCCGGT
mRNA.BnaA.GLN1.1.a  ----------------------------------------------------------------------------------------TGGGAGTTCCAAGTTGGTCCAGCTGTTGGTATCTCGGCCGGT
   BnaGln1.1_C1  ----------------------------------------------------------------------------------------TGGGAGTTCCAAGTTGGTCCAGCTGTTGGTATCTCGGCCGGT
      Consensus  ........................................................................................TGGGAGTTCCAAGTTGGTCCAGCTGTTGGTATCTCGGCCGGT

                 2211  2220      2230      2240      2250      2260      2270      2280      2290      2300      2310      2320      2330      2340
                 |--------+---------+---------+---------+---------+---------+---------+---------+---------+---------+---------+---------+---------|
  BnaA.GLN1.1.a  GATGAAATTTGGGTCGCACGTTTCATTTTGGAGGTATGTTCTAACGATTAAACATTCGAACTTTAATAACTGTTGTGTAGATGTTAATTAATAACATTCTTTGTTGGTTCTAAAAACAGAGGATCACAGA
mRNA.BnaA.GLN1.1.a  GATGAAATTTGGGTCGCACGTTTCATTTTGGAG--------------------------------------------------------------------------------------AGGATCACAGA
   BnaGln1.1_C1  GATGAAATTTGGGTCGCACGTTTCATTTTGGAG--------------------------------------------------------------------------------------AGGATCACAGA
      Consensus  GATGAAATTTGGGTCGCACGTTTCATTTTGGAG......................................................................................AGGATCACAGA

                 2341  2350      2360      2370      2380      2390      2400      2410      2420      2430      2440      2450      2460      2470
                 |--------+---------+---------+---------+---------+---------+---------+---------+---------+---------+---------+---------+---------|
  BnaA.GLN1.1.a  GATTGCTGGTGTGGTGGTATCTTTTGACCCAAAACCGATTCCCGGTGACTGGAATGGTGCTGGTGCTCACTGCAACTATAGGTACACATCTCAAGAATCTGCTATTGTAGTTGAGATCTTCTGATGCTGA
mRNA.BnaA.GLN1.1.a  GATTGCTGGTGTGGTGGTATCTTTTGACCCAAAACCGATTCCCGGTGACTGGAATGGTGCTGGTGCTCACTGCAACTATAG-------------------------------------------------
   BnaGln1.1_C1  GATTGCTGGTGTGGTGGTATCTTTTGACCCAAAACCGATTCCCGGTGACTGGAATGGTGCTGGTGCTCACTGCAACTATAG-------------------------------------------------
      Consensus  GATTGCTGGTGTGGTGGTATCTTTTGACCCAAAACCGATTCCCGGTGACTGGAATGGTGCTGGTGCTCACTGCAACTATAG.................................................

                 2471  2480      2490      2500      2510      2520      2530      2540      2550      2560      2570      2580      2590      2600
                 |--------+---------+---------+---------+---------+---------+---------+---------+---------+---------+---------+---------+---------|
  BnaA.GLN1.1.a  TTCATGTTAAACACACAAATGGTGAATTGTTTCAGTACCAAGTCAATGAGGGAAGATGGTGGTTACGAGATTATCAAGAAGGCAATTGATAAACTGGGACTGAGACACAAGGAACACATTGCTGCTTATG
mRNA.BnaA.GLN1.1.a  -----------------------------------TACCAAGTCAATGAGGGAAGATGGTGGTTACGAGATTATCAAGAAGGCAATTGATAAACTGGGACTGAGACACAAGGAACACATTGCTGCTTATG
   BnaGln1.1_C1  -----------------------------------TACCAAGTCAATGAGGGAAGATGGTGGTTACGAGATTATCAAGAAGGCAATTGATAAACTGGGACTGAGACACAAGGAACACATTGCTGCTTATG
      Consensus  ...................................TACCAAGTCAATGAGGGAAGATGGTGGTTACGAGATTATCAAGAAGGCAATTGATAAACTGGGACTGAGACACAAGGAACACATTGCTGCTTATG

                 2601  2610      2620      2630      2640      2650      2660      2670      2680      2690      2700      2710      2720      2730
                 |--------+---------+---------+---------+---------+---------+---------+---------+---------+---------+---------+---------+---------|
  BnaA.GLN1.1.a  GTGAAGGCAATGAGCGTCGTCTCACGGGTCACCACGAGACTGCTGACATCAACACTTTCCTCTGGGTAAAACTAACTAACTAACCAACACTTTGTGGGTTTCAAATAGTAAAAGGTTGCATTACATTTTA
mRNA.BnaA.GLN1.1.a  GTGAAGGCAATGAGCGTCGTCTCACGGGTCACCACGAGACTGCTGACATCAACACTTTCCTCTGGG----------------------------------------------------------------
   BnaGln1.1_C1  GTGAAGGCAATGAGCGTCGTCTCACGGGTCACCACGAGACTGCTGACATCAACACTTTCCTCTGGG----------------------------------------------------------------
      Consensus  GTGAAGGCAATGAGCGTCGTCTCACGGGTCACCACGAGACTGCTGACATCAACACTTTCCTCTGGG................................................................

                 2731  2740      2750      2760      2770      2780      2790      2800      2810      2820      2830      2840      2850      2860
                 |--------+---------+---------+---------+---------+---------+---------+---------+---------+---------+---------+---------+---------|
  BnaA.GLN1.1.a  ACTGAAGTTGTGATTGGTTTGTGTTGTGGATGTATAGGGTGTTGCGAACCGTGGAGCATCAATCCGTGTAGGACGTGACACAGAGAAAGAAGGGAAAGGATACTTTGAGGATAGGAGGCCAGCTTCGAAC
mRNA.BnaA.GLN1.1.a  --------------------------------------GTGTTGCGAACCGTGGAGCATCAATCCGTGTAGGACGTGACACAGAGAAAGAAGGGAAAGGATACTTTGAGGATAGGAGGCCAGCTTCGAAC
   BnaGln1.1_C1  --------------------------------------GTGTTGCGAACCGTGGAGCATCAATCCGTGTAGGACGTGACACAGAGAAAGAAGGGAAAGGATACTTTGAGGATAGGAGGCCAGCTTCGAAC
      Consensus  ......................................GTGTTGCGAACCGTGGAGCATCAATCCGTGTAGGACGTGACACAGAGAAAGAAGGGAAAGGATACTTTGAGGATAGGAGGCCAGCTTCGAAC

                 2861  2870      2880      2890      2900      2910      2920      2930      2940      2950      2960      2970      2980      2990
                 |--------+---------+---------+---------+---------+---------+---------+---------+---------+---------+---------+---------+---------|
  BnaA.GLN1.1.a  ATGGATCCTTACATTGTGACTTCCATGATTGCAGAGACCACAATCCTCTGGAAACCTTGATCAGATCAAAGAAGCTTGTTGAAGAATGTTCACTCCGTTTGGGTTTCTTGCATGGTTCAACGTTTGTGTG
mRNA.BnaA.GLN1.1.a  ATGGATCCTTACATTGTGACTTCCATGATTGCAGAGACCACAATCCTCTGGAAACCTTGATCAGATCAAAGAAGCTTGTTGAAGAATGTTCACTCCGTTTGGGTTTCTTGCATGGTTCAACGTTTGTGTG
   BnaGln1.1_C1  ATGGATCCTTACATTGTGACTTCCATGATTGCAGAGACCACAATCCTCTGGAAACCTTGATCAGATCAAAGAAGCTTGTTGAAGAATGTTCACTCCGTTTGGGTTTCTTGCATGGTTCAACGTTTGTGTG
      Consensus  ATGGATCCTTACATTGTGACTTCCATGATTGCAGAGACCACAATCCTCTGGAAACCTTGATCAGATCAAAGAAGCTTGTTGAAGAATGTTCACTCCGTTTGGGTTTCTTGCATGGTTCAACGTTTGTGTG

                 2991  3000      3010      3020      3030      3040      3050      3060      3070      3080      3090      3100      3110      3120
                 |--------+---------+---------+---------+---------+---------+---------+---------+---------+---------+---------+---------+---------|
  BnaA.GLN1.1.a  TTTCTCTATCAAGCATTGTCTCAGAGCAAGTCAAGAGATTTGCTTTGTTCTTATGGCTTTTATTGTTTCACATCCATTGAAAACATCTCTTTGTATCAATTTATGAATAAA
mRNA.BnaA.GLN1.1.a  TTTCTCTATCAAGCATTGTCTCAGAGCAAGTCAAGAGATTTGCTTTGTTCTTATGGCTTTTATTGTTTCACATCCATTGAAAACATCTCTTTGTATCAATTTATGAATAAA
   BnaGln1.1_C1  TTTCTCTATCAAGCATTGTCTCAGAGCAAGTCAAGAGATTTGCTTTGTTCTTATGGCTTTTATTGTTTCACATCCATTGAAAACATCTCTTTGTATCAATTTATGAATAAAGTGTGGTTTGGTTCTAAAA
      Consensus  TTTCTCTATCAAGCATTGTCTCAGAGCAAGTCAAGAGATTTGCTTTGTTCTTATGGCTTTTATTGTTTCACATCCATTGAAAACATCTCTTTGTATCAATTTATGAATAAA...................

                 3121  3130 3135
                 |--------+----|
  BnaA.GLN1.1.a
mRNA.BnaA.GLN1.1.a
   BnaGln1.1_C1  AAAAAAAAAAAAAAA
      Consensus  ...............
```

```
                   1       10        20        30        40        50        60        70        80        90        100       110       120      130
                   |--------+---------+---------+---------+---------+---------+---------+---------+---------+---------+---------+---------+---------|
      BnaC.GLN1.1.a TAATAATAGATAAAAATTAGTTTTATATATTGGTTGCGCGGGTCTTAAACTAGTAACTATTTATAGTGGTTGATATAGACCCACTTGAGCTGATCATAGATGCATAAGAGAATGCCAAGCATGATTCACA
 mRNA.BnaC.GLN1.1.a
      BnaGln1.1_C2
          Consensus ..................................................................................................................................

                   131     140       150       160       170       180       190       200       210       220       230       240       250      260
                   |--------+---------+---------+---------+---------+---------+---------+---------+---------+---------+---------+---------+---------|
      BnaC.GLN1.1.a TTAAATGAACCAACTACTGATTTGAGCGAAACATCAGTAGCAACATCTCAAGTCTTACTTTTAAGGATATATGTTCGGTAGATGGTTCTTGGACCTCTACTTCGAGCTTTAGTGAATGTGGATGAATGTA
 mRNA.BnaC.GLN1.1.a                                                                                                           CTTTAGTGAATGTGGATGAATGTA
      BnaGln1.1_C2
          Consensus ..........................................................................................................ctttagtgaatgtggatgaatgta

                   261     270       280       290       300       310       320       330       340       350       360       370       380      390
                   |--------+---------+---------+---------+---------+---------+---------+---------+---------+---------+---------+---------+---------|
      BnaC.GLN1.1.a GAAAGATAGTACTGGAAAAAAATAACTTATAGGTTTAAGAAATCAAAGACGAAGAGAATCCGCATTGCATTTGGAGTTGGAAGCGCTAAGCTGGACAACGGAAAACATGGTGTGTCATTCGACATGTCAGC
 mRNA.BnaC.GLN1.1.a GAAAGATAGTACTGGAAAAAAATAACTTATAGGTTTAAGAAATCAAAGACGAAGAGAATCCGCATTGCATTTGGAGTTGGAAGCGCTAAGCTGGACAACGGAAAACATG----------------------
      BnaGln1.1_C2
          Consensus gaaagatagtactggaaaaaaataacttataggtttaagaaatcaaagacgaagagaatccgcattgcatttggagttggaagcgctaagctggacaacggaaaacatg......................

                   391     400       410       420       430       440       450       460       470       480       490       500       510      520
                   |--------+---------+---------+---------+---------+---------+---------+---------+---------+---------+---------+---------+---------|
      BnaC.GLN1.1.a ACTTTGAGATGGACTACAAAGACTTGATAGCAATTTTAGTGGTACTTTAGCCATGGCCGATTTTTTCGACAAAATTAAAGGAGATACAAGAACTTAAGAAAAGATTTCATGATTTCAGATATGCTATATC
 mRNA.BnaC.GLN1.1.a ----------------------------------------------------------------------------------------------------------------------------------
      BnaGln1.1_C2
          Consensus ..................................................................................................................................

                   521     530       540       550       560       570       580       590       600       610       620       630       640      650
                   |--------+---------+---------+---------+---------+---------+---------+---------+---------+---------+---------+---------+---------|
      BnaC.GLN1.1.a ACTCGGGGACAAATGAGACTGCATATTTTTAGCTAAAAACACTCAATTTTTCATAACTCTTTTTTTTTTTAAAGTTTTGTTGGTTATTATATTTCGCCCTGGTTACCAAGATCACTTCAAATTTGAATAT
 mRNA.BnaC.GLN1.1.a ----------------------------------------------------------------------------------------------------------------------------------
      BnaGln1.1_C2
          Consensus ..................................................................................................................................

                   651     660       670       680       690       700       710       720       730       740       750       760       770      780
                   |--------+---------+---------+---------+---------+---------+---------+---------+---------+---------+---------+---------+---------|
      BnaC.GLN1.1.a TAATATGACATTTTTATAAGAAAACTAAAATATCGTTGAATACTTGTATTTTAGTTTTACATATCAGAATAGTTGTGATTTTGTTTCGTTGCCGAGAGTTTTCAAAATGTTATTTAGGGAAATATTATTT
 mRNA.BnaC.GLN1.1.a ----------------------------------------------------------------------------------------------------------------------------------
      BnaGln1.1_C2
          Consensus ..................................................................................................................................

                   781     790       800       810       820       830       840       850       860       870       880       890       900      910
                   |--------+---------+---------+---------+---------+---------+---------+---------+---------+---------+---------+---------+---------|
      BnaC.GLN1.1.a TACCTTTTAAAATAAATATCTAAACTATTTTACAGAGGATAAAAGAAAGCTTCAGAACCTTGGCGCGGACTCAAAACATAAAGACCGACAATTTTGCACGCGGTGCTCGGAGTCATCCGCTGGTATATAG
 mRNA.BnaC.GLN1.1.a -----------------------------------AGGATAAAAGAAAGCTTCAGAACCTTGGCGCGGACTCAAAACATAAAGACCGACAATTTTGCACGCGGTGCTCGGAGTCATCCGCTG--------
      BnaGln1.1_C2
          Consensus ...................................aggataaaagaaagcttcagaaccttggcgcggactcaaaacataaagaccgacaattttgcacgcggtgctcggagtcatccgctg........

                   911     920       930       940       950       960       970       980       990       1000      1010      1020      1030     1040
                   |--------+---------+---------+---------+---------+---------+---------+---------+---------+---------+---------+---------+---------|
      BnaC.GLN1.1.a ACACTAAGTCACATGTTTGGTAGAGTCTTAACCGAGACTGTTTATCTTGATGACAAAAAAACTATTTTTTCCATGATTTTTATACCATATTTATATGTATATATAAAACCTTCATATTATAGAGTTATAG
 mRNA.BnaC.GLN1.1.a ----------------------------------------------------------------------------------------------------------------------------------
      BnaGln1.1_C2
          Consensus ..................................................................................................................................

                   1041    1050      1060      1070      1080      1090      1100      1110      1120      1130      1140      1150      1160     1170
                   |--------+---------+---------+---------+---------+---------+---------+---------+---------+---------+---------+---------+---------|
      BnaC.GLN1.1.a ATATTTTTTTGTGTGGTTTGGAAACATTATAAAGTTATTAATTTTTTTTTTCTATTTTAACTTTAAAGCAAAATATATCAAGTAATAAATAAAAATCAATTTTTACAAATAGAAAATAAAAGGCAAAACA
 mRNA.BnaC.GLN1.1.a ----------------------------------------------------------------------------------------------------------------------------------
      BnaGln1.1_C2
          Consensus ..................................................................................................................................

                   1171    1180      1190      1200      1210      1220      1230      1240      1250      1260      1270      1280      1290     1300
                   |--------+---------+---------+---------+---------+---------+---------+---------+---------+---------+---------+---------+---------|
      BnaC.GLN1.1.a TCAATTTTTTTGTATATGAAAGGAATTTTTGTATATAAAAGAGAATTTTTGTGTATACAAAGGAGAGTGGTTGTTGTTTTTTTTCTGAGAACACACATAAAGGGGAGTAGATGTTATCCAACAAAAATAG
 mRNA.BnaC.GLN1.1.a ----------------------------------------------------------------------------------------------------------------------------------
      BnaGln1.1_C2
          Consensus ..................................................................................................................................

                   1301    1310      1320      1330      1340      1350      1360      1370      1380      1390      1400      1410      1420     1430
                   |--------+---------+---------+---------+---------+---------+---------+---------+---------+---------+---------+---------+---------|
      BnaC.GLN1.1.a ATATTTCTTCAGTTAAGGAATCAATGATTCAATTATATTCCAATGTTAACATTTATAACTCTTATTCGGACATATTACACCAAAAATCACGTAATTTTGAAAGATTACACCAGCATCATTTTCCAGTAAT
 mRNA.BnaC.GLN1.1.a ----------------------------------------------------------------------------------------------------------------------------------
      BnaGln1.1_C2
          Consensus ..................................................................................................................................

                   1431    1440      1450      1460      1470      1480      1490      1500      1510      1520      1530      1540      1550     1560
                   |--------+---------+---------+---------+---------+---------+---------+---------+---------+---------+---------+---------+---------|
      BnaC.GLN1.1.a ATTTCACCAAATTCTTTTTTATACAAATTTTTTAAATAAAATGTTTATTAGTATAAAAATTATCAAAATCTCGTAAAAATATTCATAAAAATATAGTATTTAAAAGAACTGGTAAATTTTTGATAAGTAG
 mRNA.BnaC.GLN1.1.a ----------------------------------------------------------------------------------------------------------------------------------
      BnaGln1.1_C2
          Consensus ..................................................................................................................................

                   1561    1570      1580      1590      1600      1610      1620      1630      1640      1650      1660      1670      1680     1690
                   |--------+---------+---------+---------+---------+---------+---------+---------+---------+---------+---------+---------+---------|
      BnaC.GLN1.1.a TATCTTCATAATTCCTACCGCCGCTTTTTAGAAATATTATATAGAAATGGATTTTACCTTAAATAATGTTTTTTTCCGATATCTCATGGAAAGTGTCACCCTGTGTGTACATGTCTGAAAAAACAAAAAT
 mRNA.BnaC.GLN1.1.a ----------------------------------------------------------------------------------------------------------------------------------
      BnaGln1.1_C2
          Consensus ..................................................................................................................................

                   1691    1700      1710      1720      1730      1740      1750      1760      1770      1780      1790      1800      1810     1820
                   |--------+---------+---------+---------+---------+---------+---------+---------+---------+---------+---------+---------+---------|
      BnaC.GLN1.1.a ACATAAGTATATACTAAATATATAAGATTAAACATTACTAAATAGATAAGATTCCATTGGGCCGCAGGAGAATCTTTAATATTTTATTTCCGATTTTTACGTTTTGTAATTTTGGTATTTTGTGGAGAGC
 mRNA.BnaC.GLN1.1.a ----------------------------------------------------------------------------------------------------------------------------------
      BnaGln1.1_C2
          Consensus ..................................................................................................................................

                   1821    1830      1840      1850      1860      1870      1880      1890      1900      1910      1920      1930      1940     1950
                   |--------+---------+---------+---------+---------+---------+---------+---------+---------+---------+---------+---------+---------|
      BnaC.GLN1.1.a CAGAAAGTCTTCTAACTCTTCACGTCACCAACTTTCTCGATCTCTGCACCCTCTTATTCTATAAGTACTCTTCACATCCACCAATATCAATACACAACCAAAACACGAGAAGATTTTATTCGATTTTATC
 mRNA.BnaC.GLN1.1.a ----------------------------------------------------------------------------------------------------------------------------------
      BnaGln1.1_C2                                                                                            ACACAACCAAAACACGAGAAGATTTTATTCGATTTTATC
```

```
        Consensus  ............................................................................................acacaaccaaaacacgagaagatttattcgattttatc

                   1951 1960      1970      1980      1990      2000      2010      2020      2030      2040      2050      2060      2070      2080
                   |--------+---------+---------+---------+---------+---------+---------+---------+---------+---------+---------+---------+---------|
     BnaC.GLN1.1.a TGTCATTTGCTTCAGCTGTTACTATCGACCAATCTCGGTGTTCGTAGCCATGAGTCTTCTTACAGATCTCGTTAACCTTAACCTCTCAGAGACCACTGACAAAATCATTGCGGAATACATATGGTTCGTC
mRNA.BnaC.GLN1.1.a -----------------------------------------------CCATGAGTCTTCTTACAGATCTCGTTAACCTTAACCTCTCAGAGACCACTGACAAAATCATTGCGGAATACATATGG------
    BnaGln1.1_C2   TGTCATTTGCTTCAGCTGTTACTATCGACCAATCTCGGTGTTCGTAGCCATGAGTCTTCTTACAGATCTCGTTAACCTTAACCTCTCAGAGACCACTGACAAAATCATTGCGGAATACATATGG------
        Consensus  tgtcatttgcttcagctgttactatcgaccaatctcggtgttcgtagCCATGAGTCTTCTTACAGATCTCGTTAACCTTAACCTCTCAGAGACCACTGACAAAATCATTGCGGAATACATATGG......

                   2081 2090      2100      2110      2120      2130      2140      2150      2160      2170      2180      2190      2200      2210
                   |--------+---------+---------+---------+---------+---------+---------+---------+---------+---------+---------+---------+---------|
     BnaC.GLN1.1.a TTCTTTCTTCCCAAAATCTTTGTTGTTTATGTATTAGATCGTATGCACTCTCACGTTGACCGTAAATTAAATGGGTTCATGTTGTTTTTCCAACTCTTAACTCTATTTTTGGGGTTTGTATGGAGTAGAT
mRNA.BnaC.GLN1.1.a ----------------------------------------------------------------------------------------------------------------------------------
    BnaGln1.1_C2   ----------------------------------------------------------------------------------------------------------------------------------
        Consensus  ..................................................................................................................................

                   2211 2220      2230      2240      2250      2260      2270      2280      2290      2300      2310      2320      2330      2340
                   |--------+---------+---------+---------+---------+---------+---------+---------+---------+---------+---------+---------+---------|
     BnaC.GLN1.1.a TCTGCTAAACACACATATTTAATTTTCATTTTATTAGTCCTTCGATTTTTCTTATTAGTCCTTTAGATCCAATTTGTTTATGTTATCATGGTAGTTTTTTTGTTTAGTTTTCTGATTAAATCTACTGTTT
mRNA.BnaC.GLN1.1.a ----------------------------------------------------------------------------------------------------------------------------------
    BnaGln1.1_C2   ----------------------------------------------------------------------------------------------------------------------------------
        Consensus  ..................................................................................................................................

                   2341 2350      2360      2370      2380      2390      2400      2410      2420      2430      2440      2450      2460      2470
                   |--------+---------+---------+---------+---------+---------+---------+---------+---------+---------+---------+---------+---------|
     BnaC.GLN1.1.a CAAAATCTGCTGTAAAACTATGTCTGCTCTGTTTCTGGCTCTGTTCCTAAAGAGTGTTTTTAAAAAAAAAAAATGTTATTGTTGTGTTATTTCATATGCCAAATCAGGGTTGGAGGTTCAGGAATGGATAT
mRNA.BnaC.GLN1.1.a -----------------------------------------------------------------------------------------------------------GTTGGAGGTTCAGGAATGGATAT
    BnaGln1.1_C2   -----------------------------------------------------------------------------------------------------------GTTGGAGGTTCAGGAATGGATAT
        Consensus  ...........................................................................................................GTTGGAGGTTCAGGAATGGATAT

                   2471 2480      2490      2500      2510      2520      2530      2540      2550      2560      2570      2580      2590      2600
                   |--------+---------+---------+---------+---------+---------+---------+---------+---------+---------+---------+---------+---------|
     BnaC.GLN1.1.a GAGAAGCAAAGCCAGGGTAACTTTTCTTTCTCTAGCTATTTTTGGTAATTTGTAACTTTTATCTATTTCTATTTTTGATATTACTAAAAGTTTGATTTATAGTAAATCTTGTTTATCTGTAGACTCTTCC
mRNA.BnaC.GLN1.1.a GAGAAGCAAAGCCAGG----------------------------------------------------------------------------------------------------------ACTCTTCC
    BnaGln1.1_C2   GAGAAGCAAAGCCAGG----------------------------------------------------------------------------------------------------------ACTCTTCC
        Consensus  GAGAAGCAAAGCCAGG..........................................................................................................ACTCTTCC

                   2601 2610      2620      2630      2640      2650      2660      2670      2680      2690      2700      2710      2720      2730
                   |--------+---------+---------+---------+---------+---------+---------+---------+---------+---------+---------+---------+---------|
     BnaC.GLN1.1.a TGGACCAGTGAGTGACCCTTCGGAGCTACCAAAGTGGAACTATGATGGCTCAAGCACAGGCCAAGCTCCTGGTGAAGACAGTGAAGTCATCTTATAGTTAATAATCGTTTCTTTCTGATCTTATATCATA
mRNA.BnaC.GLN1.1.a TGGACCAGTGAGTGACCCTTCGGAGCTACCAAAGTGGAACTATGATGGCTCAAGCACAGGCCAAGCTCCTGGTGAAGACAGTGAAGTCATCTTATA----------------------------------
    BnaGln1.1_C2   TGGACCAGTGAGTGACCCTTCGGAGCTACCAAAGTGGAACTATGATGGCTCAAGCACAGGCCAAGCTCCTGGTGAAGACAGTGAAGTCATCTTATA----------------------------------
        Consensus  TGGACCAGTGAGTGACCCTTCGGAGCTACCAAAGTGGAACTATGATGGCTCAAGCACAGGCCAAGCTCCTGGTGAAGACAGTGAAGTCATCTTATA..................................

                   2731 2740      2750      2760      2770      2780      2790      2800      2810      2820      2830      2840      2850      2860
                   |--------+---------+---------+---------+---------+---------+---------+---------+---------+---------+---------+---------+---------|
     BnaC.GLN1.1.a CCAATTTTCTGCATAAACATTTCTCTATGATTTTATTCAGATTCTTGTTTATTAATCATTTTTATGTTTTGTATTATGTCTTTCTAGCCCTCAAGCCATATTCAAAGATCCTTTCCGTAGAGGCAACAAC
mRNA.BnaC.GLN1.1.a ---------------------------------------------------------------------------------------CCCTCAAGCCATATTCAAAGATCCTTTCCGTAGAGGCAACAAC
    BnaGln1.1_C2   ---------------------------------------------------------------------------------------CCCTCAAGCCATATTCAAAGATCCTTTCCGTAGAGGCAACAAC
        Consensus  .......................................................................................CCCTCAAGCCATATTCAAAGATCCTTTCCGTAGAGGCAACAAC

                   2861 2870      2880      2890      2900      2910      2920      2930      2940      2950      2960      2970      2980      2990
                   |--------+---------+---------+---------+---------+---------+---------+---------+---------+---------+---------+---------+---------|
     BnaC.GLN1.1.a ATTCTTGTGAGTTTTGATCCTTCTTCTTGTTGCAGCCATATTTTTCAACATGTCATAACCTTTTAATGGTTTTAATAATCAGGTCATGTGCGATGCTTACACTCCAGCGGGCGAACCAATCCCAACAAAC
mRNA.BnaC.GLN1.1.a ATTCTTGT----------------------------------------------------------------------------CATGTGCGATGCTTACACTCCAGCGGGCGAACCAATCCCAACAAAC
    BnaGln1.1_C2   ATTCTTGT----------------------------------------------------------------------------CATGTGCGATGCTTACACTCCAGCGGGCGAACCAATCCCAACAAAC
        Consensus  ATTCTTGT............................................................................CATGTGCGATGCTTACACTCCAGCGGGCGAACCAATCCCAACAAAC

                   2991 3000      3010      3020      3030      3040      3050      3060      3070      3080      3090      3100      3110      3120
                   |--------+---------+---------+---------+---------+---------+---------+---------+---------+---------+---------+---------+---------|
     BnaC.GLN1.1.a AAAAGACACGCTGCGGCTAAGGTCTTTAGCCACCCTGATGTTGTAGCTGAAGTGCCATGGTGAGTCCCTATTTAATATCGTTTTAGCCGGTTCTTGTAAAAATGCGTAGTTATTAATGAGATCTTACTTT
mRNA.BnaC.GLN1.1.a AAAAGACACGCTGCGGCTAAGGTCTTTAGCCACCCTGATGTTGTAGCTGAAGTGCCATGGT---------------------------------------------------------------------
    BnaGln1.1_C2   AAAAGACACGCTGCGGCTAAGGTCTTTAGCCACCCTGATGTTGTAGCTGAAGTGCCATGGT---------------------------------------------------------------------
        Consensus  AAAAGACACGCTGCGGCTAAGGTCTTTAGCCACCCTGATGTTGTAGCTGAAGTGCCATGGT.....................................................................

                   3121 3130      3140      3150      3160      3170      3180      3190      3200      3210      3220      3230      3240      3250
                   |--------+---------+---------+---------+---------+---------+---------+---------+---------+---------+---------+---------+---------|
     BnaC.GLN1.1.a GAAAACCAGGTATGGTATTGAGCAAGAGTACACTTTACTTCAGAAAGATGTGAAGTGGCCTGTTGGTTGGCCTATTGGTGGCTTCCCCGGTCCTCAGGTGAAGAAGACGATTGATACATGGATTTTGAAT
mRNA.BnaC.GLN1.1.a -----------ATGGTATTGAGCAAGAGTACACTTTACTTCAGAAAGATGTGAAGTGGCCTGTTGGTTGGCCTATTGGTGGCTTCCCCGGTCCTCAGG--------------------------------
    BnaGln1.1_C2   -----------ATGGTATTGAGCAAGAGTACACTTTACTTCAGAAAGATGTGAAGTGGCCTGTTGGTTGGCCTATTGGTGGCTTCCCCGGTCCTCAGG--------------------------------
        Consensus  ...........ATGGTATTGAGCAAGAGTACACTTTACTTCAGAAAGATGTGAAGTGGCCTGTTGGTTGGCCTATTGGTGGCTTCCCCGGTCCTCAGG................................

                   3251 3260      3270      3280      3290      3300      3310      3320      3330      3340      3350      3360      3370      3380
                   |--------+---------+---------+---------+---------+---------+---------+---------+---------+---------+---------+---------+---------|
     BnaC.GLN1.1.a CTTTTCGATTTCGATGTTCGGATTTACTAAATTCTTAAGGTGAAATTTATCTTTGCAGGGACCATACTATTGTAGTGTTGGAGCAGATAAATCTTTTGGTAGAGACATTGTTGATGCTCATTACAAGGCC
mRNA.BnaC.GLN1.1.a -----------------------------------------------------------GACCATACTATTGTAGTGTTGGAGCAGATAAATCTTTTGGTAGAGACATTGTTGATGCTCATTACAAGGCC
    BnaGln1.1_C2   -----------------------------------------------------------GACCATACTATTGTAGTGTTGGAGCAGATAAATCTTTTGGTAGAGACATTGTTGATGCTCATTACAAGGCC
        Consensus  ...........................................................GACCATACTATTGTAGTGTTGGAGCAGATAAATCTTTTGGTAGAGACATTGTTGATGCTCATTACAAGGCC

                   3381 3390      3400      3410      3420      3430      3440      3450      3460      3470      3480      3490      3500      3510
                   |--------+---------+---------+---------+---------+---------+---------+---------+---------+---------+---------+---------+---------|
     BnaC.GLN1.1.a TGCTTATACGCTGGCATCACCATTAGTGGCATCAATGGAGAAGTCATGCCGGGTCAGGTAGTTTCCGTAACCTACAAGAAATTTAGATCCTTTTTTATAAATATCTGATATTTTCTATTTGACATTAATT
mRNA.BnaC.GLN1.1.a TGCTTATACGCTGGCATCACCATTAGTGGCATCAATGGAGAAGTCATGCCGGGTCAG-------------------------------------------------------------------------
    BnaGln1.1_C2   TGCTTATACGCTGGCATCACCATTAGTGGCATCAATGGAGAAGTCATGCCGGGTCAG-------------------------------------------------------------------------
        Consensus  TGCTTATACGCTGGCATCACCATTAGTGGCATCAATGGAGAAGTCATGCCGGGTCAG.........................................................................

                   3511 3520      3530      3540      3550      3560      3570      3580      3590      3600      3610      3620      3630      3640
                   |--------+---------+---------+---------+---------+---------+---------+---------+---------+---------+---------+---------+---------|
     BnaC.GLN1.1.a TATGATCATAGAGATCAAATCTTTTTTTTGATCTTTTTTTCTTTTTCTTGTTGATAATTAATTGTGATATTCAAGAATCAAAAGAACAAATCTTTTGCATAAAGTACTAATTACCAAGTCTTTTTGTGTT
mRNA.BnaC.GLN1.1.a ----------------------------------------------------------------------------------------------------------------------------------
    BnaGln1.1_C2   ----------------------------------------------------------------------------------------------------------------------------------
        Consensus  ..................................................................................................................................

                   3641 3650      3660      3670      3680      3690      3700      3710      3720      3730      3740      3750      3760      3770
                   |--------+---------+---------+---------+---------+---------+---------+---------+---------+---------+---------+---------+---------|
     BnaC.GLN1.1.a GAGGGCAGTGGGAGTTCCAAGTTGGTCCAGCTGTTGGTATCTCGGCCGGTGATGAAATTTGGGTCGCACGTTACATTTTGGAGGCAAGCCTTAACGATTAAACATTATAACTCCAATAACGTAACTGTTG
mRNA.BnaC.GLN1.1.a --------TGGGAGTTCCAAGTTGGTCCAGCTGTTGGTATCTCGGCCGGTGATGAAATTTGGGTCGCACGTTACATTTTGGAG-----------------------------------------------
    BnaGln1.1_C2   --------TGGGAGTTCCAAGTTGGTCCAGCTGTTGGTATCTCGGCCGGTGATGAAATTTGGGTCGCACGTTACATTTTGGAG-----------------------------------------------
        Consensus  ........TGGGAGTTCCAAGTTGGTCCAGCTGTTGGTATCTCGGCCGGTGATGAAATTTGGGTCGCACGTTACATTTTGGAG...............................................

                   3771 3780      3790      3800      3810      3820      3830      3840      3850      3860      3870      3880      3890      3900
                   |--------+---------+---------+---------+---------+---------+---------+---------+---------+---------+---------+---------+---------|
     BnaC.GLN1.1.a TATAGATTTTGAATAATAACATTCTTTGCTTGTTCTAAAAACAGAGGATCACAGAGGTTGCTGGTGTGGTGGTATCTTTTGACCCAAAACCTATTCCCGGTGACTGGAATGGTGCTGGTGCTCACTGCAA
mRNA.BnaC.GLN1.1.a --------------------------------------------AGGATCACAGAGGTTGCTGGTGTGGTGGTATCTTTTGACCCAAAACCTATTCCCGGTGACTGGAATGGTGCTGGTGCTCACTGCAA
```

```
[illegible]
     BnaGln1.1_C2 --------------------------------------------AGGATCACAGAGGTTGCTGGTGTGGTGGTATCTTTTGACCCAAAACCTATTCCCGGTGACTGGAATGGTGCTGGTGCTCACTGCAA
        Consensus ............................................AGGATCACAGAGGTTGCTGGTGTGGTGGTATCTTTTGACCCAAAACCTATTCCCGGTGACTGGAATGGTGCTGGTGCTCACTGCAA

                  3901  3910      3920      3930      3940      3950      3960      3970      3980      3990      4000      4010      4020      4030
                  |--------+---------+---------+---------+---------+---------+---------+---------+---------+---------+---------+---------+---------|
     BnaC.GLN1.1.a CTATAGGTACTCATAAATCTCAATAATCTGCATTTGTAGTTGATCCTTTGATGCTGATTCATGTTAAAACACAAATGGTGATTGTTTTCAGTACCAAGTCAATGAGGGAAGATGGTGGTTACGAGATTAT
mRNA.BnaC.GLN1.1.a CTATAG-------------------------------------------------------------------------------------TACCAAGTCAATGAGGGAAGATGGTGGTTACGAGATTAT
      BnaGln1.1_C2 CTATAG-------------------------------------------------------------------------------------TACCAAGTCAATGAGGGAAGATGGTGGTTACGAGATTAT
         Consensus CTATAG.....................................................................................TACCAAGTCAATGAGGGAAGATGGTGGTTACGAGATTAT

                  4031  4040      4050      4060      4070      4080      4090      4100      4110      4120      4130      4140      4150      4160
                  |--------+---------+---------+---------+---------+---------+---------+---------+---------+---------+---------+---------+---------|
     BnaC.GLN1.1.a CAAGAAGGCAATCGATAAACTGGGAATGAGACACAAGGAACACATTGCTGCTTATGGTGAAGGCAATGAGCGTCGTCTCACGGGTCATCACGAGACTGCTGACATCAACACTTTCCTCTGGGTAAACTAA
mRNA.BnaC.GLN1.1.a CAAGAAGGCAATCGATAAACTGGGAATGAGACACAAGGAACACATTGCTGCTTATGGTGAAGGCAATGAGCGTCGTCTCACGGGTCATCACGAGACTGCTGACATCAACACTTTCCTCTGGG--------
      BnaGln1.1_C2 CAAGAAGGCAATCGATAAACTGGGAATGAGACACAAGGAACACATTGCTGCTTATGGTGAAGGCAATGAGCGTCGTCTCACGGGTCATCACGAGACTGCTGACATCAACACTTTCCTCTGGG--------
         Consensus CAAGAAGGCAATCGATAAACTGGGAATGAGACACAAGGAACACATTGCTGCTTATGGTGAAGGCAATGAGCGTCGTCTCACGGGTCATCACGAGACTGCTGACATCAACACTTTCCTCTGGG........

                  4161  4170      4180      4190      4200      4210      4220      4230      4240      4250      4260      4270      4280      4290
                  |--------+---------+---------+---------+---------+---------+---------+---------+---------+---------+---------+---------+---------|
     BnaC.GLN1.1.a CTAACTAACCAACACTTTGTGGGTTTCAAATAGTTAAAGGTTGCATTACATTTTAACTGAAGTTGTGATTGGTTTGTGTTGGATGTATAGGGTGTTGCGAATCGTGGAGCATCAATCCGTGTAGGACGCG
mRNA.BnaC.GLN1.1.a -------------------------------------------------------------------------------------------GTGTTGCGAATCGTGGAGCATCAATCCGTGTAGGACGCG
      BnaGln1.1_C2 -------------------------------------------------------------------------------------------GTGTTGCGAATCGTGGAGCATCAATCCGTGTAGGACGCG
         Consensus ...........................................................................................GTGTTGCGAATCGTGGAGCATCAATCCGTGTAGGACGCG

                  4291  4300      4310      4320      4330      4340      4350      4360      4370      4380      4390      4400      4410      4420
                  |--------+---------+---------+---------+---------+---------+---------+---------+---------+---------+---------+---------+---------|
     BnaC.GLN1.1.a ACACAGAGAAAGAAGGGAAAGGATACTTTGAGGATAGGAGGCCAGCTTCGAACATGGATCCTTACATTGTGACTTCCATGATTGCAGAGACCACAATCCTCTGGAAACCTTGATCAGATCAAAGAAGATT
mRNA.BnaC.GLN1.1.a ACACAGAGAAAGAAGGGAAAGGATACTTTGAGGATAGGAGGCCAGCTTCGAACATGGATCCTTACATTGTGACTTCCATGATTGCAGAGACCACAATCCTCTGGAAACCTTGATCAGATCAAAGAAGATT
      BnaGln1.1_C2 ACACAGAGAAAGAAGGGAAAGGATACTTTGAGGATAGGAGGCCAGCTTCGAACATGGATCCTTACATTGTGACTTCCATGATTGCAGAGACCACAATCCTCTGGAAACCTTGATCAGATCAAAGAAGATT
         Consensus ACACAGAGAAAGAAGGGAAAGGATACTTTGAGGATAGGAGGCCAGCTTCGAACATGGATCCTTACATTGTGACTTCCATGATTGCAGAGACCACAATCCTCTGGAAACCTTGATCAGATCAAAGAAGATT

                  4421  4430      4440      4450      4460      4470      4480      4490      4500      4510      4520      4530      4540      4550
                  |--------+---------+---------+---------+---------+---------+---------+---------+---------+---------+---------+---------+---------|
     BnaC.GLN1.1.a GTTGAAGAATGTTCACTCCATTTGGGTTTCTTGCATGGTTCAACGTTTGTATGTTTCTCTATCAAGCATTGTCTCAGAACAAAGTCAAGAGATTTGCTCTGTTCTTATGGCTTTTATTGTTTCACATCCA
mRNA.BnaC.GLN1.1.a GTTGAAGAATGTTCACTCCATTTGGGTTTCTTGCATGGTTCAACGTTTGTATGTTTCTCTATCAAGCATTGTCTCAGAACAAAGTCAAGAGATTTGCTCTGTTCTTATGGCTTTTATTGTTTCACATCCA
      BnaGln1.1_C2 GTTGAAGAATGTTCACTCCATTTGGGTTTCTTGCATGGTTCAACGTTTGTATGTTTCTCTATCAAGCATTGTCTCAGAACAAAGTCAAGAGATTTGCTCTGTTCTTATGGCTTTTATTGTTTCACATCCA
         Consensus GTTGAAGAATGTTCACTCCATTTGGGTTTCTTGCATGGTTCAACGTTTGTATGTTTCTCTATCAAGCATTGTCTCAGAACAAAGTCAAGAGATTTGCTCTGTTCTTATGGCTTTTATTGTTTCACATCCA

                  4551  4560      4570      4580      4590      4600       4611
                  |--------+---------+---------+---------+---------+---------+|
     BnaC.GLN1.1.a TTGAAAACATCTCTTTGTATCAATTTATGAATAAA
mRNA.BnaC.GLN1.1.a TTGAAAACATCTCTTTGTATCAATTTATGAATAAA
      BnaGln1.1_C2 TTGAAAACATCTCTTTGTATCAATTTATGAATAAAGTGTGGTTTGGTTCAAAAAAAAAAAA
         Consensus TTGAAAACATCTCTTTGTATCAATTTATGAATAAA..........................
```

```
                   1       10        20        30        40        50        60        70        80        90       100       110       120       130
                   |--------+---------+---------+---------+---------+---------+---------+---------+---------+---------+---------+---------+---------|
     BnaA.GLN1.2.a TATAAATAGAAATAAAACAGTATTTTTTGGTTACTTTCATTTTTGGTGTTTTGGAGTACATTAGTTTTTTGTCCAATTTATTTTGCGTATTTTTCTTTGTAAATATATGATCTACCTTTTAATTTTTTGA
mRNA.BnaA.GLN1.2.a
      BnaGln1.2_C1
         Consensus ..................................................................................................................................

                   131     140       150       160       170       180       190       200       210       220       230       240       250       260
                   |--------+---------+---------+---------+---------+---------+---------+---------+---------+---------+---------+---------+---------|
     BnaA.GLN1.2.a ACGATGATATTACGTCTATTATATGCTAATCATATTTATCGTGTTTCATCCATTTGATATTTTTATTTTCAGCGTCTTCTGCTCTTATATTCTTACGTCTGTAATAAGCTAATTAGTAAATTATACGTCT
mRNA.BnaA.GLN1.2.a                                                                                                          AAGCTAATTAGTAAATTATACGTCT
      BnaGln1.2_C1
         Consensus .........................................................................................................aagctaattagtaaattatacgtct

                   261     270       280       290       300       310       320       330       340       350       360       370       380       390
                   |--------+---------+---------+---------+---------+---------+---------+---------+---------+---------+---------+---------+---------|
     BnaA.GLN1.2.a ATTATATTTTGATTTTCATCCATTTGATATTTTATATTTATCATTCGGACTTTTTAAGTCTGTTTTTTTCCACAAACGATACTTACAAAACATTTTTGGTCCAAAATCGGTGAAAAAACGGTTTTTTTAT
mRNA.BnaA.GLN1.2.a ATTATATTTTGATTTTCATCCATTTGATATTTTATATTTATCATTCGGACTTTTTAAGTCTGTTTTTTTCCACAAACGATACTTACAAAACATTTTTGGTCCAAAATCGGTGAAAAAACGGTTTTTTTAT
      BnaGln1.2_C1
         Consensus attatattttgattttcatccatttgatattttatatttatcattcggactttttaagtctgtttttttccacaaacgatacttacaaaacatttttggtccaaaatcggtgaaaaaacggtttttttat

                   391     400       410       420       430       440       450       460       470       480       490       500       510       520
                   |--------+---------+---------+---------+---------+---------+---------+---------+---------+---------+---------+---------+---------|
     BnaA.GLN1.2.a TTTTCCTCGCCTATTTGGCCAACTTCCTTGTAAGACAAAAGTATGAACGGTTACTTTATCATGCTAAAGTTATTATTACCCTAGAATACCTACTCAGCTACTCTTACAAGAAAATCAACGCATACATAAC
mRNA.BnaA.GLN1.2.a TTTTCCTCGCCTATTTGGCCAACTTCCTTGTAAGACAAAAGTATGAACGGTTACTTTATCATGCTAAAGTTATTATTACCCTAGAATACCTACTCAGCTACTCTTACAAGAAAATCAACGCATACATAAC
      BnaGln1.2_C1
         Consensus ttttcctcgcctatttggccaacttccttgtaagacaaaagtatgaacggttactttatcatgctaaagttattattaccctagaatacctactcagctactcttacaagaaaatcaacgcatacataac

                   521     530       540       550       560       570       580       590       600       610       620       630       640       650
                   |--------+---------+---------+---------+---------+---------+---------+---------+---------+---------+---------+---------+---------|
     BnaA.GLN1.2.a TTCTCATAAATTATCCACCTACTTGAATATCAAATGTACAATATACAGTAATCAGAGAATAAGTCAATAATGAATTATTTTGATTCTTGTAAAAAGTCAACAATCAAATTTAAGGACGACACGAAAACAA
mRNA.BnaA.GLN1.2.a TTCTCATAAATTATCCACCTACTTGAATATCAAATGTACAATATACAGTAATCAGAGAATAAGTCAATAATGAATTATTTTGATTCTTGTAAAAAGTCAACAATCAAATTTAAGGACGACACGAAAACAA
      BnaGln1.2_C1
         Consensus ttctcataaattatccacctacttgaatatcaaatgtacaatatacagtaatcagagaataagtcaataatgaattattttgattcttgtaaaaagtcaacaatcaaatttaaggacgacacgaaaacaa

                   651     660       670       680       690       700       710       720       730       740       750       760       770       780
                   |--------+---------+---------+---------+---------+---------+---------+---------+---------+---------+---------+---------+---------|
     BnaA.GLN1.2.a AAATCACCATTAAACTATATAACTTCAATTTAATACATTGGTTAGATAAAAAGAAATCGCGTTAATATATTTTATGGTTTTAATTTAAAAATAATATTTGGAAAACAGAGGCTCTGCTCTCTCTCCCTAT
mRNA.BnaA.GLN1.2.a AAATCACCATTAAACTATATAACTTCAATTTAATACATTGGTTAGATAAAAAGAAATCGCGTTAATATATTTTATGGTTTTAATTTAAAAATAATATTTGGAAAACAGAGGCTCTGCTCTCTCTCCCTAT
      BnaGln1.2_C1                                                                                                              GGGTCGACCCACGCGTCCG--
         Consensus aaatcaccattaaactatataacttcaatttaatacattggttagataaaaagaaatcgcgttaatatattttatggttttaatttaaaaataatatttggaaaacagaGGcTCtgCtCtCtCtcCCtat

                   781     790       800       810       820       830       840       850       860       870       880       890       900       910
                   |--------+---------+---------+---------+---------+---------+---------+---------+---------+---------+---------+---------+---------|
     BnaA.GLN1.2.a CTCTGTAGTATGTACCCTCGTTGCTCTCTATAAGTACTCCCACAACCACGAACTCCAAAACATCATCTCATAAACCAAAAACCACATTATCCGAGATTTGAGTATATTTCATTGCAACCTTCTTGTCATT
mRNA.BnaA.GLN1.2.a CTCTGTAGTATGTACCCTCGTTGCTCTCTATAAGTACTCCCACAACCACGAACTCCAAAACATCATCTCATAAACCAAAAACCACATTATCCGAGATTTGAGTATATTTCATTGCAACCTTCTTGTCATT
      BnaGln1.2_C1 ---------------------------------------------------------------------------CAAAAACCACATTATCCGAGATTTGAGTATATTTCATTGCAACCTTCTTGTCATT
         Consensus ctctgtagtatgtaccctcgttgctctctataagtactcccacaaccacgaactccaaaacatcatctcataaacCAAAAACCACATTATCCGAGATTTGAGTATATTTCATTGCAACCTTCTTGTCATT

                   911     920       930       940       950       960       970       980       990      1000      1010      1020      1030      1040
                   |--------+---------+---------+---------+---------+---------+---------+---------+---------+---------+---------+---------+---------|
     BnaA.GLN1.2.a TTCTCTGTAACCATGAGTCTTCTGACCGATCTCGTTAACCTTGACCTCTCAGACAACACTGAGAAAATCATCGCTGAATACATATGGTTCGTCTTCCTCCTTCGTTCTTGCCTCTTTCTTACACATTCTT
mRNA.BnaA.GLN1.2.a TTCTCTGTAACCATGAGTCTTCTGACCGATCTCGTTAACCTTGACCTCTCAGACAACACTGAGAAAATCATCGCTGAATACATATGG-------------------------------------------
      BnaGln1.2_C1 TTCTCTGTAACCATGAGTCTTCTGACCGATCTCGTTAACCTTGACCTCTCAGACAACACTGAGAAAATCATCGCTGAATACATATGG-------------------------------------------
         Consensus TTCTCTGTAACCATGAGTCTTCTGACCGATCTCGTTAACCTTGACCTCTCAGACAACACTGAGAAAATCATCGCTGAATACATATGG...........................................

                   1041   1050      1060      1070      1080      1090      1100      1110      1120      1130      1140      1150      1160      1170
                   |--------+---------+---------+---------+---------+---------+---------+---------+---------+---------+---------+---------+---------|
     BnaA.GLN1.2.a GTTTATGTTCTTGTTCTTTCCTTATCCAGATAGATAGAAGATATTGTTTTGTAATTACATATTTACATCTTCTACCACACAAAAAAAGGGTTATGTGTTTTGTCTTTTCTTATTTGTTTTTTTTTTTTTA
mRNA.BnaA.GLN1.2.a ----------------------------------------------------------------------------------------------------------------------------------
      BnaGln1.2_C1 ----------------------------------------------------------------------------------------------------------------------------------
         Consensus ..................................................................................................................................

                   1171   1180      1190      1200      1210      1220      1230      1240      1250      1260      1270      1280      1290      1300
                   |--------+---------+---------+---------+---------+---------+---------+---------+---------+---------+---------+---------+---------|
     BnaA.GLN1.2.a GAAAAAAAAACAGGGTTGGTGGTTCAGGAATGGATATGAGAAGCAAAGCCAGGGTATATGTTTTTCACTTAACTCGACGAAAAATAAAAAAAAATTGTCGGAAAATTGACCCACACGGAGAAAGATTCTG
mRNA.BnaA.GLN1.2.a --------------GTTGGTGGTTCAGGAATGGATATGAGAAGCAAAGCCAGG-----------------------------------------------------------------------------
      BnaGln1.2_C1 --------------GTTGGTGGTTCAGGAATGGATATGAGAAGCAAAGCCAGG-----------------------------------------------------------------------------
         Consensus ..............GTTGGTGGTTCAGGAATGGATATGAGAAGCAAAGCCAGG.............................................................................

                   1301   1310      1320      1330      1340      1350      1360      1370      1380      1390      1400      1410      1420      1430
                   |--------+---------+---------+---------+---------+---------+---------+---------+---------+---------+---------+---------+---------|
     BnaA.GLN1.2.a AACTTTTTTGGTATTAGAAAGATTAGGAGTTTGATTATACTGAGTTACTGATTCTCTGTTTTATTTACCTGCAGACTCTCCCTGGACCTGTGACCGATCCATCAAAGCTCCCAAAATGGAATTATGATGG
mRNA.BnaA.GLN1.2.a --------------------------------------------------------------------------ACTCTCCCTGGACCTGTGACCGATCCATCAAAGCTCCCAAAATGGAATTATGATGG
      BnaGln1.2_C1 --------------------------------------------------------------------------ACTCTCCCTGGACCTGTGACCGATCCATCAAAGCTCCCAAAATGGAATTATGATGG
         Consensus ..........................................................................ACTCTCCCTGGACCTGTGACCGATCCATCAAAGCTCCCAAAATGGAATTATGATGG

                   1431   1440      1450      1460      1470      1480      1490      1500      1510      1520      1530      1540      1550      1560
                   |--------+---------+---------+---------+---------+---------+---------+---------+---------+---------+---------+---------+---------|
     BnaA.GLN1.2.a TTCAAGCACTGGCCAAGCTCCTGGTGAAGACAGTGAAGTGATCTTATAGTAAGGCCTCTTCTCAAATTTTTATCACTATTTCAAACTCATCTTTTAATTTTATTTTTTGCTTAATAACCTTCCTTTTTAT
mRNA.BnaA.GLN1.2.a TTCAAGCACTGGCCAAGCTCCTGGTGAAGACAGTGAAGTGATCTTATA----------------------------------------------------------------------------------
      BnaGln1.2_C1 TTCAAGCACTGGCCAAGCTCCTGGTGAAGACAGTGAAGTGATCTTATA----------------------------------------------------------------------------------
         Consensus TTCAAGCACTGGCCAAGCTCCTGGTGAAGACAGTGAAGTGATCTTATA..................................................................................

                   1561   1570      1580      1590      1600      1610      1620      1630      1640      1650      1660      1670      1680      1690
                   |--------+---------+---------+---------+---------+---------+---------+---------+---------+---------+---------+---------+---------|
     BnaA.GLN1.2.a TTGTGTTCTTTAGCCCTCAAGCGATTTTCAAAGATCCGTTCCGTAGAGGCAACAACATTCTTGTGAGTTGTTAAACTTCGGTTTTCTTGCGCATGTTCTGTTTTGGCAGTTAATGATAACGTTTTTTTTA
mRNA.BnaA.GLN1.2.a -------------CCCTCAAGCGATTTTCAAAGATCCGTTCCGTAGAGGCAACAACATTCTTGT------------------------------------------------------------------
      BnaGln1.2_C1 -------------CCCTCAAGCGATTTTCAAAGATCCGTTCCGTAGAGGCAACAACATTCTTGT------------------------------------------------------------------
         Consensus .............CCCTCAAGCGATTTTCAAAGATCCGTTCCGTAGAGGCAACAACATTCTTGT..................................................................

                   1691   1700      1710      1720      1730      1740      1750      1760      1770      1780      1790      1800      1810      1820
                   |--------+---------+---------+---------+---------+---------+---------+---------+---------+---------+---------+---------+---------|
     BnaA.GLN1.2.a ATCGGTTTTGTTAGGTCATGTGTGATACTTACACCCCTGCGGGTGAACCAATCCCTACGAACAAGAGACATGCTGCAGCTCAGATCTTTAGCAACCCTGATGTTGTTGCTGAAGTGCCATGGTTAACCCA
mRNA.BnaA.GLN1.2.a ----------------CATGTGTGATACTTACACCCCTGCGGGTGAACCAATCCCTACGAACAAGAGACATGCTGCAGCTCAGATCTTTAGCAACCCTGATGTTGTTGCTGAAGTGCCATGGT-------
      BnaGln1.2_C1 ----------------CATGTGTGATACTTACACCCCTGCGGGTGAACCAATCCCTACGAACAAGAGACATGCTGCAGCTCAGATCTTTAGCAACCCTGATGTTGTTGCTGAAGTGCCATGGT-------
         Consensus ................CATGTGTGATACTTACACCCCTGCGGGTGAACCAATCCCTACGAACAAGAGACATGCTGCAGCTCAGATCTTTAGCAACCCTGATGTTGTTGCTGAAGTGCCATGGT.......

                   1821   1830      1840      1850      1860      1870      1880      1890      1900      1910      1920      1930      1940      1950
                   |--------+---------+---------+---------+---------+---------+---------+---------+---------+---------+---------+---------+---------|
     BnaA.GLN1.2.a AATTCCCCTGTTCCCGTTTTCGTTATATATTTTTTTGGTTTCTTGCGGTCTGATTTAGCTCTGCATACTAGGTATGGAATCGAACAAGAATACACTCTGTTGCAGAAAGATGTGAATTGGCCTGTCGGAT
mRNA.BnaA.GLN1.2.a -------------------------------------------------------------------------ATGGAATCGAACAAGAATACACTCTGTTGCAGAAAGATGTGAATTGGCCTGTCGGAT
      BnaGln1.2_C1 -------------------------------------------------------------------------ATGGAATCGAACAAGAATACACTCTGTTGCAGAAAGATGTGAATTGGCCTGTCGGAT
```

```
         Consensus  .........................................................................ATGGAATCGAACAAGAATACACTCTGTTGCAGAAAGATGTGAATTGGCCTGTCGGAT

                    1951 1960      1970      1980      1990      2000      2010      2020      2030      2040      2050      2060      2070      2080
                    |--------+---------+---------+---------+---------+---------+---------+---------+---------+---------+---------+---------+---------|
      BnaA.GLN1.2.a GGCCCATTGGTGGATTCCCCGGCCCTCAGGGACCATACTACTGCAGTGTTGGAGCTGACAAATCTTTTGGAAGAGACATTGTTGATGCTCACTACAAGGCTTGTTTGTATGCTGGAATTAACATCAGTGG
 mRNA.BnaA.GLN1.2.a GGCCCATTGGTGGATTCCCCGGCCCTCAGGGACCATACTACTGCAGTGTTGGAGCTGACAAATCTTTTGGAAGAGACATTGTTGATGCTCACTACAAGGCTTGTTTGTATGCTGGAATTAACATCAGTGG
      BnaGln1.2_C1 GGCCCATTGGTGGATTCCCCGGCCCTCAGGGACCATACTACTGCAGTGTTGGAGCTGACAAATCTTTTGGAAGAGACATTGTTGATGCTCACTACAAGGCTTGTTTGTATGCTGGAATTAACATCAGTGG
         Consensus  GGCCCATTGGTGGATTCCCCGGCCCTCAGGGACCATACTACTGCAGTGTTGGAGCTGACAAATCTTTTGGAAGAGACATTGTTGATGCTCACTACAAGGCTTGTTTGTATGCTGGAATTAACATCAGTGG

                    2081 2090      2100      2110      2120      2130      2140      2150      2160      2170      2180      2190      2200      2210
                    |--------+---------+---------+---------+---------+---------+---------+---------+---------+---------+---------+---------+---------|
      BnaA.GLN1.2.a AATCAATGGAGAAGTCATGCCTGGTCAGGTATAACTTCAAGATCCAACAGAATTAAATCTTTTTTGCATAATGTAGTAATGACCAAATAATTTTTTTTTTTGTTGAGACAGTGGGAGTTCCAAGTCGGAC
 mRNA.BnaA.GLN1.2.a AATCAATGGAGAAGTCATGCCTGGTCAG-----------------------------------------------------------------------------------TGGGAGTTCCAAGTCGGAC
      BnaGln1.2_C1 AATCAATGGAGAAGTCATGCCTGGTCAG-----------------------------------------------------------------------------------TGGGAGTTCCAAGTCGGAC
         Consensus  AATCAATGGAGAAGTCATGCCTGGTCAG...................................................................................TGGGAGTTCCAAGTCGGAC

                    2211 2220      2230      2240      2250      2260      2270      2280      2290      2300      2310      2320      2330      2340
                    |--------+---------+---------+---------+---------+---------+---------+---------+---------+---------+---------+---------+---------|
      BnaA.GLN1.2.a CGTCGGTTGGTATCTCAGCTGCTGATGAAGTGTGGATTGCTCGTTTTATTTTGGAGGTATCATCCACTCTCAATTGTTGTTGCTCTCTTAATGGAATATAAAATTGAGTACAATTTTTTGGTGGTTTTAA
 mRNA.BnaA.GLN1.2.a CGTCGGTTGGTATCTCAGCTGCTGATGAAGTGTGGATTGCTCGTTTTATTTTGGAG--------------------------------------------------------------------------
      BnaGln1.2_C1 CGTCGGTTGGTATCTCAGCTGCTGATGAAGTGTGGATTGCTCGTTTTATTTTGGAG--------------------------------------------------------------------------
         Consensus  CGTCGGTTGGTATCTCAGCTGCTGATGAAGTGTGGATTGCTCGTTTTATTTTGGAG..........................................................................

                    2341 2350      2360      2370      2380      2390      2400      2410      2420      2430      2440      2450      2460      2470
                    |--------+---------+---------+---------+---------+---------+---------+---------+---------+---------+---------+---------+---------|
      BnaA.GLN1.2.a AAAACAGAGGATCACAGAGATTGCTGGTGTGGTTGTATCTTTTGACCCAAAACCAATTCCGGGTGACTGGAACGGAGCTGGTGCTCACACCAATTACAGGTAAAAGGATCATGCAACTTAAGCTTGTTAT
 mRNA.BnaA.GLN1.2.a -------AGGATCACAGAGATTGCTGGTGTGGTTGTATCTTTTGACCCAAAACCAATTCCGGGTGACTGGAACGGAGCTGGTGCTCACACCAATTACAG-------------------------------
      BnaGln1.2_C1 -------AGGATCACAGAGATTGCTGGTGTGGTTGTATCTTTTGACCCAAAACCAATTCCGGGTGACTGGAACGGAGCTGGTGCTCACACCAATTACAG-------------------------------
         Consensus  .......AGGATCACAGAGATTGCTGGTGTGGTTGTATCTTTTGACCCAAAACCAATTCCGGGTGACTGGAACGGAGCTGGTGCTCACACCAATTACAG...............................

                    2471 2480      2490      2500      2510      2520      2530      2540      2550      2560      2570      2580      2590      2600
                    |--------+---------+---------+---------+---------+---------+---------+---------+---------+---------+---------+---------+---------|
      BnaA.GLN1.2.a ATAATTTGTTCTTTTGGAAAAAAAAAATCTGAAATATGGTAAATAATTTTTTCAGTACTAAATCGATGAGGGAGGAAGGAGGATACGAGATAATCAAGAAGGCAATTGATAAGCTCGGACTGAGACACAA
 mRNA.BnaA.GLN1.2.a -------------------------------------------------------TACTAAATCGATGAGGGAGGAAGGAGGATACGAGATAATCAAGAAGGCAATTGATAAGCTCGGACTGAGACACAA
      BnaGln1.2_C1 -------------------------------------------------------TACTAAATCGATGAGGGAGGAAGGAGGATACGAGATAATCAAGAAGGCAATTGATAAGCTCGGACTGAGACACAA
         Consensus  .......................................................TACTAAATCGATGAGGGAGGAAGGAGGATACGAGATAATCAAGAAGGCAATTGATAAGCTCGGACTGAGACACAA

                    2601 2610      2620      2630      2640      2650      2660      2670      2680      2690      2700      2710      2720      2730
                    |--------+---------+---------+---------+---------+---------+---------+---------+---------+---------+---------+---------+---------|
      BnaA.GLN1.2.a GGAGCACATTTCTGCTTACGGTGAAGGCAACGAGCGTCGTCTCACTGGACACCATGAAACTGCTGATATCAACACTTTCAAATGGGTAAACATTTAAATTTTTTAAAGAGTTGATAACATTTTTGTGTAC
 mRNA.BnaA.GLN1.2.a GGAGCACATTTCTGCTTACGGTGAAGGCAACGAGCGTCGTCTCACTGGACACCATGAAACTGCTGATATCAACACTTTCAAATGGG--------------------------------------------
      BnaGln1.2_C1 GGAGCACATTTCTGCTTACGGTGAAGGCAACGAGCGTCGTCTCACTGGACACCATGAAACTGCTGATATCAACACTTTCAAATGGG--------------------------------------------
         Consensus  GGAGCACATTTCTGCTTACGGTGAAGGCAACGAGCGTCGTCTCACTGGACACCATGAAACTGCTGATATCAACACTTTCAAATGGG............................................

                    2731 2740      2750      2760      2770      2780      2790      2800      2810      2820      2830      2840      2850      2860
                    |--------+---------+---------+---------+---------+---------+---------+---------+---------+---------+---------+---------+---------|
      BnaA.GLN1.2.a TGAACTTTGTGTTTTTTTTTTTTTTGAATCTTTGTTAAAAGGGTGTTGCAAACCGTGGAGCATCAATCCGTGTAGGACGTGACACGGAGAAGGAAGGGAAAGGATACTTTGAGGATAGGAGGCCAGCTTC
 mRNA.BnaA.GLN1.2.a ------------------------------------------GTGTTGCAAACCGTGGAGCATCAATCCGTGTAGGACGTGACACGGAGAAGGAAGGGAAAGGATACTTTGAGGATAGGAGGCCAGCTTC
      BnaGln1.2_C1 ------------------------------------------GTGTTGCAAACCGTGGAGCATCAATCCGTGTAGGACGTGACACGGAGAAGGAAGGGAAAGGATACTTTGAGGATAGGAGGCCAGCTTC
         Consensus  ..........................................GTGTTGCAAACCGTGGAGCATCAATCCGTGTAGGACGTGACACGGAGAAGGAAGGGAAAGGATACTTTGAGGATAGGAGGCCAGCTTC

                    2861 2870      2880      2890      2900      2910      2920      2930      2940      2950      2960      2970      2980      2990
                    |--------+---------+---------+---------+---------+---------+---------+---------+---------+---------+---------+---------+---------|
      BnaA.GLN1.2.a CAACATGGACCCTTACACTGTAACTTCCATGATTGCAGAGACTACACTTCTTTGGAATCCTTGAAGAAATATGACAATAATATAACTTGAATCTGGTTTCAAGTTTGTGTTTCTACAGTTTATTAAGCAA
 mRNA.BnaA.GLN1.2.a CAACATGGACCCTTACACTGTAACTTCCATGATTGCAGAGACTACACTTCTTTGGAATCCTTGAAGAAATATGACAATAATATAACTTGAATCTGGTTTCAAGTTTGTGTTTCTACAGTTTATTAAGCAA
      BnaGln1.2_C1 CAACATGGACCCTTACACTGTAACTTCCATGATTGCAGAGACTACACTTCTTTGGAATCCTTGAAGAAATATGACAATAATATAACTTGAATCTGGTTTCAAGTTTGTGTTTCTACAGTTTATTAAGCAA
         Consensus  CAACATGGACCCTTACACTGTAACTTCCATGATTGCAGAGACTACACTTCTTTGGAATCCTTGAAGAAATATGACAATAATATAACTTGAATCTGGTTTCAAGTTTGTGTTTCTACAGTTTATTAAGCAA

                    2991 3000      3010      3020      3030      3040      3050      3060      3070      3080      3090      3100      3110      3120
                    |--------+---------+---------+---------+---------+---------+---------+---------+---------+---------+---------+---------+---------|
      BnaA.GLN1.2.a TTACCGGGTTGATACTGCCGGAGTTTGTGGTTTGAGGCCTTTCTTTTAATCTCTTTGTGTTTTGGGGTTTGTGATTGGAGCAAAAGCCCTGATTTGCTCTGTTTCTTTGACCTTTTATTTGAACCCTTTG
 mRNA.BnaA.GLN1.2.a TTACCGGGTTGATACTGCCGGAGTTTGTGGTTTGAGGCCTTTCTTTTAATCTCTTTGTGTTTTGGGGTTTGTGATTGGAGCAAAAGCCCTGATTTGCTCTGTTTCTTTGACCTTTTATTTGAACCCTTTG
      BnaGln1.2_C1 TTACCGGGTTGATACTGCCGGAGTTTGTGGTTTGAGGCCTTTCTTTTAATCTCTTTGTGTTTTGGGGTTTGTGATTGGAGCAAAAGCCCTGATTTGCTCTGTTTCTTTGACCTTTTATTTGAACCCTTTG
         Consensus  TTACCGGGTTGATACTGCCGGAGTTTGTGGTTTGAGGCCTTTCTTTTAATCTCTTTGTGTTTTGGGGTTTGTGATTGGAGCAAAAGCCCTGATTTGCTCTGTTTCTTTGACCTTTTATTTGAACCCTTTG

                    3121 3130      3140      3150      3160      3170      3180      3190      3200      3210      3220      3230      3240      3250
                    |--------+---------+---------+---------+---------+---------+---------+---------+---------+---------+---------+---------+---------|
      BnaA.GLN1.2.a TATTTGTATTAATAAGACGATCTGAAAAGGCCTTTTCATGTTTCTATCTGAGACTGCTCAACAAGTTCACAAATAACAAAACTCTTTGGTAGGCCGAGAACCTGTAAGCTAGACGGGTCCATCTATAAAT
 mRNA.BnaA.GLN1.2.a TATTTGTATTAATAAGACGATCTGAAAAGGCCTTTTCATGTTTCTATCTGAGACTGCTCAACAAGTTCACAAATAACAAAACTCTTTGGTAGGCCGAGAACCTGTAAGCTAGACGGGTCCATCTATAAAT
      BnaGln1.2_C1 TATTTGTATTAATAAGACGATCTGAAAAGGCCTTTTCATGTTTCAAA-----------------------------------------------------------------------------------
         Consensus  TATTTGTATTAATAAGACGATCTGAAAAGGCCTTTTCATGTTTCtAtctgagactgctcaacaagttcacaaataacaaaactctttggtaggccgagaacctgtaagctagacgggtccatctataaat

                    3251 3260      3270      3280      3290      3300      3310      3320      3330      3340      3350      3360      3370      3380
                    |--------+---------+---------+---------+---------+---------+---------+---------+---------+---------+---------+---------+---------|
      BnaA.GLN1.2.a ATATTGGTACACACATAAATGTATTCTAGGCACCGTATAAAATACAATCATAGATCATATTCAATCCGGAGAAACATGTCTTTCTCGAACAATAGATGGAACCTAACTAATTAGAAATTGGGCATTTGAA
 mRNA.BnaA.GLN1.2.a ATATTGGTACACACATAAATGTATTCTAGGCACCGTATAAAATACAATCATAGATCATATTCAATCCGGAGAAACATGTCTTTCTCGAACAATAGATGGAACCTAACTAATTAGAAATTGGGCATTTGAA
      BnaGln1.2_C1 ----------------------------------------------------------------------------------------------------------------------------------
         Consensus  atattggtacacacataaatgtattctaggcaccgtataaaatacaatcatagatcatattcaatccggagaaacatgtctttctcgaacaatagatggaacctaactaattagaaattgggcatttgaa

                    3381 3390      3400      3410      3420      3430      3440      3450      3460      3470      3480      3490      3500      3510
                    |--------+---------+---------+---------+---------+---------+---------+---------+---------+---------+---------+---------+---------|
      BnaA.GLN1.2.a ATGTTTGTAATGCGAGTATCAAGTCCTCAATCAAACTTGAATTGGCATGATGTTGCTGGTTGCACCTCCAAGAAAATGACAACTCGATCACCTTATCCTTTGGATCATGCGATGAGAGTCCCAAGATTGT
 mRNA.BnaA.GLN1.2.a ATGTTTGTAATGCGAGTATCAAGTCCTCAATCAAACTTGAATTGGCATGATGTTGCTGGTTGCACCTCCAAGAAAATGACAACTCGATCACCTTATCCTTTGGATCATGCGATGAGAGTCCCAAGATTGT
      BnaGln1.2_C1 ----------------------------------------------------------------------------------------------------------------------------------
         Consensus  atgtttgtaatgcgagtatcaagtcctcaatcaaacttgaattggcatgatgttgctggttgcacctccaagaaaatgacaactcgatcaccttatcctttggatcatgcgatgagagtcccaagattgt

                    3511 3520      3530      3540      3550      3560      3570      3580      3590      3600      3610      3620      3630      3640
                    |--------+---------+---------+---------+---------+---------+---------+---------+---------+---------+---------+---------+---------|
      BnaA.GLN1.2.a GCAGTCTTAAGCAACAAGAATTTCTCGAGATCAATCTTGTTCTGTGATCCATCGAACGTTGTCCACTTGAGACTTTTTATTTGTTTATGTCAGTTTGCCTCCATGATTAGGAACCATACACAACCGGTA
 mRNA.BnaA.GLN1.2.a GCAGTCTT
      BnaGln1.2_C1 ----------------------------------------------------------------------------------------------------------------------------------
         Consensus  gcagtctt..........................................................................................................................

                    3641 3650      3660      3670      3680      3690      3700      3710      3720      3730      3740      3750      3760      3770
                    |--------+---------+---------+---------+---------+---------+---------+---------+---------+---------+---------+---------+---------|
      BnaA.GLN1.2.a TTTATGAAAAGTTATTTTCATAACTCCTGATTTTTGGTTTGAACAAATGTGTTTCTTCGTTGAAAATATATGTAAAATTTTCATATTATTTCTTGGTTGTTATATATATGAGACAATGCAAAGAAGAGAT
 mRNA.BnaA.GLN1.2.a
      BnaGln1.2_C1 -----------------------------------------------------------------------------------------------------------------------AAAAAAAAAAA
         Consensus  .......................................................................................................................aaa.aa.a.a.

                    3771 3780      3790      3800      3810      3820      3830      3840      3850      3860      3870      3880      3890      3900
                    |--------+---------+---------+---------+---------+---------+---------+---------+---------+---------+---------+---------+---------|
      BnaA.GLN1.2.a AATCAAAACAAAAACAACAGTTAAGGACTGTTGTTAGAATTCTATAGAAAAATTCAAGAGATAGTATTGAGATGAAGACTTGAAGAGAAGTTAACATGATCTCTGCATGTTTAACATTTATTCTCCTAGGA
 mRNA.BnaA.GLN1.2.a
```

```
[illegible]
      BnaGln1.2_C1  AAAAAAAAAAAAAAAAAAA
         Consensus  aa..aaaa.aaaaa.aa.a...............................................................................................................

                    3901  3910      3920      3930      3940      3950      3960      3970      3980      3990      4000      4010      4020      4030
                    |--------+---------+---------+---------+---------+---------+---------+---------+---------+---------+---------+---------+---------|
       BnaA.GLN1.2.a  AATGAAGATCTCTTGTATGTTCATCATTTAAATTATTCATGCAAGAGCATGCAAGTGTTGTCGCATTTATTGATAAATCGTGTATTGTCTATATAAAGGTTGTGACTTCTCTCTTGTAATATATAGAAAT
  mRNA.BnaA.GLN1.2.a
      BnaGln1.2_C1
         Consensus  ..................................................................................................................................

                    4031  4040     4049
                    |--------+--------|
       BnaA.GLN1.2.a  ACACTCAATATAAATAAAA
  mRNA.BnaA.GLN1.2.a
      BnaGln1.2_C1
         Consensus  ...................
```

```
                   1       10        20        30        40        50        60        70        80        90        100       110       120      130
                   |--------+---------+---------+---------+---------+---------+---------+---------+---------+---------+---------+---------+---------|
     BnaC.GLN1.2.a TATATAAACCGAACCGAAGTAAATATAGATTTAGAATGATACTTATATTTTACTAACTGAAATACCGAAAACCCAAAAAAAACGAACCCAAACCGAAACGATATCCGGATTGAACACCGCTATTAAATTA
mRNA.BnaC.GLN1.2.a
      BnaGln1.2_C2
         Consensus ..................................................................................................................................

                   131     140       150       160       170       180       190       200       210       220       230       240       250      260
                   |--------+---------+---------+---------+---------+---------+---------+---------+---------+---------+---------+---------+---------|
     BnaC.GLN1.2.a ATGTATTTTAATTTCATTTATTTATTTTCTATTTTATTTAATCATTTGAATTTTATTTAGTTTTCTGATAGCTTTACAAAGAATATATGGCTCATTTCGTATAACTAGATTTTCCACTAATCATTTTCAA
mRNA.BnaC.GLN1.2.a
      BnaGln1.2_C2
         Consensus ..................................................................................................................................

                   261     270       280       290       300       310       320       330       340       350       360       370       380      390
                   |--------+---------+---------+---------+---------+---------+---------+---------+---------+---------+---------+---------+---------|
     BnaC.GLN1.2.a TAATATAATATTAATTTAACAAAAAAAAAATTATAATGTAAGATGATGAAAATGAAGAATATTAATTTATATGAAAAATGATGACGTCAAAGCTTTTATTGTTGGTGTTTTCAAAGGGGAAGTCTCCTCC
mRNA.BnaC.GLN1.2.a
      BnaGln1.2_C2                                                                                                                   CGGGGCGG--------
         Consensus ...................................................................................................................gggg..g........

                   391     400       410       420       430       440       450       460       470       480       490       500       510      520
                   |--------+---------+---------+---------+---------+---------+---------+---------+---------+---------+---------+---------+---------|
     BnaC.GLN1.2.a AATGCATTTTCCAGTCAGCAAATTATGCGCTTGTATTGTATTTTTTATAGAGAAATTGCGCTCTCTACACAGCACTTTTATCATAATTAGCCTCATACCCTAAATCCCACAATCTTCTACCTTATTCCAT
mRNA.BnaC.GLN1.2.a
      BnaGln1.2_C2 ----------------------------------------------------------------------------------------------------------------------------------
         Consensus ..................................................................................................................................

                   521     530       540       550       560       570       580       590       600       610       620       630       640      650
                   |--------+---------+---------+---------+---------+---------+---------+---------+---------+---------+---------+---------+---------|
     BnaC.GLN1.2.a AGATACAAATTAGTCAAATCACAGGTATTCTCTTTTTTTTGGGTTTTCAACCGATTGCTTCTTTTTTATAAGTAAAAATAAAACAGTATCAAATTCATTTTTTTTTTGTGTGTTTTGGAGTACATTAGTT
mRNA.BnaC.GLN1.2.a
      BnaGln1.2_C2 ----------------------------------------------------------------------------------------------------------------------------------
         Consensus ..................................................................................................................................

                   651     660       670       680       690       700       710       720       730       740       750       760       770      780
                   |--------+---------+---------+---------+---------+---------+---------+---------+---------+---------+---------+---------+---------|
     BnaC.GLN1.2.a TTGCGTATATTTCTTTGTAAATATATGATCTACCTTATTATTTTTTGAACTATGGGATTACCTCTATTATATGCTAATCATATTTATCCTGTTTCATTCATTTGATATTTTTATTTTCACCGTCTTCTGC
mRNA.BnaC.GLN1.2.a
      BnaGln1.2_C2 ----------------------------------------------------------------------------------------------------------------------------------
         Consensus ..................................................................................................................................

                   781     790       800       810       820       830       840       850       860       870       880       890       900      910
                   |--------+---------+---------+---------+---------+---------+---------+---------+---------+---------+---------+---------+---------|
     BnaC.GLN1.2.a TCTTATATTTTTACGTCTATAATAAGTTTTGCGTATATTTCTTTGTAAATATATGATCTACCTTATTATTTTTTGAACTATGAGATTACCTCTATTATATGCTAATCATATTTATCCTGTTTCATTCATT
mRNA.BnaC.GLN1.2.a
      BnaGln1.2_C2 ----------------------------------------------------------------------------------------------------------------------------------
         Consensus ..................................................................................................................................

                   911     920       930       940       950       960       970       980       990       1000      1010      1020      1030    1040
                   |--------+---------+---------+---------+---------+---------+---------+---------+---------+---------+---------+---------+---------|
     BnaC.GLN1.2.a TGATATTTTTATTTTCACCGTCTTCTGCTCTTATATTTTTACTTCTATAATAAGTTAATTATACGTCTATTATACTTTGATTTTCATCCATTTGATATTTTATATTTATCATTCGGACTTTTGTAAGTCT
mRNA.BnaC.GLN1.2.a                                                        TAATTATACGTCTATTATACTTTGATTTTCATCCATTTGATATTTTATATTTATCATTCGGACTTTTGTAAGTCT
      BnaGln1.2_C2 ----------------------------------------------------------------------------------------------------------------------------------
         Consensus .......................................................taattatacgtctattatactttgattttcatccatttgatattttatatttatcattcggacttttgtaagtct

                   1041 1050     1060      1070      1080      1090      1100      1110      1120      1130      1140      1150      1160    1170
                   |--------+---------+---------+---------+---------+---------+---------+---------+---------+---------+---------+---------+---------|
     BnaC.GLN1.2.a AATTTTTTCCACAAACGCTAATTATTTACAAAACATTTTTGGTCAAAAATCGATGAAAAAAAAATGGTTTCTATTTTTCCTCGCCTATTAGGCCAACCTCGTAGAAAAAGTATGAACGGTTACTTTATAA
mRNA.BnaC.GLN1.2.a AATTTTTTCCACAAACGCTAATTATTTACAAAACATTTTTGGTCAAAAATCGATGAAAAAAAAATGGTTTCTATTTTTCCTCGCCTATTAGGCCAACCTCGTAGAAAAAGTATGAACGGTTACTTTATAA
      BnaGln1.2_C2 ----------------------------------------------------------------------------------------------------------------------------------
         Consensus aatttttttccacaaacgctaattatttacaaaacatttttggtcaaaaatcgatgaaaaaaaaatggtttctatttttcctcgcctattaggccaacctcgtagaaaaagtatgaacggttactttataa

                   1171 1180     1190      1200      1210      1220      1230      1240      1250      1260      1270      1280      1290    1300
                   |--------+---------+---------+---------+---------+---------+---------+---------+---------+---------+---------+---------+---------|
     BnaC.GLN1.2.a TGCTAAAGTTATTATTACCCTAGAATACCTACTCAGCTACTCTTACAAGAAAATCAACGCATACATAACTTCTCATAAATTATCCACCTACTTGAATATCAAATGTACAATATACAGTAATTTGGGAATA
mRNA.BnaC.GLN1.2.a TGCTAAAGTTATTATTACCCTAGAATACCTACTCAGCTACTCTTACAAGAAAATCAACGCATACATAACTTCTCATAAATTATCCACCTACTTGAATATCAAATGTACAATATACAGTAATTTGGGAATA
      BnaGln1.2_C2 ----------------------------------------------------------------------------------------------------------------------------------
         Consensus tgctaaagttattattaccctagaatacctactcagctactcttacaagaaaatcaacgcatacataacttctcataaattatccacctacttgaatatcaaatgtacaatatacagtaatttgggaata

                   1301 1310     1320      1330      1340      1350      1360      1370      1380      1390      1400      1410      1420    1430
                   |--------+---------+---------+---------+---------+---------+---------+---------+---------+---------+---------+---------+---------|
     BnaC.GLN1.2.a AGTCAATAATTATTTGTTTTTAATTTTTGTAAAAAGTCAATAATCAAATTTAAGGACGACACGAAAACAATAATCACCATTAAACTATATAACTTCAATTTAATACATTGGTTAGATAAAAAGAAATCGC
mRNA.BnaC.GLN1.2.a AGTCAATAATTATTTGTTTTTAATTTTTGTAAAAAGTCAATAATCAAATTTAAGGACGACACGAAAACAATAATCACCATTAAACTATATAACTTCAATTTAATACATTGGTTAGATAAAAAGAAATCGC
      BnaGln1.2_C2 ----------------------------------------------------------------------------------------------------------------------------------
         Consensus agtcaataattatttgtttttaatttttgtaaaaagtcaataatcaaatttaaggacgacacgaaaacaataatcaccattaaactatataacttcaatttaatacattggttagataaaaagaaatcgc

                   1431 1440     1450      1460      1470      1480      1490      1500      1510      1520      1530      1540      1550    1560
                   |--------+---------+---------+---------+---------+---------+---------+---------+---------+---------+---------+---------+---------|
     BnaC.GLN1.2.a GTTATTATATTTTATGGTTTTAATTTAAAAATAATATTTGGAAAACAGAGGCTCTGCTCTCTCTCTCCCTATCTCTGTAGGTACCCTCGTTGCTCTCTATAAGTACTCCCACAACCACGAACTCCAAAAA
mRNA.BnaC.GLN1.2.a GTTATTATATTTTATGGTTTTAATTTAAAAATAATATTTGGAAAACAGAGGCTCTGCTCTCTCTCTCCCTATCTCTGTAGGTACCCTCGTTGCTCTCTATAAGTACTCCCACAACCACGAACTCCAAAAA
      BnaGln1.2_C2 ----------------------------------------------------------------------------------------------------------------------------------
         Consensus gttattatattttatggttttaatttaaaaataatatttggaaaacagaggctctgctctctctctccctatctctgtaggtaccctcgttgctctctataagtactcccacaaccacgaactccaaaaa

                   1561 1570     1580      1590      1600      1610      1620      1630      1640      1650      1660      1670      1680    1690
                   |--------+---------+---------+---------+---------+---------+---------+---------+---------+---------+---------+---------+---------|
     BnaC.GLN1.2.a ATCATCTCATAAACCAAAAACCACATTATCCGAGATTTGAGTATA---TTTCACTACAACCTTCTTGTCATTTTCTCTGTAACCATGAGTCTTCTGACCGATCTCGTTAACCTTGACCTCTCAGACAACA
mRNA.BnaC.GLN1.2.a ATCATCTCATAAACCAAAAACCACATTATCCGAGATTTGAGTATA---TTTCACTACAACCTTCTTGTCATTTTCTCTGTAACCATGAGTCTTCTGACCGATCTCGTTAACCTTGACCTCTCAGACAACA
      BnaGln1.2_C2 ---ATCTCATAAACCAAAAACCACATTATCCGAGATTTGAGTATAATATTTCACTACAACCTTCTTGTCATTTTCTCTGTAACCATGAGTCTTCTGACCGATCTCGTTAACCTTGACCTCTCAGACAACA
         Consensus atcATCTCATAAACCAAAAACCACATTATCCGAGATTTGAGTATA...TTTCACTACAACCTTCTTGTCATTTTCTCTGTAACCATGAGTCTTCTGACCGATCTCGTTAACCTTGACCTCTCAGACAACA

                   1691 1700     1710      1720      1730      1740      1750      1760      1770      1780      1790      1800      1810    1820
                   |--------+---------+---------+---------+---------+---------+---------+---------+---------+---------+---------+---------+---------|
     BnaC.GLN1.2.a CTGAGAAAATCATCGCTGAATACATATGGTTCGTCTTTCTCCTTCGTTCTTGCCTCTTTCTTACACATTCTTGTTTATGTTCTTGTTCTTTCCTTATCCAGATAGATAGAAGATATTATTTTGTAATTAC
mRNA.BnaC.GLN1.2.a CTGAGAAAATCATCGCTGAATACATATGG-----------------------------------------------------------------------------------------------------
      BnaGln1.2_C2 CTGAGAAAATCATCGCTGAATACATATGG-----------------------------------------------------------------------------------------------------
         Consensus CTGAGAAAATCATCGCTGAATACATATGG.....................................................................................................

                   1821 1830     1840      1850      1860      1870      1880      1890      1900      1910      1920      1930      1940    1950
                   |--------+---------+---------+---------+---------+---------+---------+---------+---------+---------+---------+---------+---------|
     BnaC.GLN1.2.a ATATTTACATCTTCTACCACACAAAAAAAGAGTTATGTGTTTTGTCTTTTCTTATTTTGGTTTTTTCTTTTGAAAAAAAAAACAGGGTTGGTGGTTCAGGAATGGATATGAGAAGCAAAGCCAGGGTATA
mRNA.BnaC.GLN1.2.a --------------------------------------------------------------------------------------GTTGGTGGTTCAGGAATGGATATGAGAAGCAAAGCCAGG-----
      BnaGln1.2_C2 --------------------------------------------------------------------------------------GTTGGTGGTTCAGGAATGGATATGAGAAGCAAAGCCAGG-----
```

```
         Consensus  ......................................................................................GTTGGTGGTTCAGGAATGGATATGAGAAGCAAAGCCAGG.....

                    1951 1960      1970      1980      1990      2000      2010      2020      2030      2040      2050      2060      2070      2080
                    |--------+---------+---------+---------+---------+---------+---------+---------+---------+---------+---------+---------+---------|
     BnaC.GLN1.2.a  TGTTTTTCACTTTAACTCGACGAAAAATAAAAAAAAATTGTCGGAAAAATTGACCCAGACGGAGAAAGCTTAGGAGTTTGACTATACTGAGTTACTGATTCTCTGTTTTGTTTACCTGCAGACTCTCCCTG
mRNA.BnaC.GLN1.2.a  ------------------------------------------------------------------------------------------------------------------------ACTCTCCCTG
     BnaGln1.2_C2   ------------------------------------------------------------------------------------------------------------------------ACTCTCCCTG
         Consensus  ........................................................................................................................ACTCTCCCTG

                    2081 2090      2100      2110      2120      2130      2140      2150      2160      2170      2180      2190      2200      2210
                    |--------+---------+---------+---------+---------+---------+---------+---------+---------+---------+---------+---------+---------|
     BnaC.GLN1.2.a  GACCTGTGACCGATCCATCAAAGCTCCCAAAATGGAATTATGATGGTTCAAGCACTGGCCAAGCTCCTGGTGAAGACAGTGAAGTGATCTTATAGTAAGGCCTTTTCTCAACTTTTATCACTATTTCAAA
mRNA.BnaC.GLN1.2.a  GACCTGTGACCGATCCATCAAAGCTCCCAAAATGGAATTATGATGGTTCAAGCACTGGCCAAGCTCCTGGTGAAGACAGTGAAGTGATCTTATA------------------------------------
     BnaGln1.2_C2   GACCTGTGACCGATCCATCAAAGCTCCCAAAATGGAATTATGATGGTTCAAGCACTGGCCAAGCTCCTGGTGAAGACAGTGAAGTGATCTTATA------------------------------------
         Consensus  GACCTGTGACCGATCCATCAAAGCTCCCAAAATGGAATTATGATGGTTCAAGCACTGGCCAAGCTCCTGGTGAAGACAGTGAAGTGATCTTATA....................................

                    2211 2220      2230      2240      2250      2260      2270      2280      2290      2300      2310      2320      2330      2340
                    |--------+---------+---------+---------+---------+---------+---------+---------+---------+---------+---------+---------+---------|
     BnaC.GLN1.2.a  CTCATCTTTTTATTTTTTTTTTTGCTTAATAACCTTCCTTTTTATTTGTGTTCCTTAGCCCTCAAGCGATTTTCAAAGATCCGTTCCGTAGAGGCAACAACATTCTTGTGAGTTGTTAAACTTTGGTTTTCT
mRNA.BnaC.GLN1.2.a  --------------------------------------------------------CCCTCAAGCGATTTTCAAAGATCCGTTCCGTAGAGGCAACAACATTCTTGT-----------------------
     BnaGln1.2_C2   --------------------------------------------------------CCCTCAAGCGATTTTCAAAGATCCGTTCCGTAGAGGCAACAACATTCTTGT-----------------------
         Consensus  ........................................................CCCTCAAGCGATTTTCAAAGATCCGTTCCGTAGAGGCAACAACATTCTTGT.......................

                    2341 2350      2360      2370      2380      2390      2400      2410      2420      2430      2440      2450      2460      2470
                    |--------+---------+---------+---------+---------+---------+---------+---------+---------+---------+---------+---------+---------|
     BnaC.GLN1.2.a  TGCGCATGTTCTGTTTTGGCAGTTAATGATAACGTTTTTTATCGGTTTGGTTAGGTCATGTGTGATACTTACACCCCTGCGGGTGAACCAATCCCTACGAACAAGAGACATGCTGCAGCTCAGATCTTTA
mRNA.BnaC.GLN1.2.a  --------------------------------------------------------CATGTGTGATACTTACACCCCTGCGGGTGAACCAATCCCTACGAACAAGAGACATGCTGCAGCTCAGATCTTTA
     BnaGln1.2_C2   --------------------------------------------------------CATGTGTGATACTTACACCCCTGCGGGTGAACCAATCCCTACGAACAAGAGACATGCTGCAGCTCAGATCTTTA
         Consensus  ........................................................CATGTGTGATACTTACACCCCTGCGGGTGAACCAATCCCTACGAACAAGAGACATGCTGCAGCTCAGATCTTTA

                    2471 2480      2490      2500      2510      2520      2530      2540      2550      2560      2570      2580      2590      2600
                    |--------+---------+---------+---------+---------+---------+---------+---------+---------+---------+---------+---------+---------|
     BnaC.GLN1.2.a  GCAACCCTGATGTTGTTGCTGAAGTGCCATGGTTAACCCAAATTCCCCTGTTCCTGTTTTCGTTATATATTTTTTTGGTTTCTTGCGGTCTGATTTAGCTCTGCATACTAGGTATGGAATCGAACAAGAG
mRNA.BnaC.GLN1.2.a  GCAACCCTGATGTTGTTGCTGAAGTGCCATGGT--------------------------------------------------------------------------------ATGGAATCGAACAAGAG
     BnaGln1.2_C2   GCAACCCTGATGTTGTTGCTGAAGTGCCATGGT--------------------------------------------------------------------------------ATGGAATCGAACAAGAG
         Consensus  GCAACCCTGATGTTGTTGCTGAAGTGCCATGGT................................................................................ATGGAATCGAACAAGAG

                    2601 2610      2620      2630      2640      2650      2660      2670      2680      2690      2700      2710      2720      2730
                    |--------+---------+---------+---------+---------+---------+---------+---------+---------+---------+---------+---------+---------|
     BnaC.GLN1.2.a  TACACTCTGTTGCAGAAAGATGTGAAGTGGCCTGTTGGATGGCCCATTGGTGGATTCCCCGGCCCTCAGGTATATTCCGTTTTCAGAGTTCTTGTTCAATTCTCTGTTCTCAGCTTCTAATGTTTATACA
mRNA.BnaC.GLN1.2.a  TACACTCTGTTGCAGAAAGATGTGAAGTGGCCTGTTGGATGGCCCATTGGTGGATTCCCCGGCCCTCAGG------------------------------------------------------------
     BnaGln1.2_C2   TACACTCTGTTGCAGAAAGATGTGAAGTGGCCTGTTGGATGGCCCATTGGTGGATTCCCCGGCCCTCAGG------------------------------------------------------------
         Consensus  TACACTCTGTTGCAGAAAGATGTGAAGTGGCCTGTTGGATGGCCCATTGGTGGATTCCCCGGCCCTCAGG............................................................

                    2731 2740      2750      2760      2770      2780      2790      2800      2810      2820      2830      2840      2850      2860
                    |--------+---------+---------+---------+---------+---------+---------+---------+---------+---------+---------+---------+---------|
     BnaC.GLN1.2.a  AAAACTATCTTTCTCAGGGACCATACTACTGCAGTGTTGGAGCTGACAAATCTTTTGGAAGAGACATTGTTGATGCTCACTACAAGGCTTGTTTGTATGCCGGAATTAACATCAGTGGAATCAATGGAGA
mRNA.BnaC.GLN1.2.a  ------------------GACCATACTACTGCAGTGTTGGAGCTGACAAATCTTTTGGAAGAGACATTGTTGATGCTCACTACAAGGCTTGTTTGTATGCCGGAATTAACATCAGTGGAATCAATGGAGA
     BnaGln1.2_C2   ------------------GACCATACTACTGCAGTGTTGGAGCTGACAAATCTTTTGGAAGAGACATTGTTGATGCTCACTACAAGGCTTGTTTGTATGCCGGAATTAACATCAGTGGAATCAATGGAGA
         Consensus  ..................GACCATACTACTGCAGTGTTGGAGCTGACAAATCTTTTGGAAGAGACATTGTTGATGCTCACTACAAGGCTTGTTTGTATGCCGGAATTAACATCAGTGGAATCAATGGAGA

                    2861 2870      2880      2890      2900      2910      2920      2930      2940      2950      2960      2970      2980      2990
                    |--------+---------+---------+---------+---------+---------+---------+---------+---------+---------+---------+---------+---------|
     BnaC.GLN1.2.a  AGTCATGCCTGGTCAGGTATAACTTCAAGATCCAACAGAATTAAATCTTTTTGCATAATGTAGTAATGACCAAATCATTTTTTTTTTTGTTGAGACAGTGGGAGTTCCAAGTCGGACCATCGGTTGGTAT
mRNA.BnaC.GLN1.2.a  AGTCATGCCTGGTCAG----------------------------------------------------------------------------------TGGGAGTTCCAAGTCGGACCATCGGTTGGTAT
     BnaGln1.2_C2   AGTCATGCCTGGTCAG----------------------------------------------------------------------------------TGGGAGTTCCAAGTCGGACCATCGGTTGGTAT
         Consensus  AGTCATGCCTGGTCAG..................................................................................TGGGAGTTCCAAGTCGGACCATCGGTTGGTAT

                    2991 3000      3010      3020      3030      3040      3050      3060      3070      3080      3090      3100      3110      3120
                    |--------+---------+---------+---------+---------+---------+---------+---------+---------+---------+---------+---------+---------|
     BnaC.GLN1.2.a  CTCAGCTGCTGATGAAGTGTGGATTGCTCGTTACATTTTGGAGGTATCATTCAACCATCATCCACTCTTAATTCTTGTTGTTATCCTAATGGAATATAAACTTGAGTACAATTTTTTGGTGGTTTTAAAA
mRNA.BnaC.GLN1.2.a  CTCAGCTGCTGATGAAGTGTGGATTGCTCGTTACATTTTGGAG---------------------------------------------------------------------------------------
     BnaGln1.2_C2   CTCAGCTGCTGATGAAGTGTGGATTGCTCGTTACATTTTGGAG---------------------------------------------------------------------------------------
         Consensus  CTCAGCTGCTGATGAAGTGTGGATTGCTCGTTACATTTTGGAG.......................................................................................

                    3121 3130      3140      3150      3160      3170      3180      3190      3200      3210      3220      3230      3240      3250
                    |--------+---------+---------+---------+---------+---------+---------+---------+---------+---------+---------+---------+---------|
     BnaC.GLN1.2.a  AACAGAGGATCACAGAGATTGCTGGTGTGGTTGTATCTTTTGACCCAAAACCAATTCCGGGTGACTGGAACGGAGCTGGTGCTCACACCAATTACAGGTAAAAGGATCATGCAACTTAAGCTTGTTATAT
mRNA.BnaC.GLN1.2.a  -----AGGATCACAGAGATTGCTGGTGTGGTTGTATCTTTTGACCCAAAACCAATTCCGGGTGACTGGAACGGAGCTGGTGCTCACACCAATTACAG---------------------------------
     BnaGln1.2_C2   -----AGGATCACAGAGATTGCTGGTGTGGTTGTATCTTTTGACCCAAAACCAATTCCGGGTGACTGGAATGGAGCTGGTGCTCACACCAATTACAG---------------------------------
         Consensus  .....AGGATCACAGAGATTGCTGGTGTGGTTGTATCTTTTGACCCAAAACCAATTCCGGGTGACTGGAAcGGAGCTGGTGCTCACACCAATTACAG.................................

                    3251 3260      3270      3280      3290      3300      3310      3320      3330      3340      3350      3360      3370      3380
                    |--------+---------+---------+---------+---------+---------+---------+---------+---------+---------+---------+---------+---------|
     BnaC.GLN1.2.a  AATTTGTTCTTTTGGAAAGAAAAAATCTGAAATATGGTAAATGACTTTTTCAGTACTAAATCGATGAGGGAGGAAGGAGGATACGAGATAATCAAGAAGGCAATTGATAAGCTCGGACTGAGACACAAGG
mRNA.BnaC.GLN1.2.a  -----------------------------------------------------TACTAAATCGATGAGGGAGGAAGGAGGATACGAGATAATCAAGAAGGCAATTGATAAGCTCGGACTGAGACACAAGG
     BnaGln1.2_C2   -----------------------------------------------------TACTAAATCGATGAGGGAGGAAGGAGGATACGAGATAATCAAGAAGGCAATTGATAAGCTCGGACTGAGACACAAGG
         Consensus  .....................................................TACTAAATCGATGAGGGAGGAAGGAGGATACGAGATAATCAAGAAGGCAATTGATAAGCTCGGACTGAGACACAAGG

                    3381 3390      3400      3410      3420      3430      3440      3450      3460      3470      3480      3490      3500      3510
                    |--------+---------+---------+---------+---------+---------+---------+---------+---------+---------+---------+---------+---------|
     BnaC.GLN1.2.a  AGCACATTTCTGCTTACGGTGAAGGCAACGAGCGTCGTCTCACCGGACACCATGAAACTGCTGACATCAACACTTTCAAATGGGTAAACATTTAAATTTTTTAAAGAGTTGATAACATTTTTGTGTACTG
mRNA.BnaC.GLN1.2.a  AGCACATTTCTGCTTACGGTGAAGGCAACGAGCGTCGTCTCACCGGACACCATGAAACTGCTGACATCAACACTTTCAAATGGG----------------------------------------------
     BnaGln1.2_C2   AGCACATTTCTGCTTACGGTGAAGGCAACGAGCGTCGTCTCACCGGACACCATGAAACTGCTGACATCAACACTTTCAAATGGG----------------------------------------------
         Consensus  AGCACATTTCTGCTTACGGTGAAGGCAACGAGCGTCGTCTCACCGGACACCATGAAACTGCTGACATCAACACTTTCAAATGGG..............................................

                    3511 3520      3530      3540      3550      3560      3570      3580      3590      3600      3610      3620      3630      3640
                    |--------+---------+---------+---------+---------+---------+---------+---------+---------+---------+---------+---------+---------|
     BnaC.GLN1.2.a  AACTTTGTGTTAATTTTTTTTGAATCTTTGTTAAAAGGGTGTTGCAAACCGTGGAGCATCAATCCGTGTAGGACGTGACACGGAGAAGGAAGGGAAAGGATACTTTGAGGATAGGAGGCCAGCTTCCAAC
mRNA.BnaC.GLN1.2.a  --------------------------------------GTGTTGCAAACCGTGGAGCATCAATCCGTGTAGGACGTGACACGGAGAAGGAAGGGAAAGGATACTTTGAGGATAGGAGGCCAGCTTCCAAC
     BnaGln1.2_C2   --------------------------------------GTGTTGCAAACCGTGGAGCATCAATCCGTGTAGGACGTGACACGGAGAAGGAAGGGAAAGGATACTTTGAGGATAGGAGGCCAGCTTCCAAC
         Consensus  ......................................GTGTTGCAAACCGTGGAGCATCAATCCGTGTAGGACGTGACACGGAGAAGGAAGGGAAAGGATACTTTGAGGATAGGAGGCCAGCTTCCAAC

                    3641 3650      3660      3670      3680      3690      3700      3710      3720      3730      3740      3750      3760      3770
                    |--------+---------+---------+---------+---------+---------+---------+---------+---------+---------+---------+---------+---------|
     BnaC.GLN1.2.a  ATGGACCCTTACACTGTAACTTCCATGATTGCAGAGACTACACTTCTTTGGAATCCTTGAAGAAATATGACAATAATATAACTTGAATCTGGTTCTCTGGGGCTTCAAGTTTGTGTTTTCTACAGTTTAT
mRNA.BnaC.GLN1.2.a  ATGGACCCTTACACTGTAACTTCCATGATTGCAGAGACTACACTTCTTTGGAATCCTTGAAGAAATATGACAATAATATAACTTGAATCTGGTTCTCTGGGGCTTCAAGTTTGTGTTTTCTACAGTTTAT
     BnaGln1.2_C2   ATGGACCCTTACACTGTAACTTCCATGATTGCAGAGACTACACTTCTTTGGAATCCTTGAAGAAATATGACAATAATATAACTTGAATCTGGTTCTCTGGGGCTTCAAGTTTGTGTTTTCTACAGTTTAT
         Consensus  ATGGACCCTTACACTGTAACTTCCATGATTGCAGAGACTACACTTCTTTGGAATCCTTGAAGAAATATGACAATAATATAACTTGAATCTGGTTCTCTGGGGCTTCAAGTTTGTGTTTTCTACAGTTTAT

                    3771 3780      3790      3800      3810      3820      3830      3840      3850      3860      3870      3880      3890      3900
                    |--------+---------+---------+---------+---------+---------+---------+---------+---------+---------+---------+---------+---------|
     BnaC.GLN1.2.a  TAAGCAATTACCGGGTTGATACTGCCGGAGTTTGTGGTTTGAGGC-TTTCTTTTAATCTCTTTGTGTTTTGGGGTTTGTGATTGAAGCAAAAACCCTGATTTGCTCTGTTTCTTTGACCTTTTATTTGAA
mRNA.BnaC.GLN1.2.a  TAAGCAATTACCGGGTTGATACTGCCGGAGTTTGTGGTTTGAGGC-TTTCTTTTAATCTCTTTGTGTTTTGGGGTTTGTGATTGAAGCAAAAACCCTGATTTGCTCTGTTTCTTTGACCTTTTATTTGAA
```

```
mRNA.BnaC.GLN1.2.a  [illegible]
      BnaGln1.2_C2  TAAGCAATTACCGGGTTGATACTGCCAGAGTTTGTGGTTTGAGGCCTTTCTTTTAATCTCTTTGTGTTTTGGGGTTTGTGATTGAAGCAAAAACCCTGATTTGCTCTGTTTCTTTGACCTTTTATTTGAA
         Consensus  TAAGCAATTACCGGGTTGATACTGCCgGAGTTTGTGGTTTGAGGC.TTTCTTTTAATCTCTTTGTGTTTTGGGGTTTGTGATTGAAGCAAAAACCCTGATTTGCTCTGTTTCTTTGACCTTTTATTTGAA

                    3901 3910      3920      3930      3940      3950      3960      3970      3980      3990      4000      4010      4020      4030
                    |--------+---------+---------+---------+---------+---------+---------+---------+---------+---------+---------+---------+---------|
    BnaC.GLN1.2.a   CCCTTTGTATTTCTATTAATAAGACGATCTGAGAAGGCCTTTTCATGTTTCAACTTAGACTGTTCAACAAGTTCACAAATAACAAAACTCTTTGGTAGGCCCAGAACCTGTAAGCTAGACTCTAAAACTA
mRNA.BnaC.GLN1.2.a  CCCTTTGTATTTCTATTAATAAGACGATCTGAGAAGGCCTTTTCATGTTTCAACTTAGACTGTTCAACAAGTTCACAAATAACAAAACTCTTTGGTAGGCCCAGAACCTGTAAGCTAGACTCTAAAACTA
      BnaGln1.2_C2  CCCTTTGTATTTCTATTAATAAGACGATCTGAGAAGGCCTTTCCATGTTCCAAAAAAAAAAAAAAAAAAAAA
         Consensus  CCCTTTGTATTTCTATTAATAAGACGATCTGAGAAGGCCTTTtCATGTTtCAActtAgActgttcAAcAAgttcacaaataacaaaactctttggtaggcccagaacctgtaagctagactctaaaacta

                    4031 4040      4050      4060      4070   4077
                    |--------+---------+---------+---------+------|
    BnaC.GLN1.2.a   TTGGTAGTTGGTACACACATAAATGTATTGTAGGAACCGTTATAAAA
mRNA.BnaC.GLN1.2.a  TTGGTAGTTGGTACACACATAAATGTATTGTAGGAACCGTTATAAAA
      BnaGln1.2_C2
         Consensus  ttggtagttggtacacacataaatgtattgtaggaaccgttataaaa
```

```
                1         10        20        30        40        50        60        70        80        90        100       110       120       130
BnaA.GLN1.3.a   ATAAAAATGACAACTTTTTTTTTGTTACAGTACAACTAAAGGAGAAAAGTTTCTAGTTGTATACAAAAGATGTATCGATCTTATAGTTTCTCTGTACGATTCAATTATTGAGCTGCATTGATTGAGTCCA
mRNA.BnaA.GLN1.3.a
BnaGln1.3_C2                                                                                                                          TGAATGCTGTGA
Consensus       ......................................................................................................................tga.tg.....a

                131       140       150       160       170       180       190       200       210       220       230       240       250       260
BnaA.GLN1.3.a   TGCAAGTTGGTTAAAAGGTCATGTCTATTTAGACACATCAGGTTAATGCGAAGAGCAAAACAGGGCGCAACAAAATGGGCTTTATGACAAATTGGAGGAAAACGTTACGTACGCAAAATACAAAAGTGTC
mRNA.BnaA.GLN1.3.a                      ATGTCTATTTAGACACATCAGGTTAATGCGAAGAGCAAAACAGGGCGCAACAAAATGGGCTTTATGACAAATTGGAGGAAAACGTTACGTACGCAAAATACAAAAGTGTC
BnaGln1.3_C2    CGCCAGCTCGAAATAACC----------------------------------------------------------------------------------------------------------------
Consensus       .gc.ag.t.g..a.aa....atgtctatttagacacatcaggttaatgcgaagagcaaaacagggcgcaacaaaatgggctttatgacaaattggaggaaaacgttacgtacgcaaaatacaaaagtgtc

                261       270       280       290       300       310       320       330       340       350       360       370       380       390
BnaA.GLN1.3.a   CACGTCTTGATTCATCATATGATAATACTTTTATAGTCATTAGTGCAAAACTCATTTCATTTCGTATCTCACTAAACTACGTTTACGTGTTCATCAACAATTCTAATTTTACTGATTTACTCATAAACAT
mRNA.BnaA.GLN1.3.a CACGTCTTGATTCATCATATGATAATACTTTTATAGTCATTAGTGCAAAACTCATTTCATTTCGTATCTCACTAAACTACGTTTACGTGTTCATCAACAATTCTAATTTTACTGATTTACTCATAAACAT
BnaGln1.3_C2    ----------------------------------------------------------------------------------------------------------------------------------
Consensus       cacgtcttgattcatcatatgataatacttttatagtcattagtgcaaaactcatttcatttcgtatctcactaaactacgtttacgtgttcatcaacaattctaattttactgatttactcataaacat

                391       400       410       420       430       440       450       460       470       480       490       500       510       520
BnaA.GLN1.3.a   ATCCTCCAGTTATCCATAATCAGGTTTGTACTAAAAGACAAAAGATATGAAGAATATCAAGAAACTTCTTAGTTTTTGCGAATTGCACCCGTTTCATCATCCTACGATTCATAAAACTTAGCTACAACAA
mRNA.BnaA.GLN1.3.a ATCCTCCAGTTATCCATAATCAGGTTTGTACTAAAAGACAAAAGATATGAAGAATATCAAGAAACTTCTTAGTTTTTGCGAATTGCACCCGTTTCATCATCCTACGATTCATAAAACTTAGCTACAACAA
BnaGln1.3_C2    ----------------------------------------------------------------------------------------------------------------------------------
Consensus       atcctccagttatccataatcaggtttgtactaaaagacaaaagatatgaagaatatcaagaaacttcttagtttttgcgaattgcacccgtttcatcatcctacgattcataaaacttagctacaacaa

                521       530       540       550       560       570       580       590       600       610       620       630       640       650
BnaA.GLN1.3.a   ACTAAAAGACCTTCGTCTATCAATAGTGCAACCACTTTTTCTTCATGTAGTAACCACAGAAGAGAATTAGTTCCGTTTGATCATAAATAAACTCCTTTCGCCTGTTCATAATAATAAAGAAACACTTAAA
mRNA.BnaA.GLN1.3.a ACTAAAAGACCTTCGTCTATCAATAGTGCAACCACTTTTTCTTCATGTAGTAACCACAGAAGAGAATTAGTTCCGTTTGATCATAAATAAACTCCTTTCGCCTGTTCATAATAATAAAGAAACACTTAAA
BnaGln1.3_C2    ----------------------------------------------------------------------------------------------------------------------------------
Consensus       actaaaagaccttcgtctatcaatagtgcaaccactttttcttcatgtagtaaccacagaagagaattagttccgtttgatcataaataaactcctttcgcctgttcataataataaagaaacacttaaa

                651       660       670       680       690       700       710       720       730       740       750       760       770       780
BnaA.GLN1.3.a   ATAATAATTGTAATTATCTCTGTGTAAATGTCAGGAGAATCCTAACGGAATCGTTTATCTAATAAAATTAACTATTAAAAAAATCAAAAGATAATGTTGGCAGACAACAGTAAATAAAATCATGTAATTA
mRNA.BnaA.GLN1.3.a ATAATAATTGTAATTATCTCTGTGTAAATGTCAGGAGAATCCTAACGGAATCGTTTATCTAATAAAATTAACTATTAAAAAAATCAAAAGATAATGTTGGCAGACAACAGTAAATAAAATCATGTAATTA
BnaGln1.3_C2    -----------------------------------------------------------------------CTACTAAAGGGAACAAAAGCTGGAGCTCC------------------------------
Consensus       ataataattgtaattatctctgtgtaaatgtcaggagaatcctaacggaatcgtttatctaataaaattaaCTAtTAAAaaaAtCAAAAGaTaatGtTggcagacaacagtaaataaaatcatgtaatta

                781       790       800       810       820       830       840       850       860       870       880       890       900       910
BnaA.GLN1.3.a   TTTGAAAATAAAAAATAAATTAAAAAAAACAAAAAAATAAGGCTATAAAATAAAAACACTCGTCAGGAGAGAAGCTGCATTGATCGTCTTCCCTTAGACAAACACTGATTGATTATCTTTCTCTTTTCTC
mRNA.BnaA.GLN1.3.a TTTGAAAATAAAAAATAAATTAAAAAAAACAAAAAAATAAGGCTATAAAATAAAAACACTCGTCAGGAGAGAAGCTGCATTGATCGTCTTCCCTTAGACAAACACTGATTGATTATCTTTCTCTTTTCTC
BnaGln1.3_C2    -------------------------------------------------------ACCGCGGTGGCGGCCGCTCTGGCATTGATCGTCTTCCCTTAGACAAACACTGATTGATTATCTTTCTCTTTTCTC
Consensus       tttgaaaataaaaaataaattaaaaaaaacaaaaaaataaggctataaaataaaaACactcGTcagGagaGaagctGCATTGATCGTCTTCCCTTAGACAAACACTGATTGATTATCTTTCTCTTTTCTC

                911       920       930       940       950       960       970       980       990       1000      1010      1020      1030      1040
BnaA.GLN1.3.a   TCGTGACGGCGCCATGTCTCTCCTCTCAGATCTCGTCAACCTCAACCTCTCTGACTCCACCGAGAAAATCATCGCCGAATACATATGGTTCGATCTCCCCTTCTCTCTCTTTCACGACGAGTGTCATTAT
mRNA.BnaA.GLN1.3.a TCGTGACGGCGCCATGTCTCTCCTCTCAGATCTCGTCAACCTCAACCTCTCTGACTCCACCGAGAAAATCATCGCCGAATACATATGG------------------------------------------
BnaGln1.3_C2    TCGTGACGGCGCCATGTCTCTCCTCTCAGATCTCGTCAACCTCAACCTCTCTGACTCCACCGAGAAAATCATCGCCGAATACATATGG------------------------------------------
Consensus       TCGTGACGGCGCCATGTCTCTCCTCTCAGATCTCGTCAACCTCAACCTCTCTGACTCCACCGAGAAAATCATCGCCGAATACATATGG..........................................

                1041      1050      1060      1070      1080      1090      1100      1110      1120      1130      1140      1150      1160      1170
BnaA.GLN1.3.a   TTTGGTTTCTTTCTATTAATACGATCGTAAAGTTTTCACCTTTCTTCATCTTGCTATTATTGATCTGTTATGGTTTTGAGAGTTTCTCTATGAACACGGTTCACTGAGTTTGACGTAGTATACATCATAA
mRNA.BnaA.GLN1.3.a ----------------------------------------------------------------------------------------------------------------------------------
BnaGln1.3_C2    ----------------------------------------------------------------------------------------------------------------------------------
Consensus       ..................................................................................................................................

                1171      1180      1190      1200      1210      1220      1230      1240      1250      1260      1270      1280      1290      1300
BnaA.GLN1.3.a   AGCATGTCTCTTTTTTGATATTTTCTTTCTTGTCGTCTTCTGGGTTTGTGAGTTTTCGTATGAACTTTGAATGAACAGGATCGGTGGATCTGGAATGGACATCAGAAGCAAAGCCAGGGTAATTAAATAA
mRNA.BnaA.GLN1.3.a -------------------------------------------------------------------------------ATCGGTGGATCTGGAATGGACATCAGAAGCAAAGCCAGG------------
BnaGln1.3_C2    -------------------------------------------------------------------------------ATCGGTGGATCTGGAATGGACATCAGAAGCAAAGCCAGG------------
Consensus       ...............................................................................ATCGGTGGATCTGGAATGGACATCAGAAGCAAAGCCAGG............

                1301      1310      1320      1330      1340      1350      1360      1370      1380      1390      1400      1410      1420      1430
BnaA.GLN1.3.a   ATACTCTGTTTCTTGATCCTCTGTTTTTATCCTCTGTTTTTTTATTTGGCTGATCTGATTTGTTTTGGATGATACAGACACTACCAGGACCAGTGAGCGATCCATCAAAGCTGCCTAAGTGGAACTACGA
mRNA.BnaA.GLN1.3.a -----------------------------------------------------------------------------ACACTACCAGGACCAGTGAGCGATCCATCAAAGCTGCCTAAGTGGAACTACGA
BnaGln1.3_C2    -----------------------------------------------------------------------------ACACTACCAGGACCAGTGAGCGATCCATCAAAGCTGCCTAAGTGGAACTACGA
Consensus       .............................................................................ACACTACCAGGACCAGTGAGCGATCCATCAAAGCTGCCTAAGTGGAACTACGA

                1431      1440      1450      1460      1470      1480      1490      1500      1510      1520      1530      1540      1550      1560
BnaA.GLN1.3.a   CGGATCCAGCACCGGTCAAGCCGCCGGAGATGACAGTGAAGTCATTCTATAGTATGTTCTTTTATTATCTCTATTTACATATAAACATCAAGTAAATATGAAGTAAAAAGTGTTGATCTTTGTTTTGTT
mRNA.BnaA.GLN1.3.a CGGATCCAGCACCGGTCAAGCCGCCGGAGATGACAGTGAAGTCATTCTATA-------------------------------------------------------------------------------
BnaGln1.3_C2    CGGATCCAGCACCGGTCAAGCCGCCGGAGATGACAGTGAAGTCATTCTATA-------------------------------------------------------------------------------
Consensus       CGGATCCAGCACCGGTCAAGCCGCCGGAGATGACAGTGAAGTCATTCTATA...............................................................................

                1561      1570      1580      1590      1600      1610      1620      1630      1640      1650      1660      1670      1680      1690
BnaA.GLN1.3.a   TTGTTAATGAATGATCGATAGCCCTCAGGCGATATTCCGTGATCCGTTCAGGAAAGGCAACAACATTCTGGTAGGTTTAATTTTATATGATTAGAGCGTGACAATATTCATTTGTTACAAAATCAAATTT
mRNA.BnaA.GLN1.3.a ---------------------CCCTCAGGCGATATTCCGTGATCCGTTCAGGAAAGGCAACAACATTCTGGT----------------------------------------------------------
BnaGln1.3_C2    ---------------------CCCTCAGGCGATATTCCGTGATCCGTTCAGGAAAGGCAACAACATTCTGGT----------------------------------------------------------
Consensus       .....................CCCTCAGGCGATATTCCGTGATCCGTTCAGGAAAGGCAACAACATTCTGGT..........................................................

                1691      1700      1710      1720      1730      1740      1750      1760      1770      1780      1790      1800      1810      1820
BnaA.GLN1.3.a   TAAGAAAATCATTGTTTATTGTTATCTATATATGCAGGTGATGTGTGATGCTTACACGCCGGCAGGGAATCCAATTCCGACCAACAAGAGGCACAACGCTGCTAAGATCTTCAGCAACCCCAAAGTTGCC
mRNA.BnaA.GLN1.3.a ---------------------------------------GATGTGTGATGCTTACACGCCGGCAGGGAATCCAATTCCGACCAACAAGAGGCACAACGCTGCTAAGATCTTCAGCAACCCCAAAGTTGCC
BnaGln1.3_C2    ---------------------------------------GATGTGTGATGCTTACACGCCGGCAGGGAATCCAATTCCGACCAACAAGAGGCACAACGCTGCTAAGATCTTCAGCAACCCCAAAGTTGCC
Consensus       .......................................GATGTGTGATGCTTACACGCCGGCAGGGAATCCAATTCCGACCAACAAGAGGCACAACGCTGCTAAGATCTTCAGCAACCCCAAAGTTGCC

                1821      1830      1840      1850      1860      1870      1880      1890      1900      1910      1920      1930      1940      1950
BnaA.GLN1.3.a   TCTGAGGAGCCTTGGTAAGCTCCTCTTCTTCTTCCTCTATTGATCTCTCTGTTAATCAGTTAATCATATACTAATACTCTTCTCTTCCCTTTGACTTAAAAAGGTATGGGATTGAGCAAGAATACACATT
mRNA.BnaA.GLN1.3.a TCTGAGGAGCCTTGGTA-----------------------------------------------------------------------------------------TGGGATTGAGCAAGAATACACATT
BnaGln1.3_C2    TCTGAGGAGCCTTGGTA-----------------------------------------------------------------------------------------TGGGATTGAGCAAGAATACACATT
```

```
        Consensus  TCTGAGGAGCCTTGGTA.........................................................................................TGGGATTGAGCAAGAATACACATT

                   1951 1960      1970      1980      1990      2000      2010      2020      2030      2040      2050      2060      2070      2080
                   |--------+---------+---------+---------+---------+---------+---------+---------+---------+---------+---------+---------+---------|
   BnaA.GLN1.3.a   GATGCAGAAGGGTGTGAACTGGCCTATTGGTTGGCCTATCGGTGGCTTCCCTGGCCCTCAGGGACCTTACTACTGTGGTGTGGGAGCTGACAAGGCAATTGGTCGTGACATTGTGGATGCACACTACAAG
mRNA.BnaA.GLN1.3.a GATGCAGAAGGGTGTGAACTGGCCTATTGGTTGGCCTATCGGTGGCTTCCCTGGCCCTCAGGGACCTTACTACTGTGGTGTGGGAGCTGACAAGGCAATTGGTCGTGACATTGTGGATGCACACTACAAG
    BnaGln1.3_C2   GATGCAGAAGGGTGTGAACTGGCCTATTGGTTGGCCTATCGGTGGCTTCCCTGGCCCTCAGGGACCTTACTACTGTGGTGTGGGAGCTGACAAGGCAATTGGTCGTGACATTGTGGATGCACACTACAAG
       Consensus   GATGCAGAAGGGTGTGAACTGGCCTATTGGTTGGCCTATCGGTGGCTTCCCTGGCCCTCAGGGACCTTACTACTGTGGTGTGGGAGCTGACAAGGCAATTGGTCGTGACATTGTGGATGCACACTACAAG

                   2081 2090      2100      2110      2120      2130      2140      2150      2160      2170      2180      2190      2200      2210
                   |--------+---------+---------+---------+---------+---------+---------+---------+---------+---------+---------+---------+---------|
   BnaA.GLN1.3.a   GCCTGTATTTACGCAGGTATTGGCATCTCTGGTGTCAATGGAGAAGTCATGCCTGGTCAGTGGGAGTTCCAAGTCGGTCCTGTTGAGGGTATTAGTGCTGGTGACCAAGTTTGGGTCGCTAGATACCTTC
mRNA.BnaA.GLN1.3.a GCCTGTATTTACGCAGGTATTGGCATCTCTGGTGTCAATGGAGAAGTCATGCCTGGTCAGTGGGAGTTCCAAGTCGGTCCTGTTGAGGGTATTAGTGCTGGTGACCAAGTTTGGGTCGCTAGATACCTTC
    BnaGln1.3_C2   GCCTGTATTTACGCAGGTATTGGCATCTCTGGTGTCAATGGAGAAGTCATGCCTGGTCAGTGGGAGTTCCAAGTCGGTCCTGTTGAGGGTATTAGTGCTGGTGACCAAGTTTGGGTCGCTAGATACCTTC
       Consensus   GCCTGTATTTACGCAGGTATTGGCATCTCTGGTGTCAATGGAGAAGTCATGCCTGGTCAGTGGGAGTTCCAAGTCGGTCCTGTTGAGGGTATTAGTGCTGGTGACCAAGTTTGGGTCGCTAGATACCTTC

                   2211 2220      2230      2240      2250      2260      2270      2280      2290      2300      2310      2320      2330      2340
                   |--------+---------+---------+---------+---------+---------+---------+---------+---------+---------+---------+---------+---------|
   BnaA.GLN1.3.a   TCGAGGTAAGACTAATCTGTACTTCACCGGTTATGTTCCCCATAAAATGCTTCAAACTTTGCTAACGGTTATCATCTTTCCGACAGAGGATCACTGAGATCTCTGGTGTGAATGTCAGCTTCGACCCTAA
mRNA.BnaA.GLN1.3.a TCGAG---------------------------------------------------------------------------------AGGATCACTGAGATCTCTGGTGTGAATGTCAGCTTCGACCCTAA
    BnaGln1.3_C2   TCGAG---------------------------------------------------------------------------------AGGATCACTGAGATCTCTGGTGTGAATGTCAGCTTCGACCCTAA
       Consensus   TCGAG.................................................................................AGGATCACTGAGATCTCTGGTGTGAATGTCAGCTTCGACCCTAA

                   2341 2350      2360      2370      2380      2390      2400      2410      2420      2430      2440      2450      2460      2470
                   |--------+---------+---------+---------+---------+---------+---------+---------+---------+---------+---------+---------+---------|
   BnaA.GLN1.3.a   ACCAGTCCCGGTTAGTGTCTCTAATCTCTAACCACACTCTTCTTGTGCAATAAGTTGTAACATTGTTGCGTTGGTTCAGGGTGATTGGAACGGAGCTGGAGCTCACTGCAACTACAGCACGAAGTCAATG
mRNA.BnaA.GLN1.3.a ACCAGTCCCGG---------------------------------------------------------------------GTGATTGGAACGGAGCTGGAGCTCACTGCAACTACAGCACGAAGTCAATG
    BnaGln1.3_C2   ACCAGTCCCGG---------------------------------------------------------------------GTGATTGGAACGGAGCTGGAGCTCACTGCAACTACAGCACGAAGTCAATG
       Consensus   ACCAGTCCCGG.....................................................................GTGATTGGAACGGAGCTGGAGCTCACTGCAACTACAGCACGAAGTCAATG

                   2471 2480      2490      2500      2510      2520      2530      2540      2550      2560      2570      2580      2590      2600
                   |--------+---------+---------+---------+---------+---------+---------+---------+---------+---------+---------+---------+---------|
   BnaA.GLN1.3.a   AGGAACGACGGAGGATTAGCTGTGATTAAGAAAGCGATAGAGAAGCTTCAGGTGAAGCACAAGGAGCACATTGCTGCTTACGGTGAAGGCAACGAGCGTCGTCTCACGGGGAAGCACGAAACTGCAGACA
mRNA.BnaA.GLN1.3.a AGGAACGACGGAGGATTAGCTGTGATTAAGAAAGCGATAGAGAAGCTTCAGGTGAAGCACAAGGAGCACATTGCTGCTTACGGTGAAGGCAACGAGCGTCGTCTCACGGGGAAGCACGAAACTGCAGACA
    BnaGln1.3_C2   AGGAACGACGGAGGATTAGCTGTGATTAAGAAAGCGATAGAGAAGCTTCAGGTGAAGCACAAGGAGCACATTGCTGCTTACGGTGAAGGCAACGAGCGTCGTCTCACGGGGAAGCACGAAACTGCAGACA
       Consensus   AGGAACGACGGAGGATTAGCTGTGATTAAGAAAGCGATAGAGAAGCTTCAGGTGAAGCACAAGGAGCACATTGCTGCTTACGGTGAAGGCAACGAGCGTCGTCTCACGGGGAAGCACGAAACTGCAGACA

                   2601 2610      2620      2630      2640      2650      2660      2670      2680      2690      2700      2710      2720      2730
                   |--------+---------+---------+---------+---------+---------+---------+---------+---------+---------+---------+---------+---------|
   BnaA.GLN1.3.a   TCAACACGTTCTCTTGGGGAGTGGCGAACCGTGGGGCTTCGGTGAGAGTGGGGAGAGACACTGAGAAAGAAGGCAAAGGTTACTTCGAGGACAGAAGGCCAGCTTCTAACATGGATCCTTACGTTGTTAC
mRNA.BnaA.GLN1.3.a TCAACACGTTCTCTTGGGGAGTGGCGAACCGTGGGGCTTCGGTGAGAGTGGGGAGAGACACTGAGAAAGAAGGCAAAGGTTACTTCGAGGACAGAAGGCCAGCTTCTAACATGGATCCTTACGTTGTTAC
    BnaGln1.3_C2   TCAACACGTTCTCTTGGGGAGTGGCGAACCGTGGGGCTTCGGTGAGAGTGGGGAGAGACACTGAGAAAGAAGGCAAAGGTTACTTCGAGGACAGAAGGCCAGCTTCTAACATGGATCCTTACGTTGTTAC
       Consensus   TCAACACGTTCTCTTGGGGAGTGGCGAACCGTGGGGCTTCGGTGAGAGTGGGGAGAGACACTGAGAAAGAAGGCAAAGGTTACTTCGAGGACAGAAGGCCAGCTTCTAACATGGATCCTTACGTTGTTAC

                   2731 2740      2750      2760      2770      2780      2790      2800      2810      2820      2830      2840      2850      2860
                   |--------+---------+---------+---------+---------+---------+---------+---------+---------+---------+---------+---------+---------|
   BnaA.GLN1.3.a   GTCCATGATCGCTGAAACCACCATCCTCGGTTAAACCACACACATTTAGTAATATTTGATTTTCTCCGGTTTGGTTTTATGATTTGAATACTGTTGTACTTGTGATCGCGGTCTATTTCGGTTTCAATAA
mRNA.BnaA.GLN1.3.a GTCCATGATCGCTGAAACCACCATCCTCGGTTAAACCACACACATTTAGTAATATTTGATTTTCTCCGGTTTGGTTTTATGATTTGAATACTGTTGTACTTGTGATCGCGGTCTATTTCGGTTTCAATAA
    BnaGln1.3_C2   GTCCATGATCGCTGAAACCACCATCCTCGGTTAAACCACACACATTTAGTAATATTTGATTTTCTCCGGTTTGGTTTTATGATTTGAATACTGTTGTACTTGTGATCGCGGTCTATTTCGGTTTCAATAA
       Consensus   GTCCATGATCGCTGAAACCACCATCCTCGGTTAAACCACACACATTTAGTAATATTTGATTTTCTCCGGTTTGGTTTTATGATTTGAATACTGTTGTACTTGTGATCGCGGTCTATTTCGGTTTCAATAA

                   2861 2870      2880      2890      2900      2910      2920      2930    2938
                   |--------+---------+---------+---------+---------+---------+-------|
   BnaA.GLN1.3.a   TTCTTATGACGACATTTTGTGTTTTTTCTTCTTCTTAGTTTCGAATAATTAAATAAGGGTATTCATGCGGTGAAAAAA
mRNA.BnaA.GLN1.3.a TTCTTATGACGACATTTTGTGTTTTTTCTTCTTCTTAGTTTCGAATAATTAAATAAGGGTATTCATGCGGTGAAAAAA
    BnaGln1.3_C2   TTCTTATGACGAAAA----------------------------AAAAAAAAAAAAA
       Consensus   TTCTTATGACGAcAttttgtgttttttcttcttcttagtttcgAAtAAttAAAtAAgggtattcatgcggtgaaaaaa
```

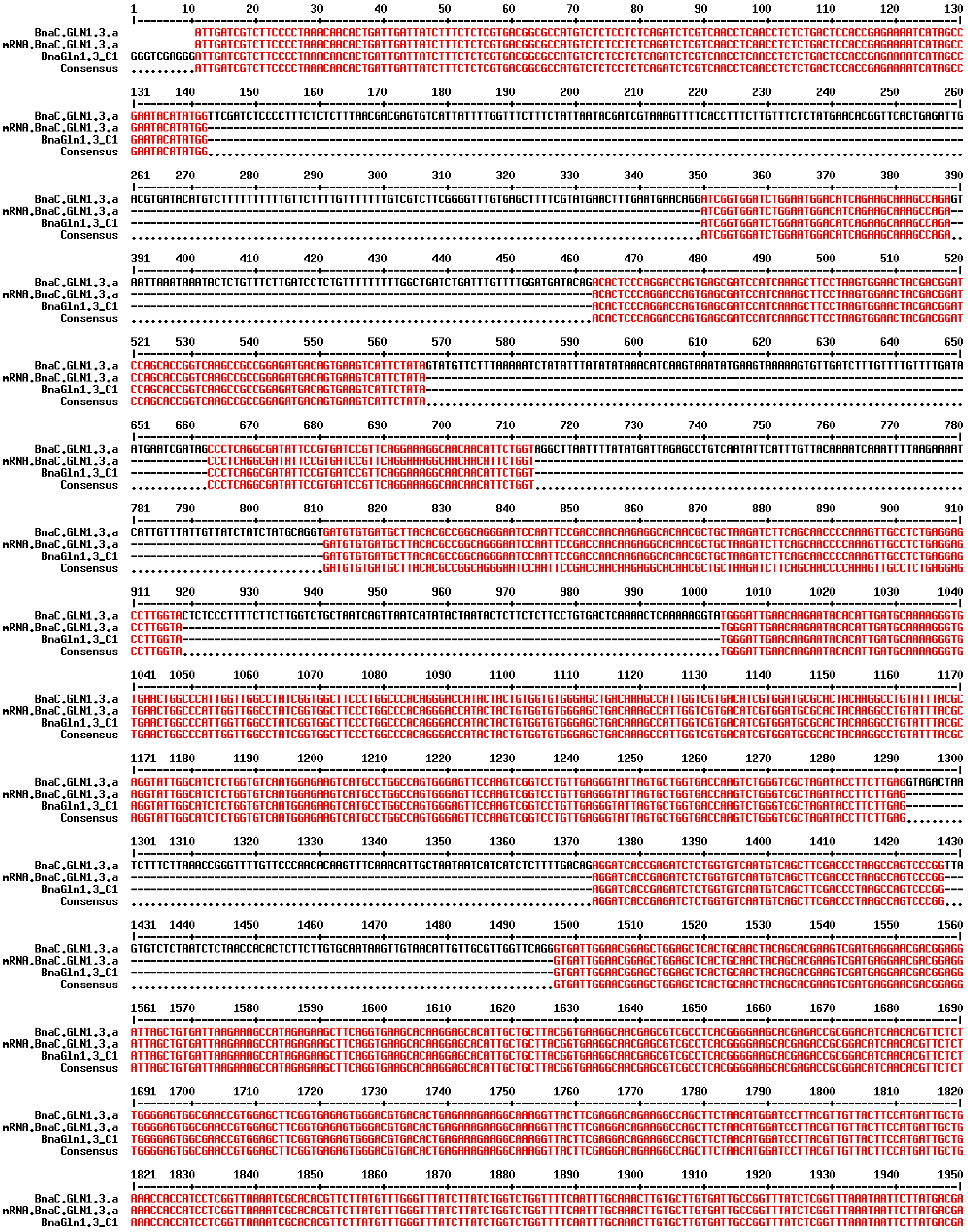
BnaC.GLN1.3.a
mRNA.BnaC.GLN1.3.a
BnaGln1.3_C1
Consensus

```
       Consensus  AAACCACCATCCTCGGTTAAAATCGCACACGTTCTTATGTTTGGGTTTATCTTATCTGGTCTGGTTTTCAATTTGCAAACTTGTGCTTGTGATTGCCGGTTTATCTCGGTTTAAATAATTCTTATGACGA

                  1951  1960      1970      1980      1990      2000      2010      2020      2030      2040      2050      2060      2070      2080
                  |--------+---------+---------+---------+---------+---------+---------+---------+---------+---------+---------+---------+---------|
   BnaC.GLN1.3.a  CATTTTGTATTTTTTTTTCTTCCTAGTTTCGAA-TAATTAAATAAGGTATTCATATGGTGAAAAAGTCTATCAGCGACTTTTGTGTTGTTGTTTTGAAATGATAATAATAATAACAAAGTACGGCCTGGC
mRNA.BnaC.GLN1.3.a  CATTTTGTATTTTTTTTTCTTCCTAGTTTCGAA-TAATTAAATAAGGTATTCATATGGTGAAAAAGTCTATCAGCGACTTTTGTGTTGTTGTTTTGAAATGATAATAATAATAACAAAGTACGGCCTGGC
    BnaGln1.3_C1  CATTTTGTATTTTTTTTTCTTCCTAGTTTCGAAATAATTAAATAAGGTATCCAAATCGCCCTATAG------------TGAGTCGTATTACAATTCAACTGGCCGTCGTTTTTACAACGTCGTGACTGGG
       Consensus  CATTTTGTATTTTTTTTTCTTCCTAGTTTCGAA.TAATTAAATAAGGTATtCAtATgGtgaaAaAagtctatcagcgacTtttgtGTtgTtgttTTgAAaTGataaTaaTaaTaACAAaGTacgGcCTGGc

                  2081  2090      2100      2110      2120      2130      2140      2150      2160      2170      2180      2190      2200      2210
                  |--------+---------+---------+---------+---------+---------+---------+---------+---------+---------+---------+---------+---------|
   BnaC.GLN1.3.a  G---CGTTGGAGGTTAATGAGCTTGTCGGGAGTAGTAAAGTAGGTGGATAAGATCTGGTGATTAATAATTATGAATTTATCTTAAAATTTAAATGTTGAATGTTCTTTACAACATTATGCAGGATGTGGC
mRNA.BnaC.GLN1.3.a  G---CGTTGGAGGTTAATGAGCTTGTCGGGAGTAGTAAAGTAGGTGGATAAGATCTGGTGATTAATAATTATGAATTTATCTTAAAATTTAAATGTTGAATGTTCTTTACAACATTATGCAGGATGTGGC
    BnaGln1.3_C1  AAAACCCCTGCGGTTACCCACCTT--------------------------------------------------------------------------AAATCGCCTTGCAGCACAATTCCCCTTTTCGC
       Consensus  g...CgttgGaGGTTAatgAgCTTgtcgggagtagtaaagtaggtggataagatctggtgattaataattatgaatttatcttaaaatttaaatgttgAAtgttCtTTaCAaCAttATgCaggaTgTgGC

                  2211  2220      2230      2240      2250      2260      2270      228082
                  |--------+---------+---------+---------+---------+---------+---------+-|
   BnaC.GLN1.3.a  ATGTTACAGAACATCTCCAATGTATACGTAGAAATATCATATTTATATATTTTTTTCTATAAATAAA
mRNA.BnaC.GLN1.3.a  ATGTTACAGAACATCTCCAATGTATACGTAGAAATATCATATTTATATATTTTTTTCTATAAATAAA
    BnaGln1.3_C1  AGCTTGGCGTAATTAACGAAAAGGCCCGGAACCGATGGCCCTTTTCCAACGTTGCGCCAGCCTGAATGGCCA
       Consensus  AtgTTacaGaAcaTctCcAAtgtataCGtAgaaatatcataTTTatatAttTTtttCtAtaaatAAa.....
```

file:///C:/Users/Camille/Desktop/Supp%20Figure%20alignements/C3a.gif[25/09/2013 13:29:05]

```
                  1       10        20        30        40        50        60        70        80        90        100       110       120       130
                  |--------+---------+---------+---------+---------+---------+---------+---------+---------+---------+---------+---------+---------|
     BnaA.GLN1.3.b TGAATGGAACTTGAGATTGTTGATTGTTGGATTAGATAAGTTTAAAGATAAATTCAATAGAAAAAGAATAGAGGTTACATGATTGTGTCGAACTGTTGGGTAATGCTAGGTTTTATTTCATTGTCTAAGT
mRNA.BnaA.GLN1.3.b
      BnaGln1.3_C4
         Consensus ..................................................................................................................................

                  131     140       150       160       170       180       190       200       210       220       230       240       250       260
                  |--------+---------+---------+---------+---------+---------+---------+---------+---------+---------+---------+---------+---------|
     BnaA.GLN1.3.b GGTGATTAAAGTTTCATATTAGGATATTTCATTGTCTTAGTTATTTGTTTGATTTAATTTTATTTGTCTCTTTCTTTTTAGATTTATAAGTATTAAATGTAACTTCTACCAAATCTACAAAATCACTCCA
mRNA.BnaA.GLN1.3.b
      BnaGln1.3_C4
         Consensus ..................................................................................................................................

                  261     270       280       290       300       310       320       330       340       350       360       370       380       390
                  |--------+---------+---------+---------+---------+---------+---------+---------+---------+---------+---------+---------+---------|
     BnaA.GLN1.3.b CAATCATGATTATATATTTTGCAGATCATTGAATAAGAATAGAATTAAAAAAATACAATCCTTACTATTTTTTCTCATGAACAAAACTATAGATTTTTAGTGATTCTTGTGAATGGACTATTTTTGTCTT
mRNA.BnaA.GLN1.3.b
      BnaGln1.3_C4
         Consensus ..................................................................................................................................

                  391     400       410       420       430       440       450       460       470       480       490       500       510       520
                  |--------+---------+---------+---------+---------+---------+---------+---------+---------+---------+---------+---------+---------|
     BnaA.GLN1.3.b TTGTATTAGATGTTAATAAAAAAAAAACAAAAAGTATGATATATGTTCTAATTCGTAATTGAATGTATATTTAAAACTAATAACATACTAAAAACCATTTTTCGCATATTCTCTGAGTTTCTTGATTTTC
mRNA.BnaA.GLN1.3.b
      BnaGln1.3_C4
         Consensus ..................................................................................................................................

                  521     530       540       550       560       570       580       590       600       610       620       630       640       650
                  |--------+---------+---------+---------+---------+---------+---------+---------+---------+---------+---------+---------+---------|
     BnaA.GLN1.3.b AAGTAATTATGTAGGCTTGGAGATCACTGCATGAATAGTGAGTAGCAAAATTTTGAACAATGACCATATGTAAATAGTCCACTCCCTTTCTTGTTTTAGGTTTTACGCCTCTTTCATGGTGATATCTTTT
mRNA.BnaA.GLN1.3.b
      BnaGln1.3_C4
         Consensus ..................................................................................................................................

                  651     660       670       680       690       700       710       720       730       740       750       760       770       780
                  |--------+---------+---------+---------+---------+---------+---------+---------+---------+---------+---------+---------+---------|
     BnaA.GLN1.3.b TTTGCTTTTCCATCATGGGTTTGCATTTCTTTAAGAGCTTCAAATGTAAAAAAAAGAAGCTAATTGTGCTTCTTTCTTTTATCAATTTTAGATCAGTACAATGTCTAGGCTAGTTGAAATAGATGTTAAT
mRNA.BnaA.GLN1.3.b
      BnaGln1.3_C4
         Consensus ..................................................................................................................................

                  781     790       800       810       820       830       840       850       860       870       880       890       900       910
                  |--------+---------+---------+---------+---------+---------+---------+---------+---------+---------+---------+---------+---------|
     BnaA.GLN1.3.b CTATTGAAGAAATTAAATGGGAAAAGGGTATGACATTGTTCACGTTAGATTCTTGTTTCTTTCATTTCTCAGTTACTTAACACAATTTCTTATGAATGTTTATAAACAAGTGACATTAAAGCCATTAGTT
mRNA.BnaA.GLN1.3.b
      BnaGln1.3_C4
         Consensus ..................................................................................................................................

                  911     920       930       940       950       960       970       980       990       1000      1010      1020      1030      1040
                  |--------+---------+---------+---------+---------+---------+---------+---------+---------+---------+---------+---------+---------|
     BnaA.GLN1.3.b TTGCTAGTAGGTTAACTTCTAGTCTAGTTTAACATGTAGATGGACACGGACGATCGATAAAATGAGCAGCACCGAGACATGATGTTCCGACATGATGAATGAAACAGTATTTGATTTCACCTGTTTGAAT
mRNA.BnaA.GLN1.3.b                                                     ATCGATAAAATGAGCAGCACCGAGACATGATGTTCCGACATGATGAATGAAACAGTATTTGATTTCACCTGTTTGAAT
      BnaGln1.3_C4                              CCACGCGTCCGCGGACGCGTGGGAT----------------------------------------------------------------------------
         Consensus ...............................ac..gt....ggac.cg...gATcgataaaatgagcagcaccgagacatgatgttccgacatgatgaatgaaacagtatttgatttcacctgtttgaat

                  1041 1050      1060      1070      1080      1090      1100      1110      1120      1130      1140      1150      1160      1170
                  |--------+---------+---------+---------+---------+---------+---------+---------+---------+---------+---------+---------+---------|
     BnaA.GLN1.3.b TGCATCACTTTGAGTTTGTTTTTAAGTATTGGTGTGCAAAAATGACAAGATTTTGTCACACTACTAAAAAAAAGTTTAGCTGTATACATCATGTACATGTATGTTGCATTTTCATGACTATTGGCGTTGC
mRNA.BnaA.GLN1.3.b TGCATCACTTTGAGTTTGTTTTTAAGTATTGGTGTGCAAAAATGACAAGATTTTGTCACACTACTAAAAAAAAGTTTAGCTGTATACATCATGTACATGTATGTTGCATTTTCATGACTATTGGCGTTGC
      BnaGln1.3_C4 ----------------------------------------------------------------------------------------------------------------------------------
         Consensus tgcatcactttgagtttgtttttaagtattggtgtgcaaaaatgacaagattttgtcacactactaaaaaaaagtttagctgtatacatcatgtacatgtatgttgcattttcatgactattggcgttgc

                  1171 1180      1190      1200      1210      1220      1230      1240      1250      1260      1270      1280      1290      1300
                  |--------+---------+---------+---------+---------+---------+---------+---------+---------+---------+---------+---------+---------|
     BnaA.GLN1.3.b TAGAAAAAAGAACTTGCTTTCAAAATTAACAAATACGTGATGATTGAATCCAACTTAGCATGATAAAAAGAATCATTATGTACGCAAAGACAAAAATATCCAAGACTCGATTAATACAAATCTCGTTTCG
mRNA.BnaA.GLN1.3.b TAGAAAAAAGAACTTGCTTTCAAAATTAACAAATACGTGATGATTGAATCCAACTTAGCATGATAAAAAGAATCATTATGTACGCAAAGACAAAAATATCCAAGACTCGATTAATACAAATCTCGTTTCG
      BnaGln1.3_C4 ----------------------------------------------------------------------------------------------------------------------------------
         Consensus tagaaaaaagaacttgctttcaaaattaacaaatacgtgatgattgaatccaacttagcatgataaaaagaatcattatgtacgcaaagacaaaaatatccaagactcgattaatacaaatctcgtttcg

                  1301 1310      1320      1330      1340      1350      1360      1370      1380      1390      1400      1410      1420      1430
                  |--------+---------+---------+---------+---------+---------+---------+---------+---------+---------+---------+---------+---------|
     BnaA.GLN1.3.b TTTATTTACCTAAGTAGTTTTCGTGTTCATCAACAATTCTATTTATACCAATTTAATCATAGATTACATATATTTTAATATTGTTGTCATAATCTGTATCTAAATCACTGGAGAATCTTACCTGAAACAA
mRNA.BnaA.GLN1.3.b TTTATTTACCTAAGTAGTTTTCGTGTTCATCAACAATTCTATTTATACCAATTTAATCATAGATTACATATATTTTAATATTGTTGTCATAATCTGTATCTAAATCACTGGAGAATCTTACCTGAAACAA
      BnaGln1.3_C4 ----------------------------------------------------------------------------------------------------------------------------------
         Consensus tttatttacctaagtagttttcgtgttcatcaacaattctatttataccaatttaatcatagattacatatattttaatattgttgtcataatctgtatctaaatcactggagaatcttacctgaaacaa

                  1431 1440      1450      1460      1470      1480      1490      1500      1510      1520      1530      1540      1550      1560
                  |--------+---------+---------+---------+---------+---------+---------+---------+---------+---------+---------+---------+---------|
     BnaA.GLN1.3.b TATAACAATATTAAATAATTGTTTAAAATTCTAAAAGATAATTATTGACACACAACAATAAATAATCACCAATTTACTAGAATCATGTATTGACCACCGTAAATATCTGAAAATTTAAAAAAAAAAACAA
mRNA.BnaA.GLN1.3.b TATAACAATATTAAATAATTGTTTAAAATTCTAAAAGATAATTATTGACACACAACAATAAATAATCACCAATTTACTAGAATCATGTATTGACCACCGTAAATATCTGAAAATTTAAAAAAAAAAACAA
      BnaGln1.3_C4 ----------------------------------------------------------------------------------------------------------------------------------
         Consensus tataacaatattaaataattgtttaaaattctaaaagataattattgacacacaacaataaataatcaccaatttactagaatcatgtattgaccaccgtaaatatctgaaaatttaaaaaaaaaaacaa

                  1561 1570      1580      1590      1600      1610      1620      1630      1640      1650      1660      1670      1680      1690
                  |--------+---------+---------+---------+---------+---------+---------+---------+---------+---------+---------+---------+---------|
     BnaA.GLN1.3.b AAACAAAAATAGCTCTATAAACACTCGAATAAGTTTTAATGATCGTCTTCCCTAAACAACACTGATTAATTTTAGTATTTTTTTTCTCTCGTATTCTCCTCTCAGACGCAGCCATGTCTCTGCTCTCAGA
mRNA.BnaA.GLN1.3.b AAACAAAAATAGCTCTATAAACACTCGAATAAGTTTTAATGATCGTCTTCCCTAAACAACACTGATTAATTTTAGTATTTTTTTTCTCTCGTATTCTCCTCTCAGACGCAGCCATGTCTCTGCTCTCAGA
      BnaGln1.3_C4 ---------------------------------------------------------------------TTTAGTATTTTTTTTTCTCTCGTATTCTCCTCTCAGACGCAGCCATGTCTCTGCTCTCAGA
         Consensus aaacaaaaatagctctataaacactcgaataagttttaatgatcgtcttccctaaacaacactgattaaTTTtagtaTTTTTTTTCTCTCGTATTCTCCTCTCAGACGCAGCCATGTCTCTGCTCTCAGA

                  1691 1700      1710      1720      1730      1740      1750      1760      1770      1780      1790      1800      1810      1820
                  |--------+---------+---------+---------+---------+---------+---------+---------+---------+---------+---------+---------+---------|
     BnaA.GLN1.3.b TCTCGTCAACCTCAACCTCTCCGACACAACCAAGCAAATCATCGCCGAATACATATGGTTTGATCTACCTTCATCTCTGTTTATCTCTCACGACAATTCTCATTATCTCTGTTCTTACTATTGCTACGTT
mRNA.BnaA.GLN1.3.b TCTCGTCAACCTCAACCTCTCCGACACAACCAAGCAAATCATCGCCGAATACATATGG------------------------------------------------------------------------
      BnaGln1.3_C4 TCTCGTCAACCTCAACCTCTCCGACACAACCAAGCAAATCATCGCCGAATACATATGG------------------------------------------------------------------------
         Consensus TCTCGTCAACCTCAACCTCTCCGACACAACCAAGCAAATCATCGCCGAATACATATGG........................................................................

                  1821 1830      1840      1850      1860      1870      1880      1890      1900      1910      1920      1930      1940      1950
                  |--------+---------+---------+---------+---------+---------+---------+---------+---------+---------+---------+---------+---------|
     BnaA.GLN1.3.b CAGAGAGCTTTGTCTTTTATATTTTTTTTTTGGTACCGATCTTTTATGTGGTTGGTTTGAACACGATTCGTTTCACTCGGTTAACGCTGTCATGCCATGCGGCGATGAGATTGACGTGTACAAACATAAA
mRNA.BnaA.GLN1.3.b ----------------------------------------------------------------------------------------------------------------------------------
      BnaGln1.3_C4 ----------------------------------------------------------------------------------------------------------------------------------
```

```
      Consensus  ..................................................................................................................................

                 1951 1960      1970      1980      1990      2000      2010      2020      2030      2040      2050      2060      2070      2080
   BnaA.GLN1.3.b GCTTGTCTCTTTTTTCTTTGTCGTCTTCTGGGTTTGTGATGTTTTTCGTGTGTATTGTGAATGAACAGGATCGGTGGATCTGGAATGGACATTAGAAGCAAAGCCAGAGTAAGTCGATACTCTGTTTTCT
mRNA.BnaA.GLN1.3.b ---------------------------------------------------------------------ATCGGTGGATCTGGAATGGACATTAGAAGCAAAGCCAGA----------------------
     BnaGln1.3_C4 ---------------------------------------------------------------------ATCGGTGGATCTGGAATGGACATTAGAAGCAAAGCCAGA----------------------
      Consensus  .....................................................................ATCGGTGGATCTGGAATGGACATTAGAAGCAAAGCCAGA......................

                 2081 2090      2100      2110      2120      2130      2140      2150      2160      2170      2180      2190      2200      2210
   BnaA.GLN1.3.b TGCTCTGTTTTTACTTTTTACTCTGTTTCTTGTATGTTTGATCTGTTTTTGTTTTGGTTTATATAGACACTACCAGGACCAGTGACCGATCCATCAAAGCTTCCCAAGTGGAACTACGACGGATCTAGCA
mRNA.BnaA.GLN1.3.b ------------------------------------------------------------------ACACTACCAGGACCAGTGACCGATCCATCAAAGCTTCCCAAGTGGAACTACGACGGATCTAGCA
     BnaGln1.3_C4 ------------------------------------------------------------------ACACTACCAGGACCAGTGACCGATCCATCAAAGCTTCCCAAGTGGAACTACGACGGATCTAGCA
      Consensus  ..................................................................ACACTACCAGGACCAGTGACCGATCCATCAAAGCTTCCCAAGTGGAACTACGACGGATCTAGCA

                 2211 2220      2230      2240      2250      2260      2270      2280      2290      2300      2310      2320      2330      2340
   BnaA.GLN1.3.b CCGGTCAGGCCGCTGGAGATGACAGTGAAGTCATTCTATAGTATGTTCTTCCATCATCAGCAAGTAATATGAAATAAAAACAGATCTTATCTTAATCTTGATCTTGTTTTGTTTCGTTTATAGTCCTCAG
mRNA.BnaA.GLN1.3.b CCGGTCAGGCCGCTGGAGATGACAGTGAAGTCATTCTATA-----------------------------------------------------------------------------------TCCTCAG
     BnaGln1.3_C4 CCGGTCAGGCCGCTGGAGATGACAGTGAAGTCATTCTATA-----------------------------------------------------------------------------------TCCTCAG
      Consensus  CCGGTCAGGCCGCTGGAGATGACAGTGAAGTCATTCTATA...................................................................................TCCTCAG

                 2341 2350      2360      2370      2380      2390      2400      2410      2420      2430      2440      2450      2460      2470
   BnaA.GLN1.3.b GCTATATTCCGTGATCCGTTCAGGAGAGGCAACAACATTCTGGTGATGTGTGATGCTTACACGCCGGCCGGTAATCCAATTCCGACCAACAAGAGGCACAACGCTGCAAAGATCTTCAGCAACTCAAAAG
mRNA.BnaA.GLN1.3.b GCTATATTCCGTGATCCGTTCAGGAGAGGCAACAACATTCTGGTGATGTGTGATGCTTACACGCCGGCCGGTAATCCAATTCCGACCAACAAGAGGCACAACGCTGCAAAGATCTTCAGCAACTCAAAAG
     BnaGln1.3_C4 GCTATATTCCGTGATCCGTTCAGGAGAGGCAACAACATTCTGGTGATGTGTGATGCTTACACGCCGGCCGGTAATCCAATTCCGACCAACAAGAGGCACAACGCTGCAAAGATCTTCAGCAACTCAAAAG
      Consensus  GCTATATTCCGTGATCCGTTCAGGAGAGGCAACAACATTCTGGTGATGTGTGATGCTTACACGCCGGCCGGTAATCCAATTCCGACCAACAAGAGGCACAACGCTGCAAAGATCTTCAGCAACTCAAAAG

                 2471 2480      2490      2500      2510      2520      2530      2540      2550      2560      2570      2580      2590      2600
   BnaA.GLN1.3.b TTGCCTCTGAGGAGCCTTGGTACTAACTCTCCTCTTCTTCTTATTGGTCTATGTCACTCTCTGCTTAATCATTTACTTATATACTAAAACTCTTGAAAAGGTATGGGATTGAGCAAGAATACACATTGAT
mRNA.BnaA.GLN1.3.b TTGCCTCTGAGGAGCCTTGGTA---------------------------------------------------------------------------------TGGGATTGAGCAAGAATACACATTGAT
     BnaGln1.3_C4 TTGCCTCTGAGGAGCCTTGGTA---------------------------------------------------------------------------------TGGGATTGAGCAAGAATACACATTGAT
      Consensus  TTGCCTCTGAGGAGCCTTGGTA.................................................................................TGGGATTGAGCAAGAATACACATTGAT

                 2601 2610      2620      2630      2640      2650      2660      2670      2680      2690      2700      2710      2720      2730
   BnaA.GLN1.3.b GCAAAAGGGTGTGAATTGGCCCATTGGTTGGCCTGTTGGTGGCTTCCCTGGCCCACAGGGACCGTACTACTGTGGCGTGGGAGCTGACAAAGCCATTGGTCGTGACATCGTGGATGCACACTACAAAGCC
mRNA.BnaA.GLN1.3.b GCAAAAGGGTGTGAATTGGCCCATTGGTTGGCCTGTTGGTGGCTTCCCTGGCCCACAGGGACCGTACTACTGTGGCGTGGGAGCTGACAAAGCCATTGGTCGTGACATCGTGGATGCACACTACAAAGCC
     BnaGln1.3_C4 GCAAAAGGGTGTGAATTGGCCCATTGGTTGGCCTGTTGGTGGCTTCCCTGGCCCACAGGGACCGTACTACTGTGGCGTGGGAGCTGACAAAGCCATTGGTCGTGACATCGTGGATGCACACTACAAAGCC
      Consensus  GCAAAAGGGTGTGAATTGGCCCATTGGTTGGCCTGTTGGTGGCTTCCCTGGCCCACAGGGACCGTACTACTGTGGCGTGGGAGCTGACAAAGCCATTGGTCGTGACATCGTGGATGCACACTACAAAGCC

                 2731 2740      2750      2760      2770      2780      2790      2800      2810      2820      2830      2840      2850      2860
   BnaA.GLN1.3.b TGTCTTTACGCAGGTATTAGCATCTCTGGTGTCAATGGAGAAGTCATGCCTGGCCAGTGGGAGTTCCAAGTCGGTCCTGTTGAAGGGATTAGTGCCGGTGATCAAGTCTGGATAGCTAGATTCCTTCTCG
mRNA.BnaA.GLN1.3.b TGTCTTTACGCAGGTATTAGCATCTCTGGTGTCAATGGAGAAGTCATGCCTGGCCAGTGGGAGTTCCAAGTCGGTCCTGTTGAAGGGATTAGTGCCGGTGATCAAGTCTGGATAGCTAGATTCCTTCTCG
     BnaGln1.3_C4 TGTCTTTACGCAGGTATTAGCATCTCTGGTGTCAATGGAGAAGTCATGCCTGGCCAGTGGGAGTTCCAAGTCGGTCCTGTTGAAGGGATTAGTGCCGGTGATCAAGTCTGGATAGCTAGATTCCTTCTCG
      Consensus  TGTCTTTACGCAGGTATTAGCATCTCTGGTGTCAATGGAGAAGTCATGCCTGGCCAGTGGGAGTTCCAAGTCGGTCCTGTTGAAGGGATTAGTGCCGGTGATCAAGTCTGGATAGCTAGATTCCTTCTCG

                 2861 2870      2880      2890      2900      2910      2920      2930      2940      2950      2960      2970      2980      2990
   BnaA.GLN1.3.b AGGTAATAATCTACTTAACCAGTTCCGTTCACATTACATGCTTCAAAATTTTATTTTATTTTTAAGCATCAAAATTTTGCTCATGGGTCATCTTTTCTTTTGACAGAGGATCACTGAGATCTCTGGTGTA
mRNA.BnaA.GLN1.3.b AG--------------------------------------------------------------------------------------------------------AGGATCACTGAGATCTCTGGTGTA
     BnaGln1.3_C4 AG--------------------------------------------------------------------------------------------------------AGGATCACTGAGATCTCTGGTGTA
      Consensus  AG........................................................................................................AGGATCACTGAGATCTCTGGTGTA

                 2991 3000      3010      3020      3030      3040      3050      3060      3070      3080      3090      3100      3110      3120
   BnaA.GLN1.3.b AACGTCAGCTTCGACCCAAAACCAGTCCCGGTTAGTGTCTCTCTAGTGCTCTTTATGTGCAATAACTAGAGTGTTGTTGTAACTTTTTTTGTTATTTTTGCTAAATTATATTCTATTAAAATAGTTTTTT
mRNA.BnaA.GLN1.3.b AACGTCAGCTTCGACCCAAAACCAGTCCCGG---------------------------------------------------------------------------------------------------
     BnaGln1.3_C4 AACGTCAGCTTCGACCCAAAACCAGTCCCGG---------------------------------------------------------------------------------------------------
      Consensus  AACGTCAGCTTCGACCCAAAACCAGTCCCGG...................................................................................................

                 3121 3130      3140      3150      3160      3170      3180      3190      3200      3210      3220      3230      3240      3250
   BnaA.GLN1.3.b TTTTTTTTGCTAAAATTTGGTTCTATTAAAATTGTTGTAACTTGTAACGTATTGTTGTATGGGTTTCAGGGTGATTGGAACGGAGCGGGAGCGCACTGCAACTACAGTACGAAGACGATGAGGAACACTG
mRNA.BnaA.GLN1.3.b ----------------------------------------------------------------------GTGATTGGAACGGAGCGGGAGCGCACTGCAACTACAGTACGAAGACGATGAGGAACACTG
     BnaGln1.3_C4 ----------------------------------------------------------------------GTGATTGGAACGGAGCGGGAGCGCACTGCAACTACAGTACGAAGACGATGAGGAACACTG
      Consensus  ......................................................................GTGATTGGAACGGAGCGGGAGCGCACTGCAACTACAGTACGAAGACGATGAGGAACACTG

                 3251 3260      3270      3280      3290      3300      3310      3320      3330      3340      3350      3360      3370      3380
   BnaA.GLN1.3.b GAGGATTAGCGGTGATAAAGAAAGCGATAGAGAAGCTTCAGGTGAAGCACAAGCAGCACATTGCTGCTTACGGTGAAGGCAACGAGCGTCGTCTCACGGGCAAGCACGAGACGGCAGATATCAACACGTT
mRNA.BnaA.GLN1.3.b GAGGATTAGCGGTGATAAAGAAAGCGATAGAGAAGCTTCAGGTGAAGCACAAGCAGCACATTGCTGCTTACGGTGAAGGCAACGAGCGTCGTCTCACGGGCAAGCACGAGACGGCAGATATCAACACGTT
     BnaGln1.3_C4 GAGGATTAGCGGTGATAAAGAAAGCGATAGAGAAGCTTCAGGTGAAGCACAAGCAGCACATTGCTGCTTACGGTGAAGGCAACGAGCGTCGTCTCACGGGCAAGCACGAGACGGCAGATATCAACACGTT
      Consensus  GAGGATTAGCGGTGATAAAGAAAGCGATAGAGAAGCTTCAGGTGAAGCACAAGCAGCACATTGCTGCTTACGGTGAAGGCAACGAGCGTCGTCTCACGGGCAAGCACGAGACGGCAGATATCAACACGTT

                 3381 3390      3400      3410      3420      3430      3440      3450      3460      3470      3480      3490      3500      3510
   BnaA.GLN1.3.b CTCATGGGGAGTGGCGAACCGTGGAGCTTCGGTGAGAGTGGGACGTGACACTGAGAAAGAAGGCAAAGGTTACTTCGAGGATCGTAGGCCAGCTTCTAACATGGATCCTTATGTCGTCACTTCCATGATC
mRNA.BnaA.GLN1.3.b CTCATGGGGAGTGGCGAACCGTGGAGCTTCGGTGAGAGTGGGACGTGACACTGAGAAAGAAGGCAAAGGTTACTTCGAGGATCGTAGGCCAGCTTCTAACATGGATCCTTATGTCGTCACTTCCATGATC
     BnaGln1.3_C4 CTCATGGGGAGTGGCGAACCGTGGAGCTTCGGTGAGAGTGGGACGTGACACTGAGAAAGAAGGCAAAGGTTACTTCGAGGATCGTAGGCCAGCTTCTAACATGGATCCTTATGTCGTCACTTCCATGATC
      Consensus  CTCATGGGGAGTGGCGAACCGTGGAGCTTCGGTGAGAGTGGGACGTGACACTGAGAAAGAAGGCAAAGGTTACTTCGAGGATCGTAGGCCAGCTTCTAACATGGATCCTTATGTCGTCACTTCCATGATC

                 3511 3520      3530      3540      3550      3560      3570      3580      3590      3600      3610      3620      3630      3640
   BnaA.GLN1.3.b GCCGAAACCACAATCCTCGGTTAAGTAATCAATACGCATTTTAATGTTTAGTTTTTTCCTTAATTTGCGGATTTTTGTGCTTGTGGTTGCACTACTAATAATTATTATCATGCCCTAGTAGGATTCACGT
mRNA.BnaA.GLN1.3.b GCCGAAACCACAATCCTCGGTTAAGTAATCAATACGCATTTTAATGTTTAGTTTTTTCCTTAATTTGCGGATTTTTGTGCTTGTGGTTGCACTACTAATAATTATTATCATGCCCTAGTAGGATTCACGT
     BnaGln1.3_C4 GCCGAAACCACAATCCTCGGTTAAGTAATCAATACGCATTTTAATGTTTAGTTTTTTCCTTAATTTGCGGATTTTTGTGCTTGTGGTTGCACTACTAATAATTATTATCATGCCCTAGTAGGATTCACGT
      Consensus  GCCGAAACCACAATCCTCGGTTAAGTAATCAATACGCATTTTAATGTTTAGTTTTTTCCTTAATTTGCGGATTTTTGTGCTTGTGGTTGCACTACTAATAATTATTATCATGCCCTAGTAGGATTCACGT

                 3641 3650      3660      3670      3680      3690      3700      3710      3720      3730      3740      3750      3760      3770
   BnaA.GLN1.3.b GTTTATGTTTTTTTTTTTTCATTTCGAATAATTAAATAAGGAATTCTTATGGATGAAATTTAAACAAAAGAATGTATTATTAGTGATTTTTTTTGTTGATGTGGTTGAACTTGTTAGGGATAAGATTTTG
mRNA.BnaA.GLN1.3.b GTTTATGTTTTTTTTTTTTCATTTCGAATAATTAAATAAGGAATTCTTATGGATGAAATTTAAACAAAAGAATGTATTATTAGTGATTTTTTTTGTTGATGTGGTTGAACTTGTTAGGGATAAGATTTTG
     BnaGln1.3_C4 GTTTATGTTTTTTTTTTTTCATTTCGAATAATT
      Consensus  GTTTATGTTTTTTTTTTTTCATTTCGAATAATTaaataaggaattcttatggatgaaatttaaacaaaagaatgtattattagtgattttttttgttgatgtggttgaacttgttagggataagattttg

                 3771 3780      3790      3800      3809
   BnaA.GLN1.3.b TGATTATTGATAACATAAGAAATTAATAAGAAATAAAAA
mRNA.BnaA.GLN1.3.b TGATTATTGATAACATAAGAAATTAATAAGAAATAAAAA
```

file:///C:/Users/Camille/Desktop/Supp%20Figure%20alignements/A3b.gif[25/09/2013 13:27:18]

```
[illegible]  [illegible]
BnaGln1.3_C4
   Consensus  tgattattgataacataagaaattaataagaaataaaaa
```

file:///C:/Users/Camille/Desktop/Supp%20Figure%20alignements/A3b.gif[25/09/2013 13:27:18]

```
                  1       10        20        30        40        50        60        70        80        90        100       110       120      130
                  |--------+---------+---------+---------+---------+---------+---------+---------+---------+---------+---------+---------+---------|
  BnaC.GLN1.3.b   TCTAACAATGATTAATCTTTACTACCTTAAACCAATGAAAACAAATTTTAAACTATATAGTTTATTTTAAAAATTAAACAAAAACTAAATGTTTAATTATTTACTCGATAATATAAATCTATGAAGGGAA
  mRNA.BnaC.GLN1.3.b
  BnaGln1.3_C3
  Consensus       ..................................................................................................................................

                  131     140       150       160       170       180       190       200       210       220       230       240       250      260
                  |--------+---------+---------+---------+---------+---------+---------+---------+---------+---------+---------+---------+---------|
  BnaC.GLN1.3.b   AAGTTTAATTTCTTAAAAACTTTCTAAATTTGTGAAATGTTACAATATCTTTGAATATGAGAATAAAACAATATTTTACTAATCTTTATATATATAGTTACGATTTTAATAATGAAATAATAATCCGAAA
  mRNA.BnaC.GLN1.3.b
  BnaGln1.3_C3
  Consensus       ..................................................................................................................................

                  261     270       280       290       300       310       320       330       340       350       360       370       380      390
                  |--------+---------+---------+---------+---------+---------+---------+---------+---------+---------+---------+---------+---------|
  BnaC.GLN1.3.b   ATATATATATATAAGAAAATACAAATACATGTGAAAGTTTGAAACAATCTATTCAAAGGAAAAAAATATACCGTAAACTTATTTTGTTTTAAAAATTGATAGACACATATATATTATAATATATACCAAT
  mRNA.BnaC.GLN1.3.b
  BnaGln1.3_C3
  Consensus       ..................................................................................................................................

                  391     400       410       420       430       440       450       460       470       480       490       500       510      520
                  |--------+---------+---------+---------+---------+---------+---------+---------+---------+---------+---------+---------+---------|
  BnaC.GLN1.3.b   TTAGAATTGAAAACAAAATATTTATATAAAAATAAATGAAAATAAATGAAAACAAAAATCTGCGCGGTTGCGCGGATCGAGATCTAGTTTCTTTTATTTCTCAATTGCTTAACACTATTTTATGAATGTT
  mRNA.BnaC.GLN1.3.b
  BnaGln1.3_C3
  Consensus       ..................................................................................................................................

                  521     530       540       550       560       570       580       590       600       610       620       630       640      650
                  |--------+---------+---------+---------+---------+---------+---------+---------+---------+---------+---------+---------+---------|
  BnaC.GLN1.3.b   TATAAACAAGTGACATTGAAGCCACTAGTTTTGCTAGTATGTTAACTTCTAACCTAGTTTAACATGTAGATGGGGACAAGGACAATCGATAAAGTGGGCAGCTCCGAGGCATAATTTTTTGACACGATGA
  mRNA.BnaC.GLN1.3.b
  BnaGln1.3_C3
  Consensus       ..................................................................................................................................

                  651     660       670       680       690       700       710       720       730       740       750       760       770      780
                  |--------+---------+---------+---------+---------+---------+---------+---------+---------+---------+---------+---------+---------|
  BnaC.GLN1.3.b   ATGAATGAAACAGTATTTGATTTCACCTGTTTGAATTGCATCACTTTGAGTTTGTTTTTAAGTATTGGTGTGTATAAATGACAAGATTTTGTCACACTACTAAAAAAAAGTTTTATCCGTATACATCAGA
  mRNA.BnaC.GLN1.3.b
  BnaGln1.3_C3
  Consensus       ..................................................................................................................................

                  781     790       800       810       820       830       840       850       860       870       880       890       900      910
                  |--------+---------+---------+---------+---------+---------+---------+---------+---------+---------+---------+---------+---------|
  BnaC.GLN1.3.b   TATATCTATCTTATATACATGTATATTGCATTTTCATGAATATTGGCGTTGGCTTGAAAAATAAAAACTTGGTTTCAAAATCAACAAATACGAGATGATTGAATCCAACTTAACATGATAGAGTCATTAT
  mRNA.BnaC.GLN1.3.b                                                   GCGTTGGCTTGAAAAATAAAAACTTGGTTTCAAAATCAACAAATACGAGATGATTGAATCCAACTTAACATGATAGAGTCATTAT
  BnaGln1.3_C3
  Consensus       .................................................gcgttggcttgaaaaataaaaacttggtttcaaaatcaacaaatacgagatgattgaatccaacttaacatgatagagtcattat

                  911     920       930       940       950       960       970       980       990       1000      1010      1020      1030    1040
                  |--------+---------+---------+---------+---------+---------+---------+---------+---------+---------+---------+---------+---------|
  BnaC.GLN1.3.b   GTACGCAAAAGACAAAAATGCCAAGACTCGATTCATCATCACAGGCTCACAGTGATGATACCTATATAGGCAAGTAATACAAAACTCGTTTCGTTTAGTTACTTGACTATTTACGTGTTCATAAACAATT
  mRNA.BnaC.GLN1.3.b   GTACGCAAAAGACAAAAATGCCAAGACTCGATTCATCATCACAGGCTCACAGTGATGATACCTATATAGGCAAGTAATACAAAACTCGTTTCGTTTAGTTACTTGACTATTTACGTGTTCATAAACAATT
  BnaGln1.3_C3
  Consensus       gtacgcaaaagacaaaaatgccaagactcgattcatcatcacaggctcacagtgatgatacctatataggcaagtaatacaaaactcgtttcgtttagttacttgactatttacgtgttcataaacaatt

                  1041  1050      1060      1070      1080      1090      1100      1110      1120      1130      1140      1150      1160    1170
                  |--------+---------+---------+---------+---------+---------+---------+---------+---------+---------+---------+---------+---------|
  BnaC.GLN1.3.b   CTATTTAACTGATTAAATCATTGATTACATATATGTAAATATTGTTGTCATAATCTGTATTTAAATCACAGGAGATCTTAACTGAAACAATATAATAATACAAATTAATTATTTAAAATTCTAAAAGATA
  mRNA.BnaC.GLN1.3.b   CTATTTAACTGATTAAATCATTGATTACATATATGTAAATATTGTTGTCATAATCTGTATTTAAATCACAGGAGATCTTAACTGAAACAATATAATAATACAAATTAATTATTTAAAATTCTAAAAGATA
  BnaGln1.3_C3
  Consensus       ctatttaactgattaaatcattgattacatatatgtaaatattgttgtcataatctgtatttaaatcacaggagatcttaactgaaacaatataataatacaaattaattatttaaaattctaaaagata

                  1171  1180      1190      1200      1210      1220      1230      1240      1250      1260      1270      1280      1290    1300
                  |--------+---------+---------+---------+---------+---------+---------+---------+---------+---------+---------+---------+---------|
  BnaC.GLN1.3.b   AATATTAGTATCACACAACAATAAAGAAATTACCAATTTACTAGAATCATGTATTGACCACCGTAAATATCTGAAAACAATAATAAATAAATAAACAAAAATAGCTCTATAAACACTCAGAGAAGTTTTA
  mRNA.BnaC.GLN1.3.b   AATATTAGTATCACACAACAATAAAGAAATTACCAATTTACTAGAATCATGTATTGACCACCGTAAATATCTGAAAACAATAATAAATAAATAAACAAAAATAGCTCTATAAACACTCAGAGAAGTTTTA
  BnaGln1.3_C3
  Consensus       aatattagtatcacacaacaataaagaaattaccaatttactagaatcatgtattgaccaccgtaaatatctgaaaacaataataaataaataaacaaaaatagctctataaacactcagagaagtttta

                  1301  1310      1320      1330      1340      1350      1360      1370      1380      1390      1400      1410      1420    1430
                  |--------+---------+---------+---------+---------+---------+---------+---------+---------+---------+---------+---------+---------|
  BnaC.GLN1.3.b   ATGATCGTCTTCCCTAAACAGCACTGATTGATTAGTGTATTTTTTTCTCTCGTATTCTCCTCTCAGACGCAGCCATGTCTCTGCTCTCAGATCTCGTCAACCTCAACCTCTCCGACACCACCAAGCAAAT
  mRNA.BnaC.GLN1.3.b   ATGATCGTCTTCCCTAAACAGCACTGATTGATTAGTGTATTTTTTTCTCTCGTATTCTCCTCTCAGACGCAGCCATGTCTCTGCTCTCAGATCTCGTCAACCTCAACCTCTCCGACACCACCAAGCAAAT
  BnaGln1.3_C3                                                    TCTCGTATTCTCCTCTCAGACGCAGCCATGTCTCTGCTCTCAGATCTCGTCAACCTCAACCTCTCCGACACCACCAAGCAAAT
  Consensus       atgatcgtcttccctaaacagcactgattgattagtgtatttttttcTCTCGTATTCTCCTCTCAGACGCAGCCATGTCTCTGCTCTCAGATCTCGTCAACCTCAACCTCTCCGACACCACCAAGCAAAT

                  1431  1440      1450      1460      1470      1480      1490      1500      1510      1520      1530      1540      1550    1560
                  |--------+---------+---------+---------+---------+---------+---------+---------+---------+---------+---------+---------+---------|
  BnaC.GLN1.3.b   CATCGCCGAATACATATGGTTTGATCTCCTTTCCTCTCACGACAACTCTCATTATCTCTGTTCTTAATATTGCTACGGTCAGAAAAATTTGCTTTTTATGTTGTTTTGGTTTGAATATGATTCGTTTCAC
  mRNA.BnaC.GLN1.3.b   CATCGCCGAATACATATGG---------------------------------------------------------------------------------------------------------------
  BnaGln1.3_C3    CATCGCCGAATACATATGG---------------------------------------------------------------------------------------------------------------
  Consensus       CATCGCCGAATACATATGG...............................................................................................................

                  1561  1570      1580      1590      1600      1610      1620      1630      1640      1650      1660      1670      1680    1690
                  |--------+---------+---------+---------+---------+---------+---------+---------+---------+---------+---------+---------+---------|
  BnaC.GLN1.3.b   TCGGTTAACGCTGTCATGTCATGCCATGCGGCGATGAAATTAACGTGTACAAACATAAAGCTCGTCTCTTTTTTCTTTGTCGTCTTCTGGGTTTGTGATGTTTTTCGTGTGTATTGTGAATGAACAGGAT
  mRNA.BnaC.GLN1.3.b   --------------------------------------------------------------------------------------------------------------------------------AT
  BnaGln1.3_C3    --------------------------------------------------------------------------------------------------------------------------------AT
  Consensus       ................................................................................................................................AT

                  1691  1700      1710      1720      1730      1740      1750      1760      1770      1780      1790      1800      1810    1820
                  |--------+---------+---------+---------+---------+---------+---------+---------+---------+---------+---------+---------+---------|
  BnaC.GLN1.3.b   CGGTGGATCTGGAATGGACATTAGAAGCAAAGCCAGGGTAAGTCGATACTCTGTTTTTTTTACTCTGTTTCTTGTATGGTTGATCTGTTTTTGTTTTGGTTTATACAGACACTCCCAGGACCAGTGACCG
  mRNA.BnaC.GLN1.3.b   CGGTGGATCTGGAATGGACATTAGAAGCAAAGCCAGG-----------------------------------------------------------------------ACACTCCCAGGACCAGTGACCG
  BnaGln1.3_C3    CGGTGGATCTGGAATGGACATTAGAAGCAAAGCCAGG-----------------------------------------------------------------------ACACTCCCAGGACCAGTGACCG
  Consensus       CGGTGGATCTGGAATGGACATTAGAAGCAAAGCCAGG.......................................................................ACACTCCCAGGACCAGTGACCG

                  1821  1830      1840      1850      1860      1870      1880      1890      1900      1910      1920      1930      1940    1950
                  |--------+---------+---------+---------+---------+---------+---------+---------+---------+---------+---------+---------+---------|
  BnaC.GLN1.3.b   ATCCATCAAAGCTTCCCAAGTGGAACTACGACGGATCAAGCACCGGTCAGGCCGCTGGAGATGACAGTGAAGTCATTCTATAGTATGTTCTTCCATCATGTCTATATACAGCAAGTAATATGAAATAAAA
  mRNA.BnaC.GLN1.3.b   ATCCATCAAAGCTTCCCAAGTGGAACTACGACGGATCAAGCACCGGTCAGGCCGCTGGAGATGACAGTGAAGTCATTCTATA------------------------------------------------
  BnaGln1.3_C3    ATCCATCAAAGCTTCCCAAGTGGAACTACGACGGATCAAGCACCGGTCAGGCCGCTGGAGATGACAGTGAAGTCATTCTATA------------------------------------------------
```

file:///C:/Users/Camille/Desktop/Supp%20Figure%20alignements/C3b.gif[25/09/2013 13:29:18]

```
       Consensus  ATCCATCAAAGCTTCCCAAGTGGAACTACGACGGATCAAGCACCGGTCAGGCCGCTGGAGATGACAGTGAAGTCATTCTATA................................................

                  1951 1960      1970      1980      1990      2000      2010      2020      2030      2040      2050      2060      2070      2080
                  |--------+---------+---------+---------+---------+---------+---------+---------+---------+---------+---------+---------+---------|
    BnaC.GLN1.3.b ACATATCTTATCTTAATCTTGATTTTGTTTCGTTTATAGTCCTCAGGCTATATTCCGTGATCCGTTCAGGAGAGGCAACAACATTCTGGTGATGTGTGATGCTTACACGCCGGCCGGTAATCCAATTCCG
mRNA.BnaC.GLN1.3.b ---------------------------------------TCCTCAGGCTATATTCCGTGATCCGTTCAGGAGAGGCAACAACATTCTGGTGATGTGTGATGCTTACACGCCGGCCGGTAATCCAATTCCG
     BnaGln1.3_C3 ---------------------------------------TCCTCAGGCTATATTCCGTGATCCGTTCAGGAGAGGCAACAACATTCTGGTGATGTGTGATGCTTACACGCCGGCCGGTAATCCAATTCCG
        Consensus .......................................TCCTCAGGCTATATTCCGTGATCCGTTCAGGAGAGGCAACAACATTCTGGTGATGTGTGATGCTTACACGCCGGCCGGTAATCCAATTCCG

                  2081 2090      2100      2110      2120      2130      2140      2150      2160      2170      2180      2190      2200      2210
                  |--------+---------+---------+---------+---------+---------+---------+---------+---------+---------+---------+---------+---------|
    BnaC.GLN1.3.b ACCAACAAGAGGCATAACGCTGCTAAGATCTTCAGCAACTCTAAAGTTGCCTCTGAGGAGCCTTGGTATTAACTCTCCTCTTCTTCTTATTGGTCTATTCTCTGCTTAATCATTTAATTATATACTAAAA
mRNA.BnaC.GLN1.3.b ACCAACAAGAGGCATAACGCTGCTAAGATCTTCAGCAACTCTAAAGTTGCCTCTGAGGAGCCTTGGTAT-------------------------------------------------------------
     BnaGln1.3_C3 ACCAACAAGAGGCATAACGCTGCTAAGATCTTCAGCAACTCTAAAGTTGCCTCTGAGGAGCCTTGGTAT-------------------------------------------------------------
        Consensus ACCAACAAGAGGCATAACGCTGCTAAGATCTTCAGCAACTCTAAAGTTGCCTCTGAGGAGCCTTGGTAT.............................................................

                  2211 2220      2230      2240      2250      2260      2270      2280      2290      2300      2310      2320      2330      2340
                  |--------+---------+---------+---------+---------+---------+---------+---------+---------+---------+---------+---------+---------|
    BnaC.GLN1.3.b CTCTTGAAAAGGTATGGGATTGAGCAAGAATACACATTGATGCAAAAGGGTGTGAACTGGCCTATTGGTTGGCCTGTTGGTGGCTTCCCTGGCCCACAGGGACCATACTACTGTGGTGTGGGAGCTGACA
mRNA.BnaC.GLN1.3.b ---------------GGGATTGAGCAAGAATACACATTGATGCAAAAGGGTGTGAACTGGCCTATTGGTTGGCCTGTTGGTGGCTTCCCTGGCCCACAGGGACCATACTACTGTGGTGTGGGAGCTGACA
     BnaGln1.3_C3 ---------------GGGATTGAGCAAGAATACACATTGATGCAAAAGGGTGTGAACTGGCCTATTGGTTGGCCTGTTGGTGGCTTCCCTGGCCCACAGGGACCATACTACTGTGGTGTGGGAGCTGACA
        Consensus ...............GGGATTGAGCAAGAATACACATTGATGCAAAAGGGTGTGAACTGGCCTATTGGTTGGCCTGTTGGTGGCTTCCCTGGCCCACAGGGACCATACTACTGTGGTGTGGGAGCTGACA

                  2341 2350      2360      2370      2380      2390      2400      2410      2420      2430      2440      2450      2460      2470
                  |--------+---------+---------+---------+---------+---------+---------+---------+---------+---------+---------+---------+---------|
    BnaC.GLN1.3.b AAGCCATTGGTCGTGACATCGTGGATGCACACTACAAAGCCTGTCTTTACGCAGGTATTAGCATCTCTGGTGTCAATGGAGAAGTCATGCCTGGCCAGTGGGAGTTCCAAGTCGGTCCTGTTGAAGGTAT
mRNA.BnaC.GLN1.3.b AAGCCATTGGTCGTGACATCGTGGATGCACACTACAAAGCCTGTCTTTACGCAGGTATTAGCATCTCTGGTGTCAATGGAGAAGTCATGCCTGGCCAGTGGGAGTTCCAAGTCGGTCCTGTTGAAGGTAT
     BnaGln1.3_C3 AAGCCATTGGTCGTGACATCGTGGATGCACACTACAAAGCCTGTCTTTACGCAGGTATTAGCATCTCTGGTGTCAATGGAGAAGTCATGCCTGGCCAGTGGGAGTTCCAAGTCGGTCCTGTTGAAGGTAT
        Consensus AAGCCATTGGTCGTGACATCGTGGATGCACACTACAAAGCCTGTCTTTACGCAGGTATTAGCATCTCTGGTGTCAATGGAGAAGTCATGCCTGGCCAGTGGGAGTTCCAAGTCGGTCCTGTTGAAGGTAT

                  2471 2480      2490      2500      2510      2520      2530      2540      2550      2560      2570      2580      2590      2600
                  |--------+---------+---------+---------+---------+---------+---------+---------+---------+---------+---------+---------+---------|
    BnaC.GLN1.3.b TAGTGCTGGTGATCAAGTCTGGATCGCTAGATTCCTTCTCGAGGTAGTAATCTACGTCTGCTTAACCAGTTCTGTTCACATTACAAGCTTCAAAATTTTATTTTATTTTTAATTTTATTTAATTTTTAAG
mRNA.BnaC.GLN1.3.b TAGTGCTGGTGATCAAGTCTGGATCGCTAGATTCCTTCTCGAG---------------------------------------------------------------------------------------
     BnaGln1.3_C3 TAGTGCTGGTGATCAAGTCTGGATCGCTAGATTCCTTCTCGAG---------------------------------------------------------------------------------------
        Consensus TAGTGCTGGTGATCAAGTCTGGATCGCTAGATTCCTTCTCGAG.......................................................................................

                  2601 2610      2620      2630      2640      2650      2660      2670      2680      2690      2700      2710      2720      2730
                  |--------+---------+---------+---------+---------+---------+---------+---------+---------+---------+---------+---------+---------|
    BnaC.GLN1.3.b CATCAAAATTTTGCTAATGGGTCAGGGTCATCTTTTCTTTTGACAGAGGATCACTGAGATCTCTGGTGTAAACGTCAGCTTCGACCCAAAACCAGTCCCGGTTAGTGTCTCTCTAATGCTCTTTATGTGC
mRNA.BnaC.GLN1.3.b ----------------------------------------------AGGATCACTGAGATCTCTGGTGTAAACGTCAGCTTCGACCCAAAACCAGTCCCGG-----------------------------
     BnaGln1.3_C3 ----------------------------------------------AGGATCACTGAGATCTCTGGTGTAAACGTCAGCTTCGACCCAAAACCAGTCCCGG-----------------------------
        Consensus ..............................................AGGATCACTGAGATCTCTGGTGTAAACGTCAGCTTCGACCCAAAACCAGTCCCGG.............................

                  2731 2740      2750      2760      2770      2780      2790      2800      2810      2820      2830      2840      2850      2860
                  |--------+---------+---------+---------+---------+---------+---------+---------+---------+---------+---------+---------+---------|
    BnaC.GLN1.3.b AATAAATAGTGTTGTTGTAACTTTTTCTTTTTTTTGCTAAATTATACTCCTTCTTTTTCCGTTATAAGTGTCGTTTTAGTTTTTGACCCACGGATAAGGAAACAATTAATTTTGTATTTTTCCTATAAAA
mRNA.BnaC.GLN1.3.b ----------------------------------------------------------------------------------------------------------------------------------
     BnaGln1.3_C3 ----------------------------------------------------------------------------------------------------------------------------------
        Consensus ..................................................................................................................................

                  2861 2870      2880      2890      2900      2910      2920      2930      2940      2950      2960      2970      2980      2990
                  |--------+---------+---------+---------+---------+---------+---------+---------+---------+---------+---------+---------+---------|
    BnaC.GLN1.3.b AAAACACTATTACCTATCCACCTAACCATATTTCAACTAATAGGAAAATAAATTTTGCATAAAATTAATAAATTTTGCATTTAAAATCGAAAACGACACTTATTTTGTAAGGAAAAAATTTCTCTAAAAC
mRNA.BnaC.GLN1.3.b ----------------------------------------------------------------------------------------------------------------------------------
     BnaGln1.3_C3 ----------------------------------------------------------------------------------------------------------------------------------
        Consensus ..................................................................................................................................

                  2991 3000      3010      3020      3030      3040      3050      3060      3070      3080      3090      3100      3110      3120
                  |--------+---------+---------+---------+---------+---------+---------+---------+---------+---------+---------+---------+---------|
    BnaC.GLN1.3.b GACACTTAATATGAAACGGAGGGTGTATTCTATTAAAATTGTTGTAACTTGTAACGTTTTTTTTTGTAACGTATTGTTGCATGGGTTTCAGGGTGATTGGAACGGAGCTGGAGCTCACTGTAACTACAGC
mRNA.BnaC.GLN1.3.b --------------------------------------------------------------------------------------------GTGATTGGAACGGAGCTGGAGCTCACTGTAACTACAGC
     BnaGln1.3_C3 --------------------------------------------------------------------------------------------GTGATTGGAACGGAGCTGGAGCTCACTGTAACTACAGC
        Consensus ............................................................................................GTGATTGGAACGGAGCTGGAGCTCACTGTAACTACAGC

                  3121 3130      3140      3150      3160      3170      3180      3190      3200      3210      3220      3230      3240      3250
                  |--------+---------+---------+---------+---------+---------+---------+---------+---------+---------+---------+---------+---------|
    BnaC.GLN1.3.b ACGAAGACGATGAGGAACACTGGAGGACTAGCGGTGATAAAGAAAGCGATAGAGAAGCTTCAGGTGAAGCACAAGCAGCACATTGCTGCTTACGGTGAAGGCAACGAGCGTCGTCTCACGGGGAAGCACG
mRNA.BnaC.GLN1.3.b ACGAAGACGATGAGGAACACTGGAGGACTAGCGGTGATAAAGAAAGCGATAGAGAAGCTTCAGGTGAAGCACAAGCAGCACATTGCTGCTTACGGTGAAGGCAACGAGCGTCGTCTCACGGGGAAGCACG
     BnaGln1.3_C3 ACGAAGACGATGAGGAACACTGGAGGACTAGCGGTGATAAAGAAAGCGATAGAGAAGCTTCAGGTGAAGCACAAGCAGCACATTGCTGCTTACGGTGAAGGCAACGAGCGTCGTCTCACGGGGAAGCACG
        Consensus ACGAAGACGATGAGGAACACTGGAGGACTAGCGGTGATAAAGAAAGCGATAGAGAAGCTTCAGGTGAAGCACAAGCAGCACATTGCTGCTTACGGTGAAGGCAACGAGCGTCGTCTCACGGGGAAGCACG

                  3251 3260      3270      3280      3290      3300      3310      3320      3330      3340      3350      3360      3370      3380
                  |--------+---------+---------+---------+---------+---------+---------+---------+---------+---------+---------+---------+---------|
    BnaC.GLN1.3.b AGACGGCAGATATCAACACGTTCTCGTGGGGAGTGGCGAACCGTGGAGCATCGGTGAGAGTGGGACGTGACACTGAGAAAGAAGGCAAAGGTTACTTCGAGGATCGTAGGCCAGCTTCTAACATGGATCC
mRNA.BnaC.GLN1.3.b AGACGGCAGATATCAACACGTTCTCGTGGGGAGTGGCGAACCGTGGAGCATCGGTGAGAGTGGGACGTGACACTGAGAAAGAAGGCAAAGGTTACTTCGAGGATCGTAGGCCAGCTTCTAACATGGATCC
     BnaGln1.3_C3 AGACGGCAGATATCAACACGTTCTCGTGGGGAGTGGCGAACCGTGGAGCATCGGTGAGAGTGGGACGTGACACTGAGAAAGAAGGCAAAGGTTACTTCGAGGATCGTAGGCCAGCTTCTAACATGGATCC
        Consensus AGACGGCAGATATCAACACGTTCTCGTGGGGAGTGGCGAACCGTGGAGCATCGGTGAGAGTGGGACGTGACACTGAGAAAGAAGGCAAAGGTTACTTCGAGGATCGTAGGCCAGCTTCTAACATGGATCC

                  3381 3390      3400      3410      3420      3430      3440      3450      3460      3470      3480      3490      3500      3510
                  |--------+---------+---------+---------+---------+---------+---------+---------+---------+---------+---------+---------+---------|
    BnaC.GLN1.3.b TTATGTCGTTACTTCCATGATCGCTGAAACCACAATCCTCGGTTAAGTAATCGCACGCATTTTATAATGTTTGGTTTATCCGGTTTCGTTTATTTCTTAATTTGCGGATTTTTGTGCTTGTGGTTGCACT
mRNA.BnaC.GLN1.3.b TTATGTCGTTACTTCCATGATCGCTGAAACCACAATCCTCGGTTAAGTAATCGCACGCATTTTATAATGTTTGGTTTATCCGGTTTCGTTTATTTCTTAATTTGCGGATTTTTGTGCTTGTGGTTGCACT
     BnaGln1.3_C3 TTATGTCGTTACTTCCATGATCGCTGAAACCACAATCCTCGGTTAAGTAATCGCACGCATTTTATAATGTTTGGTTTATCCGGTTTCGTTTATTTCTTAATTTGCGGATTTTTGTGCTTGTGGTTGCACT
        Consensus TTATGTCGTTACTTCCATGATCGCTGAAACCACAATCCTCGGTTAAGTAATCGCACGCATTTTATAATGTTTGGTTTATCCGGTTTCGTTTATTTCTTAATTTGCGGATTTTTGTGCTTGTGGTTGCACT

                  3511 3520      3530      3540      3550      3560      3570      3580      3590      3600      3610 3615
                  |--------+---------+---------+---------+---------+---------+---------+---------+---------+---------+----|
    BnaC.GLN1.3.b ACTAATAATTATTATCATGCCCTAGTAGGATTCACGTGTTTTATGTTTTTTTTTCATTTCGAATAATTAAATAAGGAATTCTTATGGATGAAATTTAAACAAAAA
mRNA.BnaC.GLN1.3.b ACTAATAATTATTATCATGCCCTAGTAGGATTCACGTGTTTTATGTTTTTTTTTCATTTCGAATAATTAAATAAGGAATTCTTATGGATGAAATTTAAACAAAAA
     BnaGln1.3_C3 ACTAATAATTATTATCATGCCCTAGTAGGATTCACGTGTTTTATGTTTTTTTTTCATTTCGAATAATTAAATAAGGAATTCTTATGGATGAAATTTAAA
        Consensus ACTAATAATTATTATCATGCCCTAGTAGGATTCACGTGTTTTATGTTTTTTTTTCATTTCGAATAATTAAATAAGGAATTCTTATGGATGAAATTTAAAcaaaaa
```

file:///C:/Users/Camille/Desktop/Supp%20Figure%20alignements/C3b.gif[25/09/2013 13:29:18]

```
                 1       10        20        30        40        50        60        70        80        90        100       110       120       130
                 |--------+---------+---------+---------+---------+---------+---------+---------+---------+---------+---------+---------+---------|
   BnaA.GLN1.3.c TATATATAAACACTCTCAGGAGAGAAGCTGTATCGAGATCGTCTTCCCAAAACAACACTCATTGATTGATTACTATCCGACGCAGCCATGTCTCTGCTCTCAGATCTCGTCAACCTCAACCTCTCCGACT
mRNA.BnaA.GLN1.3.c TATATATAAACACTCTCAGGAGAGAAGCTGTATCGAGATCGTCTTCCCAAAACAACACTCATTGATTGATTACTATCCGACGCAGCCATGTCTCTGCTCTCAGATCTCGTCAACCTCAACCTCTCCGACT
    BnaGln1.3_C5       GGGTCGACGGCCATTACGGCCGGGGATCGAGATCGTCTTCCCAAAACAACACTCATTGATTGATTACTATCCGACGCAGCCATGTCTCTGCTCTCAGATCTCGTCAACCTCAACCTCTCCGACT
       Consensus tatatataaaCactctCaggagaGaagctGtATCGAGATCGTCTTCCCAAAACAACACTCATTGATTGATTACTATCCGACGCAGCCATGTCTCTGCTCTCAGATCTCGTCAACCTCAACCTCTCCGACT

                 131  140       150       160       170       180       190       200       210       220       230       240       250       260
                 |--------+---------+---------+---------+---------+---------+---------+---------+---------+---------+---------+---------+---------|
   BnaA.GLN1.3.c CCACCAAGCAAATCATCGCCGAATACATATGGTTCCAACATCTCCCTTCTCTCTGTTTCTCTCACCACGAGTCTCATTATCTCTGTTTTCTTATCTTCGTTATCTCTGTTTGTTACTATTGCTGCGAT
mRNA.BnaA.GLN1.3.c CCACCAAGCAAATCATCGCCGAATACATATGG--------------------------------------------------------------------------------------------------
    BnaGln1.3_C5 CCACCAAGCAAATCATCGCCGAATACATATGG--------------------------------------------------------------------------------------------------
       Consensus CCACCAAGCAAATCATCGCCGAATACATATGG..................................................................................................

                 261  270       280       290       300       310       320       330       340       350       360       370       380       390
                 |--------+---------+---------+---------+---------+---------+---------+---------+---------+---------+---------+---------+---------|
   BnaA.GLN1.3.c CGAAACACTTTGATCTCTTATGTTTTGACAATTTTGGTTTGAACAAGATTCAGTTCACTCTGTTTTTAGCTGTCCTGACTCGATGAGATTGACGTGTACATCGTAAAGCTTGTCTCTTTCTTTGTTGTCG
mRNA.BnaA.GLN1.3.c ----------------------------------------------------------------------------------------------------------------------------------
    BnaGln1.3_C5 ----------------------------------------------------------------------------------------------------------------------------------
       Consensus ..................................................................................................................................

                 391  400       410       420       430       440       450       460       470       480       490       500       510       520
                 |--------+---------+---------+---------+---------+---------+---------+---------+---------+---------+---------+---------+---------|
   BnaA.GLN1.3.c TCTTGTGGGTTTGTAATTTTCTCGTATGAATGTTGAATGAACAGGATCGGTGGATCTGGCATGGACATTAGAAGCAAAGCCAGAGTAAATTTGATACTCTGTTTTTTTTCATTCCTCTCTGTTTCTTGTATG
mRNA.BnaA.GLN1.3.c ---------------------------------------------ATCGGTGGATCTGGCATGGACATTAGAAGCAAAGCCAGA----------------------------------------------
    BnaGln1.3_C5 ---------------------------------------------ATCGGTGGATCTGGCATGGACATTAGAAGCAAAGCCAGA----------------------------------------------
       Consensus .............................................ATCGGTGGATCTGGCATGGACATTAGAAGCAAAGCCAGA..............................................

                 521  530       540       550       560       570       580       590       600       610       620       630       640       650
                 |--------+---------+---------+---------+---------+---------+---------+---------+---------+---------+---------+---------+---------|
   BnaA.GLN1.3.c ATCGTTCTGATTTGCTAATGGATAATACAGACACTCCCAGGACCAGTGAGCGATCCATCAAAGCTTCCCAAGTGGAACTACGACGGATCCAGCACCGGTCAGGCCTCTGGAGATAACAGTGAAGTCATTC
mRNA.BnaA.GLN1.3.c ------------------------------ACACTCCCAGGACCAGTGAGCGATCCATCAAAGCTTCCCAAGTGGAACTACGACGGATCCAGCACCGGTCAGGCCTCTGGAGATAACAGTGAAGTCATTC
    BnaGln1.3_C5 ------------------------------ACACTCCCAGGACCAGTGAGCGATCCATCAAAGCTTCCCAAGTGGAACTACGACGGATCCAGCACCGGTCAGGCCTCTGGAGATAACAGTGAAGTCATTC
       Consensus ..............................ACACTCCCAGGACCAGTGAGCGATCCATCAAAGCTTCCCAAGTGGAACTACGACGGATCCAGCACCGGTCAGGCCTCTGGAGATAACAGTGAAGTCATTC

                 651  660       670       680       690       700       710       720       730       740       750       760       770       780
                 |--------+---------+---------+---------+---------+---------+---------+---------+---------+---------+---------+---------+---------|
   BnaA.GLN1.3.c TATAGTATGTTCCTCTTTATCTACTGTATAAGCATTAACACAAAATCTTGTCTTAATCTTCTTTTTTTGTTTTGTCTCGTTTTATGAATCGATAGCCCTCAGGCCATATTCCGTGATCCGTTCAGGAGAGG
mRNA.BnaA.GLN1.3.c TATA------------------------------------------------------------------------------------------CCCTCAGGCCATATTCCGTGATCCGTTCAGGAGAGG
    BnaGln1.3_C5 TATA------------------------------------------------------------------------------------------CCCTCAGGCCATATTCCGTGATCCGTTCAGGAGAGG
       Consensus TATA..........................................................................................CCCTCAGGCCATATTCCGTGATCCGTTCAGGAGAGG

                 781  790       800       810       820       830       840       850       860       870       880       890       900       910
                 |--------+---------+---------+---------+---------+---------+---------+---------+---------+---------+---------+---------+---------|
   BnaA.GLN1.3.c CGACAACATCCTGGTAGGGCTGATTTTTAAATGTTTTTTTTTTGTGCACAAAATTTAAAAATGATTAGTGTGACTAAATTCAATTTGGATCAGAATAAAAAATTGGTTAATGTGATATATTCAGGTGATG
mRNA.BnaA.GLN1.3.c CGACAACATCCTGGT---------------------------------------------------------------------------------------------------------------GATG
    BnaGln1.3_C5 CGACAACATCCTGGT---------------------------------------------------------------------------------------------------------------GATG
       Consensus CGACAACATCCTGGT...............................................................................................................GATG

                 911  920       930       940       950       960       970       980       990       1000      1010      1020      1030      1040
                 |--------+---------+---------+---------+---------+---------+---------+---------+---------+---------+---------+---------+---------|
   BnaA.GLN1.3.c TGTGATGCATACACACCGGCCGGAGATCCAATTCCGACAAACAAGAGGCACAAGGCTGCTAAGATCTTTAGCCATCCTAACGTTGCCAAGGAGGTGCCTTGGTAATCTCTTCTTCTTTGTATATTGATCT
mRNA.BnaA.GLN1.3.c TGTGATGCATACACACCGGCCGGAGATCCAATTCCGACAAACAAGAGGCACAAGGCTGCTAAGATCTTTAGCCATCCTAACGTTGCCAAGGAGGTGCCTTGGTA--------------------------
    BnaGln1.3_C5 TGTGATGCATACACACCGGCCGGAGATCCAATTCCGACAAACAAGAGGCACAAGGCTGCTAAGATCTTTAGCCATCCTAACGTTGCCAAGGAGGTGCCTTGGTA--------------------------
       Consensus TGTGATGCATACACACCGGCCGGAGATCCAATTCCGACAAACAAGAGGCACAAGGCTGCTAAGATCTTTAGCCATCCTAACGTTGCCAAGGAGGTGCCTTGGTA..........................

                 1041 1050      1060      1070      1080      1090      1100      1110      1120      1130      1140      1150      1160      1170
                 |--------+---------+---------+---------+---------+---------+---------+---------+---------+---------+---------+---------+---------|
   BnaA.GLN1.3.c CTCTGCAAATCAATCTTATATACTTATTCTCTCTCTCTCTCTGACTCAAAAAGGTATGGGATTGAGCAAGAATACACTTTGATGCAAAAGGGTGTGAACTGGCCTATTGGTTGGCCAATTGGTGGCTT
mRNA.BnaA.GLN1.3.c ----------------------------------------------------------TGGGATTGAGCAAGAATACACTTTGATGCAAAAGGGTGTGAACTGGCCTATTGGTTGGCCAATTGGTGGCTT
    BnaGln1.3_C5 ----------------------------------------------------------TGGGATTGAGCAAGAATACACTTTGATGCAAAAGGGTGTGAACTGGCCTATTGGTTGGCCAATTGGTGGCTT
       Consensus ..........................................................TGGGATTGAGCAAGAATACACTTTGATGCAAAAGGGTGTGAACTGGCCTATTGGTTGGCCAATTGGTGGCTT

                 1171 1180      1190      1200      1210      1220      1230      1240      1250      1260      1270      1280      1290      1300
                 |--------+---------+---------+---------+---------+---------+---------+---------+---------+---------+---------+---------+---------|
   BnaA.GLN1.3.c CCCTGGTCCTCAGGTAAATCTAAATGATCAACTGATCTTGATGTCATCACAACACTTTTTTTTTTTACTTAACTTTCTAATATTATGACGCAGGGACCATACTACTGTGGTGTGGGAGCTGACAAAGCCA
mRNA.BnaA.GLN1.3.c CCCTGGTCCTCAGG--------------------------------------------------------------------------------GACCATACTACTGTGGTGTGGGAGCTGACAAAGCCA
    BnaGln1.3_C5 CCCTGGTCCTCAGG--------------------------------------------------------------------------------GACCATACTACTGTGGTGTGGGAGCTGACAAAGCCA
       Consensus CCCTGGTCCTCAGG................................................................................GACCATACTACTGTGGTGTGGGAGCTGACAAAGCCA

                 1301 1310      1320      1330      1340      1350      1360      1370      1380      1390      1400      1410      1420      1430
                 |--------+---------+---------+---------+---------+---------+---------+---------+---------+---------+---------+---------+---------|
   BnaA.GLN1.3.c TTGGTCGTGACATTGTGGACGCACACTACAAGGCCTGTCTTTACGCAGGTATTGGCATCTCTGGTGTCAATGGAGAGGTCATGCCTGGACAGTGGGAGTTCCAAGTCGGTCCAGTTGAGGGTATTAGTTC
mRNA.BnaA.GLN1.3.c TTGGTCGTGACATTGTGGACGCACACTACAAGGCCTGTCTTTACGCAGGTATTGGCATCTCTGGTGTCAATGGAGAGGTCATGCCTGGACAGTGGGAGTTCCAAGTCGGTCCAGTTGAGGGTATTAGTTC
    BnaGln1.3_C5 TTGGTCGTGACATTGTGGACGCACACTACAAGGCCTGTCTTTACGCAGGTATTGGCATCTCTGGTGTCAATGGAGAGGTCATGCCTGGACAGTGGGAGTTCCAAGTCGGTCCAGTTGAGGGTATTAGTTC
       Consensus TTGGTCGTGACATTGTGGACGCACACTACAAGGCCTGTCTTTACGCAGGTATTGGCATCTCTGGTGTCAATGGAGAGGTCATGCCTGGACAGTGGGAGTTCCAAGTCGGTCCAGTTGAGGGTATTAGTTC

                 1431 1440      1450      1460      1470      1480      1490      1500      1510      1520      1530      1540      1550      1560
                 |--------+---------+---------+---------+---------+---------+---------+---------+---------+---------+---------+---------+---------|
   BnaA.GLN1.3.c TGGTGATCAAGTCTGGGTCGCTAGATACCTTCTTGAGGTAGAGTACTCTACTTTAACCGGTTCTATTGTGAATATCTCTACAAGATTTGCTAATGGCCATCTTATCTCTTTTGACAGAGGATCACTGAGA
mRNA.BnaA.GLN1.3.c TGGTGATCAAGTCTGGGTCGCTAGATACCTTCTTGAG--------------------------------------------------------------------------------AGGATCACTGAGA
    BnaGln1.3_C5 TGGTGATCAAGTCTGGGTCGCTAGATACCTTCTTGAG--------------------------------------------------------------------------------AGGATCACTGAGA
       Consensus TGGTGATCAAGTCTGGGTCGCTAGATACCTTCTTGAG................................................................................AGGATCACTGAGA

                 1561 1570      1580      1590      1600      1610      1620      1630      1640      1650      1660      1670      1680      1690
                 |--------+---------+---------+---------+---------+---------+---------+---------+---------+---------+---------+---------+---------|
   BnaA.GLN1.3.c TCTCTGGTGTAAATGTCAGCTTCGACCCAAAACCAGTCCCGGTTAGTGTCTCTTTACCACACTCTCTCAACACTCTCTTCATGTAAAATAAGTTGTAACAATGTTTGCATGGGTTGTTCAGGGTGATTGT
mRNA.BnaA.GLN1.3.c TCTCTGGTGTAAATGTCAGCTTCGACCCAAAACCAGTCCCGGTTAGTGTCTCTTTACCACACTCTCTCAACA----------------------------------------------------------
    BnaGln1.3_C5 TCTCTGGTGTAAATGTCAGCTTCGACCCTAAACCAGTTCCGGGTGATTGGAACGGAGCTGGAGCTCACTGCA----------------------------------------------------------
       Consensus TCTCTGGTGTAAATGTCAGCTTCGACCCaAAACCAGTcCCGGtTagTgtctctttAcCacactCTCtCaaCA..........................................................

                 1691 1700      1710      1720      1730      1740      1750      1760      1770      1780      1790      1800      1810      1820
                 |--------+---------+---------+---------+---------+---------+---------+---------+---------+---------+---------+---------+---------|
   BnaA.GLN1.3.c GCAACTACAGCACGAAGTCGATGAGGAACAACGGAGGATTAGCAGTGATAAAGAACGCGATAGAGAAGCTTCAGGTGAAGCACAAGGAGAACATTGCTGCGTACGGTGAAGGCAGCGAGCGTCGTCTCAC
mRNA.BnaA.GLN1.3.c ---ACTACAGCACGAAGTCGATGAGGAACAACGGAGGATTAGCAGTGATAAAGAACGCGATAGAGAAGCTTCAGGTGAAGCACAAGGAGAACATTGCTGCGTACGGTGAAGGCAGCGAGCGTCGTCTCAC
    BnaGln1.3_C5 ---ACTACAGTACGAAGACGATGAGGAACGACGGAGGATTAGCAGTGATAAAGAAAGCAATAGAGAAGCTTCAGGTGAAGCACAAGGAGCACATTGCTGCTTACGGTGAAGGCAACGAGCGTCGTCTCAC
       Consensus ...ACTACAGcACGAAGtCGATGAGGAACaACGGAGGATTAGCAGTGATAAAGAAcGCgATAGAGAAGCTTCAGGTGAAGCACAAGGAGaACATTGCTGCgTACGGTGAAGGCAgCGAGCGTCGTCTCAC

                 1821 1830      1840      1850      1860      1870      1880      1890      1900      1910      1920      1930      1940      1950
                 |--------+---------+---------+---------+---------+---------+---------+---------+---------+---------+---------+---------+---------|
   BnaA.GLN1.3.c GGGGAAGCACGAGACCGCATACATCAACACGTTCTCTTGGGGAGTGGCGAACCGTGGAGCTTCGGTGAGAGTGGGACGAGACACTGAGAAGGAAGGCAAAGGTTACTTCGAAGACAGAAGGCCAGCTTCT
mRNA.BnaA.GLN1.3.c GGGGAAGCACGAGACCGCATACATCAACACGTTCTCTTGGGGAGTGGCGAACCGTGGAGCTTCGGTGAGAGTGGGACGAGACACTGAGAAGGAAGGCAAAGGTTACTTCGAAGACAGAAGGCCAGCTTCT
    BnaGln1.3_C5 GGGGAAGCATGAAACAGCAGACATCAACACGTTCTCTTGGGGAGTGGCGAACCGTGGAGCTTCGGTGAGAGTGGGACGTGACACTGAGAAAGAAGGCAAAGGTTACTTCGAGGATCGTAGGCCAGCTTCT
```

```
Consensus              GGGGAAGCAcGAgACcGCAtACATCAACACGTTCTCTTGGGGAGTGGCGAACCGTGGAGCTTCGGTGAGAGTGGGACGaGACACTGAGAAgGAAGGCAAAGGTTACTTCGAaGAcaGaAGGCCAGCTTCT

                       1951 1960      1970      1980      1990      2000      2010      2020      2030      2040      2050      2060      2070      2080
                       |--------+---------+---------+---------+---------+---------+---------+---------+---------+---------+---------+---------+---------|
BnaA.GLN1.3.c          AACATGGATCCTTATGTCGTTACGTCCATGATCGCTGAAACCACCATCCTCGGTTAATCAATCATGCACGTTTTAATGTTTTGGTTTTCTCCGGTCTGGTTTTCAATTTGTGAACTTATGTGCTTGTGAT
mRNA.BnaA.GLN1.3.c     AACATGGATCCTTATGTCGTTACGTCCATGATCGCTGAAACCACCATCCTCGGTTAATCAATCATGCACGTTTTAATGTTTTGGTTTTCTCCGGTCTGGTTTTCAATTTGTGAACTTATGTGCTTGTGAT
BnaGln1.3_C5           AACATGGATCCTTATGTCGTCACTTCCATGATCGCTGAAACCACCATCCTCGGTTAATCAGA-------------------------------------------------------ATGTTTCTCTAAT
Consensus              AACATGGATCCTTATGTCGTtACgTCCATGATCGCTGAAACCACCATCCTCGGTTAATCAatcatgcacgttttaatgttttggttttctccggtctggttttcaatttgtgaacttATGTgctTgTgAT

                       2081 2090      2100      2110      2120      2130      2140      2150      2160      2170      2180      2190      2200      2210
                       |--------+---------+---------+---------+---------+---------+---------+---------+---------+---------+---------+---------+---------|
BnaA.GLN1.3.c          TGCAGTCTATTTAGGTTTAAATAATTCTTATGACGACATTTGGTGTCTTTTTCTTTAGTTTCGAATTATTAGATAAAAGGATTCTTATGGTGAAAAAGATAAGTGTTGTTTGAAAATTGAAATGATAATA
mRNA.BnaA.GLN1.3.c     TGCAGTCTATTTAGGTTTAAATAATTCTTATGACGACATTTGGTGTCTTTTTCTTTAGTTTCGAATTATTAGATAAAAGGATTCTTATGGTGAAAAAGATAAGTGTTGTTTGAAAATTGAAATGATAATA
BnaGln1.3_C5           TGCA--CTAATAATTCTTATCATGGACTTAGTAGCTCGACGGTGTTTGGTGTGTTTCGTTTCGAATAATTAAATAAA-GGATTTTCATGATG
Consensus              TGCAgtCTAtTtAggtTTAaataattCTTAtgAcgaCatttGgtgTcttTtTcTTTaGTTTCGAATtATTAgATAAAaGGATTcTtATGgTGaaaaagataagtgttgtttgaaaattgaaatgataata

                       2211 2220      2230      2240      2250      2260      2270      2280      2290      2300      2310      2320      2330      2340
                       |--------+---------+---------+---------+---------+---------+---------+---------+---------+---------+---------+---------+---------|
BnaA.GLN1.3.c          TTAGTATCAACGTATGGCTCGGCACATTGGGAGGTTGATAGTGTATAACCAGAGAATTATCAAAGAACCTATAAACAGATATTTAGTTATTGTTACTATATATAAATATAATTTCTAAAAACAGTTGTTC
mRNA.BnaA.GLN1.3.c     TTAGTATCAACGTATGGCTCGGCACATTGGGAGGTTGATAGTGTATAACCAGAGAATTATCAAAGAACCTATAAACAGATATTTAGTTATTGTTACTATATATAAATATAATTTCTAAAAACAGTTGTTC
BnaGln1.3_C5
Consensus              ttagtatcaacgtatggctcggcacattgggaggttgatagtgtataaccagagaattatcaaagaacctataaacagatatttagttattgttactatatataaatataatttctaaaaacagttgttc

                       2341 2350      2360      2370      2380      2390      2400      2410      2420      2430      2440      2450      2460      2470
                       |--------+---------+---------+---------+---------+---------+---------+---------+---------+---------+---------+---------+---------|
BnaA.GLN1.3.c          TATTGAGCTTTTTGGCAACATAATCTAATGTATACACCACTATCTAGCTTACACCTTCAAGTAAGAAATTTTGTTAGAATTAGCATGAATGGAACACTCTAAAAACATGATCTTCCTGGATTCACAACCC
mRNA.BnaA.GLN1.3.c     TATTGAGCTTTTTGGCAACATAATCTAATGTATACACCACTATCTAGCTTACACCTTCAAGTAAGAAATTTTGTTAGAATTAGCATGAATGGAACACTCTAAAAACATGATCTTCCTGGATTCACAACCC
BnaGln1.3_C5
Consensus              tattgagctttttggcaacataatctaatgtatacaccactatctagcttacaccttcaagtaagaaattttgttagaattagcatgaatggaacactctaaaaacatgatcttcctggattcacaaccc

                       2471 2480      2490      2500      2510      2520      2530      2540      2550      2560      2570      2580      2590      2600
                       |--------+---------+---------+---------+---------+---------+---------+---------+---------+---------+---------+---------+---------|
BnaA.GLN1.3.c          AATTTTTATTTTTATTTTTAAACAAACAACAATTTCATCTAAAACCGTTTTTTCATGTCGACTTTCTTATAACTATATGGGGAGAAGAAGTGAAAGTTTCAGATAATTACAAAAGTATAAAAAACGAGTT
mRNA.BnaA.GLN1.3.c     AATTTTTATTTTTATTTTTAAACAAACAACAATTTCATCTAAAACCGTTTTTTCATGTCGACTTTCTTATAACTATATGGGGAGAAGAAGTGAAAGTTTCAGATAATTACAAAAGTATAAAAAACGAGTT
BnaGln1.3_C5
Consensus              aatttttatttttatttttaaacaaacaacaatttcatctaaaaccgttttttcatgtcgactttcttataactatatggggagaagaagtgaaagtttcagataattacaaaagtataaaaaacgagtt

                       2601 2610      2620      2630      2640      2650      2660      2670      2680      2690 2695
                       |--------+---------+---------+---------+---------+---------+---------+---------+---------+----|
BnaA.GLN1.3.c          ATTTTGGTTACGAGCACTAATTCAAAAGTATACAAATTTAAATACAAAAAATTAAATTATAATGTATTATATTTTAAACTATTGAAAAATAAAAA
mRNA.BnaA.GLN1.3.c     ATTTTGGTTACGAGCACTAATTCAAAAGTATACAAATTTAAATACAAAAAATTAAATTATAATGTATTATATTTTAAACTATTGAAAAATAAAAA
BnaGln1.3_C5
Consensus              attttggttacgagcactaattcaaaagtatacaaatttaaatacaaaaaattaaattataatgtattatattttaaactattgaaaaataaaaa
```

```
                   1        10        20        30        40        50        60        70        80        90        100       110       120       130
  BnaC.GLN1.3.c    ATAAAAACACTCTCAGGAGAAGAGCTGTATCGAGATCGTCTTCCCTAAACAACACTCATTGATTGATTACTATCCGACGCAGCCATGTCTCTGCTCTCAGATCTCGTCAACCTCAACCTCTCCGACTCCA
mRNA.BnaC.GLN1.3.c ATAAAAACACTCTCAGGAGAAGAGCTGTATCGAGATCGTCTTCCCTAAACAACACTCATTGATTGATTACTATCCGACGCAGCCATGTCTCTGCTCTCAGATCTCGTCAACCTCAACCTCTCCGACTCCA
  BnaGln1.3_C6                                   GGAGATCGTCTTCCCTAAACAACACTCATTGATTGATTACTATCCGACGCAGCCATGTCTCTGCTCTCAGATCTCGTCAACCTCAACCTCTCCGACTCCA
  Consensus        ataaaaacactctcaggagaagagctgtatcGAGATCGTCTTCCCTAAACAACACTCATTGATTGATTACTATCCGACGCAGCCATGTCTCTGCTCTCAGATCTCGTCAACCTCAACCTCTCCGACTCCA

                   131      140       150       160       170       180       190       200       210       220       230       240       250       260
  BnaC.GLN1.3.c    CCAAGCAAATCATCGCCGAATACATATGGTTCCATCATCTCCCTTCTCTCTGTTTCTCTCTCACGACTAGTCTCATTATCTCTGTTTTCTTTTCCTCGTTATCTCTGTTTGTTACTATTGCTGTGATCGA
mRNA.BnaC.GLN1.3.c CCAAGCAAATCATCGCCGAATACATATGG-----------------------------------------------------------------------------------------------------
  BnaGln1.3_C6     CCAAGCAAATCATCGCCGAATACATATGG-----------------------------------------------------------------------------------------------------
  Consensus        CCAAGCAAATCATCGCCGAATACATATGG.....................................................................................................

                   261      270       280       290       300       310       320       330       340       350       360       370       380       390
  BnaC.GLN1.3.c    AAAGCTTTGATCTCTTATGTTTTTGACAATTTCGGTTTGAACACGATTCACTTCACTCTGTTTTCACTTGTTCTGTCTCGATGAGATTGACGTGTACATCGTAAAGCTTGTCTCTTTCTTTGTTGTCGTC
mRNA.BnaC.GLN1.3.c ----------------------------------------------------------------------------------------------------------------------------------
  BnaGln1.3_C6     ----------------------------------------------------------------------------------------------------------------------------------
  Consensus        ..................................................................................................................................

                   391      400       410       420       430       440       450       460       470       480       490       500       510       520
  BnaC.GLN1.3.c    TTCTGGGTTTGTAATTTTCTCGTATGAATGTTGAATGAACAGGATCGGTGGATCTGGCATGGACATTAGAAGCAAAGCCAGAGTAAATTTGATACTCTGTTTTTTTCATCCTCTGTTTATTGTATGATCG
mRNA.BnaC.GLN1.3.c -------------------------------------------ATCGGTGGATCTGGCATGGACATTAGAAGCAAAGCCAGA------------------------------------------------
  BnaGln1.3_C6     -------------------------------------------ATCGGTGGATCTGGCATGGACATTAGAAGCAAAGCCAGA------------------------------------------------
  Consensus        ...........................................ATCGGTGGATCTGGCATGGACATTAGAAGCAAAGCCAGA................................................

                   521      530       540       550       560       570       580       590       600       610       620       630       640       650
  BnaC.GLN1.3.c    TTCTGATTTGTTTGTGGATGATACAGACACTTCCAGGACCAGTGAGCGATCCATCAAAGCTTCCCAAGTGGAACTACGACGGATCCAGCACCGGTCAGGCCTCTGGAGACAACAGTGAAGTCATTCTATA
mRNA.BnaC.GLN1.3.c --------------------------ACACTTCCAGGACCAGTGAGCGATCCATCAAAGCTTCCCAAGTGGAACTACGACGGATCCAGCACCGGTCAGGCCTCTGGAGACAACAGTGAAGTCATTCTATA
  BnaGln1.3_C6     --------------------------ACACTTCCAGGACCAGTGAGCGATCCATCAAAGCTTCCCAAGTGGAACTACGACGGATCCAGCACCGGTCAGGCCTCTGGAGACAACAGTGAAGTCATTCTATA
  Consensus        ..........................ACACTTCCAGGACCAGTGAGCGATCCATCAAAGCTTCCCAAGTGGAACTACGACGGATCCAGCACCGGTCAGGCCTCTGGAGACAACAGTGAAGTCATTCTATA

                   651      660       670       680       690       700       710       720       730       740       750       760       770       780
  BnaC.GLN1.3.c    GTATGTTCTTCGATCCTCTTTATCTACTGTATAAGCATTTACACAAATCTTGTCTTAATCTTCTTTCTTGTTTTGTCTCGTTTTATGAATCGATAGCCCTCAGGCGATATTCCGTGATCCGTTCAGGAGA
mRNA.BnaC.GLN1.3.c ------------------------------------------------------------------------------------------------CCCTCAGGCGATATTCCGTGATCCGTTCAGGAGA
  BnaGln1.3_C6     ------------------------------------------------------------------------------------------------CCCTCAGGCGATATTCCGTGATCCGTTCAGGAGA
  Consensus        ................................................................................................CCCTCAGGCGATATTCCGTGATCCGTTCAGGAGA

                   781      790       800       810       820       830       840       850       860       870       880       890       900       910
  BnaC.GLN1.3.c    GGCGACAACATCCTGGTAGGGCTGATTTTTTATATGATTAGTGTGAGACTAAATTCAATTTGGATCAGAATTTTTTTAAAAAATGATTGGTCAATGTGATATATATTCAGGTGATGTGTGATGCATACAC
mRNA.BnaC.GLN1.3.c GGCGACAACATCCTGGT-----------------------------------------------------------------------------------------------GATGTGTGATGCATACAC
  BnaGln1.3_C6     GGCGACAACATCCTGGT-----------------------------------------------------------------------------------------------GATGTGTGATGCATACAC
  Consensus        GGCGACAACATCCTGGT...............................................................................................GATGTGTGATGCATACAC

                   911      920       930       940       950       960       970       980       990       1000      1010      1020      1030      1040
  BnaC.GLN1.3.c    ACCGGCCGGAGATCCAATTCCGACCAACAAGAGGCACAAGGCTGCTAAGATCTTCAGCCATCCTAACGTTGCCAAAGAAGTGCCTTGGTAATCTCTTCTTCTTTGTATATTGATCTCTCTGCAAATCAAT
mRNA.BnaC.GLN1.3.c ACCGGCCGGAGATCCAATTCCGACCAACAAGAGGCACAAGGCTGCTAAGATCTTCAGCCATCCTAACGTTGCCAAAGAAGTGCCTTGGTA----------------------------------------
  BnaGln1.3_C6     ACCGGCCGGAGATCCAATTCCGACCAACAAGAGGCACAAGGCTGCTAAGATCTTCAGCCATCCTAACGTTGCCAAAGAAGTGCCTTGGTA----------------------------------------
  Consensus        ACCGGCCGGAGATCCAATTCCGACCAACAAGAGGCACAAGGCTGCTAAGATCTTCAGCCATCCTAACGTTGCCAAAGAAGTGCCTTGGTA........................................

                   1041 1050      1060      1070      1080      1090      1100      1110      1120      1130      1140      1150      1160      1170
  BnaC.GLN1.3.c    CTTATATAATTATACTCTCTCTCTCTGTATCAAAAGGTATGGGATTGAGCAAGAATACACTTTGATGCAAAAGGGTATGAACTGGCCTATTGGTTGGCCTGTTGGTGGCTTCCCTGGTCCTCAGGTAAAT
mRNA.BnaC.GLN1.3.c ---------------------------------------TGGGATTGAGCAAGAATACACTTTGATGCAAAAGGGTATGAACTGGCCTATTGGTTGGCCTGTTGGTGGCTTCCCTGGTCCTCAGG-----
  BnaGln1.3_C6     ---------------------------------------TGGGATTGAGCAAGAATACACTTTGATGCAAAAGGGTATGAACTGGCCTATTGGTTGGCCTGTTGGTGGCTTCCCTGGTCCTCAGG-----
  Consensus        .......................................TGGGATTGAGCAAGAATACACTTTGATGCAAAAGGGTATGAACTGGCCTATTGGTTGGCCTGTTGGTGGCTTCCCTGGTCCTCAGG.....

                   1171 1180      1190      1200      1210      1220      1230      1240      1250      1260      1270      1280      1290      1300
  BnaC.GLN1.3.c    CTAAATAATCAACTGATCCTAATGTCATCACAACTCTTTTTTTTTACCTAACTTTCTAATATTATGACGCAGGGACCATACTACTGTGGTGTGGGAGCTGACAAAGCCATTGGTCGTGACATTGTGGACG
mRNA.BnaC.GLN1.3.c -------------------------------------------------------------------------GACCATACTACTGTGGTGTGGGAGCTGACAAAGCCATTGGTCGTGACATTGTGGACG
  BnaGln1.3_C6     -------------------------------------------------------------------------GACCATACTACTGTGGTGTGGGAGCTGACAAAGCCATTGGTCGTGACATTGTGGACG
  Consensus        .........................................................................GACCATACTACTGTGGTGTGGGAGCTGACAAAGCCATTGGTCGTGACATTGTGGACG

                   1301 1310      1320      1330      1340      1350      1360      1370      1380      1390      1400      1410      1420      1430
  BnaC.GLN1.3.c    CACACTACAAGGCCTGTCTTTACGCAGGTATTGGCATCTCTGGTGTCAATGGAGAGGTCATGCCTGGACAGTGGGAGTTCCAAGTCGGTCCAGTTGAGGGTATTAGTTCTGGTGATCAAGTCTGGGTCGC
mRNA.BnaC.GLN1.3.c CACACTACAAGGCCTGTCTTTACGCAGGTATTGGCATCTCTGGTGTCAATGGAGAGGTCATGCCTGGACAGTGGGAGTTCCAAGTCGGTCCAGTTGAGGGTATTAGTTCTGGTGATCAAGTCTGGGTCGC
  BnaGln1.3_C6     CACACTACAAGGCCTGTCTTTACGCAGGTATTGGCATCTCTGGTGTCAATGGAGAGGTCATGCCTGGACAGTGGGAGTTCCAAGTCGGTCCAGTTGAGGGTATTAGTTCTGGTGATCAAGTCTGGGTCGC
  Consensus        CACACTACAAGGCCTGTCTTTACGCAGGTATTGGCATCTCTGGTGTCAATGGAGAGGTCATGCCTGGACAGTGGGAGTTCCAAGTCGGTCCAGTTGAGGGTATTAGTTCTGGTGATCAAGTCTGGGTCGC

                   1431 1440      1450      1460      1470      1480      1490      1500      1510      1520      1530      1540      1550      1560
  BnaC.GLN1.3.c    TAGATACCTTCTTGAGGTAGAGTACTCTACTTTAACCGGTTCTATTGTGAATATCTCTACAAGATTTGCTAATGGCCATCTTATCTCTTTTGACAGAGGATCACTGAGATCTCTGGTGTAAATGTCAGCT
mRNA.BnaC.GLN1.3.c TAGATACCTTCTTGAG--------------------------------------------------------------------------------AGGATCACTGAGATCTCTGGTGTAAATGTCAGCT
  BnaGln1.3_C6     TAGATACCTTCTTGAG--------------------------------------------------------------------------------AGGATCACTGAGATCTCTGGTGTAAATGTCAGCT
  Consensus        TAGATACCTTCTTGAG................................................................................AGGATCACTGAGATCTCTGGTGTAAATGTCAGCT

                   1561 1570      1580      1590      1600      1610      1620      1630      1640      1650      1660      1670      1680      1690
  BnaC.GLN1.3.c    TCGACCCAAAACCAGTCCCGGTTAGTGTCTCTTTACCACACTCTCTCAACACTCTCTTCATGTAAAATAAGTTGTAACAATGTTTGCATGGGTTGTTCAGGGTGATTGTGCAACTACAGCACGAAGTCGA
mRNA.BnaC.GLN1.3.c TCGACCCAAAACCAGTCCCGGTTAGTGTCTCTTTACCACACTCTCTCAACA-------------------------------------------------------------ACTACAGCACGAAGTCGA
  BnaGln1.3_C6     TCGACCCAAAACCAGTCCCGGGTGATTGGAACGGAGCTGGAGCTCACTGCA-------------------------------------------------------------ACTACAGTACGAAGACGA
  Consensus        TCGACCCAAAACCAGTCCCGGtTagTgtctctttAcCacactCTCtCaaCA.............................................................ACTACAGcACGAAGtCGA

                   1691 1700      1710      1720      1730      1740      1750      1760      1770      1780      1790      1800      1810      1820
  BnaC.GLN1.3.c    TGAGGAACAACGGAGGATTAGCAGTGATAAAGAACGCGATAGAGAAGCTTCAGGTGAAGCACAAGGAGAACATTGCTGCGTACGGTGAAGGCAGCGAGCGTCGTCTCACGGGGAAGCACGAGACCGCATA
mRNA.BnaC.GLN1.3.c TGAGGAACAACGGAGGATTAGCAGTGATAAAGAACGCGATAGAGAAGCTTCAGGTGAAGCACAAGGAGAACATTGCTGCGTACGGTGAAGGCAGCGAGCGTCGTCTCACGGGGAAGCACGAGACCGCATA
  BnaGln1.3_C6     TGAGGAACGACGGAGGCTTAGCAGTGATAAAGAAAGCAATAGAGAAGCTTCAGGTGAAGCACAAGGAGCACATTGCTGCTTATGGTGAAGGCAACGAGCGTCGTCTCACGGGGAAGCATGAAACAGCAGA
  Consensus        TGAGGAACaACGGAGGaTTAGCAGTGATAAAGAAcGCgATAGAGAAGCTTCAGGTGAAGCACAAGGAGaACATTGCTGCgTAcGGTGAAGGCAgCGAGCGTCGTCTCACGGGGAAGCAcGAgACcGCAtA

                   1821 1830      1840      1850      1860      1870      1880      1890      1900      1910      1920      1930      1940      1950
  BnaC.GLN1.3.c    CATCAACACGTTCTCTTGGGGAGTGGCGAACCGTGGAGCTTCGGTGAGAGTGGGACGAGACACTGAGAAGGAAGGCAAAGGTTACTTCGAAGACAGAAGGCCAGCTTCTAACATGGATCCTTATGTCGTT
mRNA.BnaC.GLN1.3.c CATCAACACGTTCTCTTGGGGAGTGGCGAACCGTGGAGCTTCGGTGAGAGTGGGACGAGACACTGAGAAGGAAGGCAAAGGTTACTTCGAAGACAGAAGGCCAGCTTCTAACATGGATCCTTATGTCGTT
  BnaGln1.3_C6     CATCAACACGTTCTCTTGGGGAGTGGCGAACCGTGGAGCTTCGGTGAGAGTGGGACGTGACACTGAGAAAGAAGGCAAAGGTTACTTCGAGGATCGTAGGCCAGCTTCTAATATGGATCCTTATGTCGTC
```

file:///C:/Users/Camille/Desktop/Supp%20Figure%20alignements/C3c.gif[25/09/2013 13:29:32]

```
        Consensus  CATCAACACGTTCTCTTGGGGAGTGGCGAACCGTGGAGCTTCGGTGAGAGTGGGACGaGACACTGAGAAgGAAGGCAAAGGTTACTTCGAaGAcaGaAGGCCAGCTTCTAAcATGGATCCTTATGTCGTt

                   1951 1960      1970      1980      1990      2000      2010      2020      2030      2040      2050      2060      2070      2080
                   |--------+---------+---------+---------+---------+---------+---------+---------+---------+---------+---------+---------+---------|
    BnaC.GLN1.3.c  ACGTCCATGATCTG---AACCACCATCCTCGGTTAATCAGTCATGCACGTTTTAATGTTTTGGTTTTCTCCGGTCTGGTTTTCAATTTGTGAACTTATGTGCTTGTGATTGCAGTC----TATTTAGGTT
mRNA.BnaC.GLN1.3.c ACGTCCATGATCTG---AACCACCATCCTCGGTTAATCAGT-------------------------------------------------------ATGTGCTTGTGATTGCAGTC----TATTTAGGTT
    BnaGln1.3_C6   ACTTCCATGATCGCTGAAACCACCATCCTCGGTTAATCACA-------------------------------------------------------ATGTTTTTCTAATTGCACTAATAATTCTTATCAT
        Consensus  ACgTCCATGATCtg...AACCACCATCCTCGGTTAATCAgt.......................................................ATGTgcTTgTgATTGCAgTc....TatTTAggtT

                   2081 2090      2100      2110      2120      2130      2140      2150      2160      2170      2180      2190      2200      2210
                   |--------+---------+---------+---------+---------+---------+---------+---------+---------+---------+---------+---------+---------|
    BnaC.GLN1.3.c  TAAATAATTCTTATGACGACATTTGGTGTCTTTTTCTTTAGTTTCGAATTATTAGATAAAAGGATTCTTATGGTGAAAAAGATAAGTGTTGTTTGAAAATTGAAATGATAATATTAGTATCAACGTATGG
mRNA.BnaC.GLN1.3.c TAAATAATTCTTATGACGACATTTGGTGTCTTTTTCTTTAGTTTCGAATTATTAGATAAAAGGATTCTTATGGTGAAAAAGATAAGTGTTGTTTGAAAATTGAAATGATAATATTAGTATCAACGTATGG
    BnaGln1.3_C6   GGACTCAGTAGCACGACGGTGTTTGGTGTGTTTC------GTTTCGAATAATTAAATAAA-GGATTTTCATGATGAAAAAAAAAA---------------------------------------------
        Consensus  taAaTaAtTcttAtGACGacaTTTGGTGTcTTTttctttaGTTTCGAATtATTAgATAAAaGGATTcTtATGgTGAAAAAgAtAAgtgttgtttgaaaattgaaatgataatattagtatcaacgtatgg

                   2211 2220      2230      2240      2250      2260      2270      2280      2290      2300      2310      2320      2330      2340
                   |--------+---------+---------+---------+---------+---------+---------+---------+---------+---------+---------+---------+---------|
    BnaC.GLN1.3.c  CTCGGCACATTGGGAGGTTGATAGTGTATAACCAGAGAATTATCAAAGAACCTATAAACAGATATTTAGTTATTGTTACTATATATAAATATAATTTCTAAAAACAGTTGTTCTATTGAGCTTTTTGGCA
mRNA.BnaC.GLN1.3.c CTCGGCACATTGGGAGGTTGATAGTGTATAACCAGAGAATTATCAAAGAACCTATAAACAGATATTTAGTTATTGTTACTATATATAAATATAATTTCTAAAAACAGTTGTTCTATTGAGCTTTTTGGCA
    BnaGln1.3_C6   ----------------------------------------------------------------------------------------------------------------------------------
        Consensus  ctcggcacattgggaggttgatagtgtataaccagagaattatcaaagaacctataaacagatatttagttattgttactatatataaatataatttctaaaaacagttgttctattgagctttttggca

                   2341 2350      2360      2370      2380      2390      2400      2410      2420      2430      2440      2450      2460      2470
                   |--------+---------+---------+---------+---------+---------+---------+---------+---------+---------+---------+---------+---------|
    BnaC.GLN1.3.c  ACATAATCTAATGTATACACCACTATCTAGCTTACATCTTCAAGTAAGAAATTTTGTTAGAATTAGCATGAATGGAACACTCTAAAAACATGATCTTCCTGGATTCACAACCCAATTTTTATTTTTACTT
mRNA.BnaC.GLN1.3.c ACATAATCTAATGTATACACCACTATCTAGCTTACATCTTCAAGTAAGAAATTTTGTTAGAATTAGCATGAATGGAACACTCTAAAAACATGATCTTCCTGGATTCACAACCCAATTTTTATTTTTACTT
    BnaGln1.3_C6   ----------------------------------------------------------------------------------------------------------------------------------
        Consensus  acataatctaatgtatacaccactatctagcttacatcttcaagtaagaaattttgttagaattagcatgaatggaacactctaaaaacatgatcttcctggattcacaacccaatttttatttttactt

                   2471 2480      2490      2500      2510      2520      2530      2540      2550      2560      2570      2580      2590      2600
                   |--------+---------+---------+---------+---------+---------+---------+---------+---------+---------+---------+---------+---------|
    BnaC.GLN1.3.c  TTAAACAAACAACAATTTCATCTAAAACCGTTTTTTCATGTCGACTTTCTTATAACTATATGGGGAGAAGAAGTGAAAGTTTCAGATAATTACAAAAGTATAAAAAACGAGTTATTTTGGTTACGAGCAC
mRNA.BnaC.GLN1.3.c TTAAACAAACAACAATTTCATCTAAAACCGTTTTTTCATGTCGACTTTCTTATAACTATATGGGGAGAAGAAGTGAAAGTTTCAGATAATTACAAAAGTATAAAAAACGAGTTATTTTGGTTACGAGCAC
    BnaGln1.3_C6   ----------------------------------------------------------------------------------------------------------------------------------
        Consensus  ttaaacaaacaacaatttcatctaaaaccgttttttcatgtcgactttcttataactatatggggagaagaagtgaaagtttcagataattacaaaagtataaaaaacgagttattttggttacgagcac

                   2601 2610      2620      2630      2640      2650      2660      2670 2676
                   |--------+---------+---------+---------+---------+---------+---------+-----|
    BnaC.GLN1.3.c  TAATTCAAAAGTATACAAATTTAAATACAAAAAATTAAATTATAATGTATTATATTTTAAACTATTGAAAAATAAA
mRNA.BnaC.GLN1.3.c TAATTCAAAAGTATACAAATTTAAATACAAAAAATTAAATTATAATGTATTATATTTTAA
    BnaGln1.3_C6   -------------------------------------------------------------------AAAAAAAAA
        Consensus  taattcaaaagtatacaaatttaaatacaaaaaattaaattataatgtattatattttaa.......aaaaa.aaa
```

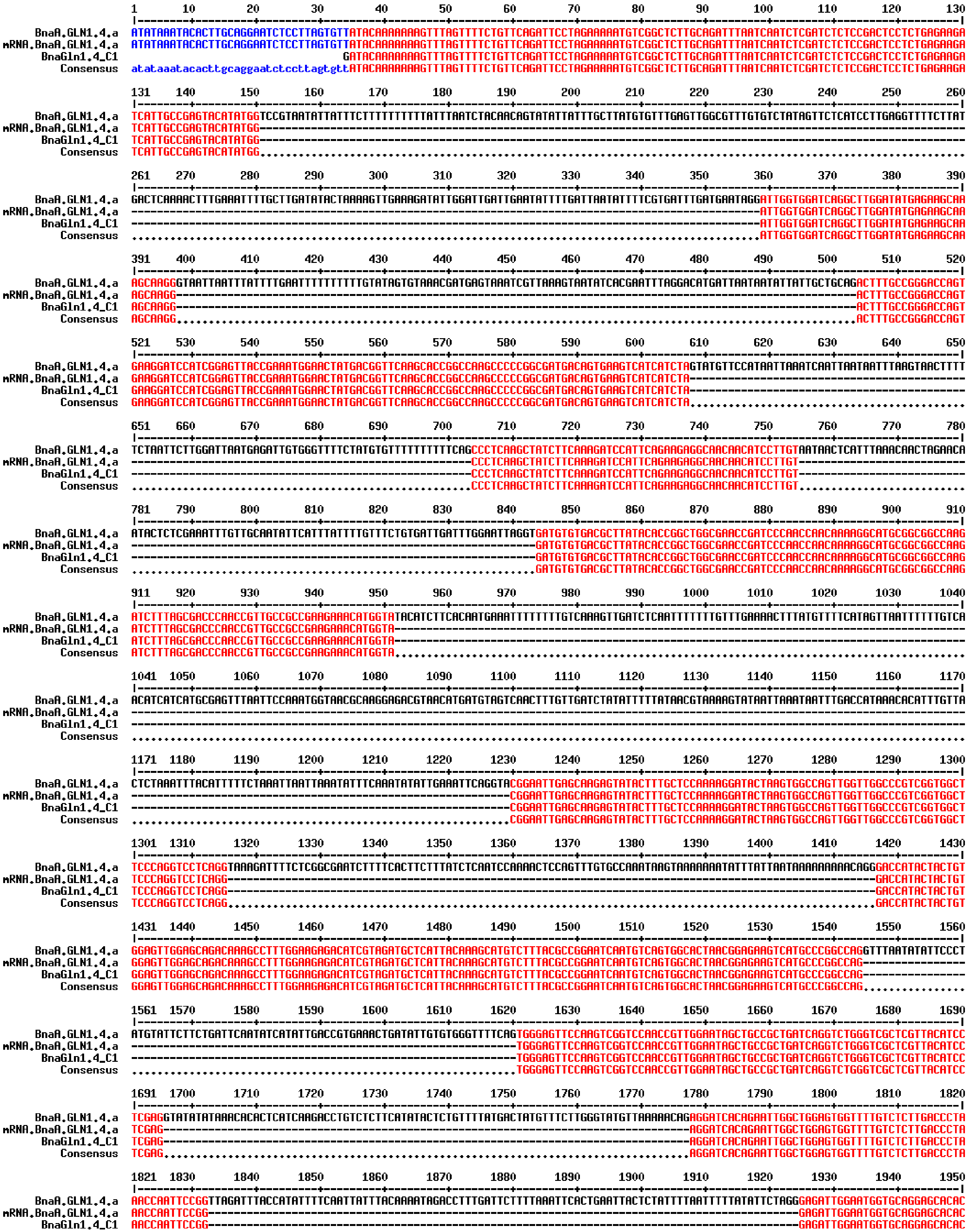
BnaA.GLN1.4.a
mRNA.BnaA.GLN1.4.a
BnaGln1.4_C1
Consensus

```
           Consensus  AACCAATTCCGG............................................................................................GAGATTGGAATGGTGCAGGAGCACAC

                      1951 1960      1970      1980      1990      2000      2010      2020      2030      2040      2050      2060      2070      2080
                      |--------+---------+---------+---------+---------+---------+---------+---------+---------+---------+---------+---------+---------|
     BnaA.GLN1.4.a    ACAAATTACAGGTCTGCTATGATTCCGTTTATATGTTTTTTTCTCTGTGTTTTTTTTTGTTTTCTGATTTTGGTTTTCTTGTTTTCTGCAGCACCAAGTCGATGAGGGAAGATGGAGGGTACGAGATCAT
mRNA.BnaA.GLN1.4.a    ACAAATTACAG--------------------------------------------------------------------------------CACCAAGTCGATGAGGGAAGATGGAGGGTACGAGATCAT
     BnaGln1.4_C1     ACAAATTACAG--------------------------------------------------------------------------------CACCAAGTCGATGAGGGAAGATGGAGGGTACGAGATCAT
           Consensus  ACAAATTACAG................................................................................CACCAAGTCGATGAGGGAAGATGGAGGGTACGAGATCAT

                      2081 2090      2100      2110      2120      2130      2140      2150      2160      2170      2180      2190      2200      2210
                      |--------+---------+---------+---------+---------+---------+---------+---------+---------+---------+---------+---------+---------|
     BnaA.GLN1.4.a    AAAGAAAGCGATAGAGAAGCTTGGACTGCGTCACAAGGAACACATCGCTGCTTATGGTGAAGGCAACGAGCGTCGTCTCACTGGAAGACACGAGACTGCTGATATCAACACTTTCTTATGGGTAAGACTT
mRNA.BnaA.GLN1.4.a    AAAGAAAGCGATAGAGAAGCTTGGACTGCGTCACAAGGAACACATCGCTGCTTATGGTGAAGGCAACGAGCGTCGTCTCACTGGAAGACACGAGACTGCTGATATCAACACTTTCTTATGGG--------
     BnaGln1.4_C1     AAAGAAAGCGATAGAGAAGCTTGGACTGCGTCACAAGGAACACATCGCTGCTTATGGTGAAGGCAACGAGCGTCGTCTCACTGGAAGACACGAGACTGCTGATATCAACACTTTCTTATGGG--------
           Consensus  AAAGAAAGCGATAGAGAAGCTTGGACTGCGTCACAAGGAACACATCGCTGCTTATGGTGAAGGCAACGAGCGTCGTCTCACTGGAAGACACGAGACTGCTGATATCAACACTTTCTTATGGG........

                      2211 2220      2230      2240      2250      2260      2270      2280      2290      2300      2310      2320      2330      2340
                      |--------+---------+---------+---------+---------+---------+---------+---------+---------+---------+---------+---------+---------|
     BnaA.GLN1.4.a    TTCTTAAAATGTATACATTATTAGCCTAAAGCATGATCTAACTTAAACCGGTTTTGTAAAGGGTGTTGCAAACCGTGGGGCATCGATTAGGGTTGGTCGTGACACAGAGAAAGATGGGAAAGGATACTTT
mRNA.BnaA.GLN1.4.a    --------------------------------------------------------------GTGTTGCAAACCGTGGGGCATCGATTAGGGTTGGTCGTGACACAGAGAAAGATGGGAAAGGATACTTT
     BnaGln1.4_C1     --------------------------------------------------------------GTGTTGCAAACCGTGGGGCATCGATTAGGGTTGGTCGTGACACAGAGAAAGATGGGAAAGGATACTTT
           Consensus  ..............................................................GTGTTGCAAACCGTGGGGCATCGATTAGGGTTGGTCGTGACACAGAGAAAGATGGGAAAGGATACTTT

                      2341 2350      2360      2370      2380      2390      2400      2410      2420      2430      2440      2450      2460      2470
                      |--------+---------+---------+---------+---------+---------+---------+---------+---------+---------+---------+---------+---------|
     BnaA.GLN1.4.a    GAAGATCGTAGGCCAGCGTCGAACATGGATCCCTACACTGTGACCTCCATGGTTGCTGAAACCACAATCCTCTGGAAACCATGAAGGAAGAAACCTTGAGTCTCAAGGAACCTCTTATATCAGTTCATGT
mRNA.BnaA.GLN1.4.a    GAAGATCGTAGGCCAGCGTCGAACATGGATCCCTACACTGTGACCTCCATGGTTGCTGAAACCACAATCCTCTGGAAACCATGAAGGAAGAAACCTTGAGTCTCAAGGAACCTCTTATATCAGTTCATGT
     BnaGln1.4_C1     GAAGATCGTAGGCCAGCGTCGAACATGGATCCCTACACTGTGACCTCCATGGTTGCTGAAACCACAATCCTCTGGAAACCATGAAGGAAGAAACCTTGAGTCTCAAGGAACCTCTTATATCAGTTCATGT
           Consensus  GAAGATCGTAGGCCAGCGTCGAACATGGATCCCTACACTGTGACCTCCATGGTTGCTGAAACCACAATCCTCTGGAAACCATGAAGGAAGAAACCTTGAGTCTCAAGGAACCTCTTATATCAGTTCATGT

                      2471 2480      2490      2500      2510      2520      2530      2540      2550      2560      2570      2580      2590      2600
                      |--------+---------+---------+---------+---------+---------+---------+---------+---------+---------+---------+---------+---------|
     BnaA.GLN1.4.a    TGATTCTTCTATTGTCTATTCCTCTTTATGAAACACTTCTCATGTGTTCTTTGTTTAAGAATGTTTGATTTAAACTCTTTCCAAGAAATAATAGTAGTTCTTTTCCCTAGCTTTTACTTTCTTCTATTTC
mRNA.BnaA.GLN1.4.a    TGATTCTTCTATTGTCTATTCCTCTTTATGAAACACTTCTCATGTGTTCTTTGTTTAAGAATGTTTGATTTAAACTCTTTCCAAGAAATAATAGTAGTTCTTTTCCCTAGCTTTTACTTTCTTCTATTTC
     BnaGln1.4_C1     TGATTCTTCTATTGTCTATTCCTCTTTATGAAACACTTCTCATGTGTTCTTTGTTTAAGAATGTTTGATTTAAACTCTTTCCAAGAAATAATAGTAGTTCTTTTCCCTAAAAAA
           Consensus  TGATTCTTCTATTGTCTATTCCTCTTTATGAAACACTTCTCATGTGTTCTTTGTTTAAGAATGTTTGATTTAAACTCTTTCCAAGAAATAATAGTAGTTCTTTTCCCTAgcttttactttcttctatttc

                      2601 2610      2620      2630      2640      2650      2660      2670      2680      2690      2700      2710      2720      2730
                      |--------+---------+---------+---------+---------+---------+---------+---------+---------+---------+---------+---------+---------|
     BnaA.GLN1.4.a    AGAATCTATATGTTATTACAAGATTCAAGTCTCGGTTTAGTCAAGTTTTTGACATTAACTTAAACCGAAGCTTACAAACTTCTCCATTACTAGGCGGCCCAAACTAGACCCGACTCGACGGTAACCGAAG
mRNA.BnaA.GLN1.4.a    AGAATCTATATGTTATTACAAGATTCAAGTCTCGGTTTAGTCAAGTTTTTGACATTAACTTAAACCGAAGCTTACAAACTTCTCCATTACTAGGCGGCCCAAACTAGACCCGACTCGACGGTAACCGAAG
     BnaGln1.4_C1
           Consensus  agaatctatatgttattacaagattcaagtctcggtttagtcaagtttttgacattaacttaaaccgaagcttacaaacttctccattactaggcggcccaaactagacccgactcgacggtaaccgaag

                      2731 2739
                      |-------|
     BnaA.GLN1.4.a    ATGGATAAA
mRNA.BnaA.GLN1.4.a    ATGGATAAA
     BnaGln1.4_C1
           Consensus  atggataaa
```

```
                  1       10        20        30        40        50        60        70        80        90        100       110       120      130
                  |--------+---------+---------+---------+---------+---------+---------+---------+---------+---------+---------+---------+---------|
    BnaC.GLN1.4.a TATATAAATACACTTGCAGGAATCTCTTTAGTGTCATACAAAAAAAGTTTAGTATTCTCTTCAGAGTCCTAGAAAAATGTCGGCTCTTGCAGATTTAATCAATCTCGATCTCTCCGACTCCTCTGAGAAG
mRNA.BnaC.GLN1.4.a TATATAAATACACTTGCAGGAATCTCTTTAGTGTCATACAAAAAAAGTTTAGTATTCTCTTCAGAGTCCTAGAAAAATGTCGGCTCTTGCAGATTTAATCAATCTCGATCTCTCCGACTCCTCTGAGAAG
     BnaGln1.4_C2           GGGTCGACGGCCATTACGGCCGGGGATACAAAAAAAGTTTAGTATTCTCTTCAGAGTCCTAGAAAAATGTCGGCTCTTGCAGATTTAATCAATCTCGATCTCTCCGACTCCTCTGAGAAG
        Consensus tatataaatacacttgCaGgaATctCtttaGtGtcATACAAAAAAAGTTTAGTATTCTCTTCAGAGTCCTAGAAAAATGTCGGCTCTTGCAGATTTAATCAATCTCGATCTCTCCGACTCCTCTGAGAAG

                  131     140       150       160       170       180       190       200       210       220       230       240       250      260
                  |--------+---------+---------+---------+---------+---------+---------+---------+---------+---------+---------+---------+---------|
    BnaC.GLN1.4.a ATCATTGCCGAGTACATATGGTCCGTAAAATTCTTCATCGATCGATGTCAAATAATATTTCTTTTTGATTTAATCTACAACAGTATATTATTTGTTTATGTGTTTGAGTTGGCGTTTGTGTCTATAGTTC
mRNA.BnaC.GLN1.4.a ATCATTGCCGAGTACATATGG-------------------------------------------------------------------------------------------------------------
     BnaGln1.4_C2 ATCATTGCCGAGTACATATGG-------------------------------------------------------------------------------------------------------------
        Consensus ATCATTGCCGAGTACATATGG.............................................................................................................

                  261     270       280       290       300       310       320       330       340       350       360       370       380      390
                  |--------+---------+---------+---------+---------+---------+---------+---------+---------+---------+---------+---------+---------|
    BnaC.GLN1.4.a TCATCCATGAGGTTTTCTTATGACTCAAAACTTTGAAATTTTGCTTGATATACTAAAAGTTGAAAGATATTGGATTGATTGAATATTTTGATTAATATTTTCGTGATTGATGAATAGGATTGGTGGATCA
mRNA.BnaC.GLN1.4.a ----------------------------------------------------------------------------------------------------------------------ATTGGTGGATCA
     BnaGln1.4_C2 ----------------------------------------------------------------------------------------------------------------------ATTGGTGGATCA
        Consensus ......................................................................................................................ATTGGTGGATCA

                  391     400       410       420       430       440       450       460       470       480       490       500       510      520
                  |--------+---------+---------+---------+---------+---------+---------+---------+---------+---------+---------+---------+---------|
    BnaC.GLN1.4.a GGCTTGGATATGAGAAGCAAAGCAAGGGTAATTTATTTATTTTTTTGTGTATAGAGTAAATCGTTAAAGTAATATCAAGAATTTAGGACATGATTAATAATATTATTGCTGCAGACTTTGCCGGGACCTG
mRNA.BnaC.GLN1.4.a GGCTTGGATATGAGAAGCAAAGCAAGG---------------------------------------------------------------------------------------ACTTTGCCGGGACCTG
     BnaGln1.4_C2 GGCTTGGATATGAGAAGCAAAGCAAGG---------------------------------------------------------------------------------------ACTTTGCCGGGACCTG
        Consensus GGCTTGGATATGAGAAGCAAAGCAAGG.......................................................................................ACTTTGCCGGGACCTG

                  521     530       540       550       560       570       580       590       600       610       620       630       640      650
                  |--------+---------+---------+---------+---------+---------+---------+---------+---------+---------+---------+---------+---------|
    BnaC.GLN1.4.a TGAAGGATCCATCGGAGTTACCGAAATGGAACTATGACGGTTCAAGCACCGGGCAAGCTCCCGGCGATGATAGTGAAGTCATCATCTAGTATGTTCCGTAATTAAATCGATTAATAAATTAAAAGTAAAA
mRNA.BnaC.GLN1.4.a TGAAGGATCCATCGGAGTTACCGAAATGGAACTATGACGGTTCAAGCACCGGGCAAGCTCCCGGCGATGATAGTGAAGTCATCATCTA------------------------------------------
     BnaGln1.4_C2 TGAAGGATCCATCGGAGTTACCGAAATGGAACTATGACGGTTCAAGCACCGGGCAAGCTCCCGGCGATGATAGTGAAGTCATCATCTA------------------------------------------
        Consensus TGAAGGATCCATCGGAGTTACCGAAATGGAACTATGACGGTTCAAGCACCGGGCAAGCTCCCGGCGATGATAGTGAAGTCATCATCTA..........................................

                  651     660       670       680       690       700       710       720       730       740       750       760       770      780
                  |--------+---------+---------+---------+---------+---------+---------+---------+---------+---------+---------+---------+---------|
    BnaC.GLN1.4.a AATTAATTCTTGAATTAATGAGATTTTGGGTTTTCTATGTGTTTTTTCAGCCCTCAAGCTATCTTCAAAGATCCATTCAGAAGAGGCAACAACATCCTTGTAATAACTCAATTAAACAACTAGAACAATA
mRNA.BnaC.GLN1.4.a --------------------------------------------------CCCTCAAGCTATCTTCAAAGATCCATTCAGAAGAGGCAACAACATCCTTGT-----------------------------
     BnaGln1.4_C2 --------------------------------------------------CCCTCAAGCTATCTTCAAAGATCCATTCAGAAGAGGCAACAACATCCTTGT-----------------------------
        Consensus ..................................................CCCTCAAGCTATCTTCAAAGATCCATTCAGAAGAGGCAACAACATCCTTGT.............................

                  781     790       800       810       820       830       840       850       860       870       880       890       900      910
                  |--------+---------+---------+---------+---------+---------+---------+---------+---------+---------+---------+---------+---------|
    BnaC.GLN1.4.a CTCTTGGAATTTGTTGCAATATTCATTTATTTTGTTTCTGTGATTGATTTGGAATTAGGTGATGTGTGACGCTTATACACCGGCTGGCGAACCGATCCCAACCAACAAAAGGCATGCGGCGGCTAAGATC
mRNA.BnaC.GLN1.4.a ------------------------------------------------------------GATGTGTGACGCTTATACACCGGCTGGCGAACCGATCCCAACCAACAAAAGGCATGCGGCGGCTAAGATC
     BnaGln1.4_C2 ------------------------------------------------------------GATGTGTGACGCTTATACACCGGCTGGCGAACCGATCCCAACCAACAAAAGGCATGCGGCGGCTAAGATC
        Consensus ............................................................GATGTGTGACGCTTATACACCGGCTGGCGAACCGATCCCAACCAACAAAAGGCATGCGGCGGCTAAGATC

                  911     920       930       940       950       960       970       980       990       1000      1010      1020      1030    1040
                  |--------+---------+---------+---------+---------+---------+---------+---------+---------+---------+---------+---------+---------|
    BnaC.GLN1.4.a TTTAGCGACCCAACCGTTGCCGCCGAAGAAACATGGTATACATCTTCACAATGAAATTTTTTTTGTCAAAGTTGACCTCAATTTTTTTGTTTGAAAACTTTATGTTTGCATAGTTAATTTTTTGTCAACA
mRNA.BnaC.GLN1.4.a TTTAGCGACCCAACCGTTGCCGCCGAAGAAACATGGTAT-------------------------------------------------------------------------------------------
     BnaGln1.4_C2 TTTAGCGACCCAACCGTTGCCGCCGAAGAAACATGGTAT-------------------------------------------------------------------------------------------
        Consensus TTTAGCGACCCAACCGTTGCCGCCGAAGAAACATGGTAT...........................................................................................

                  1041    1050      1060      1070      1080      1090      1100      1110      1120      1130      1140      1150      1160    1170
                  |--------+---------+---------+---------+---------+---------+---------+---------+---------+---------+---------+---------+---------|
    BnaC.GLN1.4.a CCATCGTGCGAGTTTAATTCCAAATGGTACCGCAAGGAGACGTAACATGACGTAGTACTTTATTGATATATATTTTTATAACGTAAAAGTATAATAAAATAATTTGACCATAAACACATTTGTTACTCTA
mRNA.BnaC.GLN1.4.a ----------------------------------------------------------------------------------------------------------------------------------
     BnaGln1.4_C2 ----------------------------------------------------------------------------------------------------------------------------------
        Consensus ..................................................................................................................................

                  1171    1180      1190      1200      1210      1220      1230      1240      1250      1260      1270      1280      1290    1300
                  |--------+---------+---------+---------+---------+---------+---------+---------+---------+---------+---------+---------+---------|
    BnaC.GLN1.4.a AATCTACATTTTTCTAAATTAATTAAATATTTCAAAAATATATTGAAATTAAGGTATGGAATTGAGCAAGAGTATACTTTGCTTCAAAAGGATACCAAGTGGCCAGTTGGTTGGCCCGTCGGTGGCTTCC
mRNA.BnaC.GLN1.4.a ---------------------------------------------------------GGAATTGAGCAAGAGTATACTTTGCTTCAAAAGGATACCAAGTGGCCAGTTGGTTGGCCCGTCGGTGGCTTCC
     BnaGln1.4_C2 ---------------------------------------------------------GGAATTGAGCAAGAGTATACTTTGCTTCAAAAGGATACCAAGTGGCCAGTTGGTTGGCCCGTCGGTGGCTTCC
        Consensus .........................................................GGAATTGAGCAAGAGTATACTTTGCTTCAAAAGGATACCAAGTGGCCAGTTGGTTGGCCCGTCGGTGGCTTCC

                  1301    1310      1320      1330      1340      1350      1360      1370      1380      1390      1400      1410      1420    1430
                  |--------+---------+---------+---------+---------+---------+---------+---------+---------+---------+---------+---------+---------|
    BnaC.GLN1.4.a CAGGTCCTCAGGTAAAGATTTTTACGGCGAATCTTTTCACTTCTTGATCTCAATCCAAAACTCCAGTTAGTGGCAAATAAATATATAAATATTTATTAATAAAAAAACAGGGACCATACTACTGTGGAGT
mRNA.BnaC.GLN1.4.a CAGGTCCTCAGG---------------------------------------------------------------------------------------------------GACCATACTACTGTGGAGT
     BnaGln1.4_C2 CAGGTCCTCAGG---------------------------------------------------------------------------------------------------GACCATACTACTGTGGAGT
        Consensus CAGGTCCTCAGG...................................................................................................GACCATACTACTGTGGAGT

                  1431    1440      1450      1460      1470      1480      1490      1500      1510      1520      1530      1540      1550    1560
                  |--------+---------+---------+---------+---------+---------+---------+---------+---------+---------+---------+---------+---------|
    BnaC.GLN1.4.a TGGAGCAGACAAAGCCTTTGGAAGAGACATCGTTGATGCTCATTACAAAGCATGTCTTTACGCTGGAATCAATGTCAGTGGCACTAACGGAGAAGTCATGCCCGGACAGGTTTAATATAATTCCCTTTTA
mRNA.BnaC.GLN1.4.a TGGAGCAGACAAAGCCTTTGGAAGAGACATCGTTGATGCTCATTACAAAGCATGTCTTTACGCTGGAATCAATGTCAGTGGCACTAACGGAGAAGTCATGCCCGGACAG---------------------
     BnaGln1.4_C2 TGGAGCAGACAAAGCCTTTGGAAGAGACATCGTTGATGCTCATTACAAAGCATGTCTTTACGCTGGAATCAATGTCAGTGGCACTAACGGAGAAGTCATGCCCGGACAG---------------------
        Consensus TGGAGCAGACAAAGCCTTTGGAAGAGACATCGTTGATGCTCATTACAAAGCATGTCTTTACGCTGGAATCAATGTCAGTGGCACTAACGGAGAAGTCATGCCCGGACAG.....................

                  1561    1570      1580      1590      1600      1610      1620      1630      1640      1650      1660      1670      1680    1690
                  |--------+---------+---------+---------+---------+---------+---------+---------+---------+---------+---------+---------+---------|
    BnaC.GLN1.4.a TTCTTCTGATTTAATATCATATTGATCGCGATCAGACCGTGAAACTGATATTGTGTGGGGTTTAGTGGGAATTCCAAGTCGGTCCAACCGTTGGAATAGCTGCAGCCGATCAGGTCTGGGTCGCTCGTTA
mRNA.BnaC.GLN1.4.a -----------------------------------------------------------------TGGGAATTCCAAGTCGGTCCAACCGTTGGAATAGCTGCAGCCGATCAGGTCTGGGTCGCTCGTTA
     BnaGln1.4_C2 -----------------------------------------------------------------TGGGAATTCCAAGTCGGTCCAACCGTTGGAATAGCTGCAGCCGATCAGGTCTGGGTCGCTCGTTA
        Consensus .................................................................TGGGAATTCCAAGTCGGTCCAACCGTTGGAATAGCTGCAGCCGATCAGGTCTGGGTCGCTCGTTA

                  1691    1700      1710      1720      1730      1740      1750      1760      1770      1780      1790      1800      1810    1820
                  |--------+---------+---------+---------+---------+---------+---------+---------+---------+---------+---------+---------+---------|
    BnaC.GLN1.4.a CATCCTCGAGGTATATATAACACACACTCATCAAGACCTGTCTCTTCATATACTCTGATTTATGACTATGTTTCTTGGGTATGTTAAAAACAGAGGATCACAGAACTGGCTGGAGTTGTTTTATCTCTTG
mRNA.BnaC.GLN1.4.a CATCCTCGAG-----------------------------------------------------------------------------------AGGATCACAGAACTGGCTGGAGTTGTTTTATCTCTTG
     BnaGln1.4_C2 CATCCTCGAG-----------------------------------------------------------------------------------AGGATCACAGAACTGGCTGGAGTTGTTTTATCTCTTG
        Consensus CATCCTCGAG...................................................................................AGGATCACAGAACTGGCTGGAGTTGTTTTATCTCTTG

                  1821    1830      1840      1850      1860      1870      1880      1890      1900      1910      1920      1930      1940    1950
                  |--------+---------+---------+---------+---------+---------+---------+---------+---------+---------+---------+---------+---------|
    BnaC.GLN1.4.a ACCCTAAACCAATTCCGGTTAGCTTTACCATATTTTCAATTATTTACCAAATAAACCTTTGATTCTTTTAAAATTCACTGAATTACTCTATTTTGATTTTTATATTCTAGGGAGATTGGAATGGTGCAGG
mRNA.BnaC.GLN1.4.a ACCCTAAACCAATTCCGG---------------------------------------------------------------------------------------------GAGATTGGAATGGTGCAGG
     BnaGln1.4_C2 ACCCTAAACCAATTCCGG---------------------------------------------------------------------------------------------GAGATTGGAATGGTGCAGG
```

```
      Consensus  ACCCTAAACCAATTCCGG.............................................................................................GAGATTGGAATGGTGCAGG

                 1951 1960      1970      1980      1990      2000      2010      2020      2030      2040      2050      2060      2070      2080
                 |--------+---------+---------+---------+---------+---------+---------+---------+---------+---------+---------+---------+---------|
   BnaC.GLN1.4.a  AGCACACACAAATTACAGGTCTGCTACGATTCCGTTTATATGTTTTTTCTCTGTGTTTTTTTTTTGTTTTCTGAATTTGGTTTTCTTGTTTCATATAGTACGAAGTCGATGAGAGAAGATGGAGGGTACG
mRNA.BnaC.GLN1.4.a  AGCACACACAAATTACAG--------------------------------------------------------------------------------TACGAAGTCGATGAGAGAAGATGGAGGGTACG
    BnaGln1.4_C2  AGCACACACAAATTACAG--------------------------------------------------------------------------------TACGAAGTCGATGAGAGAAGATGGAGGGTACG
      Consensus  AGCACACACAAATTACAG................................................................................TACGAAGTCGATGAGAGAAGATGGAGGGTACG

                 2081 2090      2100      2110      2120      2130      2140      2150      2160      2170      2180      2190      2200      2210
                 |--------+---------+---------+---------+---------+---------+---------+---------+---------+---------+---------+---------+---------|
   BnaC.GLN1.4.a  AGATCATAAAGAAAGCGATAGAGAAGCTTGGACTTCGTCACAAGGAACACATTGCTGCTTATGGTGAAGGCAACGAGCGTCGTCTCACTGGAAAACACGAGACTGCTGATATCAACACTTTCTTATGGGT
mRNA.BnaC.GLN1.4.a  AGATCATAAAGAAAGCGATAGAGAAGCTTGGACTTCGTCACAAGGAACACATTGCTGCTTATGGTGAAGGCAACGAGCGTCGTCTCACTGGAAAACACGAGACTGCTGATATCAACACTTTCTTATGGG-
    BnaGln1.4_C2  AGATCATAAAGAAAGCGATAGAGAAGCTTGGACTTCGTCACAAGGAACACATTGCTGCTTATGGTGAAGGCAACGAGCGTCGTCTCACTGGAAAACACGAGACTGCTGATATCAACACTTTCTTATGGG-
      Consensus  AGATCATAAAGAAAGCGATAGAGAAGCTTGGACTTCGTCACAAGGAACACATTGCTGCTTATGGTGAAGGCAACGAGCGTCGTCTCACTGGAAAACACGAGACTGCTGATATCAACACTTTCTTATGGG.

                 2211 2220      2230      2240      2250      2260      2270      2280      2290      2300      2310      2320      2330      2340
                 |--------+---------+---------+---------+---------+---------+---------+---------+---------+---------+---------+---------+---------|
   BnaC.GLN1.4.a  AAGAAGACTTTTCTTTAAAATGTATCATTGAATATTACTCCACATTTACACGTTACAGTATGTGAAAGTTGATTATTATATCACATATGCCTAAAGCATGATATAACTTAAACCGGTTTTGTACAGGGTG
mRNA.BnaC.GLN1.4.a  -------------------------------------------------------------------------------------------------------------------------------GTG
    BnaGln1.4_C2  -------------------------------------------------------------------------------------------------------------------------------GTG
      Consensus  ...............................................................................................................................GTG

                 2341 2350      2360      2370      2380      2390      2400      2410      2420      2430      2440      2450      2460      2470
                 |--------+---------+---------+---------+---------+---------+---------+---------+---------+---------+---------+---------+---------|
   BnaC.GLN1.4.a  TTGCAAACCGTGGGGCATCGATTAGGGTTGGTCGTGACACTGAGAAAGATGGGAAAGGATACTTTGAAGATCGTAGGCCAGCGTCGAACATGGATCCATACACTGTAACCTCCATGGTTGCTGAAACCAC
mRNA.BnaC.GLN1.4.a  TTGCAAACCGTGGGGCATCGATTAGGGTTGGTCGTGACACTGAGAAAGATGGGAAAGGATACTTTGAAGATCGTAGGCCAGCGTCGAACATGGATCCATACACTGTAACCTCCATGGTTGCTGAAACCAC
    BnaGln1.4_C2  TTGCAAACCGTGGGGCATCGATTAGGGTTGGTCGTGACACTGAGAAAGATGGGAAAGGATACTTTGAAGATCGTAGGCCAGCGTCGAACATGGATCCATACACTGTAACCTCCATGGTTGCTGAAACCAC
      Consensus  TTGCAAACCGTGGGGCATCGATTAGGGTTGGTCGTGACACTGAGAAAGATGGGAAAGGATACTTTGAAGATCGTAGGCCAGCGTCGAACATGGATCCATACACTGTAACCTCCATGGTTGCTGAAACCAC

                 2471 2480      2490      2500      2510      2520      2530      2540      2550      2560      2570      2580      2590      2600
                 |--------+---------+---------+---------+---------+---------+---------+---------+---------+---------+---------+---------+---------|
   BnaC.GLN1.4.a  AATCCTCTGGAAACCATGAATGAGGAATAGACAATAGAAGAATCAATATGAAACAGTTCTCATGTGTTCTTTGTTTAAGAATGTTTGATTTAAACTCTTTCCAAGAAATAATGATAGTCTTTTCTCTAGC
mRNA.BnaC.GLN1.4.a  AATCCTCTGGAAACCATGAATGAGGAATAGACAATAGAAGAATCAATATGAAACAGTTCTCATGTGTTCTTTGTTTAAGAATGTTTGATTTAAACTCTTTCCAAGAAATAATGATAGTCTTTTCTCTAGC
    BnaGln1.4_C2  AATCCTCTGGAAACCATGAATGAGGAATAGACAATAGAAGAATCAATATGAAACAGTTCTCATGTGTTCTTTGTTTAAGAATGTTTGATTTAAACTCTTTCCAAGAAATAATGATAGTCTTT-CTCTAGC
      Consensus  AATCCTCTGGAAACCATGAATGAGGAATAGACAATAGAAGAATCAATATGAAACAGTTCTCATGTGTTCTTTGTTTAAGAATGTTTGATTTAAACTCTTTCCAAGAAATAATGATAGTCTTTtCTCTAGC

                 2601 2610      2620      2630      2640      2650      2660      2670      2680      2690      2700      2710      2720      2730
                 |--------+---------+---------+---------+---------+---------+---------+---------+---------+---------+---------+---------+---------|
   BnaC.GLN1.4.a  TTTTACTTTGTTGTATTTCAGAATCTATATGTATTTACAAGATTCAAGTCTCGGTTTATTCAGTTTTTGACATGAGCCAAAACCGAAGTTTACAAACTTCTCCGTTACTCTGTGGCCCAAACTAGACCCG
mRNA.BnaC.GLN1.4.a  TTTTACTTTGTTGTATTTCAGAATCTATATGTATTTACAAGATTCAAGTCTCGGTTTATTCAGTTTTTGACATGAGCCAAAACCGAAGTTTACAAACTTCTCCGTTACTCTGTGGCCCAAACTAGACCCG
    BnaGln1.4_C2  TTTTACTTTGT
      Consensus  TTTTACTTTGTtgtatttcagaatctatatgtatttacaagattcaagtctcggtttattcagtttttgacatgagccaaaaccgaagtttacaaacttctccgttactctgtggcccaaactagacccg

                 2731 2740      2750   2757
                 |--------+---------+------|
   BnaC.GLN1.4.a  ACTCGAGGGTAACCGAAGATGGATAAA
mRNA.BnaC.GLN1.4.a  ACTCGAGGGTAACCGAAGATGGATAAA
    BnaGln1.4_C2
      Consensus  actcgagggtaaccgaagatggataaa
```

```
                  1       10        20        30        40        50        60        70        80        90        100       110       120       130
BnaA.GLN1.4.b     TATATATACACTTGCAGGAATCTCTTTAGTGTTATACAAAACAAAGCTAATTATTTTTTTTAGATTCCTAGAGAAAATGTTGGCACTTGCAGATTTGATCAATCTCGATCTCTCCGATTCCACTGAGAAG
mRNA.BnaA.GLN1.4.b TATATATACACTTGCAGGAATCTCTTTAGTGTTATACAAAACAAAGCTAATTATTTTTTTTAGATTCCTAGAGAAAATGTTGGCACTTGCAGATTTGATCAATCTCGATCTCTCCGATTCCACTGAGAAG
BnaGln1.4_C4                                                                               AAAATGTCGGCACTTGCAGATTTGATCAATCTCGATCTCTCCGATTCCACTGAGAAG
Consensus         tatatatacacttgcaggaatctctttagtgttatacaaaacaaagctaattattttttttagattcctagagAAAATGTtGGCACTTGCAGATTTGATCAATCTCGATCTCTCCGATTCCACTGAGAAG

                  131     140       150       160       170       180       190       200       210       220       230       240       250       260
BnaA.GLN1.4.b     ATCATTGCGGAGTACATATGGTCCGTACAATTCTTCATTCACCTCTGTCAAATAATATTCAGTCTATATTAGTATATGGATGATTTTGGTTGATCTTTGCTTCTGTGTTTGAGTTGTGTTTGTTTCTAAA
mRNA.BnaA.GLN1.4.b ATCATTGCGGAGTACATATGG---------------------------------------------------------------------------------------------------------------
BnaGln1.4_C4      ATCATTGCGGAGTACATATGG---------------------------------------------------------------------------------------------------------------
Consensus         ATCATTGCGGAGTACATATGG...............................................................................................................

                  261     270       280       290       300       310       320       330       340       350       360       370       380       390
BnaA.GLN1.4.b     CGTCTCTTCCTTGAGGTTTTCTTGAATATGTATGAGTTAAACTTTTAAACAATATACTAGAACGTTGAAAGACTTTGGAATGATTGAACCTTCTGGTGAATATATTGTGGTTTTAATGAATAGGATTGGT
mRNA.BnaA.GLN1.4.b ----------------------------------------------------------------------------------------------------------------------------ATTGGT
BnaGln1.4_C4      ----------------------------------------------------------------------------------------------------------------------------ATTGGT
Consensus         ............................................................................................................................ATTGGT

                  391     400       410       420       430       440       450       460       470       480       490       500       510       520
BnaA.GLN1.4.b     GGATCAGGCTTGGATATGAGAAGCAAAGCAAGGGTAACTATATTATTTTTTGTTTGTTTTTTAGAGCATAAAACGATGAATACATCTTTGAAATAATGTTAAGAACATAAGAAAGAATGATTATATTCAT
mRNA.BnaA.GLN1.4.b GGATCAGGCTTGGATATGAGAAGCAAAGCAAGG-------------------------------------------------------------------------------------------------
BnaGln1.4_C4      GGATCAGGCTTGGATATGAGAAGCAAAGCAAGG-------------------------------------------------------------------------------------------------
Consensus         GGATCAGGCTTGGATATGAGAAGCAAAGCAAGG.................................................................................................

                  521     530       540       550       560       570       580       590       600       610       620       630       640       650
BnaA.GLN1.4.b     TATTATTGCAGACTTTGCCCGGACCAGTGAAGGATCCATCGGAGTTACCGAAATGGAACTATGACGGTTCAAGCACCGGCCAAGCTCCCGGCAGCGACAGTGAAGTCATCCTCTAGTATGTTTCAATATA
mRNA.BnaA.GLN1.4.b -----------ACTTTGCCCGGACCAGTGAAGGATCCATCGGAGTTACCGAAATGGAACTATGACGGTTCAAGCACCGGCCAAGCTCCCGGCAGCGACAGTGAAGTCATCCTCTA---------------
BnaGln1.4_C4      -----------ACTTTGCCCGGACCAGTGAAGGATCCATCGGAGTTACCGAAATGGAACTATGACGGTTCAAGCACCGGCCAAGCTCCCGGCAGCGACAGTGAAGTCATCCTCTA---------------
Consensus         ...........ACTTTGCCCGGACCAGTGAAGGATCCATCGGAGTTACCGAAATGGAACTATGACGGTTCAAGCACCGGCCAAGCTCCCGGCAGCGACAGTGAAGTCATCCTCTA...............

                  651     660       670       680       690       700       710       720       730       740       750       760       770       780
BnaA.GLN1.4.b     AACTAATACATTTAATAATCTAATTAAAATTTGTAAATCGTGGATTAATAAGATTGTGGTGTCTCTATGTTTTAGCCCTCAAGCTATCTTCAAAGACCCCTTCAGAAGAGGCAACAACATCCTTGTAATAA
mRNA.BnaA.GLN1.4.b ---------------------------------------------------------------------------CCCTCAAGCTATCTTCAAAGACCCCTTCAGAAGAGGCAACAACATCCTTGT-----
BnaGln1.4_C4      ---------------------------------------------------------------------------CCCTCAAGCTATCTTCAAAGACCCCTTCAGAAGAGGCAACAACATCCTTGT-----
Consensus         ...........................................................................CCCTCAAGCTATCTTCAAAGACCCCTTCAGAAGAGGCAACAACATCCTTGT.....

                  781     790       800       810       820       830       840       850       860       870       880       890       900       910
BnaA.GLN1.4.b     TTCAATTACACAATGAAAACAATAATCTTCAAATTTGTTATATTATATATATATATATATATATTTTTGTTTTGTTTTTGCGATTGATTTGGAAATTAGGTGATGTGTGATGCATATACACCGGCCGGCGAA
mRNA.BnaA.GLN1.4.b ----------------------------------------------------------------------------------------------------GATGTGTGATGCATATACACCGGCCGGCGAA
BnaGln1.4_C4      ----------------------------------------------------------------------------------------------------GATGTGTGATGCATATACACCGGCCGGCGAA
Consensus         ....................................................................................................GATGTGTGATGCATATACACCGGCCGGCGAA

                  911     920       930       940       950       960       970       980       990       1000      1010      1020      1030      1040
BnaA.GLN1.4.b     CCGATCCCGACAAACAAAAGGCATGCGGCGGCCAAGATCTTTAGCGACCCGAGCGTTGCCGCCGAAGAAACATGGTATTTATCTTTTAAATGAAATATTTAGTCAAAGTTTTATTTCAATTGTTTGCCAA
mRNA.BnaA.GLN1.4.b CCGATCCCGACAAACAAAAGGCATGCGGCGGCCAAGATCTTTAGCGACCCGAGCGTTGCCGCCGAAGAAACATGGTAT----------------------------------------------------
BnaGln1.4_C4      CCGATCCCGACAAACAAAAGGCATGCGGCGGCCAAGATCTTTAGCGACCCGAGCGTTGCCGCCGAAGAAACATGGTAT----------------------------------------------------
Consensus         CCGATCCCGACAAACAAAAGGCATGCGGCGGCCAAGATCTTTAGCGACCCGAGCGTTGCCGCCGAAGAAACATGGTAT....................................................

                  1041    1050      1060      1070      1080      1090      1100      1110      1120      1130      1140      1150      1160      1170
BnaA.GLN1.4.b     AAAAAGAAGAAGTTCATTTCAATTTTTGTTTGAAAACTACTAGTTTAAGTTTGCATACTCCCTTATATTTGTTGACATCATCATGCTAGTTTTAAATGTAAATGGTAATAGCATGTCCTAGTCAACTTTG
mRNA.BnaA.GLN1.4.b ----------------------------------------------------------------------------------------------------------------------------------
BnaGln1.4_C4      ----------------------------------------------------------------------------------------------------------------------------------
Consensus         ..................................................................................................................................

                  1171    1180      1190      1200      1210      1220      1230      1240      1250      1260      1270      1280      1290      1300
BnaA.GLN1.4.b     CATAGTTGCTACATTTTCTTATAACTTAAAAATATAATAAAAGTAAATTGATCATAAACAAACTGGTTAGTCTAAATTTTCAGGATGACATTAGTGACAATATATTTTCATTTTTGGTTGTAATTATTTT
mRNA.BnaA.GLN1.4.b ----------------------------------------------------------------------------------------------------------------------------------
BnaGln1.4_C4      ----------------------------------------------------------------------------------------------------------------------------------
Consensus         ..................................................................................................................................

                  1301    1310      1320      1330      1340      1350      1360      1370      1380      1390      1400      1410      1420      1430
BnaA.GLN1.4.b     CAGTAACCAATCCTTTTTTTATTATTGAAATTTAGGTATGGAATTGAGCAAGAGTATACTTTGCTACAAAAGGATATTAAATGGCCGGTAGGTTGGCCTGTTGGCGGCTTCCCAGGTCCTCAGGTAATGA
mRNA.BnaA.GLN1.4.b ---------------------------------------GGAATTGAGCAAGAGTATACTTTGCTACAAAAGGATATTAAATGGCCGGTAGGTTGGCCTGTTGGCGGCTTCCCAGGTCCTCAGG------
BnaGln1.4_C4      ---------------------------------------GGAATTGAGCAAGAGTATACTTTGCTACAAAAGGATATTAAATGGCCGGTAGGTTGGCCTGTTGGCGGCTTCCCAGGTCCTCAGG------
Consensus         .......................................GGAATTGAGCAAGAGTATACTTTGCTACAAAAGGATATTAAATGGCCGGTAGGTTGGCCTGTTGGCGGCTTCCCAGGTCCTCAGG......

                  1431    1440      1450      1460      1470      1480      1490      1500      1510      1520      1530      1540      1550      1560
BnaA.GLN1.4.b     CTTTTTCGGCAAATCTATTCACATTAACCTACCCTTTCGTTATTGAGTTTTAACCCAAAAATAATAATGAAAAATCAAAAAACAGGGACCGTACTACTGTGGTGCTGGAGCAGACAAAGCCTTTGGAAGA
mRNA.BnaA.GLN1.4.b --------------------------------------------------------------------------------------GACCGTACTACTGTGGTGCTGGAGCAGACAAAGCCTTTGGAAGA
BnaGln1.4_C4      --------------------------------------------------------------------------------------GACCGTACTACTGTGGTGCTGGAGCAGACAAAGCCTTTGGAAGA
Consensus         ......................................................................................GACCGTACTACTGTGGTGCTGGAGCAGACAAAGCCTTTGGAAGA

                  1561    1570      1580      1590      1600      1610      1620      1630      1640      1650      1660      1670      1680      1690
BnaA.GLN1.4.b     GACATAGTGGATTCTCATTACAAAGCCTGTCTTTACGCCGGAATCAATGTCAGTGGCACTAACGGAGAAGTCATGCCCGGACAGGTTTAAAACTTACCAGTCACTCTTTTTTATTCTTCCGATTCATTAT
mRNA.BnaA.GLN1.4.b GACATAGTGGATTCTCATTACAAAGCCTGTCTTTACGCCGGAATCAATGTCAGTGGCACTAACGGAGAAGTCATGCCCGGACAG----------------------------------------------
BnaGln1.4_C4      GACATAGTGGATTCTCATTACAAAGCCTGTCTTTACGCCGGAATCAATGTCAGTGGCACTAACGGAGAAGTCATGCCCGGACAG----------------------------------------------
Consensus         GACATAGTGGATTCTCATTACAAAGCCTGTCTTTACGCCGGAATCAATGTCAGTGGCACTAACGGAGAAGTCATGCCCGGACAG..............................................

                  1691    1700      1710      1720      1730      1740      1750      1760      1770      1780      1790      1800      1810      1820
BnaA.GLN1.4.b     CATATTGATTGAGTTCAGAACAAGAAATCAAACTGTTTAAACAAATCTTGTGTGGGCTTCAGTGGGAGTTCCAAGTCGGTCCAACCGTTGGAATCGCTGCCGCCGATCAGGTCTGGGTCGCTCGTTACAT
mRNA.BnaA.GLN1.4.b --------------------------------------------------------------TGGGAGTTCCAAGTCGGTCCAACCGTTGGAATCGCTGCCGCCGATCAGGTCTGGGTCGCTCGTTACAT
BnaGln1.4_C4      --------------------------------------------------------------TGGGAGTTCCAAGTCGGTCCAACCGTTGGAATCGCTGCCGCCGATCAGGTCTGGGTCGCTCGTTACAT
Consensus         ..............................................................TGGGAGTTCCAAGTCGGTCCAACCGTTGGAATCGCTGCCGCCGATCAGGTCTGGGTCGCTCGTTACAT

                  1821    1830      1840      1850      1860      1870      1880      1890      1900      1910      1920      1930      1940      1950
BnaA.GLN1.4.b     CCTCGAGGTATATATAATAAACACACTCATGGTTATGTACACCCTGTAACCTTATCTCTTCATATAATCTGTTTTTATGACTCTTTTCTTGGTTATGTGAAAACAGAGGATCACAGAATTGGCTGGAGTT
mRNA.BnaA.GLN1.4.b CCTCGAG-----------------------------------------------------------------------------------------------------AGGATCACAGAATTGGCTGGAGTT
BnaGln1.4_C4      CCTCGAG-----------------------------------------------------------------------------------------------------AGGATCACAGAATTGGCTGGAGTT
```

```
     Consensus CCTCGAG...................................................................................................AGGATCACAGAATTGGCTGGAGTT

               1951 1960      1970      1980      1990      2000      2010      2020      2030      2040      2050      2060      2070      2080
               |--------+---------+---------+---------+---------+---------+---------+---------+---------+---------+---------+---------+---------|
 BnaA.GLN1.4.b GTTCTGTCTCTTGACCCAAAACCAATTCCGGTTTATTTTTTACCATAAATTTAAATATTATAACCAAAAAGTTTTTTTTTCAAATTCATTCAATTTTTCTATTTTAGGGAGATTGGAATGGTGCAGGAG
mRNA.BnaA.GLN1.4.b GTTCTGTCTCTTGACCCAAAACCAATTCCGG------------------------------------------------------------------------------GAGATTGGAATGGTGCAGGAG
  BnaGln1.4_C4 GTTCTGTCTCTTGACCCAAAACCAATTCCGG------------------------------------------------------------------------------GAGATTGGAATGGTGCAGGAG
     Consensus GTTCTGTCTCTTGACCCAAAACCAATTCCGG..............................................................................GAGATTGGAATGGTGCAGGAG

               2081 2090      2100      2110      2120      2130      2140      2150      2160      2170      2180      2190      2200      2210
               |--------+---------+---------+---------+---------+---------+---------+---------+---------+---------+---------+---------+---------|
 BnaA.GLN1.4.b CACACACAAATTACAGGTTCTTTGTGTTTTCAAGATCTGCTCTGATTCATTTTATATGGTTTGTGTTTGTTTTTCTGAATTGGGTTTCTTGTATTCTGCAGTACAAAGTCCATGAGAGAAGATGGAGGGT
mRNA.BnaA.GLN1.4.b CACACACAAATTACAG-------------------------------------------------------------------------------------TACAAAGTCCATGAGAGAAGATGGAGGGT
  BnaGln1.4_C4 CACACACAAATTACAG-------------------------------------------------------------------------------------TACAAAGTCCATGAGAGAAGATGGAGGGT
     Consensus CACACACAAATTACAG.....................................................................................TACAAAGTCCATGAGAGAAGATGGAGGGT

               2211 2220      2230      2240      2250      2260      2270      2280      2290      2300      2310      2320      2330      2340
               |--------+---------+---------+---------+---------+---------+---------+---------+---------+---------+---------+---------+---------|
 BnaA.GLN1.4.b ACGAGATCATAAAGAAAGCGATAGAGAAGCTTGGATTGCGTCACAAGGAACACATCTCTGCTTATGGTGAAGGCAACGAGCGTCGTCTCACTGGCAAACACGAGACTGCCGATATCAACACTTTCTTATG
mRNA.BnaA.GLN1.4.b ACGAGATCATAAAGAAAGCGATAGAGAAGCTTGGATTGCGTCACAAGGAACACATCTCTGCTTATGGTGAAGGCAACGAGCGTCGTCTCACTGGCAAACACGAGACTGCCGATATCAACACTTTCTTATG
  BnaGln1.4_C4 ACGAGATCATAAAGAAAGCGATAGAGAAGCTTGGATTGCGTCACAAGGAACACATCTCTGCTTATGGTGAAGGCAACGAGCGTCGTCTCACTGGCAAACACGAGACTGCCGATATCAACACTTTCTTATG
     Consensus ACGAGATCATAAAGAAAGCGATAGAGAAGCTTGGATTGCGTCACAAGGAACACATCTCTGCTTATGGTGAAGGCAACGAGCGTCGTCTCACTGGCAAACACGAGACTGCCGATATCAACACTTTCTTATG

               2341 2350      2360      2370      2380      2390      2400      2410      2420      2430      2440      2450      2460      2470
               |--------+---------+---------+---------+---------+---------+---------+---------+---------+---------+---------+---------+---------|
 BnaA.GLN1.4.b GGTAAGAGTTTTCTTTCATAGCATGACAAAGTGTGCATTCATATCACATGAGCCTAAGTCATGCATGATCTAACTTAACTGGTTTGTACAGGGTGTGGCCAACCGTGGGGCATCGATTAGGGTTGGTCGG
mRNA.BnaA.GLN1.4.b GG------------------------------------------------------------------------------------------GTGTGGCCAACCGTGGGGCATCGATTAGGGTTGGTCGG
  BnaGln1.4_C4 GG------------------------------------------------------------------------------------------GTGTGGCCAACCGTGGGGCATCGATTAGGGTTGGTCGG
     Consensus GG..........................................................................................GTGTGGCCAACCGTGGGGCATCGATTAGGGTTGGTCGG

               2471 2480      2490      2500      2510      2520      2530      2540      2550      2560      2570      2580      2590      2600
               |--------+---------+---------+---------+---------+---------+---------+---------+---------+---------+---------+---------+---------|
 BnaA.GLN1.4.b GACACTGAGCAAGCTGGGAAAGGGTACTTCGAAGATCGTAGGCCAGCGTCCAACATGGATCCCTACACTGTGACCTCCATGATTGCTGAAACTACAATCCTCTGGAAGCCATGAATGAAGAAAACTTGAG
mRNA.BnaA.GLN1.4.b GACACTGAGCAAGCTGGGAAAGGGTACTTCGAAGATCGTAGGCCAGCGTCCAACATGGATCCCTACACTGTGACCTCCATGATTGCTGAAACTACAATCCTCTGGAAGCCATGAATGAAGAAAACTTGAG
  BnaGln1.4_C4 GACACTGAGCAAGCTGGGAAAGGGTACTTCGAAGATCGTAGGCCAGCGTCCAACATGGATCCCTACACTGTGACCTCCATGATTGCTGAAACTACAATCCTCTGGAAGCCATGAATGAAGAAAACTTGAG
     Consensus GACACTGAGCAAGCTGGGAAAGGGTACTTCGAAGATCGTAGGCCAGCGTCCAACATGGATCCCTACACTGTGACCTCCATGATTGCTGAAACTACAATCCTCTGGAAGCCATGAATGAAGAAAACTTGAG

               2601 2610      2620      2630      2640      2650      2660      2670      2680      2690      2700      2710      2720      2730
               |--------+---------+---------+---------+---------+---------+---------+---------+---------+---------+---------+---------+---------|
 BnaA.GLN1.4.b CTCCTCCAAGGAACCTCTAATATCAGTTCATGTTCATTCTTCTATTGTCTCGTTCTGTCTTCTTTGTTTAAGTATGTTTGATTTAAACTCTTAGAACTTGTTCTATTTCTATATTATGTTATTATACAAA
mRNA.BnaA.GLN1.4.b CTCCTCCAAGGAACCTCTAATATCAGTTCATGTTCATTCTTCTATTGTCTCGTTCTGTCTTCTTTGTTTAAGTATGTTTGATTTAAACTCTTAGAACTTGTTCTATTTCTATATTATGTTATTATACAAA
  BnaGln1.4_C4 CTCCTCCAAGGA
     Consensus CTCCTCCAAGGAacctctaatatcagttcatgttcattcttctattgtctcgttctgtcttctttgtttaagtatgtttgatttaaactcttagaacttgttctatttctatattatgttattatacaaa

               2731 2740      2750      2760      2770      2780      2790      2800      2810      2820      2830      2840      2850      2860
               |--------+---------+---------+---------+---------+---------+---------+---------+---------+---------+---------+---------+---------|
 BnaA.GLN1.4.b TTCAAATATCAGTTTTAGAATATGAAGAGGATCTGAACTTTAAACTTCTTATAGAAACCAAAATCGCAAGTTGCAAGCTCCTCCGTTACTAGCCAACCCAAACTAGACCGGCCGAATCGACATTTAGCAC
mRNA.BnaA.GLN1.4.b TTCAAATATCAGTTTTAGAATATGAAGAGGATCTGAACTTTAAACTTCTTATAGAAACCAAAATCGCAAGTTGCAAGCTCCTCCGTTACTAGCCAACCCAAACTAGACCGGCCGAATCGACATTTAGCAC
  BnaGln1.4_C4
     Consensus ttcaaatatcagttttagaatatgaagaggatctgaactttaaacttcttatagaaaccaaaatcgcaagttgcaagctcctccgttactagccaacccaaactagaccggccgaatcgacatttagcac

               2861 2870      2880      2890      2900      2910      2920      2930      2940      2950      2960      2970      2980      2990
               |--------+---------+---------+---------+---------+---------+---------+---------+---------+---------+---------+---------+---------|
 BnaA.GLN1.4.b ACTCTATTTGGCCCATTTTAGCTTAATGGACTCAAATTTATAACTTTTAAATATTGAAACTCTGTGCTAAAAATATAATAATTTTGAAACTCTATTATACAATGAACTAACGTACTTAAACAAGAAGTAA
mRNA.BnaA.GLN1.4.b ACTCTATTTGGCCCATTTTAGCTTAATGGACTCAAATTTATAACTTTTAAATATTGAAACTCTGTGCTAAAAATATAATAATTTTGAAACTCTATTATACAATGAACTAACGTACTTAAACAAGAAGTAA
  BnaGln1.4_C4
     Consensus actctatttggcccattttagcttaatggactcaaatttataacttttaaatattgaaactctgtgctaaaaatataataattttgaaactctattatacaatgaactaacgtacttaaacaagaagtaa

               2991 3000      3010      3020      3030      3040      3050      3060      3070      3080      3090      3100      3110      3120
               |--------+---------+---------+---------+---------+---------+---------+---------+---------+---------+---------+---------+---------|
 BnaA.GLN1.4.b GAACACGCAGAGAGTACACTACCAATCAATTTATCTTATGCTACTTTGATTCTCGGAAACATTTTCTTGAAACTTCGATATTTCCTTTCTTTTCGTATTTGGTTTAAATTATTATGACTACTATTCATCT
mRNA.BnaA.GLN1.4.b GAACACGCAGAGAGTACACTACCAATCAATTTATCTTATGCTACTTTGATTCTCGGAAACATTTTCTTGAAACTTCGATATTTCCTTTCTTTTCGTATTTGGTTTAAATTATTATGACTACTATTCATCT
  BnaGln1.4_C4
     Consensus gaacacgcagagagtacactaccaatcaatttatcttatgctactttgattctcggaaacattttcttgaaacttcgatatttcctttcttttcgtatttggtttaaattattatgactactattcatct

               3121 3130      3140      3150      3160      3170      3180      3190      3200      3210      3220      3230      3240      3250
               |--------+---------+---------+---------+---------+---------+---------+---------+---------+---------+---------+---------+---------|
 BnaA.GLN1.4.b TAAGAAACTAACAAAATGAGTTCAGTTTGGATTCATTTTTCAGAATGTATGGGAAGAGAGTTTATTTTCTGTTCGTCTGACATTTTGTTGAAGTCAGGTATGAAATTAAAAACAAAAAAACTATTAGCTT
mRNA.BnaA.GLN1.4.b TAAGAAACTAACAAAATGAGTTCAGTTTGGATTCATTTTTCAGAATGTATGGGAAGAGAGTTTATTTTCTGTTCGTCTGACATTTTGTTGAAGTCAGGTATGAAATTAAAAACAAAAAAACTATTAGCTT
  BnaGln1.4_C4
     Consensus taagaaactaacaaaatgagttcagtttggattcatttttcagaatgtatgggaagagagtttattttctgttcgtctgacattttgttgaagtcaggtatgaaattaaaaacaaaaaaactattagctt

               3251 3260      3270      3280      3290      3300      3310      3320      3330      3340      3350      3360      3370      3380
               |--------+---------+---------+---------+---------+---------+---------+---------+---------+---------+---------+---------+---------|
 BnaA.GLN1.4.b TAAAGTATTATTTTATTCAAGGTTTGGTTTCTCATAATCGAGTTTAAATCCTAATTTTCTGTCCAGGAAAAAACTTCTAATTTGGTCATCAAAAATTTCAAGATATTTTTAATCAATTTTTCATCTACTT
mRNA.BnaA.GLN1.4.b TAAAGTATTATTTTATTCAAGGTTTGGTTTCTCATAATCGAGTTTAAATCCTAATTTTCTGTCCAGGAAAAAACTTCTAATTTGGTCATCAAAAATTTCAAGATATTTTTAATCAATTTTTCATCTACTT
  BnaGln1.4_C4
     Consensus taaagtattattttattcaaggtttggtttctcataatcgagtttaaatcctaattttctgtccaggaaaaaacttctaatttggtcatcaaaaatttcaagatatttttaatcaatttttcatctactt

               3381 3390      3400      3410      3420      3430      3440      3450      3460      3470      3480      3490      3500      3510
               |--------+---------+---------+---------+---------+---------+---------+---------+---------+---------+---------+---------+---------|
 BnaA.GLN1.4.b ATGATTTAAGATTGACAAAGTAATTTACAGCAGACTAAAATATTAACAGTTCAAAGCTATTACGAAATACGCCGATAGGACTAAAACATTTTTTTTCTAAAACATAGGACTAAAACTTTAATGCCAAAAT
mRNA.BnaA.GLN1.4.b ATGATTTAAGATTGACAAAGTAATTTACAGCAGACTAAAATATTAACAGTTCAAAGCTATTACGAAATACGCCGATAGGACTAAAACATTTTTTTTCTAAAACATAGGACTAAAACTTTAATGCCAAAAT
  BnaGln1.4_C4
     Consensus atgatttaagattgacaaagtaatttacagcagactaaaatattaacagttcaaagctattacgaaatacgccgataggactaaaacattttttttctaaaacataggactaaaactttaatgccaaaat

               3511 3520      3530      3540      3550      3560      3570      3580      3590      3600      3610      3620      3630      3640
               |--------+---------+---------+---------+---------+---------+---------+---------+---------+---------+---------+---------+---------|
 BnaA.GLN1.4.b CTAATGGTTGAGAGCTCTTCTTGCAATTCCGTGTTAAAACTTTCATTCATGTAACTAACTGGTAGTATACATCAGTTACCCATACAAGTTAAAGTAAAAAATCTTTATGTTAAGAACTTAAGATTGACAT
mRNA.BnaA.GLN1.4.b CTAATGGTTGAGAGCTCTTCTTGCAATTCCGTGTTAAAACTTTCATTCATGTAACTAACTGGTAGTATACATCAGTTACCCATACAAGTTAAAGTAAAAAATCTTTATGTTAAGAACTTAAGATTGACAT
  BnaGln1.4_C4
     Consensus ctaatggttgagagctcttcttgcaattccgtgttaaaactttcattcatgtaactaactggtagtatacatcagttacccatacaagttaaagtaaaaaatctttatgttaagaacttaagattgacat

               3641 3650      3660      3670      3680      3690      3700      3710      3720      3730      3740      3750      3760      3770
               |--------+---------+---------+---------+---------+---------+---------+---------+---------+---------+---------+---------+---------|
 BnaA.GLN1.4.b GCATGCATGTTTACATGTGTATATAAACACACACTTAGTTTGGAATTAAAGAAAAACACACATGTATATAAAGAATTGTTTGATCGTCTTTCTCCGAATAAGAGGCGAAACATAATCAACTTGAATTCGG
mRNA.BnaA.GLN1.4.b GCATGCATGTTTACATGTGTATATAAACACACACTTAGTTTGGAATTAAAGAAAAACACACATGTATATAAAGAATTGTTTGATCGTCTTTCTCCGAATAAGAGGCGAAACATAATCAACTTGAATTCGG
  BnaGln1.4_C4
     Consensus gcatgcatgtttacatgtgtatataaacacacacttagtttggaattaaagaaaaacacacatgtatataaagaattgtttgatcgtctttctccgaataagaggcgaaacataatcaacttgaattcgg

               3771 3780      3790      3800      3810      3820      3830      3840      3850      3860      3870      3880      3890      3900
               |--------+---------+---------+---------+---------+---------+---------+---------+---------+---------+---------+---------+---------|
 BnaA.GLN1.4.b GACAGCTATTCGAAATCTTAGTGAAAATTTTGATTTGTTATCTCATGTTTGCTTTTCGTGAAAAAATACCAATTGAAGTGGAGGGTTTCTTCAAACAACAAGGACCATAAACACACACACCTAGTTTGGA
mRNA.BnaA.GLN1.4.b GACAGCTATTCGAAATCTTAGTGAAAATTTTGATTTGTTATCTCATGTTTGCTTTTCGTGAAAAAATACCAATTGAAGTGGAGGGTTTCTTCAAACAACAAGGACCATAAACACACACACCTAGTTTGGA
```

```
[illegible]
     BnaGln1.4_C4
        Consensus  gacagctattcgaaatcttagtgaaaattttgatttgttatctcatgtttgcttttcgtgaaaaaataccaattgaagtggagggtttcttcaaacaacaaggaccataaacacacacacctagtttgga

                   3901  3910      3920      3930      3940      3950      3960      3970      3980      3990      4000      4010     4019
                   |--------+---------+---------+---------+---------+---------+---------+---------+---------+---------+---------+--------|
   BnaA.GLN1.4.b   ATTAAAGAAAAAAACACACACGCATATAGTTTAGGAGAAGGTTTGTTAACTGTGAAGTGAATGCGCCGAAGTTTTTTAAAAGTAAAAAGATAAGTAAATGCTTCAAGGATTGAAATAAA
mRNA.BnaA.GLN1.4.b ATTAAAGAAAAAAACACACACGCATATAGTTTAGGAGAAGGTTTGTTAACTGTGAAGTGAATGCGCCGAAGTTTTTTAAAAGTAAAAAGATAAGTAAATGCTTCAAGGATTGAAATAAA
     BnaGln1.4_C4
        Consensus  attaaagaaaaaaacacacacgcatatagtttaggagaaggtttgttaactgtgaagtgaatgcgccgaagttttttaaaagtaaaaagataagtaaatgcttcaaggattgaaataaa
```

```
                    1       10        20        30        40        50        60        70        80        90        100       110       120       130
                    |--------+---------+---------+---------+---------+---------+---------+---------+---------+---------+---------+---------+---------|
     BnaC.GLN1.4.b  TATATATACACTTGCAGGAATCTCTTTAGTGTTATACAAAACAAAGCTAATATTTTTTTTTAGATTCCTAGAGAAAATGTCGGCACTTGCAGATTTGATCAATCTCGATCTCTCCGATTACACTGAGAAG
mRNA.BnaC.GLN1.4.b  TATATATACACTTGCAGGAATCTCTTTAGTGTTATACAAAACAAAGCTAATATTTTTTTTTAGATTCCTAGAGAAAATGTCGGCACTTGCAGATTTGATCAATCTCGATCTCTCCGATTACACTGAGAAG
     BnaGln1.4_C3                                   GATACAAAACAAAGCTAATATTTTTTTTTAGATTCCTAGAGAAAATGTCGGCACTTGCAGATTTGATCAATCTCGATCTCTCCGATTACACTGAGAAG
         Consensus  tatatatacacttgcaggaatctctttagtgttATACAAAACAAAGCTAATATTTTTTTTTAGATTCCTAGAGAAAATGTCGGCACTTGCAGATTTGATCAATCTCGATCTCTCCGATTACACTGAGAAG

                    131     140       150       160       170       180       190       200       210       220       230       240       250       260
                    |--------+---------+---------+---------+---------+---------+---------+---------+---------+---------+---------+---------+---------|
     BnaC.GLN1.4.b  ATCATTGCGGAGTACATATGGTCCGTACAATTCATTCACCTCTATCAAATAATATTCAGTCTATATTAGTAGATGGATGATTTTGGTTGATCTTTGCCTTCTGTGTTTGAGTTGTGTTTGTTTCTAAACG
mRNA.BnaC.GLN1.4.b  ATCATTGCGGAGTACATATGG-------------------------------------------------------------------------------------------------------------
     BnaGln1.4_C3  ATCATTGCGGAGTACATATGG-------------------------------------------------------------------------------------------------------------
         Consensus  ATCATTGCGGAGTACATATGG.............................................................................................................

                    261     270       280       290       300       310       320       330       340       350       360       370       380       390
                    |--------+---------+---------+---------+---------+---------+---------+---------+---------+---------+---------+---------+---------|
     BnaC.GLN1.4.b  TCTCTTCCTTGAGTTTTTTTTGAATATGTATGAGTTAAAACTTTTAAACAATATACTAGAACGTTGAAAGACTTTGGACTGATTGAACCTTCTGGTTAATATATTGTGGTTTTAATGAATAGGATTGGTG
mRNA.BnaC.GLN1.4.b  ---------------------------------------------------------------------------------------------------------------------------ATTGGTG
     BnaGln1.4_C3  ---------------------------------------------------------------------------------------------------------------------------ATTGGTG
         Consensus  ...........................................................................................................................ATTGGTG

                    391     400       410       420       430       440       450       460       470       480       490       500       510       520
                    |--------+---------+---------+---------+---------+---------+---------+---------+---------+---------+---------+---------+---------|
     BnaC.GLN1.4.b  GATCAGGCTTGGATATGAGAAGCAAAGCAAGGGTAACTATATTATTTTTGTTTGTTTTTTAGAGCATAAAACGATGAATACATCTTTGAAATAATGTTAAGAACTTAAGCAAATGAATGATTATATTCAT
mRNA.BnaC.GLN1.4.b  GATCAGGCTTGGATATGAGAAGCAAAGCAAGG--------------------------------------------------------------------------------------------------
     BnaGln1.4_C3  GATCAGGCTTGGATATGAGAAGCAAAGCAAGG--------------------------------------------------------------------------------------------------
         Consensus  GATCAGGCTTGGATATGAGAAGCAAAGCAAGG..................................................................................................

                    521     530       540       550       560       570       580       590       600       610       620       630       640       650
                    |--------+---------+---------+---------+---------+---------+---------+---------+---------+---------+---------+---------+---------|
     BnaC.GLN1.4.b  TATTATTGCAGACTTTGCCCGGACCAGTGAAGGATCCATCGGAGTTACCGAAATGGAACTATGACGGTTCAAGCACCGGCCAAGCTCCCGGCAGTGACAGTGAAGTCATCCTCTAGTATGTTTCAATATA
mRNA.BnaC.GLN1.4.b  -----------ACTTTGCCCGGACCAGTGAAGGATCCATCGGAGTTACCGAAATGGAACTATGACGGTTCAAGCACCGGCCAAGCTCCCGGCAGTGACAGTGAAGTCATCCTCTA---------------
     BnaGln1.4_C3  -----------ACTTTGCCCGGACCAGTGAAGGATCCATCGGAGTTACCGAAATGGAACTATGACGGTTCAAGCACCGGCCAAGCTCCCGGCAGTGACAGTGAAGTCATCCTCTA---------------
         Consensus  ...........ACTTTGCCCGGACCAGTGAAGGATCCATCGGAGTTACCGAAATGGAACTATGACGGTTCAAGCACCGGCCAAGCTCCCGGCAGTGACAGTGAAGTCATCCTCTA...............

                    651     660       670       680       690       700       710       720       730       740       750       760       770       780
                    |--------+---------+---------+---------+---------+---------+---------+---------+---------+---------+---------+---------+---------|
     BnaC.GLN1.4.b  AATTAATACATATAATAAGCTAGATAAAATTGTAAATCGTGGATTAATAAGATTGTGGTTTCTCTATGTATTTTTTAGCCCTCAAGCTATCTTCAAAGACCCCTTCAGAAGAGGCAACAACATCCTTGTA
mRNA.BnaC.GLN1.4.b  ------------------------------------------------------------------------------CCCTCAAGCTATCTTCAAAGACCCCTTCAGAAGAGGCAACAACATCCTTGTA
     BnaGln1.4_C3  ------------------------------------------------------------------------------CCCTCAAGCTATCTTCAAAGACCCCTTCAGAAGAGGCAACAACATCCTTGTA
         Consensus  ..............................................................................CCCTCAAGCTATCTTCAAAGACCCCTTCAGAAGAGGCAACAACATCCTTGTA

                    781     790       800       810       820       830       840       850       860       870       880       890       900       910
                    |--------+---------+---------+---------+---------+---------+---------+---------+---------+---------+---------+---------+---------|
     BnaC.GLN1.4.b  ATAACTCAATTACACAATGAAAACAATAATATTCAAATTTGTTATATAAATTAAACATTTTTTTGTTTTTGGGATTGATTTGAAAATTAGGTAATGTGTGACGCATATACACCGGCCGGTGAACCGATTC
mRNA.BnaC.GLN1.4.b  AT---------------------------------------------------------------------------------------------GTGTGACGCATATACACCGGCCGGTGAACCGATTC
     BnaGln1.4_C3  AT---------------------------------------------------------------------------------------------GTGTGACGCATATACACCGGCCGGTGAACCGATTC
         Consensus  AT.............................................................................................GTGTGACGCATATACACCGGCCGGTGAACCGATTC

                    911     920       930       940       950       960       970       980       990       1000      1010      1020      1030      1040
                    |--------+---------+---------+---------+---------+---------+---------+---------+---------+---------+---------+---------+---------|
     BnaC.GLN1.4.b  CGACGAACAAAAGGCATGCTGCAGCTAAGATCTTTAGCGACCCCAGCGTTGCCGCCGAAGAAACATGGTATATATCTTCTAAATGAAATACTTAGTCGAAGTTTTATTTCAATTGTTTGCCAAAAAAAGA
mRNA.BnaC.GLN1.4.b  CGACGAACAAAAGGCATGCTGCAGCTAAGATCTTTAGCGACCCCAGCGTTGCCGCCGAAGAAACATGGTAT----------------------------------------------------------
     BnaGln1.4_C3  CGACGAACAAAAGGCATGCTGCAGCTAAGATCTTTAGCGACCCCAGCGTTGCCGCCGAAGAAACATGGTAT----------------------------------------------------------
         Consensus  CGACGAACAAAAGGCATGCTGCAGCTAAGATCTTTAGCGACCCCAGCGTTGCCGCCGAAGAAACATGGTAT..........................................................

                    1041  1050      1060      1070      1080      1090      1100      1110      1120      1130      1140      1150      1160      1170
                    |--------+---------+---------+---------+---------+---------+---------+---------+---------+---------+---------+---------+---------|
     BnaC.GLN1.4.b  AATTCATTTCAATTTTTGTTTGAAAACTACTAGTTTAAGTTTGCATACTCCCTTATATTTGTTGACATCATCATGCTAGTTTTAAATGTAAATGGTATAACATGCCCTAGTCAACTTTGATAGTTGTTAC
mRNA.BnaC.GLN1.4.b  ----------------------------------------------------------------------------------------------------------------------------------
     BnaGln1.4_C3  ----------------------------------------------------------------------------------------------------------------------------------
         Consensus  ..................................................................................................................................

                    1171  1180      1190      1200      1210      1220      1230      1240      1250      1260      1270      1280      1290      1300
                    |--------+---------+---------+---------+---------+---------+---------+---------+---------+---------+---------+---------+---------|
     BnaC.GLN1.4.b  ATTTTCTTATAACTTAAAAATATAATAAAAGTAAATTGATCATAAACACACTGGTTAGTCTAAATTTTCAGGATGACATTAGTGACAATAAATTTTCATTTTTGGTTGTAATTATTTTCAGTAACCATTC
mRNA.BnaC.GLN1.4.b  ----------------------------------------------------------------------------------------------------------------------------------
     BnaGln1.4_C3  ----------------------------------------------------------------------------------------------------------------------------------
         Consensus  ..................................................................................................................................

                    1301  1310      1320      1330      1340      1350      1360      1370      1380      1390      1400      1410      1420      1430
                    |--------+---------+---------+---------+---------+---------+---------+---------+---------+---------+---------+---------+---------|
     BnaC.GLN1.4.b  ATTATTGAAATTTAGGTATGGAATTGAGCAAGAGTATACTTTGCTTCAAAAGGATATTAAGTGGCCGGTAGGTTGGCCTGTTGGCGGCTTCCCAGGTCCTCAGGTAATGACTTTCAGCAAATCTATTCAC
mRNA.BnaC.GLN1.4.b  -------------------GGAATTGAGCAAGAGTATACTTTGCTTCAAAAGGATATTAAGTGGCCGGTAGGTTGGCCTGTTGGCGGCTTCCCAGGTCCTCAGG--------------------------
     BnaGln1.4_C3  -------------------GGAATTGAGCAAGAGTATACTTTGCTTCAAAAGGATATTAAGTGGCCGGTAGGTTGGCCTGTTGGCGGCTTCCCAGGTCCTCAGG--------------------------
         Consensus  ...................GGAATTGAGCAAGAGTATACTTTGCTTCAAAAGGATATTAAGTGGCCGGTAGGTTGGCCTGTTGGCGGCTTCCCAGGTCCTCAGG..........................

                    1431  1440      1450      1460      1470      1480      1490      1500      1510      1520      1530      1540      1550      1560
                    |--------+---------+---------+---------+---------+---------+---------+---------+---------+---------+---------+---------+---------|
     BnaC.GLN1.4.b  ATTAACGTACCCTTTCGTTATTGAGTTTCAACCCAAAAATAATAATGAAAAATCAGAAAACAGGGACCGTACTACTGTGGAGTTGGAGCAGACAAAGCCTTTGGAAGAGACATAGTAGATTCTCATTACA
mRNA.BnaC.GLN1.4.b  ----------------------------------------------------------------GACCGTACTACTGTGGAGTTGGAGCAGACAAAGCCTTTGGAAGAGACATAGTAGATTCTCATTACA
     BnaGln1.4_C3  ----------------------------------------------------------------GACCGTACTACTGTGGAGTTGGAGCAGACAAAGCCTTTGGAAGAGACATAGTAGATTCTCATTACA
         Consensus  ................................................................GACCGTACTACTGTGGAGTTGGAGCAGACAAAGCCTTTGGAAGAGACATAGTAGATTCTCATTACA

                    1561  1570      1580      1590      1600      1610      1620      1630      1640      1650      1660      1670      1680      1690
                    |--------+---------+---------+---------+---------+---------+---------+---------+---------+---------+---------+---------+---------|
     BnaC.GLN1.4.b  AAGCCTGTCTTTACGCCGGAATCAATGTCAGTGGCACTAACGGAGAAGTCATGCCCGGACAGGTTTAACACTTACCAGTCACTCTTTTTTATTCTTCCGATTCATTATCATATTGATTTGAGTTCAGAAC
mRNA.BnaC.GLN1.4.b  AAGCCTGTCTTTACGCCGGAATCAATGTCAGTGGCACTAACGGAGAAGTCATGCCCGGACAG--------------------------------------------------------------------
     BnaGln1.4_C3  AAGCCTGTCTTTACGCCGGAATCAATGTCAGTGGCACTAACGGAGAAGTCATGCCCGGACAG--------------------------------------------------------------------
         Consensus  AAGCCTGTCTTTACGCCGGAATCAATGTCAGTGGCACTAACGGAGAAGTCATGCCCGGACAG....................................................................

                    1691  1700      1710      1720      1730      1740      1750      1760      1770      1780      1790      1800      1810      1820
                    |--------+---------+---------+---------+---------+---------+---------+---------+---------+---------+---------+---------+---------|
     BnaC.GLN1.4.b  AAGAAATCAAACCGTTAAAACAAATCTTGTGTGGGTTTCAGTGGGAGTTCCAAGTCGGTCCAACGGTTGGAATCGCTGCCGCCGATCAAGTCTGGGTCGCTCGTTACATCCTCGAGGTATATATGATAAA
mRNA.BnaC.GLN1.4.b  -----------------------------------------TGGGAGTTCCAAGTCGGTCCAACGGTTGGAATCGCTGCCGCCGATCAAGTCTGGGTCGCTCGTTACATCCTCGAG--------------
     BnaGln1.4_C3  -----------------------------------------TGGGAGTTCCAAGTCGGTCCAACGGTTGGAATCGCTGCCGCCGATCAAGTCTGGGTCGCTCGTTACATCCTCGAG--------------
         Consensus  .........................................TGGGAGTTCCAAGTCGGTCCAACGGTTGGAATCGCTGCCGCCGATCAAGTCTGGGTCGCTCGTTACATCCTCGAG..............

                    1821  1830      1840      1850      1860      1870      1880      1890      1900      1910      1920      1930      1940      1950
                    |--------+---------+---------+---------+---------+---------+---------+---------+---------+---------+---------+---------+---------|
     BnaC.GLN1.4.b  CACACTCAAGGTTATGTATACACCCTGTAACCTTATCTCTTCATATACTCTGTTTTAATGACTCTTTTCTTGGTTATGTGAAAACAGAGGATCACAGAATTGGCTGGAGTTGTTTTATCTCTTGACCCTA
mRNA.BnaC.GLN1.4.b  ---------------------------------------------------------------------------------------AGGATCACAGAATTGGCTGGAGTTGTTTTATCTCTTGACCCTA
     BnaGln1.4_C3  ---------------------------------------------------------------------------------------AGGATCACAGAATTGGCTGGAGTTGTTTTATCTCTTGACCCTA
```

```
        Consensus  .......................................................................................AGGATCACAGAATTGGCTGGAGTTGTTTTATCTCTTGACCCTA

                   1951 1960      1970      1980      1990      2000      2010      2020      2030      2040      2050      2060      2070      2080
                   |--------+---------+---------+---------+---------+---------+---------+---------+---------+---------+---------+---------+---------|
     BnaC.GLN1.4.b AACCAATTCCGGTTTGTTTTTACCATAACTTTAAATATATAACCAAATTTTGTTCTTTTCAAATTCACTAAATTACTCTGTTTGATTTTTCTATTTTAGGGAGATTGGAATGGTGCAGGAGCGCACACAA
mRNA.BnaC.GLN1.4.b AACCAATTCCGG----------------------------------------------------------------------------------------GAGATTGGAATGGTGCAGGAGCGCACACAA
      BnaGln1.4_C3 AACCAATTCCGG----------------------------------------------------------------------------------------GAGATTGGAATGGTGCAGGAGCGCACACAA
         Consensus AACCAATTCCGG........................................................................................GAGATTGGAATGGTGCAGGAGCGCACACAA

                   2081 2090      2100      2110      2120      2130      2140      2150      2160      2170      2180      2190      2200      2210
                   |--------+---------+---------+---------+---------+---------+---------+---------+---------+---------+---------+---------+---------|
     BnaC.GLN1.4.b ATTACAGGTTCTTTGTGTTTTCAAGATCTGCTCTGATTCATTTTATATGGTTTTTGTTTGTTTTTATGAATTTGGTTTCTTGTATTCTGCAGTACAAAGTCGATGAGAGAAGATGGAGGGTACGAGGTGA
mRNA.BnaC.GLN1.4.b ATTACAG-------------------------------------------------------------------------------------TACAAAGTCGATGAGAGAAGATGGAGGGTACGAGGTGA
      BnaGln1.4_C3 ATTACAG-------------------------------------------------------------------------------------TACAAAGTCGATGAGAGAAGATGGAGGGTACGAGGTGA
         Consensus ATTACAG.....................................................................................TACAAAGTCGATGAGAGAAGATGGAGGGTACGAGGTGA

                   2211 2220      2230      2240      2250      2260      2270      2280      2290      2300      2310      2320      2330      2340
                   |--------+---------+---------+---------+---------+---------+---------+---------+---------+---------+---------+---------+---------|
     BnaC.GLN1.4.b TAAAGAAAGCGATAGAGAAGCTTGGATTGCGTCACAAGGAACACATCTCTGCTTATGGTGAAGGCAACGAGCGTCGCCTCACTGGCAAACACGAGACTGCCGATATCAACACTTTCTTATGGGTAATAGT
mRNA.BnaC.GLN1.4.b TAAAGAAAGCGATAGAGAAGCTTGGATTGCGTCACAAGGAACACATCTCTGCTTATGGTGAAGGCAACGAGCGTCGCCTCACTGGCAAACACGAGACTGCCGATATCAACACTTTCTTATGGG-------
      BnaGln1.4_C3 TAAAGAAAGCGATAGAGAAGCTTGGATTGCGTCACAAGGAACACATCTCTGCTTATGGTGAAGGCAACGAGCGTCGCCTCACTGGCAAACACGAGACTGCCGATATCAACACTTTCTTATGGG-------
         Consensus TAAAGAAAGCGATAGAGAAGCTTGGATTGCGTCACAAGGAACACATCTCTGCTTATGGTGAAGGCAACGAGCGTCGCCTCACTGGCAAACACGAGACTGCCGATATCAACACTTTCTTATGGG.......

                   2341 2350      2360      2370      2380      2390      2400      2410      2420      2430      2440      2450      2460      2470
                   |--------+---------+---------+---------+---------+---------+---------+---------+---------+---------+---------+---------+---------|
     BnaC.GLN1.4.b TTTATTTCATAGCATGTCAAAGTGTGCATTCATATCACATGAGCCTAAGTCATGCATGATCTAACTTAACTGGTTTGTACAGGGTGTGGCCAACCGTGGGGCATCGATTAGAGTTGGTCGGGACACTGAG
mRNA.BnaC.GLN1.4.b -----------------------------------------------------------------------------------GTGTGGCCAACCGTGGGGCATCGATTAGAGTTGGTCGGGACACTGAG
      BnaGln1.4_C3 -----------------------------------------------------------------------------------GTGTGGCCAACCGTGGGGCATCGATTAGAGTTGGTCGGGACACTGAG
         Consensus ...................................................................................GTGTGGCCAACCGTGGGGCATCGATTAGAGTTGGTCGGGACACTGAG

                   2471 2480      2490      2500      2510      2520      2530      2540      2550      2560      2570      2580      2590      2600
                   |--------+---------+---------+---------+---------+---------+---------+---------+---------+---------+---------+---------+---------|
     BnaC.GLN1.4.b CAAGCTGGGAAAGGGTACTTTGAAGATCGTAGGCCAGCGTCCAACATGGATCCGTACACTGTGACCTCCATGATTGCTGAAACTACAATCCTCTGGAAGCCATGAATGAAGAAAACTCGAGCTTCAAGGA
mRNA.BnaC.GLN1.4.b CAAGCTGGGAAAGGGTACTTTGAAGATCGTAGGCCAGCGTCCAACATGGATCCGTACACTGTGACCTCCATGATTGCTGAAACTACAATCCTCTGGAAGCCATGAATGAAGAAAACTCGAGCTTCAAGGA
      BnaGln1.4_C3 CAAGCTGGGAAAGGGTACTTTGAAGATCGTAGGCCAGCGTCCAACATGGATCCGTACACTGTGACCTCCATGATTGCTGAAACTACAATCCTCTGGAAGCCATGAATGAAGAA
         Consensus CAAGCTGGGAAAGGGTACTTTGAAGATCGTAGGCCAGCGTCCAACATGGATCCGTACACTGTGACCTCCATGATTGCTGAAACTACAATCCTCTGGAAGCCATGAATGAAGAAaactcgagcttcaagga

                   2601 2610      2620      2630      2640      2650      2660      2670      2680      2690      2700      2710      2720      2730
                   |--------+---------+---------+---------+---------+---------+---------+---------+---------+---------+---------+---------+---------|
     BnaC.GLN1.4.b ACCTCTAATATCAGTTCATGTGTCATTCTTATATTGTCTCGTTCTGTCTTCTTTGTTTAAGTGTGTTTGATTTAAACTCTTAGAACTTGTTCTATTTCTATATTATGTTATTACAAATTCAAATATCAGT
mRNA.BnaC.GLN1.4.b ACCTCTAATATCAGTTCATGTGTCATTCTTATATTGTCTCGTTCTGTCTTCTTTGTTTAAGTGTGTTTGATTTAAACTCTTAGAACTTGTTCTATTTCTATATTATGTTATTACAAATTCAAATATCAGT
      BnaGln1.4_C3
         Consensus acctctaatatcagttcatgtgtcattcttatattgtctcgttctgtcttctttgtttaagtgtgtttgatttaaactcttagaacttgttctatttctatattatgttattacaaattcaaatatcagt

                   2731 2740      2750      2760      2770      2780      2790      2800      2810      2820      2830      2840      2850      2860
                   |--------+---------+---------+---------+---------+---------+---------+---------+---------+---------+---------+---------+---------|
     BnaC.GLN1.4.b TTTAGAATATGAAGAGGATCTGAACTTTAAACTTTTTATAGAAACCAAAATCGCAAGTTGCAAGCTCCTCCGTTACTAGCCAACCCAAACTAGACCGGCCGAATCGACATTTAGCACACTCTATTTGGCC
mRNA.BnaC.GLN1.4.b TTTAGAATATGAAGAGGATCTGAACTTTAAACTTTTTATAGAAACCAAAATCGCAAGTTGCAAGCTCCTCCGTTACTAGCCAACCCAAACTAGACCGGCCGAATCGACATTTAGCACACTCTATTTGGCC
      BnaGln1.4_C3
         Consensus tttagaatatgaagaggatctgaactttaaactttttatagaaaccaaaatcgcaagttgcaagctcctccgttactagccaacccaaactagaccggccgaatcgacatttagcacactctatttggcc

                   2861 2870      2880      2890      2900      2910      292022
                   |--------+---------+---------+---------+---------+--|
     BnaC.GLN1.4.b CATTTTAGCTTAATGGACTCAAATTTATAACTTTTAAATATTGAAACTCTGTGCAAAAAAAA
mRNA.BnaC.GLN1.4.b CATTTTAGCTTAATGGACTCAAATTTATAACTTTTAAATATTGAAACTCTGTGCAAAAAAAA
      BnaGln1.4_C3
         Consensus cattttagcttaatggactcaaatttataacttttaaatattgaaactctgtgcaaaaaaaa
```

file:///C:/Users/Camille/Desktop/Supp%20Figure%20alignements/C4b.gif[25/09/2013 13:30:10]

```
                    1       10        20        30        40        50        60        70        80        90        100       110       120       130
BnaA.GLN1.5.a                                 TATATAAAAAGTGAGTGAGCGAAGCAGAGTGAGCCAGTGCTCACCTCAGACTGATTATTATAACTCGATCGTCATCTTCTTCGGCTTGATGGAAACAGAAAAAAT
mRNA.BnaA.GLN1.5.a                             ATATAAAAAGTGAGTGAGCGAAGCAGAGTGAGCCAGTGCTCACCTCAGACTGATTATTATAACTCGATCGTCATCTTCTTCGGCTTGATGGAAACAGAAAAAAT
BnaGln1.5_C2        GGATCTCCGGATAGTTCGCCGCTTCTTATATAAAAAGTGAGTGAGCGAAGCAGAGTGAGCCAGTGCTCACCTCAGACTGATTATTATAACTCGATCGTCATCTTCTTCGGCTTGATGGAAACAGAAAAAAT
Consensus           ..........................tATATAAAAAGTGAGTGAGCGAAGCAGAGTGAGCCAGTGCTCACCTCAGACTGATTATTATAACTCGATCGTCATCTTCTTCGGCTTGATGGAAACAGAAAAAAT

                    131     140       150       160       170       180       190       200       210       220       230       240       250       260
BnaA.GLN1.5.a       GTCTCCACTCTCCGATCTCCTAAACCTCAATCTCGACACCAAGCAAATCATCGCTGAATACATATGGTTCGATCATTTTCCCTGATCATGTCACTCTTTTGTGTTTGCTTAATCTCTTGATTGCTATGTC
mRNA.BnaA.GLN1.5.a  GTCTCCACTCTCCGATCTCCTAAACCTCAATCTCGACACCAAGCAAATCATCGCTGAATACATATGG---------------------------------------------------------------
BnaGln1.5_C2        GTCTCCACTCTCCGATCTCCTAAACCTCAATCTCGACACCAAGCAAATCATCGCTGAATACATATGG---------------------------------------------------------------
Consensus           GTCTCCACTCTCCGATCTCCTAAACCTCAATCTCGACACCAAGCAAATCATCGCTGAATACATATGG...............................................................

                    261     270       280       290       300       310       320       330       340       350       360       370       380       390
BnaA.GLN1.5.a       CCGTTTTGGACATGAACACTGCTGCATTTCATCTAGATTTCTCGATCTTTTTTTTTTGTTCTGTAGATTTCTCGATCTTACTGCATGCGTATTTTTTTTTTTATCAAAGCATGCGCATGTTCTTACATGG
mRNA.BnaA.GLN1.5.a  ----------------------------------------------------------------------------------------------------------------------------------
BnaGln1.5_C2        ----------------------------------------------------------------------------------------------------------------------------------
Consensus           ..................................................................................................................................

                    391     400       410       420       430       440       450       460       470       480       490       500       510       520
BnaA.GLN1.5.a       CTCTGTTAACAACATAAAGTTTTTATTCTTGTCTTCGTGAGAACAGGATCGGTGGGTCTGGAATGGACATTAGAAGCAAAGGCAGGGTAAGAATAATAATATTTACACTATTTTCTCTTTTATTTTAACT
mRNA.BnaA.GLN1.5.a  -----------------------------------------------ATCGGTGGGTCTGGAATGGACATTAGAAGCAAAGGCAGG--------------------------------------------
BnaGln1.5_C2        -----------------------------------------------ATCGGTGGGTCTGGAATGGACATTAGAAGCAAAGGCAGG--------------------------------------------
Consensus           ...............................................ATCGGTGGGTCTGGAATGGACATTAGAAGCAAAGGCAGG............................................

                    521     530       540       550       560       570       580       590       600       610       620       630       640       650
BnaA.GLN1.5.a       CTATAATCAAAAAACTTTTTTTTTAATACAGACATTACCAGGACCAGTAAGTGATCCATCAAAGCTTCCGAAATGGAACTACGATGGATCCAGCACCAATCAAGCCGCCGGAGATGACAGTGAAGTCATT
mRNA.BnaA.GLN1.5.a  -------------------------------ACATTACCAGGACCAGTAAGTGATCCATCAAAGCTTCCGAAATGGAACTACGATGGATCCAGCACCAATCAAGCCGCCGGAGATGACAGTGAAGTCATT
BnaGln1.5_C2        -------------------------------ACATTACCAGGACCAGTAAGTGATCCATCAAAGCTTCCGAAATGGAACTACGATGGATCCAGCACCAATCAAGCCGCCGGAGATGACAGTGAAGTCATT
Consensus           ...............................ACATTACCAGGACCAGTAAGTGATCCATCAAAGCTTCCGAAATGGAACTACGATGGATCCAGCACCAATCAAGCCGCCGGAGATGACAGTGAAGTCATT

                    651     660       670       680       690       700       710       720       730       740       750       760       770       780
BnaA.GLN1.5.a       CTATAGTAATCTTCTTCACATAAATTATTTTCAAGTATAATGAATTAAAACTTATTATTATTAACTATTATGTTTGTTTAATGTTATTTTTGAAATGAATCTAAAAAAGTCCTCAGGCGATTTTTAAAGA
mRNA.BnaA.GLN1.5.a  CTATA--------------------------------------------------------------------------------------------------------TCCTCAGGCGATTTTTAAAGA
BnaGln1.5_C2        CTATA--------------------------------------------------------------------------------------------------------TCCTCAGGCGATTTTTAAAGA
Consensus           CTATA........................................................................................................TCCTCAGGCGATTTTTAAAGA

                    781     790       800       810       820       830       840       850       860       870       880       890       900       910
BnaA.GLN1.5.a       CCCATTCAGGAAAGGGAATAACATTCTCGTAAGCTTATCTGATTTTCTTTAGCAAAAGATTCATCTGATTATTTTTTTTGTTGGAGGATTTTATGAATTCAAAATTTCTTTAATAATTATAATTAAATCA
mRNA.BnaA.GLN1.5.a  CCCATTCAGGAAAGGGAATAACATTCTCGT----------------------------------------------------------------------------------------------------
BnaGln1.5_C2        CCCATTCAGGAAAGGGAATAACATTCTCGT----------------------------------------------------------------------------------------------------
Consensus           CCCATTCAGGAAAGGGAATAACATTCTCGT....................................................................................................

                    911     920       930       940       950       960       970       980       990       1000      1010      1020      1030      1040
BnaA.GLN1.5.a       GGTGATGTGTGATGCTTACACACCGAAAGGAGATCCAATCCCGACCAACAATAGGCACAAAGCCGTGAAAATCTTCGATCATCCCAATGTGAAGGCTGAAGAGCCTTGGTAAGATCATTCCATATTTCAC
mRNA.BnaA.GLN1.5.a  ---GATGTGTGATGCTTACACACCGAAAGGAGATCCAATCCCGACCAACAATAGGCACAAAGCCGTGAAAATCTTCGATCATCCCAATGTGAAGGCTGAAGAGCCTTGGT--------------------
BnaGln1.5_C2        ---GATGTGTGATGCTTACACACCGAAAGGAGATCCAATCCCGACCAACAATAGGCACAAAGCCGTGAAAATCTTCGATCATCCCAATGTGAAGGCTGAAGAGCCTTGGT--------------------
Consensus           ...GATGTGTGATGCTTACACACCGAAAGGAGATCCAATCCCGACCAACAATAGGCACAAAGCCGTGAAAATCTTCGATCATCCCAATGTGAAGGCTGAAGAGCCTTGGT....................

                    1041    1050      1060      1070      1080      1090      1100      1110      1120      1130      1140      1150      1160      1170
BnaA.GLN1.5.a       TTTGAGTCTTGGTTTGGAGCCGGTTTGGTTTAGGACAAATATATATTTTAGTATCTGTTAATTGTCTACAAAAATTATTTTCTTGAAAATTGTGGTTGATTCAGTTCGGTTTGGTATGACTAATATCGGG
mRNA.BnaA.GLN1.5.a  ----------------------------------------------------------------------------------------------------------------------------------
BnaGln1.5_C2        ----------------------------------------------------------------------------------------------------------------------------------
Consensus           ..................................................................................................................................

                    1171    1180      1190      1200      1210      1220      1230      1240      1250      1260      1270      1280      1290      1300
BnaA.GLN1.5.a       TTAAGTTTAACTTTCGGTTCGCTGAAAACATTTGATAACCGATCGGTTGATGAATATATATAATTCTTAGTTTGGTTAATTTCATAATTATAATTCGGGTTGATATTTGGATGTGTTATTTATTAGGTTT
mRNA.BnaA.GLN1.5.a  --------------------------------------------------------------------------------------------------------------------------------TT
BnaGln1.5_C2        --------------------------------------------------------------------------------------------------------------------------------TT
Consensus           ................................................................................................................................TT

                    1301    1310      1320      1330      1340      1350      1360      1370      1380      1390      1400      1410      1420      1430
BnaA.GLN1.5.a       GGGATAGAGCAAGAATACACATTACTTAAGAAAGACGTCAAGTGGCCATTGGGTTGGCCCCTTGGTGGCTTTCCTGGTCCTCAGGTACACTAATGCTTAACATTTCTCCTTATAAATTCATAGACTAAAC
mRNA.BnaA.GLN1.5.a  GGGATAGAGCAAGAATACACATTACTTAAGAAAGACGTCAAGTGGCCATTGGGTTGGCCCCTTGGTGGCTTTCCTGGTCCTCAGG---------------------------------------------
BnaGln1.5_C2        GGGATAGAGCAAGAATACACATTACTTAAGAAAGACGTCAAGTGGCCATTGGGTTGGCCCCTTGGTGGCTTTCCTGGTCCTCAGG---------------------------------------------
Consensus           GGGATAGAGCAAGAATACACATTACTTAAGAAAGACGTCAAGTGGCCATTGGGTTGGCCCCTTGGTGGCTTTCCTGGTCCTCAGG.............................................

                    1431    1440      1450      1460      1470      1480      1490      1500      1510      1520      1530      1540      1550      1560
BnaA.GLN1.5.a       TCTTAATATGAAGAACATGTTTCTGATATCAATATCTCTCCCTTTTGTGTGTGTGTGTGTCTGTGTGTGTGTTTGATGAACAGGGACCGTACTATTGTGCGGTGGGTGCAGACAAAGCCTTTGGGCGT
mRNA.BnaA.GLN1.5.a  --------------------------------------------------------------------------------------GACCGTACTATTGTGCGGTGGGTGCAGACAAAGCCTTTGGGCGT
BnaGln1.5_C2        --------------------------------------------------------------------------------------GACCGTACTATTGTGCGGTGGGTGCAGACAAAGCCTTTGGGCGT
Consensus           ......................................................................................GACCGTACTATTGTGCGGTGGGTGCAGACAAAGCCTTTGGGCGT

                    1561    1570      1580      1590      1600      1610      1620      1630      1640      1650      1660      1670      1680      1690
BnaA.GLN1.5.a       GACATTGTGGATGGTCACTACAAAGCTTGTCTTTACGCTGGTTTAAGCATAGGTGGTGCCAATGGTGAAGTCATGCCTGGTCAATGGGAGTTTCAAATCAGCCCTACTGTTGGTATTGGTGCAGGTGATC
mRNA.BnaA.GLN1.5.a  GACATTGTGGATGGTCACTACAAAGCTTGTCTTTACGCTGGTTTAAGCATAGGTGGTGCCAATGGTGAAGTCATGCCTGGTCAATGGGAGTTTCAAATCAGCCCTACTGTTGGTATTGGTGCAGGTGATC
BnaGln1.5_C2        GACATTGTGGATGGTCACTACAAAGCTTGTCTTTACGCTGGTTTAAGCATAGGTGGTGCCAATGGTGAAGTCATGCCTGGTCAATGGGAGTTTCAAATCAGCCCTACTGTTGGTATTGGTGCAGGTGATC
Consensus           GACATTGTGGATGGTCACTACAAAGCTTGTCTTTACGCTGGTTTAAGCATAGGTGGTGCCAATGGTGAAGTCATGCCTGGTCAATGGGAGTTTCAAATCAGCCCTACTGTTGGTATTGGTGCAGGTGATC

                    1691    1700      1710      1720      1730      1740      1750      1760      1770      1780      1790      1800      1810      1820
BnaA.GLN1.5.a       AGTTATGGGTTGCTCGCTACATACTTGAGGTGATTAAAAAACGATATACATAAAGCATTTTTATTTCTTTGTACGAAGTAGCATTTTTTGTATCTCTTATGAATCATTTTGTGTTTATTTTGGCAGAGGA
mRNA.BnaA.GLN1.5.a  AGTTATGGGTTGCTCGCTACATACTTGAG-------------------------------------------------------------------------------------------------AGGA
BnaGln1.5_C2        AGTTATGGGTTGCTCGCTACATACTTGAG-------------------------------------------------------------------------------------------------AGGA
Consensus           AGTTATGGGTTGCTCGCTACATACTTGAG.................................................................................................AGGA

                    1821    1830      1840      1850      1860      1870      1880      1890      1900      1910      1920      1930      1940      1950
BnaA.GLN1.5.a       TTACTGAGATATGCGGCGTAATTGTCTCATTTGATCCCAAACCAATCGAGGTACGGATCATCAAGACTTTTTTTTTTGTAGTTATCATGGTTTTTTTGTTTTTGAGTTGATTTTGTTATCTATGGCGGT
mRNA.BnaA.GLN1.5.a  TTACTGAGATATGCGGCGTAATTGTCTCATTTGATCCCAAACCAATCGAGG-------------------------------------------------------------------------------
BnaGln1.5_C2        TTACTGAGATATGCGGCGTAATTGTCTCATTTGATCCCAAACCAATCGAGG-------------------------------------------------------------------------------
```

```
     Consensus  TTACTGAGATATGCGGCGTAATTGTCTCATTTGATCCCAAACCAATCGAGG...............................................................................

                1951 1960      1970      1980      1990      2000      2010      2020      2030      2040      2050      2060      2070      2080
                |--------+---------+---------+---------+---------+---------+---------+---------+---------+---------+---------+---------+---------|
 BnaA.GLN1.5.a  TTGATAATGATTAGGGTGATTGGAACGGAGCAGCTGCTCATACAAACTTCAGTACAAAATCAATGAGGAAAGAAGGAGGATTGGACTTGATCAAGAAAGCAATAGGGAAGCTTGAAGTGAAGCATAAACA
mRNA.BnaA.GLN1.5.a  ---------------GTGATTGGAACGGAGCAGCTGCTCATACAAACTTCAGTACAAAATCAATGAGGAAAGAAGGAGGATTGGACTTGATCAAGAAAGCAATAGGGAAGCTTGAAGTGAAGCATAAACA
   BnaGln1.5_C2  ---------------GTGATTGGAACGGAGCAGCTGCTCATACAAACTTCAGTACAAAATCAATGAGGAAAGAAGGAGGATTGGACTTGATCAAGAAAGCAATAGGGAAGCTTGAAGTGAAGCATAAACA
     Consensus  ...............GTGATTGGAACGGAGCAGCTGCTCATACAAACTTCAGTACAAAATCAATGAGGAAAGAAGGAGGATTGGACTTGATCAAGAAAGCAATAGGGAAGCTTGAAGTGAAGCATAAACA

                2081 2090      2100      2110      2120      2130      2140      2150      2160      2170      2180      2190      2200      2210
                |--------+---------+---------+---------+---------+---------+---------+---------+---------+---------+---------+---------+---------|
 BnaA.GLN1.5.a  ACACATTGCTGCTTATGGTGAAGGCAATGAGAGGCGTCTCACTGGGAAGCATGAAACCGCAGACATCAACAAGTTCTCTTGGGTATGAGATATTGAATAAAATCAAATATATAGTTTTGGATTTGTGATG
mRNA.BnaA.GLN1.5.a  ACACATTGCTGCTTATGGTGAAGGCAATGAGAGGCGTCTCACTGGGAAGCATGAAACCGCAGACATCAACAAGTTCTCTTGGG-----------------------------------------------
   BnaGln1.5_C2  ACACATTGCTGCTTATGGTGAAGGCAATGAGAGGCGTCTCACTGGGAAGCATGAAACCGCAGACATCAACAAGTTCTCTTGGG-----------------------------------------------
     Consensus  ACACATTGCTGCTTATGGTGAAGGCAATGAGAGGCGTCTCACTGGGAAGCATGAAACCGCAGACATCAACAAGTTCTCTTGGG...............................................

                2211 2220      2230      2240      2250      2260      2270      2280      2290      2300      2310      2320      2330      2340
                |--------+---------+---------+---------+---------+---------+---------+---------+---------+---------+---------+---------+---------|
 BnaA.GLN1.5.a  CTTTATACAAGCTTTCTTGATGTGTGTAATTTGGTTTTGTTGGGAGTAGGGAGTTGCGGATCGTGGAGCATCGGTGAGAGTGGGAAGAGATACGGAGAAAGAAGGGAAAGGTTATTTTGAAGATCGAAGA
mRNA.BnaA.GLN1.5.a  --------------------------------------------------GAGTTGCGGATCGTGGAGCATCGGTGAGAGTGGGAAGAGATACGGAGAAAGAAGGGAAAGGTTATTTTGAAGATCGAAGA
   BnaGln1.5_C2  --------------------------------------------------GAGTTGCGGATCGTGGAGCATCGGTGAGAGTGGGAAGAGATACGGAGAAAGAAGGGAAAGGTTATTTTGAAGATCGAAGA
     Consensus  ..................................................GAGTTGCGGATCGTGGAGCATCGGTGAGAGTGGGAAGAGATACGGAGAAAGAAGGGAAAGGTTATTTTGAAGATCGAAGA

                2341 2350      2360      2370      2380      2390      2400      2410      2420      2430      2440      2450      2460      2470
                |--------+---------+---------+---------+---------+---------+---------+---------+---------+---------+---------+---------+---------|
 BnaA.GLN1.5.a  CCTTCGTCTAATATGGATCCTTATCTTGTTACCTCCATGATAGCTGAAACCACCATCCTCGGCTAAGCTTTCTTTTGAAGTTGTTGCATACGTTCTTTTGTTTCTTCATGTTTCGGTTTAATTTCGGTTT
mRNA.BnaA.GLN1.5.a  CCTTCGTCTAATATGGATCCTTATCTTGTTACCTCCATGATAGCTGAAACCACCATCCTCGGCTAAGCTTTCTTTTGAAGTTGTTGCATACGTTCTTTTGTTTCTTCATGTTTCGGTTTAATTTCGGTTT
   BnaGln1.5_C2  CCTTCGTCTAATATGGATCCTTATCTTGTTACCTCCATGATAGCTGAAACCACCATCCTCGGCTAAGCTTTCTTTTGAAGTTGTTGCATACGTTCTTTTGTTTCTTCATGTTTCGGTTTAATTTCGGTTT
     Consensus  CCTTCGTCTAATATGGATCCTTATCTTGTTACCTCCATGATAGCTGAAACCACCATCCTCGGCTAAGCTTTCTTTTGAAGTTGTTGCATACGTTCTTTTGTTTCTTCATGTTTCGGTTTAATTTCGGTTT

                2471 2480      2490      2500      2510      2520      2530      2540      2550      2560      2570      2580      2590      2600
                |--------+---------+---------+---------+---------+---------+---------+---------+---------+---------+---------+---------+---------|
 BnaA.GLN1.5.a  GAGACTTTTTTTTTTATTGCTAATAATTCATGGGATGGTCTTGATCCTATTGTTTGTTTATCCTGGTTCAGTTGTTAGTGTTAAACAAAATTGAATTGGGAAAA
mRNA.BnaA.GLN1.5.a  GAGACTTTTTTTTTTATTGCTAATAATTCATGGGATGGTCTTGATCCTATTGTTTGTTTATCCTGGTTCAGTTGTTAGTGTTAAACAAAATTGAATTGGGAAAA
   BnaGln1.5_C2  GAGACCTTTTTTTTTAGTGCTAATAATTCATGGGATGGTCTTGATCCTATTGTTTGTTTATCCTGGTTCAGTTGTTAGTGTTAAACAAAATTGAATTGGGAAAATAAAGGTTCTTAGTTCTTACTTTTTA
     Consensus  GAGACtTTTTTTTTTAtTGCTAATAATTCATGGGATGGTCTTGATCCTATTGTTTGTTTATCCTGGTTCAGTTGTTAGTGTTAAACAAAATTGAATTGGGAAAA..........................

                2601   2611
                |--------+|
 BnaA.GLN1.5.a
mRNA.BnaA.GLN1.5.a
   BnaGln1.5_C2  AAAAAAAAAAA
     Consensus  ...........
```

file:///C:/Users/Camille/Desktop/Supp%20Figure%20alignements/A5a.gif[25/09/2013 13:28:44]

```
                    1        10        20        30        40        50        60        70        80        90        100       110       120       130
BnaC.GLN1.5.a       TATATAAAAAGTGAGTTAGCGAAGCAGAGTGAGCCAATGCTCACCTCAGACTGATTATTATAACTCGATCGTCTTCTTCTTCGGCTTGATGGAAACAGAAAGAATGTCTCCACTCTCAGATCTCCTGAAC
mRNA.BnaC.GLN1.5.a  TATATAAAAAGTGAGTTAGCGAAGCAGAGTGAGCCAATGCTCACCTCAGACTGATTATTATAACTCGATCGTCTTCTTCTTCGGCTTGATGGAAACAGAAAGAATGTCTCCACTCTCAGATCTCCTGAAC
BnaGln1.5_C1        GATATAAAAAGTGAGTTAGCGAAGCAGAGTGAGCCAATGCTCACCTCAGACTGATTATTATAACTCGATCGTCTTCTTCTTCGGCTTGATGGAAACAGAAAGAATGTCTCCACTCTCAGATCTCCTGAAC
Consensus           tATATAAAAAGTGAGTTAGCGAAGCAGAGTGAGCCAATGCTCACCTCAGACTGATTATTATAACTCGATCGTCTTCTTCTTCGGCTTGATGGAAACAGAAAGAATGTCTCCACTCTCAGATCTCCTGAAC

                    131      140       150       160       170       180       190       200       210       220       230       240       250       260
BnaC.GLN1.5.a       CTCAACCTCGACACCAAGCAAATCATCGCTGAATACATATGGTTCGATCATTTTCCCTGATCATGTCACTCTTTTGTGTTTGCTTAATCGCTTGATTGCTATGTCCCGTTTTGGACATGAACACTGCTGC
mRNA.BnaC.GLN1.5.a  CTCAACCTCGACACCAAGCAAATCATCGCTGAATACATATGG----------------------------------------------------------------------------------------
BnaGln1.5_C1        CTCAACCTCGACACCAAGCAAATCATCGCTGAATACATATGG----------------------------------------------------------------------------------------
Consensus           CTCAACCTCGACACCAAGCAAATCATCGCTGAATACATATGG........................................................................................

                    261      270       280       290       300       310       320       330       340       350       360       370       380       390
BnaC.GLN1.5.a       ATCTCATCTAGATTTCTCGAACTTTTTTTTTTCTGTAGATTTCTCGATCTTACTGCATGCGTATGTTTTTTTCTTTTTTTATCGAAGCATGCGCATGTTCTTACATGGCTCTGTTAACAACATTAAGTTT
mRNA.BnaC.GLN1.5.a  ----------------------------------------------------------------------------------------------------------------------------------
BnaGln1.5_C1        ----------------------------------------------------------------------------------------------------------------------------------
Consensus           ..................................................................................................................................

                    391      400       410       420       430       440       450       460       470       480       490       500       510       520
BnaC.GLN1.5.a       TTAATCTTGTCTTCGTGAGAACAGGATCGGTGGGTCTGGAATGGACATTAGAAGCAAAGGCAGGGTACGAATAATAATATTTACACTATTTTCTCTTTTATTTTAACTCTAAAATCAAAAACTTTTTTTT
mRNA.BnaC.GLN1.5.a  -------------------------ATCGGTGGGTCTGGAATGGACATTAGAAGCAAAGGCAGG------------------------------------------------------------------
BnaGln1.5_C1        -------------------------ATCGGTGGGTCTGGAATGGACATTAGAAGCAAAGGCAGG------------------------------------------------------------------
Consensus           .........................ATCGGTGGGTCTGGAATGGACATTAGAAGCAAAGGCAGG..................................................................

                    521      530       540       550       560       570       580       590       600       610       620       630       640       650
BnaC.GLN1.5.a       TAATACAGACATTACCAGGACCTGTAAGTGATCCATCAAAGCTTCCGAAATGGAACTACGATGGATCCAGCACCAATCAAGCCGCCGGAGATGACAGTGAAGTCATTCTATAGTAATCTTCTTCACATAA
mRNA.BnaC.GLN1.5.a  --------ACATTACCAGGACCTGTAAGTGATCCATCAAAGCTTCCGAAATGGAACTACGATGGATCCAGCACCAATCAAGCCGCCGGAGATGACAGTGAAGTCATTCTATA------------------
BnaGln1.5_C1        --------ACATTACCAGGACCTGTAAGTGATCCATCAAAGCTTCCGAAATGGAACTACGATGGATCCAGCACCAATCAAGCCGCCGGAGATGACAGTGAAGTCATTCTATA------------------
Consensus           ........ACATTACCAGGACCTGTAAGTGATCCATCAAAGCTTCCGAAATGGAACTACGATGGATCCAGCACCAATCAAGCCGCCGGAGATGACAGTGAAGTCATTCTATA..................

                    651      660       670       680       690       700       710       720       730       740       750       760       770       780
BnaC.GLN1.5.a       ATTATTTTCAAGTATAATGAATTAAAACTTATTATTATTAACTATTATATTTGTTTAATGTTATCTTTGAAATGAATCTAAAAAAGTCCTCAGGCGATTTTTAAAGACCCGTTCAGGAAAGGGAATAACA
mRNA.BnaC.GLN1.5.a  --------------------------------------------------------------------------------------TCCTCAGGCGATTTTTAAAGACCCGTTCAGGAAAGGGAATAACA
BnaGln1.5_C1        --------------------------------------------------------------------------------------TCCTCAGGCGATTTTTAAAGACCCGTTCAGGAAAGGGAATAACA
Consensus           ......................................................................................TCCTCAGGCGATTTTTAAAGACCCGTTCAGGAAAGGGAATAACA

                    781      790       800       810       820       830       840       850       860       870       880       890       900       910
BnaC.GLN1.5.a       TTCTCGTAAGCTCATCTGATTTTCTTTAGCAAAAGATTCATCTGATTATTTTTTAGTTGGAGGATTTTATGAATTCAAAATTTCTTTAATAATTATAATTAAATCAGGTGATGTGTGATGCTTACACACC
mRNA.BnaC.GLN1.5.a  TTCTCGT------------------------------------------------------------------------------------------------------GATGTGTGATGCTTACACACC
BnaGln1.5_C1        TTCTCGT------------------------------------------------------------------------------------------------------GATGTGTGATGCTTACACACC
Consensus           TTCTCGT......................................................................................................GATGTGTGATGCTTACACACC

                    911      920       930       940       950       960       970       980       990       1000      1010      1020      1030      1040
BnaC.GLN1.5.a       GAAAGGAGATCCAATCCCGACCAACAATAGGCACAAAGCCGTGAAAATCTTCGATCATCCCAATGTGAAGGCTGAAGAGCCTTGGTAAGATCATTCCATATTTCACTTTGAGTCTTGGTTTGGAGCCGGT
mRNA.BnaC.GLN1.5.a  GAAAGGAGATCCAATCCCGACCAACAATAGGCACAAAGCCGTGAAAATCTTCGATCATCCCAATGTGAAGGCTGAAGAGCCTTGGT--------------------------------------------
BnaGln1.5_C1        GAAAGGAGATCCAATCCCGACCAACAATAGGCACAAAGCCGTGAAAATCTTCGATCATCCCAATGTGAAGGCTGAAGAGCCTTGGT--------------------------------------------
Consensus           GAAAGGAGATCCAATCCCGACCAACAATAGGCACAAAGCCGTGAAAATCTTCGATCATCCCAATGTGAAGGCTGAAGAGCCTTGGT............................................

                    1041     1050      1060      1070      1080      1090      1100      1110      1120      1130      1140      1150      1160      1170
BnaC.GLN1.5.a       TTAGTTTAGGACAAATATATATATTTTAGTATCTGTTAATTGTCTACAAAAATTGTTTTCTTGAAAATTGTGGTTGATTCAGTTCGGTTCGGTATGACTAATATCGGGTTAAGTTACTTTCGGTTCGGTGAA
mRNA.BnaC.GLN1.5.a  ----------------------------------------------------------------------------------------------------------------------------------
BnaGln1.5_C1        ----------------------------------------------------------------------------------------------------------------------------------
Consensus           ..................................................................................................................................

                    1171     1180      1190      1200      1210      1220      1230      1240      1250      1260      1270      1280      1290      1300
BnaC.GLN1.5.a       AACATTTGATAACCGATCGGTTGATGAATATATAATTCTTAGTTTGGTTAATTTCATAATTATAATTCGGTTTGATATTTGGATGTGTTATTTATTAGGTTTGGGATAGAGCAAGAATACACATTACTTA
mRNA.BnaC.GLN1.5.a  ----------------------------------------------------------------------------------------------------TTGGGATAGAGCAAGAATACACATTACTTA
BnaGln1.5_C1        ----------------------------------------------------------------------------------------------------TTGGGATAGAGCAAGAATACACATTACTTA
Consensus           ....................................................................................................TTGGGATAGAGCAAGAATACACATTACTTA

                    1301     1310      1320      1330      1340      1350      1360      1370      1380      1390      1400      1410      1420      1430
BnaC.GLN1.5.a       AGAAAGACGTCAAGTGGCCATTGGGTTGGCCCCTTGGTGGCTTTCCTGGTCCTCAGGTACACTAATGCTTAACATTTCTCCTTACAAATTCATAGACTAAACTCTTAATATGAAGAACATGTTTCTGATA
mRNA.BnaC.GLN1.5.a  AGAAAGACGTCAAGTGGCCATTGGGTTGGCCCCTTGGTGGCTTTCCTGGTCCTCAGG-------------------------------------------------------------------------
BnaGln1.5_C1        AGAAAGACGTCAAGTGGCCATTGGGTTGGCCCCTTGGTGGCTTTCCTGGTCCTCAGG-------------------------------------------------------------------------
Consensus           AGAAAGACGTCAAGTGGCCATTGGGTTGGCCCCTTGGTGGCTTTCCTGGTCCTCAGG.........................................................................

                    1431     1440      1450      1460      1470      1480      1490      1500      1510      1520      1530      1540      1550      1560
BnaC.GLN1.5.a       TCGAATATCTCTCCTCTTTTTTTGTGTGTGCGTGTTTGATGAAAACAGGGACCGTACTATTGTGCGGTCGGTGCAGACAAAGCCTTTGGTCGTGACATTGTGGATGGTCACTACAAAGCTTGTCTTTACG
mRNA.BnaC.GLN1.5.a  -------------------------------------------------GACCGTACTATTGTGCGGTCGGTGCAGACAAAGCCTTTGGTCGTGACATTGTGGATGGTCACTACAAAGCTTGTCTTTACG
BnaGln1.5_C1        -------------------------------------------------GACCGTACTATTGTGCGGTCGGTGCAGACAAAGCCTTTGGTCGTGACATTGTGGATGGTCACTACAAAGCTTGTCTTTACG
Consensus           .................................................GACCGTACTATTGTGCGGTCGGTGCAGACAAAGCCTTTGGTCGTGACATTGTGGATGGTCACTACAAAGCTTGTCTTTACG

                    1561     1570      1580      1590      1600      1610      1620      1630      1640      1650      1660      1670      1680      1690
BnaC.GLN1.5.a       CTGGTTTAAGCATAGGTGGTGCCAATGGTGAAGTCATGCCTGGTCAATGGGAGTTTCAAATCAGCCCTACTGTTGGTATTGGTGCAGGTGATCAGTTATGGGTTGCTCGCTACATACTCGAGGTGAATAA
mRNA.BnaC.GLN1.5.a  CTGGTTTAAGCATAGGTGGTGCCAATGGTGAAGTCATGCCTGGTCAATGGGAGTTTCAAATCAGCCCTACTGTTGGTATTGGTGCAGGTGATCAGTTATGGGTTGCTCGCTACATACTCGAG--------
BnaGln1.5_C1        CTGGTTTAAGCATAGGTGGTGCCAATGGTGAAGTCATGCCTGGTCAATGGGAGTTTCAAATCAGCCCTACTGTTGGTATTGGTGCAGGTGATCAGTTATGGGTTGCTCGCTACATACTCGAG--------
Consensus           CTGGTTTAAGCATAGGTGGTGCCAATGGTGAAGTCATGCCTGGTCAATGGGAGTTTCAAATCAGCCCTACTGTTGGTATTGGTGCAGGTGATCAGTTATGGGTTGCTCGCTACATACTCGAG........

                    1691     1700      1710      1720      1730      1740      1750      1760      1770      1780      1790      1800      1810      1820
BnaC.GLN1.5.a       AAAAACGATATACATAAAGCATTTTTGTTTCTTTGTACTAAGTAGCATTTTTTTTTTGTATTTCTCTTATGAATCATTTAGTGTTTATTTTGGCAGAGGATTACTGAGATATGCGGCGTGATTGTCTCAT
mRNA.BnaC.GLN1.5.a  ------------------------------------------------------------------------------------------------AGGATTACTGAGATATGCGGCGTGATTGTCTCAT
BnaGln1.5_C1        ------------------------------------------------------------------------------------------------AGGATTACTGAGATATGCGGCGTGATTGTCTCAT
Consensus           ................................................................................................AGGATTACTGAGATATGCGGCGTGATTGTCTCAT

                    1821     1830      1840      1850      1860      1870      1880      1890      1900      1910      1920      1930      1940      1950
BnaC.GLN1.5.a       TTGATCCCAAACCAATCGAGGTATGGATCATCAAGACTCTTTTTTTTTGTAGTTATCATGGATTTTTTTTGTTTGAGTTGGGTTTGTTATCTATGGCGGTTTGATAATGATTAGGGTGATTGGAACGGAG
mRNA.BnaC.GLN1.5.a  TTGATCCCAAACCAATCGAGG----------------------------------------------------------------------------------------------GTGATTGGAACGGAG
BnaGln1.5_C1        TTGATCCCAAACCAATCGAGG----------------------------------------------------------------------------------------------GTGATTGGAACGGAG
```

file:///C:/Users/Camille/Desktop/Supp%20Figure%20alignements/C5a.gif[25/09/2013 13:30:22]

```
        Consensus  TTGATCCCAAACCAATCGAGG..............................................................................................GTGATTGGAACGGAG

                   1951  1960      1970      1980      1990      2000      2010      2020      2030      2040      2050      2060      2070      2080
                   |--------+---------+---------+---------+---------+---------+---------+---------+---------+---------+---------+---------+---------|
     BnaC.GLN1.5.a CAGCTGCTCATACAAACTTCAGTACAAAATCAATGAGGAAAGAAGGAGGATTGGACTTGATAAAAAAAGCAATAGGGAAGCTTGAAGTGAAGCATAAACAACACATTGCTGCTTATGGTGAAGGCAATGA
mRNA.BnaC.GLN1.5.a CAGCTGCTCATACAAACTTCAGTACAAAATCAATGAGGAAAGAAGGAGGATTGGACTTGATAAAAAAAGCAATAGGGAAGCTTGAAGTGAAGCATAAACAACACATTGCTGCTTATGGTGAAGGCAATGA
      BnaGln1.5_C1 CAGCTGCTCATACAAACTTCAGTACAAAATCAATGAGGAAAGAAGGAGGATTGGACTTGATAAAAAAAGCAATAGGGAAGCTTGAAGTGAAGCATAAACAACACATTGCTGCTTATGGTGAAGGCAATGA
         Consensus CAGCTGCTCATACAAACTTCAGTACAAAATCAATGAGGAAAGAAGGAGGATTGGACTTGATAAAAAAAGCAATAGGGAAGCTTGAAGTGAAGCATAAACAACACATTGCTGCTTATGGTGAAGGCAATGA

                   2081  2090      2100      2110      2120      2130      2140      2150      2160      2170      2180      2190      2200      2210
                   |--------+---------+---------+---------+---------+---------+---------+---------+---------+---------+---------+---------+---------|
     BnaC.GLN1.5.a GAGGCGCCTCACTGGGAAGCATGAAACCGCAGACATCAACAAGTTCTCTTGGGTATGAGATATCGAATAAAATCAAATATATAATTTTGGATTAGTGATGCTTATACAAGCTTTCTTGATGTGTGTAATT
mRNA.BnaC.GLN1.5.a GAGGCGCCTCACTGGGAAGCATGAAACCGCAGACATCAACAAGTTCTCTTGGG-----------------------------------------------------------------------------
      BnaGln1.5_C1 GAGGCGCCTCACTGGGAAGCATGAAACCGCAGACATCAACAAGTTCTCTTGGG-----------------------------------------------------------------------------
         Consensus GAGGCGCCTCACTGGGAAGCATGAAACCGCAGACATCAACAAGTTCTCTTGGG.............................................................................

                   2211  2220      2230      2240      2250      2260      2270      2280      2290      2300      2310      2320      2330      2340
                   |--------+---------+---------+---------+---------+---------+---------+---------+---------+---------+---------+---------+---------|
     BnaC.GLN1.5.a TGGCTTTGTTGGGAGTAGGGAGTTGCGGATCGTGGAGCATCGGTGAGAGTGGGAAGAGATACGGAGAAAGAAGGGAAAGGGTATTTTGAAGATCGAAGGCCTTCGTCTAATATGGATCCTTATCTTGTTA
mRNA.BnaC.GLN1.5.a -------------------GAGTTGCGGATCGTGGAGCATCGGTGAGAGTGGGAAGAGATACGGAGAAAGAAGGGAAAGGGTATTTTGAAGATCGAAGGCCTTCGTCTAATATGGATCCTTATCTTGTTA
      BnaGln1.5_C1 -------------------GAGTTGCGGATCGTGGAGCATCGGTGAGAGTGGGAAGAGATACGGAGAAAGAAGGGAAAGGGTATTTTGAAGATCGAAGGCCTTCGTCTAATATGGATCCTTATCTTGTTA
         Consensus ...................GAGTTGCGGATCGTGGAGCATCGGTGAGAGTGGGAAGAGATACGGAGAAAGAAGGGAAAGGGTATTTTGAAGATCGAAGGCCTTCGTCTAATATGGATCCTTATCTTGTTA

                   2341  2350      2360      2370      2380      2390      2400      2410      2420      2430      2440      2450      2460      2470
                   |--------+---------+---------+---------+---------+---------+---------+---------+---------+---------+---------+---------+---------|
     BnaC.GLN1.5.a CCTCCATGATAGCTGAAACCACCATCCTCGGCTAAGCTTTCGTTTGAAGTTGTTGCATACGTTCTTTTGTTTCTTCATGTTTCGGTTTAATTTCAGTTTGAGACTCTTTTTTTTATTGCTAATAATTCAT
mRNA.BnaC.GLN1.5.a CCTCCATGATAGCTGAAACCACCATCCTCGGCTAAGCTTTCGTTTGAAGTTGTTGCATACGTTCTTTTGTTTCTTCATGTTTCGGTTTAATTTCAGTTTGAGACTCTTTTTTTTATTGCTAATAATTCAT
      BnaGln1.5_C1 CCTCCATGATAGCTGAAACCACCATCCTCGGCTAAGCTTTCGTTTGAAGTTGTTGCATACGTTCTTTTGTTTCTTCATGTTTCGGTTTAATTTCAGTTTGAGACTCTTTTTTTTATTGCTAATAATTCAT
         Consensus CCTCCATGATAGCTGAAACCACCATCCTCGGCTAAGCTTTCGTTTGAAGTTGTTGCATACGTTCTTTTGTTTCTTCATGTTTCGGTTTAATTTCAGTTTGAGACTCTTTTTTTTATTGCTAATAATTCAT

                   2471  2480      2490      2500      2510      2520      2530      2540      2550      2560      2570      2580      2590593
                   |--------+---------+---------+---------+---------+---------+---------+---------+---------+---------+---------+---------+--|
     BnaC.GLN1.5.a GGGATGGTCTTGATCCTATTGTTTGTTTATCCTGGTTCAGTTGTTTGTGTTAAAACAAAATTGAATTGGGAAAATTGAATTGGCAAAATAAA
mRNA.BnaC.GLN1.5.a GGGATGGTCTTGATCCTATTGTTTGTTTATCCTGGTTCAGTTGTTTGTGTTAAAACAAAATTGAATTGGGAAAATTGAATTGGCAAAATAAA
      BnaGln1.5_C1 GGGATGGTCTTGATCCTATTGTTTGTTTATCCTGGTTCAGTTGTTTGTGTTAAAACAAAATTGAATTGGGAAAATTGAATTGGCAAAATAAAGGTTCTTAGTTCTTACTTTTAAAAAAAAAAA
         Consensus GGGATGGTCTTGATCCTATTGTTTGTTTATCCTGGTTCAGTTGTTTGTGTTAAAACAAAATTGAATTGGGAAAATTGAATTGGCAAAATAAA..............................
```

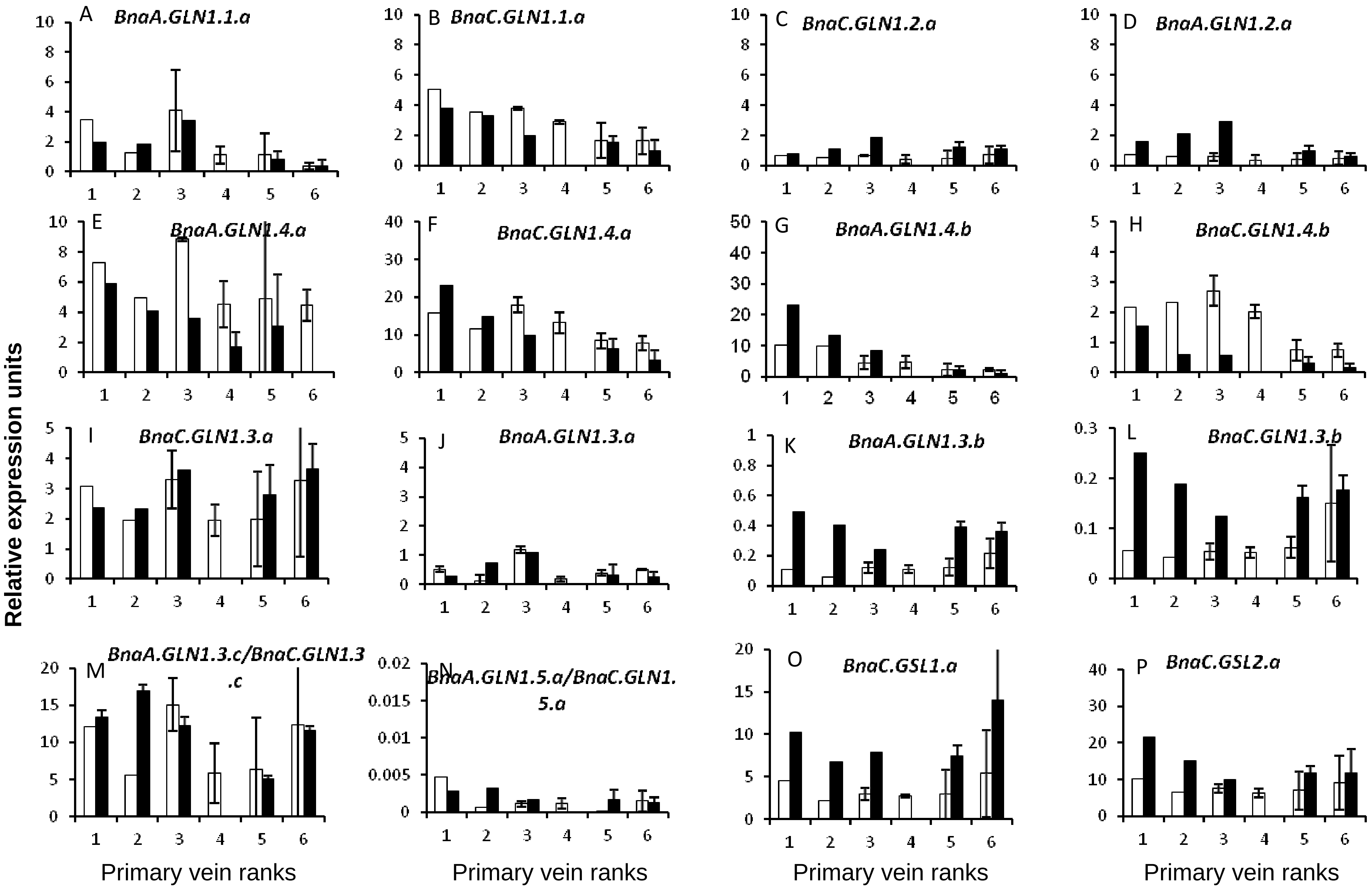

Supplementary Figure S5: Expression of *BnaGLN1* genes is modified depending on nitrate availability and ageing in primary veins of vegetative *B. napus* plants.

BnaGLN1 genes relative expression level was monitored in primary veins of six leaf ranks harvested on vegetative plants grown under low (white bars) or high (black bars) nitrate conditions. Leaf ranks represented as number 1 (bottom and older leaf) to 6 (top and younger leaf) showed differential senescence symptoms. Mean and standard deviation of four plant repeats are shown.
