# Supplementary material for: Sixteen cytosolic glutamine synthetase genes identified in the Brassica napus L. genome are differentially regulated depending on nitrogen regimes and leaf senescence
Source: J Exp Bot. 2014 Feb 24;65(14):3927–47. doi: 10.1093/jxb/eru041 (PMC4106436; doi:10.1093/jxb/eru041)
Supplement: Supplementary Data [file supp_eru041_jexbot114215_file003.pdf]

BnaGLN1_JXB

```
                                                        20                            40                            60                            80
At5g37600_GLN1.1__cds  --- --- --- --- --- --- --- --- --- --- --- --- --- --- --- --- --- --- --- --- --- --- --- --- --- --- --- --- --- --- -
BnaGLN1.1_C1           --- --- --- --- --- --- --- --- --- --- --- --- --- --- --- --- --- --- --- --- --- --- --- --- --- --- --- --- --- --- -
X82997                 --- --- --- --- --- --- --- --- --- --- --- --- --- --- --- --- --- --- --- --- --- --- --- --- --- --- --- --- --- --- -
Bra028132_cds          --- --- --- --- --- --- --- --- --- --- --- --- --- --- --- --- --- --- --- --- --- --- --- --- --- --- --- --- --- --- -
BraGLN1.1_C1           --- --- --- --- --- --- --- --- --- --- --- --- --- --- --- --- --- --- --- --- --- --- --- --- --- --- --- --- --- --- -
BnaGLN1.1_C2           --- --- --- --- --- --- --- --- --- --- --- --- --- --- --- --- --- --- --- --- --- --- --- --- --- --- --- --- --- --- -
Y12460                 --- --- --- --- --- --- --- --- --- --- --- --- --- --- --- --- --- --- --- --- --- --- --- --- --- --- --- --- --- --- -
BolGLN1.1_C1           --- --- --- --- --- --- --- --- --- --- --- --- --- --- --- --- --- --- --- --- --- --- --- --- --- --- --- --- --- --- -
At1g66200.1_GLN1.2__cds --- --- --- --- --- --- --- --- --- --- --- --- --- --- --- --- --- --- --- --- --- --- --- --- --- --- --- --- --- --- -
BnaGLN1.2_C1           --- --- --- --- --- --- --- --- --- --- --- --- --- --- --- --- --- --- --- --- --- --- --- --- --- --- --- --- --- --- -
X76736                 --- --- --- --- --- --- --- --- --- --- --- --- --- --- --- --- --- --- --- --- --- --- --- --- --- --- --- --- --- --- -
Bra039756_cds          --- --- --- --- --- --- --- --- --- --- --- --- --- --- --- --- --- --- --- --- --- --- --- --- --- --- --- --- --- --- -
BraGLN1.2_C1           --- --- --- --- --- --- --- --- --- --- --- --- --- --- --- --- --- --- --- --- --- --- --- --- --- --- --- --- --- --- -
EU499383               --- --- --- --- --- --- --- --- --- --- --- --- --- --- --- --- --- --- --- --- --- --- --- --- --- --- --- --- --- --- -
AY773089               --- --- --- --- --- --- --- --- --- --- --- --- --- --- --- --- --- --- --- --- --- --- --- --- --- --- --- --- --- --- -
BnaGLN1.2_C2           --- --- --- --- --- --- --- --- --- --- --- --- --- --- --- --- --- --- --- --- --- --- --- --- --- --- --- --- --- --- -
Y12459                 --- --- --- --- --- --- --- --- --- --- --- --- --- --- --- --- --- --- --- --- --- --- --- --- --- --- --- --- --- --- -
BolGLN1.2_C1           --- --- --- --- --- --- --- --- --- --- --- --- --- --- --- --- --- --- --- --- --- --- --- --- --- --- --- --- --- --- -
EU822334               --- --- --- --- --- --- --- --- --- --- --- --- --- --- --- --- --- --- --- --- --- --- --- --- --- --- --- --- --- --- -
EU822335               --- --- --- --- --- --- --- --- --- --- --- --- --- --- --- --- --- --- --- --- --- --- --- --- --- --- --- --- --- --- -
At3g17820_GLN1.3__cds  --- --- --- --- --- --- --- --- --- --- --- --- --- --- --- --- --- --- --- --- --- --- --- --- --- --- --- --- --- --- -
BnaGLN1.3_C2           --- --- --- --- --- --- --- --- --- --- --- --- --- --- --- --- --- --- --- --- --- --- --- --- --- --- --- --- --- --- -
JX306693               --- --- --- --- --- --- --- --- --- --- --- --- --- --- --- --- --- --- --- --- --- --- --- --- --- --- --- --- --- --- -
Bra022247_cds          --- --- --- --- --- --- --- --- --- --- --- --- --- --- --- --- --- --- --- --- --- --- --- --- --- --- --- --- --- --- -
BraGLN1.3_C1           --- --- --- --- --- --- --- --- --- --- --- --- --- --- --- --- --- --- --- --- --- --- --- --- --- --- --- --- --- --- -
BnaGLN1.3_C1           --- --- --- --- --- --- --- --- --- --- --- --- --- --- --- --- --- --- --- --- --- --- --- --- --- --- --- --- --- --- -
JX306690               --- --- --- --- --- --- --- --- --- --- --- --- --- --- --- --- --- --- --- --- --- --- --- --- --- --- --- --- --- --- -
BolGLN1.3_C1           --- --- --- --- --- --- --- --- --- --- --- --- --- --- --- --- --- --- --- --- --- --- --- --- --- --- --- --- --- --- -
BnaGLN1.3_C4           --- --- --- --- --- --- --- --- --- --- --- --- --- --- --- --- --- --- --- --- --- --- --- --- --- --- --- --- --- --- -
Bra021276_cds          --- --- --- --- --- --- --- --- --- --- --- --- --- --- --- --- --- --- --- --- --- --- --- --- --- --- --- --- --- --- -
BraGLN1.3_C3           --- --- --- --- --- --- --- --- --- --- --- --- --- --- --- --- --- --- --- --- --- --- --- --- --- --- --- --- --- --- -
BnaGLN1.3_C3           --- --- --- --- --- --- --- --- --- --- --- --- --- --- --- --- --- --- --- --- --- --- --- --- --- --- --- --- --- --- -
BnaGLN1.3_C5           --- --- --- --- --- --- --- --- --- --- --- --- --- --- --- --- --- --- --- --- --- --- --- --- --- --- --- --- --- --- -
JX306694               --- --- --- --- --- --- --- --- --- --- --- --- --- --- --- --- --- --- --- --- --- --- --- --- --- --- --- --- --- --- -
Bra001686_cds          --- --- --- --- --- --- --- --- --- --- --- --- --- --- --- --- --- --- --- --- --- --- --- --- --- --- --- --- --- --- -
BraGLN1.3_C2           TTT TTT TTA TTC TCT CTC ATT ATC TCT GTT TTT TAC TAT TGC TGT GAT CGA AAA GCT TTG CCT TAT GCT ATT GAT CTC TTA TGT TTT TGA 90
BnaGLN1.3_C6           --- --- --- --- --- --- --- --- --- --- --- --- --- --- --- --- --- --- --- --- --- --- --- --- --- --- --- --- --- --- -
BolGLN1.3_C2           --- --- --- --- --- --- --- --- --- --- --- --- --- --- --- --- --- --- --- --- --- --- --- --- --- --- --- --- --- --- -
At5g16570_GLN1.4__cds  --- --- --- --- --- --- --- --- --- --- --- --- --- --- --- --- --- --- --- --- --- --- --- --- --- --- --- --- --- --- -
BnaGLN1.4_C1           --- --- --- --- --- --- --- --- --- --- --- --- --- --- --- --- --- --- --- --- --- --- --- --- --- --- --- --- --- --- -
JX306697               --- --- --- --- --- --- --- --- --- --- --- --- --- --- --- --- --- --- --- --- --- --- --- --- --- --- --- --- --- --- -
JX306692               --- --- --- --- --- --- --- --- --- --- --- --- --- --- --- --- --- --- --- --- --- --- --- --- --- --- --- --- --- --- -
Bra023573_cds          --- --- --- --- --- --- --- --- --- --- --- --- --- --- --- --- --- --- --- --- --- --- --- --- --- --- --- --- --- --- -
BraGLN1.4_C1           --- --- --- --- --- --- --- --- --- --- --- --- --- --- --- --- --- --- --- --- --- --- --- --- --- --- --- --- --- --- -
BnaGLN1.4_C2           --- --- --- --- --- --- --- --- --- --- --- --- --- --- --- --- --- --- --- --- --- --- --- --- --- --- --- --- --- --- -
JX306695               --- --- --- --- --- --- --- --- --- --- --- --- --- --- --- --- --- --- --- --- --- --- --- --- --- --- --- --- --- --- -
JX306696               --- --- --- --- --- --- --- --- --- --- --- --- --- --- --- --- --- --- --- --- --- --- --- --- --- --- --- --- --- --- -
BolGLN1.4_C1           --- --- --- --- --- --- --- --- --- --- --- --- --- --- --- --- --- --- --- --- --- --- --- --- --- --- --- --- --- --- -
BnaGLN1.4_C4           --- --- --- --- --- --- --- --- --- --- --- --- --- --- --- --- --- --- --- --- --- --- --- --- --- --- --- --- --- --- -
JX306700               --- --- --- --- --- --- --- --- --- --- --- --- --- --- --- --- --- --- --- --- --- --- --- --- --- --- --- --- --- --- -
JX306701               --- --- --- --- --- --- --- --- --- --- --- --- --- --- --- --- --- --- --- --- --- --- --- --- --- --- --- --- --- --- -
```

```
       Bra008612_cds --- --- --- --- --- --- --- --- --- --- --- --- --- --- --- --- --- --- --- --- --- --- --- --- --- --- --- --- --- --- -
        BraGLN1.4_C2 --- --- --- --- --- --- --- --- --- --- --- --- --- --- --- --- --- --- --- --- --- --- --- --- --- --- --- --- --- --- -
        BnaGLN1.4_C3 --- --- --- --- --- --- --- --- --- --- --- --- --- --- --- --- --- --- --- --- --- --- --- --- --- --- --- --- --- --- -
            JX306698 --- --- --- --- --- --- --- --- --- --- --- --- --- --- --- --- --- --- --- --- --- --- --- --- --- --- --- --- --- --- -
            JX306699 --- --- --- --- --- --- --- --- --- --- --- --- --- --- --- --- --- --- --- --- --- --- --- --- --- --- --- --- --- --- -
        BolGln1.4_C2 --- --- --- --- --- --- --- --- --- --- --- --- --- --- --- --- --- --- --- --- --- --- --- --- --- --- --- --- --- --- -
At1g48470_GLN1.5__cds --- --- --- --- --- --- --- --- --- --- --- --- --- --- --- --- --- --- --- --- --- --- --- --- --- --- --- --- --- --- -
        BnaGLN1.5_C2 --- --- --- --- --- --- --- --- --- --- --- --- --- --- --- --- --- --- --- --- --- --- --- --- --- --- --- --- --- --- -
       Bra018729_cds --- --- --- --- --- --- --- --- --- --- --- --- --- --- --- --- --- --- --- --- --- --- --- --- --- --- --- --- --- --- -
        BraGLN1.5_C1 --- --- --- --- --- --- --- --- --- --- --- --- --- --- --- --- --- --- --- --- --- --- --- --- --- --- --- --- --- --- -
        BnaGLN1.5_C1 --- --- --- --- --- --- --- --- --- --- --- --- --- --- --- --- --- --- --- --- --- --- --- --- --- --- --- --- --- --- -
            JX306691 --- --- --- --- --- --- --- --- --- --- --- --- --- --- --- --- --- --- --- --- --- --- --- --- --- --- --- --- --- --- -
        BolGLN1.5_C1 --- --- --- --- --- --- --- --- --- --- --- --- --- --- --- --- --- --- --- --- --- --- --- --- --- --- --- --- --- --- -
```

BnaGLN1_JXB

	100 · 120 · 140 · 160 · 180
At5g37600_GLN1.1__cds	--- -
BnaGLN1.1_C1	--- -
X82997	--- -
Bra028132_cds	--- -
BraGLN1.1_C1	--- -
BnaGLN1.1_C2	--- -
Y12460	--- -
BolGLN1.1_C1	--- -
At1g66200.1_GLN1.2__cds	--- -
BnaGLN1.2_C1	--- -
X76736	--- -
Bra039756_cds	--- -
BraGLN1.2_C1	--- -
EU499383	--- -
AY773089	--- -
BnaGLN1.2_C2	--- -
Y12459	--- -
BolGLN1.2_C1	--- -
EU822334	--- -
EU822335	--- -
At3g17820_GLN1.3__cds	--- -
BnaGLN1.3_C2	--- -
JX306693	--- -
Bra022247_cds	--- -
BraGLN1.3_C1	--- -
BnaGLN1.3_C1	--- -
JX306690	--- -
BolGLN1.3_C1	--- -
BnaGLN1.3_C4	--- -
Bra021276_cds	--- -
BraGLN1.3_C3	--- -
BnaGLN1.3_C3	--- -
BnaGLN1.3_C5	--- -
JX306694	--- -
Bra001686_cds	--- -
BraGLN1.3_C2	**CAA CTT CGG TTT GAA ACA CGA TTC ACT TCA CTC TGT TTT CGC TTG TTC TGT CTC GAT GAG ATT GAC GTG TAC ATC GTA AA- GCT TGT CTC** 179
BnaGLN1.3_C6	--- -
BolGLN1.3_C2	--- -
At5g16570_GLN1.4__cds	--- -
BnaGLN1.4_C1	--- -
JX306697	--- -
JX306692	--- -
Bra023573_cds	--- -
BraGLN1.4_C1	--- -
BnaGLN1.4_C2	--- -
JX306695	--- -
JX306696	--- -
BolGLN1.4_C1	--- -
BnaGLN1.4_C4	--- -
JX306700	--- -
JX306701	--- -

```
      Bra008612_cds --- --- --- --- --- --- --- --- --- --- --- --- --- --- --- --- --- --- --- --- --- --- --- --- --- --- --- --- --- ----
      BraGLN1.4_C2 --- --- --- --- --- --- --- --- --- --- --- --- --- --- --- --- --- --- --- --- --- --- --- --- --- --- --- --- --- ----
      BnaGLN1.4_C3 --- --- --- --- --- --- --- --- --- --- --- --- --- --- --- --- --- --- --- --- --- --- --- --- --- --- --- --- --- ----
          JX306698 --- --- --- --- --- --- --- --- --- --- --- --- --- --- --- --- --- --- --- --- --- --- --- --- --- --- --- --- --- ----
          JX306699 --- --- --- --- --- --- --- --- --- --- --- --- --- --- --- --- --- --- --- --- --- --- --- --- --- --- --- --- --- ----
      BolGln1.4_C2 --- --- --- --- --- --- --- --- --- --- --- --- --- --- --- --- --- --- --- --- --- --- --- --- --- --- --- --- --- ----
At1g48470_GLN1.5__cds --- --- --- --- --- --- --- --- --- --- --- --- --- --- --- --- --- --- --- --- --- --- --- --- --- --- --- --- --- ----
      BnaGLN1.5_C2 --- --- --- --- --- --- --- --- --- --- --- --- --- --- --- --- --- --- --- --- --- --- --- --- --- --- --- --- --- ----
      Bra018729_cds --- --- --- --- --- --- --- --- --- --- --- --- --- --- --- --- --- --- --- --- --- --- --- --- --- --- --- --- --- ----
      BraGLN1.5_C1 --- --- --- --- --- --- --- --- --- --- --- --- --- --- --- --- --- --- --- --- --- --- --- --- --- --- --- --- --- ----
      BnaGLN1.5_C1 --- --- --- --- --- --- --- --- --- --- --- --- --- --- --- --- --- --- --- --- --- --- --- --- --- --- --- --- --- ----
          JX306691 --- --- --- --- --- --- --- --- --- --- --- --- --- --- --- --- --- --- --- --- --- --- --- --- --- --- --- --- --- ----
      BolGLN1.5_C1 --- --- --- --- --- --- --- --- --- --- --- --- --- --- --- --- --- --- --- --- --- --- --- --- --- --- --- --- --- ----
```

BnaGLN1_JXB

```
                                                   200                           220                           240                           260
At5g37600_GLN1.1__cds --- --- --- --- --- --- --- --- --- --- --- --- --- --- --- --- --- --- --- --- --- --- --- --- --- --- --- --- --- ---- -
BnaGLN1.1_C1          --- --- --- --- --- --- --- --- --- --- --- --- --- --- --- --- --- --- --- --- --- --- --- --- --- --- --- --- --- ---- -
X82997                --- --- --- --- --- --- --- --- --- --- --- --- --- --- --- --- --- --- --- --- --- --- --- --- --- --- --- --- --- ---- -
Bra028132_cds         --- --- --- --- --- --- --- --- --- --- --- --- --- --- --- --- --- --- --- --- --- --- --- --- --- --- --- --- --- ---- -
BraGLN1.1_C1          --- --- --- --- --- --- --- --- --- --- --- --- --- --- --- --- --- --- --- --- --- --- --- --- --- --- --- --- --- ---- -
BnaGLN1.1_C2          --- --- --- --- --- --- --- --- --- --- --- --- --- --- --- --- --- --- --- --- --- --- --- --- --- --- --- --- --- ---- -
Y12460                --- --- --- --- --- --- --- --- --- --- --- --- --- --- --- --- --- --- --- --- --- --- --- --- --- --- --- --- --- ---- -
BolGLN1.1_C1          --- --- --- --- --- --- --- --- --- --- --- --- --- --- --- --- --- --- --- --- --- --- --- --- --- --- --- --- --- ---- -
At1g66200.1_GLN1.2__cds --- --- --- --- --- --- --- --- --- --- --- --- --- --- --- --- --- --- --- --- --- --- --- --- --- --- --- --- --- ---- -
BnaGLN1.2_C1          --- --- --- --- --- --- --- --- --- --- --- --- --- --- --- --- --- --- --- --- --- --- --- --- --- --- --- --- --- ---- -
X76736                --- --- --- --- --- --- --- --- --- --- --- --- --- --- --- --- --- --- --- --- --- --- --- --- --- --- --- --- --- ---- -
Bra039756_cds         --- --- --- --- --- --- --- --- --- --- --- --- --- --- --- --- --- --- --- --- --- --- --- --- --- --- --- --- --- ---- -
BraGLN1.2_C1          --- --- --- --- --- --- --- --- --- --- --- --- --- --- --- --- --- --- --- --- --- --- --- --- --- --- --- --- --- ---- -
EU499383              --- --- --- --- --- --- --- --- --- --- --- --- --- --- --- --- --- --- --- --- --- --- --- --- --- --- --- --- --- ---- -
AY773089              --- --- --- --- --- --- --- --- --- --- --- --- --- --- --- --- --- --- --- --- --- --- --- --- --- --- --- --- --- ---- -
BnaGLN1.2_C2          --- --- --- --- --- --- --- --- --- --- --- --- --- --- --- --- --- --- --- --- --- --- --- --- --- --- --- --- --- ---- -
Y12459                --- --- --- --- --- --- --- --- --- --- --- --- --- --- --- --- --- --- --- --- --- --- --- --- --- --- --- --- --- ---- -
BolGLN1.2_C1          --- --- --- --- --- --- --- --- --- --- --- --- --- --- --- --- --- --- --- --- --- --- --- --- --- --- --- --- --- ---- -
EU822334              --- --- --- --- --- --- --- --- --- --- --- --- --- --- --- --- --- --- --- --- --- --- --- --- --- --- --- --- --- ---- -
EU822335              --- --- --- --- --- --- --- --- --- --- --- --- --- --- --- --- --- --- --- --- --- --- --- --- --- --- --- --- --- ---- -
At3g17820_GLN1.3__cds --- --- --- --- --- --- --- --- --- --- --- --- --- --- --- --- --- --- --- --- --- --- --- --- --- --- --- --- --- ---- -
BnaGLN1.3_C2          --- --- --- --- --- --- --- --- --- --- --- --- --- --- --- --- --- --- --- TGA ATG CTG TGA CGC CAG CTC GAA ATA ACC CTA 33
JX306693              --- --- --- --- --- --- --- --- --- --- --- --- --- --- --- --- --- --- --- --- --- --- --- --- --- --- --- --- --- ---- -
Bra022247_cds         --- --- --- --- --- --- --- --- --- --- --- --- --- --- --- --- --- --- --- --- --- --- --- --- --- --- --- --- --- ---- -
BraGLN1.3_C1          --- --- --- --- --- --- --- --- --- --- --- --- --- --- --- --- --- --- --- --- --- --- --- --- --- --- --- --- --- ---- -
BnaGLN1.3_C1          --- --- --- --- --- --- --- --- --- --- --- --- --- --- --- --- --- --- --- --- --- --- --- --- --- --- --- --- --- ---- -
JX306690              --- --- --- --- --- --- --- --- --- --- --- --- --- --- --- --- --- --- --- --- --- --- --- --- --- --- --- --- --- ---- -
BolGLN1.3_C1          --- --- --- --- --- --- --- --- --- --- --- --- --- --- --- --- --- --- --- --- --- --- --- --- --- --- --- --- --- ---- -
BnaGLN1.3_C4          --- --- --- --- --- --- --- --- --- --- --- --- --- --- --- --- --- --- --- --- --- --- --- --- --- --- --- --- --- ---- -
Bra021276_cds         --- --- --- --- --- --- --- --- --- --- --- --- --- --- --- --- --- --- --- --- --- --- --- --- --- --- --- --- --- ---- -
BraGLN1.3_C3          --- --- --- --- --- --- --- --- --- --- --- --- --- --- --- --- --- --- --- --- --- --- --- --- --- --- --- --- --- ---- -
BnaGLN1.3_C3          --- --- --- --- --- --- --- --- --- --- --- --- --- --- --- --- --- --- --- --- --- --- --- --- --- --- --- --- --- ---- -
BnaGLN1.3_C5          --- --- --- --- --- --- --- --- --- --- --- --- --- --- --- --- --- --- --- --- --- --- --- --- --- --- --- --- --- ---- -
JX306694              --- --- --- --- --- --- --- --- --- --- --- --- --- --- --- --- --- --- --- --- --- --- --- --- --- --- --- --- --- ---- -
Bra001686_cds         --- --- --- --- --- --- --- --- --- --- --- --- --- --- --- --- --- --- --- --- --- --- --- --- --- --- --- --- --- ---- -
BraGLN1.3_C2          TTT CTT TGT TGT CGT CTT GTG GGT TTG TAA TTT TCT CGT ATG AAT GTT GAA TGA ACA --- --- --- --- --- --- --- --- --- --- ---- 236
BnaGLN1.3_C6          --- --- --- --- --- --- --- --- --- --- --- --- --- --- --- --- --- --- --- --- --- --- --- --- --- --- --- --- --- ---- -
BolGLN1.3_C2          --- --- --- --- --- --- --- --- --- --- --- --- --- --- --- --- --- --- --- --- --- --- --- --- --- --- --- --- --- ---- -
At5g16570_GLN1.4__cds --- --- --- --- --- --- --- --- --- --- --- --- --- --- --- --- --- --- --- --- --- --- --- --- --- --- --- --- --- ---- -
BnaGLN1.4_C1          --- --- --- --- --- --- --- --- --- --- --- --- --- --- --- --- --- --- --- --- --- --- --- --- --- --- --- --- --- ---- -
JX306697              --- --- --- --- --- --- --- --- --- --- --- --- --- --- --- --- --- --- --- --- --- --- --- --- --- --- --- --- --- ---- -
JX306692              --- --- --- --- --- --- --- --- --- --- --- --- --- --- --- --- --- --- --- --- --- --- --- --- --- --- --- --- --- ---- -
Bra023573_cds         --- --- --- --- --- --- --- --- --- --- --- --- --- --- --- --- --- --- --- --- --- --- --- --- --- --- --- --- --- ---- -
BraGLN1.4_C1          --- --- --- --- --- --- --- --- --- --- --- --- --- --- --- --- --- --- --- --- --- --- --- --- --- --- --- -AA GCA CCG 8
BnaGLN1.4_C2          --- --- --- --- --- --- --- --- --- --- --- --- --- --- --- --- --- --- --- --- --- --- --- --- --- --- --- --- --- ---- -
JX306695              --- --- --- --- --- --- --- --- --- --- --- --- --- --- --- --- --- --- --- --- --- --- --- --- --- --- --- --- --- ---- -
JX306696              --- --- --- --- --- --- --- --- --- --- --- --- --- --- --- --- --- --- --- --- --- --- --- --- --- --- --- --- --- ---- -
BolGLN1.4_C1          --- --- --- --- --- --- --- --- --- --- --- --- --- --- --- --- --- --- --- --- --- --- --- --- --- --- --- --- --- ---- -
BnaGLN1.4_C4          --- --- --- --- --- --- --- --- --- --- --- --- --- --- --- --- --- --- --- --- --- --- --- --- --- --- --- --- --- ---- -
JX306700              --- --- --- --- --- --- --- --- --- --- --- --- --- --- --- --- --- --- --- --- --- --- --- --- --- --- --- --- --- ---- -
JX306701              --- --- --- --- --- --- --- --- --- --- --- --- --- --- --- --- --- --- --- --- --- --- --- --- --- --- --- --- --- ---- -
```

```
Bra008612_cds          --- --- --- --- --- --- --- --- --- --- --- --- --- --- --- --- --- --- --- --- --- --- --- --- --- --- --- --- --- --- -
BraGLN1.4_C2           --- --- --- --- --- --- --- --- --- --- --- --- --- --- --- --- --- --- --- --- --- --- --- --- --- --- --- --- --- --- -
BnaGLN1.4_C3           --- --- --- --- --- --- --- --- --- --- --- --- --- --- --- --- --- --- --- --- --- --- --- --- --- --- --- --- --- --- -
JX306698               --- --- --- --- --- --- --- --- --- --- --- --- --- --- --- --- --- --- --- --- --- --- --- --- --- --- --- --- --- --- -
JX306699               --- --- --- --- --- --- --- --- --- --- --- --- --- --- --- --- --- --- --- --- --- --- --- --- --- --- --- --- --- --- -
BolGln1.4_C2           --- --- --- --- --- --- --- --- --- --- --- --- --- --- --- --- --- --- --- --- --- --- --- --- --- --- --- --- --- --- -
At1g48470_GLN1.5__cds  --- --- --- --- --- --- --- --- --- --- --- --- --- --- --- --- --- --- --- --- --- --- --- --- --- --- --- --- --- --- -
BnaGLN1.5_C2           --- --- --- --- --- --- --- --- --- --- --- --- --- --- --- --- --- --- --- --- --- --- --- --- --- -GG ATC TCC GGA TAG 14
Bra018729_cds          --- --- --- --- --- --- --- --- --- --- --- --- --- --- --- --- --- --- --- --- --- --- --- --- --- --- --- --- --- --- -
BraGLN1.5_C1           --- --- --- --- --- --- --- --- --- --- --- --- --- --- --- --- --- --- --- --- --- --- --- --- --- --- --- --- --- --- -
BnaGLN1.5_C1           --- --- --- --- --- --- --- --- --- --- --- --- --- --- --- --- --- --- --- --- --- --- --- --- --- --- --- --- --- --- -
JX306691               --- --- --- --- --- --- --- --- --- --- --- --- --- --- --- --- --- --- --- --- --- --- --- --- --- --- --- --- --- --- -
BolGLN1.5_C1           --- --- --- --- --- --- --- --- --- --- --- --- --- --- --- --- --- --- --- --- --- --- --- --- --- --- --- --- --- --- -
```

```
                        280                      300                      320                      340                      360
At5g37600_GLN1.1__cds --- --- --- --- --- --- --- --- --- --- --- --- --- --- --- --- --- --- --- --- --- --- --- --- --- --- --- --- --- --- -
BnaGLN1.1_C1          --- --- --- --- --- --- --- --- --A CAC AAC CAA AAC ACG AGA AGA TTT TAT TCG ATT TTA TCT GTC ATT TGC TTC AAC TGT TAC TAT 64
X82997                --- --- --- --- --- --- --- --- --- --- --- --- --- --- --- --- --T TCG ATT TTA TCT GTC ATT TGC TTC --- AAC TGT TAC TAT 37
Bra028132_cds         --- --- --- --- --- --- --- --- --- --- --- --- --- --- --- --- --- --- --- --- --- --- --- --- --- --- --- --- --- --- -
BraGLN1.1_C1          --- --- --- --- --- --- --- --A AAA CAC AAC CAA AAC ACG AGA AGA TTT TAT TCG ATT TTA TCT GTC ATT TGC TTC AAC TGT TAC TAT 67
BnaGLN1.1_C2          --- --- --- --- --- --- --- --- --A CAC AAC CAA AAC ACG AGA AGA TTT TAT TCG ATT TTA TCT GTC ATT TGC TTC AGC TGT TAC TAT 64
Y12460                --- --- --- --- --- --- --- --- --- --- --- --- --- --- --- --- --- --- --- --- --- --- --- --- --- --- --- --- --- --- -
BolGLN1.1_C1          --- --- --- --- --- --- --- --- --- --- --A GCG TCC GCG AGA AGA TTT TAT TCG ATT TTA TCT GTC ATT TGC TTC AGC TGT TAC TAT 58
At1g66200.1_GLN1.2__cds --- --- --- --- --- --- --- --- --- --- --- --- --- --- --- --- --- --- --- --- --- --- --- --- --- --- --- --- --- --- -
BnaGLN1.2_C1          --- --- --- --- --- --- --- --- -GG GTC GAC CCA CGC GTC CGC AAA AAC CAC ATT ATC CGA GAT TTG AGT ATA --- TTT CAT TGC AAC 62
X76736                --- --- --- --- --- --- --- --- --- --- --- --- --- --- --C AAA AAC CAC ATT ATC CGA GAT TTG AGT ATA --- TTT CAT TGC AAC 43
Bra039756_cds         --- --- --- --- --- --- --- --- --- --- --- --- --- --- --- --- --- --- --- --- --- --- --- --- --- --- --- --- --- --- -
BraGLN1.2_C1          --- --- --- --- --- --- --- --- --- --G ATC ATC TCA TAA ACC AAA AAC CAC ATT ATC CGA GAT TTG AGT ATA --- TTT CAT TGC AAC 58
EU499383              --- --- --- --- --- --- --- --- --- CAA AAA CCA CAT TAT ATC AAA AAC CAC ATT ATC CGA GAT TTG AGT ATA --- TTT CAT TGC AAC 60
AY773089              --- --- --- --- --- --- --- --A CGC GGG ATC ATC TCA TAA ACC AAA AAC CAC ATT ATC CGA GAT TTG AGT ATA --- TTT CAT TGC AAC 64
BnaGLN1.2_C2          --- --- --- --- --- --- --- --- -CG GGG CGG ATC TCA TAA ACC AAA AAC CAC ATT ATC CGA GAT TTG AGT ATA ATA TTT CAC TAC AAC 65
Y12459                --- --- --- --- --- --- --- --- --- --- --- --- --- --- --- --- --- --- --- --- --- --- --- --- --- --- --- --C TAC AAC 7
BolGLN1.2_C1          --- --- --- --- --- --- --- --- --- --- --- --- --- --- --- --- --- --- --- --- --- --- --- --- --- --- --- --- --- --- -
EU822334              --- --- --- --- --- --- --- --- --- --- --- --- --- --- --- --- --- --- --- --- --- --- --- --- --- --- --- --- --A TAC 4
EU822335              --- --- --- --- --- --- --- --- --- --- --- --- --- --- --- --- --- --- --- --- --- --- --- --- --- --- --- --- --- --C 1
At3g17820_GLN1.3__cds --- --- --- --- --- --- --- --- --- --- --- --- --- --- --- --- --- --- --- --- --- --- --- --- --- --- --- --- --- --- -
BnaGLN1.3_C2          CTA AAG GGA ACA AAA GCT GGA GCT CCA CCG CGG TGG CGG CCG CTC TGG CAT TGA TCG TCT TCC CTT AGA CAA ACA CTG ATT GAT TAT CTT 123
JX306693              --- --- --- --- --- --- --- --- --- --- --- --- --- --- --- --- --- --- --- --- --- --- --- --- --- --- --- --- --- --- -
Bra022247_cds         --- --- --- --- --- --- --- --- --- --- --- --- --- --- --- --- --- --- --- --- --- --- --- --- --- --- --- --- --- --- -
BraGLN1.3_C1          --- --- --- --- --- --- --- --- --- --- --- --- --- --- --A ATT GAT CGT CTT CCC CCT AAA CAA TAA ACA CTG ATT GAT TAT CTT 46
BnaGLN1.3_C1          --- --- --- --- --- --- --- --- --- --- --- --- --- --- --- --G GGT CGA GGG ATT GAT CGT CTT CCC CTA AAC AAC ACT GAT TGA 43
JX306690              --- --- --- --- --- --- --- --- --- --- --- --- --- --- --- --- --- --- --- --- --- --- --- --- --- --- --C ACT GAT TGA 10
BolGLN1.3_C1          --- --- --- --- --- --- --- --- --- --- --- --- --- --- --- --- --- --- --- --- --- --- --- --- CTA AAC AAC ACT GAT TGA 18
BnaGLN1.3_C4          --- --- --- --- --- --- --- --- --- --- --- --- --- --- --- CCA CGC GTC CGC GGA CGC GTG GGA TTT TAG TAT TTT TTT TTC TCT 45
Bra021276_cds         --- --- --- --- --- --- --- --- --- --- --- --- --- --- --- --- --- --- --- --- --- --- --- --- --- --- --- --- --- --- -
BraGLN1.3_C3          --- --- --- --- --- --- --- --- --- --- --- --- --- --- --- --- --- --- --- --- --- --- --- --- --- --- --- --- --- --- -
BnaGLN1.3_C3          --- --- --- --- --- --- --- --- --- --- --- --- --- --- --- --- --- --- --- --- --- --- --- --- --- --- --- --- --- TCT 3
BnaGLN1.3_C5          --- --- --- --- --- --- --- --- --- --- --- GGG TCG ACG GCC ATT ACG GCC GGG GAT CGA GAT CGT CTT CCC AAA ACA ACA CTC ATT 57
JX306694              --- --- --- --- --- --- --- --- --- --- --- --- --- --- --- --- --- --- --- -AT CGA GAT CGT CTT CCC AAA ACA ACA CTC ATT 32
Bra001686_cds         --- --- --- --- --- --- --- --- --- --- --- --- --- --- --- --- --- --- --- --- --- --- --- --- --- --- --- --- --- --- -
BraGLN1.3_C2          --- --- --- --- --- --- --- --- --- --- --- --- --- --- --- --- --- --- --- --- --- --- --- --- --- --- --- --- --- --- 236
BnaGLN1.3_C6          --- --- --- --- --- --- --- --- --- --- --- --- --- --- --- --- --- --- --- --- GGA GAT CGT CTT CCC TAA ACA ACA CTC ATT 30
BolGLN1.3_C2          --- --- --- --- --- --- --- --- --- --- --- --- --- --- --- --- --- --- --- --- --- --- --- --- --- --- --- --- --- --- -
At5g16570_GLN1.4__cds --- --- --- --- --- --- --- --- --- --- --- --- --- --- --- --- --- --- --- --- --- --- --- --- --- --- --- --- --- --- -
BnaGLN1.4_C1          --- --- --- --- --- --- --- --- --- --- --- --- --- --- --- --- --- --- --- --- --- --- --- --- GAT ACA AAA AAA GTT TAG 18
JX306697              --- --- --- --- --- --- --- --- --- --- --- --- --- --- --- --- --- --- --- --- --- --- --- --- --- --- --- --- --- --- -
JX306692              --- --- --- --- --- --- --- --- --- --- --- --- --- --- --- --- --- --- --- --- --- --- --- --- --- --- --- --- --- --- -
Bra023573_cds         --- --- --- --- --- --- --- --- --- --- --- --- --- --- --- --- --- --- --- --- --- --- --- --- --- --- --- --- --- --- -
BraGLN1.4_C1          GCA TCT ACG CAA AGA GGC CAT TAC GTG CCG GTA AGC AGA GGT ATC AAC TGC AGA GTC ACC ATT ACG GGC GGG GAT ACA AAA AAA GTT TAG 98
BnaGLN1.4_C2          --- --- --- --- --- --- --- --- --- --- --- --- --- --- --- --G GGT CGA CGG CCA TTA CGG CCG GGG GAT ACA AAA AAA GTT TAG 43
JX306695              --- --- --- --- --- --- --- --- --- --- --- --- --- --- --- --- --- --- --- --- --- --- --- -AT ACA --- AAA AAA GTT TAG 17
JX306696              --- --- --- --- --- --- --- --- --- --- --- --- --- --- --- --- --- --- --- --- --- --- --- --- --- --- --- --- --- --- -
BolGLN1.4_C1          --- --- --- --- --- --- --- --- --- --- --- --- --- --- --- --- --- --- --- --- --- --- --- --- GAT ACA AAA AAA GTT TAG 18
BnaGLN1.4_C4          --- --- --- --- --- --- --- --- --- --- --- --- --- --- --- --- --- --- --- --- --- --- --- --- --- --- --- --- --- --- -
JX306700              --- --- --- --- --- --- --- --- --- --- --- --- --- --- --- --- --- --- --- --- --- --- --- --- --- --- --- --- --- --- -
JX306701              --- --- --- --- --- --- --- --- --- --- --- --- --- --- --- --- --- --- --- --- --- --- --- --- --- --- --- --- --- --- -
```

```
          Bra008612_cds --- --- --- --- --- --- --- --- --- --- --- --- --- --- --- --- --- --- --- --- --- --- --- --- --- --- --- --- --- --- -
          BraGLN1.4_C2 --- --- --- --- --- --- --- --- --- --- --- --- --- --- --- --- --- --- --- --- --- --- --- AAT ACA AAA CAA AGC GAA ATT 21
          BnaGLN1.4_C3 --- --- --- --- --- --- --- --- --- --- --- --- --- --- --- --- --- --- --- --- --- --- --- -GA TAC AAA ACA AAG CTA ATA 20
              JX306698 --- --- --- --- --- --- --- --- --- --- --- --- --- --- --- --- --- --- --- --- --- --- --- --- --- --- --- --- --- --- -
              JX306699 --- --- --- --- --- --- --- --- --- --- --- --- --- --- --- --- --- --- --- --- --- --- --- --- --- --- --- --- --- --- -
          BolGln1.4_C2 --- --- --- --- --- --- --- --- --- --- --- --- --- --- --- --- --- --- --- --- --- --- --- --- --- --- --- --- --- --- -
At1g48470_GLN1.5__cds --- --- --- --- --- --- --- --- --- --- --- --- --- --- --- --- --- --- --- --- --- --- --- --- --- --- --- --- --- --- -
          BnaGLN1.5_C2 TTC GCC GCT TCT ATA TAA AAA GTG AGT GAG CGA AGC AGA GTG AGC CAG TGC TCA CCT CAG ACT GAT TAT TAT AAC TCG ATC GTC ATC TTC 104
          Bra018729_cds --- --- --- --- --- --- --- --- --- --- --- --- --- --- --- --- --- --- --- --- --- --- --- --- --- --- --- --- --- --- -
          BraGLN1.5_C1 --- --- --- --- --- --- --- --- --- --- --- --- --- --- --- --- --- --- --- --- --- --- --- --- -GC TCG ATC GTC TTC TTC 17
          BnaGLN1.5_C1 --- --- --- --G ATA TAA AAA GTG AGT TAG CGA AGC AGA GTG AGC CAA TGC TCA CCT CAG ACT GAT TAT TAT AAC TCG ATC GTC TTC TTC 79
              JX306691 --- --- --- --- --- --- --- --- --- --- --- --- --- --- --- --- --- --- --- --- --- --- --- --- --- --- --- --- --- --- -
          BolGLN1.5_C1 --- --- --- --- --- --- --- --- --- GAG CGA AGC AGA GTG AGC CAA TGC TCA CCT CAG ACT GAT TAT TAT AAC TCG ATC GTC TTC TTC 63
```

BnaGLN1_JXB

```
                                                                380                                         400                                         420                                         440
At5g37600_GLN1.1__cds   --- --- --- --- --- --- --- --- ATG --- AGT CTT GTT TCA GAT CTC ATC AAC CTT AAC CTC TCA GAC TCC ACT GAC AAA ATC ATT GCT 63
BnaGLN1.1_C1            CGA CCA ATC TCG GTG TTC GTA GCC ATG --- AGT CTT CTT ACA GAT CTC GTT AAC CTT AAC CTC TCA GAG ACC ACT GAC AAA ATC ATT GCG 151
X82997                  CGA CCA ATC TCG GTG TTC GTA GCC ATG --- AGT CTT CTT ACA GAT CTC GTT AAC CTT AAC CTC TCA GAG ACC ACT GAC AAA ATC ATT GCG 124
Bra028132_cds           --- --- --- --- --- --- --- --- ATG --- AGT CTT CTT ACA GAT CTC GTT AAC CTT AAC CTC TCA GAG ACC ACT GAC AAA GTC ATT GCG 63
BraGLN1.1_C1            CGA CCA ATC TCG GTG TTC GTA GCC ATG --- AGT CTT CTT ACA GAT CTC GTT AAC CTT AAC CTC TCA GAG ACC ACT GAC AAA GTC ATT GCG 154
BnaGLN1.1_C2            CGA CCA ATC TCG GTG TTC GTA GCC ATG --- AGT CTT CTT ACA GAT CTC GTT AAC CTT AAC CTC TCA GAG ACC ACT GAC AAA ATC ATT GCG 151
Y12460                  --- --- --- --- --- --- --- --- --- --- --- --- --- --- --- --- GTT AAC CTT AAC CTC TCA GAG ACA ACT GAC AAA ATC ATT GCG 42
BolGLN1.1_C1            CGA CCA ATC TCG GTG TTC GTA GCC ATG --- AGT CTT CTT ACA GAT CTC GTT AAC CTT AAC CTC TCA GAG ACC ACT GAC AAA ATC ATT GCG 145
At1g66200.1_GLN1.2__cds --- --- --- --- --- --- --- --- ATG --- AGT CTT CTT GCA GAT CTT GTT AAC CTT GAC ATC TCA GAC AAC AGT GAA AAG ATC ATC GCT 63
BnaGLN1.2_C1            CTT CTT GTC ATT TTC TCT GTA ACC ATG --- AGT CTT CTG ACC GAT CTC GTT AAC CTT GAC CTC TCA GAC AAC ACT GAG AAA ATC ATC GCT 149
X76736                  CTT CTT GTC ATT TTC TCT GTA ACC ATG --- AGT CTT CTG ACC GAT CTC GTT AAC CTT GAC CTC TCA GAC AAC ACT GAG AAA ATC ATC GCT 130
Bra039756_cds           --- --- --- --- --- --- --- --- ATG --- AGT CTT CTG ACC GAT CTC GTT AAC CTT GAC CTC TCA GAC AAC ACT GAG AAA ATC ATC GCT 63
BraGLN1.2_C1            CTT CTT GTC ATT TTC TCT GTA ACC ATG --- AGT CTT CTG ACC GAT CTC GTT AAC CTT GAC CTC TCA GAC AAC ACT GAG AAA ATC ATC GCT 145
EU499383                CTT CTT GTC ATT TTC TCT GTA ACC ATG --- AGT CTT CTG ACC GAT CTC GTT AAC CTT GAC CTC TCA GAC AAC ACT GAG AAA ATC ATC GCT 147
AY773089                CTT CTT GTC ATT TTC TCT GTA ACC ATG --- AGT CTT CTG ACC GAT CTC GTT AAC CTT GAC CTC TCA GAC AAC ACT GAG AAA ATC ATC GCT 151
BnaGLN1.2_C2            CTT CTT GTC ATT TTC TCT GTA ACC ATG --- AGT CTT CTG ACC GAT CTC GTT AAC CTT GAC CTC TCA GAC AAC ACT GAG AAA ATC ATC GCT 152
Y12459                  CTT CTT GTC ATT TTC TCT GTA ACC ATG --- AGT CTT CTG ACC GAT CTC GTT AAC CTT GAC CTC TCA GAC AAC ACT GAG AAA ATC ATC GCT 94
BolGLN1.2_C1            --- --- --- --- --- --- --- --- --- --- --- --- --- --- --- --- GAT GTG TTT TGT CTT TTC TTA TTT TGG TTT TTT CTT TTG AAA 42
EU822334                CTT CTT GTC ATT TTC TCT GTA ACC ATG --- AGT CTT CTG ACC GAT CTC GTT AAC CTT GAC CTC TCA GAC AAC ACT GAG AAA ATC ATC GCT 91
EU822335                CTT CTT GTC ATT TTC TCT GTA ACC ATG --- AGT CTT CTG ACC GAT CTC GTT AAC CTT GAC CTC TCA GAC AAC ACT GAG AAA ATC ATC GCT 88
At3g17820_GLN1.3__cds   --- --- --- --- --- --- --- --- ATG --- TCT CTG CTC TCA GAT CTC GTT AAC CTC AAC CTC ACC GAT GCC ACC GGG AAA ATC ATC GCC 63
BnaGLN1.3_C2            TCT CTT TTC TCT CGT GAC GGC GCC ATG --- TCT CTC CTC TCA GAT CTC GTC AAC CTC AAC CTC TCT GAC TCC ACC GAG AAA ATC ATC GCC 210
JX306693                --- --- --- TCT CGT GAC GGC GCC ATG --- TCT CTC CTC TCA GAT CTC GTC AAC CTC AAC CTC TCT GAC TCC ACC GAG AAA ATC ATC GCC 78
Bra022247_cds           --- --- --- --- --- --- --- --- ATG --- TCT CTC CTC TCA GAT CTC GTC AAC CTC AAC CTC TCT GAC TCC ACC GAG AAA ATC ATC GCT 63
BraGLN1.3_C1            TCT CTT TTC TCT CGT GAC GGC GCC ATG --- TCT CTC CTC TCA GAT CTC GTC AAC CTC AAC CTC TCT GAC TCC ACC GAG AAA ATC ATC GCT 133
BnaGLN1.3_C1            TTA TCT TTC TCT CGT GAC GGC GCC ATG --- TCT CTC CTC TCA GAT CTC GTC AAC CTC AAC CTC TCT GAC TCC ACC GAG AAA ATC ATA GCC 130
JX306690                TTA TCT TTC TCT CGT GAC GGC GCC ATG --- TCT CTC CTC TCA GAT CTC GTC AAC CTC AAC CTC TCT GAC TCC ACC GAG AAA ATC ATA GCC 97
BolGLN1.3_C1            TTA TCT TTC TCT CGT GAC GGC GCC ATG --- TCT CTC CTC TCA GAT CTC GTC AAC CTC AAC CTC TCT GAC TCC ACC GAG AAA ATC ATA GCC 105
BnaGLN1.3_C4            CGT ATT CTC CTC TCA GAC GCA GCC ATG --- TCT CTG CTC TCA GAT CTC GTC AAC CTC AAC CTC TCC GAC ACA ACC AAG CAA ATC ATC GCC 132
Bra021276_cds           --- --- --- --- --- --- --- --- ATG --- TCT CTG CTC TCA GAT CTC GTC AAC CTC AAC CTG TCC GAC ACA ACC AAG CAA ATC ATC GCC 63
BraGLN1.3_C3            --- --- --- --- --- --- --- --- --- --- --- --- --- --- --- --- --- --- --- --- --- --- --- --- -CC AAG CAA ATC ATC GCC 17
BnaGLN1.3_C3            CGT ATT CTC CTC TCA GAC GCA GCC ATG --- TCT CTG CTC TCA GAT CTC GTC AAC CTC AAC CTC TCC GAC ACC ACC AAG CAA ATC ATC GCC 90
BnaGLN1.3_C5            GAT TGA TTA CTA TCC GAC GCA GCC ATG --- TCT CTG CTC TCA GAT CTC GTC AAC CTC AAC CTC TCC GAC TCC ACC AAG CAA ATC ATC GCC 144
JX306694                GAT TGA TTA CTA TCC GAC GCA GCC ATG --- TCT CTG CTC TCA GAT CTC GTC AAC CTC AAC CTC TCC GAC TCC ACC AAG CAA ATC ATC GCC 119
Bra001686_cds           --- --- --- --- --- --- --- --- --- --- --- --- --- --- --- --- --- --- --- --- --- --- --- --- --- --- --- --- --- --- -
BraGLN1.3_C2            --- --- --- --- --- --- --- --- --- --- --- --- --- --- --- --- --- --- --- --- --- --- --- --- --- --- --- --- --- --- 236
BnaGLN1.3_C6            GAT TGA TTA CTA TCC GAC GCA GCC ATG --- TCT CTG CTC TCA GAT CTC GTC AAC CTC AAC CTC TCC GAC TCC ACC AAG CAA ATC ATC GCC 117
BolGLN1.3_C2            --- --- --- --- --- --- --- --- --- --- --- --- --- --- --- --- --- --- --- --- --- --- --- --- --- --- --- ATC ATC GCC 9
At5g16570_GLN1.4__cds   --- --- --- --- --- --- --- --- ATG --- TCT TCA CTT GCA GAT TTA ATC AAT CTC GAT CTC TCC GAT TCC ACT GAC CAG ATC ATC GCC 63
BnaGLN1.4_C1            TTT TCT GTT CAG ATT CCT AGA AAA ATG --- TCG GCT CTT GCA GAT TTA ATC AAT CTC GAT CTC TCC GAC TCC TCT GAG AAG ATC ATT GCC 105
JX306697                --- --- -TT CAG ATT CCT AGA AAA ATG --- TCG GCT CTT GCA GAT TTA ATC AAT CTC GAT CTC TCC GAC TCC TCT GAG AAG ATC ATT GCC 80
JX306692                --- --- --- --- --- --- --- --- --- --- --- --- --- --- --- --- --- --- --- --- --- --- --- --- --- --- --- --- --- --- -
Bra023573_cds           --- --- --- --- --- --- --- --- ATG --- TCG GCT CTT GCA GAT TTA ATC AAT CTC GAT CTC TCC GAC TCC TCT GAG AAG ATC ATT GCC 63
BraGLN1.4_C1            TTT TCT GTT CAG ATT CCT AGA AAA ATG --- TCG GCT CTT GCA GAT TTA ATC AAT CTC GAT CTC TCC GAC TCC TCT GAG AAG ATC ATT GCC 185
BnaGLN1.4_C2            TAT TCT CTT CAG AGT CCT AGA AAA ATG --- TCG GCT CTT GCA GAT TTA ATC AAT CTC GAT CTC TCC GAC TCC TCT GAG AAG ATC ATT GCC 130
JX306695                TAT TCT CTT CAG AGT CCT AGA AAA ATG --- TCG GCT CTT GCA GAT TTA ATC AAT CTC GAT CTC TCC GAC TCC TCT GAG AAG ATC ATT GCC 104
JX306696                --- --- -TT CAG ATT CCT AGA AAA ATG --- TCG GCT CTT GCA GAT TTA ATC AAT CTC GAT CTC TCC GAC TCC TCT GAG AAG ATC ATT GCC 80
BolGLN1.4_C1            TAT TCT CTT CAG AGT CCT AGA AAA ATG --- TCG GCT CTT GCA GAT TTA ATC AAT CTC GAT CTC TCC GAC TCC TCT GAG AAG ATC ATT GCC 105
BnaGLN1.4_C4            --- --- --- --- --- --- --- AAA ATG --- TCG GCA CTT GCA GAT TTG ATC AAT CTC GAT CTC TCC GAT TCC ACT GAG AAG ATC ATT GCG 66
JX306700                --- --- --- --- --- --- --- --- --- --- --- --- --- --- --- --- --- --- --- --- --- --- --- --- --- --- --- --- --- --- -
JX306701                --- --- --- --- --- --- --- --- --- --- --- --- --- --- --- --- --- --- --- --- --- --- --- --- --- --- --- --- --- --- -
```

```
Bra008612_cds         --- --- --- --- --- --- --- --- ATG --- TCG GCA CTT GCA GAT TTG ATC AAT CTC GAT CTC TCC GAT TCC ACT GAG AAG ATC ATT GCG 63
BraGLN1.4_C2          TTT TTT TTT AGA TTC CTA GAG AAA ATG --- TCG GCA CTT GCA GAT TTG ATC AAT CTC GAT CTC TCC GAT TCC ACT GAG AAG ATC ATT GCG 108
BnaGLN1.4_C3          TTT TTT TTT AGA TTC CTA GAG AAA ATG --- TCG GCA CTT GCA GAT TTG ATC AAT CTC GAT CTC TCC GAT TAC ACT GAG AAG ATC ATT GCG 107
JX306698              --- --- --- --- --- --- --- --- --- --- --- --- --- --- --- --- --- --- --- --- --- --- --- --- --- --- --- --- --- --- -
JX306699              --- --- --- --- --- --- --- --- --- --- --- --- --- --- --- --- --- --- --- --- --- --- --- --- --- --- --- --- --- --- -
BolGln1.4_C2          --- --- --- --- --- --- --G AAA ATG --- TCG GCA CTT GCA GAT TTG ATC AAT CTC GAT CTC TCC GAT TAC ACT GAG AAG ATC ATT GCG 67
At1g48470_GLN1.5__cds --- --- --- --- --- --- --- --- ATG ACG TCT CCT CTC TCA GAT CTC CTA AAC CTT GAT CTA TCA GAC --- ACC AAG AAA ATC ATC GCT 63
BnaGLN1.5_C2          TTC GGC TTG ATG GAA ACA GAA AAA ATG --- TCT CCA CTC TCC GAT CTC CTA AAC CTC AAT CTC --- GAC --- ACC AAG CAA ATC ATC GCT 185
Bra018729_cds         --- --- --- --- --- --- --- --- ATG --- TCT CCA CTC TCC GAT CTC CTA AAC CTC AAT CTC --- GAC --- ACC AAG CAA ATC ATC GCT 57
BraGLN1.5_C1          TTC GGC TTG ATG GAA ACA GAA AAA ATG --- TCT CCA CTC CCC TAT CTC CTA AAC CTC AAT CTC --- GAC --- ACC AAG CAA ATC ATC GCT 98
BnaGLN1.5_C1          TTC GGC TTG ATG GAA ACA GAA AGA ATG --- TCT CCA CTC TCA GAT CTC CTG AAC CTC AAC CTC --- GAC --- ACC AAG CAA ATC ATC GCT 160
JX306691              --- --- --- --- --- --- --- --- --- --- --- --- --- --- --- --- --- --- --- --- --- --- --- --- --- --- --- --- --- --- -
BolGLN1.5_C1          TTC GGC TTG ATG GAA ACA GAA AGA ATG --- TCT CCA CTC TCA GAT CTC CTG AAC CTC AAT CTC --- GAC --- ACC AAG CAA ATC ATC GCT 144
```

BnaGLN1_JXB

```
                                                       460                 480                 500                     520                     540
                                                        |                   |                   |                       |                       |
At5g37600_GLN1.1__cds  GAA TAC ATA TGG GTT GGT GGT TCT GGA ATG GAC ATG AGA AGC AAA GCC AGG --- --- --- --- --- --- --- --- --- --- --- --- --- 114
BnaGLN1.1_C1           GAA TAC ATA TGG GTT GGA GGT TCA GGA ATG GAT ATG AGA AGC AAA GCC AGG --- --- --- --- --- --- --- --- --- --- --- --- --- 202
X82997                 GAA TAC ATA TGG GTT GGA GGT TCA GGA ATG GAT ATG AGA AGC AAA GCC AGG --- --- --- --- --- --- --- --- --- --- --- --- --- 175
Bra028132_cds          GAA TAC ATA TGG GTT GGA GGT TCA GGA ATG GAT ATG AGA AGC AAA GCC AGG --- --- --- --- --- --- --- --- --- --- --- --- --- 114
BraGLN1.1_C1           GAA TAC ATA TGG GTT GGA GGT TCA GGA ATG GAT ATG AGA AGC AAA GCC AGG --- --- --- --- --- --- --- --- --- --- --- --- --- 205
BnaGLN1.1_C2           GAA TAC ATA TGG GTT GGA GGT TCA GGA ATG GAT ATG AGA AGC AAA GCC AGG --- --- --- --- --- --- --- --- --- --- --- --- --- 202
Y12460                 GAA TAC ATA TGG GTT GGA GGT TCA GGA ATG GAT ATG AGA AGC AAA GCC AGG --- --- --- --- --- --- --- --- --- --- --- --- --- 93
BolGLN1.1_C1           GAA TAC ATA TGG GTT GGA GGT TCA GGA ATG GAT ATG AGA AGC AAA GCC AGG --- --- --- --- --- --- --- --- --- --- --- --- --- 196
At1g66200.1_GLN1.2__cds GAA TAC ATA TGG GTT GGT GGT TCT GGT ATG GAC ATG AGA AGC AAA GCC AGG --- --- --- --- --- --- --- --- --- --- --- --- --- 114
BnaGLN1.2_C1           GAA TAC ATA TGG GTT GGT GGT TCA GGA ATG GAT ATG AGA AGC AAA GCC AGG --- --- --- --- --- --- --- --- --- --- --- --- --- 200
X76736                 GAA TAC ATA TGG GTT GGT GGT TCA GGA ATG GAT ATG AGA AGC AAA GCC AGG --- --- --- --- --- --- --- --- --- --- --- --- --- 181
Bra039756_cds          GAA TAC ATA TGG GGT GGT GGG TCA GGA ATG GAT ATG AGA AGC AAA GCC AGG --- --- --- --- --- --- --- --- --- --- --- --- --- 114
BraGLN1.2_C1           GAA TAC ATA TGG GTT GGT GGT TCA GGA ATG GAT ATG AGA AGC AAA GCC AGG --- --- --- --- --- --- --- --- --- --- --- --- --- 196
EU499383               GAA TAC ATA TGG GTT GGT GGT TCA GGA ATG GAT ATG AGA AGC AAA GCC AGG --- --- --- --- --- --- --- --- --- --- --- --- --- 198
AY773089               GAA TAC ATA TGG GTT GGT GGT TCA GGA ATG GAT ATG AGA AGC AAA GCC AGG --- --- --- --- --- --- --- --- --- --- --- --- --- 202
BnaGLN1.2_C2           GAA TAC ATA TGG GTT GGT GGT TCA GGA ATG GAT ATG AGA AGC AAA GCC AGG --- --- --- --- --- --- --- --- --- --- --- --- --- 203
Y12459                 GAA TAC ATA TGG GTT GGT GGT TCA GGA ATG GAT ATG AGA AGC AAA GCC AGG --- --- --- --- --- --- --- --- --- --- --- --- --- 145
BolGLN1.2_C1           AAA AAA AAC AGG GTT GGT GGT TCA GGA ATG GAT ATG AGA AGC AAA GCC AGG --- --- --- --- --- --- --- --- --- --- --- --- --- 93
EU822334               GAA TAC ATA TGG GTT GGT GGT TCA GGA ATG GAT ATG AGA AGC AAA GCC AGG --- --- --- --- --- --- --- --- --- --- --- --- --- 142
EU822335               GAA TAC ATA TGG GTT GGT GGT TCA GGA ATG GAT ATG AGA AGC AAA GCC AGG --- --- --- --- --- --- --- --- --- --- --- --- --- 139
At3g17820_GLN1.3__cds  GAA TAC ATA TGG ATC GGT GGA TCT GGA ATG GAT ATC AGA AGC AAA GCC AGG --- --- --- --- --- --- --- --- --- --- --- --- --- 114
BnaGLN1.3_C2           GAA TAC ATA TGG ATC GGT GGA TCT GGA ATG GAC ATC AGA AGC AAA GCC AGG --- --- --- --- --- --- --- --- --- --- --- --- --- 261
JX306693               GAA TAC ATA TGG ATC GGT GGA TCT GGA ATG GAC ATC AGA AGC AAA GCC AGG --- --- --- --- --- --- --- --- --- --- --- --- --- 129
Bra022247_cds          GAA TAC ATA TGG ATT GGT GGA TCT GGG ATG GAC ATT AGA AGC AAA GCC AGA --- --- --- --- --- --- --- --- --- --- --- --- --- 114
BraGLN1.3_C1           GAA TAC ATA TGG ATT GGT GGA TCT GGG ATG GAC ATT AGA AGC AAA GCC AGA --- --- --- --- --- --- --- --- --- --- --- --- --- 184
BnaGLN1.3_C1           GAA TAC ATA TGG ATC GGT GGA TCT GGA ATG GAC ATC AGA AGC AAA GCC AGA --- --- --- --- --- --- --- --- --- --- --- --- --- 181
JX306690               GAA TAC ATA TGG ATC GGT GGA TCT GGA ATG GAC ATC AGA AGC AAA GCC AGA --- --- --- --- --- --- --- --- --- --- --- --- --- 148
BolGLN1.3_C1           GAA TAC ATA TGG ATC GGT GGA TCT GGA ATG GAC ATC AGA AGC AAA GCC AGA --- --- --- --- --- --- --- --- --- --- --- --- --- 156
BnaGLN1.3_C4           GAA TAC ATA TGG ATC GGT GGA TCT GGA ATG GAC ATT AGA AGC AAA GCC AGA --- --- --- --- --- --- --- --- --- --- --- --- --- 183
Bra021276_cds          GAA TAC ATA TGG ATC GGT GGA TCT GGA ATG GAC ATT AGA AGC AAA GCC AGG --- --- --- --- --- --- --- --- --- --- --- --- --- 114
BraGLN1.3_C3           GAA TAC ATA TGG ATC GGT GGA TCT GGA ATG GAC ATT AGA AGC AAA GCC AGG --- --- --- --- --- --- --- --- --- --- --- --- --- 68
BnaGLN1.3_C3           GAA TAC ATA TGG ATC GGT GGA TCT GGA ATG GAC ATT AGA AGC AAA GCC AGG --- --- --- --- --- --- --- --- --- --- --- --- --- 141
BnaGLN1.3_C5           GAA TAC ATA TGG ATC GGT GGA TCT GGC ATG GAC ATT AGA AGC AAA GCC AGA --- --- --- --- --- --- --- --- --- --- --- --- --- 195
JX306694               GAA TAC ATA TGG ATC GGT GGA TCT GGC ATG GAC ATT AGA AGC AAA GCC AGA --- --- --- --- --- --- --- --- --- --- --- --- --- 170
Bra001686_cds          --- --- --- --- --- --- --- --- --- ATG GAC ATT AGA AGC AAA GCT AGA GTA AAT TTG AAA CTC TGT TTT TTT CAT TCC TCT GTT TTT 63
BraGLN1.3_C2           --- --- --- -GG ATC GGT GGA TCT GGC ATG GAC ATT AGA AGC AAA GCT AGA --- --- --- --- --- --- --- --- --- --- --- --- --- 277
BnaGLN1.3_C6           GAA TAC ATA TGG ATC GGT GGA TCT GGC ATG GAC ATT AGA AGC AAA GCC AGA --- --- --- --- --- --- --- --- --- --- --- --- --- 168
BolGLN1.3_C2           GAA TAC ATA TGG ATC GGT GGA TCT GGC ATG GAC ATT AGA AGC AAA GCC AGA --- --- --- --- --- --- --- --- --- --- --- --- --- 60
At5g16570_GLN1.4__cds  GAG TAC ATA TGG ATT GGT GGA TCG GGC TTG GAT ATG AGA AGC AAA GCA AGG --- --- --- --- --- --- --- --- --- --- --- --- --- 114
BnaGLN1.4_C1           GAG TAC ATA TGG ATT GGT GGA TCA GGC TTG GAT ATG AGA AGC AAA GCA AGG --- --- --- --- --- --- --- --- --- --- --- --- --- 156
JX306697               GAG TAC ATA TGG ATT GGT GGA TCA GGC TTG GAT ATG AGA AGC AAA GCA AGG --- --- --- --- --- --- --- --- --- --- --- --- --- 131
JX306692               --- --- --- --- --- --- --- --- --- --- --- --- --- AGC AAA GCA AGG --- --- --- --- --- --- --- --- --- --- --- --- --- 12
Bra023573_cds          GAG TAC ATA TGG ATT GGT GGA TCA GGC TTG GAT ATG AGA AGC AAA GCA AGG --- --- --- --- --- --- --- --- --- --- --- --- --- 114
BraGLN1.4_C1           GAG TAC ATA TGG ATT GGT GGA TCA GGC TTG GAT ATG AGA AGC AAA GCA AGG --- --- --- --- --- --- --- --- --- --- --- --- --- 236
BnaGLN1.4_C2           GAG TAC ATA TGG ATT GGT GGA TCA GGC TTG GAT ATG AGA AGC AAA GCA AGG --- --- --- --- --- --- --- --- --- --- --- --- --- 181
JX306695               GAG TAC ATA TGG ATT GGT GGA TCA GGC TTG GGT ATG AGA AGC AAA GCA AGG --- --- --- --- --- --- --- --- --- --- --- --- --- 155
JX306696               GAG TAC ATA TGG ATT GGT GGA TCA GGC TTG GAT ATG AGA AGC AAA GCA AGG --- --- --- --- --- --- --- --- --- --- --- --- --- 131
BolGLN1.4_C1           GAG TAC ATA TGG ATT GGT GGA TCA GGC TTG GAT ATG AGA AGC AAA GCA AGG --- --- --- --- --- --- --- --- --- --- --- --- --- 156
BnaGLN1.4_C4           GAG TAC ATA TGG ATT GGT GGA TCA GGC TTG GAT ATG AGA AGC AAA GCA AGG --- --- --- --- --- --- --- --- --- --- --- --- --- 117
JX306700               --- --- --- --- --- --- --- --- --- --- --- --- --- --- --- --- --- --- --- --- --- --- --- --- --- --- --- --- --- --- -
JX306701               --- --- --- --- --- --- --- --- --- --- --- --- --- --- --- --- --- --- --- --- --- --- --- --- --- --- --- --- --- --- -
```

```
Bra008612_cds       GAG TAC ATA TGG ATT GGT GGA TCA GGC TTG GAT ATG AGA AGC AAA GCA AGG --- --- --- --- --- --- --- --- --- --- --- --- --- 114
BraGLN1.4_C2        GAG TAC ATA TGG ATT GGT GGA TCA GGC TTG GAT ATG AGA AGC AAA GCA AGG --- --- --- --- --- --- --- --- --- --- --- --- --- 159
BnaGLN1.4_C3        GAG TAC ATA TGG ATT GGT GGA TCA GGC TTG GAT ATG AGA AGC AAA GCA AGG --- --- --- --- --- --- --- --- --- --- --- --- --- 158
JX306698            --- --- --- --- --- --- --- --- --- --- --- --- --- --- --- --- --- --- --- --- --- --- --- --- --- --- --- --- --- --- -
JX306699            --- --- --- --- --- --- --- --- --- --- --- --- --- --- --- --- --- --- --- --- --- --- --- --- --- --- --- --- --- --- -
BolGln1.4_C2        GAG TAC ATA TGG ATT GGT GGA TCA GGC TTG GAT ATG AGA AGC AAA GCA AGG --- --- --- --- --- --- --- --- --- --- --- --- --- 118
At1g48470_GLN1.5__cds GAA TAC ATA TGG ATC GGT GGC TCT GGA ATG GAT ATT AGA AGC AAA GCC AGG --- --- --- --- --- --- --- --- --- --- --- --- --- 114
BnaGLN1.5_C2        GAA TAC ATA TGG ATC GGT GGG TCT GGA ATG GAC ATT AGA AGC AAA GGC AGG --- --- --- --- --- --- --- --- --- --- --- --- --- 236
Bra018729_cds       GAA --- --- --G ATC GGT GGG TCT GGA ATG GAC ATT AGA AGC A-- --- --- --- --- --- --- --- --- --- --- --- --- --- --- --- 92
BraGLN1.5_C1        GAA TAC ATA TGG ATC GGT GGG TCT GGA ATG GAC ATT AGA AGC AAA GGC AGG --- --- --- --- --- --- --- --- --- --- --- --- --- 149
BnaGLN1.5_C1        GAA TAC ATA TGG ATC GGT GGG TCT GGA ATG GAC ATT AGA AGC AAA GGC AGG --- --- --- --- --- --- --- --- --- --- --- --- --- 211
JX306691            --- --- --- --- --- --- --- --- --- --- --- --- --- --- --- --- --- --- --- --- --- --- --- --- --- --- --- --- --- --- -
BolGLN1.5_C1        GAA TAC ATA TGG ATC GGT GGG TCT GGA ATG GAC ATT AGA AGC AAA GGC AGG --- --- --- --- --- --- --- --- --- --- --- --- --- 195
```

BnaGLN1_JXB

```
                                                                                     560                                         580                                         600                                         620
At5g37600_GLN1.1__cds  --- --- --- --- --- --- --- --- --- --- --- --- --- --- --- --- --- ACT CTA CCT GGA CCA GTG ACT GAC CCT TCG CAG CTA CCA 153
BnaGLN1.1_C1           --- --- --- --- --- --- --- --- --- --- --- --- --- --- --- --- --- ACT CTT CCT GGA CCA GTG AGT GAC CCT TCG GAG CTA CCA 241
X82997                 --- --- --- --- --- --- --- --- --- --- --- --- --- --- --- --- --- ACT CTT CCT GGA CCA GTG AGT GAC CCT TCG GAG CTA CCA 214
Bra028132_cds          --- --- --- --- --- --- --- --- --- --- --- --- --- --- --- --- --- ACT CTT CCT GGA CCA GTG AGT GAC CCT TCG GAG CTA CCA 153
BraGLN1.1_C1           --- --- --- --- --- --- --- --- --- --- --- --- --- --- --- --- --- ACT CTT CCT GGA CCA GTG AGT GAC CCT TCG GAG CTA CCA 244
BnaGLN1.1_C2           --- --- --- --- --- --- --- --- --- --- --- --- --- --- --- --- --- ACT CTT CCT GGA CCA GTG AGT GAC CCT TCG GAG CTA CCA 241
Y12460                 --- --- --- --- --- --- --- --- --- --- --- --- --- --- --- --- --- ACT CTT CCT GGA CCA GTG AGT GAC CCT TCG GAG CTA CCA 132
BolGLN1.1_C1           --- --- --- --- --- --- --- --- --- --- --- --- --- --- --- --- --- ACT CTT CCT GGA CCA GTG AGT GAC CCT TCG GAG CTA CCA 235
At1g66200.1_GLN1.2__cds --- --- --- --- --- --- --- --- --- --- --- --- --- --- --- --- --- ACT CTC CCT GGA CCT GTG ACC GAT CCA TCA AAA CTT CCA 153
BnaGLN1.2_C1           --- --- --- --- --- --- --- --- --- --- --- --- --- --- --- --- --- ACT CTC CCT GGA CCT GTG ACC GAT CCA TCA AAG CTC CCA 239
X76736                 --- --- --- --- --- --- --- --- --- --- --- --- --- --- --- --- --- ACT CTC CCT GGA CCT GTG ACC GAT CCA TCA AAG CTC CCA 220
Bra039756_cds          --- --- --- --- --- --- --- --- --- --- --- --- --- --- --- --- --- ACT CTC CCT GGA CCT GTG ACC GAT CCA TCA AAG CTC CCA 153
BraGLN1.2_C1           --- --- --- --- --- --- --- --- --- --- --- --- --- --- --- --- --- ACT CTC CCT GGA CCT GTG ACC GAT CCA TCA AAG CTC CCA 235
EU499383               --- --- --- --- --- --- --- --- --- --- --- --- --- --- --- --- --- ACT CTC CCT GGA CCT GTG ACC GAT CCA TCA AAG CTC CCA 237
AY773089               --- --- --- --- --- --- --- --- --- --- --- --- --- --- --- --- --- ACT CTC CCT GGA CCT GTG ACC GAT CCA TCA AAG CTC CCA 241
BnaGLN1.2_C2           --- --- --- --- --- --- --- --- --- --- --- --- --- --- --- --- --- ACT CTC CCT GGA CCT GTG ACC GAT CCA TCA AAG CTC CCA 242
Y12459                 --- --- --- --- --- --- --- --- --- --- --- --- --- --- --- --- --- ACT CTC CCT GGA CCT GTG ACC GAT CCA TCA AAG CTC CCA 184
BolGLN1.2_C1           --- --- --- --- --- --- --- --- --- --- --- --- --- --- --- --- --- ACT CTC CCT GGA CCT GTG ACC GAT CCA TCA AAG CTC CCA 132
EU822334               --- --- --- --- --- --- --- --- --- --- --- --- --- --- --- --- --- ACT CTC CCT GGA CCT GTG ACC GAT CCA TCA AAG CTC CCA 181
EU822335               --- --- --- --- --- --- --- --- --- --- --- --- --- --- --- --- --- ACT CTC CCT GGA CCT GTG ACC GAT CCA TCA AAG CTC CCA 178
At3g17820_GLN1.3__cds  --- --- --- --- --- --- --- --- --- --- --- --- --- --- --- --- --- ACA CTA CCA GGA CCA GTG ACT GAT CCA TCA AAG CTT CCC 153
BnaGLN1.3_C2           --- --- --- --- --- --- --- --- --- --- --- --- --- --- --- --- --- ACA CTA CCA GGA CCA GTG AGC GAT CCA TCA AAG CTG CCT 300
JX306693               --- --- --- --- --- --- --- --- --- --- --- --- --- --- --- --- --- ACA CTA CCA GGA CCA GTG AGC GAT CCA TCA AAG CTG CCT 168
Bra022247_cds          --- --- --- --- --- --- --- --- --- --- --- --- --- --- --- --- --- ACA CTC CCA GGA CCA GTG AGC GAT CCA TCA AAG CTT CCT 153
BraGLN1.3_C1           --- --- --- --- --- --- --- --- --- --- --- --- --- --- --- --- --- ACA CTC CCA GGA CCA GTG AGC GAT CCA TCA AAG CTT CCT 223
BnaGLN1.3_C1           --- --- --- --- --- --- --- --- --- --- --- --- --- --- --- --- --- ACA CTC CCA GGA CCA GTG AGC GAT CCA TCA AAG CTT CCT 220
JX306690               --- --- --- --- --- --- --- --- --- --- --- --- --- --- --- --- --- ACA CTC CCA GGA CCA GTG AGC GAT CCA TCA AAG CTT CCT 187
BolGLN1.3_C1           --- --- --- --- --- --- --- --- --- --- --- --- --- --- --- --- --- ACA CTC CCA GGA CCA GTG AGC GAT CCA TCA AAG CTT CCT 195
BnaGLN1.3_C4           --- --- --- --- --- --- --- --- --- --- --- --- --- --- --- --- --- ACA CTA CCA GGA CCA GTG ACC GAT CCA TCA AAG CTT CCC 222
Bra021276_cds          --- --- --- --- --- --- --- --- --- --- --- --- --- --- --- --- --- ACA CTC CCA GGA CCA GTG ACC GAT CCA TCA AAG CTT CCC 153
BraGLN1.3_C3           --- --- --- --- --- --- --- --- --- --- --- --- --- --- --- --- --- ACA CTC CCA GGA CCA GTG ACC GAT CCA TCA AAG CTT CCC 107
BnaGLN1.3_C3           --- --- --- --- --- --- --- --- --- --- --- --- --- --- --- --- --- ACA CTC CCA GGA CCA GTG ACC GAT CCA TCA AAG CTT CCC 180
BnaGLN1.3_C5           --- --- --- --- --- --- --- --- --- --- --- --- --- --- --- --- --- ACA CTC CCA GGA CCA GTG AGC GAT CCA TCA AAG CTT CCC 234
JX306694               --- --- --- --- --- --- --- --- --- --- --- --- --- --- --- --- --- ACA CTC CCA GGA CCA GTG AGC GAT CCA TCA AAG CTT CCC 209
Bra001686_cds          CTT CTC TCT GTT TAT TGT ATG ATC GTT CTG ATT TGC TTT TGG ATG ATA AAG ACA CTC CCA GGA CCA GTG AGC GAT CCA TCA AAG CTT CCC 153
BraGLN1.3_C2           --- --- --- --- --- --- --- --- --- --- --- --- --- --- --- --- --- ACA CTC CCA GGA CCA GTG AGC GAT CCA TCA AAG CTT CCC 316
BnaGLN1.3_C6           --- --- --- --- --- --- --- --- --- --- --- --- --- --- --- --- --- ACA CTT CCA GGA CCA GTG AGC GAT CCA TCA AAG CTT CCC 207
BolGLN1.3_C2           --- --- --- --- --- --- --- --- --- --- --- --- --- --- --- --- --- ACA CTC CCA GGA CCA GTG AGC GAT CCA TCA AAG CTT CCC 99
At5g16570_GLN1.4__cds  --- --- --- --- --- --- --- --- --- --- --- --- --- --- --- --- --- ACT TTG CCT GGA CCA GTG ACG GAT CCA TCG CAG TTA CCG 153
BnaGLN1.4_C1           --- --- --- --- --- --- --- --- --- --- --- --- --- --- --- --- --- ACT TTG CCG GGA CCA GTG AAG GAT CCA TCG GAG TTA CCG 195
JX306697               --- --- --- --- --- --- --- --- --- --- --- --- --- --- --- --- --- ACT TTG CCG GGA CCA GTG AAG GAT CCA TCG GAG TTA CCG 170
JX306692               --- --- --- --- --- --- --- --- --- --- --- --- --- --- --- --- --- ACT TTG CCG GGA CCA GTG AAG GAT CCA TCG GAG TTA CCG 51
Bra023573_cds          --- --- --- --- --- --- --- --- --- --- --- --- --- --- --- --- --- ACT TTG CCG GGA CCA GTG AAG GAT CCA TCG GAG TTA CCG 153
BraGLN1.4_C1           --- --- --- --- --- --- --- --- --- --- --- --- --- --- --- --- --- ACT TTG CCG GGA CCA GTG AAG GAT CCA TCG GAG TTA CCG 275
BnaGLN1.4_C2           --- --- --- --- --- --- --- --- --- --- --- --- --- --- --- --- --- ACT TTG CCG GGA CCT GTG AAG GAT CCA TCG GAG TTA CCG 220
JX306695               --- --- --- --- --- --- --- --- --- --- --- --- --- --- --- --- --- ACT TTG CCG GGA CCT GTG AAG GAT CCA TCG GAG TTA CCG 194
JX306696               --- --- --- --- --- --- --- --- --- --- --- --- --- --- --- --- --- ACT TTG CCG GGA CCA GTG AAG GAT CCA TCG GAG TTA CCG 170
BolGLN1.4_C1           --- --- --- --- --- --- --- --- --- --- --- --- --- --- --- --- --- ACT TTG CCG GGA CCT GTG AAG GAT CCA TCG GAG TTA CCG 195
BnaGLN1.4_C4           --- --- --- --- --- --- --- --- --- --- --- --- --- --- --- --- --- ACT TTG CCC GGA CCA GTG AAG GAT CCA TCG GAG TTA CCG 156
JX306700               --- --- --- --- --- --- --- --- --- --- --- --- --- --- --- --- --- --- --- --- --- --- --- --- --- --- --- --- --- --- -
JX306701               --- --- --- --- --- --- --- --- --- --- --- --- --- --- --- --- --- --- --- --- --- --- --- --- --- --- --- --- --- --- -
```

```
Bra008612_cds        --- --- --- --- --- --- --- --- --- --- --- --- --- --- --- --- --- ACT TTG CCG GGA CCA GTG AAG GAT CCA TCG GAG TTA CCG 153
BraGLN1.4_C2         --- --- --- --- --- --- --- --- --- --- --- --- --- --- --- --- --- ACT TTG CCG GGA CCA GTG AAG GAT CCA TCG GAG TTA CCG 198
BnaGLN1.4_C3         --- --- --- --- --- --- --- --- --- --- --- --- --- --- --- --- --- ACT TTG CCC GGA CCA GTG AAG GAT CCA TCG GAG TTA CCG 197
JX306698             --- --- --- --- --- --- --- --- --- --- --- --- --- --- --- --- --- --- --- --- --- --- --- --- --- --- --- --- --- --- -
JX306699             --- --- --- --- --- --- --- --- --- --- --- --- --- --- --- --- --- --- --- --- --- --- --- --- --- --- --- --- --- --- -
BolGln1.4_C2         --- --- --- --- --- --- --- --- --- --- --- --- --- --- --- --- --- ACT TTG CCC GGA CCA GTG AAG GAT CCA TCG GAG TTA CCG 157
At1g48470_GLN1.5__cds --- --- --- --- --- --- --- --- --- --- --- --- --- --- --- --- --- ACA TTA CCA GGA CCA GTA AGT AAT CCA ACA AAG CTT CCA 153
BnaGLN1.5_C2         --- --- --- --- --- --- --- --- --- --- --- --- --- --- --- --- --- ACA TTA CCA GGA CCA GTA AGT GAT CCA TCA AAG CTT CCG 275
Bra018729_cds        --- --- --- --- --- --- --- --- --- --- --- --- --- --- --- --- --- ACA TTA CCA GGA CCA GTA AGT GAT CCA TCA AAG CTT CCG 131
BraGLN1.5_C1         --- --- --- --- --- --- --- --- --- --- --- --- --- --- --- --- --- ACA TTA CCA GGA CCA GTA AGT GAT CCA TCA AAG CTT CCG 188
BnaGLN1.5_C1         --- --- --- --- --- --- --- --- --- --- --- --- --- --- --- --- --- ACA TTA CCA GGA CCT GTA AGT GAT CCA TCA AAG CTT CCG 250
JX306691             --- --- --- --- --- --- --- --- --- --- --- --- --- --- --- --- --- --- --- --- --- --- --- --- --- --- --- --- --- --- -
BolGLN1.5_C1         --- --- --- --- --- --- --- --- --- --- --- --- --- --- --- --- --- ACA TTA CCA GGA CCA GTA AGT GAT CCA TCA AAG CTT CCG 234
```

BnaGLN1_JXB

	640 · 660 · 680 · 700 · 720	
At5g37600_GLN1.1__cds	AAG TGG AAC TAT GAT GGT TCA AGC ACA GGC CAA GCT CCT GGT GAA GAC AGT GAA GTC ATC TTA TAC CCT CAA GCC ATA TTC AAG GAT CCT	243
BnaGLN1.1_C1	AAG TGG AAC TAT GAT GGC TCA AGC ACA GGC CAA GCT CCT GGT GAA GAC AGT GAA GTC ATC TTA TAC CCT CAA GCC ATC TTC AAA GAT CCT	331
X82997	AAG TGG AAC TAT GAT GGC TCA AGC ACA GGC CAA GCT CCT GGT GAA GAC AGT GAA GTC ATC TTA TAC CCT CAA GCC ATC TTC AAA GAT CCT	304
Bra028132_cds	AAG TGG AAC TAT GAT GGC TCA AGC ACA GGC CAA GCT CCT GGT GAA GAC AGT GAA GTC ATC TTA TAC CCT CAA GCC ATC TTC AAA GAT CCT	243
BraGLN1.1_C1	AAG TGG AAC TAT GAT GGC TCA AGC ACA GGC CAA GCT CCT GGT GAA GAC AGT GAA GTC ATC TTA TAC CCT CAA GCC ATC TTC AAA GAT CCT	334
BnaGLN1.1_C2	AAG TGG AAC TAT GAT GGC TCA AGC ACA GGC CAA GCT CCT GGT GAA GAC AGT GAA GTC ATC TTA TAC CCT CAA GCC ATA TTC AAA GAT CCT	331
Y12460	AAG TGG AAC TAT GAT GGC TCA AGC ACA GGC CAA GCT CCT GGT GAA GAC AGT GAA GTC ATC TTA TAC CCT CAA GCC ATA TTC AAA GAT CCT	222
BolGLN1.1_C1	AAG TGG AAC TAT GAT GGC TCA AGC ACA GGC CAA GCT CCT GGT GAA GAC AGT GAA GTC ATC TTA TAC CCT CAA GCC ATA TTC AAA GAT CCT	325
At1g66200.1_GLN1.2__cds	AAG TGG AAC TAT GAT GGT TCA AGC ACT GGT CAA GCT CCT GGT CAA GAC AGT GAA GTG ATC TTA TAC CCT CAA GCA ATT TTC AAA GAT CCA	243
BnaGLN1.2_C1	AAA TGG AAT TAT GAT GGT TCA AGC ACT GGC CAA GCT CCT GGT GAA GAC AGT GAA GTG ATC TTA TAC CCT CAA GCG ATT TTC AAA GAT CCG	329
X76736	AAA TGG AAT TAT GAT GGT TCA AGC ACT GGC CAA GCT CCT GGT GAA GAC AGC GAA GTG ATC TTA TAC CCT CAA GCG ATT TTC AAA GAT CCG	310
Bra039756_cds	AAA TGG AAT TAT GAT GGT TCA AGC ACT GGC CAA GCT CCT GGT GAA GAC AGT GAA GTG ATC TTA TAC CCT CAA GCG ATT TTC AAA GAT CCG	243
BraGLN1.2_C1	AAA TGG AAT TAT GAT GGT TCA AGC ACT GGC CAA GCT CCT GGT GAA GAC AGT GAA GTG ATC TTA TAC CCT CAA GCG ATT TTC AAA GAT CCG	325
EU499383	AAA TGG AAT TAT GAT GGT TCA AGC ACT GGT CAA GCT CCT GGT GAA GAC AGT GAA GTG ATC TTA TAC CCT CAA GCG ATT TTC AAA GAT CCG	327
AY773089	AAA TGG AAT TAT GAT GGT TCA AGC ACT GGC CAA GCT CCT GGT GAA GAC AGT GAA GTG ATC TTA TAC CCT CAA GCG ATT TTC AAA GAT CCG	331
BnaGLN1.2_C2	AAA TGG AAT TAT GAT GGT TCA AGC ACT GGC CAA GCT CCT GGT GAA GAC AGT GAA GTG ATC TTA TAC CCT CAA GCG ATT TTC AAA GAT CCG	332
Y12459	AAA TGG AAT TAT GAT GGT TCA AGC ACT GGC CAA GCT CCT GGT GAA GAC AGT GAA GTG ATC TTA TAC CCT CAA GCG ATT TTC AAA GAT CCG	274
BolGLN1.2_C1	AAA TGG AAT TAT GAT GGT TCA AGC ACT GGC CAA GCT CCT GGT GAA GAC AGT GAA GTG ATC TTA TAC CCT CAA GCG ATT TTC AAA GAT CCG	222
EU822334	AAA TGG AAT TAT GAT GGT TCA AGC ACT GGC CAA GCT CCT GGT GAA GAC AGT GAA GTG ATC TTA TAC CCT CAA GCG ATT TTC AAA GAT CCG	271
EU822335	AAA TGG AAT TAT GAT GGT TCA AGC ACT GGC CAA GCT CCT GGT GAA GAC AGT GAA GTG ATC TTA TAC CCT CAA GCG ATT TTC AAA GAT CCG	268
At3g17820_GLN1.3__cds	AAG TGG AAC TAC GAC GGA TCC AGC ACC GGT CAG GCT GCT GGA GAA GAC AGT GAA GTC ATT CTA TAC CCT CAG GCA ATA TTC AAG GAT CCC	243
BnaGLN1.3_C2	AAG TGG AAC TAC GAC GGA TCC AGC ACC GGT CAA GCC GCC GGA GAT GAC AGT GAA GTC ATT CTA TAC CCT CAG GCG ATA TTC CGT GAT CCG	390
JX306693	AAG TGG AAC TAC GAC GGA TCC AGC ACC GGT CAA GCC GCC GGA GAT GAC AGT GAA GTC ATT CTA TAC CCT CAG GCG ATA TTC CGT GAT CCG	258
Bra022247_cds	AAG TGG AAC TAC GAC GGA TCC AGC ACT GGC CAG GCC GCC GGA GAT GAC AGT GAA GTC ATT CTA TAC CCT CAG GCG ATA TTC CGT GAT CCG	243
BraGLN1.3_C1	AAG TGG AAC TAC GAC GGA TCC AGC ACT GGC CAG GCC GCC GGA GAT GAC AGT GAA GTC ATT CTA TAC CCT CAG GCG ATA TTC CGT GAT CCG	313
BnaGLN1.3_C1	AAG TGG AAC TAC GAC GGA TCC AGC ACC GGT CAA GCC GCC GGA GAT GAC AGT GAA GTC ATT CTA TAC CCT CAG GCG ATA TTC CGT GAT CCG	310
JX306690	AAG TGG AAC TAC GAC GGA TCC AGC ACC GGT CAA GCC GCC GGA GAT GAC AGT GAA GTC ATT CTA TAC CCT CAG GCG ATA TTC CGT GAT CCG	277
BolGLN1.3_C1	AAG TGG AAC TAC GAC GGA TCC AGC ACC GGT CAA GCC GCC GGA GAT GAC AGT GAA GTC ATT CTA TAC CCT CAG GCG ATA TTC CGT GAT CCG	285
BnaGLN1.3_C4	AAG TGG AAC TAC GAC GGA TCT AGC ACC GGT CAG GCC GCT GGA GAT GAC AGT GAA GTC ATT CTA TAT CCT CAG GCT ATA TTC CGT GAT CCG	312
Bra021276_cds	AAG TGG AAC TAC GAC GGA TCA AGC ACC GGT CAG GCC GCC GGA GAT GAC AGT GAA GTC ATT CTA TAC CCT CAG GCT ATA TTC CGT GAT CCG	243
BraGLN1.3_C3	AAG TGG AAC TAC GAC GGA TCA AGC ACC GGT CAG GCC GCC GGA GAT GAC AGT GAA GTC ATT CTA TAC CCT CAG GCT ATA TTC CGT GAT CCG	197
BnaGLN1.3_C3	AAG TGG AAC TAC GAC GGA TCA AGC ACC GGT CAG GCC GCT GGA GAT GAC AGT GAA GTC ATT CTA TAT CCT CAG GCT ATA TTC CGT GAT CCG	270
BnaGLN1.3_C5	AAG TGG AAC TAC GAC GGA TCC AGC ACC GGT CAG GCC TCT GGA GAT AAC AGT GAA GTC ATT CTA TAC CCT CAG GCC ATA TTC CGT GAT CCG	324
JX306694	AAG TGG AAC TAC GAC GGA TCC AGC ACC GGT CAG GCC TCT GGA GAT AAC AGT GAA GTC ATT CTA TAC CCT CAG GCC ATA TTC CGT GAT CCG	299
Bra001686_cds	AAG TGG AAC TAC GAC GGA TCC AGC ACC GGT CAG GCC TCT GGG GAT AAC AGT GAA GTC ATT CTA TAC CCT CAG GCC ATA TTC CGT GAT CCG	243
BraGLN1.3_C2	AAG TGG AAC TAC GAC GGA TCC AGC ACC GGT CAG GCC TCT GGG GAT AAC AGT GAA GTC ATT CTA TAC CCT CAG GCC ATA TTC CGT GAT CCG	406
BnaGLN1.3_C6	AAG TGG AAC TAC GAC GGA TCC AGC ACC GGT CAG GCC TCT GGA GAC AAC AGT GAA GTC ATT CTA TAC CCT CAG GCG ATA TTC CGT GAT CCG	297
BolGLN1.3_C2	AAG TGG AAC TAC GAC GGA TCC AGC ACC GGT CAA GCC TCC GGA GAT AAC AGT GAA GTC ATT CTT TAC CCT CAG GCG ATA TTC CGT GAT CCG	189
At5g16570_GLN1.4__cds	AAA TGG AAC TAC GAC GGT TCA AGC ACC GGC CAA GCT CCG GGC GAT GAC AGT GAA GTC ATC ATC TAC CCT CAA GCT ATC TTC AAA GAC CCC	243
BnaGLN1.4_C1	AAA TGG AAC TAT GAC GGT TCA AGC ACC GGC CAA GCC CCC GGC GAT GAC AGT GAA GTC ATC ATC TAC CCT CAA GCT ATC TTC AAA GAT CCA	285
JX306697	AAA TGG AAC TAT GAC GGT TCA AGC ACC GGC CAA GCC CCC GGC GAT GAC AGT GAA GTC ATC ATC TAC CCT CAA GCT ATC TTC AAA GAT CCA	260
JX306692	AAA TGG AAC TAT GAC GGT TCA AGC ACC GGC CAA GCC CCC GGC GAT GAC AGT GAA GTC ATC ATC TAC CCT CAA GCT ATC TTC AAA GAT CCA	141
Bra023573_cds	AAA TGG AAC TAT GAC GGT TCA AGC ACC GGC CAA GCC CCC GGC GAT GAC AGT GAA GTC ATC ATC - - - CCT CAA GCT ATC TTC AAA GAT CCA	240
BraGLN1.4_C1	AAA TGG AAC TAT GAC GGT TCA AGC ACC GGC CAA GCC CCC GGC GAT GAC AGT GAA GTC ATC ATC TAC CCT CAA GCT ATC TTC AAA GAT CCA	365
BnaGLN1.4_C2	AAA TGG AAC TAT GAC GGT TCA AGC ACC GGG CAA GCT CCC GGC GAT GAT AGT GAA GTC ATC ATC TAC CCT CAA GCT ATC TTC AAA GAT CCA	310
JX306695	AAA TGG AAC TAT GAC GGT TCA AGC ACC GGG CAA GCT CCC GGC GAT GAT AGT GAA GTC ATC ATC TAC CCT CAA GCT ATC TTC AAA GAT CCA	284
JX306696	AAA TGG AAC TAT GAC GGT TCA AGC ACC GGC CAA GCC CCC GGC GAT GAC AGT GAA GTC ATC ATC TAC CCT CAA GCT ATC TTC AAA GAT CCA	260
BolGLN1.4_C1	AAA TGG AAC TAT GAC GGT TCA AGC ACC GGG CAA GCT CCC GGC GAT GAC AGT GAA GTC ATC ATC TAC CCT CAA GCT ATC TTC AAA GAT CCA	285
BnaGLN1.4_C4	AAA TGG AAC TAT GAC GGT TCA AGC ACC GGC CAA GCT CCC GGC AGC GAC AGT GAA GTC ATC CTC TAC CCT CAA GCT ATC TTC AAA GAC CCC	246
JX306700	- -	-
JX306701	- -	-

```
   Bra008612_cds AAA TGG AAC TAT GAC GGT TCA AGC ACC GGC CAA GCT CCC GGC AGT GAC AGT GAA GTC ATC CTC TAC CCT CAA GCT ATC TTC AAA GAC CCC 243
    BraGLN1.4_C2 AAA TGG AAC TAT GAC GGT TCA AGC ACC GGC CAA GCT CCC GGC AGT GAC AGT GAA GTC ATC CTC TAC CCT CAA GCT ATC TTC AAA GAC CCC 288
    BnaGLN1.4_C3 AAA TGG AAC TAT GAC GGT TCA AGC ACC GGC CAA GCT CCC GGC AGT GAC AGT GAA GTC ATC CTC TAC CCT CAA GCT ATC TTC AAA GAC CCC 287
        JX306698 --- --- --- --- --- --- --- --- --- --- --- --- --- --- --- --- --- --- --- --- --- --- --- --- --- --- --- --- --- --- -
        JX306699 --- --- --- --- --- --- --- --- --- --- --- --- --- --- --- --- --- --- --- --- --- --- --- --- --- --- --- --- --- --- -
    BolGln1.4_C2 AAA TGG AAC TAT GAC GGT TCA AGC ACC GGC CAA GCT CCC GGC AGT GAC AGT GAA GTC ATC CTC TAC CCT CAA GCT ATC TTC AAA GAC CCC 247
At1g48470_GLN1.5__cds AAA TGG AAC TAC GAT GGA TCT AGC ACC GAT CAA GCT GCC GGA GAT GAT AGT GAA GTC ATT CTT TAT CCT CAG GCA ATA TTT AAG GAC CCA 243
    BnaGLN1.5_C2 AAA TGG AAC TAC GAT GGA TCC AGC ACC AAT CAA GCC GCC GGA GAT GAC AGT GAA GTC ATT CTA TAT CCT CAG GCG ATT TTT AAA GAC CCA 365
   Bra018729_cds AAA TGG AAC TAC GAT GGA TCC AGC ACC AAT CAA GCC GCC GGA GAT GAC AGT GAA GTC AT- --- --- CCT CAA GCG ATT TTT AAA GAC CCA 214
    BraGLN1.5_C1 AAA TGG AAC TAC GAT GGA TCC AGC ACC AAT CAA GCC GCC GGA GAT GAC AGT GAA GTC ATT CTA TAT CCT CAA GCG ATT TTT AAA GAC CCA 278
    BnaGLN1.5_C1 AAA TGG AAC TAC GAT GGA TCC AGC ACC AAT CAA GCC GCC GGA GAT GAC AGT GAA GTC ATT CTA TAT CCT CAG GCG ATT TTT AAA GAC CCG 340
        JX306691 --- --- --- --- --- --- --- --- --- --- --- --- --- --- --- --- --- --- --- --- --- --- --- --- --- --- TTT AAA GAC CCG 12
    BolGLN1.5_C1 AAA TGG AAC TAC GAT GGA TCC AGC ACC AAT CAA GCC GCC GGA GAT GAC AGT GAA GTC ATT CTA TAT CCT CAG GCG ATT TTT AAA GAC CCG 324
```

BnaGLN1_JXB

						740							760						780							800				
At5g37600_GLN1.1__cds	TTC	CGT	AGA	GGA	AAC	AAC	ATT	CTT	GTC	ATG	TGC	GAT	GCG	TAC	ACT	CCC	GCG	GGT	GAA	CCA	ATC	CCG	ACT	AAC	AAA	AGA	CAC	GCT	GCG	GCT	333
BnaGLN1.1_C1	TTC	CGT	AGA	GGC	AAT	AAC	ATT	CTT	GTC	ATG	TGC	GAT	GCT	TAC	ACT	CCA	GCG	GGC	GAA	CCA	ATC	CCA	ACA	AAC	AAA	AGA	CAC	GCT	GCG	GCT	421
X82997	TTC	CGT	AGA	GGC	AAT	AAC	ATT	CTT	GTC	ATG	TGC	GAT	GCT	TAC	ACT	CCA	GCG	GGC	GAA	CCA	ATC	CCA	ACA	AAC	AAA	AGA	CAC	GCT	GCG	GCT	394
Bra028132_cds	TTC	CGT	AGA	GGC	AAT	AAC	ATT	CTT	GTC	ATG	TGC	GAT	GCT	TAC	ACT	CCA	GCG	GGC	GAA	CCA	ATC	CCA	ACA	AAC	AAA	AGA	CAC	GCT	GCG	GCT	333
BraGLN1.1_C1	TTC	CGT	AGA	GGC	AAT	AAC	ATT	CTT	GTC	ATG	TGC	GAT	GCT	TAC	ACT	CCA	GCG	GGC	GAA	CCA	ATC	CCA	ACA	AAC	AAA	AGA	CAC	GCT	GCG	GCT	424
BnaGLN1.1_C2	TTC	CGT	AGA	GGC	AAC	AAC	ATT	CTT	GTC	ATG	TGC	GAT	GCT	TAC	ACT	CCA	GCG	GGC	GAA	CCA	ATC	CCA	ACA	AAC	AAA	AGA	CAC	GCT	GCG	GCT	421
Y12460	TTC	CGT	GGA	GGC	AAT	AAC	ATT	CTT	GTC	ATG	TGC	GAT	GCT	TAC	ACT	CCA	GCG	GGC	GAA	CCA	ATC	CCA	ACA	AAC	AAA	AGA	CAC	GCT	GCG	GCT	312
BolGLN1.1_C1	TTC	CGT	GGA	GGC	AAT	AAC	ATT	CTT	GTC	ATG	TGC	GAT	GCT	TAC	ACT	CCA	GCG	GGC	GAA	CCA	ATC	CCA	ACA	AAC	AAA	AGA	CAC	GCT	GCG	GCT	415
At1g66200.1_GLN1.2__cds	TTC	CGT	AGA	GGC	AAC	AAC	ATC	CTT	GTT	ATG	TGT	GAT	GCT	TAC	ACT	CCA	GCG	GGA	GAG	CCA	ATC	CCT	ACT	AAC	AAG	CGA	CAT	GCT	GCG	GCT	333
BnaGLN1.2_C1	TTC	CGT	AGA	GGC	AAC	AAC	ATT	CTT	GTC	ATG	TGT	GAT	ACT	TAC	ACC	CCT	GCG	GGT	GAA	CCA	ATC	CCT	ACG	AAC	AAG	AGA	CAT	GCT	GCA	GCT	419
X76736	TTC	CGT	AGA	GGC	AAC	AAC	ATT	CTT	GTC	ATG	TGT	GAT	ACT	TAC	ACC	CCT	GCG	GGT	GAA	CCA	ATC	CCT	ACG	AAC	AAG	AGA	CAT	GCT	GCA	GCT	400
Bra039756_cds	TTC	CGT	AGA	GGC	AAC	AAC	ATT	CTT	GTC	ATG	TGT	GAT	ACT	TAC	ACC	CCT	GCG	GGT	GAA	CCA	ATC	CCT	ACG	AAC	AAG	AGA	CAT	GCT	GCA	GCT	333
BraGLN1.2_C1	TTC	CGT	AGA	GGC	AAC	AAC	ATT	CTT	GTC	ATG	TGT	GAT	ACT	TAC	ACC	CCT	GCG	GGT	GAA	CCA	ATC	CCT	ACG	AAC	AAG	AGA	CAT	GCT	GCA	GCT	415
EU499383	TTC	CGT	AGA	GGC	AAC	AAC	ATT	CTT	GTC	ATG	TGT	GAT	ACT	TAC	ACC	CCT	GCG	GGT	GAA	CCA	ATC	CCT	ACG	AAC	AAG	AGA	CAT	GCT	GCA	GCT	417
AY773089	TTC	CGT	AGA	GGC	AAC	AAC	ATT	CTT	GTC	ATG	TGT	GAT	ACT	TAC	ACC	CCT	GCG	GGT	GAA	CCA	ATC	CCT	ACG	AAC	AAG	AGA	CAT	GCT	GCA	GCT	421
BnaGLN1.2_C2	TTC	CGT	AGA	GGC	AAC	AAC	ATT	CTT	GTC	ATG	TGT	GAT	ACT	TAC	ACC	CCT	GCG	GGT	GAA	CCA	ATC	CCT	ACG	AAC	AAG	AGA	CAT	GCT	GCA	GCT	422
Y12459	TTC	CGT	AGA	GGC	AAC	AAC	ATT	CTT	GTC	ATG	TGT	GAT	ACT	TAC	ACC	CCT	GCG	GGT	GAA	CCA	ATC	CCT	ACG	AAC	AAG	AGA	CAT	GCT	GCA	GCT	364
BolGLN1.2_C1	TTC	CGT	AGA	GGC	AAC	AAC	ATT	CTT	GTC	ATG	TGT	GAT	ACT	TAC	ACC	CCT	GCG	GGT	GAA	CCA	ATC	CCT	ACG	AAC	AAG	AGA	CAT	GCT	GCA	GCT	312
EU822334	TTC	CGT	AGA	GGC	AAC	AAC	ATT	CTT	GTC	ATG	TGT	GAT	ACT	TAC	ACC	CCT	GCG	GGT	GAA	CCA	ATC	CCT	ACG	AAC	AAG	AGA	CAT	GCT	GCA	GCT	361
EU822335	TTC	CGT	AGA	GGC	AAC	AAC	ATT	CTT	GTC	ATG	TGT	GAT	ACT	TAC	ACC	CCT	GCG	GGT	GAA	CCA	ATC	CCT	ACG	AAC	AAG	AGA	CAT	GCT	GCA	GCT	358
At3g17820_GLN1.3__cds	TTC	AGG	AAA	GGC	AAC	AAC	ATC	CTG	GTG	ATG	TGT	GAT	GCT	TAC	ACA	CCA	GCT	GGT	GAT	CCT	ATT	CCA	ACC	AAC	AAG	AGG	CAC	AAC	GCT	GCT	333
BnaGLN1.3_C2	TTC	AGG	AAA	GGC	AAC	AAC	ATT	CTG	GTG	ATG	TGT	GAT	GCT	TAC	ACG	CCG	GCA	GGG	AAT	CCA	ATT	CCG	ACC	AAC	AAG	AGG	CAC	AAC	GCT	GCT	480
JX306693	TTC	AGG	AAA	GGC	AAC	AAC	ATT	CTG	GTG	ATG	TGT	GAT	GCT	TAC	ACG	CCG	GCA	GGG	AAT	CCA	ATT	CCG	ACC	AAC	AAG	AGG	CAC	AAC	GCT	GCT	348
Bra022247_cds	TTC	AGG	AAA	GGC	AAC	AAC	ATT	CTG	GTG	ATG	TGT	GAT	GCT	TAC	ACG	CCG	GCA	GGG	AAT	CCA	ATT	CCG	ACC	AAC	AAG	AGG	CAC	AAC	GCT	GCT	333
BraGLN1.3_C1	TTC	AGG	AAA	GGC	AAC	AAC	ATT	CTG	GTG	ATG	TGT	GAT	GCT	TAC	ACG	CCG	GCA	GGG	AAT	CCA	ATT	CCG	ACC	AAC	AAG	AGG	CAC	AAC	GCT	GCT	403
BnaGLN1.3_C1	TTC	AGG	AAA	GGC	AAC	AAC	ATT	CTG	GTG	ATG	TGT	GAT	GCT	TAC	ACG	CCG	GCA	GGG	AAT	CCA	ATT	CCG	ACC	AAC	AAG	AGG	CAC	AAC	GCT	GCT	400
JX306690	TTC	AGG	AAA	GGC	AAC	AAC	ATT	CTG	GTG	ATG	TGT	GAT	GCT	TAC	ACG	CCG	GCA	GGG	AAT	CCA	ATT	CCG	ACC	AAC	AAG	AGG	CAC	AAC	GCT	GCT	367
BolGLN1.3_C1	TTC	AGG	AAA	GGC	AAC	AAC	ATT	CTG	GTG	ATG	TGT	GAT	GCT	TAC	ACG	CCG	GCA	GGG	AAT	CCA	ATT	CCG	ACC	AAC	AAG	AGG	CAC	AAC	GCT	GCT	375
BnaGLN1.3_C4	TTC	AGG	AGA	GGC	AAC	AAC	ATT	CTG	GTG	ATG	TGT	GAT	GCT	TAC	ACG	CCG	GCC	GGT	AAT	CCA	ATT	CCG	ACC	AAC	AAG	AGG	CAC	AAC	GCT	GCA	402
Bra021276_cds	TTC	AGG	AGA	GGC	AAC	AAC	ATT	CTG	GTG	ATG	TGT	GAT	GCT	TAC	ACG	CCG	GCC	GGT	AAC	CCA	ATT	CCG	ACC	AAC	AAG	AGG	CAC	AAC	GCT	GCT	333
BraGLN1.3_C3	TTC	AGG	AGA	GGC	AAC	AAC	ATT	CTG	GTG	ATG	TGT	GAT	GCT	TAC	ACG	CCG	GCC	GGT	AAC	CCA	ATT	CCG	ACC	AAC	AAG	AGG	CAC	AAC	GCT	GCT	287
BnaGLN1.3_C3	TTC	AGG	AGA	GGC	AAC	AAC	ATT	CTG	GTG	ATG	TGT	GAT	GCT	TAC	ACG	CCG	GCC	GGT	AAT	CCA	ATT	CCG	ACC	AAC	AAG	AGG	CAT	AAC	GCT	GCT	360
BnaGLN1.3_C5	TTC	AGG	AGA	GGC	GAC	AAC	ATC	CTG	GTG	ATG	TGT	GAT	GCA	TAC	ACA	CCG	GCC	GGA	GAT	CCA	ATT	CCG	ACA	AAC	AAG	AGG	CAC	AAG	GCT	GCT	414
JX306694	TTC	AGG	AGA	GGC	GAC	AAC	ATC	CTG	GTG	ATG	TGT	GAT	GCA	TAC	ACA	CCG	GCC	GGA	GAT	CCA	ATT	CCG	ACA	AAC	AAG	AGG	CAC	AAG	GCT	GCT	389
Bra001686_cds	TTC	AGG	AGA	GGC	GAC	AAC	ATC	CTG	GTG	ATG	TGT	GAT	GCA	TAC	ACA	CCG	GCC	GGA	GAT	CCA	ATT	CCG	ACC	AAC	AAG	AGG	CAC	AAG	GCT	GCT	333
BraGLN1.3_C2	TTC	AGG	AGA	GGC	GAC	AAC	ATC	CTG	GTG	ATG	TGT	GAT	GCA	TAC	ACA	CCG	GCC	GGA	GAT	CCA	ATT	CCG	ACC	AAC	AAG	AGG	CAC	AAG	GCT	GCT	496
BnaGLN1.3_C6	TTC	AGG	AGA	GGC	GAC	AAC	ATC	CTG	GTG	ATG	TGT	GAT	GCA	TAC	ACA	CCG	GCC	GGA	GAT	CCA	ATT	CCG	ACC	AAC	AAG	AGG	CAC	AAG	GCT	GCT	387
BolGLN1.3_C2	TTC	AGA	AGA	GGT	GAC	AAC	ATC	CTG	GTG	ATG	TGT	GAT	GCA	TAC	ACA	CCG	GCC	GGA	GAT	CCA	ATC	CCA	ACC	AAC	AAG	AGG	CAC	AAG	GCT	GCT	279
At5g16570_GLN1.4__cds	TTC	AGA	AGA	GGC	AAC	AAC	ATC	CTT	GTG	ATG	TGT	GAC	GCA	TAT	ACA	CCG	GCA	GGA	GAG	CCG	ATT	CCG	ACG	AAC	AAA	AGG	CAT	GCG	GCG	GCT	333
BnaGLN1.4_C1	TTC	AGA	AGA	GGC	AAC	AAC	ATC	CTT	GTG	ATG	TGT	GAC	GCT	TAT	ACA	CCG	GCT	GGC	GAA	CCG	ATC	CCA	ACC	AAC	AAA	AGG	CAT	GCG	GCG	GCC	375
JX306697	TTC	AGA	AGA	GGC	AAC	AAC	ATC	CTT	GTG	ATG	TGT	GAC	GCT	TAT	ACA	CCG	GCT	GGC	GAA	CCG	ATC	CCA	ACC	AAC	AAA	AGG	CAT	GCG	GCG	GCC	350
JX306692	TTC	AGA	AGA	GGC	AAC	AAC	ATC	CTT	GTG	ATG	TGT	GAC	GCT	TAT	ACA	CCG	GCT	GGC	GAA	CCG	ATC	CCA	ACC	AAC	AAA	AGG	CAT	GCG	GCG	GCC	231
Bra023573_cds	TTC	AGA	AGA	GGC	AAC	AAC	ATC	CTT	GTG	ATG	TGT	GAC	GCT	TAT	ACA	CCG	GCT	GGC	GAA	CCG	ATC	CCA	ACC	AAC	AAA	AGG	CAT	GCG	GCA	GCT	330
BraGLN1.4_C1	TTC	AGA	AGA	GGC	AAC	AAC	ATC	CTT	GTG	ATG	TGT	GAC	GCT	TAT	ACA	CCG	GCT	GGC	GAA	CCG	ATC	CCA	ACC	AAC	AAA	AGG	CAT	GCG	GCA	GCT	455
BnaGLN1.4_C2	TTC	AGA	AGA	GGC	AAC	AAC	ATC	CTT	GTG	ATG	TGT	GAC	GCT	TAT	ACA	CCG	GCT	GGC	GAA	CCG	ATC	CCA	ACC	AAC	AAA	AGG	CAT	GCG	GCG	GCT	400
JX306695	TTC	AGA	AGA	GGC	AAC	AAC	ATC	CTT	GTG	ATG	TGT	GAC	GCT	TAT	ACA	CCG	GCT	GGC	GAA	CCG	ATC	CCA	ACC	AAC	AAA	AGG	CAT	GCG	GCG	GCT	374
JX306696	TTC	AGA	AGA	GGC	AAC	AAC	ATC	CTT	GTG	ATG	TGT	GAC	GCT	TAT	ACA	CCG	GCT	GGC	GAA	CCG	ATC	CCA	ACC	AAC	AAA	AGG	CAT	GCG	GCG	GCC	350
BolGLN1.4_C1	TTC	AGA	AGA	GGC	AAC	AAC	ATC	CTT	GTG	ATG	TGT	GAC	GCT	TAT	ACA	CCG	GCT	GGC	GAA	CCG	ATC	CCA	ACC	AAC	AAA	AGG	CAT	GCG	GCG	GCT	375
BnaGLN1.4_C4	TTC	AGA	AGA	GGC	AAC	AAC	ATC	CTT	GTG	ATG	TGT	GAT	GCA	TAT	ACA	CCG	GCC	GGC	GAA	CCG	ATC	CCG	ACA	AAC	AAA	AGG	CAT	GCG	GCG	GCC	336
JX306700	---	---	---	---	---	---	---	---	---	---	---	---	---	---	---	---	---	---	---	---	---	---	---	---	---	---	CAT	GCG	GCG	GCC	12
JX306701	---	---	---	---	---	---	---	---	---	---	---	---	---	---	---	---	---	---	---	---	---	---	---	---	---	---	CAT	GCG	GCG	GCC	12

Bra008612_cds	TTC	AGA	AGA	GGC	AAC	AAC	ATC	CTT	GTG	ATG	TGT	GAC	GCA	TAT	ACA	CCG	GCC	GGC	GAA	CCG	ATC	CCG	GCA	AAC	AAA	AGG	CAT	GCG	GCG	GCC	333
BraGLN1.4_C2	TTC	AGA	AGA	GGC	AAC	AAC	ATC	CTT	GTG	ATG	TGT	GAC	GCA	TAT	ACA	CCG	GCC	GGC	GAA	CCG	ATC	CCG	GCA	AAC	AAA	AGG	CAT	GCG	GCG	GCC	378
BnaGLN1.4_C3	TTC	AGA	AGA	GGC	AAC	AAC	ATC	CTT	GTA	ATG	TGT	GAC	GCA	TAT	ACA	CCG	GCC	GGT	GAA	CCG	ATT	CCG	ACG	AAC	AAA	AGG	CAT	GCT	GCA	GCT	377
JX306698	---	---	---	---	---	---	---	---	---	---	---	---	---	---	---	---	---	-GT	GAA	CCG	ATT	CCG	ACG	AAC	AAA	AGG	CAT	GCT	GCA	GCT	38
JX306699	---	---	---	---	---	---	---	---	---	---	---	---	---	---	---	---	---	-GT	GAA	CCG	ATT	CCG	ACG	AAC	AAA	AGG	CAT	GCT	GCA	GCT	38
BolGln1.4_C2	TTC	AGA	AGA	GGC	AAC	AAC	ATC	CTT	GTA	ATG	TGT	GAC	GCA	TAT	ACA	CCG	GCC	GGT	GAA	CCG	ATT	CCG	ACG	AAC	AAA	AGG	CAT	GCT	GCA	GCT	337
At1g48470_GLN1.5__cds	TTC	AGG	AAG	GGG	AAC	AAC	ATT	CTG	GTG	ATG	TGT	GAT	GCT	TAC	AGA	CCG	GCC	GGA	GAT	CCA	ATT	CCG	ACC	AAC	AAT	AGG	CAC	AAG	GCC	GTA	333
BnaGLN1.5_C2	TTC	AGG	AAA	GGG	AAT	AAC	ATT	CTC	GTG	ATG	TGT	GAT	GCT	TAC	ACA	CCG	AAA	GGA	GAT	CCA	ATC	CCG	ACC	AAC	AAT	AGG	CAC	AAA	GCC	GTG	455
Bra018729_cds	TTC	AGG	AAA	GGG	AAT	AAC	ATT	CTC	GTG	ATG	TGT	GAT	GCT	TAC	ACA	CCG	AAA	GGA	GAT	CCA	ATC	CCG	ACC	AAC	AAT	AGG	CAC	AAA	GCC	GTG	304
BraGLN1.5_C1	TTC	AGG	AAA	GGG	AAT	AAC	ATT	CTC	GTG	ATG	TGT	GAT	GCT	TAC	ACA	CCG	AAA	GGA	GAT	CCA	ATC	CCG	ACC	AAC	AAT	AGG	CAC	AAA	GCC	GTG	368
BnaGLN1.5_C1	TTC	AGG	AAA	GGG	AAT	AAC	ATT	CTC	GTG	ATG	TGT	GAT	GCT	TAC	ACA	CCG	AAA	GGA	GAT	CCA	ATC	CCG	ACC	AAC	AAT	AGG	CAC	AAA	GCC	GTG	430
JX306691	TTC	AGG	AAA	GGG	AAT	AAC	ATT	CTC	GTG	ATG	TGT	GAT	GCT	TAC	ACA	CCG	AAA	GGA	GAT	CCA	ATC	CCG	ACC	AAC	AAT	AGG	CAC	AAA	GCC	GTG	102
BolGLN1.5_C1	TTC	AGG	AAA	GGG	AAT	AAC	ATT	CTC	GTG	ATG	TGT	GAT	GCT	TAC	ACA	CCG	AAA	GGA	GAT	CCA	ATC	CCG	ACC	AAC	AAT	AGG	CAC	AAA	GCC	GTG	414

BnaGLN1_JXB

```
                                                  820                                    840                                    860                                    880                                    900
                                                   |                                      |                                      |                                      |                                      |
At5g37600_GLN1.1__cds AAG GTC TTT AGC AAC CCT GAT GTT GCA GCT GAA GTG CCA TGG TAT GGT ATT GAG CAA GAA TAC ACT TTA CTC CAG AAA GAT GTG AAG TGG 423
         BnaGLN1.1_C1 AAG GTC TTT AGC CAC CCT GAT GTT GTA GCT GAA GTG CCA TGG TAT GGT ATT GAG CAA GAG TAT ACT TTA CTT CAG AAA GAT GTG AAC TGG 511
               X82997 AAG GTC TTT AGC CAC CCT GAT GTT GTA GCT GAA GTG CCA TGG TAT GGT ATT GAG CAA GAG TAT ACT TTA CTT CAG AAA GAT GTG AAC TGG 484
        Bra028132_cds AAG GTC TTT AGC CAC CCT GAT GTT GTA GCT GAA GTG CCA TGG TAT GGT ATT GAG CAA GAG TAT ACT TTA CTT CAG AAA GAT GTG AAC TGG 423
         BraGLN1.1_C1 AAG GTC TTT AGC CAC CCT GAT GTT GTA GCT GAA GTG CCA TGG TAT GGT ATT GAG CAA GAG TAT ACT TTA CTT CAG AAA GAT GTG AAC TGG 514
         BnaGLN1.1_C2 AAG GTC TTT AGC CAC CCT GAT GTT GTA GCT GAA GTG CCA TGG TAT GGT ATT GAG CAA GAG TAC ACT TTA CTT CAG AAA GAT GTG AAG TGG 511
               Y12460 AAG GTC TTT AGC CAC CCT GAT GTT GTA GCT GAA GTG CCA TGG TAT GGT ATT GAG CAA GAG TAC ACT TTA CTT CAG AAA GAT GTG AAG TGG 402
         BolGLN1.1_C1 AAG GTC TTT AGC CAC CCT GAT GTT GTA GCT GAA GTG CCA TGG TAT GGT ATT GAG CAA GAG TAC ACT TTA CTT CAG AAA GAT GTG AAG TGG 505
At1g66200.1_GLN1.2__cds GAG ATC TTT GCT AAC CCT GAT GTT ATT GCT GAA GTG CCA TGG TAT GGA ATC GAA CAA GAA TAC ACT TTG TTG CAG AAG GAT GTG AAC TGG 423
         BnaGLN1.2_C1 CAG ATC TTT AGC AAC CCT GAT GTT GTT GCT GAA GTG CCA TGG TAT GGA ATC GAA CAA GAA TAC ACT CTG TTG CAG AAA GAT GTG AAT TGG 509
               X76736 CAG ATC TTT AGC AAC CCT GAT GTT GTT GCT GAA GTG CCA TGG TAT GGA ATC GAA CAA GAA TAC ACT CTG TTG CAG AAA GAT GTG AAT TGG 490
        Bra039756_cds CAG ATC TTT AGC AAC CCT GAT GTT GTT GCT GAA GTG CCA TGG TAC GGA ATC GAA CAA GAA TAC ACT CTG TTG CAG AAA GAT GTG AAT TGG 423
         BraGLN1.2_C1 CAG ATC TTT AGC AAC CCT GAT GTT GTT GCT GAA GTG CCA TGG TAC GGA ATC GAA CAA GAA TAC ACT CTG TTG CAG AAA GAT GTG AAT TGG 505
             EU499383 CAG ATC TTT AGC AAC CCT GAT GTT GTT GCT GAA GTG CCA TGG TAT GGA ATC GAA CAA GAA TAC ACT CTG TTG CAG AAA GAT GTG AAT TGG 507
             AY773089 CAG ATC TTT AGC AAC CCT GAT GTT GTT GCT GAA GTG CCA TGG TAC GGA ATC GAA CAA GAA TAC ACT CTG TTG CAG AAA GAT GTG AAT TGG 511
         BnaGLN1.2_C2 CAG ATC TTT AGC AAC CCT GAT GTT GTT GCT GAA GTG CCA TGG TAT GGA ATC GAA CAA GAG TAC ACT CTG TTG CAG AAA GAT GTG AAG TGG 512
               Y12459 CAG ATC TTT AGC AAC CCT GAT GTT GTT GCT GAA GTG CCA TGG TAT GGA ATC GAA CAA GAG TAC ACT CTG TTG CAG AAA GAT GTG AAG TGG 454
         BolGLN1.2_C1 CAG ATC TTT AGC AAC CCT GAT GTT GTT GCT GAA GTG CCA TGG TAT GGA ATC GAA CAA GAG TAC ACT CTG TTG CAG AAA GAT GTG AAG TGG 402
             EU822334 CAG ATC TTT AGC AAC CCT GAT GTT GTT GCT GAA GTG CCA TGG TAT GGA ATC GAA CAA GAG TAC ACT CTG TTG CAG AAA GAT GTG AAG TGG 451
             EU822335 CAG ATC TTT AGC AAC CCT GAT GTT GTT GCT GAA GTG CCA TGG TAT GGA ATC GAA CAA GAG TAC ACT CTG TTG CAG AAA GAT GTG AAG TGG 448
At3g17820_GLN1.3__cds AAG ATC TTC AGC CAC CCC GAC GTT GCC AAG GAG GAG CCT TGG TAT GGG ATT GAG CAA GAA TAC ACT TTG ATG CAA AAG GAT GTG AAC TGG 423
         BnaGLN1.3_C2 AAG ATC TTC AGC AAC CCC AAA GTT GCC TCT GAG GAG CCT TGG TAT GGG ATT GAG CAA GAA TAC ACA TTG ATG CAG AAG GGT GTG AAC TGG 570
             JX306693 AAG ATC TTC AGC AAC CCC AAA GTT GCC TCT GAG GAG CCT TGG TAT GGG ATT GAG CAA GAA TAC ACA TTG ATG CAG AAG GGT GTG AAC TGG 438
        Bra022247_cds AAG ATC TTC AGC AAC CCC AAA GTT GCC TCT GAG GAG CCT TGG TAT GGG ATT GAG CAA GAA TAC ACA TTG ATG CAG AAG GGT GTG AAC TGG 423
         BraGLN1.3_C1 AAG ATC TTC AGC AAC CCC AAA GTT GCC TCT GAG GAG CCT TGG TAT GGG ATT GAG CAA GAA TAC ACA TTG ATG CAG AAG GGT GTG AAC TGG 493
         BnaGLN1.3_C1 AAG ATC TTC AGC AAC CCC AAA GTT GCC TCT GAG GAG CCT TGG TAT GGG ATT GAA CAA GAA TAC ACA TTG ATG CAA AAG GGT GTG AAC TGG 490
             JX306690 AAG ATC TTC AGC AAC CCC AAA GTT GCC TCT GAG GAG CCT TGG TAT GGG ATT GAA CAA GAA TAC ACA TTG ATG CAA AAG GGT GTG AAC TGG 457
         BolGLN1.3_C1 AAG ATC TTC AGC AAC CCC AAA GTT GCC TCT GAG GAG CCT TGG TAT GGG ATT GAA CAA GAA TAC ACA TTG ATG CAA AAG GGT GTG AAC TGG 465
         BnaGLN1.3_C4 AAG ATC TTC AGC AAC TCA AAA GTT GCC TCT GAG GAG CCT TGG TAT GGG ATT GAG CAA GAA TAC ACA TTG ATG CAA AAG GGT GTG AAT TGG 492
        Bra021276_cds AAG ATC TTC AGC AAC TCA AAA GTT GCC TCT GAG GAG CCT TGG TAT GGG ATT GAG CAA GAA TAC ACA TTG ATG CAA AAG GGT GTG AAC TGG 423
         BraGLN1.3_C3 AAG ATC TTC AGC AAC TCA AAA GTT GCC TCT GAG GAG CCT TGG TAT GGG ATT GAG CAA GAA TAC ACA TTG ATG CAA AAG GGT GTG AAC TGG 377
         BnaGLN1.3_C3 AAG ATC TTC AGC AAC TCT AAA GTT GCC TCT GAG GAG CCT TGG TAT GGG ATT GAG CAA GAA TAC ACA TTG ATG CAA AAG GGT GTG AAC TGG 450
         BnaGLN1.3_C5 AAG ATC TTT AGC CAT CCT AAC GTT GCC AAG GAG GTG CCT TGG TAT GGG ATT GAG CAA GAA TAC ACT TTG ATG CAA AAG GGT GTG AAC TGG 504
             JX306694 AAG ATC TTT AGC CAT CCT AAC GTT GCC AAG GAG GTG CCT TGG TAT GGG ATT GAG CAA GAA TAC ACT TTG ATG CAA AAG GGT GTG AAC TGG 479
        Bra001686_cds AAG ATC TTC AGC CAT CCT AAC GTT GCC AAA GAA GTG CCT TGG TAT GGG ATT GAG CAA GAA TAC ACA TTG ATG CAA AAG GGT GTG AAC TGG 423
         BraGLN1.3_C2 AAG ATC TTC AGC CAT CCT AAC GTT GCC AAA GAA GTG CCT TGG TAT GGG ATT GAG CAA GAA TAC ACA TTG ATG CAA AAG GGT GTG AAC TGG 586
         BnaGLN1.3_C6 AAG ATC TTC AGC CAT CCT AAC GTT GCC AAA GAA GTG CCT TGG TAT GGG ATT GAG CAA GAA TAC ACT TTG ATG CAA AAG GGT ATG AAC TGG 477
         BolGLN1.3_C2 AAG ATC TTC AGC CAT CCT AAC GTT GCC AAG GAG GTG CCT TGG TAT GGG ATT GAG CAA GAA TAC ACT TTG ATG CAA AAG GGT GTG AAC TGG 369
At5g16570_GLN1.4__cds AAG ATC TTT GAA GAC CCT AGT GTT GTC GCC GAA GAA ACA TGG TAC GGA ATT GAA CAA GAG TAT ACC TTG TTG CAA AAG GAT ATT AAG TGG 423
         BnaGLN1.4_C1 AAG ATC TTT AGC GAC CCA ACC GTT GCC GCC GAA GAA ACA TGG TAC GGA ATT GAG CAA GAG TAT ACT TTG CTC CAA AAG GAT ACT AAG TGG 465
             JX306697 AAG ATC TTT AGC GAC CCA ACC GTT GCC GCC GAA GAA ACA TGG TAC GGA ATT GAG CAA GAG TAT ACT TTG CTC CAA AAG GAT ACT AAG TGG 440
             JX306692 AAG ATC TTT AGC GAC CCA ACC GTT GCC GCC GAA GAA ACA TGG TAC GGA ATT GAG CAA GAG TAT ACT TTG CTC CAA AAG GAT ACT AAG TGG 321
        Bra023573_cds AAG ATC TTT AGC GAC CCA ACC GTT GCC GCC GAA GAA ACA TGG TAT GGA ATT GAG CAA GAG TAT ACT TTG CTT CAA AAG GAT ACT AAG TGG 420
         BraGLN1.4_C1 AAG ATC TTT AGC GAC CCA ACC GTT GCC GCC GAA GAA ACA TGG TAT GGA ATT GAG CAA GAG TAT ACT TTG CTT CAA AAG GAT ACT AAG TGG 545
         BnaGLN1.4_C2 AAG ATC TTT AGC GAC CCA ACC GTT GCC GCC GAA GAA ACA TGG TAT GGA ATT GAG CAA GAG TAT ACT TTG CTT CAA AAG GAT ACC AAG TGG 490
             JX306695 AAG ATC TTT AGC GAC CCA ACC GTT GCC GCC GAA GAA ACA TGG TAT GGA ATT GAG CAA GAG TAT ACT TTG CTT CAA AAG GAT ACC AAG TGG 464
             JX306696 AAG ATC TTT AGC GAC CCA ACC GTT GCC GCC GAA GAA ACA TGG TAC GGA ATT GAG CAA GAG TAT ACT TTG CTC CAA AAG GAT ACT AAG TGG 440
         BolGLN1.4_C1 AAG ATC TTT AGC GAC CCA ACC GTT GCC GCC GAA GAA ACA TGG TAT GGA ATT GAG CAA GAG TAT ACT TTG CTT CAA AAG GAT ACC AAG TGG 465
         BnaGLN1.4_C4 AAG ATC TTT AGC GAC CCG AGC GTT GCC GCC GAA GAA ACA TGG TAT GGA ATT GAG CAA GAG TAT ACT TTG CTA CAA AAG GAT ATT AAA TGG 426
             JX306700 AAG ATC TTT AGC GAC CCG AGC GTT GCC GCC GAA GAA ACA TGG TAT GGA ATT GAG CAA GAG TAT ACT TTG CTA CAA AAG GAT ATT AAA TGG 102
             JX306701 AAG ATC TTT AGC GAC CCG AGC GTT GCC GCC GAA GAA ACA TGG TAT GGA ATT GAG CAA GAG TAT ACT TTG CTA CAA AAG GAT ATT AAA TGG 102
```

Bra008612_cds	AAG	ATC	TTT	AGC	GAC	CCG	AGC	GTT	GCC	GCC	GAA	GAA	ACA	TGG	TAT	GGA	ATT	GAG	CAA	GAG	TAT	ACT	TTG	CTA	CAA	AAG	GAT	ATT	AAA	TGG	423
BraGLN1.4_C2	AAG	ATC	TTT	AGC	GAC	CCG	AGC	GTT	GCC	GCC	GAA	GAA	ACA	TGG	TAT	GGA	ATT	GAG	CAA	GAG	TAT	ACT	TTG	CTA	CAA	AAG	GAT	ATT	AAA	TGG	468
BnaGLN1.4_C3	AAG	ATC	TTT	AGC	GAC	CCC	AGC	GTT	GCC	GCC	GAA	GAA	ACA	TGG	TAT	GGA	ATT	GAG	CAA	GAG	TAT	ACT	TTG	CTT	CAA	AAG	GAT	ATT	AAG	TGG	467
JX306698	AAG	ATC	TTT	AGC	GAC	CCC	AGC	GTT	GCC	GCC	GAA	GAA	ACA	TGG	TAT	GGA	ATT	GAG	CAA	GAG	TAT	ACT	TTG	CTT	CAA	AAG	GAT	ATT	AAG	TGG	128
JX306699	AAG	ATC	TTT	AGC	GAC	CCC	AGC	GTT	GCC	GCC	GAA	GAA	ACA	TGG	TAT	GGA	ATT	GAG	CAA	GAG	TAT	ACT	TTG	CTT	CAA	AAG	GAT	ATT	AAG	TGG	128
BolGln1.4_C2	AAG	ATC	TTT	AGC	GAC	CCC	AGC	GTT	GCC	GCC	GAA	GAA	ACA	TGG	TAT	GGA	ATT	GAG	CAA	GAG	TAT	ACT	TTG	CTT	CAA	AAG	GAT	ATT	AAG	TGG	427
At1g48470_GLN1.5__cds	AAA	ATC	TTC	GAT	CAT	CCC	AAT	GTG	AAG	GCT	GAA	GAG	CCT	TGG	TTT	GGG	ATA	GAG	CAA	GAA	TAC	ACA	TTA	CTT	AAA	AAA	GAT	GTG	AAG	TGG	423
BnaGLN1.5_C2	AAA	ATC	TTC	GAT	CAT	CCC	AAT	GTG	AAG	GCT	GAA	GAG	CCT	TGG	TTT	GGG	ATA	GAG	CAA	GAA	TAC	ACA	TTA	CTT	AAG	AAA	GAC	GTC	AAG	TGG	545
Bra018729_cds	AAA	ATC	TTC	GAT	CAT	CCC	AAT	GTG	AAG	GCT	GAA	GAG	CCT	TGG	TTT	GGG	ATA	GAG	CAA	GAA	TAC	ACA	TTA	CTT	AAG	AAA	GAC	GTC	AAG	TGG	394
BraGLN1.5_C1	AAA	ATC	TTC	GAT	CAT	CCC	AAT	GTG	AAG	GCT	GAA	GAG	CCT	TGG	TTT	GGG	ATA	GAG	CAA	GAA	TAC	ACA	TTA	CTT	AAG	AAA	GAC	GTC	AAG	TGG	458
BnaGLN1.5_C1	AAA	ATC	TTC	GAT	CAT	CCC	AAT	GTG	AAG	GCT	GAA	GAG	CCT	TGG	TTT	GGG	ATA	GAG	CAA	GAA	TAC	ACA	TTA	CTT	AAG	AAA	GAC	GTC	AAG	TGG	520
JX306691	AAA	ATC	TTC	GAT	CAT	CCC	AAT	GTG	AAG	GCT	GAA	GAG	CCT	TGG	TTT	GGG	ATA	GAG	CAA	GAA	TAC	ACA	TTA	CTT	AAG	AAA	GAC	GTC	AAG	TGG	192
BolGLN1.5_C1	AAA	ATC	TTC	GAT	CAT	CCC	AAT	GTG	AAG	GCT	GAA	GAG	CCT	TGG	TTT	GGG	ATA	GAG	CAA	GAA	TAC	ACA	TTA	CTT	AAG	AAA	GAC	GTC	AAG	TGG	504

BnaGLN1_JXB

						920							940						960							980				
At5g37600_GLN1.1__cds	CCT	GTT	GGT	TGG	CCT	ATT	GGT	GGT	TAT	CCC	GGC	CCT	CAG	GGA	CCG	TAC	TAT	TGC	GGT	ATT	GGA	GCA	GAC	AAA	TCT	TTT	---	GGC	AGA	GAT	510
BnaGLN1.1_C1	CCT	CTT	GGT	TGG	CCT	ATT	GGC	GGC	TTC	CCT	GGC	CCT	CAG	GGA	CCA	TAC	TAT	TGT	AGT	GTT	GGA	GCA	GAT	AAA	TCT	TTT	---	GGT	AGA	GAC	598
X82997	CCT	CTT	GGT	TGG	CCT	ATT	GGC	GGC	TTC	CCT	GGC	CCT	CAG	GGA	CCA	TAC	TAT	TGT	AGT	GTT	GGA	GCA	GAT	AAA	TCT	TTT	---	GGT	AGA	GAC	571
Bra028132_cds	CCT	CTT	GGT	TGG	CCT	CTT	GGC	GGC	TTC	CCT	GGC	CCT	CAG	GGA	CCA	TAC	TAT	TGT	AGT	GTT	GGA	GCA	GAT	AAA	TCT	TTT	---	GGT	AGA	GAC	510
BraGLN1.1_C1	CCT	CTT	GGT	TGG	CCT	CTT	GGC	GGC	TTC	CCT	GGC	CCT	CAG	GGA	CCA	TAC	TAT	TGT	AGT	GTT	GGA	GCA	GAT	AAA	TCT	TTT	---	GGT	AGA	GAC	601
BnaGLN1.1_C2	CCT	GTT	GGT	TGG	CCT	ATT	GGT	GGC	TTC	CCC	GGT	CCT	CAG	GGA	CCA	TAC	TAT	TGT	AGT	GTT	GGA	GCA	GAT	AAA	TCT	TTT	---	GGT	AGA	GAC	598
Y12460	CCT	GTT	GGT	TGG	CCT	ATT	GGT	GGC	TTC	CCC	GGT	CCT	CAG	GGA	CCA	TAC	TAT	TGT	AGT	GTT	GGA	GCA	GAT	AAA	TCT	TTT	---	GGT	AGA	GAC	489
BolGLN1.1_C1	CCT	GTT	GGT	TGG	CCT	ATT	GGT	GGC	TTC	CCC	GGT	CCT	CAG	GGA	CCA	TAC	TAT	TGT	AGT	GTT	GGA	GCA	GAT	AAA	TCT	TTT	---	GGT	AGA	GAC	592
At1g66200.1_GLN1.2__cds	CCT	CTT	GGA	TGG	CCC	ATT	GGT	GGC	TTC	CCT	GGC	CCT	CAG	GGA	CCA	TAC	TAC	TGC	AGT	ATT	GGA	GCT	GAC	AAA	TCT	TTT	---	GGA	AGA	GAC	510
BnaGLN1.2_C1	CCT	GTC	GGA	TGG	CCC	ATT	GGT	GGA	TTC	CCC	GGC	CCT	CAG	GGA	CCA	TAC	TAC	TGC	AGT	GTT	GGA	GCT	GAC	AAA	TCT	TTT	---	GGA	AGA	GAC	596
X76736	CCT	GTC	GGA	TGG	CCC	ATT	GGT	GGA	TTC	CCC	GGC	CCT	CAG	GGA	CCA	TAC	TAC	TGC	AGT	GTT	GGA	GCT	GAC	AAA	TCT	TTT	---	GGA	AGA	GAC	577
Bra039756_cds	CCT	GTC	GGA	TGG	CCC	ATT	GGT	GGA	TTC	CCC	GGC	CCT	CAG	GGA	CCA	TAC	TAC	TGC	AGT	GTT	GGA	GCT	GAC	AAA	TCT	TTT	---	GGA	AGA	GAC	510
BraGLN1.2_C1	CCT	GTC	GGA	TGG	CCC	ATT	GGT	GGA	TTC	CCC	GGC	CCT	CAG	GGA	CCA	TAC	TAC	TGC	AGT	GTT	GGA	GCT	GAC	AAA	TCT	TTT	---	GGA	AGA	GAC	592
EU499383	CCT	GTC	GGA	TGG	CCC	ATT	GGT	GGA	TTC	CCC	GGC	CCT	CAG	GGA	CCA	TAC	TAC	TGC	AGT	GTT	GGA	GCT	GAC	AAA	TCT	TTT	---	GGA	AGA	GAC	594
AY773089	CCT	GTC	GGA	TGG	CCC	ATT	GGT	GGA	TTC	CCC	GGC	CCT	CAG	GGA	CCA	TAC	TAC	TGC	AGT	GTT	GGA	GCT	GAC	AAA	TCT	TTT	---	GGA	AGA	GAC	598
BnaGLN1.2_C2	CCT	GTT	GGA	TGG	CCC	ATT	GGT	GGA	TTC	CCC	GGC	CCT	CAG	GGA	CCA	TAC	TAC	TGC	AGT	GTT	GGA	GCT	GAC	AAA	TCT	TTT	---	GGA	AGA	GAC	599
Y12459	CCT	GTT	GGA	TGG	CCC	ATT	GGT	GGA	TTC	CCC	GGC	CCT	CAG	GGA	CCA	TAC	TAC	TGC	AGT	GTT	GGA	GCT	GAC	AAA	TCT	TTT	---	GGA	AGA	GAC	541
BolGLN1.2_C1	CCT	GTT	GGA	TGG	CCC	ATT	GGT	GGA	TTC	CCC	GGC	CCT	CAG	GGA	CCA	TAC	TAC	TGC	AGT	GTT	GGA	GCT	GAC	AAA	TCT	TTT	---	GGA	AGA	GAC	489
EU822334	CCT	GTT	GGA	TGG	CCC	ATT	GGT	GGA	TTC	CCC	GGC	CCT	CAG	GGA	CCA	TAC	TAC	TGC	AGT	GTT	GGA	GCT	GAC	AAA	TCT	TTT	---	GGA	AGA	GAC	538
EU822335	CCT	GTT	GGA	TGG	CCC	ATT	GGT	GGA	TTC	CCC	GGC	CCT	CAG	GGA	CCA	TAC	TAC	TGC	AGT	GTT	GGA	GCT	GAC	AAA	TCT	TTT	---	GGA	AGA	GAC	535
At3g17820_GLN1.3__cds	CCA	ATT	GGT	TGG	CCT	GTT	GGT	GGC	TAC	CCT	GGC	CCT	CAG	GGA	CCT	TAC	TAC	TGT	GGT	GTG	GGA	GCT	GAC	AAA	GCC	ATT	---	GGT	CGT	GAC	510
BnaGLN1.3_C2	CCT	ATT	GGT	TGG	CCT	ATC	GGT	GGC	TTC	CCT	GGC	CCT	CAG	GGA	CCT	TAC	TAC	TGT	GGT	GTG	GGA	GCT	GAC	AAG	GCA	ATT	---	GGT	CGT	GAC	657
JX306693	CCT	ATT	GGT	TGG	CCT	ATC	GGT	GGC	TTC	CCT	GGC	CCT	CAG	GGA	CCT	TAC	TAC	TGT	GGT	GTG	GGA	GCT	GAC	AAG	GCA	ATT	---	GGT	CGT	GAC	525
Bra022247_cds	CCC	ATT	GGT	TGG	CCT	ATC	GGT	GGC	TTC	CCT	GGC	CCA	CAG	GGA	CCA	TAC	TAC	TGT	GGT	GTG	GGA	GCT	GAC	AAA	GCC	ATT	---	GGT	CGT	GAC	510
BraGLN1.3_C1	CCC	ATT	GGT	TGG	CCT	ATC	GGT	GGC	TTC	CCT	GGC	CCA	CAG	GGA	CCA	TAC	TAC	TGT	GGT	GTG	GGA	GCT	GAC	AAA	GCC	ATT	---	GGT	CGT	GAC	580
BnaGLN1.3_C1	CCC	ATT	GGT	TGG	CCT	ATC	GGT	GGC	TTC	CCT	GGC	CCA	CAG	GGA	CCA	TAC	TAC	TGT	GGT	GTG	GGA	GCT	GAC	AAA	GCC	ATT	---	GGT	CGT	GAC	577
JX306690	CCC	ATT	GGT	TGG	CCT	ATC	GGT	GGC	TTC	CCT	GGC	CCA	CAG	GGA	CCA	TAC	TAC	TGT	GGT	GTG	GGA	GCT	GAC	AAA	GCC	ATT	---	GGT	CGT	GAC	544
BolGLN1.3_C1	CCC	ATT	GGT	TGG	CCT	ATC	GGT	GGC	TTC	CCT	GGC	CCA	CAG	GGA	CCA	TAC	TAC	TGT	GGT	GTG	GGA	GCT	GAC	AAA	GCC	ATT	---	GGT	CGT	GAC	552
BnaGLN1.3_C4	CCC	ATT	GGT	TGG	CCT	GTT	GGT	GGC	TTC	CCT	GGC	CCA	CAG	GGA	CCG	TAC	TAC	TGT	GGC	GTG	GGA	GCT	GAC	AAA	GCC	ATT	---	GGT	CGT	GAC	579
Bra021276_cds	CCC	ATT	GGT	TGG	CCT	GTG	GGT	GGC	TTC	CCT	GGC	CCA	CAG	GGA	CCG	TAC	TAC	TGC	GGC	GTG	GGA	GCT	GAC	AAA	GCC	ATT	---	GGT	CGT	GAC	510
BraGLN1.3_C3	CCC	ATT	GGT	TGG	CCT	GTG	GGT	GGC	TTC	CCT	GGC	CCA	CAG	GGA	CCG	TAC	TAC	TGC	GGC	GTG	GGA	GCT	GAC	AAA	GCC	ATT	---	GGT	CGT	GAC	464
BnaGLN1.3_C3	CCT	ATT	GGT	TGG	CCT	GTT	GGT	GGC	TTC	CCT	GGC	CCA	CAG	GGA	CCA	TAC	TAC	TGT	GGT	GTG	GGA	GCT	GAC	AAA	GCC	ATT	---	GGT	CGT	GAC	537
BnaGLN1.3_C5	CCT	ATT	GGT	TGG	CCA	ATT	GGT	GGC	TTC	CCT	GGT	CCT	CAG	GGA	CCA	TAC	TAC	TGT	GGT	GTG	GGA	GCT	GAC	AAA	GCC	ATT	---	GGT	CGT	GAC	591
JX306694	CCT	ATT	GGT	TGG	CCA	ATT	GGT	GGC	TTC	CCT	GGT	CCT	CAG	GGA	CCA	TAC	TAC	TGT	GGT	GTG	GGA	GCT	GAC	AAA	GCC	ATT	---	GGT	CGT	GAC	566
Bra001686_cds	CCT	ATT	GGA	TGG	CCA	ATT	GGT	GGC	TTC	CCT	GGT	CCT	CAG	GGA	CCA	TAC	TAC	TGT	GGT	GTG	GGA	GCT	GAC	AAA	GCC	ATT	---	GGT	CGT	GAC	510
BraGLN1.3_C2	CCT	ATT	GGA	TGG	CCA	ATT	GGT	GGC	TTC	CCT	GGT	CCT	CAG	GGA	CCA	TAC	TAC	TGT	GGT	GTG	GGA	GCT	GAC	AAA	GCC	ATT	---	GGT	CGT	GAC	673
BnaGLN1.3_C6	CCT	ATT	GGT	TGG	CCT	GTT	GGT	GGC	TTC	CCT	GGT	CCT	CAG	GGA	CCA	TAC	TAC	TGT	GGT	GTG	GGA	GCT	GAC	AAA	GCC	ATT	---	GGT	CGT	GAC	564
BolGLN1.3_C2	CCA	ATT	GGT	TGG	CCT	ATT	GGT	GGC	TTC	CCT	GGT	CCT	CAG	GGA	CCA	TAC	TAC	TGT	GGT	GTG	GGA	GCT	GAC	AAA	GCC	ATT	---	GGT	CGT	GAC	456
At5g16570_GLN1.4__cds	CCG	GTA	GGT	TGG	CCG	GTC	GGC	GGT	TTC	CCA	GGT	CCT	CAG	GGA	CCG	TAC	TAC	TGT	GGA	GTT	GGA	GCA	GAC	AAA	GCC	TTT	---	GGA	AGA	GAC	510
BnaGLN1.4_C1	CCA	GTT	GGT	TGG	CCC	GTC	GGT	GGC	TTC	CCA	GGT	CCT	CAG	GGA	CCA	TAC	TAC	TGT	GGA	GTT	GGA	GCA	GAC	AAA	GCC	TTT	---	GGA	AGA	GAC	552
JX306697	CCA	GTT	GGT	TGG	CCC	GTC	GGT	GGC	TTC	CCA	GGT	CCT	CAG	GGA	CCA	TAC	TAC	TGT	GGA	GTT	GGA	GCA	GAC	AAA	GCC	TTT	---	GGA	AGA	GAC	527
JX306692	CCA	GTT	GGT	TGG	CCC	GTC	GGT	GGC	TTC	CCA	GGT	CCT	CAG	GGA	CCA	TAC	TAC	TGT	GGA	GTT	GGA	GCA	GAC	AAA	GCC	TTT	---	GGA	AGA	GAC	408
Bra023573_cds	CCA	GTT	GGT	TGG	CCC	GTC	GGT	GGC	TTC	CCA	GGT	CCT	CAG	GGA	CCA	TAC	TAC	TGT	GGA	GTT	GGA	GCA	GAC	AAA	GCC	TTT	---	GGA	AGA	GAC	507
BraGLN1.4_C1	CCA	GTT	GGT	TGG	CCC	GTC	GGT	GGC	TTC	CCA	GGT	CCT	CAG	GGA	CCA	TAC	TAC	TGT	GGA	GTT	GGA	GCA	GAC	AAA	GCC	TTT	---	GGA	AGA	GAC	632
BnaGLN1.4_C2	CCA	GTT	GGT	TGG	CCC	GTC	GGT	GGC	TTC	CCA	GGT	CCT	CAG	GGA	CCA	TAC	TAC	TGT	GGA	GTT	GGA	GCA	GAC	AAA	GCC	TTT	---	GGA	AGA	GAC	577
JX306695	CCA	GTT	GGT	TGG	CCC	GTC	GGT	GGC	TTC	CCA	GGT	CCT	CAG	GGA	CCA	TAC	TAC	TGT	GGA	GTT	GGA	GCA	GAC	AAA	GCC	TTT	---	GGA	AGA	GAC	551
JX306696	CCA	GTT	GGT	TGG	CCC	GTC	GGT	GGC	TTC	CCA	GGT	CCT	CAG	GGA	CCA	TAC	TAC	TGT	GGA	GTT	GGA	GCA	GAC	AAA	GCC	TTT	--T	GGA	AGA	GAC	528
BolGLN1.4_C1	CCA	GTT	GGT	TGG	CCC	GTC	GGT	GGC	TTC	CCA	GGT	CCT	CAG	GGA	CCA	TAC	TAC	TGT	GGA	GTT	GGA	GCA	GAC	AAA	GCC	TTT	---	GGA	AGA	GAC	552
BnaGLN1.4_C4	CCG	GTA	GGT	TGG	CCT	GTT	GGC	GGC	TTC	CCA	GGT	CCT	CAG	GGA	CCG	TAC	TAC	TGT	GGT	GCT	GGA	GCA	GAC	AAA	GCC	TTT	---	GGA	AGA	GAC	513
JX306700	CCG	GTA	GGT	TGG	CCT	GTT	GGC	GGC	TTC	CCA	GGT	CCT	CAG	GGA	CCG	TAC	TAC	TGT	GGT	GCT	GGA	GCA	GAC	AAA	GCC	TTT	---	GGA	AGA	GAC	189
JX306701	CCG	GTA	GGT	TGG	CCT	GTT	GGC	GGC	TTC	CCA	GGT	CCT	CAG	GGA	CCG	TAC	TAC	TGT	GGT	GCT	GGA	GCA	GAC	AAA	GCC	TTT	---	GGA	AGA	GAC	189

```
    Bra008612_cds CCG GTA GGT TGG CCT GTT GGC GGC TTC CCA GGT CCT CAG GGA CCG TAC TAC TGT GGT GTT GGA GCA GAC AAA GCC TTT --- GGA AGA GAC 510
     BraGLN1.4_C2 CCG GTA GGT TGG CCT GTT GGC GGC TTC CCA GGT CCT CAG GGA CCG TAC TAC TGT GGT GTT GGA GCA GAC AAA GCC TTT --- GGA AGA GAC 555
     BnaGLN1.4_C3 CCG GTA GGT TGG CCT GTT GGC GGC TTC CCA GGT CCT CAG GGA CCG TAC TAC TGT GGA GTT GGA GCA GAC AAA GCC TTT --- GGA AGA GAC 554
         JX306698 CCG GTA GGT TGG CCT GTT GGC GGC TTC CCA GGT CCT CAG GGA CCG TAC TAC TGT GGA GTT GGA GCA GAC AAA GCC TTT --- GGA AGA GAC 215
         JX306699 CCG GTA GGT TGG CCT GTT GGC GGC TTC CCA GGT CCT CAG GGA CCG TAC TAC TGT GGA GTT GGA GCA GAC AAA GCC TTT --- GGA AGA GAC 215
     BolGln1.4_C2 CCG GTA GGT TGG CCT GTT GGC GGC TTC CCA GGT CCT CAG GGA CCG TAC TAC TGT GGA GTT GGA GCA GAC AAA GCC TTT --- GGA AGA GAC 514
At1g48470_GLN1.5__cds CCA CTA GGT TGG CCT CTT GGT GGC TTT CCT GGT CCT CAG GGA CCG TAC TAT TGT GCA GTA GGT GCA GAC AAA GCT TTT --- GGT CGT GAC 510
     BnaGLN1.5_C2 CCA TTG GGT TGG CCC CTT GGT GGC TTT CCT GGT CCT CAG GGA CCG TAC TAT TGT GCG GTG GGT GCA GAC AAA GCC TTT --- GGG CGT GAC 632
    Bra018729_cds CCA TTG GGT TGG CCC CTT GGT GGC TTT CCT GGT CCT CAG GGA CCG TAC TAT TGT GCG GTG GGT GCA GAC AAA GCC TTT --- GGG CGT GAC 481
     BraGLN1.5_C1 CCA TTG GGT TGG CCC CTT GGT GGC TTT CCT GGT CCT CAG GGA CCG TAC TAT TGT GCG GTG GGT GCA GAC AAA GCC TTT --- GGG CGT GAC 545
     BnaGLN1.5_C1 CCA TTG GGT TGG CCC CTT GGT GGC TTT CCT GGT CCT CAG GGA CCG TAC TAT TGT GCG GTC GGT GCA GAC AAA GCC TTT --- GGT CGT GAC 607
         JX306691 CCA TTG GGT TGG CCC CTT GGT GGC TTT CCT GGT CCT CAG GGA CCG TAC TAT TGT GCG GTC GGT GCA GAC AAA GCC TTT --- GGT CGT GAC 279
     BolGLN1.5_C1 CCA TTG GGT TGG CCC CTT GGT GGC TTT CCT GGT CCT CAG GGA CCG TAC TAT TGT GCG GTC GGT GCA GAC AAA GC- --- --- --- --- --- 578
```

Sequence	1 000 … 1 020 … 1 040 … 1 060 … 1 080	
At5g37600_GLN1.1__cds	GTT GTT GAT TCT CAC TAC AAG GCC TGC TTA TAC GCT GGG ATC AAC ATT AGT GGC ATC AAT GGA GAA GTC ATG CCG GGT CAG TGG GAG TTC	600
BnaGLN1.1_C1	ATC GTT GAT GCT CAC TAC AAG GCC TGC TTA TAC GCT GGC ATC AAT ATT AGT GGC ATC AAC GGA GAA GTC ATG CCT GGT CAG TGG GAG TTC	688
X82997	ATC GTT GAT GCT CAC TAC AAG GCC TGC TTA TAC GCT GGC ATC AAT ATT AGT GGC ATC AAC GGA GAA GTC ATG CCT GGT CAG TGG GAG TTC	661
Bra028132_cds	ATC GTT GAT GCT CAC TAC AAG GCC TGC TTA TAC GCT GGC ATC AAC ATT AGT GGC ATC AAC GGA GAA GTC ATG CCT GGT CAG TGG GAG TTC	600
BraGLN1.1_C1	ATC GTT GAT GCT CAC TAC AAG GCC TGC TTA TAC GCT GGC ATC AAC ATT AGT GGC ATC AAC GGA GAA GTC ATG CCT GGT CAG TGG GAG TTC	691
BnaGLN1.1_C2	ATT GTT GAT GCT CAT TAC AAG GCC TGC TTA TAC GCT GGC ATC ACC ATT AGT GGC ATC AAT GGA GAA GTC ATG CCG GGT CAG TGG GAG TTC	688
Y12460	ATT GTT GAT GCT CAT TAC AAG GCC TGC TTA TAC GCT GGC ATC ACC ATT AGT GGC ATC AAT GGA GAA GTC ATG CCG GGT CAG TGG GAG TTC	579
BolGLN1.1_C1	ATT GTT GAT GCT CAT TAC AAG GCC TGC TTA TAC GCT GGC ATC ACC ATT AGT GGC ATC AAT GGA GAA GTC ATG CCG GGT CAG TGG GAG TTC	682
At1g66200.1_GLN1.2__cds	ATT GTT GAT GCT CAC TAC AAA GCC TCT TTG TAT GCT GGA ATC AAC ATC AGT GGG ATC AAT GGA GAA GTC ATG CCG GGA CAA TGG GAG TTC	600
BnaGLN1.2_C1	ATT GTT GAT GCT CAC TAC AAG GCT TGT TTG TAT GCT GGA ATT AAC ATC AGT GGA ATC AAT GGA GAA GTC ATG CCT GGT CAG TGG GAG TTC	686
X76736	ATT GTT GAT GCT CAC TAC AAG GCT TGT TTG TAT GCT GGA ATT AAC ATC AGT GGA ATC AAT GGA GAA GTC ATG CCT GGT CAG TGG GAG TTC	667
Bra039756_cds	ATT GTT GAT GCT CAC TAC AAG GCT TGT TTG TAT GCT GGA ATT AAC ATC AGT GGA ATC AAT GGA GAA GTC ATG CCT GGT CAG TGG GAG TTC	600
BraGLN1.2_C1	ATT GTT GAT GCT CAC TAC AAG GCT TGT TTG TAT GCT GGA ATT AAC ATC AGT GGA ATC AAT GGA GAA GTC ATG CCT GGT CAG TGG GAG TTC	682
EU499383	ATT GTT GAT GCT CAC TAC AAG GCT TGT TTG TAT GCT GGA ATT AAC ATC AGT GGA ATC AAT GGA GAA GTC ATG CCT GGT CAG TGG GAG TTC	684
AY773089	ATT GTT GAT GCT CAC TAC AAG GCT TGT TTG TAT GCT GGA ATT AAC ATC AGT GGA ATC AAT GGA GAA GTC ATG CCT GGT CAG TGG GAG TTC	688
BnaGLN1.2_C2	ATT GTT GAT GCT CAC TAC AAG GCT TGT TTG TAT GCC GGA ATT AAC ATC AGT GGA ATC AAT GGA GAA GTC ATG CCT GGT CAG TGG GAG TTC	689
Y12459	ATT GTT GAT GCT CAC TAC AAG GCT TGT TTG TAT GCC GGA ATT AAC ATC AGT GGA ATC AAT GGA GAA GTC ATG CCT GGT CAG TGG GAG TTC	631
BolGLN1.2_C1	ATT GTT GAT GCT CAC TAC AAG GCT TGT TTG TAT GCC GGA ATT AAC ATC AGT GGA ATC AAT GGA GAA GTC ATG CCT GGT CAG TGG GAG TTC	579
EU822334	ATT GTT GAT GCT CAC TAC AAG GCT TGT TTG TAT GCC GGA ATT AAC ATC AGT GGA ATC AAT GGA GAA GTC ATG CCT GGT CAG TGG GAG TTC	628
EU822335	ATT GTT GAT GCT CAC TAC AAG GCT TGT TTG TAT GCC GGA ATT AAC ATC AGT GGA ATC AAT GGA GAA GTC ATG CCT GGT CAG TGG GAG TTC	625
At3g17820_GLN1.3__cds	ATT GTG GAT GCT CAC TAC AAG GCC TGT CTT TAC GCC GGT ATT GGT ATT TCT GGT ATC AAT GGA GAA GTC ATG CCA GGC CAG TGG GAG TTC	600
BnaGLN1.3_C2	ATT GTG GAT GCA CAC TAC AAG GCC TGT ATT TAC GCA GGT ATT GGC ATC TCT GGT GTC AAT GGA GAA GTC ATG CCT GGT CAG TGG GAG TTC	747
JX306693	ATT GTG GAT GCA CAC TAC AAG GCC TGT ATT TAC GCA GGT ATT GGC ATC TCT GGT GTC AAT GGA GAA GTC ATG CCT GGT CAG TGG GAG TTC	615
Bra022247_cds	ATT GTG GAT GCA CAC TAC AAG GCC TGT ATT TAC GCA GGT ATT GGC ATC TCT GGT GTC AAT GGA GAA GTC ATG CCT GGT CAG TGG GAG TTC	600
BraGLN1.3_C1	ATC GTG GAT GCA CAC TAC AAG GCC TGT ATT TAC GCA GGT ATT GGC ATC TCT GGT GTC AAT GGA GAA GTC ATG CCT GGT CAG TGG GAG TTC	670
BnaGLN1.3_C1	ATC GTG GAT GCG CAC TAC AAG GCC TGT ATT TAC GCA GGT ATT GGC ATC TCT GGT GTC AAT GGA GAA GTC ATG CCT GGC CAG TGG GAG TTC	667
JX306690	ATC GTG GAT GCG CAC TAC AAG GCC TGT ATT TAC GCA GGT ATT GGC ATC TCT GGT GTC AAT GGA GAA GTC ATG CCT GGC CAG TGG GAG TTC	634
BolGLN1.3_C1	ATC GTG GAT GCG CAC TAC AAG GCC TGT ATT TAC GCA GGT ATT GGC ATC TCT GGT GTC AAT GGA GAA GTC ATG CCT GGC CAG TGG GAG TTC	642
BnaGLN1.3_C4	ATC GTG GAT GCA CAC TAC AAA GCC TGT CTT TAC GCA GGT ATT AGC ATC TCT GGT GTC AAT GGA GAA GTC ATG CCT GGC CAG TGG GAG TTC	669
Bra021276_cds	ATT GTG GAT GCA CAC TAC AAA GCC TGT CTT TAC GCA GGT ATT AGC ATC TCT GGT GTC AAT GGA GAA GTC ATG CCT GGC CAG TGG GAG TTC	600
BraGLN1.3_C3	ATT GTG GAT GCA CAC TAC AAA GCC TGT CTT TAC GCA GGT ATT AGC ATC TCT GGT GTC AAT GGA GAA GTC ATG CCT GGC CAG TGG GAG TTC	554
BnaGLN1.3_C3	ATC GTG GAT GCA CAC TAC AAA GCC TGT CTT TAC GCA GGT ATT AGC ATC TCT GGT GTC AAT GGA GAA GTC ATG CCT GGC CAG TGG GAG TTC	627
BnaGLN1.3_C5	ATT GTG GAC GCA CAC TAC AAG GCC TGT CTT TAC GCA GGT ATT GGC ATC TCT GGT GTC AAT GGA GAG GTC ATG CCT GGA CAG TGG GAG TTC	681
JX306694	ATT GTG GAC GCA CAC TAC AAG GCC TGT CTT TAC GCA GGT ATT GGC ATC TCT GGT GTC AAT GGA GAG GTC ATG CCT GGA CAG TGG GAG TTC	656
Bra001686_cds	ATT GTG GAC GCA CAC TAC AAG GCC TGT CTT TAC GCA GGT ATT GGC ATC TCT GGT GTC AAT GGA GAG GTC ATG CCT GGA CAG TGG GAG TTC	600
BraGLN1.3_C2	ATT GTG GAC GCA CAC TAC AAG GCC TGT CTT TAC GCA GGT ATT GGC ATC TCT GGT GTC AAT GGA GAG GTC ATG CCT GGA CAG TGG GAG TTC	763
BnaGLN1.3_C6	ATT GTG GAC GCA CAC TAC AAG GCC TGT CTT TAC GCA GGT ATT GGC ATC TCT GGT GTC AAT GGA GAG GTC ATG CCT GGA CAG TGG GAG TTC	654
BolGLN1.3_C2	ATT GTG GAC GCA CAC TAC AAG GCC TGT CTT TAC GCA GGT ATT GGC ATC TCT GGT GTC AAT GGA GAG GTC ATG CCT GGA CAG TGG GAG TTC	546
At5g16570_GLN1.4__cds	ATC GTT GAT TCT CAT TAC AAA GCT TGT CTT TAC GCC GGA ATC AAT GTC AGT GGG ACT AAC GGC GAA GTT ATG CCT GGC CAG TGG GAG TTC	600
BnaGLN1.4_C1	ATC GTA GAT GCT CAT TAC AAA GCA TGT CTT TAC GCC GGA ATC AAT GTC AGT GGC ACT AAC GGA GAA GTC ATG CCC GGC CAG TGG GAG TTC	642
JX306697	ATC GTA GAT GCT CAT TAC AAA GCA TGT CTT TAC GCC GGA ATC AAT GTC AGT GGC ACT AAC GGA GAA GTC ATG CCC GGC CAG TGG GAG TTC	617
JX306692	ATC GTA GAT GCT CAT TAC AAA GCA TGT CTT TAC GCC GGA ATC AAT GTC AGT GGC ACT AAC GGA GAA GTC ATG CCC GGC CAG TGG GAG TTC	498
Bra023573_cds	ATC GTA GAT GCT CAT TAC AAA GCA TGT CTT TAC GCC GGA ATC AAT GTC AGT GGC ACT AAC GGA GAA GTC ATG CCC GGC CAG TGG GAG TTC	597
BraGLN1.4_C1	ATC GTA GAT GCT CAT TAC AAA GCA TGT CTT TAC GCC GGA ATC AAT GTC AGT GGC ACT AAC GGA GAA GTC ATG CCC GGC CAG TGG GAG TTC	722
BnaGLN1.4_C2	ATC GTT GAT GCT CAT TAC AAA GCA TGT CTT TAC GCT GGA ATC AAT GTC AGT GGC ACT AAC GGA GAA GTC ATG CCC GGA CAG TGG GAA TTC	667
JX306695	ATC GTT GAT GCT CAT TAC AAA GCA TGT CTT TAC GCT GGA ATC AAT GTC AGT GGC ACT AAC GGA GAA GTC ATG CCC GGA CAG TGG GAA TTC	641
JX306696	ATC GTA GAT GCT CAT TAC AAA GCA TGT CTT TAC GCC GGA ATC AAT GTC AGT GGC ACT AAC GGA GAA GTC ATG CCC GGC CAG TGG GAG TTC	618
BolGLN1.4_C1	ATC GTT GAT GCT CAT TAC AAA GCA TGT CTT TAC GCT GGA ATC AAT GTC AGT GGC ACT AAC GGA GAA GTC ATG CCC GGA CAG TGG GAA TTC	642
BnaGLN1.4_C4	ATA GTG GAT TCT CAT TAC AAA GCC TGT CTT TAC GCC GGA ATC AAT GTC AGT GGC ACT AAC GGA GAA GTC ATG CCC GGA CAG TGG GAG TTC	603
JX306700	ATA GTG GAT TCT CAT TAC AAA GCC TGT CTT TAC GCC GGA ATC AAT GTC AGT GGC ACT AAC GGA GAA GTC ATG CCC GGA CAG TGG GAG TTC	279
JX306701	ATA GTG GAT TCT CAT TAC AAA GCC TGT CTT TAC GCC GGA ATC AAT GTC AGT GGC ACT AAC GGA GAA GTC ATG CCC GGA CAG TGG GAG TTC	279

```
Bra008612_cds        ATA GTA GAT TCT CAT TAC AAA GCC TGT CTT TAC GCC GGA ATC AAT GTC AGT GGC ACT AAC GGA GAA GTC ATG CCC GGA CAG TGG GAG TTC 600
BraGLN1.4_C2         ATA GTA GAT TCT CAT TAC AAA GCC TGT CTT TAC GCC GGA ATC AAT GTC AGT GGC ACT AAC GGA GAA GTC ATG CCC GGA CAG TGG GAG TTC 645
BnaGLN1.4_C3         ATA GTA GAT TCT CAT TAC AAA GCC TGT CTT TAC GCC GGA ATC AAT GTC AGT GGC ACT AAC GGA GAA GTC ATG CCC GGA CAG TGG GAG TTC 644
JX306698             ATA GTA GAT TCT CAT TAC AAA GCC TGT CTT TAC GCC GGA ATC AAT GTC AGT GGC ACT AAC GGA GAA GTC ATG CCC GGA CAG TGG GAG TTC 305
JX306699             ATA GTA GAT TCT CAT TAC AAA GCC TGT CTT TAC GCC GGA ATC AAT GTC AGT GGC ACT AAC GGA GAA GTC ATG CCC GGA CAG TGG GAG TTC 305
BolGln1.4_C2         ATA GTA GAT TCT CAT TAC AAA GCC TGT CTT TAC GCC GGA ATC AAT GTC AGT GGC ACT AAC GGA GAA GTC ATG CCC GGA CAG TGG GAG TTC 604
At1g48470_GLN1.5__cds ATT GTC GAT GCT CAC TAT AAA GCT TGT CTA TAC TCC GGT TTG AGT ATT GGT GGT GCC AAT GGT GAA GTC ATG CCT GGA CAA TGG GAG TTT 600
BnaGLN1.5_C2         ATT GTG GAT GGT CAC TAC AAA GCT TGT CTT TAC GCT GGT TTA AGC ATA GGT GGT GCC AAT GGT GAA GTC ATG CCT GGT CAA TGG GAG TTT 722
Bra018729_cds        ATT GTG GAT GGT CAC TAC AAA GCT TGT CTT TAC GCT GGT TTA AGC ATA GGT GGT GCC AAT GGT GAA GTC ATG CCT GGT CAA TGG GAG TTT 571
BraGLN1.5_C1         ATT GTG GAT GGT CAC TAC AAA GCT TGT CTT TAC GCT GGT TTA AGC ATA GGT GGT GCC AAT GGT GAA GTC ATG CCT GGT CAA TGG GAG TTT 635
BnaGLN1.5_C1         ATT GTG GAT GGT CAC TAC AAA GCT TGT CTT TAC GCT GGT TTA AGC ATA GGT GGT GCC AAT GGT GAA GTC ATG CCT GGT CAA TGG GAG TTT 697
JX306691             ATT GTG GAT GGT CAC TAC AAA GCT TGT CTT TAC GCT GGT TTA AGC ATA GGT GGT GCC AAT GGT GAA GTC ATG CCT GGT CAA TGG GAG TTT 369
BolGLN1.5_C1         --- --- --- --- --- --- --- --- --- --- --- --- --- --- --- --- --- --- --- --- --- --- --- --- --- --- --- --- --- --- 578
```

BnaGLN1_JXB

```
                                                  1 100                         1 120                         1 140                         1 160
At5g37600_GLN1.1__cds  CAG GTC GGT CCA GCT GTT GGT ATC TCG GCT GCT GAT GAA ATT TGG GTC GCT CGT TAC ATT TTG GAG AGG ATC ACA GAG ATT GCT GGT GTA 690
BnaGLN1.1_C1           CAA GTT GGT CCA GCT GTT GGT ATC TCG GCC GGT GAT GAA ATT TGG GTC GCA CGT TTC ATT TTG GAG AGG ATC ACA GAG ATT GCT GGT GTG 778
X82997                 CAA GTT GGT CCA GCT GTT GGT ATC TCG GCC GGT GAT GAA ATT TGG GTC GCA CGT TTC ATT TTG GAG AGG ATC ACA GAG ATT GCT GGT GTG 751
Bra028132_cds          CAA GTT GGT CCA GCT GTT GGT ATC TCG GCC GGT GAT GAA ATT TTG GTC GCA CGT TTC ATT TTG GAG AGG ATC ACA GAG ATT GCT GGT GTG 690
BraGLN1.1_C1           CAA GTT GGT CCA GCT GTT GGT ATC TCG GCC GGT GAT GAA ATT TTG GTC GCA CGT TTC ATT TTG GAG AGG ATC ACA GAG ATT GCT GGT GTG 781
BnaGLN1.1_C2           CAA GTT GGT CCA GCT GTT GGT ATC TCG GCC GGT GAT GAA ATT TGG GTC GCA CGT TAC ATT TTG GAG AGG ATC ACA GAG GTT GCT GGT GTG 778
Y12460                 CAA GTT GGT CCA GCT GTT GGT ATC TCG GCC GGT GAT GAA ATT TGG GTC ACA CGT TAC ATT TTG GAG AGG ATC ACA GAG GTT GCT GGT GTG 669
BolGLN1.1_C1           CAA GTT GGT CCA GCT GTT GGT ATC TCG GCC GGT GAT GAA ATT TGG GTC GCA CGT TAC ATT TTG GAG AGG ATC ACA GAG GTT GCT GGT GTG 772
At1g66200.1_GLN1.2__cds CAA GTC GGC CCA TCG GTC GGT ATC TCA GCT GCT GAT GAA ATA TGG ATC GCT CGT TAC ATT TTG GAG AGG ATC ACA GAG ATT GCT GGT GTG 690
BnaGLN1.2_C1           CAA GTC GGA CCG TCG GTT GGT ATC TCA GCT GCT GAT GAA GTG TGG ATT GCT CGT TTT ATT TTG GAG AGG ATC ACA GAG ATT GCT GGT GTG 776
X76736                 CAA GTC GGA CCG TCG GTT GGT ATC TCA GCT GCT GAT GAA GTG TGG ATT GCT CGT TTT ATT TTG GAG AGG ATC ACA GAG ATT GCT GGT GTG 757
Bra039756_cds          CAG GTC GGA CCA TCG GTT GGT ATC TCA GCT GCT GAT GAA GTG TGG ATT GCT CGT TTT ATT TTG GAG AGG ATC ACA GAG ATT GCT GGT GTG 690
BraGLN1.2_C1           CAG GTC GGA CCA TCG GTT GGT ATC TCA GCT GCT GAT GAA GTG TGG ATT GCT CGT TTT ATT TTG GAG AGG ATC ACA GAG ATT GCT GGT GTG 772
EU499383               CAG GTC GGA CCA TCG GTT GGT ATC TCA GCT GCT GAT GAA GTG TGG ATT GCT CGT TTT ATT TTG GAG AGG ATC ACA GAG ATT GCT GGT GTG 774
AY773089               CAG GTC GGA CCA TCG GTT GGT ATC TCA GCT GCT GAT GAA GTG TGG ATT GCT CGT TTT ATT TTG GAG AGG ATC ACA GAG ATT GCT GGT GTG 778
BnaGLN1.2_C2           CAA GTC GGA CCA TCG GTT GGT ATC TCA GCT GCT GAT GAA GTG TGG ATT GCT CGT TAC ATT TTG GAG AGG ATC ACA GAG ATT GCT GGT GTG 779
Y12459                 CAA GTC GGA CCA TCG GTT GGT ATC TCA GCT GCT GAT GAA GTG TGG ATT GCT CGT TAC ATT TTG GAG AGG ATC ACA GAG ATT GCT GGT GTG 721
BolGLN1.2_C1           CAA GTC GGA CCA TCG GTT GGT ATC TCA GCT GCT GAT GAA GTG TGG ATT GCT CGT TAC ATT TTG GAG AGG ATC ACA GAG ATT GCT GGT GTG 669
EU822334               CAA GTC GGA CCA TCG GTT GGT ATC TCA GCT GCT GAT GAA GTG TGG ATT GCT CGT TAC ATT TTG GAG AGG ATC ACA GAG ATT GCT GGT GTG 718
EU822335               CAA GTC GGA CCA TCG GTT GGT ATC TCA GCT GCT GAT GAA GTG TGG ATT GCT CGT TAC ATT TTG GAG AGG ATC ACA GAG ATT GCT GGT GTG 715
At3g17820_GLN1.3__cds  CAA GTC GGC CCT GTT GAG GGT ATT AGT TCT GGT GAT CAA GTC TGG GTT GCT CGA TAC CTT CTC GAG AGG ATC ACT GAG ATC TCT GGT GTA 690
BnaGLN1.3_C2           CAA GTC GGT CCT GTT GAG GGT ATT AGT GCT GGT GAC CAA GTT TGG GTC GCT AGA TAC CTT CTC GAG AGG ATC ACT GAG ATC TCT GGT GTG 837
JX306693               CAA GTC GGT CCT GTT GAG GGT ATT AGT GCT GGT GAC CAA GTT TGG GTC GCT AGA TAC CTT CTC GAG AGG ATC ACT GAG ATC TCT GGT GTG 705
Bra022247_cds          CAA GTC GGT CCT GTT GAG GGT ATT AGT GCT GGT GAC CAA GTC TGG GTC GCT AGA TAC CTT CTC GAG AGG ATC ACT GAG ATC TCT GGT GTG 690
BraGLN1.3_C1           CAA GTC GGT CCT GTT GAG GGT ATT AGT GCT GGT GAC CAA GTC TGG GTC GCT AGA TAC CTT CTC GAG AGG ATC ACT GAG ATC TCT GGT GTG 760
BnaGLN1.3_C1           CAA GTC GGT CCT GTT GAG GGT ATT AGT GCT GGT GAC CAA GTC TGG GTC GCT AGA TAC CTT CTT GAG AGG ATC ACC GAG ATC TCT GGT GTC 757
JX306690               CAA GTC GGT CCT GTT GAG GGT ATT AGT GCT GGT GAC CAA GTC TGG GTC GCT AGA TAC CTT CTT GAG AGG ATC ACC GAG ATC TCT GGT GTC 724
BolGLN1.3_C1           CAA GTC GGT CCT GTT GAG GGT ATT AGT GCT GGT GAC CAA GTC TGG GTC GCT AGA TAC CTT CTT GAG AGG ATC ACC GAG ATC TCT GGT GTC 732
BnaGLN1.3_C4           CAA GTC GGT CCT GTT GAA GGG ATT AGT GCC GGT GAT CAA GTC TGG ATA GCT AGA TTC CTT CTC GAG AGG ATC ACT GAG ATC TCT GGT GTA 759
Bra021276_cds          CAA GTC GGT CCT GTT GAA GGG ATT AGT GCC GGT GAT CAA GTC TGG ATA GCT AGA TTC CTT CTC GAG AGG ATC ACT GAG ATC TCT GGT GTA 690
BraGLN1.3_C3           CAA GTC GGT CCT GTT GAA GGG ATT AGT GCC GGT GAT CAA GTC TGG ATA GCT AGA TTC CTT CTC GAG AGG ATC ACT GAG ATC TCT GGT GTA 644
BnaGLN1.3_C3           CAA GTC GGT CCT GTT GAA GGT ATT AGT GCT GGT GAT CAA GTC TGG ATC GCT AGA TTC CTT CTC GAG AGG ATC ACT GAG ATC TCT GGT GTA 717
BnaGLN1.3_C5           CAA GTC GGT CCA GTT GAG GGT ATT AGT TCT GGT GAT CAA GTC TGG GTC GCT AGA TAC CTT CTT GAG AGG ATC ACT GAG ATC TCT GGT GTA 771
JX306694               CAA GTC GGT CCA GTT GAG GGT ATT AGT TCT GGT GAT CAA GTC TGG GTC GCT AGA TAC CTT CTT GAG AGG ATC ACT GAG ATC TCT GGT GTA 746
Bra001686_cds          CAA GTC GGT CCA GTT GAG GGT ATT AGT TCT GGT GAT CAA GTC TGG GTC GCT AGA TAC CTT CTT GAG AGG ATC ACT GAG ATC TCT GGT GTA 690
BraGLN1.3_C2           CAA GTC GGT CCA GTT GAG GGT ATT AGT TCT GGT GAT CAA GTC TGG GTC GCT AGA TAC CTT CTT GAG AGG ATC ACT GAG ATC TCT GGT GTA 853
BnaGLN1.3_C6           CAA GTC GGT CCA GTT GAG GGT ATT AGT TCT GGT GAT CAA GTC TGG GTC GCT AGA TAC CTT CTT GAG AGG ATC ACT GAG ATC TCT GGT GTA 744
BolGLN1.3_C2           CAA GTC GGT CCA GTT GAG GGT ATT AGT TCT GGT GAT CAA GTC TGG GTT GCT AGA TAC CTT CTT GAG AGG ATC ACC GAG ATC TCT GGT GTA 636
At5g16570_GLN1.4__cds  CAA GTC GGT CCC ACC GTT GGA ATC GCT GCC GCC GAT CAG GTC TGG GTT GCT CGT TAC ATT CTT GAG AGG ATC ACA GAA TTG GCT GGA GTT 690
BnaGLN1.4_C1           CAA GTC GGT CCA ACC GTT GGA ATA GCT GCC GCT GAT CAG GTC TGG GTC GCT CGT TAC ATC CTC GAG AGG ATC ACA GAA TTG GCT GGA GTG 732
JX306697               CAA GTC GGT CCA ACC GTT GGA ATA GCT GCC GCT GAT CAG GTC TGG GTC GCT CGT TAC ATC CTC GAG AGG ATC ACA GAA TTG GCT GGA GTG 707
JX306692               CAA GTC GGT CCA ACC GTT GGA ATA GCT GCC GCT GAT CAG GTC TGG GTC GCT CGT TAC ATC CTC GAG AGG ATC ACA GAA TTG GCT GGA GTG 588
Bra023573_cds          CAA GTC GGT CCA ACC GTT GGA ATA GCT GCC GCT GAT CAG GTC TGG GTC GCT CGT TAC ATC CTC GAG AGG ATC ACA GAA TTG GCT GGA GTG 687
BraGLN1.4_C1           CAA GTC GGT CCA ACC GTT GGA ATA GCT GCC GCT GAT CAG GTC TGG GTC GCT CGT TAC ATC CTC GAG AGG ATC ACA GAA TTG GCT GGA GTG 812
BnaGLN1.4_C2           CAA GTC GGT CCA ACC GTT GGA ATA GCT GCA GCC GAT CAG GTC TGG GTC GCT CGT TAC ATC CTC GAG AGG ATC ACA GAA CTG GCT GGA GTT 757
JX306695               CAA GTC GGT CCA ACC GTT GGA ATA GCT GCA GCC GAT CAG GTC TGG GTC GCT CGT TAC ATC CTC GAG AGG ATC ACA GAA CTG GCT GGA GTT 731
JX306696               CAA GTC GGT CCA ACC GTT GGA ATA GCT GCC GCT GAT CAG GTC TGG GTC GCT CGT TAC ATC CTC GAG AGG ATC ACA GAA TTG GCT GGA GTG 708
BolGLN1.4_C1           CAA GTC GGT CCA ACC GTT GGA ATA GCT GCA GCC GAT CAG GTC TGG GTC GCT CGT TAC ATC CTC GAG AGG ATC ACA GAA CTG GCT GGA GTT 732
BnaGLN1.4_C4           CAA GTC GGT CCA ACC GTT GGA ATC GCT GCC GCC GAT CAG GTC TGG GTC GCT CGT TAC ATC CTC GAG AGG ATC ACA GAA TTG GCT GGA GTT 693
JX306700               CAA GTC GGT CCA ACC GTT GGA ATC GCT GCC GCC GAT CAG GTC TGG GTC GCT CGT TAC ATC CTC GAG AGG ATC ACA GAA TTG GCT GGA GTT 369
JX306701               CAA GTC GGT CCA ACC GTT GGA ATC GCT GCC GCC GAT CAG GTC TGG GTC GCT CGT TAC ATC CTC GAG AGG ATC ACA GAA TTG GCT GGA GTT 369
```

```
    Bra008612_cds CAA GTC GGT CCA ACC GTT GGA ATC GCT GCC GCC GAT CAG GTC TGG GTC GCT CGT TAC ATC CTC GAG AGG ATC ACA GAA TTG GCT GGA GTT 690
     BraGLN1.4_C2 CAA GTC GGT CCA ACC GTT GGA ATC GCT GCC GCC GAT CAG GTC TGG GTC GCT CGT TAC ATC CTC GAG AGG ATC ACA GAA TTG GCT GGA GTT 735
     BnaGLN1.4_C3 CAA GTC GGT CCA ACG GTT GGA ATC GCT GCC GCC GAT CAA GTC TGG GTC GCT CGT TAC ATC CTC GAG AGG ATC ACA GAA TTG GCT GGA GTT 734
         JX306698 CAA GTC GGT CCA ACG GTT GGA ATC GCT GCC GCC GAT CAA GTC TGG GTC GCT CGT TAC ATC CTC GAG AGG ATC ACA GAA TTG GCT GGA GTT 395
         JX306699 CAA GTC GGT CCA ACG GTT GGA ATC GCT GCC GCC GAT CAA GTC TGG GTC GCT CGT TAC ATC CTC GAG AGG ATC ACA GAA TTG GCT GGA GTT 395
     BolGln1.4_C2 CAA GTC GGT CCA ACG GTT GGA ATC GCT GCC GCC GAT CAA GTC TGG GTC GCT CGT TAC ATC CTC GAG AGG ATC ACA GAA TTG GCT GGA GTT 694
At1g48470_GLN1.5__cds CAA ATC AGT CCT ACT GTT GGT ATT GGT GCA GGT GAT CAA TTA TGG GTT GCT CGT TAC ATT CTT GAG AGG ATT ACT GAG ATA TGC GGT GTG 690
     BnaGLN1.5_C2 CAA ATC AGC CCT ACT GTT GGT ATT GGT GCA GGT GAT CAG TTA TGG GTT GCT CGC TAC ATA CTT GAG AGG ATT ACT GAG ATA TGC GGC GTA 812
    Bra018729_cds CAA ATC AGC CCT ACT GTT GGT ATT GGT GCA GGT GAT CAG TTA TGG GTT GCT CGC TAC AT- --- --- AGG ATT ACT GAG ATA TGC GGC GTA 654
     BraGLN1.5_C1 CAA ATC AGC CCT ACT GTT GGT ATT GGT GCA GGT GAT CAG TTA TGG GTT GCT CGC TAC ATA CTC GAG AGG ATT ACT GAG ATA TGC GGC GTA 725
     BnaGLN1.5_C1 CAA ATC AGC CCT ACT GTT GGT ATT GGT GCA GGT GAT CAG TTA TGG GTT GCT CGC TAC ATA CTC GAG AGG ATT ACT GAG ATA TGC GGC GTG 787
         JX306691 CAA ATC AGC CCT ACT GTT GGT ATT GGT GCA GGT GAT CAG TTA TGG GTT GCT CGC TAC ATA CTC GAG AGG ATT ACT GAG ATA TGC GGC GTG 459
     BolGLN1.5_C1 --- --- --- --- --- --- --- --- --- --- --- --- --- --- --- --- --- --- --- --- --- --- --- --- --- --- --- --- --- --- 578
```

BnaGLN1_JXB

1 180 | 1 200 | 1 220 | 1 240 | 1 260

At5g37600_GLN1.1__cds	GTG	GTA	TCT	TTT	GAC	CCG	AAA	CCG	ATT	CCC	GGT	GAC	TGG	AAC	GGT	GCT	GGT	GCT	CAC	TGC	AAC	TAC	AGT	ACC	AAG	TCA	ATG	AGG	GAA	GAA	780
BnaGLN1.1_C1	GTG	GTA	TCT	TTT	GAC	CCA	AAA	CCG	ATT	CCC	GGT	GAC	TGG	AAT	GGT	GCT	GGT	GCT	CAC	TGC	AAC	TAT	AGT	ACC	AAG	TCA	ATG	AGG	GAA	GAT	868
X82997	GTG	GTA	TCT	TTT	GAC	CCA	AAA	CCG	ATT	CCC	GGT	GAC	TGG	AAT	GGT	GCT	GGT	GCT	CAC	TGC	AAC	TAT	AGT	ACC	AAG	TCA	ATG	AGG	GAA	GAT	841
Bra028132_cds	GTG	GTA	TCT	TTT	GAC	CCA	AAA	CCG	ATT	CCC	GGT	GAC	TGG	AAT	GGT	GCT	GGT	GCT	CAC	TGC	AAC	TAT	AGT	ACC	AAG	TCA	ATG	AGG	GAA	GAT	780
BraGLN1.1_C1	GTG	GTA	TCT	TTT	GAC	CCA	AAA	CCG	ATT	CCC	GGT	GAC	TGG	AAT	GGT	GCT	GGT	GCT	CAC	TGC	AAC	TAT	AGT	ACC	AAG	TCA	ATG	AGG	GAA	GAT	871
BnaGLN1.1_C2	GTG	GTA	TCT	TTT	GAC	CCA	AAA	CCT	ATT	CCC	GGT	GAC	TGG	AAT	GGT	GCT	GGT	GCT	CAC	TGC	AAC	TAT	AGT	ACC	AAG	TCA	ATG	AGG	GAA	GAT	868
Y12460	GTG	GTA	TCT	TTT	GAC	CCA	AAA	CCT	ATT	CCC	GGT	GAC	TGG	AAT	GGT	GCT	GGT	GCT	CAT	TGC	AAC	TAT	AGT	ACC	AAG	TCA	ATG	AGG	GAA	GAT	759
BolGLN1.1_C1	GTG	GTA	TCT	TTT	GAC	CCA	AAA	CCT	ATT	CCC	GGT	GAC	TGG	AAT	GGT	GCT	GGT	GCT	CAC	TGC	AAC	TAT	AGT	ACC	AAG	TCA	ATG	AGG	GAA	GAT	862
At1g66200.1_GLN1.2__cds	GTT	GTA	TCT	TTT	GAC	CCA	AAA	CCT	ATT	CCT	GGT	GAC	TGG	AAT	GGA	GCT	GGT	GCT	CAC	ACC	AAT	TAC	AGT	ACT	AAA	TCA	ATG	AGG	GAA	GAA	780
BnaGLN1.2_C1	GTT	GTA	TCT	TTT	GAC	CCA	AAA	CCA	ATT	CCG	GGT	GAC	TGG	AAC	GGA	GCT	GGT	GCT	CAC	ACC	AAT	TAC	AGT	ACT	AAA	TCG	ATG	AGG	GAG	GAA	866
X76736	GTT	GTA	TCT	TTT	GAC	CCA	AAA	CCA	ATT	CCG	GGT	GAC	TGG	AAC	GGA	GCT	GGT	GCT	CAC	ACC	AAT	TAC	AGT	ACT	AAA	TCG	ATG	AGG	GAG	GAA	847
Bra039756_cds	GTT	GTA	TCT	TTT	GAC	CCG	AAA	CCA	ATT	CCG	GGT	GAC	TGG	AAC	GGA	GCT	GGT	GCT	CAC	ACC	AAT	TAC	AGT	ACT	AAA	TCG	ATG	AGG	GAG	GAA	780
BraGLN1.2_C1	GTT	GTA	TCT	TTT	GAC	CCG	AAA	CCA	ATT	CCG	GGT	GAC	TGG	AAC	GGA	GCT	GGT	GCT	CAC	ACC	AAT	TAC	AGT	ACT	AAA	TCG	ATG	AGG	GAG	GAA	862
EU499383	ATT	GTA	TCT	TTT	GAC	CCA	AAA	CCA	ATT	CCG	GGT	GAC	TGG	AAC	GGA	GCT	GGT	GCT	CAC	ACC	AAT	TAC	AGT	ACT	AAA	TCG	ATG	AGG	GAG	GAA	864
AY773089	GTT	GTA	TCT	TTT	GAC	CCG	AAA	CCA	ATT	CCG	GGT	GAC	TGG	AAC	GGA	GCT	GGT	GCT	CAC	ACC	AAT	TAC	AGT	ACT	AAA	TCG	ATG	AGG	GAG	GAA	868
BnaGLN1.2_C2	GTT	GTA	TCT	TTT	GAC	CCA	AAA	CCA	ATT	CCG	GGT	GAC	TGG	AAT	GGA	GCT	GGT	GCT	CAC	ACC	AAT	TAC	AGT	ACT	AAA	TCG	ATG	AGG	GAG	GAA	869
Y12459	GTT	GTA	TCT	TTT	GAC	CCA	AAA	CCA	ATT	CCG	GGT	GAC	TGG	AAC	GGA	GCT	GGT	GCT	CAC	ACC	AAT	TAC	AGT	ACT	AAA	TCG	ATG	AGG	GAG	GAA	811
BolGLN1.2_C1	GTT	GTA	TCT	TTT	GAC	CCA	AAA	CCA	ATT	CCG	GGT	GAC	TGG	AAC	GGA	GCT	GGT	GCT	CAC	ACC	AAT	TAC	AGT	ACT	AAA	TCG	ATG	AGG	GAG	GAA	759
EU822334	GTT	GTA	TCT	TTT	GAC	CCA	AAA	CCA	ATT	CCG	GGY	GAC	TGG	AAC	GGA	GCT	GGT	GCT	CAC	ACC	AAT	TAC	AGT	ACT	AAA	TCG	ATG	AGG	GAG	GAA	808
EU822335	GTT	GTA	TCT	TCT	GAC	CCA	AAA	CCA	ATT	CCG	GGT	GAC	TGG	AAC	GGA	GCT	GGT	GCT	CAC	ACC	AAT	TAC	AGT	ACT	AAA	TCG	ATG	AGG	GAG	GAA	805
At3g17820_GLN1.3__cds	ATT	GTC	AGC	TTC	GAC	CCG	AAA	CCA	GTC	CCG	GGT	GAC	TGG	AAT	GGA	GCT	GGA	GCT	CAC	TGC	AAC	TAC	AGC	ACT	AAG	ACA	ATG	AGA	AAC	GAT	780
BnaGLN1.3_C2	AAT	GTC	AGC	TTC	GAC	CCT	AAA	CCA	GTC	CCG	GGT	GAT	TGG	AAC	GGA	GCT	GGA	GCT	CAC	TGC	AAC	TAC	AGC	ACG	AAG	TCA	ATG	AGG	AAC	GAC	927
JX306693	AAT	GTC	AGC	TTC	GAC	CCT	AAA	CCA	GTC	CCG	GGT	GAT	TGG	AAC	GGA	GCT	GGA	GCT	CAC	TGC	AAC	TAC	AGC	ACG	AAG	TCA	ATG	AGG	AAC	GAC	795
Bra022247_cds	AAT	GTC	AGC	TTC	GAC	CCT	AAA	CCA	GTC	CCG	GGT	GAT	TGG	AAC	GGA	GCT	GGA	GCT	CAC	TGC	AAC	TAC	AGC	ACG	AAG	TCA	ATG	AGG	AAC	GAC	780
BraGLN1.3_C1	AAT	GTC	AGC	TTC	GAC	CCT	AAA	CCA	GTC	CCG	GGT	GAT	TGG	AAC	GGA	GCT	GGA	GCT	CAC	TGC	AAC	TAC	AGC	ACG	AAG	TCA	ATG	AGG	AAC	GAC	850
BnaGLN1.3_C1	AAT	GTC	AGC	TTC	GAC	CCT	AAG	CCA	GTC	CCG	GGT	GAT	TGG	AAC	GGA	GCT	GGA	GCT	CAC	TGC	AAC	TAC	AGC	ACG	AAG	TCG	ATG	AGG	AAC	GAC	847
JX306690	AAT	GTC	AGC	TTC	GAC	CCT	AAG	CCA	GTC	CCG	GGT	GAT	TGG	AAC	GGA	GCT	GGA	GCT	CAC	TGC	AAC	TAC	AGC	ACG	AAG	TCG	ATG	AGG	AAC	GAC	814
BolGLN1.3_C1	AAT	GTC	AGC	TTC	GAC	CCT	AAG	CCA	GTC	CCG	GGT	GAT	TGG	AAC	GGA	GCT	GGA	GCT	CAC	TGC	AAC	TAC	AGC	ACG	AAG	TCG	ATG	AGG	AAC	GAC	822
BnaGLN1.3_C4	AAC	GTC	AGC	TTC	GAC	CCA	AAA	CCA	GTC	CCG	GGT	GAT	TGG	AAC	GGA	GCG	GGA	GCG	CAC	TGC	AAC	TAC	AGT	ACG	AAG	ACG	ATG	AGG	AAC	ACT	849
Bra021276_cds	AAC	GTC	AGC	TTC	GAC	CCA	AAA	CCA	GTC	CCG	GGT	GAT	TGG	AAC	GGA	GCG	GGA	GCG	CAC	TGC	AAC	TAC	AGT	ACG	AAG	ACG	ATG	AGG	AAC	ACT	780
BraGLN1.3_C3	AAC	GTC	AGC	TTC	GAC	CCA	AAA	CCA	GTC	CCG	GGT	GAT	TGG	AAC	GGA	GCG	GGA	GCG	CAC	TGC	AAC	TAC	AGT	ACG	AAG	ACG	ATG	AGG	AAC	ACT	734
BnaGLN1.3_C3	AAC	GTC	AGC	TTC	GAC	CCA	AAA	CCA	GTC	CCG	GGT	GAT	TGG	AAC	GGA	GCT	GGA	GCT	CAC	TGT	AAC	TAC	AGC	ACG	AAG	ACG	ATG	AGG	AAC	ACT	807
BnaGLN1.3_C5	AAT	GTC	AGC	TTC	GAC	CCT	AAA	CCA	GTT	CCG	GGT	GAT	TGG	AAC	GGA	GCT	GGA	GCT	CAC	TGC	AAC	TAC	AGT	ACG	AAG	ACG	ATG	AGG	AAC	GAC	861
JX306694	AAT	GTC	AGC	TTC	GAC	CCT	AAA	CCA	GTT	CCG	GGT	GAT	TGG	AAC	GGA	GCT	GGA	GCT	CAC	TGC	AAC	TAC	AGT	ACG	AAG	ACG	ATG	AGG	AAC	GAC	836
Bra001686_cds	AAT	GTC	AGC	TTC	GAC	CCA	AAA	CCA	GTT	CCG	GGT	GAT	TGG	AAC	GGA	GCT	GGA	GCT	CAC	TGC	AAC	TAC	AGT	ACG	AAG	ACG	ATG	AGG	AAC	GAC	780
BraGLN1.3_C2	AAT	GTC	AGC	TTC	GAC	CCA	AAA	CCA	GTT	CCG	GGT	GAT	TGG	AAC	GGA	GCT	GGA	GCT	CAC	TGC	AAC	TAC	AGT	ACG	AAG	ACG	ATG	AGG	AAC	GAC	943
BnaGLN1.3_C6	AAT	GTC	AGC	TTC	GAC	CCA	AAA	CCA	GTC	CCG	GGT	GAT	TGG	AAC	GGA	GCT	GGA	GCT	CAC	TGC	AAC	TAC	AGT	ACG	AAG	ACG	ATG	AGG	AAC	GAC	834
BolGLN1.3_C2	AAT	GTC	AGC	TTC	GAC	CCA	AAA	CCA	GTT	CCG	GGT	GAC	TGG	AAC	GGA	GCT	GGA	GCT	CAC	TGC	AAC	TAC	AGT	ACG	AAG	ACG	ATG	AGG	AAC	GAC	726
At5g16570_GLN1.4__cds	GTT	CTG	TCT	CTA	GAC	CCT	AAA	CCA	ATT	CCG	GGA	GAT	TGG	AAT	GGT	GCA	GGG	GCA	CAC	ACA	AAT	TAC	AGT	ACG	AAG	TCG	ATG	AGA	GAA	GAT	780
BnaGLN1.4_C1	GTT	TTG	TCT	CTT	GAC	CCT	AAA	CCA	ATT	CCG	GGA	GAT	TGG	AAT	GGT	GCA	GGA	GCA	CAC	ACA	AAT	TAC	AGC	ACC	AAG	TCG	ATG	AGG	GAA	GAT	822
JX306697	GTT	TTG	TCT	CTT	GAC	CCT	AAA	CCA	ATT	CCG	GGA	GAT	TGG	AAT	GGT	GCA	GGA	GCA	CAC	ACA	AAT	TAC	AGC	ACC	AAG	TCG	ATG	AGG	GAA	GAT	797
JX306692	GTT	TTG	TCT	CTT	GAC	CCT	AAA	CCA	ATT	CCG	GGA	GAT	TGG	AAT	GGT	GCA	GGA	GCA	CAC	ACA	AAT	TAC	AGC	ACC	AAG	TCG	ATG	AGG	GAA	GAT	678
Bra023573_cds	GTT	TTG	TCC	CTT	GAC	CCT	AAA	CCA	ATT	CCG	GGA	GAT	TGG	AAT	GGT	GCA	GGA	GCA	CAC	ACA	AAT	TAC	AGC	ACC	AAG	TCG	ATG	AGA	GAA	GAT	777
BraGLN1.4_C1	GTT	TTG	TCC	CTT	GAC	CCT	AAA	CCA	ATT	CCG	GGA	GAT	TGG	AAT	GGT	GCA	GGA	GCA	CAC	ACA	AAT	TAC	AGC	ACC	AAG	TCG	ATG	AGA	GAA	GAT	902
BnaGLN1.4_C2	GTT	TTA	TCT	CTT	GAC	CCT	AAA	CCA	ATT	CCG	GGA	GAT	TGG	AAT	GGT	GCA	GGA	GCA	CAC	ACA	AAT	TAC	AGT	ACG	AAG	TCG	ATG	AGA	GAA	GAT	847
JX306695	GTT	TTA	TCT	CTT	GAC	CCT	AAA	CCA	ATT	CCG	GGA	GAT	TGG	AAT	GGT	GCA	GGA	GCA	CAC	ACA	AAT	TAC	AGT	ACG	AAG	TCG	ATG	AGA	GAA	GAT	821
JX306696	GTT	TTG	TCT	CTT	GAC	CCT	AAA	CCA	ATT	CCG	GGA	GAT	TGG	AAT	GGT	GCA	GGA	GCA	CAC	ACA	AAT	TAC	AGC	ACC	AAG	TCG	ATG	AGG	GAA	GAT	798
BolGLN1.4_C1	GTT	TTA	TCT	CTT	GAC	CCT	AAA	CCA	ATT	CCG	GGA	GAT	TGG	AAT	GGT	GCA	GGA	GCA	CAC	ACA	AAT	TAC	AGT	ACG	AAG	TCG	ATG	AGA	GAA	GAT	822
BnaGLN1.4_C4	GTT	CTG	TCT	CTT	GAC	CCA	AAA	CCA	ATT	CCG	GGA	GAT	TGG	AAT	GGT	GCA	GGA	GCA	CAC	ACA	AAT	TAC	AGT	ACA	AAG	TCC	ATG	AGA	GAA	GAT	783
JX306700	GTT	CTG	TCT	CTT	GAC	CCA	AAA	CCA	ATT	CCG	GGA	GAT	TGG	AAT	GGT	GCA	GGA	GCA	CAC	ACA	AAT	TAC	AGT	ACA	AAG	TCC	ATG	AGA	GAA	GAT	459
JX306701	GTT	CTG	TCT	CTT	GAC	CCA	AAA	CCA	ATT	CCG	GGA	GAT	TGG	AAT	GGT	GCA	GGA	GCA	CAC	ACA	AAT	TAC	AGT	ACA	AAG	TCC	ATG	AGA	GAA	GAT	459

Bra008612_cds	GTT CTG TCT CTT GAC CCT AAA CCA ATT CCG GGA GAT TGG AAT GGT GCA GGA GCA CAC ACA AAT TAC AGT ACA AAA TCG ATG AGA GAA GAT	780
BraGLN1.4_C2	GTT CTG TCT CTT GAC CCT AAA CCA ATT CCG GGA GAT TGG AAT GGT GCA GGA GCA CAC ACA AAT TAC AGT ACA AAA TCG ATG AGA GAA GAT	825
BnaGLN1.4_C3	GTT TTA TCT CTT GAC CCT AAA CCA ATT CCG GGA GAT TGG AAT GGT GCA GGA GCG CAC ACA AAT TAC AGT ACA AAG TCG ATG AGA GAA GAT	824
JX306698	GTT TTA TCT CTT GAC CCT AAA CCA ATT CCG GGA GAT TGG AAT GGT GCA GGA GCG CAC ACA AAT TAC AGT ACA AAG TCG ATG AGA GAA GAT	485
JX306699	GTT TTA TCT CTT GAC CCT AAA CCA ATT CCG GGA GAT TGG AAT GGT GCA GGA GCG CAC ACA AAT TAC AGT ACA AAG TCG ATG AGA GAA GAT	485
BolGln1.4_C2	GTT TTA TCT CTT GAC CCT AAA CCA ATT CCG GGA GAT TGG AAT GGT GCA GGA GCG CAC ACA AAT TAC AGT ACA AAG TCG ATG AGA GAA GAT	784
At1g48470_GLN1.5__cds	ATT GTC TCA TTC GAT CCA AAA CCA ATC CAG GGT GAT TGG AAT GGA GCA GCC GCT CAT ACG AAC TTC AGT ACA AAA TCG ATG AGG AAA GAT	780
BnaGLN1.5_C2	ATT GTC TCA TTT GAT CCC AAA CCA ATC GAG GGT GAT TGG AAC GGA GCA GCT GCT CAT ACA AAC TTC AGT ACA AAA TCA ATG AGG AAA GAA	902
Bra018729_cds	ATT GTC TCA TTT GAT CCC AAA --- --- --- GGT GAT TGG AAC GGA GCA GCT GCT CAT ACA AAC TTC AGT ACA AAA TCA ATG AGG AAA GAA	735
BraGLN1.5_C1	ATT GTC TCA TTT GAT CCC AAA CCA ATC GAG GGT GAT TGG AAC GGA GCA GCT GCT CAT ACA AAC TTC AGT ACA AAA TCA ATG AGG AAA GAA	815
BnaGLN1.5_C1	ATT GTC TCA TTT GAT CCC AAA CCA ATC GAG GGT GAT TGG AAC GGA GCA GCT GCT CAT ACA AAC TTC AGT ACA AAA TCA ATG AGG AAA GAA	877
JX306691	ATT GTC TCA TTT GAT CCC AAA CCA ATC GAG GGT GAT TGG AAC GGA GCA GCT GCT CAT ACA AAC TTC AGT ACA AAA TCA ATG AGG AAA GAA	549
BolGLN1.5_C1	--- ---	578

BnaGLN1_JXB

							1 280							1 300						1 320							1 340				
At5g37600_GLN1.1__cds	GGC	GGT	TAC	GAG	ATC	ATC	AAG	AAA	GCA	ATC	GAT	AAA	TTG	GGA	CTG	AGA	CAC	AAA	GAA	CAC	ATT	GCT	GCT	TAC	GGT	GAA	GGC	AAT	GAG	CGT	870
BnaGLN1.1_C1	GGT	GGT	TAC	GAG	ATT	ATC	AAG	AAG	GCA	ATT	GAT	AAA	CTG	GGA	CTG	AGA	CAC	AAG	GAA	CAC	ATT	GCT	GCT	TAT	GGT	GAA	GGC	AAT	GAG	CGT	958
X82997	GGT	GGT	TAC	GAG	ATT	ATC	AAG	AAG	GCA	ATT	GAT	AAA	CTG	GGA	CTG	AGA	CAC	AAG	GAA	CAC	ATT	GCT	GCT	TAT	GGT	GAA	GGC	AAT	GAG	CGT	931
Bra028132_cds	GGT	GGT	TAC	GAG	ATT	ATC	AAG	AAG	GCA	ATC	GAT	AAA	TTG	GGA	CTG	AGA	CAC	AAG	GAA	CAC	ATT	GCT	GCT	TAT	GGT	GAA	GGC	AAT	GAG	CGC	870
BraGLN1.1_C1	GGT	GGT	TAC	GAG	ATT	ATC	AAG	AAG	GCA	ATC	GAT	AAA	TTG	GGA	CTG	AGA	CAC	AAG	GAA	CAC	ATT	GCT	GCT	TAT	GGT	GAA	GGC	AAT	GAG	CGC	961
BnaGLN1.1_C2	GGT	GGT	TAC	GAG	ATT	ATC	AAG	AAG	GCA	ATC	GAT	AAA	CTG	GGA	ATG	AGA	CAC	AAG	GAA	CAC	ATT	GCT	GCT	TAT	GGT	GAA	GGC	AAT	GAG	CGT	958
Y12460	GGT	GGT	TAC	GAG	ATT	ATC	AAG	AAG	GCA	ATC	GAT	AAA	CTG	GGA	ATG	AGA	CAC	AAG	GAA	CAC	ATT	GCT	GCT	TAT	GGT	GAA	GGC	AAT	GAG	CGT	849
BolGLN1.1_C1	GGT	GGT	TAC	GAG	ATT	ATC	AAG	AAG	GCA	ATC	GAT	AAA	CTG	GGA	ATG	AGA	CAC	AAG	GAA	CAC	ATT	GCT	GCT	TAT	GGT	GAA	GGC	AAT	GAG	CGT	952
At1g66200.1_GLN1.2__cds	GGA	GGA	TAC	GAG	ATA	ATC	AAG	AAG	GCG	ATC	GAG	AAG	CTT	GGC	TTG	AGA	CAC	AAG	GAA	CAC	ATT	TCC	GCT	TAC	GGT	GAA	GGA	AAC	GAG	CGT	870
BnaGLN1.2_C1	GGA	GGA	TAC	GAG	ATA	ATC	AAG	AAG	GCA	ATT	GAT	AAG	CTC	GGA	CTG	AGA	CAC	AAG	GAG	CAC	ATT	TCT	GCT	TAC	GGT	GAA	GGC	AAC	GAG	CGT	956
X76736	GGA	GGA	TAC	GAG	ATA	ATC	AAG	AAG	GCA	ATT	GAT	AAG	CTC	GGA	CTG	AGA	CAC	AAG	GAG	CAC	ATT	TCT	GCT	TAC	GGT	GAA	GGC	AAC	GAG	CGT	937
Bra039756_cds	GGA	GGA	TAC	GAG	ATA	ATC	AAG	AAG	GCA	ATT	GAT	AAG	CTC	GGA	CTG	AGA	CAC	AAG	GAG	CAC	ATT	TCT	GCT	TAC	GGT	GAA	GGC	AAC	GAG	CGT	870
BraGLN1.2_C1	GGA	GGA	TAC	GAG	ATA	ATC	AAG	AAG	GCA	ATT	GAT	AAG	CTC	GGA	CTG	AGA	CAC	AAG	GAG	CAC	ATT	TCT	GCT	TAC	GGT	GAA	GGC	AAC	GAG	CGT	952
EU499383	GGA	GGA	TAC	GAG	ATA	ATC	AAG	AAG	GCA	ATT	GAT	AAG	CTC	GGA	CTG	AGA	CAC	AAG	GAG	CAC	ATT	TCT	GCT	TAC	GGT	GAA	GGC	AAC	GAG	CGT	954
AY773089	GGA	GGA	TAC	GAG	ATA	ATC	AAG	AAG	GCA	ATT	GAT	AAG	CTC	GGA	CTG	AGA	CAC	AAG	GAG	CAC	ATT	TCT	GCT	TAC	GGT	GAA	GGC	AAC	GAG	CGT	958
BnaGLN1.2_C2	GGA	GGA	TAC	GAG	ATA	ATC	AAG	AAG	GCA	ATT	GAT	AAG	CTC	GGA	CTG	AGA	CAC	AAG	GAG	CAC	ATT	TCT	GCT	TAC	GGT	GAA	GGC	AAC	GAG	CGT	959
Y12459	GGA	GGA	TAC	GAG	ATA	ATC	AAG	AAG	GCA	ATT	GAT	AAG	CTC	GGA	CTG	AGA	CAC	AAG	GAG	CAC	ATT	TCT	GCT	TAC	GGT	GAA	GGC	AAC	GAG	CGT	901
BolGLN1.2_C1	GGA	GGA	TAC	GAG	ATA	ATC	AAG	AAG	GCA	ATT	GAT	AAG	CTC	GGA	CTG	AGA	CAC	AAG	GAG	CAC	ATT	TCT	GCT	TAC	GGT	GAA	GGC	AAC	GAG	CGT	849
EU822334	GGA	GGA	TAC	GAG	ATA	ATC	AAG	AAG	GCA	ATT	GAT	AAG	CTC	GGA	CTG	AGA	CAC	AAG	GAG	CAC	ATT	TCT	GCT	TAC	GGT	GAA	GGC	AAC	GAG	CGT	898
EU822335	GGA	GGA	TAC	GAG	ATA	ATC	AAG	AAG	GCA	ATT	GAT	AAG	CTC	GGA	CTG	AGA	CAC	AAG	GAG	CAC	ATT	TCT	GCT	TAC	GGT	GAA	GGC	AAC	GAG	CGT	895
At3g17820_GLN1.3__cds	GGA	GGA	TTA	GAA	GTG	ATC	AAG	AAA	GCG	ATA	GGG	AAG	CTT	CAG	CTG	AAA	CAC	AAA	GAA	CAC	ATT	GCT	GCT	TAC	GGT	GAA	GGA	AAC	GAG	CGT	870
BnaGLN1.3_C2	GGA	GGA	TTA	GCT	GTG	ATT	AAG	AAA	GCG	ATA	GAG	AAG	CTT	CAG	GTG	AAG	CAC	AAG	GAG	CAC	ATT	GCT	GCT	TAC	GGT	GAA	GGC	AAC	GAG	CGT	1017
JX306693	GGA	GGA	TTA	GCT	GTG	ATT	AAG	AAA	GCG	ATA	GAG	AAG	CTT	CAG	GTG	AAG	CAC	AAG	GAG	CAC	ATT	GCT	GCT	TAC	GGT	GAA	GGC	AAC	GAG	CGT	885
Bra022247_cds	GGA	GGA	TTA	GCT	GTG	ATT	AAG	AAA	GCG	ATA	GAG	AAG	CTT	CAG	GTG	AAG	CAC	AAG	GAG	CAC	ATT	GCT	GCT	TAC	GGT	GAA	GGC	AAC	GAG	CGT	870
BraGLN1.3_C1	GGA	GGA	TTA	GCT	GTG	ATT	AAG	AAA	GCG	ATA	GAG	AAG	CTT	CAG	GTG	AAG	CAC	AAG	GAG	CAC	ATT	GCT	GCT	TAC	GGT	GAA	GGC	AAC	GAG	CGT	940
BnaGLN1.3_C1	GGA	GGA	TTA	GCT	GTG	ATT	AAG	AAA	GCC	ATA	GAG	AAG	CTT	CAG	GTG	AAG	CAC	AAG	GAG	CAC	ATT	GCT	GCT	TAC	GGT	GAA	GGC	AAC	GAG	CGT	937
JX306690	GGA	GGA	TTA	GCT	GTG	ATT	AAG	AAA	GCC	ATA	GAG	AAG	CTT	CAG	GTG	AAG	CAC	AAG	GAG	CAC	ATT	GCT	GCT	TAC	GGT	GAA	GGC	AAC	GAG	CGT	904
BolGLN1.3_C1	GGA	GGA	TTA	GCT	GTG	ATT	AAG	AAA	GCC	ATA	GAG	AAG	CTT	CAG	GTG	AAG	CAC	AAG	GAG	CAC	ATT	GCT	GCT	TAC	GGT	GAA	GGC	AAC	GAG	CGT	912
BnaGLN1.3_C4	GGA	GGA	TTA	GCG	GTG	ATA	AAG	AAA	GCG	ATA	GAG	AAG	CTT	CAG	GTG	AAG	CAC	AAG	CAG	CAC	ATT	GCT	GCT	TAC	GGT	GAA	GGC	AAC	GAG	CGT	939
Bra021276_cds	GGA	GGA	TTA	GCG	GTG	ATA	AAG	AAA	GCG	ATA	GAG	AAG	CTT	CAG	GGG	AAG	CAC	AAG	CAG	CAC	ATT	GCT	GCT	TAC	GGT	GAA	GGC	AAC	GAG	CGT	870
BraGLN1.3_C3	GGA	GGA	TTA	GCG	GTG	ATA	AAG	AAA	GCG	ATA	GAG	AAG	CTT	CAG	GGG	AAG	CAC	AAG	CAG	CAC	ATT	GCT	GCT	TAC	GGT	GAA	GGC	AAC	GAG	CGT	824
BnaGLN1.3_C3	GGA	GGA	CTA	GCG	GTG	ATA	AAG	AAA	GCG	ATA	GAG	AAG	CTT	CAG	GTG	AAG	CAC	AAG	CAG	CAC	ATT	GCT	GCT	TAC	GGT	GAA	GGC	AAC	GAG	CGT	897
BnaGLN1.3_C5	GGA	GGA	TTA	GCA	GTG	ATA	AAG	AAA	GCA	ATA	GAG	AAG	CTT	CAG	GTG	AAG	CAC	AAG	GAG	CAC	ATT	GCT	GCT	TAC	GGT	GAA	GGC	AAC	GAG	CGT	951
JX306694	GGA	GGA	TTA	GCA	GTG	ATA	AAG	AAA	GCA	ATA	GAG	AAG	CTT	CAG	GTG	AAG	CAC	AAG	GAG	CAC	ATT	GCT	GCT	TAC	GGT	GAA	GGC	AAC	GAG	CGT	926
Bra001686_cds	GGA	GGA	TTA	GCA	GTG	ATA	AAG	AAA	GCA	ATA	GAG	AAG	CTT	CAG	GTG	AAG	CAC	AAG	GAG	CAC	ATT	GCT	GCT	TAC	GGT	GAA	GGC	AAC	GAG	CGT	870
BraGLN1.3_C2	GGA	GGA	TTA	GCA	GTG	ATA	AAG	AAA	GCA	ATA	GAG	AAG	CTT	CAG	GTG	AAG	CAC	AAG	GAG	CAC	ATT	GCT	GCT	TAC	GGT	GAA	GGC	AAC	GAG	CGT	1033
BnaGLN1.3_C6	GGA	GGC	TTA	GCA	GTG	ATA	AAG	AAA	GCA	ATA	GAG	AAG	CTT	CAG	GTG	AAG	CAC	AAG	GAG	CAC	ATT	GCT	GCT	TAT	GGT	GAA	GGC	AAC	GAG	CGT	924
BolGLN1.3_C2	GGA	GGC	TTA	GCA	GTG	ATA	AAG	AAA	GCA	ATA	GAG	AAG	CTT	CAG	GTG	AAG	CAC	AAG	GAG	CAC	ATT	GCT	GCT	TAC	GGT	GAA	GGC	AAC	GAG	CGT	816
At5g16570_GLN1.4__cds	GGA	GGG	TAC	GAG	GTG	ATA	AAG	AAA	GCA	ATA	GAG	AAG	CTT	GGA	TTG	CGT	CAC	AAG	GAA	CAC	ATT	GCT	GCT	TAT	GGT	GAA	GGC	AAC	GAG	CGT	870
BnaGLN1.4_C1	GGA	GGG	TAC	GAG	ATC	ATA	AAG	AAA	GCG	ATA	GAG	AAG	CTT	GGA	CTG	CGT	CAC	AAG	GAA	CAC	ATC	GCT	GCT	TAT	GGT	GAA	GGC	AAC	GAG	CGT	912
JX306697	GGA	GGG	TAC	GAG	ATC	ATA	AAG	AAA	GCG	ATA	GAG	AAG	CTT	GGA	CTG	CGT	CAC	AAG	GAA	CAC	ATC	GCT	GCT	TAT	GGT	GAA	GGC	AAC	GAG	CGT	887
JX306692	GGA	GGG	TAC	GAG	ATC	ATA	AAG	AAA	GCG	ATA	GAG	AAG	CTT	GGA	CTG	CGT	CAC	AAG	GAA	CAC	ATC	GCT	GCT	TAT	GGT	GAA	GGC	AAC	GAG	CGT	768
Bra023573_cds	GGA	GGG	TAC	GAG	ATC	ATA	AAG	AAA	GCG	ATA	GAG	AAG	CTT	GGA	CTG	CGT	CAC	AAG	GAA	CAC	ATC	GCT	GCT	TAT	GGT	GAA	GGC	AAC	GAG	CGT	867
BraGLN1.4_C1	GGA	GGG	TAC	GAG	ATC	ATA	AAG	AAA	GCG	ATA	GAG	AAG	CTT	GGA	CTG	CGT	CAC	AAG	GAA	CAC	ATC	GCT	GCT	TAT	GGT	GAA	GGC	AAC	GAG	CGT	992
BnaGLN1.4_C2	GGA	GGG	TAC	GAG	ATC	ATA	AAG	AAA	GCG	ATA	GAG	AAG	CTT	GGA	CTT	CGT	CAC	AAG	GAA	CAC	ATT	GCT	GCT	TAT	GGT	GAA	GGC	AAC	GAG	CGT	937
JX306695	GGA	GGG	TAC	GAG	ATC	ATA	AAG	AAA	GCG	ATA	GAG	AAG	CTT	GGA	CTT	CGT	CAC	AAG	GAA	CAC	ATT	GCT	GCT	TAT	GGT	GAA	GGC	AAC	GAG	CGT	911
JX306696	GGA	GGG	TAC	GAG	ATC	ATA	AAG	AAA	GCG	ATA	GAG	AAG	CTT	GGA	CTG	CGT	CAC	AAG	GAA	CAC	ATC	GCT	GCT	TAT	GGT	GAA	GGC	AAC	GAG	CGT	888
BolGLN1.4_C1	GGA	GGG	TAC	GAG	ATC	ATA	AAG	AAA	GCG	ATA	GAG	AAG	CTT	GGA	CTT	CGT	CAC	AAG	GAA	CAC	ATT	GCT	GCT	TAT	GGT	GAA	GGC	AAC	GAG	CGT	912
BnaGLN1.4_C4	GGA	GGG	TAC	GAG	ATC	ATA	AAG	AAA	GCG	ATA	GAG	AAG	CTT	GGA	TTG	CGT	CAC	AAG	GAA	CAC	ATC	TCT	GCT	TAT	GGT	GAA	GGC	AAC	GAG	CGT	873
JX306700	GGA	GGG	TAC	GAG	ATC	ATA	AAG	AAA	GCG	ATA	GAG	AAG	CTT	GGA	TTG	CGT	CAC	AAG	GAA	CAC	ATC	TCT	GCT	TAT	GGT	GAA	GGC	AAC	GAG	CGT	549
JX306701	GGA	GGG	TAC	GAG	ATC	ATA	AAG	AAA	GCG	ATA	GAG	AAG	CTT	GGA	TTG	CGT	CAC	AAG	GAA	CAC	ATC	TCT	GCT	TAT	GGT	GAA	GGC	AAC	GAG	CGT	549

Bra008612_cds	GGA GGG TAC GAG GTC ATA AAG AAA GCG ATA GAG AAG CTT GGA TTG CGT CAC AAG GAA CAC ATC TCT GCT TAT GGT GAA GGC AAC GAG CGA	870
BraGLN1.4_C2	GGA GGG TAC GAG GTC ATA AAG AAA GCG ATA GAG AAG CTT GGA TTG CGT CAC AAG GAA CAC ATC TCT GCT TAT GGT GAA GGC AAC GAG CGA	915
BnaGLN1.4_C3	GGA GGG TAC GAG GTG ATA AAG AAA GCG ATA GAG AAG CTT GGA TTG CGT CAC AAG GAA CAC ATC TCT GCT TAT GGT GAA GGC AAC GAG CGT	914
JX306698	GGA GGG TAC GAG GTG ATA AAG AAA GCG ATA GAG AAG CTT GGA TTG CGT CAC AAG GAA CAC ATC TCT GCT TAT GGT GAA GGC AAC GAG CGT	575
JX306699	GGA GGG TAC GAG GTG ATA AAG AAA GCG ATA GAG AAG CTT GGA TTG CGT CAC AAG GAA CAC ATC TCT GCT TAT GGT GAA GGC AAC GAG CGT	575
BolGln1.4_C2	GGA GGG TAC GAG GTG ATA AAG AAA GCG ATA GAG AAG CTT GGA TTG CGT CAC AAG GAA CAC ATC TCT GCT TAT GGT GAA GGC AAC GAG CGT	874
At1g48470_GLN1.5__cds	GGA GGA CTG GAT TTG ATT AAG GAA GCA ATA AAG AAG CTT GAA GTG AAA CAC AAA CAA CAC ATT GCT GCT TAT GGT GAA GGC AAC GAG AGG	870
BnaGLN1.5_C2	GGA GGA TTG GAC TTG ATC AAG AAA GCA ATA GGG AAG CTT GAA GTG AAG CAT AAA CAA CAC ATT GCT GCT TAT GGT GAA GGC AAT GAG AGG	992
Bra018729_cds	GGA GGA TTG GAT TTG ATC AAG AAA GCA ATA GGG AAG CTT GAA GTG AAA CAT AAA CAA CAC ATT GCT GCT TAT GGT GAA GGC AAT GAG AGG	825
BraGLN1.5_C1	GGA GGA TTG GAT TTG ATC AAG AAA GCA ATA GGG AAG CTT GAA GTG AAA CAT AAA CAA CAC ATT GCT GCT TAT GGT GAA G-- --- --- ---	894
BnaGLN1.5_C1	GGA GGA TTG GAC TTG ATA AAA AAA GCA ATA GGG AAG CTT GAA GTG AAG CAT AAA CAA CAC ATT GCT GCT TAT GGT GAA GGC AAT GAG AGG	967
JX306691	GGA GGA TTG GAC TTG ATA AAA AAA GCA ATA GGG AAG CTT GAA GTG AAG CAT AAA CAA CAC ATT GCT GCT TAT GGT GAA GGC AAT GAG AGG	639
BolGLN1.5_C1	--- ---	578

				1 360						1 380							1 400							1 420					1 440	
At5g37600_GLN1.1__cds	CGT	CTC	ACA	GGA	CAC	CAC	GAG	ACT	GCT	GAC	ATC	AAC	ACT	TTC	CTT	TGG	GGT	GTT	GCG	AAC	CGT	GGA	---	GCA	TCG	ATC	CGA	GTA	GGA	CGT	957
BnaGLN1.1_C1	CGT	CTC	ACG	GGT	CAC	CAC	GAG	ACT	GCT	GAC	ATC	AAC	ACT	TTC	CTC	TGG	GGT	GTT	GCG	AAC	CGT	GGA	---	GCA	TCA	ATC	CGT	GTA	GGA	CGT	1045
X82997	CGT	CTC	ACG	GGT	CAC	CAC	GAG	ACT	GCT	GAC	ATC	AAC	ACT	TTC	CTC	TGG	GGT	GTT	GCG	AAC	CGT	GGA	---	GCA	TCA	ATC	CGT	GTA	GGA	CGT	1018
Bra028132_cds	CGT	CTC	ACG	GGT	CAC	CAC	GAG	ACT	GCT	GAC	ATC	AAC	ACT	TTC	CTC	TGG	GGT	GTT	GCG	AAC	CGT	GGA	---	GCA	TCA	ATC	CGT	GTA	GGA	CGT	957
BraGLN1.1_C1	CGT	CTC	ACG	GGT	CAC	CAC	GAG	ACT	GCT	GAC	ATC	AAC	ACT	TTC	CTC	TGG	GGT	GTT	GCG	AAC	CGT	GGA	---	GCA	TCA	ATC	CGT	GTA	GGA	CGT	1048
BnaGLN1.1_C2	CGT	CTC	ACG	GGT	CAT	CAC	GAG	ACT	GCT	GAC	ATC	AAC	ACT	TTC	CTC	TGG	GGT	GTT	GCG	AAT	CGT	GGA	---	GCA	TCA	ATC	CGT	GTA	GGA	CGC	1045
Y12460	CGT	CTC	ACG	GGT	CAT	CAC	GAG	ACT	GCT	GAC	ATC	AAC	ACT	TCC	TCC	TGG	GGT	GTT	GCG	AAC	CGT	GGA	---	GCA	TCA	ATC	CGT	GTA	GGA	CGT	936
BolGLN1.1_C1	CGT	CTC	ACG	GGT	CAT	CAT	GAG	ACT	GCT	GAC	ATC	AAC	ACT	TTC	CTC	TGG	GGT	GTT	GCG	AAC	CGT	GGA	---	GCA	TCA	ATC	CGT	GTA	GGA	CGT	1039
At1g66200.1_GLN1.2__cds	CGT	CTC	ACG	GGA	CAC	CAT	GAA	ACT	GCT	GAC	ATC	AAC	ACT	TTC	CTT	TGG	GGT	GTT	GCG	AAC	CGT	GGT	---	GCA	TCG	ATC	CGA	GTA	GGA	CGT	957
BnaGLN1.2_C1	CGT	CTC	ACT	GGA	CAC	CAT	GAA	ACT	GCT	GAT	ATC	AAC	ACT	TTC	AAA	TGG	GGT	GTT	GCA	AAC	CGT	GGA	---	GCA	TCA	ATC	CGT	GTA	GGA	CGT	1043
X76736	CGT	CTC	ACT	GGA	CAC	CAT	GAA	ACT	GCT	GAT	ATC	AAC	ACT	TTC	AAA	TGG	GGT	GTT	GCA	AAC	CGT	GGA	---	GCA	TCA	ATC	CGT	GTA	GGA	CGT	1024
Bra039756_cds	CGT	CTC	ACT	GGA	CAC	CAT	GAA	ACT	GCT	GAT	ATC	AAC	ACT	TTC	AAA	TGG	GGT	GTT	GCA	AAC	CGT	GGA	---	GCA	TCA	ATC	CGT	GTA	GGA	CGT	957
BraGLN1.2_C1	CGT	CTC	ACT	GGA	CAC	CAT	GAA	ACT	GCT	GAT	ATC	AAC	ACT	TTC	AAA	TGG	GGT	GTT	GCA	AAC	CGT	GGA	---	GCA	TCA	ATC	CGT	GTA	GGA	CGT	1039
EU499383	CGT	CTC	ACT	GGA	CAC	CAT	GAA	ACT	GCT	GAT	ATC	AAC	ACT	TTC	AAA	TGG	GGT	GTT	GCA	AAC	CGT	GGA	---	GCA	TCA	ATC	CGT	GTA	GGA	CGT	1041
AY773089	CGT	CTC	ACT	GGA	CAC	CAT	GAA	ACT	GCT	GAT	ATC	AAC	ACT	TTC	AAA	TGG	GGT	GTT	GCA	AAC	CGT	GGA	---	GCA	TCA	ATC	CGT	GTA	GGA	CGT	1045
BnaGLN1.2_C2	CGT	CTC	ACC	GGA	CAC	CAT	GAA	ACT	GCT	GAC	ATC	AAC	ACT	TTC	AAA	TGG	GGT	GTT	GCA	AAC	CGT	GGA	---	GCA	TCA	ATC	CGT	GTA	GGA	CGT	1046
Y12459	CGT	CTC	ACC	GGA	CAC	CAT	GAA	ACT	GCT	GAC	ATC	AAC	ACT	TTC	AAA	TGG	GGT	GTT	GCA	AAC	CGT	GGA	---	GCA	TCA	ATC	CGT	GTA	GGA	CGT	988
BolGLN1.2_C1	CGT	CTC	ACC	GGA	CAC	CAT	GAA	ACT	GCT	GAC	ATC	AAC	ACT	TTC	AAA	TGG	GGT	GTT	GCA	AAC	CGT	GGA	---	GCA	TCA	ATC	CGT	GTA	GGA	CGT	936
EU822334	CGT	CTC	ACC	GGA	CAC	CAT	GAA	ACT	GCT	GAC	ATC	AAC	ACT	TTC	AAA	TGG	GGT	GTT	GCA	AAC	CGT	GGA	---	GCA	TCA	ATC	CGT	GTA	GGA	CGT	985
EU822335	CGT	CTC	ACC	GGA	CAC	CAT	GAA	ACT	GCT	GAC	ATC	AAC	ACT	TTC	AAA	TGG	GGT	GTT	GCA	AAC	CGT	GGA	---	GCA	TCA	ATC	CGT	GTA	GGA	CGT	982
At3g17820_GLN1.3__cds	CGT	CTC	ACT	GGA	AAG	CAC	GAA	ACC	GCA	GAC	ATC	AAC	ACA	TTC	TCT	TGG	GGA	GTC	GCG	AAC	CGT	GGA	---	GCG	TCA	GTG	AGA	GTG	GGA	CGT	957
BnaGLN1.3_C2	CGT	CTC	ACG	GGG	AAG	CAC	GAA	ACT	GCA	GAC	ATC	AAC	ACG	TTC	TCT	TGG	GGA	GTG	GCG	AAC	CGT	GGG	---	GCT	TCG	GTG	AGA	GTG	GGG	AGA	1104
JX306693	CGT	CTC	ACG	GGG	AAG	CAC	GAA	ACT	GCA	GAC	ATC	AAC	ACG	TTC	TCT	TGG	GGA	GTG	GCG	AAC	CGT	GGG	---	GCT	TCG	GTG	AGA	GTG	GGG	AGA	972
Bra022247_cds	CGT	CTC	ACG	GGG	AAG	CAC	GAA	ACT	GCA	GAC	ATC	AAC	ACG	TTC	TCT	TGG	GGA	GTG	GCG	AAC	CGT	GGT	---	GCT	TCG	GTG	AGA	GTG	GGG	AGA	957
BraGLN1.3_C1	CGT	CTC	ACG	GGG	AAG	CAC	GAA	ACT	GCA	GAC	ATC	AAC	ACG	TTC	TCT	TGG	GGA	GTG	GCG	AAC	CGT	GGT	---	GCT	TCG	GTG	AGA	GTG	GGG	AGA	1027
BnaGLN1.3_C1	CGC	CTC	ACG	GGG	AAG	CAC	GAG	ACC	GCG	GAC	ATC	AAC	ACG	TTC	TCT	TGG	GGA	GTG	GCG	AAC	CGT	GGA	---	GCT	TCG	GTG	AGA	GTG	GGA	CGT	1024
JX306690	CGC	CTC	ACG	GGG	AAG	CAC	GAG	ACC	GCG	GAC	ATC	AAC	ACG	TTC	TCT	TGG	GGA	GTG	GCG	AAC	CGT	GGA	---	GCT	TCG	GTG	AGA	GTG	GGA	CGT	991
BolGLN1.3_C1	CGC	CTC	ACG	GGG	AAG	CAC	GAG	ACC	GCG	GAC	ATC	AAC	ACG	TTC	TCT	TGG	GGA	GTG	GCG	AAC	CGT	GGA	---	GCT	TCG	GTG	AGA	GTG	GGA	CGT	999
BnaGLN1.3_C4	CGT	CTC	ACG	GGC	AAG	CAC	GAG	ACG	GCA	GAT	ATC	AAC	ACG	TTC	TCA	TGG	GGA	GTG	GCG	AAC	CGT	GGA	---	GCT	TCG	GTG	AGA	GTG	GGA	CGT	1026
Bra021276_cds	CGT	CTC	ACG	GGC	AAG	CAC	GAG	ACG	GCA	GAT	ATC	AAC	ACG	TTC	TCA	TGG	GGA	GTG	GCG	AAC	CGT	GGA	---	GCT	TCG	GTG	AGA	GTG	GGA	CGT	957
BraGLN1.3_C3	CGT	CTC	ACG	GGC	AAG	CAC	GAG	ACG	GCA	GAT	ATC	AAC	ACG	TTC	TCA	TGG	GGA	GTG	GCG	AAC	CGT	GGA	---	GCT	TCG	GTG	AGA	GTG	GGA	CGT	911
BnaGLN1.3_C3	CGT	CTC	ACG	GGG	AAG	CAC	GAG	ACG	GCA	GAT	ATC	AAC	ACG	TTC	TCG	TGG	GGA	GTG	GCG	AAC	CGT	GGA	---	GCA	TCG	GTG	AGA	GTG	GGA	CGT	984
BnaGLN1.3_C5	CGT	CTC	ACG	GGG	AAG	CAT	GAA	ACA	GCA	GAC	ATC	AAC	ACG	TTC	TCT	TGG	GGA	GTG	GCG	AAC	CGT	GGA	---	GCT	TCG	GTG	AGA	GTG	GGA	CGT	1038
JX306694	CGT	CTC	ACG	GGG	AAG	CAT	GAA	ACA	GCA	GAC	ATC	AAC	ACG	TTC	TCT	TGG	GGA	GTG	GCG	AAC	CGT	GGA	---	GCT	TCG	GTG	AGA	GTG	GGA	CGT	1013
Bra001686_cds	CGT	CTC	ACG	GGG	AAG	CAT	GAA	ACA	GCA	GAC	ATC	AAC	ACG	TTC	TCT	TGG	GGA	GTG	GCG	AAC	CGT	GGA	---	GCT	TCG	GTG	AGA	GTG	GGA	CGA	957
BraGLN1.3_C2	CGT	CTC	ACG	GGG	AAG	CAT	GAA	ACA	GCA	GAC	ATC	AAC	ACG	TTC	TCT	TGG	GGA	GTG	GCG	AAC	CGT	GGA	---	GCT	TCG	GTG	AGA	GTG	GGA	CGT	1120
BnaGLN1.3_C6	CGT	CTC	ACG	GGG	AAG	CAT	GAA	ACA	GCA	GAC	ATC	AAC	ACG	TTC	TCT	TGG	GGA	GTG	GCG	AAC	CGT	GGA	---	GCT	TCG	GTG	AGA	GTG	GGA	CGT	1011
BolGLN1.3_C2	CGT	CTC	ACG	GGA	AAG	CAC	GAG	ACA	GCA	GAC	ATC	AAC	ACG	TTC	TCT	TGG	GGA	GTG	GCG	AAC	CGT	GGA	---	GCT	TCG	GTG	AGA	GTG	GGA	CGT	903
At5g16570_GLN1.4__cds	CGT	CTC	ACC	GGA	AAA	CAT	GAA	ACC	GCC	GAT	ATC	AAC	ACT	TTC	TTA	TGG	GGT	GTG	GCA	AAC	CGT	GGG	---	GCA	TCG	ATT	AGG	GTT	GGG	CGT	957
BnaGLN1.4_C1	CGT	CTC	ACT	GGA	AGA	CAC	GAG	ACT	GCT	GAT	ATC	AAC	ACT	TTC	TTA	TGG	GGT	GTT	GCA	AAC	CGT	GGG	---	GCA	TCG	ATT	AGG	GTT	GGT	CGT	999
JX306697	CGT	CTC	ACT	GGA	AGA	CAC	GAG	ACT	GCT	GAT	ATC	AAC	ACT	TTC	TTA	TGG	GGT	GTT	GCA	AAC	CGT	GGG	---	GCA	TCG	ATT	AGG	GTT	GGT	CGT	974
JX306692	CGT	CTC	ACT	GGA	AGA	CAC	GAG	ACT	GCT	GAT	ATC	AAC	ACT	TTC	TTA	TGG	GGT	GTT	GCA	AAC	CGT	GGG	G--	GCA	TCG	ATT	AGG	GTT	GGT	CGT	856
Bra023573_cds	CGT	CTC	ACT	GGA	AGA	CAC	GAG	ACT	GCT	GAT	ATC	AAC	ACT	TTC	TTA	TGG	GGT	GTT	GCA	AAC	CGT	GGG	---	GCA	TCG	ATT	AGG	GTT	GGT	CGT	954
BraGLN1.4_C1	CGT	CTC	ACT	GGA	AGA	CAC	GAG	ACT	GCT	GAT	ATC	AAC	ACT	TTC	TTA	TGG	GGT	GTT	GCA	AAC	CGT	GGG	---	GCA	TCG	ATT	AGG	GTT	GGT	CGT	1079
BnaGLN1.4_C2	CGT	CTC	ACT	GGA	AAA	CAC	GAG	ACT	GCT	GAT	ATC	AAC	ACT	TTC	TTA	TGG	GGT	GTT	GCA	AAC	CGT	GGG	---	GCA	TCG	ATT	AGG	GTT	GGT	CGT	1024
JX306695	CGT	CTC	ACT	GGA	AAA	CAC	GAG	ACT	GCT	GAT	ATC	AAC	ACT	TTC	TTA	TGG	GGT	GTT	GCA	AAC	CGT	GGG	---	GCA	TCG	ATT	AGG	GTT	GGT	CGT	998
JX306696	CGT	CTC	ACT	GGA	AGA	CAC	GAG	ACT	GCT	GAT	ATC	AAC	ACT	TTC	TTA	TGG	GGT	GTT	GCA	AAC	CGT	GGG	---	GCA	TCG	ATT	AGG	GTT	GGT	CGT	975
BolGLN1.4_C1	CGT	CTC	ACT	GGA	AAA	CAC	GAG	ACT	GCT	GAT	ATC	AAC	ACT	TTC	TTA	TGG	GGT	GTT	GCA	AAC	CGT	GGG	---	GCA	TCG	ATT	AGG	GTT	GGT	CGT	999
BnaGLN1.4_C4	CGT	CTC	ACT	GGC	AAA	CAC	GAG	ACT	GCC	GAT	ATC	AAC	ACT	TTC	TTA	TGG	GGT	GTG	GCC	AAC	CGT	GGG	---	GCA	TCG	ATT	AGG	GTT	GGT	CGG	960
JX306700	CGT	CTC	ACT	GGC	AAA	CAC	GAG	ACT	GCC	GAT	ATC	AAC	ACT	TTC	TTA	TGG	GGT	GTG	GCC	AAC	CGT	GGG	---	GCA	TCG	ATT	AGG	GTT	GGT	CGG	636
JX306701	CGT	CTC	ACT	GGC	AAA	CAC	GAG	ACT	GCC	GAT	ATC	AAC	ACT	TTC	TTA	TGG	GGT	GTG	GCC	AAC	CGT	GGG	---	GCA	TCG	ATT	AGG	GTT	GGT	CGG	636

Bra008612_cds	CGT	CTC	ACT	GGC	AAA	CAC	GAG	ACT	GCC	GAT	ATC	AAC	ACT	TTC	GTA	TGG	GGT	GTG	GCC	AAC	CGT	GGG	---	GCA	TCG	ATT	AGG	GTT	GGT	CGG	957
BraGLN1.4_C2	CGT	CTC	ACT	GGC	AAA	CAC	GAG	ACT	GCC	GAT	ATC	AAC	ACT	TTC	GTA	TGG	GGT	GTG	GCC	AAC	CGT	GGG	---	GCA	TCG	ATT	AGG	GTT	GGT	CGG	1002
BnaGLN1.4_C3	CGC	CTC	ACT	GGC	AAA	CAC	GAG	ACT	GCC	GAT	ATC	AAC	ACT	TTC	TTA	TGG	GGT	GTG	GCC	AAC	CGT	GGG	---	GCA	TCG	ATT	AGA	GTT	GGT	CGG	1001
JX306698	CGC	CTC	ACT	GGC	AAA	CAC	GAG	ACT	GCC	GAT	ATC	AAC	ACT	TTC	TTA	TGG	GGT	GTG	GCC	AAC	CGT	GGG	---	GCA	TCG	ATT	AGA	GTT	GGT	CGG	662
JX306699	CGC	CTC	ACT	GGC	AAA	CAC	GAG	ACT	GCC	GAT	ATC	AAC	ACT	TTC	TTA	TGG	GGT	GTG	GCC	AAC	CGT	GGG	---	GCA	TCG	ATT	AGA	GTT	GGT	CGG	662
BolGln1.4_C2	CGC	CTC	ACT	GGC	AAA	CAC	GAG	ACT	GCC	GAT	ATC	AAC	ACT	TTC	TTA	TGG	GGT	GTG	GCC	AAC	CGT	GGG	---	GCA	TCG	ATT	AGA	GTT	GGT	CGG	961
At1g48470_GLN1.5__cds	CGT	CTC	ACT	GGG	AAG	CAT	GAA	ACT	GCA	GAC	ATC	AAC	ACT	TTC	TCT	TGG	GGA	GTG	GCG	GAT	CGT	GGA	---	GCA	TCG	GTG	AGA	GTA	GGA	AGA	957
BnaGLN1.5_C2	CGT	CTC	ACT	GGG	AAG	CAT	GAA	ACC	GCA	GAC	ATC	AAC	AAG	TTC	TCT	TGG	GGA	GTT	GCG	GAT	CGT	GGA	---	GCA	TCG	GTG	AGA	GTG	GGA	AGA	1079
Bra018729_cds	CGT	CTC	ACT	GGG	AAG	CAT	GAA	ACC	GCA	GAC	ATC	AAC	AAG	TTC	TCT	TGG	--A	GTT	GCG	GAT	CGT	GGA	---	GCA	TCG	GTG	AGA	GTG	GGA	AGA	910
BraGLN1.5_C1	---	---	---	---	---	---	---	---	---	---	---	---	---	---	---	---	---	---	---	---	---	---	---	---	---	---	---	---	---	---	894
BnaGLN1.5_C1	CGC	CTC	ACT	GGG	AAG	CAT	GAA	ACC	GCA	GAC	ATC	AAC	AAG	TTC	TCT	TGG	GGA	GTT	GCG	GAT	CGT	GGA	---	GCA	TCG	GTG	AGA	GTG	GGA	AGA	1054
JX306691	CGC	CTC	ACT	GGG	AAG	CAT	GAA	ACC	GCA	GAC	ATC	AAC	AAG	TTC	TCT	TGG	GGA	GTT	GCG	GAT	CGT	GGA	---	GCA	TCG	GTG	AGA	GTG	GGA	AGA	726
BolGLN1.5_C1	---	---	---	---	---	---	---	---	---	---	---	---	---	---	---	---	---	---	---	---	---	---	---	---	---	---	---	---	---	---	578

BnaGLN1_JXB

							1 460							1 480						1 500							1 520				
At5g37600_GLN1.1__cds	GAT	ACG	GAG	AAA	GAA	GGG	AAA	GGA	TAC	TTT	GAG	GAC	AGG	AGG	CCA	GCT	TCG	AAC	ATG	GAT	CCT	TAC	ATT	GTC	ACT	TCC	ATG	ATT	GCA	GAG	1047
BnaGLN1.1_C1	GAC	ACA	GAG	AAA	GAA	GGG	AAA	GGA	TAC	TTT	GAG	GAT	AGG	AGG	CCA	GCT	TCG	AAC	ATG	GAT	CCT	TAC	ATT	GTG	ACT	TCC	ATG	ATT	GCA	GAG	1135
X82997	GAC	ACA	GAG	AAA	GAA	GGG	AAA	GGA	TAC	TTT	GAG	GAT	AGG	AGG	CCA	GCT	TCG	AAC	ATG	GAT	CCT	TAC	ATT	GTG	ACT	TCC	ATG	ATT	GCA	GAG	1108
Bra028132_cds	GAC	ACA	GAG	AAA	GAA	GGG	AAA	GGA	TAC	TTT	GAG	GAT	AGG	AGG	CCA	GCT	TCG	AAC	ATG	GAT	CCT	TAC	ATT	GTG	ACT	TCC	ATG	ATT	GCA	GAT	1047
BraGLN1.1_C1	GAC	ACA	GAG	AAA	GAA	GGG	AAA	GGA	TAC	TTT	GAG	GAT	AGG	AGG	CCA	GCT	TCG	AAC	ATG	GAT	CCT	TAC	ATT	GTG	ACT	TCC	ATG	ATT	GCA	GAT	1138
BnaGLN1.1_C2	GAC	ACA	GAG	AAA	GAA	GGG	AAA	GGA	TAC	TTT	GAG	GAT	AGG	AGG	CCA	GCT	TCG	AAC	ATG	GAT	CCT	TAC	ATT	GTG	ACT	TCC	ATG	ATT	GCA	GAG	1135
Y12460	GAC	ACA	GAG	AAA	GAA	GGG	AAA	GGA	TAC	TTT	GAG	GAT	AGG	AGG	CCA	GCT	TCG	AAC	ATG	GAT	CCT	TAC	ATT	GTG	ACT	TCC	ATG	ATT	GCA	GAG	1026
BolGLN1.1_C1	GAC	ACA	GAG	AAA	GAA	GGG	AAA	GGA	TAC	TTT	GAG	GAT	AGG	AGG	CCA	GCT	TCG	AAC	ATG	GAT	CCT	TAC	ATT	GTG	ACT	TCC	ATG	ATT	GCA	GAG	1129
At1g66200.1_GLN1.2__cds	GAC	ACC	GAG	AAA	GAA	GGG	AAG	GGA	TAC	TTT	GAG	GAT	AGG	AGG	CCA	GCT	TCA	AAC	ATG	GAC	CCT	TAC	GTT	GTT	ACT	TCC	ATG	ATT	GCA	GAG	1047
BnaGLN1.2_C1	GAC	ACG	GAG	AAG	GAA	GGG	AAA	GGA	TAC	TTT	GAG	GAT	AGG	AGG	CCA	GCT	TCC	AAC	ATG	GAC	CCT	TAC	ACT	GTA	ACT	TCC	ATG	ATT	GCA	GAG	1133
X76736	GAC	ACG	GAG	AAG	GAA	GGG	AAA	GGA	TAC	TTT	GAG	GAT	AGG	AGG	CCA	GCT	TCC	AAC	ATG	GAC	CCT	TAC	ACT	GTA	ACT	TCC	ATG	ATT	GCA	GAG	1114
Bra039756_cds	GAC	ACG	GAG	AAG	GAA	GGG	AAA	GGA	TAC	TTT	GAG	GAT	AGG	AGG	CCA	GCT	TCC	AAC	ATG	GAC	CCT	TAC	ACT	GTA	ACT	TCC	ATG	ATT	GCA	GAG	1047
BraGLN1.2_C1	GAC	ACG	GAG	AAG	GAA	GGG	AAA	GGA	TAC	TTT	GAG	GAT	AGG	AGG	CCA	GCT	TCC	AAC	ATG	GAC	CCT	TAC	ACT	GTA	ACT	TCC	ATG	ATT	GCA	GAG	1129
EU499383	GAC	ACG	GAG	AAG	GAA	GGG	AAA	GGA	TAC	TTT	GAG	GAT	AGG	AGG	CCA	GCT	TCC	AAC	ATG	GAC	CCT	TAC	ACT	GTA	ACT	TCC	ATG	ATT	GCA	GAG	1131
AY773089	GAC	ACG	GAG	AAG	GAA	GGG	AAA	GGA	TAC	TTT	GAG	GAT	AGG	AGG	CCA	GCT	TCC	AAC	ATG	GAC	CCT	TAC	ACT	GTA	ACT	TCC	ATG	ATT	GCA	GAG	1135
BnaGLN1.2_C2	GAC	ACG	GAG	AAG	GAA	GGG	AAA	GGA	TAC	TTT	GAG	GAT	AGG	AGG	CCA	GCT	TCC	AAC	ATG	GAC	CCT	TAC	ACT	GTA	ACT	TCC	ATG	ATT	GCA	GAG	1136
Y12459	GAC	ACG	GAG	AAG	GAA	GGG	AAA	GGA	TAC	TTT	GAG	GAT	AGG	AGG	CCA	GCT	TCC	AAC	ATG	GAC	CCT	TAC	ACT	GTA	ACT	TCC	ATG	ATT	GCA	GAG	1078
BolGLN1.2_C1	GAC	ACG	GAG	AAG	GAA	GGG	AAA	GGA	TAC	TTT	GAG	GAT	AGG	AGG	CCA	GCT	TCC	AAC	ATG	GAC	CCT	TAC	ACT	GTA	ACT	TCC	ATG	ATT	GCA	GAG	1026
EU822334	GAC	ACG	GAG	AAG	GAA	GGG	AAA	GGA	TAC	TTT	GAG	GAT	AGG	AGG	CCA	GCT	TCC	AAC	ATG	GAC	CCT	TAC	ACT	GTA	ACT	TCC	ATG	ATT	GCA	GAG	1075
EU822335	GAC	ACG	GAG	AAG	GAA	GGG	AAA	GGA	TAC	TTT	GAG	GAT	AGG	AGG	CCA	GCT	TCC	AAC	ATG	GAC	CCT	TAC	ACT	GTA	ACT	TCC	ATG	ATT	GCA	GAG	1072
At3g17820_GLN1.3__cds	GAC	ACA	GAG	AAG	GAA	GGT	AAA	GGG	TAC	TTC	GAA	GAC	AGA	AGG	CCA	GCT	TCT	AAC	ATG	GAT	CCT	TAC	GTT	GTC	ACC	TCC	ATG	ATC	GCT	GAG	1047
BnaGLN1.3_C2	GAC	ACT	GAG	AAA	GAA	GGC	AAA	GGT	TAC	TTC	GAG	GAC	AGA	AGG	CCA	GCT	TCT	AAC	ATG	GAT	CCT	TAC	GTT	GTT	ACG	TCC	ATG	ATC	GCT	GAA	1194
JX306693	GAC	ACT	GAG	AAA	GAA	GGC	AAA	GGT	TAC	TTC	GAG	GAC	AGA	AGG	CCA	GCT	TCT	AAC	ATG	GAT	CCT	TAC	GTT	GTT	ACG	TCC	ATG	ATC	GCT	GAA	1062
Bra022247_cds	GAC	ACT	GAG	AAA	GAA	GGC	AAA	GGT	TAC	TTC	GAG	GAC	AGA	AGG	CCA	GCT	TCT	AAC	ATG	GAT	CCT	TAC	GTT	GTT	ACG	TCC	ATG	ATT	GCT	GAA	1047
BraGLN1.3_C1	GAC	ACT	GAG	AAA	GAA	GGC	AAA	GGT	TAC	TTC	GAG	GAC	AGA	AGG	CCA	GCT	TCT	AAC	ATG	GAT	CCT	TAC	GTT	GTT	ACG	TCC	ATG	ATT	GCT	GAA	1117
BnaGLN1.3_C1	GAC	ACT	GAG	AAA	GAA	GGC	AAA	GGT	TAC	TTC	GAG	GAC	AGA	AGG	CCA	GCT	TCT	AAC	ATG	GAT	CCT	TAC	GTT	GTT	ACT	TCC	ATG	ATT	GCT	GAA	1114
JX306690	GAC	ACT	GAG	AAA	GAA	GGC	AAA	GGT	TAC	TTC	GAG	GAC	AGA	AGG	CCA	GCT	TCT	AAC	ATG	GAT	CCT	TAC	GTT	GTT	ACT	TCC	ATG	ATT	GCT	GAA	1081
BolGLN1.3_C1	GAC	ACT	GAG	AAA	GAA	GGC	AAA	GGT	TAC	TTC	GAG	GAC	AGA	AGG	CCA	GCT	TCT	AAC	ATG	GAT	CCT	TAC	GTT	GTT	ACT	TCT	ATG	ATT	GCT	GAA	1089
BnaGLN1.3_C4	GAC	ACT	GAG	AAA	GAA	GGC	AAA	GGT	TAC	TTC	GAG	GAT	CGT	AGG	CCA	GCT	TCT	AAC	ATG	GAT	CCT	TAT	GTC	GTC	ACT	TCC	ATG	ATC	GCC	GAA	1116
Bra021276_cds	GAC	ACT	GAG	AAA	GAA	GGC	AAA	GGT	TAC	TTC	GGG	GAT	CGT	AGG	CCA	GCT	TCT	AAC	ATG	GAT	CCT	TAT	GTC	GTC	ACT	TCC	ATG	ATC	GCC	GAA	1047
BraGLN1.3_C3	GAC	ACT	GAG	AAA	GAA	GGC	AAA	GGT	TAC	TTC	GGG	GAT	CGT	AGG	CCA	GCT	TCT	AAC	ATG	GAT	C--	---	---	---	---	---	---	---	---	---	972
BnaGLN1.3_C3	GAC	ACT	GAG	AAA	GAA	GGC	AAA	GGT	TAC	TTC	GAG	GAT	CGT	AGG	CCA	GCT	TCT	AAC	ATG	GAT	CCT	TAT	GTC	GTT	ACT	TCC	ATG	ATC	GCT	GAA	1074
BnaGLN1.3_C5	GAC	ACT	GAG	AAA	GAA	GGC	AAA	GGT	TAC	TTC	GAG	GAT	CGT	AGG	CCA	GCT	TCT	AAC	ATG	GAT	CCT	TAT	GTC	GTC	ACT	TCC	ATG	ATC	GCT	GAA	1128
JX306694	GAC	ACT	GAG	AAA	GAA	GGC	AAA	GGT	TAC	TTC	GAG	GAT	CGT	AGG	CCA	GCT	TCT	AAC	ATG	GAT	CCT	TAT	GTC	GTC	ACT	TCC	ATG	ATC	GCT	GAA	1103
Bra001686_cds	GAC	ACT	GAG	AAG	GAA	GGC	AAA	GGT	TAC	TTC	GAA	GAC	AGA	AGG	CCA	GCT	TCT	AAC	ATG	GAT	CCT	TAT	GTC	GTC	ACT	TCC	ATG	ATC	GCT	GAA	1047
BraGLN1.3_C2	GAC	ACT	GAG	AAA	GAA	GGC	AAA	GGT	TAC	TTC	GAG	GAT	CGA	AGG	CCA	GCT	TCT	AAC	ATG	GAT	CCT	TAT	GTC	GTC	ACT	TCC	ATG	ATC	GCT	GAA	1210
BnaGLN1.3_C6	GAC	ACT	GAG	AAA	GAA	GGC	AAA	GGT	TAC	TTC	GAG	GAT	CGT	AGG	CCA	GCT	TCT	AAT	ATG	GAT	CCT	TAT	GTC	GTC	ACT	TCC	ATG	ATC	GCT	GAA	1101
BolGLN1.3_C2	GAC	ACT	GAG	AAA	GAA	GGC	AAA	GGT	TAC	TTC	GAG	GAT	CGT	AGG	CCA	GCT	TCT	AAC	ATG	GAT	CCT	TAC	GTT	GTT	ACC	TCC	ATG	ATC	GCT	GAA	993
At5g16570_GLN1.4__cds	GAC	ACT	GAG	CAG	GCT	GGA	AAA	GGA	TAC	TTT	GAA	GAT	CGT	AGG	CCA	GCT	TCG	AAC	ATG	GAT	CCT	TAC	ACT	GTG	ACC	TCC	ATG	ATT	GCT	GAA	1047
BnaGLN1.4_C1	GAC	ACA	GAG	AAA	GAT	GGG	AAA	GGA	TAC	TTT	GAA	GAT	CGT	AGG	CCA	GCG	TCG	AAC	ATG	GAT	CCC	TAC	ACT	GTG	ACC	TCC	ATG	GTT	GCT	GAA	1089
JX306697	GAC	ACA	GAG	AAA	GAT	GGG	AAA	GGA	TAC	TTT	GAA	GAT	CGT	AGG	CCA	GCG	TCG	AAC	ATG	GAT	CCC	TAC	ACT	GTG	ACC	TCC	ATG	GTT	GCT	GAA	1064
JX306692	GAC	ACA	GAG	AAA	GAT	GGG	AAA	GGA	TAC	TTT	GAA	GAT	CGT	AGG	CCA	GCG	TCG	AAC	ATG	GAT	CCC	TAC	ACT	GTG	ACC	TCC	ATG	GTT	GCT	GAA	946
Bra023573_cds	GAC	ACA	GAG	AAA	GAG	GGG	AAA	GGA	TAC	TTT	GAA	GAT	CGT	AGG	CCA	GCG	TCG	AAC	ATG	GAT	CCC	TAC	ACT	GTG	ACC	TCC	ATG	GTT	GCT	GAA	1044
BraGLN1.4_C1	GAC	ACA	GAG	AAA	GAG	GGG	AAA	GGA	TAC	TTT	GAA	GAT	CGT	AGG	CCA	GCG	TCG	AAC	ATG	GAT	CCC	TAC	ACT	GTG	ACC	TCC	ATG	GTT	GCT	GAA	1169
BnaGLN1.4_C2	GAC	ACT	GAG	AAA	GAT	GGG	AAA	GGA	TAC	TTT	GAA	GAT	CGT	AGG	CCA	GCG	TCG	AAC	ATG	GAT	CCA	TAC	ACT	GTA	ACC	TCC	ATG	GTT	GCT	GAA	1114
JX306695	GAC	ACT	GAG	AAA	GAT	GGG	AAA	GGA	TAC	TTT	GAA	GAT	CGT	AGG	CCA	GCG	TCG	AAC	ATG	GAT	CCA	TAC	ACT	GTA	ACC	TCC	ATG	GTT	GCT	GAA	1088
JX306696	GAC	ACA	GAG	AAA	GAT	GGG	AAA	GGA	TAC	TTT	GAA	GAT	CGT	AGG	CCA	GCG	TCG	AAC	ATG	GAT	CCC	TAC	ACT	GTG	ACC	TCC	ATG	GTT	GCT	GAA	1065
BolGLN1.4_C1	GAC	ACT	GAG	AAA	GAT	GGG	AAA	GGA	TAC	TTT	GAA	GAT	CGT	AGG	CCA	GCG	TCG	AAC	ATG	GAT	CCA	TAC	ACT	GTA	ACC	TCC	ATG	GTT	GCT	GAA	1089
BnaGLN1.4_C4	GAC	ACT	GAG	CAA	GCT	GGG	AAA	GGG	TAC	TTC	GAA	GAT	CGT	AGG	CCA	GCG	TCC	AAC	ATG	GAT	CCC	TAC	ACT	GTG	ACC	TCC	ATG	ATT	GCT	GAA	1050
JX306700	GAC	ACT	GAG	CAA	GCT	GGG	AAA	GGG	TAC	TTC	GAA	GAT	CGT	AGG	CCA	GCG	TCC	AAC	ATG	GAT	CCC	TAC	ACT	GTG	ACC	TCC	ATG	ATT	GCT	GAA	726
JX306701	GAC	ACT	GAG	CAA	GCT	GGG	AAA	GGG	TAC	TTC	GAA	GAT	CGT	AGG	CCA	GCG	TCC	AAC	ATG	GAT	CCC	TAC	ACT	GTG	ACC	TCC	ATG	ATT	GCT	GAA	726

Bra008612_cds	GAC ACT GAG CAA GCT GGG AAA GGG TAC TTC GAA GAT CGT AGG CCA GCG TCC AAC ATG GAT CCC TAC ACT GTG ACC TCC ATG ATT GCT GAA	1047
BraGLN1.4_C2	GAC ACT GAG CAA GCT GGG AAA GGG TAC TTC GAA GAT CGT AGG CCA GCG TCC AAC ATG GAT CCC TAC ACT GTG ACC TCC ATG ATT GCT GAA	1092
BnaGLN1.4_C3	GAC ACT GAG CAA GCT GGG AAA GGG TAC TTT GAA GAT CGT AGG CCA GCG TCC AAC ATG GAT CCG TAC ACT GTG ACC TCC ATG ATT GCT GAA	1091
JX306698	GAC ACT GAG CAA GCT GGG AAA GGG TAC TTT GAA GAT CGT AGG CCA GCG TCC AAC ATG GAT CCG TAC ACT GTG ACC TCC ATG ATT GCT GAA	752
JX306699	GAC ACT GAG CAA GCT GGG AAA GGG TAC TTT GAA GAT CGT AGG CCA GCG TCC AAC ATG GAT CCG TAC ACT GTG ACC TCC ATG ATT GCT GAA	752
BolGln1.4_C2	GAC ACT GAG CAA GCT GGG AAA GGG TAC TTT GAA GAT CGT AGG CCA GCG TCC AAC ATG GAT CCG TAC ACT GTG ACC TCC ATG ATT GCT GAA	1051
At1g48470_GLN1.5__cds	GAT ACG GAG AAA GAA GGT AAA GGG TAT TTT GAA GAT CGA AGG CCT TCG TCT AAT ATG GAT CCT TAC CTA GTT ACC TCC ATG ATT GCT GAA	1047
BnaGLN1.5_C2	GAT ACG GAG AAA GAA GGG AAA GGT TAT TTT GAA GAT CGA AGA CCT TCG TCT AAT ATG GAT CCT TAT CTT GTT ACC TCC ATG ATA GCT GAA	1169
Bra018729_cds	GAT ACG GAG AAA GAA GGG AAA GGT TAT TTT GAA GAT CGA AGG CCT TCG TCT AAT ATG GAT CCT TAT CTT GTT ACC TCC ATG ATA GCT GAA	1000
BraGLN1.5_C1	--- ---	894
BnaGLN1.5_C1	GAT ACG GAG AAA GAA GGG AAA GGG TAT TTT GAA GAT CGA AGG CCT TCG TCT AAT ATG GAT CCT TAT CTT GTT ACC TCC ATG ATA GCT GAA	1144
JX306691	GAT ACG GAG AAA GAA GGG AAA GGG TAT TTT GAA GAT CGA AGG CCT TCG TCT AAT ATG GAT CCT TAT CTT GTT ACC TCC ATG ATA GCT GAA	816
BolGLN1.5_C1	--- ---	578

```
                        1 540          1 560          1 580          1 600          1 620
At5g37600_GLN1.1__cds  ACT ACA ATC CTC TGG AAT CCT TGA --- --- --- --- --- --- --- --- --- --- --- --- --- --- --- --- --- --- --- --- --- 1071
BnaGLN1.1_C1           ACC ACA ATC CTC TGG AAA CCT TGA TCA GAT CAA AGA AGC TTG TTG AAG AAT GTT CAC TCC GTT TGG GTT TCT TGC ATG GTT CAA CGT TTG 1225
X82997                 ACC ACA ATC CTC TGG AAA CCT TGA TCA GAT CAA AGA AGC TTG TTG AAG AAT GTT CAC TCC GTT TGG GTT TCT TGC ATG GTT CAA CGT TTG 1198
Bra028132_cds          ACC ACA ATC CTC TGG AAC CCT TGA --- --- --- --- --- --- --- --- --- --- --- --- --- --- --- --- --- --- --- --- --- 1071
BraGLN1.1_C1           ACC ACA ATC CTC TGG AAC CCT TGA TCA GAT CAA AGA AGA TTG TTG AAT G-- TTC ACT CCA TTT GGG TTT CTC TGC ATG ATT CAA CAT TTG 1226
BnaGLN1.1_C2           ACC ACA ATC CTC TGG AAA CCT TGA TCA GAT CAA AGA AGA TTG TTG AAG AAT GTT CAC TCC ATT TGG GTT TCT TGC ATG GTT CAA CGT TTG 1225
Y12460                 ACC ACA ATC CTC TGG AAA CCT TGA TCA GAT CAA AGA AGA TTG TTG AAG AAT GTT CAC TCC ATT TGC GTT TCT TGC ATG GTT CAA CGT TTG 1116
BolGLN1.1_C1           ACC ACA ATC CTC TGG AAA CCT TGA TCA GAT CAA AGA AGA TTG TTG AAG AAT GTT CAC TCC ATT TGG GTT TCT TGC ATG GTT CAA CGT TTG 1219
At1g66200.1_GLN1.2__cds ACT ACA CTC CTC TGG AAC CCT TGA --- --- --- --- --- --- --- --- --- --- --- --- --- --- --- --- --- --- --- --- --- 1071
BnaGLN1.2_C1           ACT ACA CTT CTT TGG AAT CCT TGA AGA AAT ATG ACA ATA ATA TAA CTT GAA TCT GGT TTC AAG TTT GTG TTT CTA CAG TTT ATT AAG CAA 1223
X76736                 ACT ACA CTT CTT TGG AAT CCT TGA AGA AAT ATG ACA ATA ATA TAA CTT GAA TCT GGT TTC AAG TTT GTG TTT CTA CAG TTT ATT AAG CAA 1204
Bra039756_cds          ACT ACA CTT CTT TGG AAT CCT TGA --- --- --- --- --- --- --- --- --- --- --- --- --- --- --- --- --- --- --- --- --- 1071
BraGLN1.2_C1           ACT ACA CTT CTT TGG AAT CCT TGA AGA AAT ATG ACA ATA ATA TAA CTT GAA TCT GGT TTC AAG TTT GTG TTT CTA CAG TTT ATT AAG CAA 1219
EU499383               ACT ACA CTT CTT TGG AAT CCT TGA AGA AAT ATG ACA ATA ATA TAA CTT GAA TCT GGT TTC AAG TTT GTG TTT CTA CAG TTT ATT AAG CAA 1221
AY773089               ACT ACA CTT CTT TGG AAT CCT TGA AGA AAT ATG ACA ATA ATA TAA CTT GAA TCT GGT TTC AAG TTT GTG TTT CTA CAG TTT ATT AAG CAA 1225
BnaGLN1.2_C2           ACT ACA CTT CTT TGG AAT CCT TGA AGA AAT ATG ACA ATA ATA TAA CTT GAA TCT GGT TCT CTG GGG CTT CAA GTT TGT GTT TTC TAC AGT 1226
Y12459                 ACT ACA CTT CTT TGG AAT CCT TGA AGA AAT ATG ACA ATA ATA TAA CTT GAA TCT GGT TCT CTG GGG CTT CAA GTT TGT GTT TTC TAC AGT 1168
BolGLN1.2_C1           ACT ACA CTT CTT TGG AAT CCT TGA AGA AAT ATG ACA ATA ATA TAA CTT GAA TCT GGT TCT CTG GGG CTT CAA GTT TGT GTT TTC TAC AGT 1116
EU822334               ACT ACA CTT CTT TGG AAT CCT TGA AGA AAT ATG ACA ATA ATA TAA CTT GAA TCT GGT TCT CTG GGG CTT CAA GTT TGT GTT T-- --- --- 1157
EU822335               ACT ACA CTT CTT TGG AAT CCT TGA AGA AAT ATG ACA ATA ATA TAA CTT GAA TCT GGT TCT CTG GGG CTT CAA GTT TGT --- --- --- --- 1150
At3g17820_GLN1.3__cds  ACG ACC ATA CTC GGT --- --- TGA --- --- --- --- --- --- --- --- --- --- --- --- --- --- --- --- --- --- --- --- --- 1065
BnaGLN1.3_C2           ACC ACC ATC CTC GGT --- --- TAA ACC ACA CAC ATT TAG TAA TAT TTG ATT TTC TCC GGT TTG GTT TTA TGA TTT GAA TAC TGT TGT ACT 1278
JX306693               ACC ACC ATC CTC GGT --- --- TAA ACC ACA CAC ATT TAG TAA TAT TTG ATT TTC TCC GGT TTG GTT TTA TGA TTT GAA TAC TGT TGT ACT 1146
Bra022247_cds          ACC ACC ATC CTC GGT --- --- TAA --- --- --- --- --- --- --- --- --- --- --- --- --- --- --- --- --- --- --- --- --- 1065
BraGLN1.3_C1           ACC ACC ATC CTC GGT --- --- TAA TCG CAC ACG TTT TAA TGT TTG GTT TTA TCC GGT CTG GTT TTC AAT TTG CAA AAC TTG TGC TTG TGG 1201
BnaGLN1.3_C1           ACC ACC ATC CTC GGT --- --- TAA AAT CGC ACA CGT TCT TAT GTT TGG GTT TAT CTT ATC TGG TCT GGT TTT CAA TTT GCA AAC TTG TGC 1198
JX306690               ACC ACC ATC CTC GGT --- --- TAA AAT CGC ACA CGT TCT TAT GTT TGG GTT TAT CTT ATC TGG TCT GGT TTT CAA TTT GCA AAC TTG TGC 1165
BolGLN1.3_C1           ACC ACC ATC CTC GGT --- --- TAA -AT CGC ACA CGT TCT TAT GTT TGT GTT TAT CCG GGC TGG TTT TCA ATT TG- --- --- --- --- --- 1156
BnaGLN1.3_C4           ACC ACA ATC CTC GGT --- --- TAA GTA ATC AAT ACG CAT TTT AAT GTT TAG TTT TTT CCT TAA TTT GCG GAT TTT TGT GCT TGT GGT TGC 1200
Bra021276_cds          ACC ACA ATC CTC GGT --- --- TAA --- --- --- --- --- --- --- --- --- --- --- --- --- --- --- --- --- --- --- --- --- 1065
BraGLN1.3_C3           --- --- --- --- --- --- --- --- --- --- --- --- --- --- --- --- --- --- --- --- --- --- --- --- --- --- --- --- --- 972
BnaGLN1.3_C3           ACC ACA ATC CTC GGT --- --- TAA GTA ATC GCA CGC ATT TTA TAA TGT TTG GTT TAT CCG GTT TCG TTT ATT TCT TAA TTT GCG GAT TTT 1158
BnaGLN1.3_C5           ACC ACC ATC CTC GGT --- --- TAA TCA GAA TGT TTC TCT AAT TGC ACT AAT AAT TCT TAT CAT GGA CTT AGT AGC TCG ACG GTG TTT GGT 1212
JX306694               ACC ACC ATC CTC GGT --- --- TAA TCA GAA TGT TTC TCT AAT TGC ACT AAT AAT TCT TAT CAT GGA CTT AGT AGC TCG ACG GTG TTT GGT 1187
Bra001686_cds          ACC ACC ATC CTC GGT --- --- TAA --- --- --- --- --- --- --- --- --- --- --- --- --- --- --- --- --- --- --- --- --- 1065
BraGLN1.3_C2           ACC ACC ATC CTC GGT --- --- TAA TCA GAA TGT TTC TCT AAT TGC ACT AAT AAT TCT TAT CAT GGA CTT AGT AGC TCG ACG GTG TTT G-- 1292
BnaGLN1.3_C6           ACC ACC ATC CTC GGT --- --- TAA TCA CAA TGT TTT TCT AAT TGC ACT AAT AAT TCT TAT CAT GGA CTC AGT AGC ACG ACG GTG TTT GGT 1185
BolGLN1.3_C2           ACC ACC ATC CTC GCT --- --- TAA TCA GCA TGT GCT TTT ATT TCG TTT CAA ATA ATT CTT ATC GCG GCC TAA GTA GTA AAA CGA CGT TTT 1077
At5g16570_GLN1.4__cds  TCC ACA ATC CTT TGG AAA CCA TGA --- --- --- --- --- --- --- --- --- --- --- --- --- --- --- --- --- --- --- --- --- 1071
BnaGLN1.4_C1           ACC ACA ATC CTC TGG AAA CCA TGA AGG AAG AAA CCT TGA GTC TCA AGG AAC CTC TTA TAT CAG TTC ATG TTG ATT CTT CTA TTG TCT ATT 1179
JX306697               ACC ACA ATC CTC TGG AAA CCA TGA AGG AAG AAA CCT TGA --- --- --- --- --- --- --- --- --- --- --- --- --- --- --- --- 1103
JX306692               ACC ACA ATC CTC TGG AAA CCA TGA AGG AAG AAA CCT TGA GTC TCA AGG AAC CTC TTA TAT CAG TTC ATG TTG ATT CTT CTA TTG TCT ATT 1036
Bra023573_cds          ACC ACA ATC CTC TGG AAA CCA TGA --- --- --- --- --- --- --- --- --- --- --- --- --- --- --- --- --- --- --- --- --- 1068
BraGLN1.4_C1           ACC ACA ATC CTC TGG AAA CCA TGA AGG AAG AAA CCT TGA GTC TCA AGG AAC CTC TTA TAT CAG TTC ATG TTG ATT CTT CTA TTG TCT ATT 1259
BnaGLN1.4_C2           ACC ACA ATC CTC TGG AAA CCA TGA ATG AGG AAT AGA CAA TAG AAG AAT CAA TAT GAA ACA GTT CTC ATG TGT TCT TTG TTT AAG AAT GTT 1204
JX306695               ACC ACA GTC CTC TGG AAA CCA TGA ATG AGG AAT AGA CAA TAG AAG AAT CAA TAT GAA ACA GTT CTC ATG TGT TCT TTG TTT AAG AAT GTT 1178
JX306696               ACC ACA ATC CTC TGG AAA CCA TGA AGG AAG AAA CCT TGA --- --- --- --- --- --- --- --- --- --- --- --- --- --- --- --- 1104
BolGLN1.4_C1           ACC ACA ATC CTC TGG AAA CCA TGA ATG AGG AAT AGA CAA TAG AAG AAT CAA TAT GAA AGA GTT CTC ATG TG- --- --- --- --- --- --- 1160
BnaGLN1.4_C4           ACT ACA ATC CTC TGG AAG CCA TGA ATG AAG AAA ACT TGA GCT CCT CCA AGG A-- --- --- --- --- --- --- --- --- --- --- --- 1102
JX306700               ACT ACA ATC CTC TGG AAG CCA TGA ATG AAG AA- --- --- --- --- --- --- --- --- --- --- --- --- --- --- --- --- --- --- 758
JX306701               ACT ACA ATC CTC TGG AAG CCA TGA ATG AAG AA- --- --- --- --- --- --- --- --- --- --- --- --- --- --- --- --- --- --- 758
```

```
Bra008612_cds        ACT ACA ATC CTC TGG AAG CCA TGA --- --- --- --- --- --- --- --- --- --- --- --- --- --- --- --- --- --- --- --- --- --- 1071
BraGLN1.4_C2         ACT ACA ATC CTC TGG AAG CCA TGA ATG AAG AAA ACT TGA GCT CCT CCA AGG AAC CTC TAA TAT CAG TTC ATG TTC ATT CTT CTA TTG TCT 1182
BnaGLN1.4_C3         ACT ACA ATC CTC TGG AAG CCA TGA ATG AAG AA- --- --- --- --- --- --- --- --- --- --- --- --- --- --- --- --- --- --- --- 1123
JX306698             ACT ACA ATC CTC TGG AAG CCA TGA ATG AAG AA- --- --- --- --- --- --- --- --- --- --- --- --- --- --- --- --- --- --- --- 784
JX306699             ACT ACA ATC CTC TGG AAG CCA TGA ATG AAG AA- --- --- --- --- --- --- --- --- --- --- --- --- --- --- --- --- --- --- --- 784
BolGln1.4_C2         ACT ACA ATC CTC TGG AAG CCA TGA ATG AAG AAA ACT CGA GCT TCA AGG AAC CTC TAA TAT CAG TTC ATG TGT CAT TCT TAT ATT GTC TCG 1141
At1g48470_GLN1.5__cds ACC ACC ATC CTC --- --- --- TAA --- --- --- --- --- --- --- --- --- --- --- --- --- --- --- --- --- --- --- --- --- --- 1062
BnaGLN1.5_C2         ACC ACC ATC CTC GGC --- --- TAA GCT TTC TTT TGA AGT TGT TGC ATA CGT TCT TTT GTT TCT TCA TGT TTC GGT TTA ATT TCG GTT TGA 1253
Bra018729_cds        ACC ACC ATC CTC GGC --- --- TAA --- --- --- --- --- --- --- --- --- --- --- --- --- --- --- --- --- --- --- --- --- --- 1018
BraGLN1.5_C1         --- --- --- --- --- --- --- --- --- --- --- --- --- --- --- --- --- --- --- --- --- --- --- --- --- --- --- --- --- --- 894
BnaGLN1.5_C1         ACC ACC ATC CTC GGC --- --- TAA GCT TTC GTT TGA AGT TGT TGC ATA CGT TCT TTT GTT TCT TCA TGT TTC GGT TTA ATT TCA GTT TGA 1228
JX306691             ACC ACC ATC CTC GGC --- --- TAA GCT TTC GTT TGA AGT TGT TGC ATA CGT TCT TTT GTT TCT TCA TGT TTC GGT TTA ATT TCA GTT TGA 900
BolGLN1.5_C1         --- --- --- --- --- --- --- --- --- --- --- --- --- --- --- --- --- --- --- --- --- --- --- --- --- --- --- --- --- --- 578
```

BnaGLN1_JXB

```
                                                   1 640                                                  1 660                                                  1 680                                                  1 700
At5g37600_GLN1.1__cds --- --- --- --- --- --- --- --- --- --- --- --- --- --- --- --- --- --- --- --- --- --- --- --- --- --- --- --- --- --- 1071
BnaGLN1.1_C1 TGT GTT TCT CTA TCA AGC ATT GTC TCA GAG CAA GTC AA- GAG ATT TGC TTT GTT CTT ATG GCT TTT ATT GTT TCA CAT CCA TTG AAA ACA 1314
X82997 TGT GTT TCT CTA TCA AGC ATT GTC TCA GAG CAA GTC AA- GAG ATT TGC TTT GTT CTT ATG GCT TTT ATT GTT TCA CAT CCA TTG AAA ACA 1287
Bra028132_cds --- --- --- --- --- --- --- --- --- --- --- --- --- --- --- --- --- --- --- --- --- --- --- --- --- --- --- --- --- --- 1071
BraGLN1.1_C1 TGT GTT TCT CTA TCA AGC ATT GTC TCA GAG CAA GTC AA- GAG ATT TGC TCT GTT CTT ATG GCT TTT ATT GTT TCA CAT CCA TTG GAA ACA 1315
BnaGLN1.1_C2 TAT GTT TCT CTA TCA AGC ATT GTC TCA GAA CAA AGT CAA GAG ATT TGC TCT GTT CTT ATG GCT TTT ATT GTT TCA CAT CCA TTG AAA ACA 1315
Y12460 TAT GTT TCT CTA TCA AGC ATT GTC TCG GAA CAA GTC AA- GAT ATT TGT TCT GTT CTT ATG GCT TTT ATT GTT TCA CAT CCA TTG AAA ACA 1205
BolGLN1.1_C1 TGT GTT TCT CTA TCA AGC ATT GTC TCA GAA CAA AGT CAA GAG ATT TGC TCT GTT CTT ATG GCT TTT ATT GTT TCA CAT CCA TTG AAA ACA 1309
At1g66200.1_GLN1.2__cds --- --- --- --- --- --- --- --- --- --- --- --- --- --- --- --- --- --- --- --- --- --- --- --- --- --- --- --- --- --- 1071
BnaGLN1.2_C1 TTA CCG GGT TGA TAC TGC CGG AGT TTG TGG TTT GAG GC- CTT TCT TTT AAT CTC TTT GTG TTT TGG GGT TTG TGA TTG GAG CAA AAG CCC 1312
X76736 TTA CCG GGT TGA TAC TGC CGG AGT TTG TGG TTT GAG GC- CTT TCT TTT AAT CTC TTT GTG TTT TGG GGT TTG TGA TTG GAG CAA AAG CCC 1293
Bra039756_cds --- --- --- --- --- --- --- --- --- --- --- --- --- --- --- --- --- --- --- --- --- --- --- --- --- --- --- --- --- --- 1071
BraGLN1.2_C1 TTA CCG GGT TGA TAC TGC CGG AGT TTG TGG TTT GAG GC- CTT TCT TTT AAT CTC TTT GTG TTT TGG GGT TTG TGA TTG GAG CAA AAA CCC 1308
EU499383 TTA CCG GGT TGA TAC TGC CGG AGT TTG TGG TTT GAG GC- CTT TCT TTT AAT CTC TTT GTG TTT TGG GGT TTG TGA TTG GAG CAA AAG CCC 1310
AY773089 TTA CCG GGT TGA TAC TGC CGG AGT TTG TGG TTT GAG GC- CTT TCT TTT AAT CTC TTT GTG TTT TGG GGT TTG TGA TTG GAG CAA AAA CCC 1314
BnaGLN1.2_C2 TTA TTA AGC AAT TAC CGG GTT GAT ACT GCC AGA GTT TGT GGT TTG AGG CCT TTC TTT TAA TCT CTT TGT GTT TTG GGG TTT GTG ATT GAA 1316
Y12459 TTA TTA AGC AAT TAC CGG GTT GAT ACT GCC GGA GTT TG- TGG TTT GAG GCT TTC TTT TAA TCT CTT TGT GTT TTG GGG TTT GTG ATT GAA 1257
BolGLN1.2_C1 TTA TTA AGC AAT TAC CGG GTT GAT ACT GCC AGA GTT TGT GGT TTG AGG CCT TTC TTT TAA TCT CTT TGT GTT TTG GGG TTT GTG ATT GAA 1206
EU822334 --- --- --- --- --- --- --- --- --- --- --- --- --- --- --- --- --- --- --- --- --- --- --- --- --- --- --- --- --- --- 1157
EU822335 --- --- --- --- --- --- --- --- --- --- --- --- --- --- --- --- --- --- --- --- --- --- --- --- --- --- --- --- --- --- 1150
At3g17820_GLN1.3__cds --- --- --- --- --- --- --- --- --- --- --- --- --- --- --- --- --- --- --- --- --- --- --- --- --- --- --- --- --- --- 1065
BnaGLN1.3_C2 TGT GAT CGC GGT CTA TTT CGG TTT CAA TAA TTC TTA TGA CGA AAA AAA AAA AAA AAA A-- --- --- --- --- --- --- --- --- --- --- 1336
JX306693 TGT GAT CGC GGT CTA TTT CGG TTT CAA TAA TTC TTA TGA CGA CAA AAA AAA AAA AAA AAA AAA AAA AAA AAA --- --- --- --- --- --- 1218
Bra022247_cds --- --- --- --- --- --- --- --- --- --- --- --- --- --- --- --- --- --- --- --- --- --- --- --- --- --- --- --- --- --- 1065
BraGLN1.3_C1 TTG TGG TCT ATT TCG GTT TAA ATA ATT CTT ATG ACG ACA TTT TGT GTT TTT TCT TCT TCT TAG TTT CGA ATA ATT AAA TAA GGG TAT TCA 1291
BnaGLN1.3_C1 TTG TGA TTG CCG GTT TAT CTC GGT TTA AAT AAT TCT TAT GAC GAC ATT TTG TAT TTT TTT TCT TCC TAG TTT CGA AAT AAT TAA ATA AGG 1288
JX306690 TTG TGA TTG CCG GTT TAT CTC GGT TTA AAT AAT TCT TAT GAC GAC WTT TTG TAT TTT TTT TCT TCC TAG TTT CGA ATA ATT AAA TAA GGT 1255
BolGLN1.3_C1 --- --- --- --- --- --- --- --- --- --- --- --- --- --- --- --- --- --- --- --- --- --- --- --- --- --- --- --- --- --- 1156
BnaGLN1.3_C4 ACT ACT AAT AAT TAT TAT CAT GCC CTA GTA GGA TTC ACG TGT TTT ATG TTT TTT TTT TTC ATT TCG AAT AAT T-- --- --- --- --- --- 1273
Bra021276_cds --- --- --- --- --- --- --- --- --- --- --- --- --- --- --- --- --- --- --- --- --- --- --- --- --- --- --- --- --- --- 1065
BraGLN1.3_C3 --- --- --- --- --- --- --- --- --- --- --- --- --- --- --- --- --- --- --- --- --- --- --- --- --- --- --- --- --- --- 972
BnaGLN1.3_C3 TGT GCT TGT GGT TGC ACT ACT AAT AAT TAT TAT CAT GCC CTA GTA GGA TTC ACG TGT TTT ATG TTT TTT TTT CAT TTC GAA TAA TTA AAT 1248
BnaGLN1.3_C5 GTG TTT CGT TTC GAA TAA TTA AAT AAA GGA TTT TCA TGA TG- --- --- --- --- --- --- --- --- --- --- --- --- --- --- --- --- 1253
JX306694 GTG TTT CGT TTC GAA TAA TTA AAT AAA GGA TTT TCA TGA TGT AAA AAA AAA AAA AAA AAA AAA AAA AAA A-- --- --- --- --- --- --- 1257
Bra001686_cds --- --- --- --- --- --- --- --- --- --- --- --- --- --- --- --- --- --- --- --- --- --- --- --- --- --- --- --- --- --- 1065
BraGLN1.3_C2 --- --- --- --- --- --- --- --- --- --- --- --- --- --- --- --- --- --- --- --- --- --- --- --- --- --- --- --- --- --- 1292
BnaGLN1.3_C6 GTG TTT CGT TTC GAA TAA TTA AAT AAA GGA TTT TCA TGA TGA AAA AAA AAA AAA AAA AAA --- --- --- --- --- --- --- --- --- --- 1245
BolGLN1.3_C2 ATG TGT GCC GTT TCA AAT AAG TAA ACA ATG GAT TAT TAA TGT GAA AAT TGC CCT TTT AAT --- --- --- --- --- --- --- --- --- --- 1137
At5g16570_GLN1.4__cds --- --- --- --- --- --- --- --- --- --- --- --- --- --- --- --- --- --- --- --- --- --- --- --- --- --- --- --- --- --- 1071
BnaGLN1.4_C1 CCT CTT TAT GAA ACA CTT CTC ATG TGT TCT TTG TTT AAG AAT GTT TGA TTT AAA CTC TTT CCA AGA AAT AAT AGT AGT TCT TTT CCC TAA 1269
JX306697 --- --- --- --- --- --- --- --- --- --- --- --- --- --- --- --- --- --- --- --- --- --- --- --- --- --- --- --- --- --- 1103
JX306692 CCT CTT TAT GAA ACA CTT CTC ATG TGT TCT TTG TTT AAG AAT GTT TGA TTT AAA CTC TTT CCA AGA AAT AAT AGT AGT TCT TTT CCC TAA 1126
Bra023573_cds --- --- --- --- --- --- --- --- --- --- --- --- --- --- --- --- --- --- --- --- --- --- --- --- --- --- --- --- --- --- 1068
BraGLN1.4_C1 CCT CTT TAT GAA ACA CTT CTC ATG TGT TCT TTG TTT AAG AAT GTT TGA TTT AAA CTA TTT CCA AGA AAT AAT AGT AGT TCT TTT CCC TGA 1349
BnaGLN1.4_C2 TGA TTT AAA CTC TTT CCA AGA AAT AAT GAT AGT CTT TCT CTA GCT TTT ACT TTG T-- --- --- --- --- --- --- --- --- --- --- --- 1259
JX306695 TGA TTT AAA CTC TTT CCA AGA AAT AAT GAT AGT CTT TTC TCT AGC TTT TAC TTT GTT GTA TTT CAG AAT CTA TAT GTA TTT ACA AGA TTC 1268
JX306696 --- --- --- --- --- --- --- --- --- --- --- --- --- --- --- --- --- --- --- --- --- --- --- --- --- --- --- --- --- --- 1104
BolGLN1.4_C1 --- --- --- --- --- --- --- --- --- --- --- --- --- --- --- --- --- --- --- --- --- --- --- --- --- --- --- --- --- --- 1160
BnaGLN1.4_C4 --- --- --- --- --- --- --- --- --- --- --- --- --- --- --- --- --- --- --- --- --- --- --- --- --- --- --- --- --- --- 1102
JX306700 --- --- --- --- --- --- --- --- --- --- --- --- --- --- --- --- --- --- --- --- --- --- --- --- --- --- --- --- --- --- 758
JX306701 --- --- --- --- --- --- --- --- --- --- --- --- --- --- --- --- --- --- --- --- --- --- --- --- --- --- --- --- --- --- 758
```

```
Bra008612_cds          --- --- --- --- --- --- --- --- --- --- --- --- --- --- --- --- --- --- --- --- --- --- --- --- --- --- --- --- --- --- 1071
BraGLN1.4_C2           CGT TCT GTC TTC TTT GTT TAA GTA TGT TTG ATT TAA ACT CTT AGA ACT TGT TCT AAA AAA AAA A-- --- --- --- --- --- --- --- --- 1246
BnaGLN1.4_C3           --- --- --- --- --- --- --- --- --- --- --- --- --- --- --- --- --- --- --- --- --- --- --- --- --- --- --- --- --- --- 1123
JX306698               --- --- --- --- --- --- --- --- --- --- --- --- --- --- --- --- --- --- --- --- --- --- --- --- --- --- --- --- --- --- 784
JX306699               --- --- --- --- --- --- --- --- --- --- --- --- --- --- --- --- --- --- --- --- --- --- --- --- --- --- --- --- --- --- 784
BolGln1.4_C2           TTC TGT --- --- --- --- --- --- --- --- --- --- --- --- --- --- --- --- --- --- --- --- --- --- --- --- --- --- --- --- 1147
At1g48470_GLN1.5__cds  --- --- --- --- --- --- --- --- --- --- --- --- --- --- --- --- --- --- --- --- --- --- --- --- --- --- --- --- --- --- 1062
BnaGLN1.5_C2           GAC CTT TTT TTT TAG TGC TAA TAA TTC ATG GGA TGG TCT TGA TCC TAT TGT TTG TTT ATC CTG GTT CAG TTG TTA GTG TTA AAC AAA ATT 1343
Bra018729_cds          --- --- --- --- --- --- --- --- --- --- --- --- --- --- --- --- --- --- --- --- --- --- --- --- --- --- --- --- --- --- 1018
BraGLN1.5_C1           --- --- --- --- --- --- --- --- --- --- --- --- --- --- --- --- --- --- --- --- --- --- --- --- --- --- --- --- --- --- 894
BnaGLN1.5_C1           GAC TCT TTT TTT TAT TGC TAA TAA TTC ATG GGA TGG TCT TGA TCC TAT TGT TTG TTT ATC CTG GTT CAG TTG TTT GTG TTA AAA CAA AAT 1318
JX306691               GAC TCT TAA AAA AAA AAA AAA AAA AAA AAA AAA AAA AAA AAA AAA AAA AA- --- --- --- --- --- --- --- --- --- --- --- --- --- 950
BolGLN1.5_C1           --- --- --- --- --- --- --- --- --- --- --- --- --- --- --- --- --- --- --- --- --- --- --- --- --- --- --- --- --- --- 578
```

1720 1740 1760 1780 1800

At5g37600_GLN1.1__cds	--- ---	1071
BnaGLN1.1_C1	TCT CTT TGT ATC AAT TTA TGA ATA AAG TGT GGT TTG GTT CTA AAA AAA AAA AAA AAA AAA --- --- --- --- --- --- --- --- --- ---	1374
X82997	TCT CTT TGT ATC AAT TTA TGA ATA AAG TGT GGT TTG GTT AAA AAA AAA AAA AAA AAA AAA AAA AAA A-- --- --- --- --- --- --- ---	1354
Bra028132_cds	--- ---	1071
BraGLN1.1_C1	T-- CTT TGT ATC AAT TTA TGA ATA AAG TTT GGT TTG GTT AAA AAA AAA A-- --- --- --- --- --- --- --- --- --- --- --- --- ---	1362
BnaGLN1.1_C2	TCT CTT TGT ATC AAT TTA TGA ATA AAG TGT GGT TTG GTT CAA AAA AAA AAA A-- --- --- --- --- --- --- --- --- --- --- --- ---	1367
Y12460	TCT CTT TGT ATC AAT TTA TGA ATA AAG TGT GGT TTG GTT CAA AAA AAA AAA AAA AAA AA- --- --- --- --- --- --- --- --- --- ---	1264
BolGLN1.1_C1	TCT CTT TGT ATC AAT TTA TGA ATA AAG TGT GGT TTG GTT --- --- --- --- --- --- --- --- --- --- --- --- --- --- --- --- ---	1348
At1g66200.1_GLN1.2__cds	--- ---	1071
BnaGLN1.2_C1	TGA TTT GCT CTG TTT CTT TGA CCT TTT ATT TGA ACC CTT TGT ATT TGT ATT AAT AAG ACG ATC TGA AAA GGC CTT TTC ATG TTT CAA AAA	1402
X76736	TGA TTT GCT CTG TTT CTT TGA CCT TTT ATT TGA ACC CTT TGT ATT TGT ATT AAT AAG GCG ATC TGA AAA GGC CTT TTC ATG TTT CTA TCA	1383
Bra039756_cds	--- ---	1071
BraGLN1.2_C1	TGA TTT GCT CTG TTT CTT TGA CCT TTT ATT TGA ACC CTT TGT ATT TGA ATT AAT AAG ACG ATC TGA AAA GGC CTT TTC ATG TTT CTA TCA	1398
EU499383	TGA TTT GCT CTG TTT CTT TGA CCT TTT ATT TGA ACC CTT TGT ATT TGT ATT AAT AAG GCG ATC TGA AAA GGC CTT TTC ATG TTT CTA AAA	1400
AY773089	TGA TTT GCT CTG TTT CTT TGA CCT TTT ATT TGA ACC CTT TGT ATT TGA ATT AAT AAG ACG ATC TGA AAA GGC CTT TTC ATG TTT TAA AAA	1404
BnaGLN1.2_C2	GCA AAA ACC CTG ATT TGC TCT GTT TCT TTG ACC TTT TAT TTG AAC CCT TTG TAT TTC TAT TAA TAA GAC GAT CTG AGA AGG CCT TTC CAT	1406
Y12459	GCA AAA ACC CTG ATT TGC TCT GTT TCT TTG ACC TTT TAT TTG AAC CCT TTG TAT TTC TAT TAA TAA GAC GAT CTG AGA AGG CCA AAA ---	1344
BolGLN1.2_C1	GCA AAA ACC CTG ATT TGC TCT GTT TCT TTG ACC TTT TAT TTG AAC CCT TTG TAT TTC TAT TAA TAA GAC GAT CTG AGA AGG CCT --- ---	1290
EU822334	--- ---	1157
EU822335	--- ---	1150
At3g17820_GLN1.3__cds	--- ---	1065
BnaGLN1.3_C2	--- ---	1336
JX306693	--- ---	1218
Bra022247_cds	--- ---	1065
BraGLN1.3_C1	TGC GGT GA- ---	1299
BnaGLN1.3_C1	TAT CCA AAT CGC CCT ATA GTG AGT CGT ATT ACA ATT CAA CTG GCC GTC GTT TTT ACA ACG TCG TGA CTG GGA AAA CCC CTG CGG TTA CCC	1378
JX306690	ATT CAT AAA AAA AAA AAA AAA AAA ---	1279
BolGLN1.3_C1	--- ---	1156
BnaGLN1.3_C4	--- ---	1273
Bra021276_cds	--- ---	1065
BraGLN1.3_C3	--- ---	972
BnaGLN1.3_C3	AAG GAA TTC TTA TGG ATG AAA TTT AAA ---	1275
BnaGLN1.3_C5	--- ---	1253
JX306694	--- ---	1257
Bra001686_cds	--- ---	1065
BraGLN1.3_C2	--- ---	1292
BnaGLN1.3_C6	--- ---	1245
BolGLN1.3_C2	--- ---	1137
At5g16570_GLN1.4__cds	--- ---	1071
BnaGLN1.4_C1	AAA A-- ---	1273
JX306697	--- ---	1103
JX306692	AAA AAA AAA AAA AAA AAA AAA AAA AAA AAA AAA AAA AAA AAA AAA AAA AAA A-- --- --- --- --- --- --- --- --- --- --- --- ---	1178
Bra023573_cds	--- ---	1068
BraGLN1.4_C1	ACA AGA GAC AGA TTC TGA AGT ATC TCC TCT GCC TCC TGA AGA AGC ATG AGA AAA TCA TAT GGA GAG ATC ACA AAG ATA ACT CTC TCA CAC	1439
BnaGLN1.4_C2	--- ---	1259
JX306695	AAG TCT CGG TCA AAA AAA AAA AAA AAA AAA AAA AAA AAA AA- --- --- --- --- --- --- --- --- --- --- --- --- --- --- --- ---	1309
JX306696	--- ---	1104
BolGLN1.4_C1	--- ---	1160
BnaGLN1.4_C4	--- ---	1102
JX306700	--- ---	758
JX306701	--- ---	758

```
Bra008612_cds       --- --- --- --- --- --- --- --- --- --- --- --- --- --- --- --- --- --- --- --- --- --- --- --- --- --- --- --- --- --- 1071
BraGLN1.4_C2        --- --- --- --- --- --- --- --- --- --- --- --- --- --- --- --- --- --- --- --- --- --- --- --- --- --- --- --- --- --- 1246
BnaGLN1.4_C3        --- --- --- --- --- --- --- --- --- --- --- --- --- --- --- --- --- --- --- --- --- --- --- --- --- --- --- --- --- --- 1123
JX306698            --- --- --- --- --- --- --- --- --- --- --- --- --- --- --- --- --- --- --- --- --- --- --- --- --- --- --- --- --- --- 784
JX306699            --- --- --- --- --- --- --- --- --- --- --- --- --- --- --- --- --- --- --- --- --- --- --- --- --- --- --- --- --- --- 784
BolGln1.4_C2        --- --- --- --- --- --- --- --- --- --- --- --- --- --- --- --- --- --- --- --- --- --- --- --- --- --- --- --- --- --- 1147
At1g48470_GLN1.5__cds --- --- --- --- --- --- --- --- --- --- --- --- --- --- --- --- --- --- --- --- --- --- --- --- --- --- --- --- --- --- 1062
BnaGLN1.5_C2        GAA TTG GGA AAA TAA AGG TTC TTA GTT CTT ACT TTT TAA AAA AAA AAA A-- --- --- --- --- --- --- --- --- --- --- --- --- --- 1392
Bra018729_cds       --- --- --- --- --- --- --- --- --- --- --- --- --- --- --- --- --- --- --- --- --- --- --- --- --- --- --- --- --- --- 1018
BraGLN1.5_C1        --- --- --- --- --- --- --- --- --- --- --- --- --- --- --- --- --- --- --- --- --- --- --- --- --- --- --- --- --- --- 894
BnaGLN1.5_C1        TGA ATT GGG AAA ATT GAA TTG GCA AAA TAA AGG TTC TTA GTT CTT ACT TTT AAA AAA AAA AA- --- --- --- --- --- --- --- --- --- 1380
JX306691            --- --- --- --- --- --- --- --- --- --- --- --- --- --- --- --- --- --- --- --- --- --- --- --- --- --- --- --- --- --- 950
BolGLN1.5_C1        --- --- --- --- --- --- --- --- --- --- --- --- --- --- --- --- --- --- --- --- --- --- --- --- --- --- --- --- --- --- 578
```

BnaGLN1_JXB

1 820 | 1 840 | 1 860 | 1 880

At5g37600_GLN1.1__cds	--- ---	1071
BnaGLN1.1_C1	--- ---	1374
X82997	--- ---	1354
Bra028132_cds	--- ---	1071
BraGLN1.1_C1	--- ---	1362
BnaGLN1.1_C2	--- ---	1367
Y12460	--- ---	1264
BolGLN1.1_C1	--- ---	1348
At1g66200.1_GLN1.2__cds	--- ---	1071
BnaGLN1.2_C1	AAA AAA AAA AAA AAA AAA AAA AAA AAA A-- ---	1430
X76736	AAA AAA AAA AAA AAA AA- ---	1400
Bra039756_cds	--- ---	1071
BraGLN1.2_C1	AAA AAA AAA AAA AAA AAA ---	1416
EU499383	AAA AAA AAA AAA A-- ---	1413
AY773089	AAA AAA AAA AAA AAA AAA AAA A-- ---	1426
BnaGLN1.2_C2	GTT CCA AAA AAA AAA AAA AAA AAA A-- ---	1431
Y12459	--- ---	1344
BolGLN1.2_C1	--- ---	1290
EU822334	--- ---	1157
EU822335	--- ---	1150
At3g17820_GLN1.3__cds	--- ---	1065
BnaGLN1.3_C2	--- ---	1336
JX306693	--- ---	1218
Bra022247_cds	--- ---	1065
BraGLN1.3_C1	--- ---	1299
BnaGLN1.3_C1	ACC TTA AAT CGC CTT GCA GCA CAA TTC CCC TTT TCG CAG CTT GGC GTA ATT AAC GAA AAG GCC CGG AAC CGA TGG CCC TTT TCC AAC GTT	1468
JX306690	--- ---	1279
BolGLN1.3_C1	--- ---	1156
BnaGLN1.3_C4	--- ---	1273
Bra021276_cds	--- ---	1065
BraGLN1.3_C3	--- ---	972
BnaGLN1.3_C3	--- ---	1275
BnaGLN1.3_C5	--- ---	1253
JX306694	--- ---	1257
Bra001686_cds	--- ---	1065
BraGLN1.3_C2	--- ---	1292
BnaGLN1.3_C6	--- ---	1245
BolGLN1.3_C2	--- ---	1137
At5g16570_GLN1.4__cds	--- ---	1071
BnaGLN1.4_C1	--- ---	1273
JX306697	--- ---	1103
JX306692	--- ---	1178
Bra023573_cds	--- ---	1068
BraGLN1.4_C1	TGC ATC AGT CTT CGA ATG ATT CTG TGT GTG CTG GTG TTG CAG ATG CCG GGT G-- --- --- --- --- --- --- --- --- --- --- --- ---	1491
BnaGLN1.4_C2	--- ---	1259
JX306695	--- ---	1309
JX306696	--- ---	1104
BolGLN1.4_C1	--- ---	1160
BnaGLN1.4_C4	--- ---	1102
JX306700	--- ---	758
JX306701	--- ---	758

Bra008612_cds	--- ---	1071
BraGLN1.4_C2	--- ---	1246
BnaGLN1.4_C3	--- ---	1123
JX306698	--- ---	784
JX306699	--- ---	784
BolGln1.4_C2	--- ---	1147
At1g48470_GLN1.5__cds	--- ---	1062
BnaGLN1.5_C2	--- ---	1392
Bra018729_cds	--- ---	1018
BraGLN1.5_C1	--- ---	894
BnaGLN1.5_C1	--- ---	1380
JX306691	--- ---	950
BolGLN1.5_C1	--- ---	578

	1 900	
At5g37600_GLN1.1__cds	--- --- --- --- --- --- ---	1071
BnaGLN1.1_C1	--- --- --- --- --- --- ---	1374
X82997	--- --- --- --- --- --- ---	1354
Bra028132_cds	--- --- --- --- --- --- ---	1071
BraGLN1.1_C1	--- --- --- --- --- --- ---	1362
BnaGLN1.1_C2	--- --- --- --- --- --- ---	1367
Y12460	--- --- --- --- --- --- ---	1264
BolGLN1.1_C1	--- --- --- --- --- --- ---	1348
At1g66200.1_GLN1.2__cds	--- --- --- --- --- --- ---	1071
BnaGLN1.2_C1	--- --- --- --- --- --- ---	1430
X76736	--- --- --- --- --- --- ---	1400
Bra039756_cds	--- --- --- --- --- --- ---	1071
BraGLN1.2_C1	--- --- --- --- --- --- ---	1416
EU499383	--- --- --- --- --- --- ---	1413
AY773089	--- --- --- --- --- --- ---	1426
BnaGLN1.2_C2	--- --- --- --- --- --- ---	1431
Y12459	--- --- --- --- --- --- ---	1344
BolGLN1.2_C1	--- --- --- --- --- --- ---	1290
EU822334	--- --- --- --- --- --- ---	1157
EU822335	--- --- --- --- --- --- ---	1150
At3g17820_GLN1.3__cds	--- --- --- --- --- --- ---	1065
BnaGLN1.3_C2	--- --- --- --- --- --- ---	1336
JX306693	--- --- --- --- --- --- ---	1218
Bra022247_cds	--- --- --- --- --- --- ---	1065
BraGLN1.3_C1	--- --- --- --- --- --- ---	1299
BnaGLN1.3_C1	GCG CCA GCC TGA ATG GCC A--	1487
JX306690	--- --- --- --- --- --- ---	1279
BolGLN1.3_C1	--- --- --- --- --- --- ---	1156
BnaGLN1.3_C4	--- --- --- --- --- --- ---	1273
Bra021276_cds	--- --- --- --- --- --- ---	1065
BraGLN1.3_C3	--- --- --- --- --- --- ---	972
BnaGLN1.3_C3	--- --- --- --- --- --- ---	1275
BnaGLN1.3_C5	--- --- --- --- --- --- ---	1253
JX306694	--- --- --- --- --- --- ---	1257
Bra001686_cds	--- --- --- --- --- --- ---	1065
BraGLN1.3_C2	--- --- --- --- --- --- ---	1292
BnaGLN1.3_C6	--- --- --- --- --- --- ---	1245
BolGLN1.3_C2	--- --- --- --- --- --- ---	1137
At5g16570_GLN1.4__cds	--- --- --- --- --- --- ---	1071
BnaGLN1.4_C1	--- --- --- --- --- --- ---	1273
JX306697	--- --- --- --- --- --- ---	1103
JX306692	--- --- --- --- --- --- ---	1178
Bra023573_cds	--- --- --- --- --- --- ---	1068
BraGLN1.4_C1	--- --- --- --- --- --- ---	1491
BnaGLN1.4_C2	--- --- --- --- --- --- ---	1259
JX306695	--- --- --- --- --- --- ---	1309
JX306696	--- --- --- --- --- --- ---	1104
BolGLN1.4_C1	--- --- --- --- --- --- ---	1160
BnaGLN1.4_C4	--- --- --- --- --- --- ---	1102
JX306700	--- --- --- --- --- --- ---	758
JX306701	--- --- --- --- --- --- ---	758

Bra008612_cds	---	---	---	---	---	---	---	1071
BraGLN1.4_C2	---	---	---	---	---	---	---	1246
BnaGLN1.4_C3	---	---	---	---	---	---	---	1123
JX306698	---	---	---	---	---	---	---	784
JX306699	---	---	---	---	---	---	---	784
BolGln1.4_C2	---	---	---	---	---	---	---	1147
At1g48470_GLN1.5__cds	---	---	---	---	---	---	---	1062
BnaGLN1.5_C2	---	---	---	---	---	---	---	1392
Bra018729_cds	---	---	---	---	---	---	---	1018
BraGLN1.5_C1	---	---	---	---	---	---	---	894
BnaGLN1.5_C1	---	---	---	---	---	---	---	1380
JX306691	---	---	---	---	---	---	---	950
BolGLN1.5_C1	---	---	---	---	---	---	---	578
