# Supplementary material for: Sixteen cytosolic glutamine synthetase genes identified in the Brassica napus L. genome are differentially regulated depending on nitrogen regimes and leaf senescence
Source: J Exp Bot. 2014 Feb 24;65(14):3927–47. doi: 10.1093/jxb/eru041 (PMC4106436; doi:10.1093/jxb/eru041)
Supplement: Supplementary Data [file supp_eru041_jexbot114215_file004.docx]

Genomic sequences of the *BnaGLN1* genes

> *BnaA.GLN1.1.a*

TTATATAAAAAAACGGAGGGAGTAGTATTTAAAAGAACTGATAAATTTTTGATAAGTAGTATCTTCATAATTCCTACCGCCGCTTTTTAGAAATATTATAGGGAAATGGATTTTACCTTAAATAATGTTTTTTTCCGATATCTCATGGAAAGTGTCACCCTGTGTGTACATGTCTGAAAAAAATAAAAAATACATAAGTATATGTTAACTTTGTAAACATTACTAAATATATAAGATTCCATTGGGCCGCAGGAGAATCTTTTATATTTTATTTCTGATTTTTACGTTTTGTAATTTTGGTATTTTGTGGAGAGCCAGAAAGTCTTCTAACTCTTCACGTCACCAACTTTCTCGATCTCTGCACCCTCTTATTCTATAAGTACTCTTCACATCGACCAATATCAATACACAACCAAAACACGAGAAGATTTTATTCGATTTTATCTGTCATTTGCTTCAACTGTTACTATCGACCAATCTCGGTGTTCGTAGCCATGAGTCTTCTTACAGATCTCGTTAACCTTAACCTCTCAGAGACCACTGACAAAATCATTGCGGAATACATATGGTTCGTCTTCTTTCTTCACAAAATCGTTGTTGTTTATGTATTAGATCGTATGCACCCTCATGTTGACCGTAAATTAAATGGGTTCATGTTGTTTTTCCAACTCTTAACTATTTTTGGGGTTTGTATGGAGCAGATTCTGCTAAACACATTTACAAAAAAAAAAAAGATTCTGCTAAACACACATATTTAATTTTCATTTTATTAGTCCTTCGATTTTTCTTATTAGTCCTTTAGATCCAATTTGGTTATGTTATCATGGTAGTTTTTTTGTTTAGTTTTCTGATTAAATCTACTGTTTCAAAATCTGCTGTAAAACTATGTCTGCTCTGTTTCTGGCTCTGTTCCTAAAGACTATTTAAAAAAATGTTATTGTTGTATTATTTCATATGCCAAATCAGGGTTGGAGGTTCAGGAATGGATATGAGAAGCAAAGCCAGGGTAACTTTTCTTTCTCTAGCTATTTTTGGTAATTTGTAACTTTTATCTATTTCTATTTTTGATACTCTACCAAAAGTTTGATTTATAGTAAATCTTGTTTATCTGTAGACTCTTCCTGGACCAGTGAGTGACCCTTCGGAGCTACCAAAGTGGAACTATGATGGCTCAAGCACAGGCCAAGCTCCTGGTGAAGACAGTGAAGTCATCTTATAGTTAATAATCGTTTCTTTCTGATCTTATATCATACCAATTTTCTGCATAAACATTTCTCTATGATTTTATTCAGATTCTTGTTTATTAATCATTTTTATGTTTTGTATTATGTCTTTGTAGCCCTCAAGCCATCTTCAAAGATCCTTTCCGTAGAGGCAATAACATTCTTGTGAGTTTTGATCTTCTTGTTGTTTGCAGCCATATTTTTTTTGACACGTCATAACCTTTTAATGGTTTTAATAATCAGGTCATGTGCGATGCTTACACTCCAGCGGGCGAACCAATCCCAACAAACAAAAGACACGCTGCGGCTAAGGTCTTTAGCCACCCTGATGTTGTAGCTGAAGTGCCATGGTGAGTCCCTATTTAATATTGTTTTAGCCGGTTCTTGTAAAAAATGCGTAGTTATTGATGAGATCTTACTTTGAAAACCAGGTATGGTATTGAGCAAGAGTATACTTTACTTCAGAAAGATGTGAACTGGCCTCTTGGTTGGCCTATTGGCGGCTTCCCTGGCCCTCAGGTGAAGAAGTGATTGATAAATGGGTTTTGATTCTTTTCGATGTTCGGATTTACTAAGTTCTTAAGGTGAAATTTATCTTTGCAGGGACCATACTATTGTAGTGTTGGAGCAGATAAATCTTTTGGTAGAGACATCGTTGATGCTCACTACAAGGCCTGCTTATACGCTGGCATCAATATTAGTGGCATCAACGGAGAAGTCATGCCTGGTCAGGTAGTTTCCGTGACCTACAAGAAATTTTGATCTTTTACATAAAGATCTGATATTTTTCTACATTAATTTATAAATCATAAAGATCAAATCTTTTTCATAATGTTTTGATCTTCCTTTTTTCTTGTTGATAATTAATTGTGAGGTTCAATAATCAAGATCAAATATTTTGAATAAAGTACTAATTACCAAATCTTTTTTTTGTGTTGAGGGCAGTGGGAGTTCCAAGTTGGTCCAGCTGTTGGTATCTCGGCCGGTGATGAAATTTGGGTCGCACGTTTCATTTTGGAGGTATGTTCTAACGATTAAACATTCGAACTTTAATAACTGTTGTGTAGATGTTAATTAATAACATTCTTTGTTGGTTCTAAAAACAGAGGATCACAGAGATTGCTGGTGTGGTGGTATCTTTTGACCCAAAACCGATTCCCGGTGACTGGAATGGTGCTGGTGCTCACTGCAACTATAGGTACACATCTCAAGAATCTGCTATTGTAGTTGAGATCTTCTGATGCTGATTCATGTTAAACACACAAATGGTGAATTGTTTCAGTACCAAGTCAATGAGGGAAGATGGTGGTTACGAGATTATCAAGAAGGCAATTGATAAACTGGGACTGAGACACAAGGAACACATTGCTGCTTATGGTGAAGGCAATGAGCGTCGTCTCACGGGTCACCACGAGACTGCTGACATCAACACTTTCCTCTGGGTAAAACTAACTAACTAACCAACACTTTGTGGGTTTCAAATAGTAAAAGGTTGCATTACATTTTAACTGAAGTTGTGATTGGTTTGTGTTGTGGATGTATAGGGTGTTGCGAACCGTGGAGCATCAATCCGTGTAGGACGTGACACAGAGAAAGAAGGGAAAGGATACTTTGAGGATAGGAGGCCAGCTTCGAACATGGATCCTTACATTGTGACTTCCATGATTGCAGAGACCACAATCCTCTGGAAACCTTGATCAGATCAAAGAAGCTTGTTGAAGAATGTTCACTCCGTTTGGGTTTCTTGCATGGTTCAACGTTTGTGTGTTTCTCTATCAAGCATTGTCTCAGAGCAAGTCAAGAGATTTGCTTTGTTCTTATGGCTTTTATTGTTTCACATCCATTGAAAACATCTCTTTGTATCAATTTATGAATAAA

>*BnaC.GLN1.1.a*

TAATAATAGATAAAAATTAGTTTTATATATTGGTTGCGCGGGTCTTAAACTAGTAACTATTTATAGTGGTTGATATAGACCCACTTGAGCTGATCATAGATGCATAAGAGAATGCCAAGCATGATTCACATTAAATGAACCAACTACTGATTTGAGCGAAACATCAGTAGCAACATCTCAAGTCTTACTTTTAAGGATATATGTTCGGTAGATGGTTCTTGGACCTCTACTTCGAGCTTTAGTGAATGTGGATGAATGTAGAAAGATAGTACTGGAAAAAATAACTTATAGGTTTAAGAAATCAAAGACGAAGAGAATCCGCATTGCATTTGGAGTTGGAAGCGCTAAGCTGGACAACGGAAAACATGGTGTGTCATTCGACATGTCAGCACTTTGAGATGGACTACAAAGACTTGATAGCAATTTTAGTGGTACTTTAGCCATGGCCGATTTTTTCGACAAAATTAAAGGAGATACAAGAACTTAAGAAAAGATTTCATGATTTCAGATATGCTATATCACTCGGGGACAAATGAGACTGCATATTTTTAGCTAAAAACACTCAATTTTTCATAACTCTTTTTTTTTTTAAAGTTTTGTTGGTTATTATATTTCGCCCTGGTTACCAAGATCACTTCAAATTTGAATATTAATATGACATTTTTATAAGAAAACTAAAATATCGTTGAATACTTGTATTTTAGTTTTACATATCAGAATAGTTGTGATTTTGTTTCGTTGCCGAGAGTTTTCAAAATGTTATTTAGGGAAATATTATTTTACCTTTTAAAATAAATATCTAAACTATTTTACAGAGGATAAAAGAAAGCTTCAGAACCTTGGCGCGGACTCAAAACATAAAGACCGACAATTTTGCACGCGGTGCTCGGAGTCATCCGCTGGTATATAGACACTAAGTCACATGTTTGGTAGAGTCTTAACCGAGACTGTTTATCTTGATGACAAAAAAACTATTTTTTCCATGATTTTTATACCATATTTATATGTATATATAAAACCTTCATATTATAGAGTTATAGATATTTTTTTGTGTGGTTTGGAAACATTATAAAGTTATTAATTTTTTTTTTCTATTTTAACTTTAAAGCAAAATATATCAAGTAATAAATAAAAATCAATTTTTACAAATAGAAAATAAAAGGCAAAACATCAATTTTTTTGTATATGAAAGGAATTTTTGTATATAAAAGAGAATTTTTGTGTATACAAAGGAGAGTGGTTGTTGTTTTTTTTCTGAGAACACACATAAAGGGGAGTAGATGTTATCCAACAAAAATAGATATTTCTTCAGTTAAGGAATCAATGATTCAATTATATTCCAATGTTAACATTTATAACTCTTATTCGGACATATTACACCAAAAATCACGTAATTTTGAAAGATTACACCAGCATCATTTTCCAGTAATATTTCACCAAATTCTTTTTTATACAAATTTTTTAAATAAAATGTTTATTAGTATAAAAATTATCAAAATCTCGTAAAAATATTCATAAAAATATAGTATTTAAAAGAACTGGTAAATTTTTGATAAGTAGTATCTTCATAATTCCTACCGCCGCTTTTTAGAAATATTATATAGAAATGGATTTTACCTTAAATAATGTTTTTTTCCGATATCTCATGGAAAGTGTCACCCTGTGTGTACATGTCTGAAAAAACAAAAATACATAAGTATATACTAAATATATAAGATTAAACATTACTAAATAGATAAGATTCCATTGGGCCGCAGGAGAATCTTTAATATTTTATTTCCGATTTTTACGTTTTGTAATTTTGGTATTTTGTGGAGAGCCAGAAAGTCTTCTAACTCTTCACGTCACCAACTTTCTCGATCTCTGCACCCTCTTATTCTATAAGTACTCTTCACATCCACCAATATCAATACACAACCAAAACACGAGAAGATTTTATTCGATTTTATCTGTCATTTGCTTCAGCTGTTACTATCGACCAATCTCGGTGTTCGTAGCCATGAGTCTTCTTACAGATCTCGTTAACCTTAACCTCTCAGAGACCACTGACAAAATCATTGCGGAATACATATGGTTCGTCTTCTTTCTTCCCAAAATCTTTGTTGTTTATGTATTAGATCGTATGCACTCTCACGTTGACCGTAAATTAAATGGGTTCATGTTGTTTTTCCAACTCTTAACTCTATTTTTGGGGTTTGTATGGAGTAGATTCTGCTAAACACACATATTTAATTTTCATTTTATTAGTCCTTCGATTTTTCTTATTAGTCCTTTAGATCCAATTTGTTTATGTTATCATGGTAGTTTTTTTGTTTAGTTTTCTGATTAAATCTACTGTTTCAAAATCTGCTGTAAAACTATGTCTGCTCTGTTTCTGGCTCTGTTCCTAAAGAGTGTTTTTAAAAAAAAAAATGTTATTGTTGTGTTATTTCATATGCCAAATCAGGGTTGGAGGTTCAGGAATGGATATGAGAAGCAAAGCCAGGGTAACTTTTCTTTCTCTAGCTATTTTTGGTAATTTGTAACTTTTATCTATTTCTATTTTTGATATTACTAAAAGTTTGATTTATAGTAAATCTTGTTTATCTGTAGACTCTTCCTGGACCAGTGAGTGACCCTTCGGAGCTACCAAAGTGGAACTATGATGGCTCAAGCACAGGCCAAGCTCCTGGTGAAGACAGTGAAGTCATCTTATAGTTAATAATCGTTTCTTTCTGATCTTATATCATACCAATTTTCTGCATAAACATTTCTCTATGATTTTATTCAGATTCTTGTTTATTAATCATTTTTATGTTTTGTATTATGTCTTTCTAGCCCTCAAGCCATATTCAAAGATCCTTTCCGTAGAGGCAACAACATTCTTGTGAGTTTTGATCCTTCTTCTTGTTGCAGCCATATTTTTCAACATGTCATAACCTTTTAATGGTTTTAATAATCAGGTCATGTGCGATGCTTACACTCCAGCGGGCGAACCAATCCCAACAAACAAAAGACACGCTGCGGCTAAGGTCTTTAGCCACCCTGATGTTGTAGCTGAAGTGCCATGGTGAGTCCCTATTTAATATCGTTTTAGCCGGTTCTTGTAAAAATGCGTAGTTATTAATGAGATCTTACTTTGAAAACCAGGTATGGTATTGAGCAAGAGTACACTTTACTTCAGAAAGATGTGAAGTGGCCTGTTGGTTGGCCTATTGGTGGCTTCCCCGGTCCTCAGGTGAAGAAGACGATTGATACATGGATTTTGAATCTTTTCGATTTCGATGTTCGGATTTACTAAATTCTTAAGGTGAAATTTATCTTTGCAGGGACCATACTATTGTAGTGTTGGAGCAGATAAATCTTTTGGTAGAGACATTGTTGATGCTCATTACAAGGCCTGCTTATACGCTGGCATCACCATTAGTGGCATCAATGGAGAAGTCATGCCGGGTCAGGTAGTTTCCGTAACCTACAAGAAATTTAGATCCTTTTTTATAAATATCTGATATTTTCTATTTGACATTAATTTATGATCATAGAGATCAAATCTTTTTTTTGATCTTTTTTTCTTTTTCTTGTTGATAATTAATTGTGATATTCAAGAATCAAAAGAACAAATCTTTTGCATAAAGTACTAATTACCAAGTCTTTTTGTGTTGAGGGCAGTGGGAGTTCCAAGTTGGTCCAGCTGTTGGTATCTCGGCCGGTGATGAAATTTGGGTCGCACGTTACATTTTGGAGGCAAGCCTTAACGATTAAACATTATAACTCCAATAACGTAACTGTTGTATAGATTTTGAATAATAACATTCTTTGCTTGTTCTAAAAACAGAGGATCACAGAGGTTGCTGGTGTGGTGGTATCTTTTGACCCAAAACCTATTCCCGGTGACTGGAATGGTGCTGGTGCTCACTGCAACTATAGGTACTCATAAATCTCAATAATCTGCATTTGTAGTTGATCCTTTGATGCTGATTCATGTTAAAACACAAATGGTGATTGTTTTCAGTACCAAGTCAATGAGGGAAGATGGTGGTTACGAGATTATCAAGAAGGCAATCGATAAACTGGGAATGAGACACAAGGAACACATTGCTGCTTATGGTGAAGGCAATGAGCGTCGTCTCACGGGTCATCACGAGACTGCTGACATCAACACTTTCCTCTGGGTAAACTAACTAACTAACCAACACTTTGTGGGTTTCAAATAGTTAAAGGTTGCATTACATTTTAACTGAAGTTGTGATTGGTTTGTGTTGGATGTATAGGGTGTTGCGAATCGTGGAGCATCAATCCGTGTAGGACGCGACACAGAGAAAGAAGGGAAAGGATACTTTGAGGATAGGAGGCCAGCTTCGAACATGGATCCTTACATTGTGACTTCCATGATTGCAGAGACCACAATCCTCTGGAAACCTTGATCAGATCAAAGAAGATTGTTGAAGAATGTTCACTCCATTTGGGTTTCTTGCATGGTTCAACGTTTGTATGTTTCTCTATCAAGCATTGTCTCAGAACAAAGTCAAGAGATTTGCTCTGTTCTTATGGCTTTTATTGTTTCACATCCATTGAAAACATCTCTTTGTATCAATTTATGAATAAA

> *BnaA.GLN1.2.a*

TATAAATAGAAATAAAACAGTATTTTTTGGTTACTTTCATTTTTGGTGTTTTGGAGTACATTAGTTTTTTGTCCAATTTATTTTGCGTATTTTTCTTTGTAAATATATGATCTACCTTTTAATTTTTTGAACGATGATATTACGTCTATTATATGCTAATCATATTTATCGTGTTTCATCCATTTGATATTTTTATTTTCAGCGTCTTCTGCTCTTATATTCTTACGTCTGTAATAAGCTAATTAGTAAATTATACGTCTATTATATTTTGATTTTCATCCATTTGATATTTTATATTTATCATTCGGACTTTTTAAGTCTGTTTTTTTCCACAAACGATACTTACAAAACATTTTTGGTCCAAAATCGGTGAAAAAACGGTTTTTTTATTTTTCCTCGCCTATTTGGCCAACTTCCTTGTAAGACAAAAGTATGAACGGTTACTTTATCATGCTAAAGTTATTATTACCCTAGAATACCTACTCAGCTACTCTTACAAGAAAATCAACGCATACATAACTTCTCATAAATTATCCACCTACTTGAATATCAAATGTACAATATACAGTAATCAGAGAATAAGTCAATAATGAATTATTTTGATTCTTGTAAAAAGTCAACAATCAAATTTAAGGACGACACGAAAACAAAAATCACCATTAAACTATATAACTTCAATTTAATACATTGGTTAGATAAAAAGAAATCGCGTTAATATATTTTATGGTTTTAATTTAAAAATAATATTTGGAAAACAGAGGCTCTGCTCTCTCTCCCTATCTCTGTAGTATGTACCCTCGTTGCTCTCTATAAGTACTCCCACAACCACGAACTCCAAAACATCATCTCATAAACCAAAAACCACATTATCCGAGATTTGAGTATATTTCATTGCAACCTTCTTGTCATTTTCTCTGTAACCATGAGTCTTCTGACCGATCTCGTTAACCTTGACCTCTCAGACAACACTGAGAAAATCATCGCTGAATACATATGGTTCGTCTTCCTCCTTCGTTCTTGCCTCTTTCTTACACATTCTTGTTTATGTTCTTGTTCTTTCCTTATCCAGATAGATAGAAGATATTGTTTTGTAATTACATATTTACATCTTCTACCACACAAAAAAAGGGTTATGTGTTTTGTCTTTTCTTATTTTGTTTTTTTTTTTTAGAAAAAAAAACAGGGTTGGTGGTTCAGGAATGGATATGAGAAGCAAAGCCAGGGTATATGTTTTTCACTTAACTCGACGAAAAATAAAAAAAAATTGTCGGAAAATTGACCCACACGGAGAAAGATTCTGAACTTTTTTGGTATTAGAAAGATTAGGAGTTTGATTATACTGAGTTACTGATTCTCTGTTTTATTTACCTGCAGACTCTCCCTGGACCTGTGACCGATCCATCAAAGCTCCCAAAATGGAATTATGATGGTTCAAGCACTGGCCAAGCTCCTGGTGAAGACAGTGAAGTGATCTTATAGTAAGGCCTCTTCTCAAATTTTTATCACTATTTCAAACTCATCTTTTAATTTTATTTTTTGCTTAATAACCTTCCTTTTTATTTGTGTTCTTTAGCCCTCAAGCGATTTTCAAAGATCCGTTCCGTAGAGGCAACAACATTCTTGTGAGTTGTTAAACTTCGGTTTTCTTGCGCATGTTCTGTTTTGGCAGTTAATGATAACGTTTTTTTTAATCGGTTTTGTTAGGTCATGTGTGATACTTACACCCCTGCGGGTGAACCAATCCCTACGAACAAGAGACATGCTGCAGCTCAGATCTTTAGCAACCCTGATGTTGTTGCTGAAGTGCCATGGTTAACCCAAATTCCCCTGTTCCCGTTTTCGTTATATATTTTTTTGGTTTCTTGCGGTCTGATTTAGCTCTGCATACTAGGTATGGAATCGAACAAGAATACACTCTGTTGCAGAAAGATGTGAATTGGCCTGTCGGATGGCCCATTGGTGGATTCCCCGGCCCTCAGGGACCATACTACTGCAGTGTTGGAGCTGACAAATCTTTTGGAAGAGACATTGTTGATGCTCACTACAAGGCTTGTTTGTATGCTGGAATTAACATCAGTGGAATCAATGGAGAAGTCATGCCTGGTCAGGTATAACTTCAAGATCCAACAGAATTAAATCTTTTTTGCATAATGTAGTAATGACCAAATAATTTTTTTTTTTGTTGAGACAGTGGGAGTTCCAAGTCGGACCGTCGGTTGGTATCTCAGCTGCTGATGAAGTGTGGATTGCTCGTTTTATTTTGGAGGTATCATCCACTCTCAATTGTTGTTGCTCTCTTAATGGAATATAAAATTGAGTACAATTTTTTGGTGGTTTTAAAAAACAGAGGATCACAGAGATTGCTGGTGTGGTTGTATCTTTTGACCCAAAACCAATTCCGGGTGACTGGAACGGAGCTGGTGCTCACACCAATTACAGGTAAAAGGATCATGCAACTTAAGCTTGTTATATAATTTGTTCTTTTGGAAAAAAAAAATCTGAAATATGGTAAATAATTTTTTCAGTACTAAATCGATGAGGGAGGAAGGAGGATACGAGATAATCAAGAAGGCAATTGATAAGCTCGGACTGAGACACAAGGAGCACATTTCTGCTTACGGTGAAGGCAACGAGCGTCGTCTCACTGGACACCATGAAACTGCTGATATCAACACTTTCAAATGGGTAAACATTTAAATTTTTTAAAGAGTTGATAACATTTTTGTGTACTGAACTTTGTGTTTTTTTTTTTTTTGAATCTTTGTTAAAAGGGTGTTGCAAACCGTGGAGCATCAATCCGTGTAGGACGTGACACGGAGAAGGAAGGGAAAGGATACTTTGAGGATAGGAGGCCAGCTTCCAACATGGACCCTTACACTGTAACTTCCATGATTGCAGAGACTACACTTCTTTGGAATCCTTGAAGAAATATGACAATAATATAACTTGAATCTGGTTTCAAGTTTGTGTTTCTACAGTTTATTAAGCAATTACCGGGTTGATACTGCCGGAGTTTGTGGTTTGAGGCCTTTCTTTTAATCTCTTTGTGTTTTGGGGTTTGTGATTGGAGCAAAAGCCCTGATTTGCTCTGTTTCTTTGACCTTTTATTTGAACCCTTTGTATTTGTATTAATAAGACGATCTGAAAAGGCCTTTTCATGTTTCTATCTGAGACTGCTCAACAAGTTCACAAATAACAAAACTCTTTGGTAGGCCGAGAACCTGTAAGCTAGACGGGTCCATCTATAAATATATTGGTACACACATAAATGTATTCTAGGCACCGTATAAAATACAATCATAGATCATATTCAATCCGGAGAAACATGTCTTTCTCGAACAATAGATGGAACCTAACTAATTAGAAATTGGGCATTTGAAATGTTTGTAATGCGAGTATCAAGTCCTCAATCAAACTTGAATTGGCATGATGTTGCTGGTTGCACCTCCAAGAAAATGACAACTCGATCACCTTATCCTTTGGATCATGCGATGAGAGTCCCAAGATTGTGCAGTCTTTAAGCAACAAGAATTTCTCGAGATCAATCTTGTTCTGTGATCCATCGAACGTTGTCCACTTGAGACTTTTTATTTGTTTATGTCAGTTTGCCTCCATGATTAGGAACCATACACAACCGGTATTTATGAAAAGTTATTTTCATAACTCCTGATTTTTGGTTTGAACAAATGTGTTTCTTCGTTGAAAATATATGTAAAATTTTCATATTATTTCTTGGTTGTTATATATATGAGACAATGCAAAGAAGAGATAATCAAAACAAAAACAACAGTTAAGGACTGTTGTTAGAATTCTATAGAAAATTCAAGAGATAGTATTGAGATGAAGACTTGAAGAGAAGTTAACATGATCTCTGCATGTTTAACATTTATTCTCCTAGGAAATGAAGATCTCTTGTATGTTCATCATTTAAATTATTCATGCAAGAGCATGCAAGTGTTGTCGCATTTATTGATAAATCGTGTATTGTCTATATAAAGGTTGTGACTTCTCTCTTGTAATATATAGAAATACACTCAATATAAATAAAA

>*BnaC.GLN1.2.a*

TATATAAACCGAACCGAAGTAAATATAGATTTAGAATGATACTTATATTTTACTAACTGAAATACCGAAAACCCAAAAAAAACGAACCCAAACCGAAACGATATCCGGATTGAACACCGCTATTAAATTAATGTATTTTAATTTCATTTATTTATTTTCTATTTTATTTAATCATTTGAATTTTATTTAGTTTTCTGATAGCTTTACAAAGAATATATGGCTCATTTCGTATAACTAGATTTTCCACTAATCATTTTCAATAATATAATATTAATTTAACAAAAAAAAAATTATAATGTAAGATGATGAAAATGAAGAATATTAATTTATATGAAAAATGATGACGTCAAAGCTTTTATTGTTGGTGTTTTCAAAGGGGAAGTCTCCTCCAATGCATTTTCCAGTCAGCAAATTATGCGCTTGTATTGTATTTTTTATAGAGAAATTGCGCTCTCTACACAGCACTTTTATCATAATTAGCCTCATACCCTAAATCCCACAATCTTCTACCTTATTCCATAGATACAAATTAGTCAAATCACAGGTATTCTCTTTTTTTTGGGTTTTCAACCGATTGCTTCTTTTTTATAAGTAAAAATAAAACAGTATCAAATTCATTTTTTTTTTGTGTGTTTTGGAGTACATTAGTTTTGCGTATATTTCTTTGTAAATATATGATCTACCTTATTATTTTTTGAACTATGGGATTACCTCTATTATATGCTAATCATATTTATCCTGTTTCATTCATTTGATATTTTTATTTTCACCGTCTTCTGCTCTTATATTTTTACGTCTATAATAAGTTTTGCGTATATTTCTTTGTAAATATATGATCTACCTTATTATTTTTTGAACTATGAGATTACCTCTATTATATGCTAATCATATTTATCCTGTTTCATTCATTTGATATTTTTATTTTCACCGTCTTCTGCTCTTATATTTTTACTTCTATAATAAGTTAATTATACGTCTATTATACTTTGATTTTCATCCATTTGATATTTTATATTTATCATTCGGACTTTTGTAAGTCTAATTTTTTCCACAAACGCTAATTATTTACAAAACATTTTTGGTCAAAAATCGATGAAAAAAAAATGGTTTCTATTTTTCCTCGCCTATTAGGCCAACCTCGTAGAAAAAGTATGAACGGTTACTTTATAATGCTAAAGTTATTATTACCCTAGAATACCTACTCAGCTACTCTTACAAGAAAATCAACGCATACATAACTTCTCATAAATTATCCACCTACTTGAATATCAAATGTACAATATACAGTAATTTGGGAATAAGTCAATAATTATTTGTTTTTAATTTTTGTAAAAAGTCAATAATCAAATTTAAGGACGACACGAAAACAATAATCACCATTAAACTATATAACTTCAATTTAATACATTGGTTAGATAAAAAGAAATCGCGTTATTATATTTTATGGTTTTAATTTAAAAATAATATTTGGAAAACAGAGGCTCTGCTCTCTCTCTCCCTATCTCTGTAGGTACCCTCGTTGCTCTCTATAAGTACTCCCACAACCACGAACTCCAAAAAATCATCTCATAAACCAAAAACCACATTATCCGAGATTTGAGTATATTTCACTACAACCTTCTTGTCATTTTCTCTGTAACCATGAGTCTTCTGACCGATCTCGTTAACCTTGACCTCTCAGACAACACTGAGAAAATCATCGCTGAATACATATGGTTCGTCTTTCTCCTTCGTTCTTGCCTCTTTCTTACACATTCTTGTTTATGTTCTTGTTCTTTCCTTATCCAGATAGATAGAAGATATTATTTTGTAATTACATATTTACATCTTCTACCACACAAAAAAAGAGTTATGTGTTTTGTCTTTTCTTATTTTGGTTTTTTCTTTTGAAAAAAAAAACAGGGTTGGTGGTTCAGGAATGGATATGAGAAGCAAAGCCAGGGTATATGTTTTTCACTTTAACTCGACGAAAAATAAAAAAAAATTGTCGGAAAATTGACCCAGACGGAGAAAGCTTAGGAGTTTGACTATACTGAGTTACTGATTCTCTGTTTTGTTTACCTGCAGACTCTCCCTGGACCTGTGACCGATCCATCAAAGCTCCCAAAATGGAATTATGATGGTTCAAGCACTGGCCAAGCTCCTGGTGAAGACAGTGAAGTGATCTTATAGTAAGGCCTTTTCTCAACTTTTATCACTATTTCAAACTCATCTTTTATTTTTTTTTTGCTTAATAACCTTCCTTTTTATTTGTGTTCCTTAGCCCTCAAGCGATTTTCAAAGATCCGTTCCGTAGAGGCAACAACATTCTTGTGAGTTGTTAAACTTTGGTTTTCTTGCGCATGTTCTGTTTTGGCAGTTAATGATAACGTTTTTTATCGGTTTGGTTAGGTCATGTGTGATACTTACACCCCTGCGGGTGAACCAATCCCTACGAACAAGAGACATGCTGCAGCTCAGATCTTTAGCAACCCTGATGTTGTTGCTGAAGTGCCATGGTTAACCCAAATTCCCCTGTTCCTGTTTTCGTTATATATTTTTTTGGTTTCTTGCGGTCTGATTTAGCTCTGCATACTAGGTATGGAATCGAACAAGAGTACACTCTGTTGCAGAAAGATGTGAAGTGGCCTGTTGGATGGCCCATTGGTGGATTCCCCGGCCCTCAGGTATATTCCGTTTTCAGAGTTCTTGTTCAATTCTCTGTTCTCAGCTTCTAATGTTTATACAAAAACTATCTTTCTCAGGGACCATACTACTGCAGTGTTGGAGCTGACAAATCTTTTGGAAGAGACATTGTTGATGCTCACTACAAGGCTTGTTTGTATGCCGGAATTAACATCAGTGGAATCAATGGAGAAGTCATGCCTGGTCAGGTATAACTTCAAGATCCAACAGAATTAAATCTTTTTGCATAATGTAGTAATGACCAAATCATTTTTTTTTTTGTTGAGACAGTGGGAGTTCCAAGTCGGACCATCGGTTGGTATCTCAGCTGCTGATGAAGTGTGGATTGCTCGTTACATTTTGGAGGTATCATTCAACCATCATCCACTCTTAATTCTTGTTGTTATCCTAATGGAATATAAACTTGAGTACAATTTTTTGGTGGTTTTAAAAAACAGAGGATCACAGAGATTGCTGGTGTGGTTGTATCTTTTGACCCAAAACCAATTCCGGGTGACTGGAACGGAGCTGGTGCTCACACCAATTACAGGTAAAAGGATCATGCAACTTAAGCTTGTTATATAATTTGTTCTTTTGGAAAGAAAAAATCTGAAATATGGTAAATGACTTTTTCAGTACTAAATCGATGAGGGAGGAAGGAGGATACGAGATAATCAAGAAGGCAATTGATAAGCTCGGACTGAGACACAAGGAGCACATTTCTGCTTACGGTGAAGGCAACGAGCGTCGTCTCACCGGACACCATGAAACTGCTGACATCAACACTTTCAAATGGGTAAACATTTAAATTTTTTAAAGAGTTGATAACATTTTTGTGTACTGAACTTTGTGTTAATTTTTTTTGAATCTTTGTTAAAAGGGTGTTGCAAACCGTGGAGCATCAATCCGTGTAGGACGTGACACGGAGAAGGAAGGGAAAGGATACTTTGAGGATAGGAGGCCAGCTTCCAACATGGACCCTTACACTGTAACTTCCATGATTGCAGAGACTACACTTCTTTGGAATCCTTGAAGAAATATGACAATAATATAACTTGAATCTGGTTCTCTGGGGCTTCAAGTTTGTGTTTTCTACAGTTTATTAAGCAATTACCGGGTTGATACTGCCGGAGTTTGTGGTTTGAGGCTTTCTTTTAATCTCTTTGTGTTTTGGGGTTTGTGATTGAAGCAAAAACCCTGATTTGCTCTGTTTCTTTGACCTTTTATTTGAACCCTTTGTATTTCTATTAATAAGACGATCTGAGAAGGCCTTTTCATGTTTCAACTTAGACTGTTCAACAAGTTCACAAATAACAAAACTCTTTGGTAGGCCCAGAACCTGTAAGCTAGACTCTAAAACTATTGGTAGTTGGTACACACATAAATGTATTGTAGGAACCGTTATAAAA

> *BnaA.GLN1.3.a*

ATAAAAATGACAACTTTTTTTTTTGTTACAGTACAACTAAAGGAGAAAAGTTTCTAGTTGTATACAAAAGATGTATCGATCTTATAGTTTCTCTGTACGATTCAATTATTGAGCTGCATGATTGAGTCCATGCAAGTTGGTTAAAAGGTCATGTCTATTTAGACACATCAGGTTAATGCGAAGAGCAAAACAGGGCGCAACAAAATGGGCTTTATGACAAATTGGAGGAAAACGTTACGTACGCAAAATACAAAAGTGTCCACGTCTTGATTCATCATATGATAATACTTTTATAGTCATTAGTGCAAAACTCATTTCATTTCGTATCTCACTAAACTACGTTTACGTGTTCATCAACAATTCTAATTTTACTGATTTACTCATAAACATATCCTCCAGTTATCCATAATCAGGTTTGTACTAAAAGACAAAAGATATGAAGAATATCAAGAAACTTCTTAGTTTTTGCGAATTGCACCCGTTTCATCATCCTACGATTCATAAAACTTAGCTACAACAAACTAAAAGACCTTCGTCTATCAATAGTGCAACCACTTTTTCTTCATGTAGTAACCACAGAAGAGAATTAGTTCCGTTTGATCATAAATAAACTCCTTTCGCCTGTTCATAATAATAAAGAAACACTTAAAATAATAATTGTAATTATCTCTGTGTAAATGTCAGGAGAATCCTAACGGAATCGTTTATCTAATAAAATTAACTATTAAAAAAATCAAAAGATAATGTTGGCAGACAACAGTAAATAAAATCATGTAATTATTTGAAAATAAAAAATAAATTAAAAAAAACAAAAAAATAAGGCTATAAAATAAAAACACTCGTCAGGAGAGAAGCTGCATTGATCGTCTTCCCTTAGACAAACACTGATTGATTATCTTTCTCTTTTCTCTCGTGACGGCGCCATGTCTCTCCTCTCAGATCTCGTCAACCTCAACCTCTCTGACTCCACCGAGAAAATCATCGCCGAATACATATGGTTCGATCTCCCCTTCTCTCTCTTTCACGACGAGTGTCATTATTTTGGTTTCTTTCTATTAATACGATCGTAAAGTTTTCACCTTTCTTCATCTTGCTATTATTGATCTGTTATGGTTTTGAGAGTTTCTCTATGAACACGGTTCACTGAGTTTGACGTAGTATACATCATAAAGCATGTCTCTTTTTTGATATTTTCTTTCTTGTCGTCTTCTGGGTTTGTGAGTTTTCGTATGAACTTTGAATGAACAGGATCGGTGGATCTGGAATGGACATCAGAAGCAAAGCCAGGGTAATTAAATAAATACTCTGTTTCTTGATCCTCTGTTTTTATCCTCTGTTTTTTTATTTGGCTGATCTGATTTGTTTTGGATGATACAGACACTACCAGGACCAGTGAGCGATCCATCAAAGCTGCCTAAGTGGAACTACGACGGATCCAGCACCGGTCAAGCCGCCGGAGATGACAGTGAAGTCATTCTATAGTATGTTCTTTTATTATCTCTATTTACATATAAAACATCAAGTAAATATGAAGTAAAAAGTGTTGATCTTTGTTTTGTTTTGTTAATGAATGATCGATAGCCCTCAGGCGATATTCCGTGATCCGTTCAGGAAAGGCAACAACATTCTGGTAGGTTTAATTTTATATGATTAGAGCGTGACAATATTCATTTGTTACAAAATCAAATTTTAAGAAAATCATTGTTTATTGTTATCTATATATGCAGGTGATGTGTGATGCTTACACGCCGGCAGGGAATCCAATTCCGACCAACAAGAGGCACAACGCTGCTAAGATCTTCAGCAACCCCAAAGTTGCCTCTGAGGAGCCTTGGTAAGCTCCTCTTCTTCTTCCTCTATTGATCTCTCTGTTAATCAGTTAATCATATACTAATACTCTTCTCTTCCCTTTGACTTAAAAAGGTATGGGATTGAGCAAGAATACACATTGATGCAGAAGGGTGTGAACTGGCCTATTGGTTGGCCTATCGGTGGCTTCCCTGGCCCTCAGGGACCTTACTACTGTGGTGTGGGAGCTGACAAGGCAATTGGTCGTGACATTGTGGATGCACACTACAAGGCCTGTATTTACGCAGGTATTGGCATCTCTGGTGTCAATGGAGAAGTCATGCCTGGTCAGTGGGAGTTCCAAGTCGGTCCTGTTGAGGGTATTAGTGCTGGTGACCAAGTTTGGGTCGCTAGATACCTTCTCGAGGTAAGACTAATCTGTACTTCACCGGTTATGTTCCCCATAAAATGCTTCAAACTTTGCTAACGGTTATCATCTTTCCGACAGAGGATCACTGAGATCTCTGGTGTGAATGTCAGCTTCGACCCTAAACCAGTCCCGGTTAGTGTCTCTAATCTCTAACCACACTCTTCTTGTGCAATAAGTTGTAACATTGTTGCGTTGGTTCAGGGTGATTGGAACGGAGCTGGAGCTCACTGCAACTACAGCACGAAGTCAATGAGGAACGACGGAGGATTAGCTGTGATTAAGAAAGCGATAGAGAAGCTTCAGGTGAAGCACAAGGAGCACATTGCTGCTTACGGTGAAGGCAACGAGCGTCGTCTCACGGGGAAGCACGAAACTGCAGACATCAACACGTTCTCTTGGGGAGTGGCGAACCGTGGGGCTTCGGTGAGAGTGGGGAGAGACACTGAGAAAGAAGGCAAAGGTTACTTCGAGGACAGAAGGCCAGCTTCTAACATGGATCCTTACGTTGTTACGTCCATGATCGCTGAAACCACCATCCTCGGTTAAACCACACACATTTAGTAATATTTGATTTTCTCCGGTTTGGTTTTATGATTTGAATACTGTTGTACTTGTGATCGCGGTCTATTTCGGTTTCAATAATTCTTATGACGACATTTTGTGTTTTTTCTTCTTCTTAGTTTCGAATAATTAAATAAGGGTATTCATGCGGTGAAAAAA

>*BnaC.GLN1.3.a*

ATTGATCGTCTTCCCCTAAACAACACTGATTGATTATCTTTCTCTCGTGACGGCGCCATGTCTCTCCTCTCAGATCTCGTCAACCTCAACCTCTCTGACTCCACCGAGAAAATCATAGCCGAATACATATGGTTCGATCTCCCCTTTCTCTCTTTAACGACGAGTGTCATTATTTTGGTTTCTTTCTATTAATACGATCGTAAAGTTTTCACCTTTCTTGTTTCTCTATGAACACGGTTCACTGAGATTGACGTGATACATGTCTTTTTTTTTTGTTCTTTTGTTTTTTTGTCGTCTTCGGGGTTTGTGAGCTTTTCGTATGAACTTTGAATGAACAGGATCGGTGGATCTGGAATGGACATCAGAAGCAAAGCCAGAGTAATTAAATAAATACTCTGTTTCTTGATCCTCTGTTTTTTTTTGGCTGATCTGATTTGTTTTGGATGATACAGACACTCCCAGGACCAGTGAGCGATCCATCAAAGCTTCCTAAGTGGAACTACGACGGATCCAGCACCGGTCAAGCCGCCGGAGATGACAGTGAAGTCATTCTATAGTATGTTCTTTAAAAATCTATATTTATATATAAACATCAAGTAAATATGAAGTAAAAAGTGTTGATCTTTGTTTTGTTTTGATAATGAATCGATAGCCCTCAGGCGATATTCCGTGATCCGTTCAGGAAAGGCAACAACATTCTGGTAGGCTTAATTTTATATGATTAGAGCCTGTCAATATTCATTTGTTACAAAATCAAATTTTAAGAAAATCATTGTTTATTGTTATCTATCTATGCAGGTGATGTGTGATGCTTACACGCCGGCAGGGAATCCAATTCCGACCAACAAGAGGCACAACGCTGCTAAGATCTTCAGCAACCCCAAAGTTGCCTCTGAGGAGCCTTGGTACTCTCCCTTTTCTTCTTGGTCTGCTAATCAGTTAATCATATACTAATACTCTTCTCTTCCTGTGACTCAAAACTCAAAAAGGTATGGGATTGAACAAGAATACACATTGATGCAAAAGGGTGTGAACTGGCCCATTGGTTGGCCTATCGGTGGCTTCCCTGGCCCACAGGGACCATACTACTGTGGTGTGGGAGCTGACAAAGCCATTGGTCGTGACATCGTGGATGCGCACTACAAGGCCTGTATTTACGCAGGTATTGGCATCTCTGGTGTCAATGGAGAAGTCATGCCTGGCCAGTGGGAGTTCCAAGTCGGTCCTGTTGAGGGTATTAGTGCTGGTGACCAAGTCTGGGTCGCTAGATACCTTCTTGAGGTAGACTAATCTTTCTTAAACCGGGTTTTGTTCCCAACACAAGTTTCAAACATTGCTAATAATCATCATCTCTTTTGACAGAGGATCACCGAGATCTCTGGTGTCAATGTCAGCTTCGACCCTAAGCCAGTCCCGGTTAGTGTCTCTAATCTCTAACCACACTCTTCTTGTGCAATAAGTTGTAACATTGTTGCGTTGGTTCAGGGTGATTGGAACGGAGCTGGAGCTCACTGCAACTACAGCACGAAGTCGATGAGGAACGACGGAGGATTAGCTGTGATTAAGAAAGCCATAGAGAAGCTTCAGGTGAAGCACAAGGAGCACATTGCTGCTTACGGTGAAGGCAACGAGCGTCGCCTCACGGGGAAGCACGAGACCGCGGACATCAACACGTTCTCTTGGGGAGTGGCGAACCGTGGAGCTTCGGTGAGAGTGGGACGTGACACTGAGAAAGAAGGCAAAGGTTACTTCGAGGACAGAAGGCCAGCTTCTAACATGGATCCTTACGTTGTTACTTCCATGATTGCTGAAACCACCATCCTCGGTTAAAATCGCACACGTTCTTATGTTTGGGTTTATCTTATCTGGTCTGGTTTTCAATTTGCAAACTTGTGCTTGTGATTGCCGGTTTATCTCGGTTTAAATAATTCTTATGACGACATTTTGTATTTTTTTTCTTCCTAGTTTCGAATAATTAAATAAGGTATTCATATGGTGAAAAAAGTCTATCAGCGACTTTTGTGTTGTTGTTTTGAAATGATAATAATAATAACAAAGTACGGCCTGGCGCGTTGGAGGTTAATGAGCTTGTCGGGAGTAGTAAAGTAGGTGGATAAGATCTGGTGATTAATAATTATGAATTTATCTTAAAATTTAAATGTTGAATGTTCTTTACAACATTATGCAGGATGTGGCATGTTACAGAACATCTCCAATGTATACGTAGAAATATCATATTTATATATTTTTTTCTATAAATAAA

> *BnaA.GLN1.3.b*

TGAATGGAACTTGAGATTGTTGATTGTTGGATTAGATAAGTTTAAAGATAAATTCAATAGAAAAAGAATAGAGGTTACATGATTGTGTCGAACTGTTGGGTAATGCTAGGTTTTATTTCATTGTCTAAGTGGTGATTAAAGTTTCATATTAGGATATTTCATTGTCTTAGTTATTTGTTTGATTTAATTTTATTTGTCTCTTTCTTTTTAGATTTATAAGTATTAAATGTAACTTCTACCAAATCTACAAAATCACTCCACAATCATGATTATATATTTTGCAGATCATTGAATAAGAATAGAATTAAAAAAATACAATCCTTACTATTTTTTCTCATGAACAAAACTATAGATTTTTAGTGATTCTTGTGAATGGACTATTTTTGTCTTTTGTATTAGATGTTAATAAAAAAAAAACAAAAAGTATGATATATGTTCTAATTCGTAATTGAATGTATATTTAAAACTAATAACATACTAAAAACCATTTTTCGCATATTCTCTGAGTTTCTTGATTTTCAAGTAATTATGTAGGCTTGGAGATCACTGCATGAATAGTGAGTAGCAAAATTTTGAACAATGACCATATGTAAATAGTCCACTCCCTTTCTTGTTTTAGGTTTTACGCCTCTTTCATGGTGATATCTTTTTTTGCTTTTCCATCATGGGTTTGCATTTCTTTAAGAGCTTCAAATGTAAAAAAAAGAAGCTAATTGTGCTTCTTTCTTTTATCAATTTTAGATCAGTACAATGTCTAGGCTAGTTGAAATAGATGTTAATCTATTGAAGAAATTAAATGGGAAAAGGGTATGACATTGTTCACGTTAGATTCTTGTTTCTTTCATTTCTCAGTTACTTAACACAATTTCTTATGAATGTTTATAAACAAGTGACATTAAAGCCATTAGTTTTGCTAGTAGGTTAACTTCTAGTCTAGTTTAACATGTAGATGGACACGGACGATCGATAAAATGAGCAGCACCGAGACATGATGTTCCGACATGATGAATGAAACAGTATTTGATTTCACCTGTTTGAATTGCATCACTTTGAGTTTGTTTTTAAGTATTGGTGTGCAAAAATGACAAGATTTTGTCACACTACTAAAAAAAAGTTTAGCTGTATACATCATGTACATGTATGTTGCATTTTCATGACTATTGGCGTTGCTAGAAAAAAGAACTTGCTTTCAAAATTAACAAATACGTGATGATTGAATCCAACTTAGCATGATAAAAAGAATCATTATGTACGCAAAGACAAAAATATCCAAGACTCGATTAATACAAATCTCGTTTCGTTTATTTACCTAAGTAGTTTTCGTGTTCATCAACAATTCTATTTATACCAATTTAATCATAGATTACATATATTTTAATATTGTTGTCATAATCTGTATCTAAATCACTGGAGAATCTTACCTGAAACAATATAACAATATTAAATAATTGTTTAAAATTCTAAAAGATAATTATTGACACACAACAATAAATAATCACCAATTTACTAGAATCATGTATTGACCACCGTAAATATCTGAAAATTTAAAAAAAAAAACAAAAACAAAAATAGCTCTATAAACACTCGAATAAGTTTTAATGATCGTCTTCCCTAAACAACACTGATTAATTTTAGTATTTTTTTTCTCTCGTATTCTCCTCTCAGACGCAGCCATGTCTCTGCTCTCAGATCTCGTCAACCTCAACCTCTCCGACACAACCAAGCAAATCATCGCCGAATACATATGGTTTGATCTACCTTCATCTCTGTTTATCTCTCACGACAATTCTCATTATCTCTGTTCTTACTATTGCTACGTTCAGAGAGCTTTGTCTTTTATATTTTTTTTTTGGTACCGATCTTTTATGTGGTTGGTTTGAACACGATTCGTTTCACTCGGTTAACGCTGTCATGCCATGCGGCGATGAGATTGACGTGTACAAACATAAAGCTTGTCTCTTTTTTCTTTGTCGTCTTCTGGGTTTGTGATGTTTTTCGTGTGTATTGTGAATGAACAGGATCGGTGGATCTGGAATGGACATTAGAAGCAAAGCCAGAGTAAGTCGATACTCTGTTTTCTTGCTCTGTTTTTACTTTTTACTCTGTTTCTTGTATGTTTGATCTGTTTTTGTTTTGGTTTATATAGACACTACCAGGACCAGTGACCGATCCATCAAAGCTTCCCAAGTGGAACTACGACGGATCTAGCACCGGTCAGGCCGCTGGAGATGACAGTGAAGTCATTCTATAGTATGTTCTTCCATCATCAGCAAGTAATATGAAATAAAAACAGATCTTATCTTAATCTTGATCTTGTTTTGTTTCGTTTATAGTCCTCAGGCTATATTCCGTGATCCGTTCAGGAGAGGCAACAACATTCTGGTGATGTGTGATGCTTACACGCCGGCCGGTAATCCAATTCCGACCAACAAGAGGCACAACGCTGCAAAGATCTTCAGCAACTCAAAAGTTGCCTCTGAGGAGCCTTGGTACTAACTCTCCTCTTCTTCTTATTGGTCTATGTCACTCTCTGCTTAATCATTTACTTATATACTAAAACTCTTGAAAAGGTATGGGATTGAGCAAGAATACACATTGATGCAAAAGGGTGTGAATTGGCCCATTGGTTGGCCTGTTGGTGGCTTCCCTGGCCCACAGGGACCGTACTACTGTGGCGTGGGAGCTGACAAAGCCATTGGTCGTGACATCGTGGATGCACACTACAAAGCCTGTCTTTACGCAGGTATTAGCATCTCTGGTGTCAATGGAGAAGTCATGCCTGGCCAGTGGGAGTTCCAAGTCGGTCCTGTTGAAGGGATTAGTGCCGGTGATCAAGTCTGGATAGCTAGATTCCTTCTCGAGGTAATAATCTACTTAACCAGTTCCGTTCACATTACATGCTTCAAAATTTTATTTTATTTTTAAGCATCAAAATTTTGCTCATGGGTCATCTTTTCTTTTGACAGAGGATCACTGAGATCTCTGGTGTAAACGTCAGCTTCGACCCAAAACCAGTCCCGGTTAGTGTCTCTCTAGTGCTCTTTATGTGCAATAACTAGAGTGTTGTTGTAACTTTTTTTGTTATTTTTGCTAAATTATATTCTATTAAAATAGTTTTTTTTTTTTTTGCTAAAATTTGGTTCTATTAAAATTGTTGTAACTTGTAACGTATTGTTGTATGGGTTTCAGGGTGATTGGAACGGAGCGGGAGCGCACTGCAACTACAGTACGAAGACGATGAGGAACACTGGAGGATTAGCGGTGATAAAGAAAGCGATAGAGAAGCTTCAGGTGAAGCACAAGCAGCACATTGCTGCTTACGGTGAAGGCAACGAGCGTCGTCTCACGGGCAAGCACGAGACGGCAGATATCAACACGTTCTCATGGGGAGTGGCGAACCGTGGAGCTTCGGTGAGAGTGGGACGTGACACTGAGAAAGAAGGCAAAGGTTACTTCGAGGATCGTAGGCCAGCTTCTAACATGGATCCTTATGTCGTCACTTCCATGATCGCCGAAACCACAATCCTCGGTTAAGTAATCAATACGCATTTTAATGTTTAGTTTTTTCCTTAATTTGCGGATTTTTGTGCTTGTGGTTGCACTACTAATAATTATTATCATGCCCTAGTAGGATTCACGTGTTTTATGTTTTTTTTTTTCATTTCGAATAATTAAATAAGGAATTCTTATGGATGAAATTTAAACAAAAGAATGTATTATTAGTGATTTTTTTTGTTGATGTGGTTGAACTTGTTAGGGATAAGATTTTGTGATTATTGATAACATAAGAAATTAATAAGAAATAAAAA

> *BnaC.GLN1.3.b*

TCTAACAATGATTAATCTTTACTACCTTAAACCAATGAAAACAAATTTTAAACTATATAGTTTATTTTAAAAATTAAACAAAAACTAAATGTTTAATTATTTACTCGATAATATAAATCTATGAAGGGAAAAGTTTAATTTCTTAAAAACTTTCTAAATTTGTGAAATGTTACAATATCTTTGAATATGAGAATAAAACAATATTTTACTAATCTTTATATATATAGTTACGATTTTAATAATGAAATAATAATCCGAAAATATATATATATAAGAAAATACAAATACATGTGAAAGTTTGAAACAATCTATTCAAAGGAAAAAAATATACCGTAAACTTATTTTGTTTTAAAAATTGATAGACACATATATATTATAATATATACCAATTTAGAATTGAAAACAAAATATTTATATAAAAATAAATGAAAATAAATGAAAACAAAAATCTGCGCGGTTGCGCGGATCGAGATCTAGTTTCTTTTATTTCTCAATTGCTTAACACTATTTTATGAATGTTTATAAACAAGTGACATTGAAGCCACTAGTTTTGCTAGTATGTTAACTTCTAACCTAGTTTAACATGTAGATGGGGACAAGGACAATCGATAAAGTGGGCAGCTCCGAGGCATAATTTTTTGACACGATGAATGAATGAAACAGTATTTGATTTCACCTGTTTGAATTGCATCACTTTGAGTTTGTTTTTAAGTATTGGTGTGTATAAATGACAAGATTTTGTCACACTACTAAAAAAAAGTTTTATCCGTATACATCAGATATATCTATCTTATATACATGTATATTGCATTTTCATGAATATTGGCGTTGGCTTGAAAAATAAAAACTTGGTTTCAAAATCAACAAATACGAGATGATTGAATCCAACTTAACATGATAGAGTCATTATGTACGCAAAAGACAAAAATGCCAAGACTCGATTCATCATCACAGGCTCACAGTGATGATACCTATATAGGCAAGTAATACAAAACTCGTTTCGTTTAGTTACTTGACTATTTACGTGTTCATAAACAATTCTATTTAACTGATTAAATCATTGATTACATATATGTAAATATTGTTGTCATAATCTGTATTTAAATCACAGGAGATCTTAACTGAAACAATATAATAATACAAATTAATTATTTAAAATTCTAAAAGATAAATATTAGTATCACACAACAATAAAGAAATTACCAATTTACTAGAATCATGTATTGACCACCGTAAATATCTGAAAACAATAATAAATAAATAAACAAAAATAGCTCTATAAACACTCAGAGAAGTTTTAATGATCGTCTTCCCTAAACAGCACTGATTGATTAGTGTATTTTTTTCTCTCGTATTCTCCTCTCAGACGCAGCCATGTCTCTGCTCTCAGATCTCGTCAACCTCAACCTCTCCGACACCACCAAGCAAATCATCGCCGAATACATATGGTTTGATCTCCTTTCCTCTCACGACAACTCTCATTATCTCTGTTCTTAATATTGCTACGGTCAGAAAAATTTGCTTTTTATGTTGTTTTGGTTTGAATATGATTCGTTTCACTCGGTTAACGCTGTCATGTCATGCCATGCGGCGATGAAATTAACGTGTACAAACATAAAGCTCGTCTCTTTTTTCTTTGTCGTCTTCTGGGTTTGTGATGTTTTTCGTGTGTATTGTGAATGAACAGGATCGGTGGATCTGGAATGGACATTAGAAGCAAAGCCAGGGTAAGTCGATACTCTGTTTTTTTTACTCTGTTTCTTGTATGGTTGATCTGTTTTTGTTTTGGTTTATACAGACACTCCCAGGACCAGTGACCGATCCATCAAAGCTTCCCAAGTGGAACTACGACGGATCAAGCACCGGTCAGGCCGCTGGAGATGACAGTGAAGTCATTCTATAGTATGTTCTTCCATCATGTCTATATACAGCAAGTAATATGAAATAAAAACATATCTTATCTTAATCTTGATTTTGTTTCGTTTATAGTCCTCAGGCTATATTCCGTGATCCGTTCAGGAGAGGCAACAACATTCTGGTGATGTGTGATGCTTACACGCCGGCCGGTAATCCAATTCCGACCAACAAGAGGCATAACGCTGCTAAGATCTTCAGCAACTCTAAAGTTGCCTCTGAGGAGCCTTGGTATTAACTCTCCTCTTCTTCTTATTGGTCTATTCTCTGCTTAATCATTTAATTATATACTAAAACTCTTGAAAAGGTATGGGATTGAGCAAGAATACACATTGATGCAAAAGGGTGTGAACTGGCCTATTGGTTGGCCTGTTGGTGGCTTCCCTGGCCCACAGGGACCATACTACTGTGGTGTGGGAGCTGACAAAGCCATTGGTCGTGACATCGTGGATGCACACTACAAAGCCTGTCTTTACGCAGGTATTAGCATCTCTGGTGTCAATGGAGAAGTCATGCCTGGCCAGTGGGAGTTCCAAGTCGGTCCTGTTGAAGGTATTAGTGCTGGTGATCAAGTCTGGATCGCTAGATTCCTTCTCGAGGTAGTAATCTACGTCTGCTTAACCAGTTCTGTTCACATTACAAGCTTCAAAATTTTATTTTATTTTTAATTTTATTTAATTTTTAAGCATCAAAATTTTGCTAATGGGTCAGGGTCATCTTTTCTTTTGACAGAGGATCACTGAGATCTCTGGTGTAAACGTCAGCTTCGACCCAAAACCAGTCCCGGTTAGTGTCTCTCTAATGCTCTTTATGTGCAATAAATAGTGTTGTTGTAACTTTTTCTTTTTTTTGCTAAATTATACTCCTTCTTTTTCCGTTATAAGTGTCGTTTTAGTTTTTGACCCACGGATAAGGAAACAATTAATTTTGTATTTTTCCTATAAAAAAAACACTATTACCTATCCACCTAACCATATTTCAACTAATAGGAAAATAAATTTTGCATAAAATTAATAAATTTTGCATTTAAAATCGAAAACGACACTTATTTTGTAAGGAAAAAATTTCTCTAAAACGACACTTAATATGAAACGGAGGGTGTATTCTATTAAAATTGTTGTAACTTGTAACGTTTTTTTTTGTAACGTATTGTTGCATGGGTTTCAGGGTGATTGGAACGGAGCTGGAGCTCACTGTAACTACAGCACGAAGACGATGAGGAACACTGGAGGACTAGCGGTGATAAAGAAAGCGATAGAGAAGCTTCAGGTGAAGCACAAGCAGCACATTGCTGCTTACGGTGAAGGCAACGAGCGTCGTCTCACGGGGAAGCACGAGACGGCAGATATCAACACGTTCTCGTGGGGAGTGGCGAACCGTGGAGCATCGGTGAGAGTGGGACGTGACACTGAGAAAGAAGGCAAAGGTTACTTCGAGGATCGTAGGCCAGCTTCTAACATGGATCCTTATGTCGTTACTTCCATGATCGCTGAAACCACAATCCTCGGTTAAGTAATCGCACGCATTTTATAATGTTTGGTTTATCCGGTTTCGTTTATTTCTTAATTTGCGGATTTTTGTGCTTGTGGTTGCACTACTAATAATTATTATCATGCCCTAGTAGGATTCACGTGTTTTATGTTTTTTTTTCATTTCGAATAATTAAATAAGGAATTCTTATGGATGAAATTTAAACAAAAA

> *BnaA.GLN1.3.c*

TATATATAAACACTCTCAGGAGAGAAGCTGTATCGAGATCGTCTTCCCAAAACAACACTCATTGATTGATTACTATCCGACGCAGCCATGTCTCTGCTCTCAGATCTCGTCAACCTCAACCTCTCCGACTCCACCAAGCAAATCATCGCCGAATACATATGGTTCCAACATCTCCCTTCTCTCTGTTTCTCTCTCACCACGAGTCTCATTATCTCTGTTTTCTTATCTTCGTTATCTCTGTTTGTTACTATTGCTGCGATCGAAACACTTTGATCTCTTATGTTTTGACAATTTTGGTTTGAACAAGATTCAGTTCACTCTGTTTTTAGCTGTCCTGACTCGATGAGATTGACGTGTACATCGTAAAGCTTGTCTCTTTCTTTGTTGTCGTCTTGTGGGTTTGTAATTTTCTCGTATGAATGTTGAATGAACAGGATCGGTGGATCTGGCATGGACATTAGAAGCAAAGCCAGAGTAAATTTGATACTCTGTTTTTTTTCATTCCTCTGTTTCTTGTATGATCGTTCTGATTTGCTAATGGATAATACAGACACTCCCAGGACCAGTGAGCGATCCATCAAAGCTTCCCAAGTGGAACTACGACGGATCCAGCACCGGTCAGGCCTCTGGAGATAACAGTGAAGTCATTCTATAGTATGTTCCTCTTTATCTACTGTATAAGCATTAACACAAAATCTTGTCTTAATCTTCTTTTTTGTTTTGTCTCGTTTTATGAATCGATAGCCCTCAGGCCATATTCCGTGATCCGTTCAGGAGAGGCGACAACATCCTGGTAGGGCTGATTTTTAAATGTTTTTTTTTTGTGCACAAAATTTAAAAATGATTAGTGTGACTAAATTCAATTTGGATCAGAATAAAAAATTGGTTAATGTGATATATTCAGGTGATGTGTGATGCATACACACCGGCCGGAGATCCAATTCCGACAAACAAGAGGCACAAGGCTGCTAAGATCTTTAGCCATCCTAACGTTGCCAAGGAGGTGCCTTGGTAATCTCTTCTTCTTTGTATATTGATCTCTCTGCAAATCAATCTTATATACTTATTCTCTCTCTCTCTCTCTGACTCAAAAAGGTATGGGATTGAGCAAGAATACACTTTGATGCAAAAGGGTGTGAACTGGCCTATTGGTTGGCCAATTGGTGGCTTCCCTGGTCCTCAGGTAAATCTAAATGATCAACTGATCTTGATGTCATCACAACACTTTTTTTTTTTACTTAACTTTCTAATATTATGACGCAGGGACCATACTACTGTGGTGTGGGAGCTGACAAAGCCATTGGTCGTGACATTGTGGACGCACACTACAAGGCCTGTCTTTACGCAGGTATTGGCATCTCTGGTGTCAATGGAGAGGTCATGCCTGGACAGTGGGAGTTCCAAGTCGGTCCAGTTGAGGGTATTAGTTCTGGTGATCAAGTCTGGGTCGCTAGATACCTTCTTGAGGTAGAGTACTCTACTTTAACCGGTTCTATTGTGAATATCTCTACAAGATTTGCTAATGGCCATCTTATCTCTTTTGACAGAGGATCACTGAGATCTCTGGTGTAAATGTCAGCTTCGACCCAAAACCAGTCCCGGTTAGTGTCTCTTTACCACACTCTCTCAACACTCTCTTCATGTAAAATAAGTTGTAACAATGTTTGCATGGGTTGTTCAGGGTGATTGTGCAACTACAGCACGAAGTCGATGAGGAACAACGGAGGATTAGCAGTGATAAAGAACGCGATAGAGAAGCTTCAGGTGAAGCACAAGGAGAACATTGCTGCGTACGGTGAAGGCAGCGAGCGTCGTCTCACGGGGAAGCACGAGACCGCATACATCAACACGTTCTCTTGGGGAGTGGCGAACCGTGGAGCTTCGGTGAGAGTGGGACGAGACACTGAGAAGGAAGGCAAAGGTTACTTCGAAGACAGAAGGCCAGCTTCTAACATGGATCCTTATGTCGTTACGTCCATGATCGCTGAAACCACCATCCTCGGTTAATCAATCATGCACGTTTTAATGTTTTGGTTTTCTCCGGTCTGGTTTTCAATTTGTGAACTTATGTGCTTGTGATTGCAGTCTATTTAGGTTTAAATAATTCTTATGACGACATTTGGTGTCTTTTTCTTTAGTTTCGAATTATTAGATAAAAGGATTCTTATGGTGAAAAAGATAAGTGTTGTTTGAAAATTGAAATGATAATATTAGTATCAACGTATGGCTCGGCACATTGGGAGGTTGATAGTGTATAACCAGAGAATTATCAAAGAACCTATAAACAGATATTTAGTTATTGTTACTATATATAAATATAATTTCTAAAAACAGTTGTTCTATTGAGCTTTTTGGCAACATAATCTAATGTATACACCACTATCTAGCTTACACCTTCAAGTAAGAAATTTTGTTAGAATTAGCATGAATGGAACACTCTAAAAACATGATCTTCCTGGATTCACAACCCAATTTTTATTTTTATTTTTAAACAAACAACAATTTCATCTAAAACCGTTTTTTCATGTCGACTTTCTTATAACTATATGGGGAGAAGAAGTGAAAGTTTCAGATAATTACAAAAGTATAAAAAACGAGTTATTTTGGTTACGAGCACTAATTCAAAAGTATACAAATTTAAATACAAAAAATTAAATTATAATGTATTATATTTTAAACTATTGAAAAATAAAAA

> *BnaC.GLN1.3.c*

ATAAAAACACTCTCAGGAGAAGAGCTGTATCGAGATCGTCTTCCCTAAACAACACTCATTGATTGATTACTATCCGACGCAGCCATGTCTCTGCTCTCAGATCTCGTCAACCTCAACCTCTCCGACTCCACCAAGCAAATCATCGCCGAATACATATGGTTCCATCATCTCCCTTCTCTCTGTTTCTCTCTCACGACTAGTCTCATTATCTCTGTTTTCTTTTCCTCGTTATCTCTGTTTGTTACTATTGCTGTGATCGAAAAGCTTTGATCTCTTATGTTTTTGACAATTTCGGTTTGAACACGATTCACTTCACTCTGTTTTCACTTGTTCTGTCTCGATGAGATTGACGTGTACATCGTAAAGCTTGTCTCTTTCTTTGTTGTCGTCTTCTGGGTTTGTAATTTTCTCGTATGAATGTTGAATGAACAGGATCGGTGGATCTGGCATGGACATTAGAAGCAAAGCCAGAGTAAATTTGATACTCTGTTTTTTTCATCCTCTGTTTATTGTATGATCGTTCTGATTTGTTTGTGGATGATACAGACACTTCCAGGACCAGTGAGCGATCCATCAAAGCTTCCCAAGTGGAACTACGACGGATCCAGCACCGGTCAGGCCTCTGGAGACAACAGTGAAGTCATTCTATAGTATGTTCTTCGATCCTCTTTATCTACTGTATAAGCATTTACACAAATCTTGTCTTAATCTTCTTTCTTGTTTTGTCTCGTTTTATGAATCGATAGCCCTCAGGCGATATTCCGTGATCCGTTCAGGAGAGGCGACAACATCCTGGTAGGGCTGATTTTTTATATGATTAGTGTGAGACTAAATTCAATTTGGATCAGAATTTTTTTAAAAAATGATTGGTCAATGTGATATATATTCAGGTGATGTGTGATGCATACACACCGGCCGGAGATCCAATTCCGACCAACAAGAGGCACAAGGCTGCTAAGATCTTCAGCCATCCTAACGTTGCCAAAGAAGTGCCTTGGTAATCTCTTCTTCTTTGTATATTGATCTCTCTGCAAATCAATCTTATATAATTATACTCTCTCTCTCTGTATCAAAAGGTATGGGATTGAGCAAGAATACACTTTGATGCAAAAGGGTATGAACTGGCCTATTGGTTGGCCTGTTGGTGGCTTCCCTGGTCCTCAGGTAAATCTAAATAATCAACTGATCCTAATGTCATCACAACTCTTTTTTTTTACCTAACTTTCTAATATTATGACGCAGGGACCATACTACTGTGGTGTGGGAGCTGACAAAGCCATTGGTCGTGACATTGTGGACGCACACTACAAGGCCTGTCTTTACGCAGGTATTGGCATCTCTGGTGTCAATGGAGAGGTCATGCCTGGACAGTGGGAGTTCCAAGTCGGTCCAGTTGAGGGTATTAGTTCTGGTGATCAAGTCTGGGTCGCTAGATACCTTCTTGAGGTAGAGTACTCTACTTTAACCGGTTCTATTGTGAATATCTCTACAAGATTTGCTAATGGCCATCTTATCTCTTTTGACAGAGGATCACTGAGATCTCTGGTGTAAATGTCAGCTTCGACCCAAAACCAGTCCCGGTTAGTGTCTCTTTACCACACTCTCTCAACACTCTCTTCATGTAAAATAAGTTGTAACAATGTTTGCATGGGTTGTTCAGGGTGATTGTGCAACTACAGCACGAAGTCGATGAGGAACAACGGAGGATTAGCAGTGATAAAGAACGCGATAGAGAAGCTTCAGGTGAAGCACAAGGAGAACATTGCTGCGTACGGTGAAGGCAGCGAGCGTCGTCTCACGGGGAAGCACGAGACCGCATACATCAACACGTTCTCTTGGGGAGTGGCGAACCGTGGAGCTTCGGTGAGAGTGGGACGAGACACTGAGAAGGAAGGCAAAGGTTACTTCGAAGACAGAAGGCCAGCTTCTAACATGGATCCTTATGTCGTTACGTCCATGATCTGAACCACCATCCTCGGTTAATCAGTCATGCACGTTTTAATGTTTTGGTTTTCTCCGGTCTGGTTTTCAATTTGTGAACTTATGTGCTTGTGATTGCAGTCTATTTAGGTTTAAATAATTCTTATGACGACATTTGGTGTCTTTTTCTTTAGTTTCGAATTATTAGATAAAAGGATTCTTATGGTGAAAAAGATAAGTGTTGTTTGAAAATTGAAATGATAATATTAGTATCAACGTATGGCTCGGCACATTGGGAGGTTGATAGTGTATAACCAGAGAATTATCAAAGAACCTATAAACAGATATTTAGTTATTGTTACTATATATAAATATAATTTCTAAAAACAGTTGTTCTATTGAGCTTTTTGGCAACATAATCTAATGTATACACCACTATCTAGCTTACATCTTCAAGTAAGAAATTTTGTTAGAATTAGCATGAATGGAACACTCTAAAAACATGATCTTCCTGGATTCACAACCCAATTTTTATTTTTACTTTTAAACAAACAACAATTTCATCTAAAACCGTTTTTTCATGTCGACTTTCTTATAACTATATGGGGAGAAGAAGTGAAAGTTTCAGATAATTACAAAAGTATAAAAAACGAGTTATTTTGGTTACGAGCACTAATTCAAAAGTATACAAATTTAAATACAAAAAATTAAATTATAATGTATTATATTTTAAACTATTGAAAAATAAA

>*BnaC.GLN1.4.b*

TATATATACACTTGCAGGAATCTCTTTAGTGTTATACAAAACAAAGCTAATATTTTTTTTTAGATTCCTAGAGAAAATGTCGGCACTTGCAGATTTGATCAATCTCGATCTCTCCGATTACACTGAGAAGATCATTGCGGAGTACATATGGTCCGTACAATTCATTCACCTCTATCAAATAATATTCAGTCTATATTAGTAGATGGATGATTTTGGTTGATCTTTGCCTTCTGTGTTTGAGTTGTGTTTGTTTCTAAACGTCTCTTCCTTGAGTTTTTTTTGAATATGTATGAGTTAAAACTTTTAAACAATATACTAGAACGTTGAAAGACTTTGGACTGATTGAACCTTCTGGTTAATATATTGTGGTTTTAATGAATAGGATTGGTGGATCAGGCTTGGATATGAGAAGCAAAGCAAGGGTAACTATATTATTTTTGTTTGTTTTTTAGAGCATAAAACGATGAATACATCTTTGAAATAATGTTAAGAACTTAAGCAAATGAATGATTATATTCATTATTATTGCAGACTTTGCCCGGACCAGTGAAGGATCCATCGGAGTTACCGAAATGGAACTATGACGGTTCAAGCACCGGCCAAGCTCCCGGCAGTGACAGTGAAGTCATCCTCTAGTATGTTTCAATATAAATTAATACATATAATAAGCTAGATAAAATTGTAAATCGTGGATTAATAAGATTGTGGTTTCTCTATGTATTTTTTAGCCCTCAAGCTATCTTCAAAGACCCCTTCAGAAGAGGCAACAACATCCTTGTAATAACTCAATTACACAATGAAAACAATAATATTCAAATTTGTTATATAAATTAAACATTTTTTTGTTTTTGGGATTGATTTGAAAATTAGGTAATGTGTGACGCATATACACCGGCCGGTGAACCGATTCCGACGAACAAAAGGCATGCTGCAGCTAAGATCTTTAGCGACCCCAGCGTTGCCGCCGAAGAAACATGGTATATATCTTCTAAATGAAATACTTAGTCGAAGTTTTATTTCAATTGTTTGCCAAAAAAAGAAATTCATTTCAATTTTTGTTTGAAAACTACTAGTTTAAGTTTGCATACTCCCTTATATTTGTTGACATCATCATGCTAGTTTTAAATGTAAATGGTATAACATGCCCTAGTCAACTTTGATAGTTGTTACATTTTCTTATAACTTAAAAATATAATAAAAGTAAATTGATCATAAACACACTGGTTAGTCTAAATTTTCAGGATGACATTAGTGACAATAAATTTTCATTTTTGGTTGTAATTATTTTCAGTAACCATTCATTATTGAAATTTAGGTATGGAATTGAGCAAGAGTATACTTTGCTTCAAAAGGATATTAAGTGGCCGGTAGGTTGGCCTGTTGGCGGCTTCCCAGGTCCTCAGGTAATGACTTTCAGCAAATCTATTCACATTAACGTACCCTTTCGTTATTGAGTTTCAACCCAAAAATAATAATGAAAAATCAGAAAACAGGGACCGTACTACTGTGGAGTTGGAGCAGACAAAGCCTTTGGAAGAGACATAGTAGATTCTCATTACAAAGCCTGTCTTTACGCCGGAATCAATGTCAGTGGCACTAACGGAGAAGTCATGCCCGGACAGGTTTAACACTTACCAGTCACTCTTTTTTATTCTTCCGATTCATTATCATATTGATTTGAGTTCAGAACAAGAAATCAAACCGTTAAAACAAATCTTGTGTGGGTTTCAGTGGGAGTTCCAAGTCGGTCCAACGGTTGGAATCGCTGCCGCCGATCAAGTCTGGGTCGCTCGTTACATCCTCGAGGTATATATGATAAACACACTCAAGGTTATGTATACACCCTGTAACCTTATCTCTTCATATACTCTGTTTTAATGACTCTTTTCTTGGTTATGTGAAAACAGAGGATCACAGAATTGGCTGGAGTTGTTTTATCTCTTGACCCTAAACCAATTCCGGTTTGTTTTTACCATAACTTTAAATATATAACCAAATTTTGTTCTTTTCAAATTCACTAAATTACTCTGTTTGATTTTTCTATTTTAGGGAGATTGGAATGGTGCAGGAGCGCACACAAATTACAGGTTCTTTGTGTTTTCAAGATCTGCTCTGATTCATTTTATATGGTTTTTGTTTGTTTTTATGAATTTGGTTTCTTGTATTCTGCAGTACAAAGTCGATGAGAGAAGATGGAGGGTACGAGGTGATAAAGAAAGCGATAGAGAAGCTTGGATTGCGTCACAAGGAACACATCTCTGCTTATGGTGAAGGCAACGAGCGTCGCCTCACTGGCAAACACGAGACTGCCGATATCAACACTTTCTTATGGGTAATAGTTTTATTTCATAGCATGTCAAAGTGTGCATTCATATCACATGAGCCTAAGTCATGCATGATCTAACTTAACTGGTTTGTACAGGGTGTGGCCAACCGTGGGGCATCGATTAGAGTTGGTCGGGACACTGAGCAAGCTGGGAAAGGGTACTTTGAAGATCGTAGGCCAGCGTCCAACATGGATCCGTACACTGTGACCTCCATGATTGCTGAAACTACAATCCTCTGGAAGCCATGAATGAAGAAAACTCGAGCTTCAAGGAACCTCTAATATCAGTTCATGTGTCATTCTTATATTGTCTCGTTCTGTCTTCTTTGTTTAAGTGTGTTTGATTTAAACTCTTAGAACTTGTTCTATTTCTATATTATGTTATTACAAATTCAAATATCAGTTTTAGAATATGAAGAGGATCTGAACTTTAAACTTTTTATAGAAACCAAAATCGCAAGTTGCAAGCTCCTCCGTTACTAGCCAACCCAAACTAGACCGGCCGAATCGACATTTAGCACACTCTATTTGGCCCATTTTAGCTTAATGGACTCAAATTTATAACTTTTAAATATTGAAACTCTGTGCAAAAAAAA

>*BnaA.GLN1.4.b*

TATATATACACTTGCAGGAATCTCTTTAGTGTTATACAAAACAAAGCTAATTATTTTTTTTAGATTCCTAGAGAAAATGTTGGCACTTGCAGATTTGATCAATCTCGATCTCTCCGATTCCACTGAGAAGATCATTGCGGAGTACATATGGTCCGTACAATTCTTCATTCACCTCTGTCAAATAATATTCAGTCTATATTAGTATATGGATGATTTTGGTTGATCTTTGCTTCTGTGTTTGAGTTGTGTTTGTTTCTAAACGTCTCTTCCTTGAGGTTTTCTTGAATATGTATGAGTTAAACTTTTAAACAATATACTAGAACGTTGAAAGACTTTGGAATGATTGAACCTTCTGGTGAATATATTGTGGTTTTAATGAATAGGATTGGTGGATCAGGCTTGGATATGAGAAGCAAAGCAAGGGTAACTATATTATTTTTTGTTTGTTTTTTAGAGCATAAAACGATGAATACATCTTTGAAATAATGTTAAGAACATAAGAAAGAATGATTATATTCATTATTATTGCAGACTTTGCCCGGACCAGTGAAGGATCCATCGGAGTTACCGAAATGGAACTATGACGGTTCAAGCACCGGCCAAGCTCCCGGCAGCGACAGTGAAGTCATCCTCTAGTATGTTTCAATATAAACTAATACATTTAATAATCTAATTAAATTTGTAAATCGTGGATTAATAAGATTGTGGTGTCTCTATGTTTTAGCCCTCAAGCTATCTTCAAAGACCCCTTCAGAAGAGGCAACAACATCCTTGTAATAATTCAATTACACAATGAAAACAATAATCTTCAAATTTGTTATATTATATATATATATATATATTTTTGTTTTGTTTTTGCGATTGATTTGGAAATTAGGTGATGTGTGATGCATATACACCGGCCGGCGAACCGATCCCGACAAACAAAAGGCATGCGGCGGCCAAGATCTTTAGCGACCCGAGCGTTGCCGCCGAAGAAACATGGTATTTATCTTTTAAATGAAATATTTAGTCAAAGTTTTATTTCAATTGTTTGCCAAAAAAAGAAGAAGTTCATTTCAATTTTTGTTTGAAAACTACTAGTTTAAGTTTGCATACTCCCTTATATTTGTTGACATCATCATGCTAGTTTTAAATGTAAATGGTAATAGCATGTCCTAGTCAACTTTGCATAGTTGCTACATTTTCTTATAACTTAAAAATATAATAAAAGTAAATTGATCATAAACAAACTGGTTAGTCTAAATTTTCAGGATGACATTAGTGACAATATATTTTCATTTTTGGTTGTAATTATTTTCAGTAACCAATCCTTTTTTTATTATTGAAATTTAGGTATGGAATTGAGCAAGAGTATACTTTGCTACAAAAGGATATTAAATGGCCGGTAGGTTGGCCTGTTGGCGGCTTCCCAGGTCCTCAGGTAATGACTTTTTCGGCAAATCTATTCACATTAACCTACCCTTTCGTTATTGAGTTTTAACCCAAAAATAATAATGAAAAATCAAAAAACAGGGACCGTACTACTGTGGTGCTGGAGCAGACAAAGCCTTTGGAAGAGACATAGTGGATTCTCATTACAAAGCCTGTCTTTACGCCGGAATCAATGTCAGTGGCACTAACGGAGAAGTCATGCCCGGACAGGTTTAAAACTTACCAGTCACTCTTTTTTATTCTTCCGATTCATTATCATATTGATTGAGTTCAGAACAAGAAATCAAACTGTTTAAACAAATCTTGTGTGGGCTTCAGTGGGAGTTCCAAGTCGGTCCAACCGTTGGAATCGCTGCCGCCGATCAGGTCTGGGTCGCTCGTTACATCCTCGAGGTATATATAATAAACACACTCATGGTTATGTACACCCTGTAACCTTATCTCTTCATATAATCTGTTTTTATGACTCTTTTCTTGGTTATGTGAAAACAGAGGATCACAGAATTGGCTGGAGTTGTTCTGTCTCTTGACCCAAAACCAATTCCGGTTTATTTTTTACCATAAATTTAAATATTATAACCAAAAAGTTTTTTTTTTCAAATTCATTCAATTTTTCTATTTTAGGGAGATTGGAATGGTGCAGGAGCACACACAAATTACAGGTTCTTTGTGTTTTCAAGATCTGCTCTGATTCATTTTATATGGTTTGTGTTTGTTTTTCTGAATTGGGTTTCTTGTATTCTGCAGTACAAAGTCCATGAGAGAAGATGGAGGGTACGAGATCATAAAGAAAGCGATAGAGAAGCTTGGATTGCGTCACAAGGAACACATCTCTGCTTATGGTGAAGGCAACGAGCGTCGTCTCACTGGCAAACACGAGACTGCCGATATCAACACTTTCTTATGGGTAAGAGTTTTCTTTCATAGCATGACAAAGTGTGCATTCATATCACATGAGCCTAAGTCATGCATGATCTAACTTAACTGGTTTGTACAGGGTGTGGCCAACCGTGGGGCATCGATTAGGGTTGGTCGGGACACTGAGCAAGCTGGGAAAGGGTACTTCGAAGATCGTAGGCCAGCGTCCAACATGGATCCCTACACTGTGACCTCCATGATTGCTGAAACTACAATCCTCTGGAAGCCATGAATGAAGAAAACTTGAGCTCCTCCAAGGAACCTCTAATATCAGTTCATGTTCATTCTTCTATTGTCTCGTTCTGTCTTCTTTGTTTAAGTATGTTTGATTTAAACTCTTAGAACTTGTTCTATTTCTATATTATGTTATTATACAAATTCAAATATCAGTTTTAGAATATGAAGAGGATCTGAACTTTAAACTTCTTATAGAAACCAAAATCGCAAGTTGCAAGCTCCTCCGTTACTAGCCAACCCAAACTAGACCGGCCGAATCGACATTTAGCACACTCTATTTGGCCCATTTTAGCTTAATGGACTCAAATTTATAACTTTTAAATATTGAAACTCTGTGCTAAAAATATAATAATTTTGAAACTCTATTATACAATGAACTAACGTACTTAAACAAGAAGTAAGAACACGCAGAGAGTACACTACCAATCAATTTATCTTATGCTACTTTGATTCTCGGAAACATTTTCTTGAAACTTCGATATTTCCTTTCTTTTCGTATTTGGTTTAAATTATTATGACTACTATTCATCTTAAGAAACTAACAAAATGAGTTCAGTTTGGATTCATTTTTCAGAATGTATGGGAAGAGAGTTTATTTTCTGTTCGTCTGACATTTTGTTGAAGTCAGGTATGAAATTAAAAACAAAAAAACTATTAGCTTTAAAGTATTATTTTATTCAAGGTTTGGTTTCTCATAATCGAGTTTAAATCCTAATTTTCTGTCCAGGAAAAAACTTCTAATTTGGTCATCAAAAATTTCAAGATATTTTTAATCAATTTTTCATCTACTTATGATTTAAGATTGACAAAGTAATTTACAGCAGACTAAAATATTAACAGTTCAAAGCTATTACGAAATACGCCGATAGGACTAAAACATTTTTTTTCTAAAACATAGGACTAAAACTTTAATGCCAAAATCTAATGGTTGAGAGCTCTTCTTGCAATTCCGTGTTAAAACTTTCATTCATGTAACTAACTGGTAGTATACATCAGTTACCCATACAAGTTAAAGTAAAAAATCTTTATGTTAAGAACTTAAGATTGACATGCATGCATGTTTACATGTGTATATAAACACACACTTAGTTTGGAATTAAAGAAAAACACACATGTATATAAAGAATTGTTTGATCGTCTTTCTCCGAATAAGAGGCGAAACATAATCAACTTGAATTCGGGACAGCTATTCGAAATCTTAGTGAAAATTTTGATTTGTTATCTCATGTTTGCTTTTCGTGAAAAAATACCAATTGAAGTGGAGGGTTTCTTCAAACAACAAGGACCATAAACACACACACCTAGTTTGGAATTAAAGAAAAAAACACACACGCATATAGTTTAGGAGAAGGTTTGTTAACTGTGAAGTGAATGCGCCGAAGTTTTTTAAAAGTAAAAAGATAAGTAAATGCTTCAAGGATTGAAATAAA

> *BnaC.GLN1.4.a*

TATATAAATACACTTGCAGGAATCTCTTTAGTGTCATACAAAAAAAGTTTAGTATTCTCTTCAGAGTCCTAGAAAAATGTCGGCTCTTGCAGATTTAATCAATCTCGATCTCTCCGACTCCTCTGAGAAGATCATTGCCGAGTACATATGGTCCGTAAAATTCTTCATCGATCGATGTCAAATAATATTTCTTTTTGATTTAATCTACAACAGTATATTATTTGTTTATGTGTTTGAGTTGGCGTTTGTGTCTATAGTTCTCATCCATGAGGTTTTCTTATGACTCAAAACTTTGAAATTTTGCTTGATATACTAAAAGTTGAAAGATATTGGATTGATTGAATATTTTGATTAATATTTTCGTGATTGATGAATAGGATTGGTGGATCAGGCTTGGATATGAGAAGCAAAGCAAGGGTAATTTATTTATTTTTTTGTGTATAGAGTAAATCGTTAAAGTAATATCAAGAATTTAGGACATGATTAATAATATTATTGCTGCAGACTTTGCCGGGACCTGTGAAGGATCCATCGGAGTTACCGAAATGGAACTATGACGGTTCAAGCACCGGGCAAGCTCCCGGCGATGATAGTGAAGTCATCATCTAGTATGTTCCGTAATTAAATCGATTAATAAATTAAAAGTAAAAAATTAATTCTTGAATTAATGAGATTTTGGGTTTTCTATGTGTTTTTTCAGCCCTCAAGCTATCTTCAAAGATCCATTCAGAAGAGGCAACAACATCCTTGTAATAACTCAATTAAACAACTAGAACAATACTCTTGGAATTTGTTGCAATATTCATTTATTTTGTTTCTGTGATTGATTTGGAATTAGGTGATGTGTGACGCTTATACACCGGCTGGCGAACCGATCCCAACCAACAAAAGGCATGCGGCGGCTAAGATCTTTAGCGACCCAACCGTTGCCGCCGAAGAAACATGGTATACATCTTCACAATGAAATTTTTTTTGTCAAAGTTGACCTCAATTTTTTTGTTTGAAAACTTTATGTTTGCATAGTTAATTTTTTGTCAACACCATCGTGCGAGTTTAATTCCAAATGGTACCGCAAGGAGACGTAACATGACGTAGTACTTTATTGATATATATTTTTATAACGTAAAAGTATAATAAAATAATTTGACCATAAACACATTTGTTACTCTAAATCTACATTTTTCTAAATTAATTAAATATTTCAAAAATATATTGAAATTAAGGTATGGAATTGAGCAAGAGTATACTTTGCTTCAAAAGGATACCAAGTGGCCAGTTGGTTGGCCCGTCGGTGGCTTCCCAGGTCCTCAGGTAAAGATTTTTACGGCGAATCTTTTCACTTCTTGATCTCAATCCAAAACTCCAGTTAGTGGCAAATAAATATATAAATATTTATTAATAAAAAAACAGGGACCATACTACTGTGGAGTTGGAGCAGACAAAGCCTTTGGAAGAGACATCGTTGATGCTCATTACAAAGCATGTCTTTACGCTGGAATCAATGTCAGTGGCACTAACGGAGAAGTCATGCCCGGACAGGTTTAATATAATTCCCTTTTATTCTTCTGATTTAATATCATATTGATCGCGATCAGACCGTGAAACTGATATTGTGTGGGGTTTAGTGGGAATTCCAAGTCGGTCCAACCGTTGGAATAGCTGCAGCCGATCAGGTCTGGGTCGCTCGTTACATCCTCGAGGTATATATAACACACACTCATCAAGACCTGTCTCTTCATATACTCTGATTTATGACTATGTTTCTTGGGTATGTTAAAAACAGAGGATCACAGAACTGGCTGGAGTTGTTTTATCTCTTGACCCTAAACCAATTCCGGTTAGCTTTACCATATTTTCAATTATTTACCAAATAAACCTTTGATTCTTTTAAAATTCACTGAATTACTCTATTTTGATTTTTATATTCTAGGGAGATTGGAATGGTGCAGGAGCACACACAAATTACAGGTCTGCTACGATTCCGTTTATATGTTTTTTCTCTGTGTTTTTTTTTTGTTTTCTGAATTTGGTTTTCTTGTTTCATATAGTACGAAGTCGATGAGAGAAGATGGAGGGTACGAGATCATAAAGAAAGCGATAGAGAAGCTTGGACTTCGTCACAAGGAACACATTGCTGCTTATGGTGAAGGCAACGAGCGTCGTCTCACTGGAAAACACGAGACTGCTGATATCAACACTTTCTTATGGGTAAGAAGACTTTTCTTTAAAATGTATCATTGAATATTACTCCACATTTACACGTTACAGTATGTGAAAGTTGATTATTATATCACATATGCCTAAAGCATGATATAACTTAAACCGGTTTTGTACAGGGTGTTGCAAACCGTGGGGCATCGATTAGGGTTGGTCGTGACACTGAGAAAGATGGGAAAGGATACTTTGAAGATCGTAGGCCAGCGTCGAACATGGATCCATACACTGTAACCTCCATGGTTGCTGAAACCACAATCCTCTGGAAACCATGAATGAGGAATAGACAATAGAAGAATCAATATGAAACAGTTCTCATGTGTTCTTTGTTTAAGAATGTTTGATTTAAACTCTTTCCAAGAAATAATGATAGTCTTTTCTCTAGCTTTTACTTTGTTGTATTTCAGAATCTATATGTATTTACAAGATTCAAGTCTCGGTTTATTCAGTTTTTGACATGAGCCAAAACCGAAGTTTACAAACTTCTCCGTTACTCTGTGGCCCAAACTAGACCCGACTCGAGGGTAACCGAAGATGGATAAA

>*BnaA.GLN1.4.a*

ATATAAATACACTTGCAGGAATCTCCTTAGTGTTATACAAAAAAAGTTTAGTTTTCTGTTCAGATTCCTAGAAAAATGTCGGCTCTTGCAGATTTAATCAATCTCGATCTCTCCGACTCCTCTGAGAAGATCATTGCCGAGTACATATGGTCCGTAATATTATTTCTTTTTTTTTTATTTAATCTACAACAGTATATTATTTGCTTATGTGTTTGAGTTGGCGTTTGTGTCTATAGTTCTCATCCTTGAGGTTTTCTTATGACTCAAAACTTTGAAATTTTGCTTGATATACTAAAAGTTGAAAGATATTGGATTGATTGAATATTTTGATTAATATTTTCGTGATTTGATGAATAGGATTGGTGGATCAGGCTTGGATATGAGAAGCAAAGCAAGGGTAATTAATTTATTTTGAATTTTTTTTTTGTATAGTGTAAACGATGAGTAAATCGTTAAAGTAATATCACGAATTTAGGACATGATTAATAATATTATTGCTGCAGACTTTGCCGGGACCAGTGAAGGATCCATCGGAGTTACCGAAATGGAACTATGACGGTTCAAGCACCGGCCAAGCCCCCGGCGATGACAGTGAAGTCATCATCTAGTATGTTCCATAATTAAATCAATTAATAATTTAAGTAACTTTTTCTAATTCTTGGATTAATGAGATTGTGGGTTTTCTATGTGTTTTTTTTTTCAGCCCTCAAGCTATCTTCAAAGATCCATTCAGAAGAGGCAACAACATCCTTGTAATAACTCATTTAAACAACTAGAACAATACTCTCGAAATTTGTTGCAATATTCATTTATTTTGTTTCTGTGATTGATTTGGAATTAGGTGATGTGTGACGCTTATACACCGGCTGGCGAACCGATCCCAACCAACAAAAGGCATGCGGCGGCCAAGATCTTTAGCGACCCAACCGTTGCCGCCGAAGAAACATGGTATACATCTTCACAATGAAATTTTTTTTGTCAAAGTTGATCTCAATTTTTTTGTTTGAAAACTTTATGTTTTCATAGTTAATTTTTTGTCAACATCATCATGCGAGTTTAATTCCAAATGGTAACGCAAGGAGACGTAACATGATGTAGTCAACTTTGTTGATCTATATTTTTATAACGTAAAAGTATAATTAAATAATTTGACCATAAACACATTTGTTACTCTAAATTTACATTTTTCTAAATTAATTAAATATTTCAAATATATTGAAATTCAGGTACGGAATTGAGCAAGAGTATACTTTGCTCCAAAAGGATACTAAGTGGCCAGTTGGTTGGCCCGTCGGTGGCTTCCCAGGTCCTCAGGTAAAGATTTTCTCGGCGAATCTTTTCACTTCTTTATCTCAATCCAAAACTCCAGTTTGTGCCAAATAAGTAAAAAAATATTTATTAATAAAAAAAAACAGGGACCATACTACTGTGGAGTTGGAGCAGACAAAGCCTTTGGAAGAGACATCGTAGATGCTCATTACAAAGCATGTCTTTACGCCGGAATCAATGTCAGTGGCACTAACGGAGAAGTCATGCCCGGCCAGGTTTAATATATTCCCTATGTATTCTTCTGATTCAATATCATATTGACCGTGAAACTGATATTGTGTGGGTTTTCAGTGGGAGTTCCAAGTCGGTCCAACCGTTGGAATAGCTGCCGCTGATCAGGTCTGGGTCGCTCGTTACATCCTCGAGGTATATATAAACACACTCATCAAGACCTGTCTCTTCATATACTCTGTTTTATGACTATGTTTCTTGGGTATGTTAAAAACAGAGGATCACAGAATTGGCTGGAGTGGTTTTGTCTCTTGACCCTAAACCAATTCCGGTTAGATTTACCATATTTTCAATTATTTACAAAATAGACCTTTGATTCTTTTAAATTCACTGAATTACTCTATTTTAATTTTTATATTCTAGGGAGATTGGAATGGTGCAGGAGCACACACAAATTACAGGTCTGCTATGATTCCGTTTATATGTTTTTTTCTCTGTGTTTTTTTTTGTTTTCTGATTTTGGTTTTCTTGTTTTCTGCAGCACCAAGTCGATGAGGGAAGATGGAGGGTACGAGATCATAAAGAAAGCGATAGAGAAGCTTGGACTGCGTCACAAGGAACACATCGCTGCTTATGGTGAAGGCAACGAGCGTCGTCTCACTGGAAGACACGAGACTGCTGATATCAACACTTTCTTATGGGTAAGACTTTTCTTAAAATGTATACATTATTAGCCTAAAGCATGATCTAACTTAAACCGGTTTTGTAAAGGGTGTTGCAAACCGTGGGGCATCGATTAGGGTTGGTCGTGACACAGAGAAAGATGGGAAAGGATACTTTGAAGATCGTAGGCCAGCGTCGAACATGGATCCCTACACTGTGACCTCCATGGTTGCTGAAACCACAATCCTCTGGAAACCATGAAGGAAGAAACCTTGAGTCTCAAGGAACCTCTTATATCAGTTCATGTTGATTCTTCTATTGTCTATTCCTCTTTATGAAACACTTCTCATGTGTTCTTTGTTTAAGAATGTTTGATTTAAACTCTTTCCAAGAAATAATAGTAGTTCTTTTCCCTAGCTTTTACTTTCTTCTATTTCAGAATCTATATGTTATTACAAGATTCAAGTCTCGGTTTAGTCAAGTTTTTGACATTAACTTAAACCGAAGCTTACAAACTTCTCCATTACTAGGCGGCCCAAACTAGACCCGACTCGACGGTAACCGAAGATGGATAAA

>*BnaC.GLN1.5.a*

TATATAAAAAGTGAGTTAGCGAAGCAGAGTGAGCCAATGCTCACCTCAGACTGATTATTATAACTCGATCGTCTTCTTCTTCGGCTTGATGGAAACAGAAAGAATGTCTCCACTCTCAGATCTCCTGAACCTCAACCTCGACACCAAGCAAATCATCGCTGAATACATATGGTTCGATCATTTTCCCTGATCATGTCACTCTTTTGTGTTTGCTTAATCGCTTGATTGCTATGTCCCGTTTTGGACATGAACACTGCTGCATCTCATCTAGATTTCTCGAACTTTTTTTTTTCTGTAGATTTCTCGATCTTACTGCATGCGTATGTTTTTTTCTTTTTTTATCGAAGCATGCGCATGTTCTTACATGGCTCTGTTAACAACATTAAGTTTTTAATCTTGTCTTCGTGAGAACAGGATCGGTGGGTCTGGAATGGACATTAGAAGCAAAGGCAGGGTACGAATAATAATATTTACACTATTTTCTCTTTTATTTTAACTCTAAAATCAAAAACTTTTTTTTTAATACAGACATTACCAGGACCTGTAAGTGATCCATCAAAGCTTCCGAAATGGAACTACGATGGATCCAGCACCAATCAAGCCGCCGGAGATGACAGTGAAGTCATTCTATAGTAATCTTCTTCACATAAATTATTTTCAAGTATAATGAATTAAAACTTATTATTATTAACTATTATATTTGTTTAATGTTATCTTTGAAATGAATCTAAAAAAGTCCTCAGGCGATTTTTAAAGACCCGTTCAGGAAAGGGAATAACATTCTCGTAAGCTCATCTGATTTTCTTTAGCAAAAGATTCATCTGATTATTTTTTAGTTGGAGGATTTTATGAATTCAAAATTTCTTTAATAATTATAATTAAATCAGGTGATGTGTGATGCTTACACACCGAAAGGAGATCCAATCCCGACCAACAATAGGCACAAAGCCGTGAAAATCTTCGATCATCCCAATGTGAAGGCTGAAGAGCCTTGGTAAGATCATTCCATATTTCACTTTGAGTCTTGGTTTGGAGCCGGTTTAGTTTAGGACAAATATATATTTTAGTATCTGTTAATTGTCTACAAAAATTGTTTTCTTGAAAATTGTGGTTGATTCAGTTCGGTTCGGTATGACTAATATCGGGTTAAGTTACTTTCGGTTCGGTGAAAACATTTGATAACCGATCGGTTGATGAATATATAATTCTTAGTTTGGTTAATTTCATAATTATAATTCGGTTTGATATTTGGATGTGTTATTTATTAGGTTTGGGATAGAGCAAGAATACACATTACTTAAGAAAGACGTCAAGTGGCCATTGGGTTGGCCCCTTGGTGGCTTTCCTGGTCCTCAGGTACACTAATGCTTAACATTTCTCCTTACAAATTCATAGACTAAACTCTTAATATGAAGAACATGTTTCTGATATCGAATATCTCTCCTCTTTTTTTGTGTGTGCGTGTTTGATGAAAACAGGGACCGTACTATTGTGCGGTCGGTGCAGACAAAGCCTTTGGTCGTGACATTGTGGATGGTCACTACAAAGCTTGTCTTTACGCTGGTTTAAGCATAGGTGGTGCCAATGGTGAAGTCATGCCTGGTCAATGGGAGTTTCAAATCAGCCCTACTGTTGGTATTGGTGCAGGTGATCAGTTATGGGTTGCTCGCTACATACTCGAGGTGAATAAAAAAACGATATACATAAAGCATTTTTGTTTCTTTGTACTAAGTAGCATTTTTTTTTTGTATTTCTCTTATGAATCATTTAGTGTTTATTTTGGCAGAGGATTACTGAGATATGCGGCGTGATTGTCTCATTTGATCCCAAACCAATCGAGGTATGGATCATCAAGACTCTTTTTTTTTGTAGTTATCATGGATTTTTTTTGTTTGAGTTGGGTTTGTTATCTATGGCGGTTTGATAATGATTAGGGTGATTGGAACGGAGCAGCTGCTCATACAAACTTCAGTACAAAATCAATGAGGAAAGAAGGAGGATTGGACTTGATAAAAAAAGCAATAGGGAAGCTTGAAGTGAAGCATAAACAACACATTGCTGCTTATGGTGAAGGCAATGAGAGGCGCCTCACTGGGAAGCATGAAACCGCAGACATCAACAAGTTCTCTTGGGTATGAGATATCGAATAAAATCAAATATATAATTTTGGATTAGTGATGCTTATACAAGCTTTCTTGATGTGTGTAATTTGGCTTTGTTGGGAGTAGGGAGTTGCGGATCGTGGAGCATCGGTGAGAGTGGGAAGAGATACGGAGAAAGAAGGGAAAGGGTATTTTGAAGATCGAAGGCCTTCGTCTAATATGGATCCTTATCTTGTTACCTCCATGATAGCTGAAACCACCATCCTCGGCTAAGCTTTCGTTTGAAGTTGTTGCATACGTTCTTTTGTTTCTTCATGTTTCGGTTTAATTTCAGTTTGAGACTCTTTTTTTTATTGCTAATAATTCATGGGATGGTCTTGATCCTATTGTTTGTTTATCCTGGTTCAGTTGTTTGTGTTAAAACAAAATTGAATTGGGAAAATTGAATTGGCAAAATAAA

> *BnaA.GLN1.5.a*

TATATAAAAAGTGAGTGAGCGAAGCAGAGTGAGCCAGTGCTCACCTCAGACTGATTATTATAACTCGATCGTCATCTTCTTCGGCTTGATGGAAACAGAAAAAATGTCTCCACTCTCCGATCTCCTAAACCTCAATCTCGACACCAAGCAAATCATCGCTGAATACATATGGTTCGATCATTTTCCCTGATCATGTCACTCTTTTGTGTTTGCTTAATCTCTTGATTGCTATGTCCCGTTTTGGACATGAACACTGCTGCATTTCATCTAGATTTCTCGATCTTTTTTTTTTGTTCTGTAGATTTCTCGATCTTACTGCATGCGTATTTTTTTTTTTATCAAAGCATGCGCATGTTCTTACATGGCTCTGTTAACAACATAAAGTTTTTATTCTTGTCTTCGTGAGAACAGGATCGGTGGGTCTGGAATGGACATTAGAAGCAAAGGCAGGGTAAGAATAATAATATTTACACTATTTTCTCTTTTATTTTAACTCTATAATCAAAAAACTTTTTTTTTAATACAGACATTACCAGGACCAGTAAGTGATCCATCAAAGCTTCCGAAATGGAACTACGATGGATCCAGCACCAATCAAGCCGCCGGAGATGACAGTGAAGTCATTCTATAGTAATCTTCTTCACATAAATTATTTTCAAGTATAATGAATTAAAACTTATTATTATTAACTATTATGTTTGTTTAATGTTATTTTTGAAATGAATCTAAAAAAGTCCTCAGGCGATTTTTAAAGACCCATTCAGGAAAGGGAATAACATTCTCGTAAGCTTATCTGATTTTCTTTAGCAAAAGATTCATCTGATTATTTTTTTTGTTGGAGGATTTTATGAATTCAAAATTTCTTTAATAATTATAATTAAATCAGGTGATGTGTGATGCTTACACACCGAAAGGAGATCCAATCCCGACCAACAATAGGCACAAAGCCGTGAAAATCTTCGATCATCCCAATGTGAAGGCTGAAGAGCCTTGGTAAGATCATTCCATATTTCACTTTGAGTCTTGGTTTGGAGCCGGTTTGGTTTAGGACAAATATATATTTTAGTATCTGTTAATTGTCTACAAAAATTATTTTCTTGAAAATTGTGGTTGATTCAGTTCGGTTTGGTATGACTAATATCGGGTTAAGTTTAACTTTCGGTTCGCTGAAAACATTTGATAACCGATCGGTTGATGAATATATATAATTCTTAGTTTGGTTAATTTCATAATTATAATTCGGGTTGATATTTGGATGTGTTATTTATTAGGTTTGGGATAGAGCAAGAATACACATTACTTAAGAAAGACGTCAAGTGGCCATTGGGTTGGCCCCTTGGTGGCTTTCCTGGTCCTCAGGTACACTAATGCTTAACATTTCTCCTTATAAATTCATAGACTAAACTCTTAATATGAAGAACATGTTTCTGATATCAATATCTCTCCCTTTTGTGTGTGTGTGTGTGTCTGTGTGTGTGTTTGATGAACAGGGACCGTACTATTGTGCGGTGGGTGCAGACAAAGCCTTTGGGCGTGACATTGTGGATGGTCACTACAAAGCTTGTCTTTACGCTGGTTTAAGCATAGGTGGTGCCAATGGTGAAGTCATGCCTGGTCAATGGGAGTTTCAAATCAGCCCTACTGTTGGTATTGGTGCAGGTGATCAGTTATGGGTTGCTCGCTACATACTTGAGGTGATTAAAAAACGATATACATAAAGCATTTTTATTTCTTTGTACGAAGTAGCATTTTTTGTATCTCTTATGAATCATTTTGTGTTTATTTTGGCAGAGGATTACTGAGATATGCGGCGTAATTGTCTCATTTGATCCCAAACCAATCGAGGTACGGATCATCAAGACTTTTTTTTTTTGTAGTTATCATGGTTTTTTTGTTTTTGAGTTGATTTTGTTATCTATGGCGGTTTGATAATGATTAGGGTGATTGGAACGGAGCAGCTGCTCATACAAACTTCAGTACAAAATCAATGAGGAAAGAAGGAGGATTGGACTTGATCAAGAAAGCAATAGGGAAGCTTGAAGTGAAGCATAAACAACACATTGCTGCTTATGGTGAAGGCAATGAGAGGCGTCTCACTGGGAAGCATGAAACCGCAGACATCAACAAGTTCTCTTGGGTATGAGATATTGAATAAAATCAAATATATAGTTTTGGATTTGTGATGCTTTATACAAGCTTTCTTGATGTGTGTAATTTGGTTTTGTTGGGAGTAGGGAGTTGCGGATCGTGGAGCATCGGTGAGAGTGGGAAGAGATACGGAGAAAGAAGGGAAAGGTTATTTTGAAGATCGAAGACCTTCGTCTAATATGGATCCTTATCTTGTTACCTCCATGATAGCTGAAACCACCATCCTCGGCTAAGCTTTCTTTTGAAGTTGTTGCATACGTTCTTTTGTTTCTTCATGTTTCGGTTTAATTTCGGTTTGAGACTTTTTTTTTTATTGCTAATAATTCATGGGATGGTCTTGATCCTATTGTTTGTTTATCCTGGTTCAGTTGTTAGTGTTAAACAAAATTGAATTGGGAAAA
