# Supplementary material for: Sixteen cytosolic glutamine synthetase genes identified in the Brassica napus L. genome are differentially regulated depending on nitrogen regimes and leaf senescence
Source: J Exp Bot. 2014 Feb 24;65(14):3927–47. doi: 10.1093/jxb/eru041 (PMC4106436; doi:10.1093/jxb/eru041)
Supplement: Supplementary Data [file supp_eru041_jexbot114215_file005.docx]

Deduced *BnaGLN1* mRNA sequences

>mRNA.BnaA.GLN1.1.a

TTATATAAAAAAACGGAGGGAGTAGTATTTAAAAGAACTGATAAATTTTTGATAAGTAGTATCTTCATAATTCCTACCGCCGCTTTTTAGAAATATTATAGGGAAATGGATTTTACCTTAAATAATGTTTTTTTCCGATATCTCATGGAAAGTGTCACCCTGTGTGTACATGTCTGAAAAAAATAAAAAATACATAAGTATATGTTAACTTTGTAAACATTACTAAATATATAAGATTCCATTGGGCCGCAGGAGAATCTTTTATATTTTATTTCTGATTTTTACGTTTTGTAATTTTGGTATTTTGTGGAGAGCCAGAAAGTCTTCTAACTCTTCACGTCACCAACTTTCTCGATCTCTGCACCCTCTTATTCTATAAGTACTCTTCACATCGACCAATATCAATACACAACCAAAACACGAGAAGATTTTATTCGATTTTATCTGTCATTTGCTTCAACTGTTACTATCGACCAATCTCGGTGTTCGTAGCCATGAGTCTTCTTACAGATCTCGTTAACCTTAACCTCTCAGAGACCACTGACAAAATCATTGCGGAATACATATGGGTTGGAGGTTCAGGAATGGATATGAGAAGCAAAGCCAGGACTCTTCCTGGACCAGTGAGTGACCCTTCGGAGCTACCAAAGTGGAACTATGATGGCTCAAGCACAGGCCAAGCTCCTGGTGAAGACAGTGAAGTCATCTTATACCCTCAAGCCATCTTCAAAGATCCTTTCCGTAGAGGCAATAACATTCTTGTCATGTGCGATGCTTACACTCCAGCGGGCGAACCAATCCCAACAAACAAAAGACACGCTGCGGCTAAGGTCTTTAGCCACCCTGATGTTGTAGCTGAAGTGCCATGGTATGGTATTGAGCAAGAGTATACTTTACTTCAGAAAGATGTGAACTGGCCTCTTGGTTGGCCTATTGGCGGCTTCCCTGGCCCTCAGGGACCATACTATTGTAGTGTTGGAGCAGATAAATCTTTTGGTAGAGACATCGTTGATGCTCACTACAAGGCCTGCTTATACGCTGGCATCAATATTAGTGGCATCAACGGAGAAGTCATGCCTGGTCAGTGGGAGTTCCAAGTTGGTCCAGCTGTTGGTATCTCGGCCGGTGATGAAATTTGGGTCGCACGTTTCATTTTGGAGAGGATCACAGAGATTGCTGGTGTGGTGGTATCTTTTGACCCAAAACCGATTCCCGGTGACTGGAATGGTGCTGGTGCTCACTGCAACTATAGTACCAAGTCAATGAGGGAAGATGGTGGTTACGAGATTATCAAGAAGGCAATTGATAAACTGGGACTGAGACACAAGGAACACATTGCTGCTTATGGTGAAGGCAATGAGCGTCGTCTCACGGGTCACCACGAGACTGCTGACATCAACACTTTCCTCTGGGGTGTTGCGAACCGTGGAGCATCAATCCGTGTAGGACGTGACACAGAGAAAGAAGGGAAAGGATACTTTGAGGATAGGAGGCCAGCTTCGAACATGGATCCTTACATTGTGACTTCCATGATTGCAGAGACCACAATCCTCTGGAAACCTTGATCAGATCAAAGAAGCTTGTTGAAGAATGTTCACTCCGTTTGGGTTTCTTGCATGGTTCAACGTTTGTGTGTTTCTCTATCAAGCATTGTCTCAGAGCAAGTCAAGAGATTTGCTTTGTTCTTATGGCTTTTATTGTTTCACATCCATTGAAAACATCTCTTTGTATCAATTTATGAATAAA

>mRNA.BnaC.GLN1.1.a

CTTTAGTGAATGTGGATGAATGTAGAAAGATAGTACTGGAAAAAATAACTTATAGGTTTAAGAAATCAAAGACGAAGAGAATCCGCATTGCATTTGGAGTTGGAAGCGCTAAGCTGGACAACGGAAAACATGAGGATAAAAGAAAGCTTCAGAACCTTGGCGCGGACTCAAAACATAAAGACCGACAATTTTGCACGCGGTGCTCGGAGTCATCCGCTGCCATGAGTCTTCTTACAGATCTCGTTAACCTTAACCTCTCAGAGACCACTGACAAAATCATTGCGGAATACATATGGGTTGGAGGTTCAGGAATGGATATGAGAAGCAAAGCCAGGACTCTTCCTGGACCAGTGAGTGACCCTTCGGAGCTACCAAAGTGGAACTATGATGGCTCAAGCACAGGCCAAGCTCCTGGTGAAGACAGTGAAGTCATCTTATACCCTCAAGCCATATTCAAAGATCCTTTCCGTAGAGGCAACAACATTCTTGTCATGTGCGATGCTTACACTCCAGCGGGCGAACCAATCCCAACAAACAAAAGACACGCTGCGGCTAAGGTCTTTAGCCACCCTGATGTTGTAGCTGAAGTGCCATGGTATGGTATTGAGCAAGAGTACACTTTACTTCAGAAAGATGTGAAGTGGCCTGTTGGTTGGCCTATTGGTGGCTTCCCCGGTCCTCAGGGACCATACTATTGTAGTGTTGGAGCAGATAAATCTTTTGGTAGAGACATTGTTGATGCTCATTACAAGGCCTGCTTATACGCTGGCATCACCATTAGTGGCATCAATGGAGAAGTCATGCCGGGTCAGTGGGAGTTCCAAGTTGGTCCAGCTGTTGGTATCTCGGCCGGTGATGAAATTTGGGTCGCACGTTACATTTTGGAGAGGATCACAGAGGTTGCTGGTGTGGTGGTATCTTTTGACCCAAAACCTATTCCCGGTGACTGGAATGGTGCTGGTGCTCACTGCAACTATAGTACCAAGTCAATGAGGGAAGATGGTGGTTACGAGATTATCAAGAAGGCAATCGATAAACTGGGAATGAGACACAAGGAACACATTGCTGCTTATGGTGAAGGCAATGAGCGTCGTCTCACGGGTCATCACGAGACTGCTGACATCAACACTTTCCTCTGGGGTGTTGCGAATCGTGGAGCATCAATCCGTGTAGGACGCGACACAGAGAAAGAAGGGAAAGGATACTTTGAGGATAGGAGGCCAGCTTCGAACATGGATCCTTACATTGTGACTTCCATGATTGCAGAGACCACAATCCTCTGGAAACCTTGATCAGATCAAAGAAGATTGTTGAAGAATGTTCACTCCATTTGGGTTTCTTGCATGGTTCAACGTTTGTATGTTTCTCTATCAAGCATTGTCTCAGAACAAAGTCAAGAGATTTGCTCTGTTCTTATGGCTTTTATTGTTTCACATCCATTGAAAACATCTCTTTGTATCAATTTATGAATAAA

>mRNA.BnaA.GLN1.2.a TATAAATAGAAATAAAACAGTATTTTTTGGTTACTTTCATTTTTGGTGTTTTGGAGTACATTAGTTTTTTGTCCAATTTATTTTGCGTATTTTTCTTTGTAAATATATGATCTACCTTTTAATTTTTTGAACGATGATATTACGTCTATTATATGCTAATCATATTTATCGTGTTTCATCCATTTGATATTTTTATTTTCAGCGTCTTCTGCTCTTATATTCTTACGTCTGTAATAAGCTAATTAGTAAATTATACGTCTATTATATTTTGATTTTCATCCATTTGATATTTTATATTTATCATTCGGACTTTTTAAGTCTGTTTTTTTCCACAAACGATACTTACAAAACATTTTTGGTCCAAAATCGGTGAAAAAACGGTTTTTTTATTTTTCCTCGCCTATTTGGCCAACTTCCTTGTAAGACAAAAGTATGAACGGTTACTTTATCATGCTAAAGTTATTATTACCCTAGAATACCTACTCAGCTACTCTTACAAGAAAATCAACGCATACATAACTTCTCATAAATTATCCACCTACTTGAATATCAAATGTACAATATACAGTAATCAGAGAATAAGTCAATAATGAATTATTTTGATTCTTGTAAAAAGTCAACAATCAAATTTAAGGACGACACGAAAACAAAAATCACCATTAAACTATATAACTTCAATTTAATACATTGGTTAGATAAAAAGAAATCGCGTTAATATATTTTATGGTTTTAATTTAAAAATAATATTTGGAAAACAGAGGCTCTGCTCTCTCTCCCTATCTCTGTAGTATGTACCCTCGTTGCTCTCTATAAGTACTCCCACAACCACGAACTCCAAAACATCATCTCATAAACCAAAAACCACATTATCCGAGATTTGAGTATATTTCATTGCAACCTTCTTGTCATTTTCTCTGTAACCATGAGTCTTCTGACCGATCTCGTTAACCTTGACCTCTCAGACAACACTGAGAAAATCATCGCTGAATACATATGGGTTGGTGGTTCAGGAATGGATATGAGAAGCAAAGCCAGGACTCTCCCTGGACCTGTGACCGATCCATCAAAGCTCCCAAAATGGAATTATGATGGTTCAAGCACTGGCCAAGCTCCTGGTGAAGACAGTGAAGTGATCTTATACCCTCAAGCGATTTTCAAAGATCCGTTCCGTAGAGGCAACAACATTCTTGTCATGTGTGATACTTACACCCCTGCGGGTGAACCAATCCCTACGAACAAGAGACATGCTGCAGCTCAGATCTTTAGCAACCCTGATGTTGTTGCTGAAGTGCCATGGTATGGAATCGAACAAGAATACACTCTGTTGCAGAAAGATGTGAATTGGCCTGTCGGATGGCCCATTGGTGGATTCCCCGGCCCTCAGGGACCATACTACTGCAGTGTTGGAGCTGACAAATCTTTTGGAAGAGACATTGTTGATGCTCACTACAAGGCTTGTTTGTATGCTGGAATTAACATCAGTGGAATCAATGGAGAAGTCATGCCTGGTCAGTGGGAGTTCCAAGTCGGACCGTCGGTTGGTATCTCAGCTGCTGATGAAGTGTGGATTGCTCGTTTTATTTTGGAGAGGATCACAGAGATTGCTGGTGTGGTTGTATCTTTTGACCCAAAACCAATTCCGGGTGACTGGAACGGAGCTGGTGCTCACACCAATTACAGTACTAAATCGATGAGGGAGGAAGGAGGATACGAGATAATCAAGAAGGCAATTGATAAGCTCGGACTGAGACACAAGGAGCACATTTCTGCTTACGGTGAAGGCAACGAGCGTCGTCTCACTGGACACCATGAAACTGCTGATATCAACACTTTCAAATGGGGTGTTGCAAACCGTGGAGCATCAATCCGTGTAGGACGTGACACGGAGAAGGAAGGGAAAGGATACTTTGAGGATAGGAGGCCAGCTTCCAACATGGACCCTTACACTGTAACTTCCATGATTGCAGAGACTACACTTCTTTGGAATCCTTGAAGAAATATGACAATAATATAACTTGAATCTGGTTTCAAGTTTGTGTTTCTACAGTTTATTAAGCAATTACCGGGTTGATACTGCCGGAGTTTGTGGTTTGAGGCCTTTCTTTTAATCTCTTTGTGTTTTGGGGTTTGTGATTGGAGCAAAAGCCCTGATTTGCTCTGTTTCTTTGACCTTTTATTTGAACCCTTTGTATTTGTATTAATAAGACGATCTGAAAAGGCCTTTTCATGTTTCTATCTGAGACTGCTCAACAAGTTCACAAATAACAAAACTCTTTGGTAGGCCGAGAACCTGTAAGCTAGACGGGTCCATCTATAAATATATTGGTACACACATAAATGTATTCTAGGCACCGTATAAAATACAATCATAGATCATATTCAATCCGGAGAAACATGTCTTTCTCGAACAATAGATGGAACCTAACTAATTAGAAATTGGGCATTTGAAATGTTTGTAATGCGAGTATCAAGTCCTCAATCAAACTTGAATTGGCATGATGTTGCTGGTTGCACCTCCAAGAAAATGACAACTCGATCACCTTATCCTTTGGATCATGCGATGAGAGTCCCAAGATTGTGCAGTCTT

>mRNA.BnaC.GLN1.2.a

TAATTATACGTCTATTATACTTTGATTTTCATCCATTTGATATTTTATATTTATCATTCGGACTTTTGTAAGTCTAATTTTTTCCACAAACGCTAATTATTTACAAAACATTTTTGGTCAAAAATCGATGAAAAAAAAATGGTTTCTATTTTTCCTCGCCTATTAGGCCAACCTCGTAGAAAAAGTATGAACGGTTACTTTATAATGCTAAAGTTATTATTACCCTAGAATACCTACTCAGCTACTCTTACAAGAAAATCAACGCATACATAACTTCTCATAAATTATCCACCTACTTGAATATCAAATGTACAATATACAGTAATTTGGGAATAAGTCAATAATTATTTGTTTTTAATTTTTGTAAAAAGTCAATAATCAAATTTAAGGACGACACGAAAACAATAATCACCATTAAACTATATAACTTCAATTTAATACATTGGTTAGATAAAAAGAAATCGCGTTATTATATTTTATGGTTTTAATTTAAAAATAATATTTGGAAAACAGAGGCTCTGCTCTCTCTCTCCCTATCTCTGTAGGTACCCTCGTTGCTCTCTATAAGTACTCCCACAACCACGAACTCCAAAAAATCATCTCATAAACCAAAAACCACATTATCCGAGATTTGAGTATATTTCACTACAACCTTCTTGTCATTTTCTCTGTAACCATGAGTCTTCTGACCGATCTCGTTAACCTTGACCTCTCAGACAACACTGAGAAAATCATCGCTGAATACATATGGGTTGGTGGTTCAGGAATGGATATGAGAAGCAAAGCCAGGACTCTCCCTGGACCTGTGACCGATCCATCAAAGCTCCCAAAATGGAATTATGATGGTTCAAGCACTGGCCAAGCTCCTGGTGAAGACAGTGAAGTGATCTTATACCCTCAAGCGATTTTCAAAGATCCGTTCCGTAGAGGCAACAACATTCTTGTCATGTGTGATACTTACACCCCTGCGGGTGAACCAATCCCTACGAACAAGAGACATGCTGCAGCTCAGATCTTTAGCAACCCTGATGTTGTTGCTGAAGTGCCATGGTATGGAATCGAACAAGAGTACACTCTGTTGCAGAAAGATGTGAAGTGGCCTGTTGGATGGCCCATTGGTGGATTCCCCGGCCCTCAGGGACCATACTACTGCAGTGTTGGAGCTGACAAATCTTTTGGAAGAGACATTGTTGATGCTCACTACAAGGCTTGTTTGTATGCCGGAATTAACATCAGTGGAATCAATGGAGAAGTCATGCCTGGTCAGTGGGAGTTCCAAGTCGGACCATCGGTTGGTATCTCAGCTGCTGATGAAGTGTGGATTGCTCGTTACATTTTGGAGAGGATCACAGAGATTGCTGGTGTGGTTGTATCTTTTGACCCAAAACCAATTCCGGGTGACTGGAACGGAGCTGGTGCTCACACCAATTACAGTACTAAATCGATGAGGGAGGAAGGAGGATACGAGATAATCAAGAAGGCAATTGATAAGCTCGGACTGAGACACAAGGAGCACATTTCTGCTTACGGTGAAGGCAACGAGCGTCGTCTCACCGGACACCATGAAACTGCTGACATCAACACTTTCAAATGGGGTGTTGCAAACCGTGGAGCATCAATCCGTGTAGGACGTGACACGGAGAAGGAAGGGAAAGGATACTTTGAGGATAGGAGGCCAGCTTCCAACATGGACCCTTACACTGTAACTTCCATGATTGCAGAGACTACACTTCTTTGGAATCCTTGAAGAAATATGACAATAATATAACTTGAATCTGGTTCTCTGGGGCTTCAAGTTTGTGTTTTCTACAGTTTATTAAGCAATTACCGGGTTGATACTGCCGGAGTTTGTGGTTTGAGGCTTTCTTTTAATCTCTTTGTGTTTTGGGGTTTGTGATTGAAGCAAAAACCCTGATTTGCTCTGTTTCTTTGACCTTTTATTTGAACCCTTTGTATTTCTATTAATAAGACGATCTGAGAAGGCCTTTTCATGTTTCAACTTAGACTGTTCAACAAGTTCACAAATAACAAAACTCTTTGGTAGGCCCAGAACCTGTAAGCTAGACTCTAAAACTATTGGTAGTTGGTACACACATAAATGTATTGTAGGAACCGTTATAAAA

>mRNA.BnaA.GLN1.3.a

ATGTCTATTTAGACACATCAGGTTAATGCGAAGAGCAAAACAGGGCGCAACAAAATGGGCTTTATGACAAATTGGAGGAAAACGTTACGTACGCAAAATACAAAAGTGTCCACGTCTTGATTCATCATATGATAATACTTTTATAGTCATTAGTGCAAAACTCATTTCATTTCGTATCTCACTAAACTACGTTTACGTGTTCATCAACAATTCTAATTTTACTGATTTACTCATAAACATATCCTCCAGTTATCCATAATCAGGTTTGTACTAAAAGACAAAAGATATGAAGAATATCAAGAAACTTCTTAGTTTTTGCGAATTGCACCCGTTTCATCATCCTACGATTCATAAAACTTAGCTACAACAAACTAAAAGACCTTCGTCTATCAATAGTGCAACCACTTTTTCTTCATGTAGTAACCACAGAAGAGAATTAGTTCCGTTTGATCATAAATAAACTCCTTTCGCCTGTTCATAATAATAAAGAAACACTTAAAATAATAATTGTAATTATCTCTGTGTAAATGTCAGGAGAATCCTAACGGAATCGTTTATCTAATAAAATTAACTATTAAAAAAATCAAAAGATAATGTTGGCAGACAACAGTAAATAAAATCATGTAATTATTTGAAAATAAAAAATAAATTAAAAAAAACAAAAAAATAAGGCTATAAAATAAAAACACTCGTCAGGAGAGAAGCTGCATTGATCGTCTTCCCTTAGACAAACACTGATTGATTATCTTTCTCTTTTCTCTCGTGACGGCGCCATGTCTCTCCTCTCAGATCTCGTCAACCTCAACCTCTCTGACTCCACCGAGAAAATCATCGCCGAATACATATGGATCGGTGGATCTGGAATGGACATCAGAAGCAAAGCCAGGACACTACCAGGACCAGTGAGCGATCCATCAAAGCTGCCTAAGTGGAACTACGACGGATCCAGCACCGGTCAAGCCGCCGGAGATGACAGTGAAGTCATTCTATACCCTCAGGCGATATTCCGTGATCCGTTCAGGAAAGGCAACAACATTCTGGTGATGTGTGATGCTTACACGCCGGCAGGGAATCCAATTCCGACCAACAAGAGGCACAACGCTGCTAAGATCTTCAGCAACCCCAAAGTTGCCTCTGAGGAGCCTTGGTATGGGATTGAGCAAGAATACACATTGATGCAGAAGGGTGTGAACTGGCCTATTGGTTGGCCTATCGGTGGCTTCCCTGGCCCTCAGGGACCTTACTACTGTGGTGTGGGAGCTGACAAGGCAATTGGTCGTGACATTGTGGATGCACACTACAAGGCCTGTATTTACGCAGGTATTGGCATCTCTGGTGTCAATGGAGAAGTCATGCCTGGTCAGTGGGAGTTCCAAGTCGGTCCTGTTGAGGGTATTAGTGCTGGTGACCAAGTTTGGGTCGCTAGATACCTTCTCGAGAGGATCACTGAGATCTCTGGTGTGAATGTCAGCTTCGACCCTAAACCAGTCCCGGGTGATTGGAACGGAGCTGGAGCTCACTGCAACTACAGCACGAAGTCAATGAGGAACGACGGAGGATTAGCTGTGATTAAGAAAGCGATAGAGAAGCTTCAGGTGAAGCACAAGGAGCACATTGCTGCTTACGGTGAAGGCAACGAGCGTCGTCTCACGGGGAAGCACGAAACTGCAGACATCAACACGTTCTCTTGGGGAGTGGCGAACCGTGGGGCTTCGGTGAGAGTGGGGAGAGACACTGAGAAAGAAGGCAAAGGTTACTTCGAGGACAGAAGGCCAGCTTCTAACATGGATCCTTACGTTGTTACGTCCATGATCGCTGAAACCACCATCCTCGGTTAAACCACACACATTTAGTAATATTTGATTTTCTCCGGTTTGGTTTTATGATTTGAATACTGTTGTACTTGTGATCGCGGTCTATTTCGGTTTCAATAATTCTTATGACGACATTTTGTGTTTTTTCTTCTTCTTAGTTTCGAATAATTAAATAAGGGTATTCATGCGGTGAAAAAA

>mRNA.BnaC.GLN1.3.a

ATTGATCGTCTTCCCCTAAACAACACTGATTGATTATCTTTCTCTCGTGACGGCGCCATGTCTCTCCTCTCAGATCTCGTCAACCTCAACCTCTCTGACTCCACCGAGAAAATCATAGCCGAATACATATGGATCGGTGGATCTGGAATGGACATCAGAAGCAAAGCCAGAACACTCCCAGGACCAGTGAGCGATCCATCAAAGCTTCCTAAGTGGAACTACGACGGATCCAGCACCGGTCAAGCCGCCGGAGATGACAGTGAAGTCATTCTATACCCTCAGGCGATATTCCGTGATCCGTTCAGGAAAGGCAACAACATTCTGGTGATGTGTGATGCTTACACGCCGGCAGGGAATCCAATTCCGACCAACAAGAGGCACAACGCTGCTAAGATCTTCAGCAACCCCAAAGTTGCCTCTGAGGAGCCTTGGTATGGGATTGAACAAGAATACACATTGATGCAAAAGGGTGTGAACTGGCCCATTGGTTGGCCTATCGGTGGCTTCCCTGGCCCACAGGGACCATACTACTGTGGTGTGGGAGCTGACAAAGCCATTGGTCGTGACATCGTGGATGCGCACTACAAGGCCTGTATTTACGCAGGTATTGGCATCTCTGGTGTCAATGGAGAAGTCATGCCTGGCCAGTGGGAGTTCCAAGTCGGTCCTGTTGAGGGTATTAGTGCTGGTGACCAAGTCTGGGTCGCTAGATACCTTCTTGAGAGGATCACCGAGATCTCTGGTGTCAATGTCAGCTTCGACCCTAAGCCAGTCCCGGGTGATTGGAACGGAGCTGGAGCTCACTGCAACTACAGCACGAAGTCGATGAGGAACGACGGAGGATTAGCTGTGATTAAGAAAGCCATAGAGAAGCTTCAGGTGAAGCACAAGGAGCACATTGCTGCTTACGGTGAAGGCAACGAGCGTCGCCTCACGGGGAAGCACGAGACCGCGGACATCAACACGTTCTCTTGGGGAGTGGCGAACCGTGGAGCTTCGGTGAGAGTGGGACGTGACACTGAGAAAGAAGGCAAAGGTTACTTCGAGGACAGAAGGCCAGCTTCTAACATGGATCCTTACGTTGTTACTTCCATGATTGCTGAAACCACCATCCTCGGTTAAAATCGCACACGTTCTTATGTTTGGGTTTATCTTATCTGGTCTGGTTTTCAATTTGCAAACTTGTGCTTGTGATTGCCGGTTTATCTCGGTTTAAATAATTCTTATGACGACATTTTGTATTTTTTTTCTTCCTAGTTTCGAATAATTAAATAAGGTATTCATATGGTGAAAAAAGTCTATCAGCGACTTTTGTGTTGTTGTTTTGAAATGATAATAATAATAACAAAGTACGGCCTGGCGCGTTGGAGGTTAATGAGCTTGTCGGGAGTAGTAAAGTAGGTGGATAAGATCTGGTGATTAATAATTATGAATTTATCTTAAAATTTAAATGTTGAATGTTCTTTACAACATTATGCAGGATGTGGCATGTTACAGAACATCTCCAATGTATACGTAGAAATATCATATTTATATATTTTTTTCTATAAATAAA

>mRNA.BnaA.GLN1.3.b

ATCGATAAAATGAGCAGCACCGAGACATGATGTTCCGACATGATGAATGAAACAGTATTTGATTTCACCTGTTTGAATTGCATCACTTTGAGTTTGTTTTTAAGTATTGGTGTGCAAAAATGACAAGATTTTGTCACACTACTAAAAAAAAGTTTAGCTGTATACATCATGTACATGTATGTTGCATTTTCATGACTATTGGCGTTGCTAGAAAAAAGAACTTGCTTTCAAAATTAACAAATACGTGATGATTGAATCCAACTTAGCATGATAAAAAGAATCATTATGTACGCAAAGACAAAAATATCCAAGACTCGATTAATACAAATCTCGTTTCGTTTATTTACCTAAGTAGTTTTCGTGTTCATCAACAATTCTATTTATACCAATTTAATCATAGATTACATATATTTTAATATTGTTGTCATAATCTGTATCTAAATCACTGGAGAATCTTACCTGAAACAATATAACAATATTAAATAATTGTTTAAAATTCTAAAAGATAATTATTGACACACAACAATAAATAATCACCAATTTACTAGAATCATGTATTGACCACCGTAAATATCTGAAAATTTAAAAAAAAAAACAAAAACAAAAATAGCTCTATAAACACTCGAATAAGTTTTAATGATCGTCTTCCCTAAACAACACTGATTAATTTTAGTATTTTTTTTCTCTCGTATTCTCCTCTCAGACGCAGCCATGTCTCTGCTCTCAGATCTCGTCAACCTCAACCTCTCCGACACAACCAAGCAAATCATCGCCGAATACATATGGATCGGTGGATCTGGAATGGACATTAGAAGCAAAGCCAGAACACTACCAGGACCAGTGACCGATCCATCAAAGCTTCCCAAGTGGAACTACGACGGATCTAGCACCGGTCAGGCCGCTGGAGATGACAGTGAAGTCATTCTATATCCTCAGGCTATATTCCGTGATCCGTTCAGGAGAGGCAACAACATTCTGGTGATGTGTGATGCTTACACGCCGGCCGGTAATCCAATTCCGACCAACAAGAGGCACAACGCTGCAAAGATCTTCAGCAACTCAAAAGTTGCCTCTGAGGAGCCTTGGTATGGGATTGAGCAAGAATACACATTGATGCAAAAGGGTGTGAATTGGCCCATTGGTTGGCCTGTTGGTGGCTTCCCTGGCCCACAGGGACCGTACTACTGTGGCGTGGGAGCTGACAAAGCCATTGGTCGTGACATCGTGGATGCACACTACAAAGCCTGTCTTTACGCAGGTATTAGCATCTCTGGTGTCAATGGAGAAGTCATGCCTGGCCAGTGGGAGTTCCAAGTCGGTCCTGTTGAAGGGATTAGTGCCGGTGATCAAGTCTGGATAGCTAGATTCCTTCTCGAGAGGATCACTGAGATCTCTGGTGTAAACGTCAGCTTCGACCCAAAACCAGTCCCGGGTGATTGGAACGGAGCGGGAGCGCACTGCAACTACAGTACGAAGACGATGAGGAACACTGGAGGATTAGCGGTGATAAAGAAAGCGATAGAGAAGCTTCAGGTGAAGCACAAGCAGCACATTGCTGCTTACGGTGAAGGCAACGAGCGTCGTCTCACGGGCAAGCACGAGACGGCAGATATCAACACGTTCTCATGGGGAGTGGCGAACCGTGGAGCTTCGGTGAGAGTGGGACGTGACACTGAGAAAGAAGGCAAAGGTTACTTCGAGGATCGTAGGCCAGCTTCTAACATGGATCCTTATGTCGTCACTTCCATGATCGCCGAAACCACAATCCTCGGTTAAGTAATCAATACGCATTTTAATGTTTAGTTTTTTCCTTAATTTGCGGATTTTTGTGCTTGTGGTTGCACTACTAATAATTATTATCATGCCCTAGTAGGATTCACGTGTTTTATGTTTTTTTTTTTCATTTCGAATAATTAAATAAGGAATTCTTATGGATGAAATTTAAACAAAAGAATGTATTATTAGTGATTTTTTTTGTTGATGTGGTTGAACTTGTTAGGGATAAGATTTTGTGATTATTGATAACATAAGAAATTAATAAGAAATAAAAA

>mRNA.BnaC.GLN1.3.b

GCGTTGGCTTGAAAAATAAAAACTTGGTTTCAAAATCAACAAATACGAGATGATTGAATCCAACTTAACATGATAGAGTCATTATGTACGCAAAAGACAAAAATGCCAAGACTCGATTCATCATCACAGGCTCACAGTGATGATACCTATATAGGCAAGTAATACAAAACTCGTTTCGTTTAGTTACTTGACTATTTACGTGTTCATAAACAATTCTATTTAACTGATTAAATCATTGATTACATATATGTAAATATTGTTGTCATAATCTGTATTTAAATCACAGGAGATCTTAACTGAAACAATATAATAATACAAATTAATTATTTAAAATTCTAAAAGATAAATATTAGTATCACACAACAATAAAGAAATTACCAATTTACTAGAATCATGTATTGACCACCGTAAATATCTGAAAACAATAATAAATAAATAAACAAAAATAGCTCTATAAACACTCAGAGAAGTTTTAATGATCGTCTTCCCTAAACAGCACTGATTGATTAGTGTATTTTTTTCTCTCGTATTCTCCTCTCAGACGCAGCCATGTCTCTGCTCTCAGATCTCGTCAACCTCAACCTCTCCGACACCACCAAGCAAATCATCGCCGAATACATATGGATCGGTGGATCTGGAATGGACATTAGAAGCAAAGCCAGGACACTCCCAGGACCAGTGACCGATCCATCAAAGCTTCCCAAGTGGAACTACGACGGATCAAGCACCGGTCAGGCCGCTGGAGATGACAGTGAAGTCATTCTATATCCTCAGGCTATATTCCGTGATCCGTTCAGGAGAGGCAACAACATTCTGGTGATGTGTGATGCTTACACGCCGGCCGGTAATCCAATTCCGACCAACAAGAGGCATAACGCTGCTAAGATCTTCAGCAACTCTAAAGTTGCCTCTGAGGAGCCTTGGTATGGGATTGAGCAAGAATACACATTGATGCAAAAGGGTGTGAACTGGCCTATTGGTTGGCCTGTTGGTGGCTTCCCTGGCCCACAGGGACCATACTACTGTGGTGTGGGAGCTGACAAAGCCATTGGTCGTGACATCGTGGATGCACACTACAAAGCCTGTCTTTACGCAGGTATTAGCATCTCTGGTGTCAATGGAGAAGTCATGCCTGGCCAGTGGGAGTTCCAAGTCGGTCCTGTTGAAGGTATTAGTGCTGGTGATCAAGTCTGGATCGCTAGATTCCTTCTCGAGAGGATCACTGAGATCTCTGGTGTAAACGTCAGCTTCGACCCAAAACCAGTCCCGGGTGATTGGAACGGAGCTGGAGCTCACTGTAACTACAGCACGAAGACGATGAGGAACACTGGAGGACTAGCGGTGATAAAGAAAGCGATAGAGAAGCTTCAGGTGAAGCACAAGCAGCACATTGCTGCTTACGGTGAAGGCAACGAGCGTCGTCTCACGGGGAAGCACGAGACGGCAGATATCAACACGTTCTCGTGGGGAGTGGCGAACCGTGGAGCATCGGTGAGAGTGGGACGTGACACTGAGAAAGAAGGCAAAGGTTACTTCGAGGATCGTAGGCCAGCTTCTAACATGGATCCTTATGTCGTTACTTCCATGATCGCTGAAACCACAATCCTCGGTTAAGTAATCGCACGCATTTTATAATGTTTGGTTTATCCGGTTTCGTTTATTTCTTAATTTGCGGATTTTTGTGCTTGTGGTTGCACTACTAATAATTATTATCATGCCCTAGTAGGATTCACGTGTTTTATGTTTTTTTTTCATTTCGAATAATTAAATAAGGAATTCTTATGGATGAAATTTAAACAAAAA

>mRNA.BnaA.GLN1.3.c

TATATATAAACACTCTCAGGAGAGAAGCTGTATCGAGATCGTCTTCCCAAAACAACACTCATTGATTGATTACTATCCGACGCAGCCATGTCTCTGCTCTCAGATCTCGTCAACCTCAACCTCTCCGACTCCACCAAGCAAATCATCGCCGAATACATATGGATCGGTGGATCTGGCATGGACATTAGAAGCAAAGCCAGAACACTCCCAGGACCAGTGAGCGATCCATCAAAGCTTCCCAAGTGGAACTACGACGGATCCAGCACCGGTCAGGCCTCTGGAGATAACAGTGAAGTCATTCTATACCCTCAGGCCATATTCCGTGATCCGTTCAGGAGAGGCGACAACATCCTGGTGATGTGTGATGCATACACACCGGCCGGAGATCCAATTCCGACAAACAAGAGGCACAAGGCTGCTAAGATCTTTAGCCATCCTAACGTTGCCAAGGAGGTGCCTTGGTATGGGATTGAGCAAGAATACACTTTGATGCAAAAGGGTGTGAACTGGCCTATTGGTTGGCCAATTGGTGGCTTCCCTGGTCCTCAGGGACCATACTACTGTGGTGTGGGAGCTGACAAAGCCATTGGTCGTGACATTGTGGACGCACACTACAAGGCCTGTCTTTACGCAGGTATTGGCATCTCTGGTGTCAATGGAGAGGTCATGCCTGGACAGTGGGAGTTCCAAGTCGGTCCAGTTGAGGGTATTAGTTCTGGTGATCAAGTCTGGGTCGCTAGATACCTTCTTGAGAGGATCACTGAGATCTCTGGTGTAAATGTCAGCTTCGACCCAAAACCAGTCCCGGTTAGTGTCTCTTTACCACACTCTCTCAACAACTACAGCACGAAGTCGATGAGGAACAACGGAGGATTAGCAGTGATAAAGAACGCGATAGAGAAGCTTCAGGTGAAGCACAAGGAGAACATTGCTGCGTACGGTGAAGGCAGCGAGCGTCGTCTCACGGGGAAGCACGAGACCGCATACATCAACACGTTCTCTTGGGGAGTGGCGAACCGTGGAGCTTCGGTGAGAGTGGGACGAGACACTGAGAAGGAAGGCAAAGGTTACTTCGAAGACAGAAGGCCAGCTTCTAACATGGATCCTTATGTCGTTACGTCCATGATCGCTGAAACCACCATCCTCGGTTAATCAATCATGCACGTTTTAATGTTTTGGTTTTCTCCGGTCTGGTTTTCAATTTGTGAACTTATGTGCTTGTGATTGCAGTCTATTTAGGTTTAAATAATTCTTATGACGACATTTGGTGTCTTTTTCTTTAGTTTCGAATTATTAGATAAAAGGATTCTTATGGTGAAAAAGATAAGTGTTGTTTGAAAATTGAAATGATAATATTAGTATCAACGTATGGCTCGGCACATTGGGAGGTTGATAGTGTATAACCAGAGAATTATCAAAGAACCTATAAACAGATATTTAGTTATTGTTACTATATATAAATATAATTTCTAAAAACAGTTGTTCTATTGAGCTTTTTGGCAACATAATCTAATGTATACACCACTATCTAGCTTACACCTTCAAGTAAGAAATTTTGTTAGAATTAGCATGAATGGAACACTCTAAAAACATGATCTTCCTGGATTCACAACCCAATTTTTATTTTTATTTTTAAACAAACAACAATTTCATCTAAAACCGTTTTTTCATGTCGACTTTCTTATAACTATATGGGGAGAAGAAGTGAAAGTTTCAGATAATTACAAAAGTATAAAAAACGAGTTATTTTGGTTACGAGCACTAATTCAAAAGTATACAAATTTAAATACAAAAAATTAAATTATAATGTATTATATTTTAAACTATTGAAAAATAAAAA

>mRNA.BnaC.GLN1.3.c

ATAAAAACACTCTCAGGAGAAGAGCTGTATCGAGATCGTCTTCCCTAAACAACACTCATTGATTGATTACTATCCGACGCAGCCATGTCTCTGCTCTCAGATCTCGTCAACCTCAACCTCTCCGACTCCACCAAGCAAATCATCGCCGAATACATATGGATCGGTGGATCTGGCATGGACATTAGAAGCAAAGCCAGAACACTTCCAGGACCAGTGAGCGATCCATCAAAGCTTCCCAAGTGGAACTACGACGGATCCAGCACCGGTCAGGCCTCTGGAGACAACAGTGAAGTCATTCTATACCCTCAGGCGATATTCCGTGATCCGTTCAGGAGAGGCGACAACATCCTGGTGATGTGTGATGCATACACACCGGCCGGAGATCCAATTCCGACCAACAAGAGGCACAAGGCTGCTAAGATCTTCAGCCATCCTAACGTTGCCAAAGAAGTGCCTTGGTATGGGATTGAGCAAGAATACACTTTGATGCAAAAGGGTATGAACTGGCCTATTGGTTGGCCTGTTGGTGGCTTCCCTGGTCCTCAGGGACCATACTACTGTGGTGTGGGAGCTGACAAAGCCATTGGTCGTGACATTGTGGACGCACACTACAAGGCCTGTCTTTACGCAGGTATTGGCATCTCTGGTGTCAATGGAGAGGTCATGCCTGGACAGTGGGAGTTCCAAGTCGGTCCAGTTGAGGGTATTAGTTCTGGTGATCAAGTCTGGGTCGCTAGATACCTTCTTGAGAGGATCACTGAGATCTCTGGTGTAAATGTCAGCTTCGACCCAAAACCAGTCCCGGTTAGTGTCTCTTTACCACACTCTCTCAACAACTACAGCACGAAGTCGATGAGGAACAACGGAGGATTAGCAGTGATAAAGAACGCGATAGAGAAGCTTCAGGTGAAGCACAAGGAGAACATTGCTGCGTACGGTGAAGGCAGCGAGCGTCGTCTCACGGGGAAGCACGAGACCGCATACATCAACACGTTCTCTTGGGGAGTGGCGAACCGTGGAGCTTCGGTGAGAGTGGGACGAGACACTGAGAAGGAAGGCAAAGGTTACTTCGAAGACAGAAGGCCAGCTTCTAACATGGATCCTTATGTCGTTACGTCCATGATCTGAACCACCATCCTCGGTTAATCAGTATGTGCTTGTGATTGCAGTCTATTTAGGTTTAAATAATTCTTATGACGACATTTGGTGTCTTTTTCTTTAGTTTCGAATTATTAGATAAAAGGATTCTTATGGTGAAAAAGATAAGTGTTGTTTGAAAATTGAAATGATAATATTAGTATCAACGTATGGCTCGGCACATTGGGAGGTTGATAGTGTATAACCAGAGAATTATCAAAGAACCTATAAACAGATATTTAGTTATTGTTACTATATATAAATATAATTTCTAAAAACAGTTGTTCTATTGAGCTTTTTGGCAACATAATCTAATGTATACACCACTATCTAGCTTACATCTTCAAGTAAGAAATTTTGTTAGAATTAGCATGAATGGAACACTCTAAAAACATGATCTTCCTGGATTCACAACCCAATTTTTATTTTTACTTTTAAACAAACAACAATTTCATCTAAAACCGTTTTTTCATGTCGACTTTCTTATAACTATATGGGGAGAAGAAGTGAAAGTTTCAGATAATTACAAAAGTATAAAAAACGAGTTATTTTGGTTACGAGCACTAATTCAAAAGTATACAAATTTAAATACAAAAAATTAAATTATAATGTATTATATTTTAA

>mRNA.BnaA.GLN1.4.a

ATATAAATACACTTGCAGGAATCTCCTTAGTGTTATACAAAAAAAGTTTAGTTTTCTGTTCAGATTCCTAGAAAAATGTCGGCTCTTGCAGATTTAATCAATCTCGATCTCTCCGACTCCTCTGAGAAGATCATTGCCGAGTACATATGGATTGGTGGATCAGGCTTGGATATGAGAAGCAAAGCAAGGACTTTGCCGGGACCAGTGAAGGATCCATCGGAGTTACCGAAATGGAACTATGACGGTTCAAGCACCGGCCAAGCCCCCGGCGATGACAGTGAAGTCATCATCTACCCTCAAGCTATCTTCAAAGATCCATTCAGAAGAGGCAACAACATCCTTGTGATGTGTGACGCTTATACACCGGCTGGCGAACCGATCCCAACCAACAAAAGGCATGCGGCGGCCAAGATCTTTAGCGACCCAACCGTTGCCGCCGAAGAAACATGGTACGGAATTGAGCAAGAGTATACTTTGCTCCAAAAGGATACTAAGTGGCCAGTTGGTTGGCCCGTCGGTGGCTTCCCAGGTCCTCAGGGACCATACTACTGTGGAGTTGGAGCAGACAAAGCCTTTGGAAGAGACATCGTAGATGCTCATTACAAAGCATGTCTTTACGCCGGAATCAATGTCAGTGGCACTAACGGAGAAGTCATGCCCGGCCAGTGGGAGTTCCAAGTCGGTCCAACCGTTGGAATAGCTGCCGCTGATCAGGTCTGGGTCGCTCGTTACATCCTCGAGAGGATCACAGAATTGGCTGGAGTGGTTTTGTCTCTTGACCCTAAACCAATTCCGGGAGATTGGAATGGTGCAGGAGCACACACAAATTACAGCACCAAGTCGATGAGGGAAGATGGAGGGTACGAGATCATAAAGAAAGCGATAGAGAAGCTTGGACTGCGTCACAAGGAACACATCGCTGCTTATGGTGAAGGCAACGAGCGTCGTCTCACTGGAAGACACGAGACTGCTGATATCAACACTTTCTTATGGGGTGTTGCAAACCGTGGGGCATCGATTAGGGTTGGTCGTGACACAGAGAAAGATGGGAAAGGATACTTTGAAGATCGTAGGCCAGCGTCGAACATGGATCCCTACACTGTGACCTCCATGGTTGCTGAAACCACAATCCTCTGGAAACCATGAAGGAAGAAACCTTGAGTCTCAAGGAACCTCTTATATCAGTTCATGTTGATTCTTCTATTGTCTATTCCTCTTTATGAAACACTTCTCATGTGTTCTTTGTTTAAGAATGTTTGATTTAAACTCTTTCCAAGAAATAATAGTAGTTCTTTTCCCTAGCTTTTACTTTCTTCTATTTCAGAATCTATATGTTATTACAAGATTCAAGTCTCGGTTTAGTCAAGTTTTTGACATTAACTTAAACCGAAGCTTACAAACTTCTCCATTACTAGGCGGCCCAAACTAGACCCGACTCGACGGTAACCGAAGATGGATAAA

>mRNA.BnaC.GLN1.4.a

TATATAAATACACTTGCAGGAATCTCTTTAGTGTCATACAAAAAAAGTTTAGTATTCTCTTCAGAGTCCTAGAAAAATGTCGGCTCTTGCAGATTTAATCAATCTCGATCTCTCCGACTCCTCTGAGAAGATCATTGCCGAGTACATATGGATTGGTGGATCAGGCTTGGATATGAGAAGCAAAGCAAGGACTTTGCCGGGACCTGTGAAGGATCCATCGGAGTTACCGAAATGGAACTATGACGGTTCAAGCACCGGGCAAGCTCCCGGCGATGATAGTGAAGTCATCATCTACCCTCAAGCTATCTTCAAAGATCCATTCAGAAGAGGCAACAACATCCTTGTGATGTGTGACGCTTATACACCGGCTGGCGAACCGATCCCAACCAACAAAAGGCATGCGGCGGCTAAGATCTTTAGCGACCCAACCGTTGCCGCCGAAGAAACATGGTATGGAATTGAGCAAGAGTATACTTTGCTTCAAAAGGATACCAAGTGGCCAGTTGGTTGGCCCGTCGGTGGCTTCCCAGGTCCTCAGGGACCATACTACTGTGGAGTTGGAGCAGACAAAGCCTTTGGAAGAGACATCGTTGATGCTCATTACAAAGCATGTCTTTACGCTGGAATCAATGTCAGTGGCACTAACGGAGAAGTCATGCCCGGACAGTGGGAATTCCAAGTCGGTCCAACCGTTGGAATAGCTGCAGCCGATCAGGTCTGGGTCGCTCGTTACATCCTCGAGAGGATCACAGAACTGGCTGGAGTTGTTTTATCTCTTGACCCTAAACCAATTCCGGGAGATTGGAATGGTGCAGGAGCACACACAAATTACAGTACGAAGTCGATGAGAGAAGATGGAGGGTACGAGATCATAAAGAAAGCGATAGAGAAGCTTGGACTTCGTCACAAGGAACACATTGCTGCTTATGGTGAAGGCAACGAGCGTCGTCTCACTGGAAAACACGAGACTGCTGATATCAACACTTTCTTATGGGGTGTTGCAAACCGTGGGGCATCGATTAGGGTTGGTCGTGACACTGAGAAAGATGGGAAAGGATACTTTGAAGATCGTAGGCCAGCGTCGAACATGGATCCATACACTGTAACCTCCATGGTTGCTGAAACCACAATCCTCTGGAAACCATGAATGAGGAATAGACAATAGAAGAATCAATATGAAACAGTTCTCATGTGTTCTTTGTTTAAGAATGTTTGATTTAAACTCTTTCCAAGAAATAATGATAGTCTTTTCTCTAGCTTTTACTTTGTTGTATTTCAGAATCTATATGTATTTACAAGATTCAAGTCTCGGTTTATTCAGTTTTTGACATGAGCCAAAACCGAAGTTTACAAACTTCTCCGTTACTCTGTGGCCCAAACTAGACCCGACTCGAGGGTAACCGAAGATGGATAAA

>mRNA.BnaA.GLN1.4.b

TATATATACACTTGCAGGAATCTCTTTAGTGTTATACAAAACAAAGCTAATTATTTTTTTTAGATTCCTAGAGAAAATGTTGGCACTTGCAGATTTGATCAATCTCGATCTCTCCGATTCCACTGAGAAGATCATTGCGGAGTACATATGGATTGGTGGATCAGGCTTGGATATGAGAAGCAAAGCAAGGACTTTGCCCGGACCAGTGAAGGATCCATCGGAGTTACCGAAATGGAACTATGACGGTTCAAGCACCGGCCAAGCTCCCGGCAGCGACAGTGAAGTCATCCTCTACCCTCAAGCTATCTTCAAAGACCCCTTCAGAAGAGGCAACAACATCCTTGTGATGTGTGATGCATATACACCGGCCGGCGAACCGATCCCGACAAACAAAAGGCATGCGGCGGCCAAGATCTTTAGCGACCCGAGCGTTGCCGCCGAAGAAACATGGTATGGAATTGAGCAAGAGTATACTTTGCTACAAAAGGATATTAAATGGCCGGTAGGTTGGCCTGTTGGCGGCTTCCCAGGTCCTCAGGGACCGTACTACTGTGGTGCTGGAGCAGACAAAGCCTTTGGAAGAGACATAGTGGATTCTCATTACAAAGCCTGTCTTTACGCCGGAATCAATGTCAGTGGCACTAACGGAGAAGTCATGCCCGGACAGTGGGAGTTCCAAGTCGGTCCAACCGTTGGAATCGCTGCCGCCGATCAGGTCTGGGTCGCTCGTTACATCCTCGAGAGGATCACAGAATTGGCTGGAGTTGTTCTGTCTCTTGACCCAAAACCAATTCCGGGAGATTGGAATGGTGCAGGAGCACACACAAATTACAGTACAAAGTCCATGAGAGAAGATGGAGGGTACGAGATCATAAAGAAAGCGATAGAGAAGCTTGGATTGCGTCACAAGGAACACATCTCTGCTTATGGTGAAGGCAACGAGCGTCGTCTCACTGGCAAACACGAGACTGCCGATATCAACACTTTCTTATGGGGTGTGGCCAACCGTGGGGCATCGATTAGGGTTGGTCGGGACACTGAGCAAGCTGGGAAAGGGTACTTCGAAGATCGTAGGCCAGCGTCCAACATGGATCCCTACACTGTGACCTCCATGATTGCTGAAACTACAATCCTCTGGAAGCCATGAATGAAGAAAACTTGAGCTCCTCCAAGGAACCTCTAATATCAGTTCATGTTCATTCTTCTATTGTCTCGTTCTGTCTTCTTTGTTTAAGTATGTTTGATTTAAACTCTTAGAACTTGTTCTATTTCTATATTATGTTATTATACAAATTCAAATATCAGTTTTAGAATATGAAGAGGATCTGAACTTTAAACTTCTTATAGAAACCAAAATCGCAAGTTGCAAGCTCCTCCGTTACTAGCCAACCCAAACTAGACCGGCCGAATCGACATTTAGCACACTCTATTTGGCCCATTTTAGCTTAATGGACTCAAATTTATAACTTTTAAATATTGAAACTCTGTGCTAAAAATATAATAATTTTGAAACTCTATTATACAATGAACTAACGTACTTAAACAAGAAGTAAGAACACGCAGAGAGTACACTACCAATCAATTTATCTTATGCTACTTTGATTCTCGGAAACATTTTCTTGAAACTTCGATATTTCCTTTCTTTTCGTATTTGGTTTAAATTATTATGACTACTATTCATCTTAAGAAACTAACAAAATGAGTTCAGTTTGGATTCATTTTTCAGAATGTATGGGAAGAGAGTTTATTTTCTGTTCGTCTGACATTTTGTTGAAGTCAGGTATGAAATTAAAAACAAAAAAACTATTAGCTTTAAAGTATTATTTTATTCAAGGTTTGGTTTCTCATAATCGAGTTTAAATCCTAATTTTCTGTCCAGGAAAAAACTTCTAATTTGGTCATCAAAAATTTCAAGATATTTTTAATCAATTTTTCATCTACTTATGATTTAAGATTGACAAAGTAATTTACAGCAGACTAAAATATTAACAGTTCAAAGCTATTACGAAATACGCCGATAGGACTAAAACATTTTTTTTCTAAAACATAGGACTAAAACTTTAATGCCAAAATCTAATGGTTGAGAGCTCTTCTTGCAATTCCGTGTTAAAACTTTCATTCATGTAACTAACTGGTAGTATACATCAGTTACCCATACAAGTTAAAGTAAAAAATCTTTATGTTAAGAACTTAAGATTGACATGCATGCATGTTTACATGTGTATATAAACACACACTTAGTTTGGAATTAAAGAAAAACACACATGTATATAAAGAATTGTTTGATCGTCTTTCTCCGAATAAGAGGCGAAACATAATCAACTTGAATTCGGGACAGCTATTCGAAATCTTAGTGAAAATTTTGATTTGTTATCTCATGTTTGCTTTTCGTGAAAAAATACCAATTGAAGTGGAGGGTTTCTTCAAACAACAAGGACCATAAACACACACACCTAGTTTGGAATTAAAGAAAAAAACACACACGCATATAGTTTAGGAGAAGGTTTGTTAACTGTGAAGTGAATGCGCCGAAGTTTTTTAAAAGTAAAAAGATAAGTAAATGCTTCAAGGATTGAAATAAA

>mRNA.BnaC.GLN1.4.b

TATATATACACTTGCAGGAATCTCTTTAGTGTTATACAAAACAAAGCTAATATTTTTTTTTAGATTCCTAGAGAAAATGTCGGCACTTGCAGATTTGATCAATCTCGATCTCTCCGATTACACTGAGAAGATCATTGCGGAGTACATATGGATTGGTGGATCAGGCTTGGATATGAGAAGCAAAGCAAGGACTTTGCCCGGACCAGTGAAGGATCCATCGGAGTTACCGAAATGGAACTATGACGGTTCAAGCACCGGCCAAGCTCCCGGCAGTGACAGTGAAGTCATCCTCTACCCTCAAGCTATCTTCAAAGACCCCTTCAGAAGAGGCAACAACATCCTTGTAATGTGTGACGCATATACACCGGCCGGTGAACCGATTCCGACGAACAAAAGGCATGCTGCAGCTAAGATCTTTAGCGACCCCAGCGTTGCCGCCGAAGAAACATGGTATGGAATTGAGCAAGAGTATACTTTGCTTCAAAAGGATATTAAGTGGCCGGTAGGTTGGCCTGTTGGCGGCTTCCCAGGTCCTCAGGGACCGTACTACTGTGGAGTTGGAGCAGACAAAGCCTTTGGAAGAGACATAGTAGATTCTCATTACAAAGCCTGTCTTTACGCCGGAATCAATGTCAGTGGCACTAACGGAGAAGTCATGCCCGGACAGTGGGAGTTCCAAGTCGGTCCAACGGTTGGAATCGCTGCCGCCGATCAAGTCTGGGTCGCTCGTTACATCCTCGAGAGGATCACAGAATTGGCTGGAGTTGTTTTATCTCTTGACCCTAAACCAATTCCGGGAGATTGGAATGGTGCAGGAGCGCACACAAATTACAGTACAAAGTCGATGAGAGAAGATGGAGGGTACGAGGTGATAAAGAAAGCGATAGAGAAGCTTGGATTGCGTCACAAGGAACACATCTCTGCTTATGGTGAAGGCAACGAGCGTCGCCTCACTGGCAAACACGAGACTGCCGATATCAACACTTTCTTATGGGGTGTGGCCAACCGTGGGGCATCGATTAGAGTTGGTCGGGACACTGAGCAAGCTGGGAAAGGGTACTTTGAAGATCGTAGGCCAGCGTCCAACATGGATCCGTACACTGTGACCTCCATGATTGCTGAAACTACAATCCTCTGGAAGCCATGAATGAAGAAAACTCGAGCTTCAAGGAACCTCTAATATCAGTTCATGTGTCATTCTTATATTGTCTCGTTCTGTCTTCTTTGTTTAAGTGTGTTTGATTTAAACTCTTAGAACTTGTTCTATTTCTATATTATGTTATTACAAATTCAAATATCAGTTTTAGAATATGAAGAGGATCTGAACTTTAAACTTTTTATAGAAACCAAAATCGCAAGTTGCAAGCTCCTCCGTTACTAGCCAACCCAAACTAGACCGGCCGAATCGACATTTAGCACACTCTATTTGGCCCATTTTAGCTTAATGGACTCAAATTTATAACTTTTAAATATTGAAACTCTGTGCAAAAAAAA

>mRNA.BnaA.GLN1.5.a

ATATAAAAAGTGAGTGAGCGAAGCAGAGTGAGCCAGTGCTCACCTCAGACTGATTATTATAACTCGATCGTCATCTTCTTCGGCTTGATGGAAACAGAAAAAATGTCTCCACTCTCCGATCTCCTAAACCTCAATCTCGACACCAAGCAAATCATCGCTGAATACATATGGATCGGTGGGTCTGGAATGGACATTAGAAGCAAAGGCAGGACATTACCAGGACCAGTAAGTGATCCATCAAAGCTTCCGAAATGGAACTACGATGGATCCAGCACCAATCAAGCCGCCGGAGATGACAGTGAAGTCATTCTATATCCTCAGGCGATTTTTAAAGACCCATTCAGGAAAGGGAATAACATTCTCGTGATGTGTGATGCTTACACACCGAAAGGAGATCCAATCCCGACCAACAATAGGCACAAAGCCGTGAAAATCTTCGATCATCCCAATGTGAAGGCTGAAGAGCCTTGGTTTGGGATAGAGCAAGAATACACATTACTTAAGAAAGACGTCAAGTGGCCATTGGGTTGGCCCCTTGGTGGCTTTCCTGGTCCTCAGGGACCGTACTATTGTGCGGTGGGTGCAGACAAAGCCTTTGGGCGTGACATTGTGGATGGTCACTACAAAGCTTGTCTTTACGCTGGTTTAAGCATAGGTGGTGCCAATGGTGAAGTCATGCCTGGTCAATGGGAGTTTCAAATCAGCCCTACTGTTGGTATTGGTGCAGGTGATCAGTTATGGGTTGCTCGCTACATACTTGAGAGGATTACTGAGATATGCGGCGTAATTGTCTCATTTGATCCCAAACCAATCGAGGGTGATTGGAACGGAGCAGCTGCTCATACAAACTTCAGTACAAAATCAATGAGGAAAGAAGGAGGATTGGACTTGATCAAGAAAGCAATAGGGAAGCTTGAAGTGAAGCATAAACAACACATTGCTGCTTATGGTGAAGGCAATGAGAGGCGTCTCACTGGGAAGCATGAAACCGCAGACATCAACAAGTTCTCTTGGGGAGTTGCGGATCGTGGAGCATCGGTGAGAGTGGGAAGAGATACGGAGAAAGAAGGGAAAGGTTATTTTGAAGATCGAAGACCTTCGTCTAATATGGATCCTTATCTTGTTACCTCCATGATAGCTGAAACCACCATCCTCGGCTAAGCTTTCTTTTGAAGTTGTTGCATACGTTCTTTTGTTTCTTCATGTTTCGGTTTAATTTCGGTTTGAGACTTTTTTTTTTATTGCTAATAATTCATGGGATGGTCTTGATCCTATTGTTTGTTTATCCTGGTTCAGTTGTTAGTGTTAAACAAAATTGAATTGGGAAAA

>mRNA.BnaC.GLN1.5.a

TATATAAAAAGTGAGTTAGCGAAGCAGAGTGAGCCAATGCTCACCTCAGACTGATTATTATAACTCGATCGTCTTCTTCTTCGGCTTGATGGAAACAGAAAGAATGTCTCCACTCTCAGATCTCCTGAACCTCAACCTCGACACCAAGCAAATCATCGCTGAATACATATGGATCGGTGGGTCTGGAATGGACATTAGAAGCAAAGGCAGGACATTACCAGGACCTGTAAGTGATCCATCAAAGCTTCCGAAATGGAACTACGATGGATCCAGCACCAATCAAGCCGCCGGAGATGACAGTGAAGTCATTCTATATCCTCAGGCGATTTTTAAAGACCCGTTCAGGAAAGGGAATAACATTCTCGTGATGTGTGATGCTTACACACCGAAAGGAGATCCAATCCCGACCAACAATAGGCACAAAGCCGTGAAAATCTTCGATCATCCCAATGTGAAGGCTGAAGAGCCTTGGTTTGGGATAGAGCAAGAATACACATTACTTAAGAAAGACGTCAAGTGGCCATTGGGTTGGCCCCTTGGTGGCTTTCCTGGTCCTCAGGGACCGTACTATTGTGCGGTCGGTGCAGACAAAGCCTTTGGTCGTGACATTGTGGATGGTCACTACAAAGCTTGTCTTTACGCTGGTTTAAGCATAGGTGGTGCCAATGGTGAAGTCATGCCTGGTCAATGGGAGTTTCAAATCAGCCCTACTGTTGGTATTGGTGCAGGTGATCAGTTATGGGTTGCTCGCTACATACTCGAGAGGATTACTGAGATATGCGGCGTGATTGTCTCATTTGATCCCAAACCAATCGAGGGTGATTGGAACGGAGCAGCTGCTCATACAAACTTCAGTACAAAATCAATGAGGAAAGAAGGAGGATTGGACTTGATAAAAAAAGCAATAGGGAAGCTTGAAGTGAAGCATAAACAACACATTGCTGCTTATGGTGAAGGCAATGAGAGGCGCCTCACTGGGAAGCATGAAACCGCAGACATCAACAAGTTCTCTTGGGGAGTTGCGGATCGTGGAGCATCGGTGAGAGTGGGAAGAGATACGGAGAAAGAAGGGAAAGGGTATTTTGAAGATCGAAGGCCTTCGTCTAATATGGATCCTTATCTTGTTACCTCCATGATAGCTGAAACCACCATCCTCGGCTAAGCTTTCGTTTGAAGTTGTTGCATACGTTCTTTTGTTTCTTCATGTTTCGGTTTAATTTCAGTTTGAGACTCTTTTTTTTATTGCTAATAATTCATGGGATGGTCTTGATCCTATTGTTTGTTTATCCTGGTTCAGTTGTTTGTGTTAAAACAAAATTGAATTGGGAAAATTGAATTGGCAAAATAAA
